# Supplementary material for: Genomic analysis of 116 autism families strengthens known risk genes and highlights promising candidates
Source: NPJ Genom Med. 2024 Mar 22;9:21. doi: 10.1038/s41525-024-00411-1 (PMC10959942; doi:10.1038/s41525-024-00411-1)
Supplement: Supplementary file 1 — Supplementary information [file 41525_2024_411_MOESM1_ESM.pdf]

Genomic analysis of 116 autism families strengthens known risk genes and highlights promising candidates

Running title: Genomic analysis in Autism Spectrum Disorder

Marta Viggiano[1,#], Fabiola Ceroni[1,2#], Paola Visconti[3], Annio Posar[3,4], Maria Cristina Scaduto[3], Laura Sandoni[1], Irene Baravelli[1], Cinzia Cameli[1], Magali J. Rochat[5], Alessandra Maresca[6], Alessandro Vaisfeld[7], Davide Gentilini[8,9], Luciano Calzari[9], Valerio Carelli[6,4], Michael C Zody[10], Elena Maestrini[1,✉], Elena Bacchelli[1,✉]

1 Department of Pharmacy and Biotechnology, University of Bologna, Bologna, Italy.

2 Faculty of Health and Life Sciences, Oxford Brookes University, Oxford, UK.

3 IRCCS Istituto delle Scienze Neurologiche di Bologna, UOSI Disturbi dello Spettro Autistico, Bologna, Italy.

4 Department of Biomedical and Neuromotor Sciences, University of Bologna, Bologna, Italy.

5 IRCCS Istituto delle Scienze Neurologiche di Bologna, Functional and Molecular Neuroimaging Unit, Bologna, Italy.

6 IRCCS Istituto delle Scienze Neurologiche di Bologna, Programma di Neurogenetica, Bologna, Italy.

7 Department of Medical and Surgical Sciences, University of Bologna, Bologna, Italy.

8 Department of Brain and Behavioral Sciences, University of Pavia, Pavia, Italy.

9 Bioinformatics and Statistical Genomic Unit, IRCCS Istituto Auxologico Italiano, Milan, Italy.

10 New York Genome Center (NYGC), New York, NY, USA.

[#] These authors contributed equally to this work

✉ Correspondence:

- Elena Maestrini: elena.maestrini@unibo.it
- Elena Bacchelli: elena.bacchelli@unibo.it

The contents of supplementary materials

Supplementary Data: Clinical description of probands with *BRSK2*, *SCN3A* and *NFIX de novo* variants

Supplementary References

Supplementary Figures:

- Supplementary Figure 1. Age of onset of first symptoms and age of diagnosis of ASD individuals, stratified by sex
- Supplementary Figure 2. Autism severity in the entire cohort of 144 probands and stratified by family type (SPX/MPX)
- Supplementary Figure 3. Score distribution of the Broad Autism Phenotype Questionnaire (BAPQ) in parents and the Social and Communication Disorders Checklist (SCDC) in the entire cohort
- Supplementary Figure 4. Analysis workflow
- Supplementary Figure 5. Genotype-computed ancestry of individuals included in this study
- Supplementary Figure 6. Rate of rare coding *de novo* and inherited pdSNVs in ASD individuals and unaffected siblings
- Supplementary Figure 7. Enrichment for *de novo* and inherited novel pdSNVS in SynGO Genes
- Supplementary Figure 8. Contribution of *de novo* and inherited novel pdSNVs to high confidence ASD/NDD genes.
- Supplementary Figure 9. *De novo* large duplication in Family 9
- Supplementary Figure 10. Multiple hits in families with CNVs in genomic disorders loci
- Supplementary Figure 11. PRS analysis.
- Supplementary Figure 12. *SCN3A de novo* stop-gain variant in Family 40
- Supplementary Figure 13. *HOMER1* deletion in Family 82
- Supplementary Figure 14. Facial photographs of probands with *BRSK2*, *SCN3A* and *NFIX de novo* variants

Supplementary Tables:

- Supplementary Table 1. Summary of phenotype information for individuals with ASD
- Supplementary Table 2. Analysis of subclinical features using BAPQ and SCDC scores
- Supplementary Table 3. Distribution of rare *de novo* variants
- Supplementary Table 4. List of *de novo* pdSNVs identified in affected individuals (extended version of Table 1)
- Supplementary Table 5. Gene ontology from 17 genes carrying the 19 most severe *de novo* pdSNVs identified in 144 cases
- Supplementary Table 6. List of *de novo* and inherited pdSNVs in cases
- Supplementary Table 7. SynGO Gene Set Enrichment Analysis (GSEA) of genes carrying *de novo* and inherited pdSNVs in 144 cases

- Supplementary Table 8. List of high-confidence ASD/NDD genes
- Supplementary Table 9: Comorbidities of probands with pdSNVs in the list of 684 high-confidence ASD/NDD genes
- Supplementary Table 10: Rare genic CNVs in affected individuals and SLD siblings
- Supplementary Table 11: Rare homozygous and compound heterozygous pdSNVs
- Supplementary Table 12: Rare hemizygous pdSNVs

Supplementary Data: Clinical description of probands with *BRSK2*, *SCN3A* and *NFIX de novo* variants

Proband 14.4

The patient is an 11-year-old twin boy with ASD, diagnosed at 3 years of age. Dichorionic diamniotic pregnancy was complicated by threatened miscarriage since the end of the first trimester. Caesarean section was performed at 38 weeks. Birth weight was 2900 g.

After birth, the baby showed mild hypotonia and hypoglicemia, which gradually resolved in the first days of life. Motor milestones were only slightly delayed, and he walked unsupported at 18-20 months. Verbal skills were severely impaired: first words were reported at 2 years of age without any further improvement in the subsequent months. Due to poor eye contact, he was first referred to a child neurologist at the age of 2 years and got ASD diagnosis at the age of 3 years. Despite medical intervention, language improvements have not been reported until the age of 8-9 years, however in the last few years he acquired the ability to speak in short but clear sentences. Other remarkable findings included motor stereotipies, inability to cope with frustration, delayed fine motor skills and mild myopia (-2 D). No anomalies were detected at EEG, ECG and brain MRI.

He was born from healthy, non-consanguineous parents. His dizygotic twin brother (14.3) also has an ASD diagnosis, with poorer performances in speech fluency and autonomy in daily life activities. The remaining family history is unremarkable. Both brothers underwent CMA and *FMR1* genetic analyses, with no abnormalities detected. WGS identified a *de novo BRSK2* heterozygous variant (NP_001243556.1:p.(Ala158Thr)) in patient 14.4, not shared with his twin brother.

On the last evaluation, at the age of 11 years and 2/12, height was 152 cm (85thpercentile) and OFC 52 cm (20th percentile). His speech abilities were improved from the previous evaluation, although inadequate for his age, likewise for fine motor control. No major dysmorphisms were noted, however trigonocephaly, wide mouth with thick lips, bilateral broad hallux and a supernumerary right nipple, just below the normal one, were reported (Supplementary Figure 14a). At the time of our last examination, puberal development had just begun.

Proband 32.3

The patient is a 10 years old boy with ASD, diagnosed at 3 years of age. He was born from healthy non consanguineous parents at 38 weeks after an uneventful pregnancy. Birth weight was 3850 g and neonatal parameters were normal.

Motor development was regular, he walked at age 13 months. At 18 months his parents noted a delay in verbal skills, poor eye contact and stereotyped behaviors, but only at 3 years he was diagnosed with ASD. His nonverbal IQ, evaluated at 4 years by Leiter-R test, was normal. Despite rehabilitation interventions he has important language problems (few single words). Maternal and paternal family history is positive for language disorders. Other remarkable findings included motor stereotypies, frustration intolerance and agitation. No major dysmorphisms were noted. ABR, EEG and brain MRI were all normal.

CMA and *FMR1* analyses were performed, with no abnormalities detected. WGS revealed a *de novo BRSK2* frameshift variant (NP_001243556.1:p.(Asp540GlufsTer9)).

Proband 40.3

The patient is a 12-year-old twin boy with ASD, diagnosed at 4 years of age. Dichorionic diamniotic pregnancy was uneventful, caesarean section was performed at 39 weeks. Birth weight was 2900 g and neonatal parameters were normal.

Motor development was regular, he walked unsupported at age 10 months. At 18 months, his parents noted a mild delay in verbal skills, but only at 3 years he was referred to child neurologist and he was diagnosed with ASD at the age of 4 years. His IQ, evaluated at 4 years through Leiter-R test, was 111. After 5 years of age, he started composing complex sentences. No anomalies were detected at EEG and brain MRI.

He was born from healthy, non-consanguineous parents. His dizygotic twin brother (40.4) also has an ASD diagnosis, with no ID, but a less structured language. EEG and MRI analyses did not reveal any anomalies. The remaining family history is unremarkable.

CMA and *FMR1* analyses were performed on both twins, with no abnormalities detected. WGS revealed a *de novo SCN3A* variant (NP_008853.3:p.(Arg1630Ter)), not shared with his twin.

At the last evaluation, at the age of 11 years and 8 months, his height was 150 cm (64th percentile) and OFC 53 cm (35th percentile). No major dysmorphisms were noticeable (Supplementary Figure 14b). We only noted mild kyphosis, bilateral clinodactyly of the 5th finger, bilateral syndactyly of 2nd-3rd toes.

Proband 81.3

The patient is a 7 years old boy, with a clinical history of global developmental delay and ASD. He was born at 41 weeks after an uneventful pregnancy. Birth weight was 3300 g, length 52 cm and OFC 35 cm.

At 8 days of life, he started showing episodes of generalized pallor with perioral cyanosis, hypotonia and apneas. EEG and ECG were performed with normal results. Cerebral US detected ventricular asymmetry (left>right). The episodes were attributed to gastroesophageal reflux disease (GERD) and became milder and less frequent with anti-reflux therapy. At 15 months of age, a cluster of 3 seizures occurred, characterized by eye deviation, hypertonia and myoclonus, during an episode of gastroenteritis. Since the age of 6 years, the child has presented focal epileptic motor seizures with or without secondary generalization. Currently he is taking Valproic Acid. EEG shows epileptic abnormalities in right central-parietal regions. Brain MRI (3 Tesla) was normal.

Motor milestones were regularly acquired, while verbal skills were delayed. Due to absent language, poor eye contact and stereotyped behaviors, he was referred to child neurologist at the age of 2 years, where developmental delay and ASD diagnosis was diagnosed at the age of 2 years and 10 months. Macrocephaly (OFC> +2 SD) was reported since then. Despite speech therapy, language is still absent with the emission of only mono-syllabic sounds. Severe ID is present.

He was born from non-consanguineous parents. He has an older healthy brother. Family history is positive for nonfebrile seizures (mother and maternal grandmother), eventually resolved without sequelae.

He first underwent FXS analysis, with detection of a normal *FMR1* allele. CMA was then performed, with the detection of a large duplication 15q13.2-13.3, also confirmed by SNP array and WGS data. The duplication is inherited from the mother, who has a personal and family history of uncomplicated seizures. In addition to this recurrent CNV, WGS detected a *de novo NFIX* heterozygous variant (NP_001352831.1:p.(Leu75Pro)) in the proband, providing a clinical diagnosis of Malan syndrome. This result also confirmed that the duplication was not the main cause of the patient’s condition, even if we couldn’t exclude a partial contribution to his complex phenotype.

At the last evaluation, at the age of 7 years and 9 months, height was 129 cm (65th percentile) and OFC 56,9 (+3.5 SD), indicating the presence of macrocephaly. In addition, he had dolicocephaly with a wide forehead, low set eyes, high-arched and narrow palate (Supplementary Figure 14c). As collateral findings, we also reported a supernumerary left nipple and two cafè-au-lait spots, one in the back between scapulae and one in the right forearm.

Supplementary References

1. Sasson, N.J. et al. The broad autism phenotype questionnaire: prevalence and diagnostic classification. *Autism Res* **6**, 134-143 (2013).
2. Skuse, D.H., Mandy, W.P., Scourfield, J. Measuring autistic traits: heritability, reliability and validity of the Social and Communication Disorders Checklist. *Br J Psychiatry* **187**, 568-72 (2005).
3. Fu, J.M. et al. Rare coding variation provides insight into the genetic architecture and phenotypic context of autism. *Nature Genetics* **54,** 1320-1331 (2022).
4. Trost, B. et al. Genomic architecture of autism from comprehensive whole-genome sequence annotation. *Cell* **185**, 4409-4427.e4418 (2022).
5. Satterstrom, F. K. et al. Large-Scale Exome Sequencing Study Implicates Both Developmental and Functional Changes in the Neurobiology of Autism. *Cell* **180**, 568-584.e523 (2020).
6. Dickinson, M. E. et al. High-throughput discovery of novel developmental phenotypes. *Nature* **537**, 508-514 (2016).
7. Lamar, T. et al. SCN3A deficiency associated with increased seizure susceptibility. *Neurobiol Dis* **102**, 38-48 (2017).
8. Planells-Cases, R. et al. Neuronal death and perinatal lethality in voltage-gated sodium channel alpha(II)-deficient mice. *Biophys J* **78**, 2878-2891 (2000).
9. Li, M. et al. Antisense oligonucleotide therapy reduces seizures and extends life span in an SCN2A gain-of-function epilepsy model. *J Clin Invest* **131** (2021).
10. Costantini, D.L. et al. The homeodomain transcription factor Irx5 establishes the mouse cardiac ventricular repolarization gradient. *Cell* **123**, 347-358 (2005).
11. Kohl, S. et al. A duplication on chromosome 16q12 affecting the IRXB gene cluster is associated with autosomal dominant cone dystrophy with early tritanopic color vision defect. *Hum Mol Genet* **30**, 1218-1229 (2021).
12. Peça, J. et al. Shank3 mutant mice display autistic-like behaviours and striatal dysfunction. *Nature* **472**, 437-442 (2011).
13. Wang, X. et al. Synaptic dysfunction and abnormal behaviors in mice lacking major isoforms of Shank3. *Hum Mol Genet* **20**, 3093-3108 (2011).
14. Bidinosti, M. et al. CLK2 inhibition ameliorates autistic features associated with SHANK3 deficiency. *Science* **351**, 1199-1203 (2016).
15. Mei, Y. et al. Adult restoration of Shank3 expression rescues selective autistic-like phenotypes. *Nature* **530**, 481-484 (2016).
16. Han, K. et al. SHANK3 overexpression causes manic-like behaviour with unique pharmacogenetic properties. *Nature* **503**, 72-77 (2013).
17. Cox, G.A. et al. Sodium/hydrogen exchanger gene defect in slow-wave epilepsy mutant mice. *Cell* **91**, 139-148 (1997).
18. Kröll-Hermi, A. et al. Proteasome subunit PSMC3 variants cause neurosensory syndrome combining deafness and cataract due to proteotoxic stress. *EMBO Mol Med* **12**, e11861 (2020).
19. Hafezparast, M. et al. Mutations in dynein link motor neuron degeneration to defects in retrograde transport. *Science* **300**, 808-812 (2003).
20. Braunstein, K.E. et al. A point mutation in the dynein heavy chain gene leads to striatal atrophy and compromises neurite outgrowth of striatal neurons. *Hum Mol Genet* **19**, 4385-4398 (2010).

21. Eschbach, J. et al. Dynein mutations associated with hereditary motor neuropathies impair mitochondrial morphology and function with age. *Neurobiol Dis* **58**, 220-230 (2013).
22. Ori-McKenney, K. M., Vallee, R. B. Neuronal migration defects in the Loa dynein mutant mouse. *Neural Dev* **6**, 26 (2011).
23. Driller, K. et al. Nuclear factor I X deficiency causes brain malformation and severe skeletal defects. *Mol Cell Biol* **27**, 3855-3867 (2007).
24. Campbell, C.E. et al. The transcription factor Nfix is essential for normal brain development. *BMC Dev Biol* **8**, 52 (2008).
25. Harris, L. et al. Heterozygosity for nuclear factor one x affects hippocampal-dependent behaviour in mice. *PLoS One* **8**, e65478 (2013).
26. Zhou, B. et al. Loss of NFIX Transcription Factor Biases Postnatal Neural Stem/Progenitor Cells Toward Oligodendrogenesis. *Stem Cells Dev* **24**, 2114-2126 (2015).
27. Paul, M.S., Duncan, A.R., Genetti, C.A., et al. Rare EIF4A2 variants are associated with a neurodevelopmental disorder characterized by intellectual disability, hypotonia, and epilepsy. *Am J Hum Genet* **110**, 120-145 (2023).
28. Kobayashi, T., Minowa, O., Sugitani, Y., et al. A germ-line Tsc1 mutation causes tumor development and embryonic lethality that are similar, but not identical to, those caused by Tsc2 mutation in mice. *Proc Natl Acad Sci USA* **98**, 8762-8767 (2001).
29. Kwiatkowski, D.J., Zhang, H., Bandura, J.L., et al. A mouse model of TSC1 reveals sex-dependent lethality from liver hemangiomas, and up-regulation of p70S6 kinase activity in Tsc1 null cells. *Hum Mol Genet* **11**, 525-534 (2002).
30. Uhlmann, E.J., Apicelli, A.J., Baldwin, R.L., et al. Heterozygosity for the tuberous sclerosis complex (TSC) gene products results in increased astrocyte numbers and decreased p27-Kip1 expression in TSC2+/- cells. *Oncogene* **21**, 4050-4059 (2002).
31. Goorden, S.M., van Woerden, G.M., van der Weerd, L., Cheadle, J.P., Elgersma, Y. Cognitive deficits in Tsc1+/- mice in the absence of cerebral lesions and seizures. *Ann Neurol* **62**, 648-655 (2007).
32. Zeng, L.H., Xu, L., Gutmann, D.H., Wong, M. Rapamycin prevents epilepsy in a mouse model of tuberous sclerosis complex. *Ann Neurol* **63**, 444-453 (2008).
33. Abs, E., Goorden, S.M., Schreiber, J., et al. TORC1-dependent epilepsy caused by acute biallelic Tsc1 deletion in adult mice. *Ann Neurol* **74**, 569-579 (2013).
34. Tsai, P.T., Hull, C., Chu, Y., et al. Autistic-like behaviour and cerebellar dysfunction in Purkinje cell Tsc1 mutant mice. *Nature* **488**, 647-651 (2012).
35. Lim, J.S., Gopalappa, R., Kim, S.H. et al. Somatic Mutations in TSC1 and TSC2 Cause Focal Cortical Dysplasia. *Am J Hum Genet* **100**, 454-472 (2017).
36. Ercan, E., Han, J.M., Di Nardo, A., et al. Neuronal CTGF/CCN2 negatively regulates myelination in a mouse model of tuberous sclerosis complex. *J Exp Med* **214**, 681-697 (2017).
37. Hirotsune, S., Fleck, M.W., Gambello, M.J. et al. Graded reduction of Pafah1b1 (Lis1) activity results in neuronal migration defects and early embryonic lethality. *Nat Genet* **19**, 333-339 (1998).
38. Assadi, A.H., Zhang, G., Beffert, U., et al. Interaction of reelin signaling and Lis1 in brain development. *Nat Genet* **35**, 270-276 (2003).
39. Pawlisz, A.S., Mutch, C., Wynshaw-Boris, A., Chenn, A., Walsh, C.A., Feng, Y. Lis1-Nde1-dependent neuronal fate control determines cerebral cortical size and lamination. *Hum Mol Genet* **17**, 2441-2455 (2008).

40. Yamada, M., Yoshida, Y., Mori, D., et al. Inhibition of calpain increases LIS1 expression and partially rescues in vivo phenotypes in a mouse model of lissencephaly. *Nat Med* **15**, 1202-1207 (2009).

41. Greenwood, J.S., Wang, Y., Estrada, R.C., Ackerman, L., Ohara, P.T., Baraban, S.C. Seizures, enhanced excitation, and increased vesicle number in Lis1 mutant mice. *Ann Neurol* **66**, 644-653 (2009).

42. Cahana, A., Escamez, T., Nowakowski, R.S. et al. Targeted mutagenesis of Lis1 disrupts cortical development and LIS1 homodimerization. *Proc Natl Acad Sci USA* **98**, 6429-6434 (2001).

43. Liu, Z., Steward, R., Luo, L. Drosophila Lis1 is required for neuroblast proliferation, dendritic elaboration and axonal transport. *Nat Cell Biol* **2**, 776-783 (2000).

44. Williams, S.N., Locke, C.J., Braden, A.L., Caldwell, K.A., Caldwell, G.A. Epileptic-like convulsions associated with LIS-1 in the cytoskeletal control of neurotransmitter signaling in Caenorhabditis elegans. *Hum Mol Genet* 13, 2043-2459 (2004).

45. Stephen, J., Maddirevula, S., Nampoothiri, S. et al. Bi-allelic TMEM94 Truncating Variants Are Associated with Neurodevelopmental Delay, Congenital Heart Defects, and Distinct Facial Dysmorphism. *Am J Hum Genet* **103**, 948-967 (2018).

46. Leblond, C.S., Le, T.L., Malesys, S. et al. Operative list of genes associated with autism and neurodevelopmental disorders based on database review. *Mol Cell Neurosci* **113**, 103623 (2021).

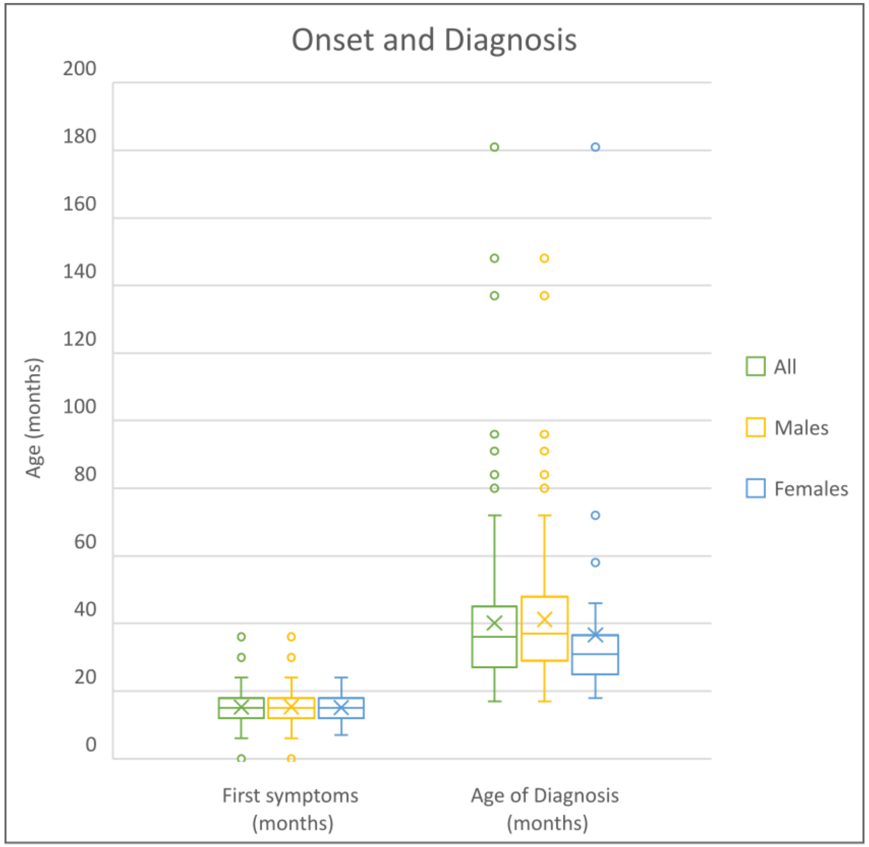

Supplementary Figure 1. Age of onset of first symptoms and age of diagnosis of ASD individuals, stratified by sex.

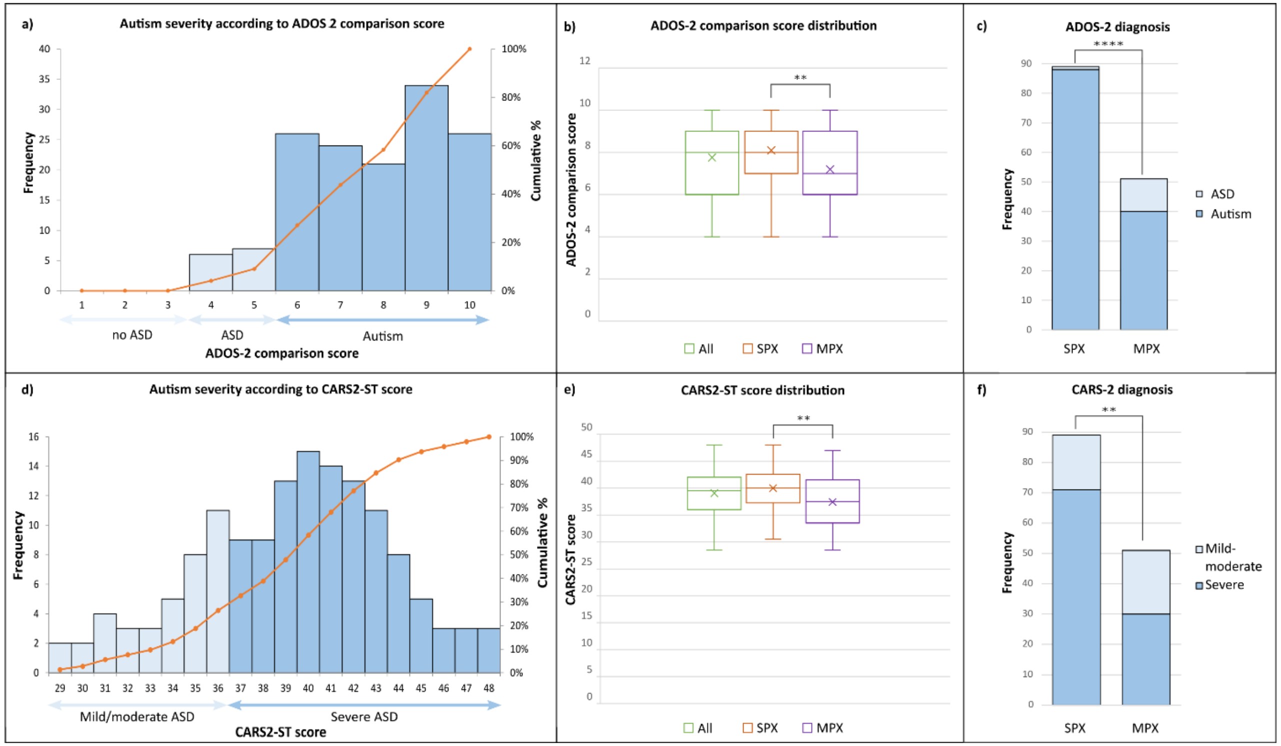

Supplementary Figure 2. Autism severity in the entire cohort of 144 probands and stratified by family type (SPX/MPX). Autism severity in the entire cohort according to ADOS-2 comparison score (a) and to CARS2-ST score (d). ADOS-2 comparison scores 1-3 correspond to non-spectrum related symptoms scores 4-5 correspond to autism-spectrum related symptoms, while scores of ≥6 correspond to autism-related symptoms. CARS total scores <30 indicate that an individual is "non-autistic," scores 30-36.5 indicate "mild to moderate autism" while scores 37-60 correspond to "severe autism". (b) ADOS-2 comparison score and (e) CARS2-ST score distribution in the entire cohort of probands and in probands from SPX and MPX families. The mean ADOS-2 comparison score and CARS2-ST score of simplex probands are significantly higher than multiplex probands (ADOS-2 and CARS2-ST scores two-sided t-test *p*-value =0.005 and 0.001, respectively). Comparison of ADOS-2 (c) and CARS-2 (f) diagnosis categories in SPX and MPX. ADOS-2 "autism" diagnosis and CARS-2 "severe autism" diagnosis are significantly higher in SPX families (ADOS-2 Fisher's Exact Test *p*-value $=6x10^{-5}$ and CARS-2 chi2 test *p*-value = 0.008).

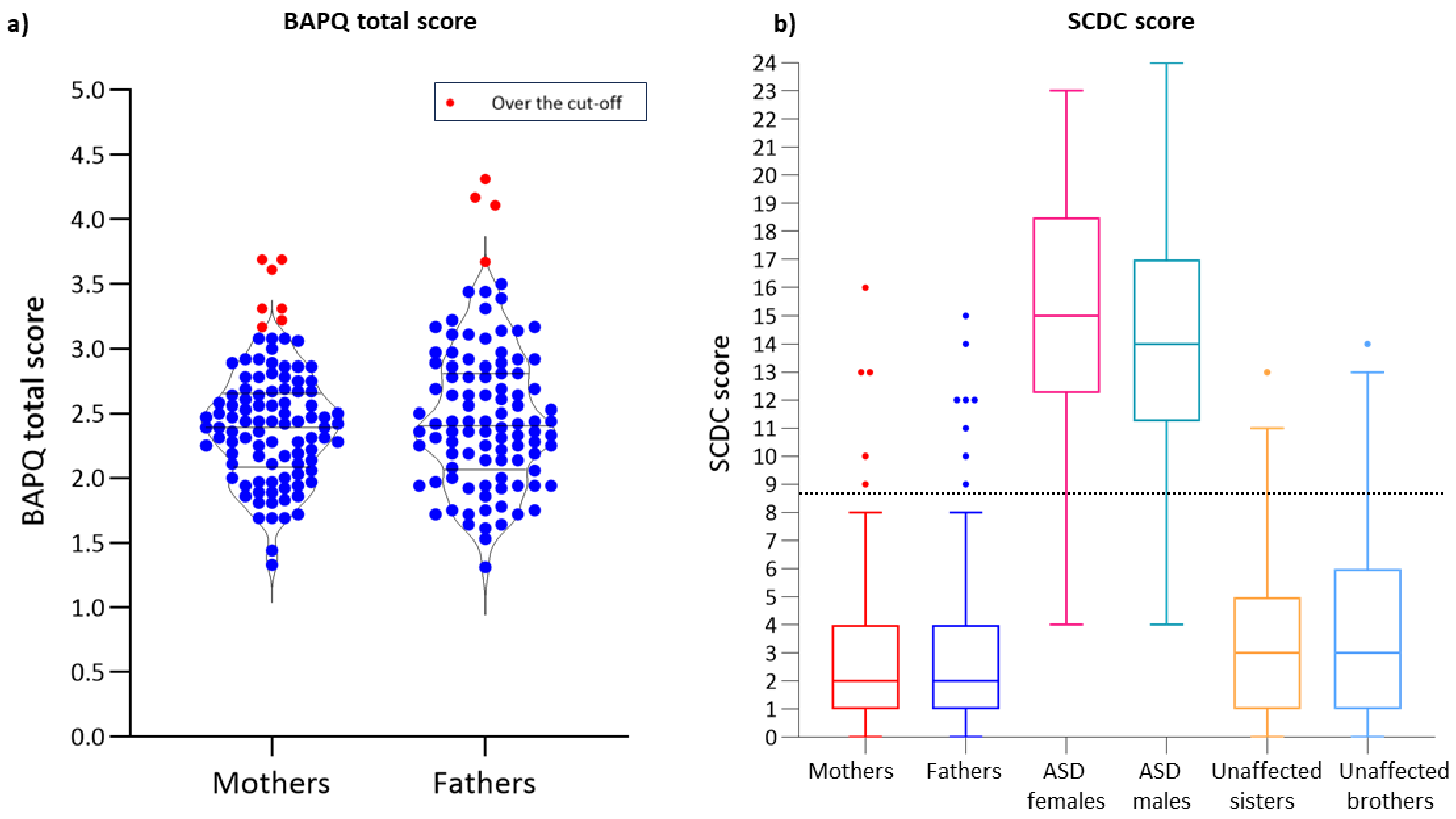

Supplementary Figure 3. Score distribution of the Broad Autism Phenotype Questionnaire (BAPQ) in parents and the Social and Communication Disorders Checklist (SCDC) in the entire cohort. a) BAPQ total score in parents. Red points indicate individuals with BAPQ score over the cutoffs (male threshold= 3.55; female threshold=3.17)[1]. b) Boxplots of SCDC scores in parents, affected individuals and unaffected siblings. The dotted line indicates the suggested cut-off score (a score of 9 or above reflects difficulties in social interaction and communication)[2].

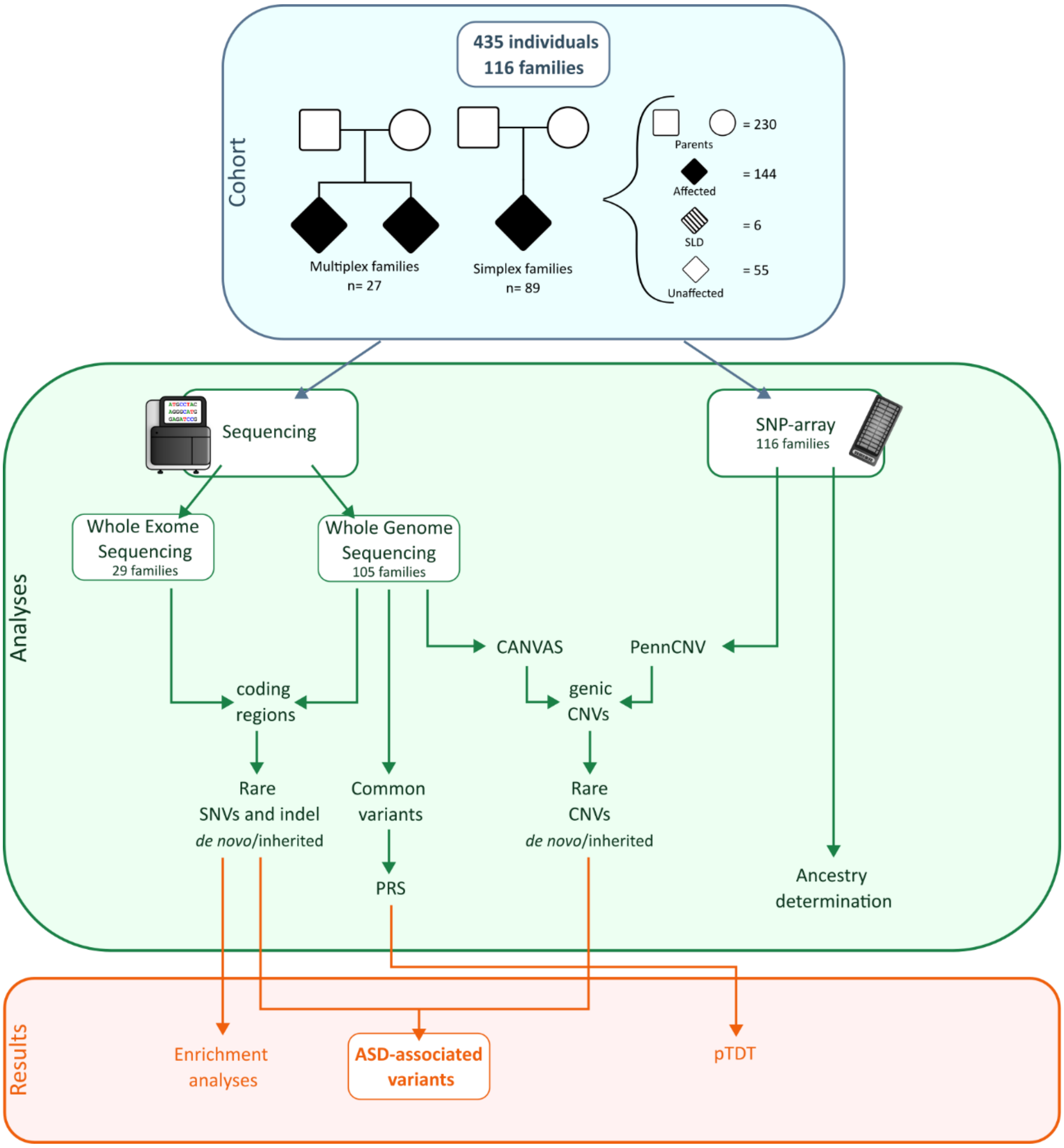

Supplementary Figure 4. Analysis workflow. This workflow shows the cohort composition and the primary analyses performed in this study. 18 families were analysed by both WES and WGS. Filtering steps employed to prioritize rare coding variants that could be associated with ASD susceptibility are described in detail in the Methods.

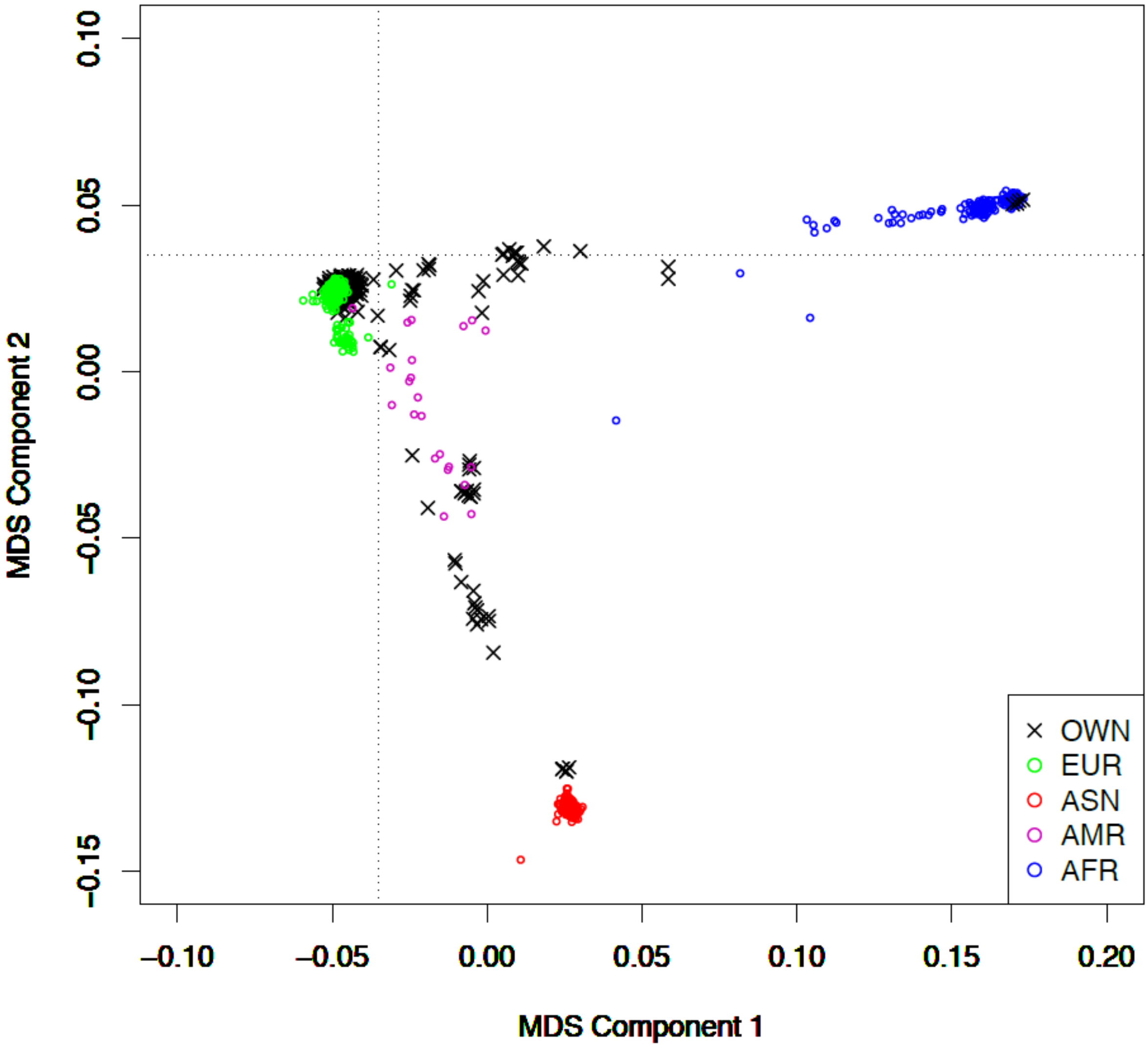

Supplementary Figure 5. Genotype-computed ancestry of individuals included in this study. Multidimensional scaling (MDS) analysis was performed merging our cohort data with the 1000 Genomes Project reference panel (20100804 release). We assigned ancestry labels to all individuals of our cohort through visual inspection of the first two MDS coordinates and confirmed that 19 families are of non-European origin, consistent with self-reported ancestry. Green, red, pink and blue circles indicate European (EUR), Asian (ASN), Admixed American (AMR) and African (AFR) individuals of the 1000G, respectively. Black crosses indicate all individuals included in this study (n=435).

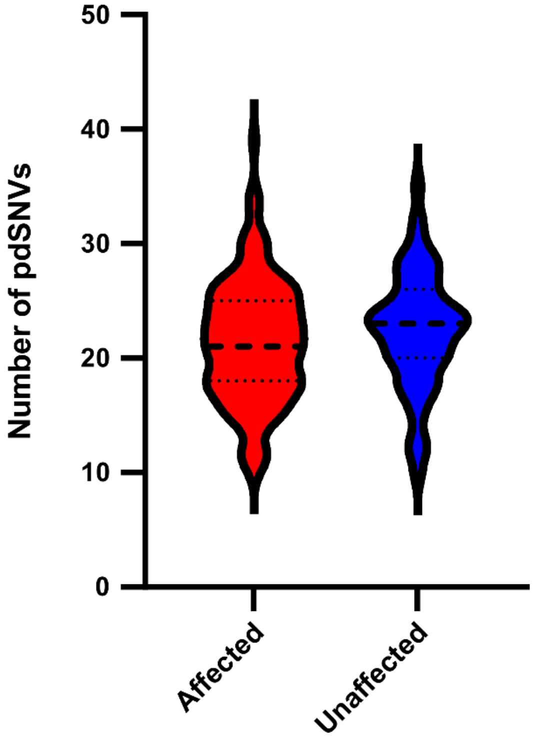

Supplementary Figure 6. Rate of rare coding *de novo* and inherited pdSNVs in ASD individuals and unaffected siblings. Comparison of individual rate of *de novo* and inherited pdSNVs in cases and unaffected siblings (ASD cases=144, unaffected siblings=55). pdSNVs include PTVs in genes with LOEUF>0.6 (PTV_{LOEUF}), missense variants with MPC score ≥2 (DmisB) and missense variants with MPC score 1-2 (DmisA).

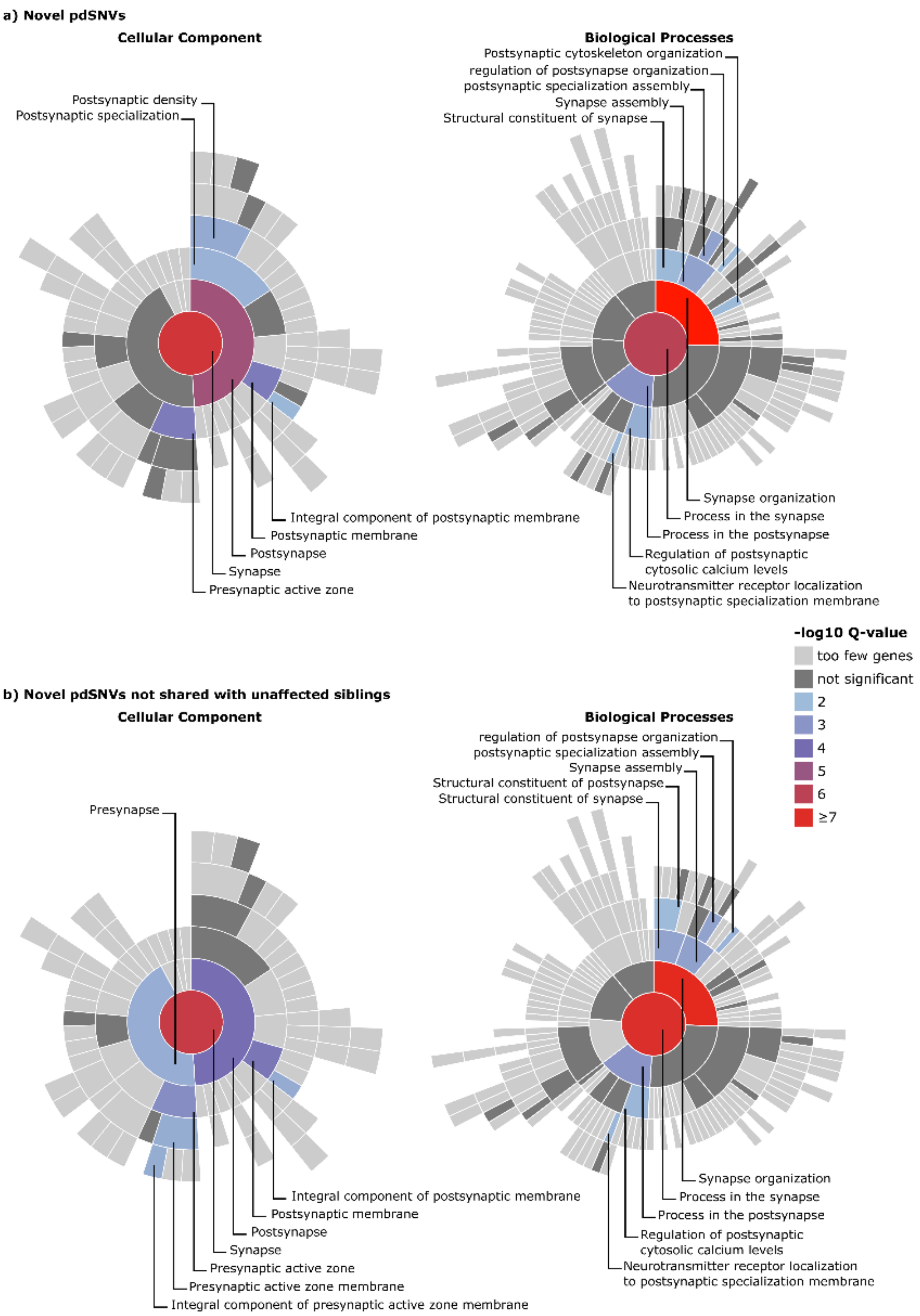

Supplementary Figure 7. Enrichment for *de novo* and inherited novel pdSNVS in SynGO Genes. Visualisation of gene set enrichment analyses (GSEA) of genes harbouring novel pdSNVs (a) and novel pdSNVs not shared with unaffected siblings (b) in affected individuals, each compared to a background set of brain-expressed genes. All Cell Components (CC) or Biological Process (BP) related terms with gene annotations in SynGO are plotted in a circular fashion, with the highest hierarchical term ("synapse" for CC or "process in synapse" for BP) in the centre and each layer of subclasses in outward concentric rings. Over-represented synaptic terms are indicated with different colours, according to the Q-value, and are reported in detail in Supplementary Table 7b-c. Genes harbouring novel pdSNVs and novel pdSNVs not shared with unaffected siblings in cases show an enrichment of synaptic terms comparable to genes harbouring pdSNVs. The four most enriched CC and BP are retained.

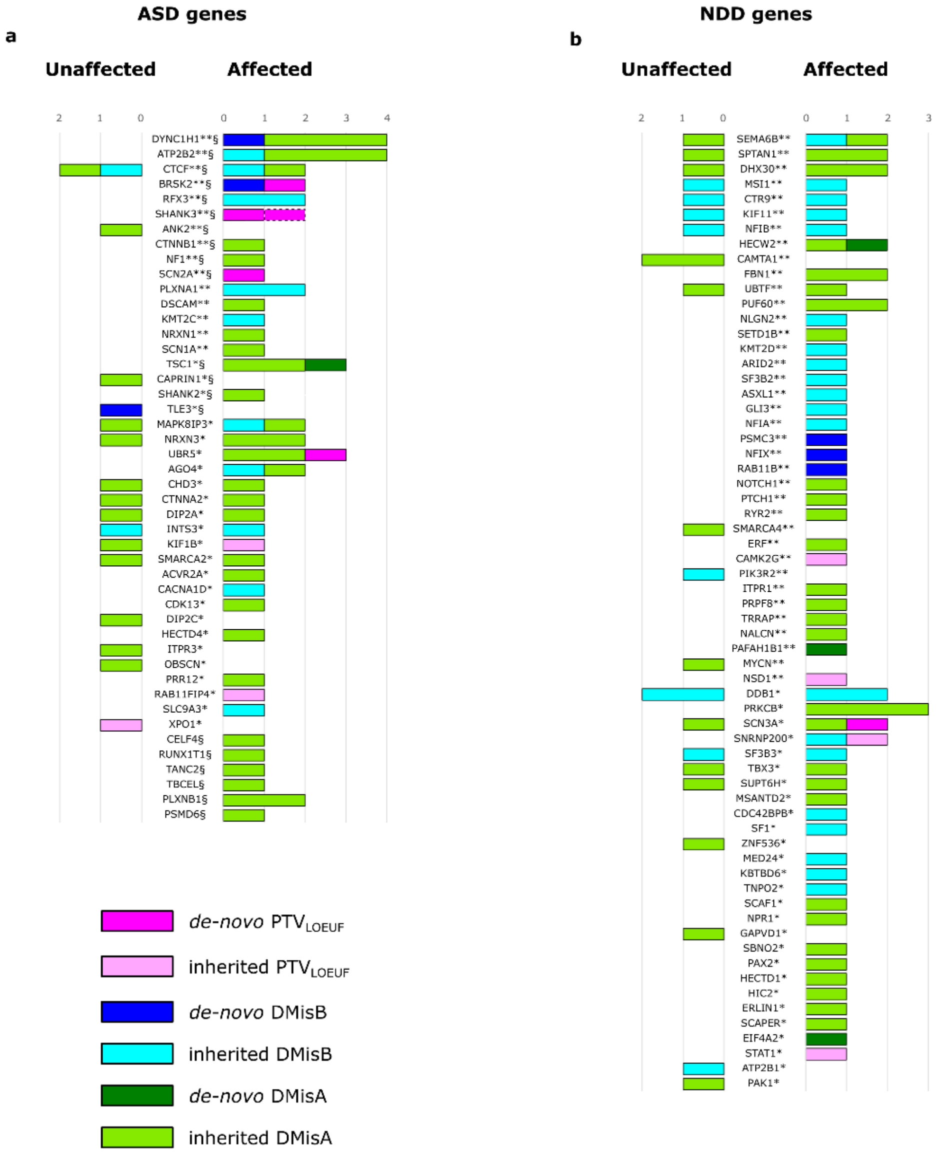

Supplementary Figure 8. Contribution of *de novo* and inherited novel pdSNVs to high confidence ASD/NDD genes. *De novo* and inherited novel pdSNVs include PTVs in genes with LOEUF score <0.6 (PTV_{LOEUF}), missense variants with MPC score ≥2 (DmisB) and missense variants with MPC score 1-2 (DmisA). Contribution of each variant type identified in ASD individuals and unaffected siblings for a list of genes previously associated to ASD (a) and NDD (b). The list of ASD genes comprised 185 genes associated at FDR ≤0.05[3] and 135 genes with FDR <0.1[4] (88 of which were common between the two lists). In our cohort, novel pdSNVs were identified in 46 ASD genes (a). The list of NDD genes included 452 genes from a list of 664 genes associated at FDR ≤0.05, after the exclusion of the genes already included among the 232 ASD genes[3]. In our cohort, novel pdSNVs were identified in 64 NDD genes (b). **, genes with FDR ≤0.001[3]; *, genes with FDR ≤0.05[3]; §, genes with FDR <0.1[4]; dotted line indicates a putative *de novo* PTV_{LOEUF}.

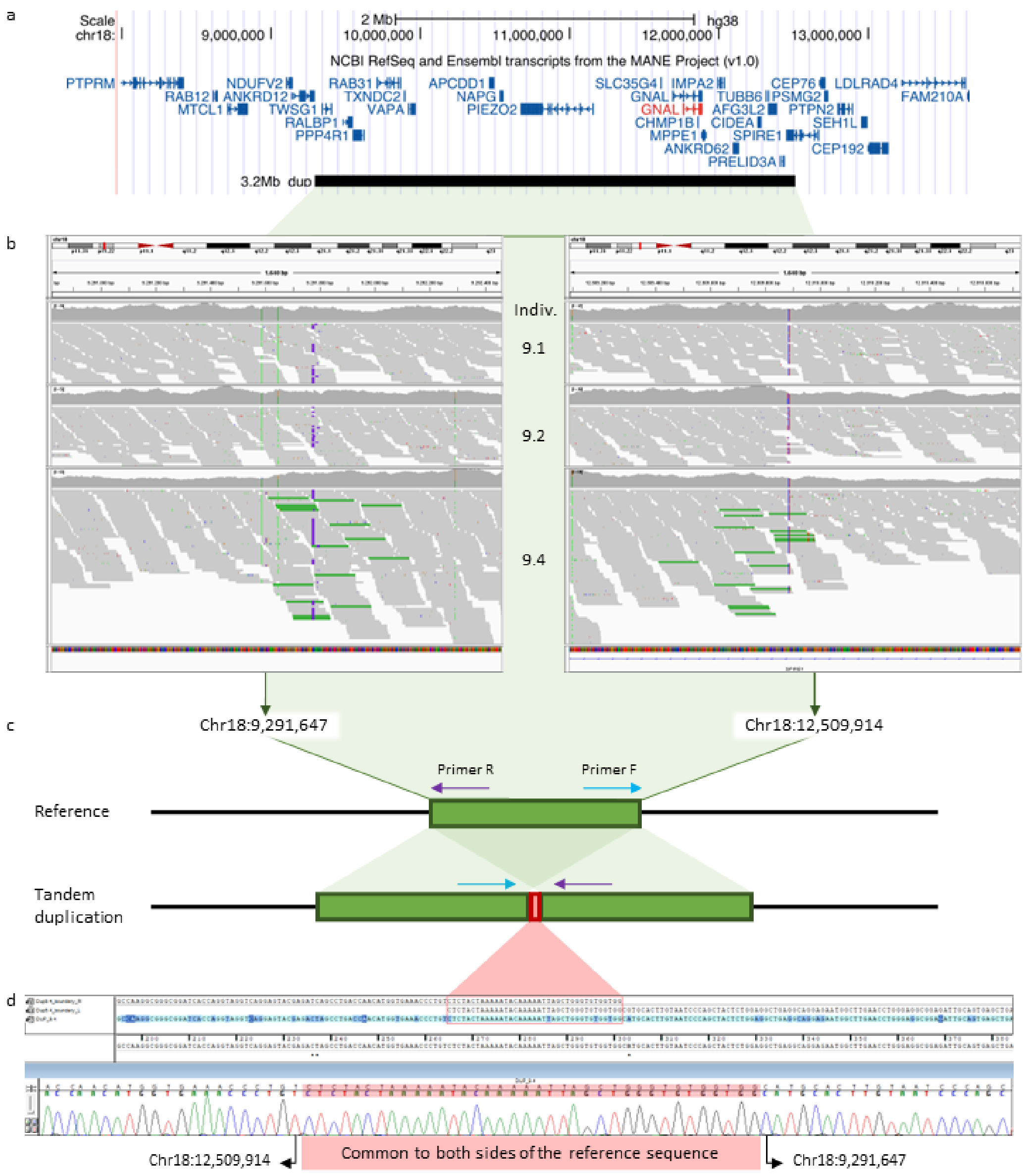

Supplementary Figure 9. ***De novo*** **large duplication in Family 9.** a) UCSC schematic (hg38) showing the 18p11.22-p11.21 region. The 3.2 Mb *de novo* duplication identified in individual 9.4 is indicated by a black bar. The transcripts from the MANE track are shown for each gene. b) IGV visualisation of the breakpoints. Read alignments are shown for the father (9.1), mother (9.2) and SLD child (9.4). The positions of the two clusters of green reads in 9.4 indicate the boundaries of the tandem duplicated fragment. c) Schematic indicating the location of the primers used to validate the position of the breakpoints. Tandem duplicated sequence indicated in green, forward and reverse primers are indicated with blue and purple arrows, respectively. The primers are designed such that in the reference sequence they are divergent (top), whereas in the tandem duplicated allele they are convergent and can generate a PCR product across the junction. d) Chromatogram of the junction between the duplicated segments demonstrating their tandem orientation. The sequence highlighted in red is common to both ends of the duplicated fragment and therefore cannot be definitively ascribed to either.

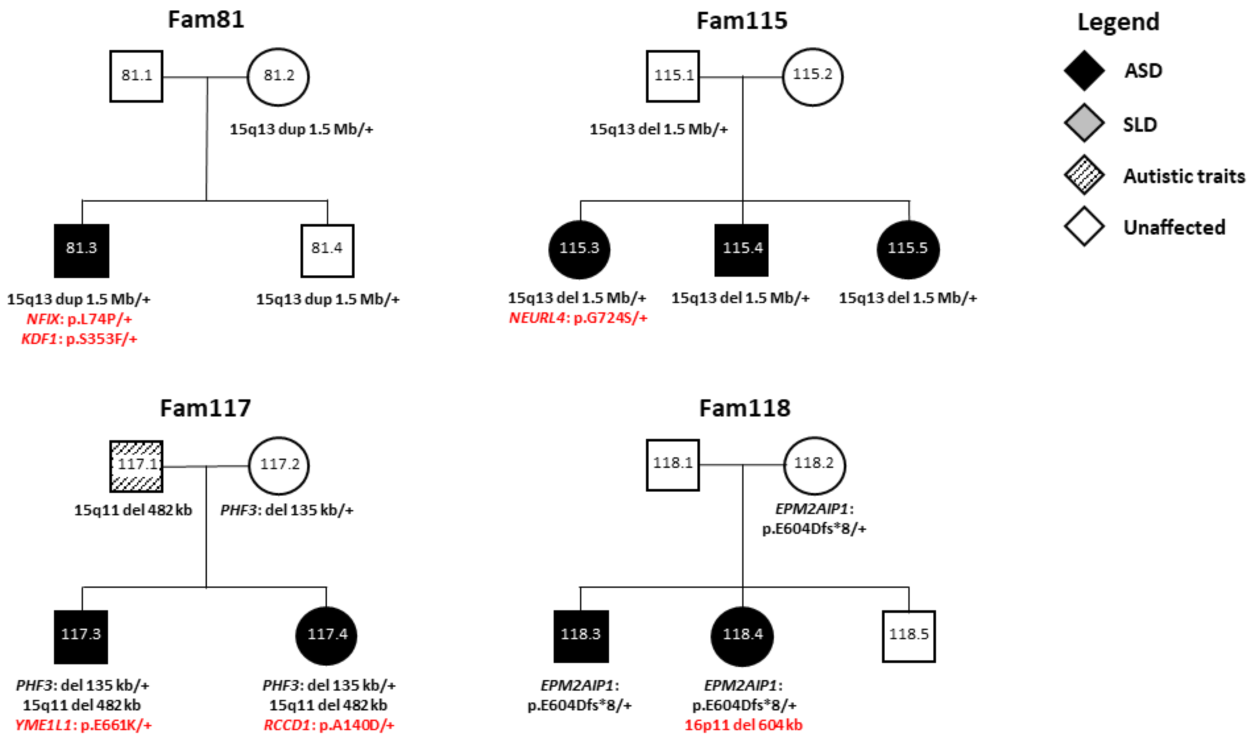

Supplementary Figure 10. Multiple hits in families with CNVs in genomic disorders loci. Pedigrees of families carrying a CNV associated with recurrent genomic disorders (RGD) and additional hits consisting of i) *de novo* pdSNVs, ii) $PTVs_{LOEUF}$ in NDD genes inherited from the parent not transmitting the recurrent CNV, iii) pdCNVs inherited from the parent not transmitting the recurrent CNV (listed in Table 2). *De novo* variants are reported in red. ASD, autism spectrum disorder; SLD, specific learning disabilities; del, deletion; dup, duplication. All HGVS nomenclatures are based on the MANE select transcripts.

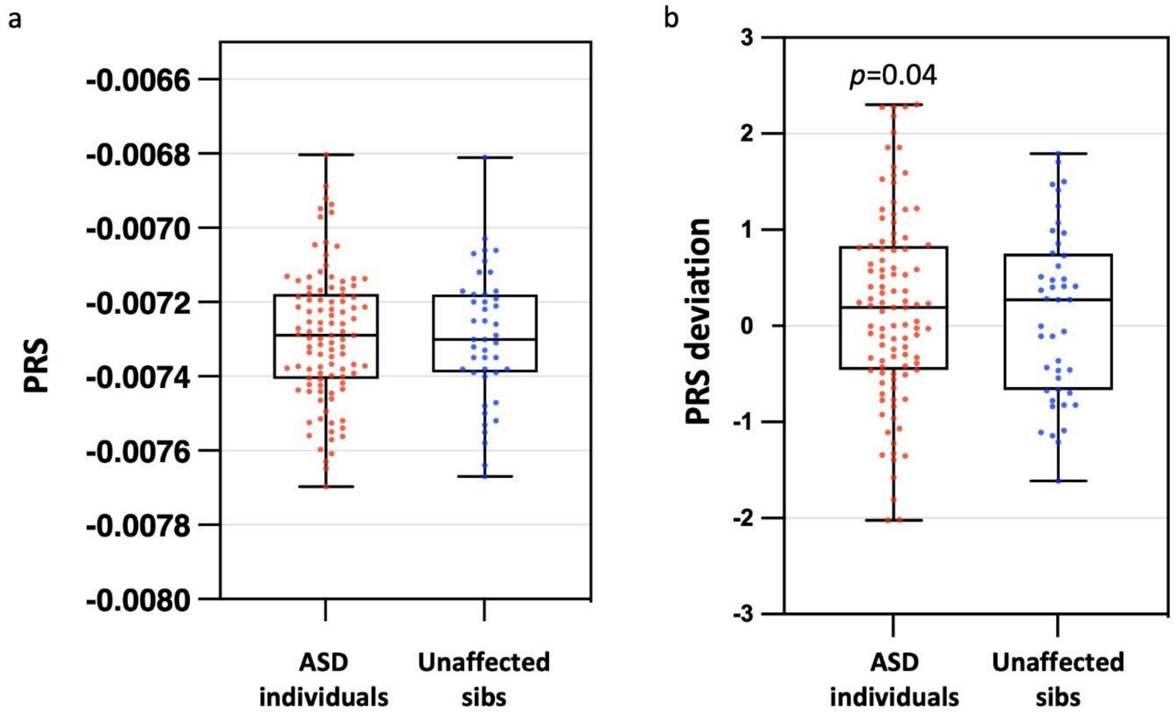

Supplementary Figure 11. PRS analysis. a) PRS distributions in cases (n=103) and unaffected siblings (n=44). b) Polygenic transmission disequilibrium test (pTDT) showed that PRS was significantly over-transmitted in individuals with ASD (pTDT mean=0.20, *p*-value=0.0), but not in non-ASD siblings (pTDT mean=0.11, *p*-value=0.39).

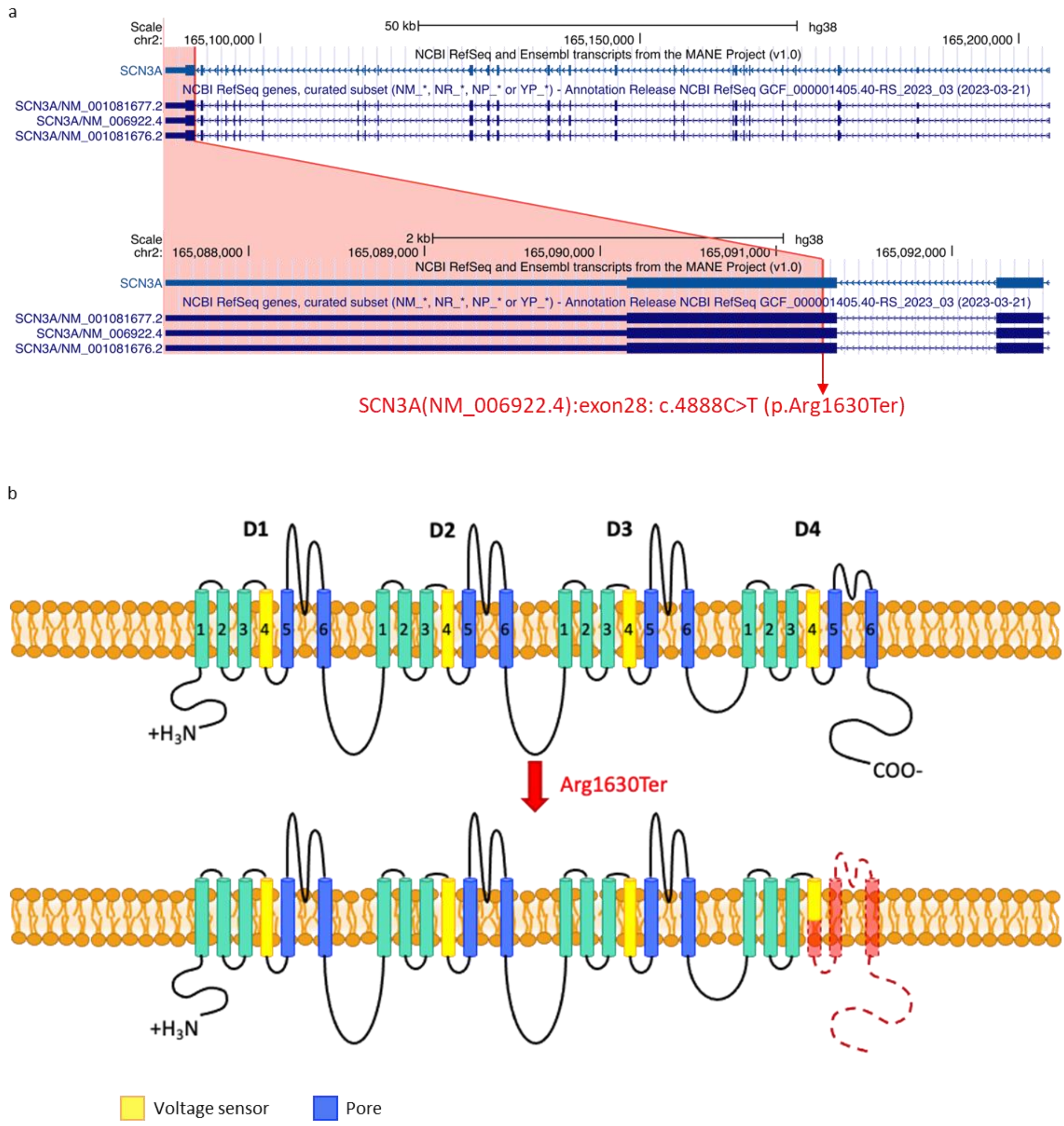

Supplementary Figure 12. *SCN3A de novo* stop-gain variant in Family 40. a) UCSC schematic (hg38) showing the genomic structure of *SCN3A*. The position of the *de novo* stop-gain variant, mapping within the last exon of all Refseq isoforms, is indicated by the red arrow. b) Schematic of the SCN3A protein. *SCN3A* encodes the type III voltage-gated sodium (Na+) α subunit Nav1.3, a transmembrane protein with four domains (D1-D4), each formed by six subunits (S1-6). Subunits 4 (S4) act as "voltage sensor" and transduce the transmembrane voltage into mechanical changes that alter channel gating. Subunits S5-6 form the channel pore. The premature stop-gain identified in our study is predicted to determine the loss of half of S4D4, critical for voltage sensing, the two pore-building segments S5D4 and S6D4, and the entire cytoplasmic C-terminal tail, needed for the protein-protein interaction with one of the β subunits.

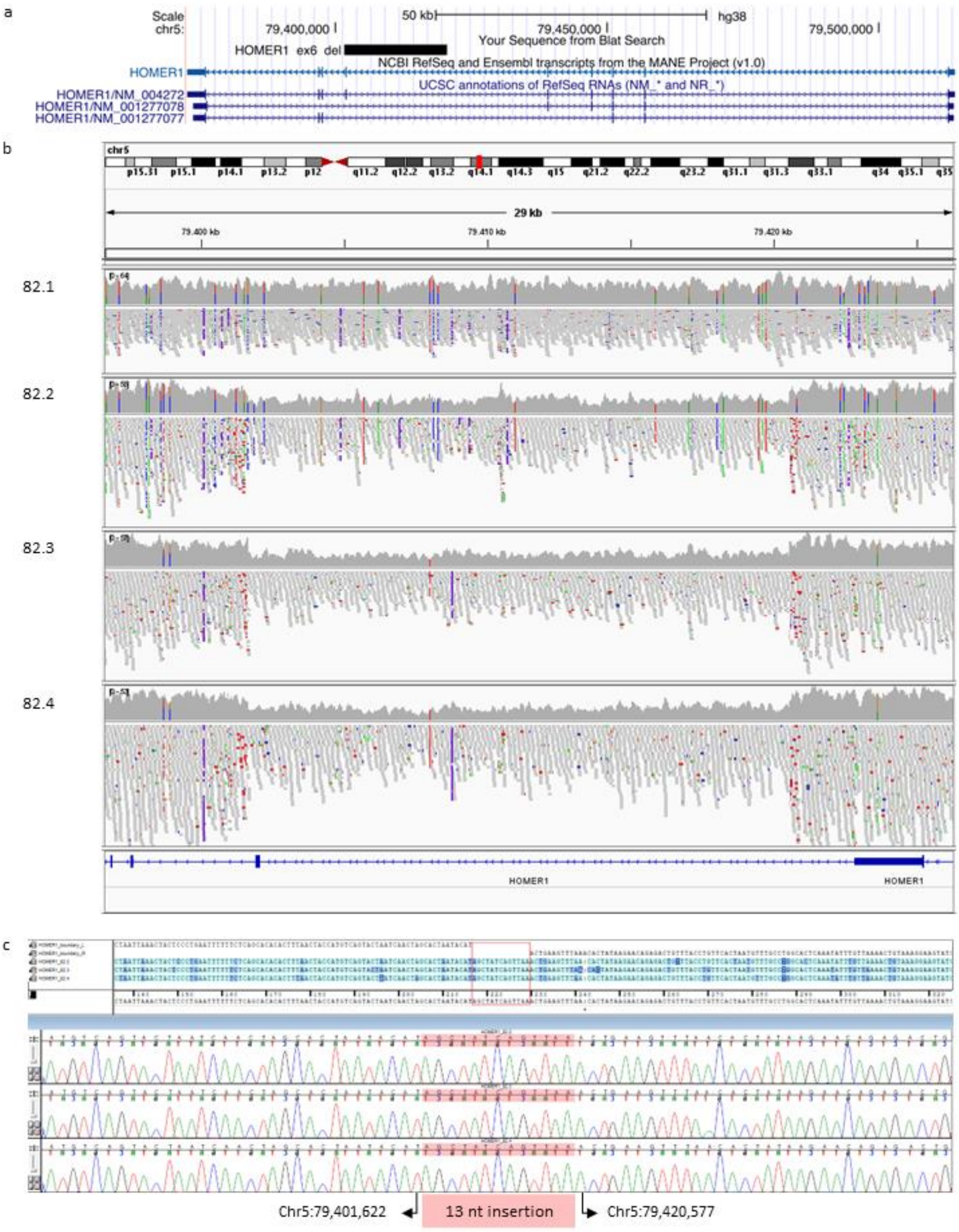

Supplementary Figure 13. *HOMER1* deletion in Family 82. a) UCSC schematic (hg38) showing the genomic structure of *HOMER1*. The 19 kb deletion identified in family 82 is indicated by a black bar and encompasses exon 6 of the isoform NM_004272 (MANE transcript). b) IGV visualisation of the deleted region. Coverage tracks and read alignments are shown for the father (82.1), mother (82.2), ASD child (82.3) and unaffected sister (82.4). The deleted region shows a decreased read depth in 82.2, 82.3 and 82.4, with clusters of red reads at both sides indicating the positions of the breakpoints. c) Sanger sequencing confirming the location of the breakpoints. The chromatograms indicate that an insertion of 13 nucleotides (highlighted in red) has occurred between the breakpoints.

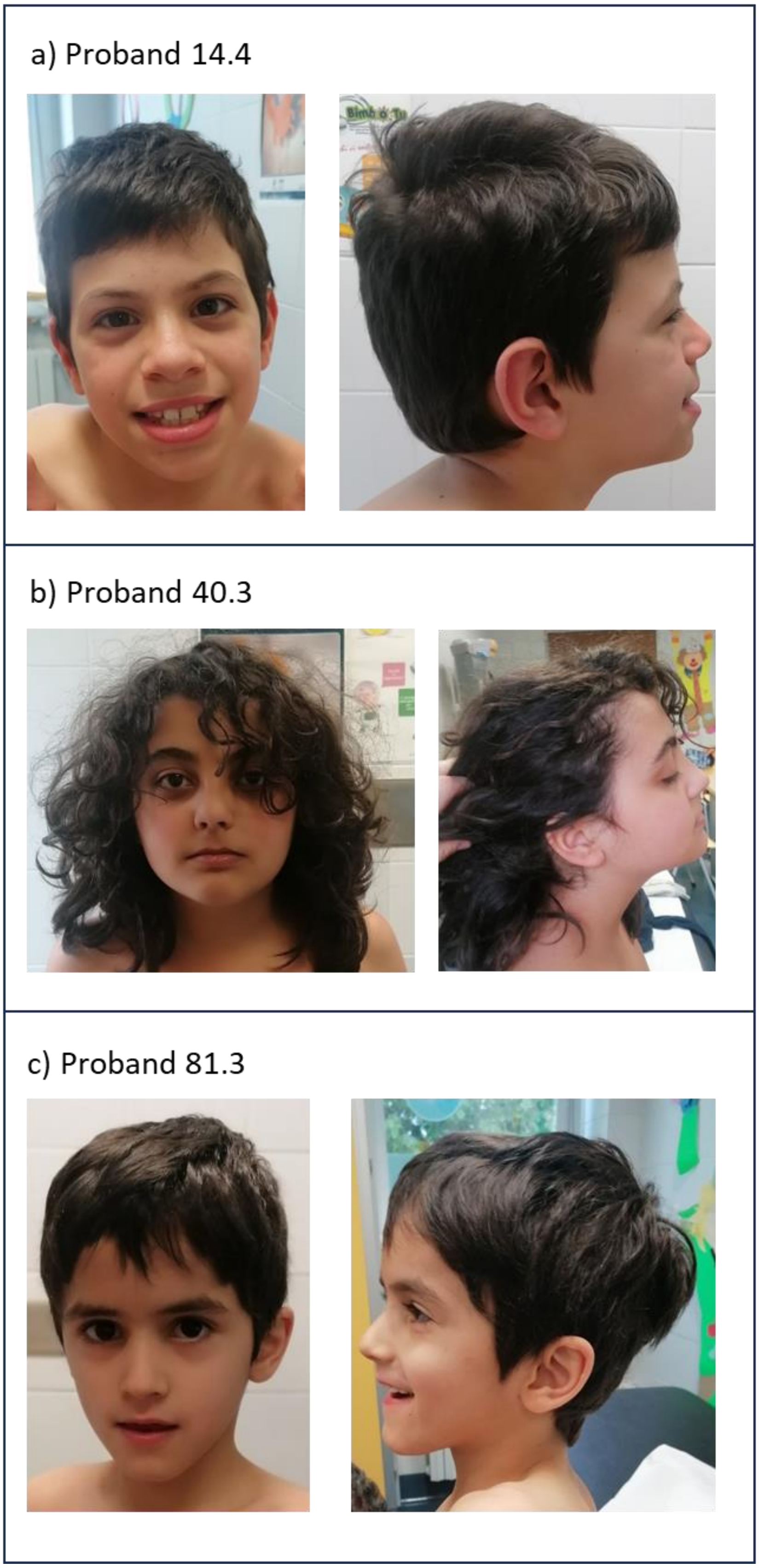

Supplementary Figure 14. Facial photographs of probands with *BRSK2*, *SCN3A* and *NFIX de novo* variants. For detailed descriptions please see Supplementary Data. Written consent was obtained for publication of the photographs.

Supplementary Table 1: Summary of phenotype information for individuals with ASD

	All individuals	Males	Females	Multiplex families (n= 25)	Simplex families (n= 89)
**Number (n)**	144	110	34	51	89
**Investigation method**					
WGS	92	71	21	7	83
WES	20	12	8	17	3
WGS and WES	32	27	5	27	3
SNP-array	144	110	34	51	89
**Clinical Diagnosis (ICD-10) [n (%)]**					
F84.0	123 (85.4%)	93 (84.5%)	30 (88.2%)	36 (70.6%)	84 (94.4%)
F84.5	2 (1.4%)	2 (1.8%)	0 (0%)	2 (3.9%)	0 (0%)
F84.9	19 (13.2%)	15 (13.6%)	4 (11.8%)	13 (25.5%)	5 (5.6%)
**Age of Diagnosis (months) [Mean; SD; range]**	40.1; 22.4; 17-181	41.2; 20.5; 17-148	36.5; 27.7; 18-181	40.0; 16.9; 17-91	39.8; 25.3; 18-181
**Symptoms Onset Pattern [n (%)]**					
Early Onset	113 (78.5%)	85 (77.3%)	28 (82.4%)	41 (80.4%)	68 (76.4%)
Regressive	10 (6.9%)	10 (9.1%)	0 (0%)	3 (5.9%)	7 (7.9%)
Plateau	2 (1.4%)	1 (0.9%)	1 (2.9%)	0 (0%)	2 (2.2%)
Mixed	19 (13.2%)	14 (12.7%)	5 (14.7%)	7 (13.7%)	12 (13.5%)
**Onset of first symptoms (months) [Mean; SD; range]**	15.6; 4.9; 6-36	15.8; 5.1; 6-36	15.3; 4.1; 7-24	16.2; 5.5; 6-36	15.4; 4.6; 6-36
**ADOS-2 diagnosis [n (%)]**					
Autism	132 (91.7%)	101 (91.8%)	31 (91.2%)	40 (78.4%)	88 (98.9%)
ASD	12 (8.3%)	9 (8.2%)	3 (8.8%)	11 (21.6%)	1 (1.1%)
**ADOS-2 comparison score [Mean; SD; range]**	7.8; 1.7; 4-10	7.7; 1.7; 4-10	7.9; 1.7; 5-10	7.2; 1.9; 4-10	8.1; 1.5; 4-10
**CARS-2 diagnosis [n (%)]**					
Severe	103 (71.5%)	79 (71.8%)	24 (70.6%)	30 (58.8%)	71 (79.8%)
Mild-moderate	41 (28.5%)	31 (28.2%)	10 (29.4%)	21 (41.2%)	18 (20.2%)
**CARS2-ST [Mean; SD; range]**	39.0; 4.3; 28.5-48	38.9; 4.3; 28.5-48	39.6; 4.1; 31-48	37.4; 4.7; 28.5-47	40.0; 3.7; 30.5-48
**Language level [n (%)]**					
0 (no problems)	2 (1.4%)	2 (1.8%)	0 (0%)	2 (3.9%)	0 (0%)
1 (atypical)	32 (22.2%)	28 (25.5%)	4 (11.8%)	15 (29.4%)	17 (19.1%)
2 (single words)	64 (44.4%)	50 (45.5%)	14 (41.2%)	19 (37.3%)	43 (48.3%)
3 (absent)	46 (31.9%)	30 (27.3%)	16 (47.1%)	15 (29.4%)	29 (32.6%)
**IQ level (non verbal) [n (%)]**					
normal	42 (29.2%)	35 (31.8%)	7 (20.6%)	13 (25.5%)	27 (30.3%)
borderline	21 (14.6%)	16 (14.5%)	5 (14.7%)	6 (11.8%)	15 (16.9%)
mild ID	36 (25%)	27 (24.5%)	9 (26.5%)	12 (23.5%)	24 (27.0%)
moderate ID	29 (20.1%)	22 (20%)	7 (20.6%)	12 (23.5%)	16 (18.0%)
severe/profound ID	16 (11.1%)	10 (9.1%)	6 (17.6%)	8 (15.7%)	7 (7.9%)
**Epilepsy [n (%)]**	12 (8.3%)	7 (6.4%)	5 (14.7%)	3 (5.9%)	7 (7.9%)
**EEG [n (%)]**					
Not assessed	18 (12.5%)	13 (11.8%)	5 (14.7%)	10 (19.6%)	8 (9.0%)
Normal	96 (66.7%)	75 (68.2%)	21 (61.8%)	30 (58.8%)	63 (70.8%)
Anomalies	30 (20.8%)	22 (20.0%)	8 (23.5%)	11 (21.6%)	18 (20.2%)
**MRI [n (%)]**					
Not assessed	19 (13.2%)	13 (11.8%)	6 (17.6%)	13 (25.5%)	6 (6.7%)
Normal	89 (61.8%)	70 (63.6%)	19 (55.9%)	29 (56.9%)	56 (62.9%)
Anomalies	36 (25.0%)	27 (24.5%)	9 (26.5%)	9 (17.6%)	27 (30.3%)

Supplementary Table 2: Analysis of subclinical features using BAPQ and SCDC scores

a) BAPQ total score distribution.

	Count	Mean	Standard Deviation	Range	two-sided t-test *p* -value
Mother (all)	99	2.44	0.47	1.33-3.69	
Multiplex families	23	2.46	0.50	1.33-3.61	0.755
Simplex families	74	2.43	0.47	1.44-3.69	
ASD male only families	70	2.45	0.46	1.44-3.69	0.644
ASD female containing families	29	2.40	0.50	1.33-3.69	
Father (all)	100	2.50	0.58	1.31-4.31	
Multiplex families	24	2.59	0.65	1.53-4.11	0.422
Simplex families	74	2.48	0.57	1.31-4.31	
ASD male only families	70	2.49	0.61	1.31-4.31	0.792
ASD female containing families	30	2.53	0.53	1.61-3.44	

b) SCDC score distribution.

	Count	Mean	Standard Deviation	Range	two-sided t-test *p* -value
Father (all)	100	3.2	3.40	0-15	0.522
Mother (all)	101	2.9	2.98	0-16	
Parents from multiplex families	49	3.6	3.67	0-15	0.142
Parents from simplex families	148	2.9	3.02	0-16	
ASD males	96	14.2	4.45	4-24	0.522
ASD females	32	14.8	4.77	4-23	
ASD individuals from multiplex families	51	14.3	4.41	4-24	0.835
ASD individuals from simplex families	73	14.1	4.53	4-23	
Unaffected brothers	27	4.2	3.86	0-14	0.734
Unaffected sisters	25	3.8	3.45	0-13	
Unaffected siblings from multiplex families	6	5.0	5.02	0-13	0.533
Unaffected siblings from simplex families	44	4.0	3.47	0-14	

Supplementary Table 3: Distribution of rare *de novo* variants

Group	This study		Group	Satterstrom et al, 2020
	144 cases	55 unaffected siblings		6430 cases (Family-data)
PTV (LOEUF<0.6)	8 (5.1%)	0	PTV (pLI≥0.995)	366 (5.1%)
			PTV_Middle(pLI=0.5-0.995)	164 (2.3%)
PTV (LOEUF≥0.6)	11 (6.9%)	4 (7.0%)	PTV_Lowest(pLI=0-0.5)	442 (6.2%)
DmisB (MPC≥2)	11 (6.9%)	4 (7.0%)	DmisB (MPC≥2)	354 (5.0%)
DmisA (1≤MPC<2)	18 (11.3%)	9 (15.8%)	DmisA (1≤MPC<2)	894 (12.5%)
Missense_Lowest (MPC<1)	69 (43.4%)	19 (33.3%)	Missense_Lowest (MPC<1)	3155 (44.2%)
Synonymous	42 (26.4%)	21 (36.8%)	Synonymous	1756 (24.6%)
Total	159	57	Total	7131

Supplementary Table 4: List of *de novo* pdSNVs identified in affected individuals (extended version of Table 1)

Genomic change (hg38)	Gene	ASD/NDD gene list [1]	Amino acid change [2]	GnomAD "non-neuro" max AF (v.2.1.1/v.3.1.2)	pdSNVs class (LOEUF/MPC score)	ACMG classification [3]	AlphaMissense prediction	pext score brain (mean proportion) [4]	OMIM disease [5]	Animal models with neurological phenotypes (#= from OMIM; §= from IMPC)	Individual (Family structure) [6]	Symptoms Onset Pattern (Ozonoff); ADOS-2 (Diagnosis, comparison score); CARS2-ST score; Language level; ID level (test, score); Epilepsy [7]
1:111695356-C-T	*RAP1A*	-	NM_002884.4:c.73C>T:p.Q25X	novel	PTV_{LOEUF} (0.433)	P<br>(PVS1,PS2,PM2,PP3)	.	0.96 (0.96)	-	-	69.3<br>(69: f/m/aM)	Early Onset; ADOS-2 (Autism, 9); CARS2-ST: 41.5; Absent speech; Moderate ID (NA); Focal epilepsy
1:178457852-C-T	*RASAL2*	-	NM_170692.4:c.2560C>T:p.Q854X	novel	PTV_{LOEUF} (0.552)	LP<br>(PS2,PM2,PP3)	.	0.92 (0.84)	-	knock-out mouse (Dickinson et al. 2016 §)	19.3<br>(19: f/m/aM)	Early Onset; ADOS-2 (Autism, 6); CARS2-ST: 42.5; Absent speech; Mild ID (NA); No epilepsy
2:165091265-G-A	*SCN3A*	NDD	NM_006922.4:c.4888C>T:p.R1630X	novel	PTV_{LOEUF} (0.348)	LP<br>(PS2,PM2,PP3,BS4)	.	0.95 (0.98)	Developmental and epileptic encephalopathy 62 (617938, AD)<br>Epilepsy, familial focal, with variable foci 4 (617935, AD)	knock-down mouse (Lamar et al. 2017 #)	40.3<br>(40: f/m/aM/aM)	Mixed; ADOS-2 (Autism, 9); CARS2-ST: 37.5; Single words; Normal IQ (Leiter-R, Brief IQ 117, Fluid Reasoning 108); No epilepsy
2:165374892-C-T	*SCN2A*	ASD	NM_001040142.2:c.4180C>T:p.Q1394X	novel	PTV_{LOEUF} (0.161)	P<br>(PVS1,PS2,PM2,PP3,BS4)	.	0.99 (0.99)	Developmental and epileptic encephalopathy 11 (613721, AD)<br>Episodic ataxia, type 9 (618924, AD)<br>Seizures, benign familial infantile, 3 (607745, AD)	knock-out mouse (Planells-Cases et al. 2000 #); overexpressing mouse (Li et al. 2021 #)	113.4<br>(113: f/m/aF/raM/uF)	Early Onset; ADOS-2 (Autism, 10); CARS2-ST: 43; Absent speech; Severe ID (NA); No epilepsy
8:102265260delG	*UBR5*	ASD	NM_015902.6:c.7441del:p.H2481Mfs*7	novel	PTV_{LOEUF} (0.073)	LP<br>(PVS1,PS2,PM2,BS4)	.	0.46 (0.5)	-	-	110.3<br>(110: f/m/aM/aM/uMs)	Early Onset; ADOS-2 (Autism, 6); CARS2-ST: 37; Atypical language; Borderline IQ (Leiter-R, Brief IQ 74); No epilepsy
11:1454559delAC	*BRSK2*	ASD	NM_001256627.2:c.1620_1621del:p.D540Efs*9	novel	PTV_{LOEUF} (0.361)	P<br>(PVS1,PS2,PM2)	.	0.69 (0.1)	-	-	32.3<br>(32: f/m/aM/uM)	Early Onset; ADOS-2 (Autism, 6); CARS2-ST: 39.5; Single words; Normal IQ (Leiter-R, Brief IQ 96, Fluid Reasoning 108); No epilepsy
16:54931226-C-T	*IRX5*	-	NM_005853.6:c.28C>T:p.Q10X	novel	PTV_{LOEUF} (0.413)	P<br>(PVS1,PS2,PM2,PP3)	.	0.98 (0.93)	Hamamy syndrome (611174, AR)	knock-out mouse (Costantini et al. 2005 #); overexpressing zebrafish (Kohl et al. 2021 #)	102.3<br>(102: f/m/aM)	Early Onset; ADOS-2 (Autism, 10); CARS2-ST: 39; Atypical language; Borderline IQ (NA); No epilepsy
22:50720258-G-T	*SHANK3*	ASD	NM_001372044.2:c.2611G>T:p.E871X	novel	PTV_{LOEUF} (0.428)	P<br>(PVS1,PM2,PM6,PP3)	.	1 (1)	Schizophrenia 15(613950, AD)<br>Phelan-McDermid syndrome (606232, AD)	knock-out mouse (Peça et al. 2011, Wang et al. 2011, Bidinosti et al. 2016 #), knock-out mouse with conditional knock-in (adulthood) (Mei et al. 2016 #), overexpressing mouse (Han et al. 2013 #)	123.3<br>(123:f/aF) [8]	Early Onset; ADOS-2 (Autism, 9); CARS2-ST: 43.5; Absent speech; Severe ID (NA); No epilepsy
22:50730801-C-A	*SHANK3*	ASD	NM_001372044.2:c.4871C>A:p.S1624X	novel	PTV_{LOEUF} (0.428)	LP<br>(PS2,PM2,PP3)	.	1 (1)	Schizophrenia 15(613950, AD)<br>Phelan-McDermid syndrome (606232, AD)	knock-out mouse (Peça et al. 2011, Wang et al. 2011, Bidinosti et al. 2016 #), knock-out mouse with conditional knock-in (adulthood) (Mei et al. 2016 #), overexpressing mouse (Han et al. 2013 #)	29.3<br>(29: f/m/aF/uM)	Early Onset; ADOS-2 (Autism, 8); CARS2-ST: 43; Absent speech; Profound ID (GMDS 0-2, CA 11 years, total AE 12,75 months); Focal epilepsy
1:27107758-T-C	*SLC9A1*	-	NM_003047.5:c.1172A>G:p.E391G	novel	DmisB (MPC=2.3)	LP<br>(PS2,PM1,PM2,PP2)	Likely pathogenic	0.39 (0.53)	Lichtenstein-Knorr syndrome (107310, AR)	spontaneous knock-out mouse (Cox et al. 1997 #)	98.3<br>(98: f/m/aM)	Early Onset; ADOS-2 (Autism, 10); CARS2-ST: 42; Single words; Borderline IQ (Leiter-R, Brief IQ 76, Fluid Reasoning 84); No epilepsy
10:27114547-C-T	*YME1L1*	-	NM_014263.4:c.1981G>A:p.E661K	novel	DmisB (MPC=2.0)	LP<br>(PS2,PM1,PM2,PP3,BS4)	Likely pathogenic	0.98 (0.98)	-	-	117.3<br>(117: f/m/aM/aF)	Early Onset; ADOS-2 (Autism, 9); CARS2-ST: 35.5; Atypical language; Mild ID (WPPSI-III, Verbal IQ 80, Performance IQ 65, Total IQ 66); Focal epilepsy
11:1442548-G-A	*BRSK2*	ASD	NM_001256627.2:c.472G>A:p.A158T	novel [9]	DmisB (MPC=2.0)	LP<br>(PS2,PM1,PM2,BS4)	Likely pathogenic	0.37 (0.18)	-		14.4<br>(14: f/m/aM/aM)	Early Onset; ADOS-2 (Autism, 8); CARS2-ST: 39; Absent speech; Moderate ID (NA); No epilepsy
11:47419145-C-T	*PSMC3*	NDD	NM_002804.5:c.1180G>A:p.A394T	novel	DmisB (MPC=2.1)	LP<br>(PS2,PM1,PM2,PP3)	Likely pathogenic	0.85 (0.85)	Deafness, cataract, impaired intellectual development, and polyneuropathy(619354, AR)	Zebrafish knock-out (Kroll-Hermi et al. 2020 #)	106.3<br>(106: f/m/aM)	Early Onset; ADOS-2 (Autism, 9); CARS2-ST: 42.5; Absent speech; Severe ID (NA); No epilepsy
14:102016376-T-C	*DYNC1H1*	ASD	NM_001376.5:c.7501T>C:p.S2501P	novel	DmisB (MPC=2.5)	LP<br>(PS2,PM2)	Likely pathogenic	0.14 (0.16) [10]	Charcot-Marie-Tooth disease, axonal, type 2O (614228, AD)<br>Cortical dysplasia, complex, with other brain malformations 13 (614563, AD)<br>Spinal muscular atrophy, lower extremity-predominant 1 (158600, AD)	chemically-induced missense variants mice (Hafezparast et al. 2003, Braunstein et al. 2010, Eschbach et al. 2013, Ori-McKenney and Vallee 2011 #)	122.3<br>(122: f/m/aM)	Early Onset; ADOS-2 (Autism, 9); CARS2-ST: 36; Single words; Normal IQ (NA); No epilepsy
15:63249686-C-T	*RAB8B*	-	NM_016530.3:c.227C>T:p.A76V	./0.000016	DmisB (MPC=2.1)	LP<br>(PS2,PM1,PM2,PP3,BS4)	Likely pathogenic	1 (0.99)	-	-	85.3<br>(85: f/m/aM/aM)	Mixed; ADOS-2 (Autism, 7); CARS2-ST: 45; Absent speech; Moderate ID (NA); No epilepsy
17:76926616-G-A	*MGAT5B*	-	NM_001199172.2:c.1177G>A:p.D393N	./0.000016	DmisB (MPC=2.0)	LP<br>(PS2,PM1,PM2,PP3,BS4)	Likely pathogenic	0.38 (0.12) [10]	-	-	15.3<br>(15: f/m/aM/aM)	Early Onset; ADOS-2 (Autism, 9); CARS2-ST: 42.5; Single words; Normal IQ (Leiter-R, Brief IQ 89, Fluid Reasoning 84); No epilepsy
19:8399920-G-A	*RAB11B*	NDD	NM_004218.4:c.98G>A:p.R33H	novel	DmisB (MPC=2.6)	LP<br>(PS2,PM1,PM2,PP2)	Likely pathogenic	1 (1)	Neurodevelopmental disorder with ataxic gait, absent speech, and decreased cortical white matter (617807, AD)	-	52.3<br>(52: f/m/aM)	Early Onset; ADOS-2 (Autism, 6); CARS2-ST: 39; Single words; Mild ID (Leiter-R, Brief IQ 56, Fluid Reasoning 63); Focal epilepsy
19:13025217-T-C	*NFIX*	NDD	NM_001365902.3:c.224T>C:p.L75P	novel	DmisB (MPC=3.3)	LP<br>(PS2,PM1,PM2,PP3,BP1)	Likely pathogenic	0.98 (0.99)	Malan syndrome (614753, AD)<br>Marshall-Smith syndrome (602535, AD)	knock-out and knock-down mice (Driller et al. 2007, Campbell et al. 2008, Harris et al. 2013, Zhou et al. 2015 #)	81.3<br>(81: f/m/aM/uM)	Early Onset; ADOS-2 (Autism, 10); CARS2-ST: 39.5; Absent speech; Severe ID (GMDS 0-2, CA 34 months, total AE 10 months); No epilepsy
21:43970678-G-A	*AGPAT3*	-	NM_020132.5:c.536G>A:p.R179H	novel	DmisB (MPC=2.2)	LP<br>(PS2,PM1,PM2,PP3)	Likely pathogenic	0.94 (0.86)	-	-	17.3<br>(17: f/m/aM/uM)	Regressive; ADOS-2 (Autism, 9); CARS2-ST: 37; Single words; Mild ID (Leiter-R, Full IQ 57, Fluid Reasoning 48); No epilepsy
22:39488750-C-T	*MGAT3*	-	NM_002409.5:c.1403C>T:p.T468M	0/0.000067	DmisB (MPC=2.2)	LP<br>(PS2,PM2)	Likely benign	0.99 (0.99)	-	-	5.3<br>(5: f/m/aM)	Early Onset; ADOS-2 (Autism, 5); CARS2-ST: 39.5; Single words; Borderline IQ (Leiter-R, Brief IQ 79, Fluid Reasoning 62); No epilepsy
1:26950738-G-A	*KDF1*	-	NM_152365.3:c.1058C>T:p.S353F	novel	DmisA (MPC=1.0)	LP<br>(PS2,PM2,PP2)	Likely pathogenic	1 (1)	-	-	81.3<br>(81: f/m/aM/uM)	Early Onset; ADOS-2 (Autism, 10); CARS2-ST: 39.5; Absent speech; Severe ID (GMDS 0-2, CA 34 months, total AE 10 months); No epilepsy
1:52033586-C-T	*KTI12*	-	NM_138417.3:c.176G>A:p.R59H	novel	DmisA (MPC=1.7)	LP<br>(PS2,PM2,PP3,BS4)	Likely pathogenic	1 (1)	-	-	16.4<br>(16: f/m/aM/aM)	Early Onset; ADOS-2 (Autism, 9); CARS2-ST: 39; Absent speech; Moderate ID (PEP-3, CA 2.7 years, cognitive-verbal AE <12 months); No epilepsy
2:196317282-A-G	*HECW2*	NDD	NM_001348768.2:c.2426T>C:p.L809P	novel	DmisA (MPC=1.3)	P<br>(PS2,PM1,PM2,PP2,PP3)	Likely pathogenic	1 (0.99)	Neurodevelopmental disorder with hypotonia, seizures, and absent language (617268, AD)	-	68.3<br>(68: f/m/aF/uF/uM)	Mixed; ADOS-2 (Autism, 10); CARS2-ST: 38.5; Absent speech; Borderline IQ (Leiter-R, Brief IQ 74, Fluid Reasoning 73); No epilepsy
2:241742460-G-A	*D2HGDH*	-	NM_152783.5:c.376G>A:p.V126M	0.000056/0.0001<br>43	DmisA (MPC=1.2)	LP<br>(PS2,PM1,PM2,PP3)	Ambiguous	0.42 (0.31)	D-2-hydroxyglutaric aciduria (600721, AR)	-	87.3<br>(87: f/m/aM/uM)	Early Onset; ADOS-2 (Autism, 6); CARS2-ST: 40.5; Single words; Mild ID (Bayley-III, CA 38 months, equivalent age 19 months); No epilepsy
3:186786601-A-G	*EIF4A2*	NDD	NM_001967.4:c.727A>G:p.T243A	novel	DmisA (MPC=1.7)	LP<br>(PS2,PM1,PM2,BS4)	Likely pathogenic	0.77 (0.64)	Neurodevelopmental disorder with hypotonia and speech delay, with or without seizures(620455; AD/AR)	missense mutation induced in *Drodophila* (Paul et al. 2023 #)	110.4<br>(110: f/m/aM/aM/uMs)	Early Onset; ADOS-2 (ASD, 4); CARS2-ST: 33; Atypical language; Normal IQ (Leiter-R, Brief IQ 107, Fluid Reasoning 102); No epilepsy
6:122997919-C-G	*CLVS2*	-	NM_001010852.4:c.142C>G:p.R48G	novel	DmisA (MPC=1.3)	LP<br>(PS2,PM1,PM2)	Likely pathogenic	1 (0.98)	-	-	44.3<br>(44: f/m/aM/uF)	Early Onset; ADOS-2 (Autism, 7); CARS2-ST: 45; Single words; Mild ID (Leiter-R, Brief IQ 54, Fluid Reasoning 52); No epilepsy
9:132904429-C-A	*TSC1*	ASD	NM_000368.5:c.2023G>T:p.D675Y	novel	DmisA (MPC=1.6)	LP<br>(PS2,PM1,PM2,PP3)	Likely pathogenic	0.9 (0.79)	Focal cortical dysplasia, type II, somatic (607341)<br>Tuberous sclerosis-1 (191100, AD)<br>Lymphangioleiomyomatosis (606690)	Knock-out and knock-down mice (Kobayashi et al. 2001, Kwiatkowski et al. 2002, Uhlmann et al. 2002, Goorden et al. 2007 #), conditional knock-out mouse (glia) (Zeng et al. 2008), conditional knock-out mouse (adulthood)(Abs et al. 2013), conditional knock-out mouse (Purkinje cells)(Tsai et al. 2012), conditional knock-out mouse (developing mouse neurons)(Lim et al. 2017, Ercan et al. 2017)	100.3<br>(100: f/m/aF)	Early Onset; ADOS-2 (Autism, 9); CARS2-ST: 40; Single words; Mild ID (NA); No epilepsy
10:91482997-G-A	*HECTD2*	-	NM_182765.6:c.742G>A:p.V248I	novel	DmisA (MPC=1.0)	LP<br>(PS2,PM1,PM2)	Likely benign	0.31 (0.53)	-	knock-out mouse (Dickinson et al. 2016 §)	30.3<br>(30: f/m/aM/uM)	Early Onset; ADOS-2 (Autism, 9); CARS2-ST: 41.5; Absent speech; Moderate ID (PEP-3, CA 3 years, cognitive-verbal AE 17 months); No epilepsy
10:96551263-A-G	*TM9SF3*	-	NM_020123.4:c.941T>C:p.I314T	novel	DmisA (MPC=1.2)	LP<br>(PS2,PM2,BS4)	Likely benign	1 (0.96)	-	-	12.5<br>(12: f/m/aM/uM/aM)	Early Onset; ADOS-2 (Autism, 6); CARS2-ST: 33.5; Single words; Mild ID (GMDS 0-2, CA 17 months, total AE 11 months); No epilepsy
13:41192409-G-A	*KBTBD7*	NDD	NM_032138.7:c.1849C>T:p.R617C	0.000206/0.00038	DmisA (MPC=1.9)	VUS<br>(PS2,PP3,BS4)	Ambiguous	1 (1)	-	knock-out mouse (Dickinson et al. 2016 §)	40.4<br>(40: f/m/aM/aM)	Mixed; ADOS-2 (Autism, 8); CARS2-ST: 34.5; Atypical language; Normal IQ (Leiter-R, Brief IQ 111, Fluid Reasoning 88); No epilepsy
14:73103376-G-A	*RBM25*	-	NM_021239.3:c.1052G>A:p.R351H	0.000034/.	DmisA (MPC=1.3)	LP<br>(PS2,PM1,PM2)	Ambiguous	0.74 (0.66)	-	knock-out mouse (Dickinson et al. 2016 §)	60.3<br>(60: f/m/aF)	Early Onset; ADOS-2 (Autism, 10); CARS2-ST: 48; Single words; Moderate ID (PEP-3, CA 5.6 years, cognitive-verbal AE 36months); No epilepsy
15:90957365-C-A	*RCCD1*	-	NM_001017919.2:c.419C>A:p.A140D	novel	DmisA (MPC=1.6)	VUS<br>(PS2,PM1,PM2,BS4,BP4)	Likely benign	0.7 (0.57)	-	knock-out mouse (Dickinson et al. 2016 §)	117.4<br>(117: f/m/aM/aF)	Early Onset; ADOS-2 (Autism, 8); CARS2-ST: 34.5; Single words; Normal IQ (Leiter-3, IQ 97); No epilepsy
17:2670194-G-A	*PAFAH1B1*	NDD	NM_000430.4:c.431G>A:p.R144Q	novel	DmisA (MPC=1.6)	LP<br>(PS2,PM1,PM2,BS4)	Ambiguous	0.91 (0.72)	Lissencephaly 1 (607432, AD)<br>Subcortical laminar heterotopia (607432, AD)	knock-out and knock-down mice (Hirotsune et al. 1998, Assadi et al. 2003, Pawlisz et al. 2008, Yamada et al. 2009, Greenwood et al. 2009 #), start-loss mutation induced in mouse (Cahana et al. 2001 #), knock-out induced in *Drosophila* (Liu et al. 2000 #), Knock out and knock down in *C. elegans* (Williams et al. 2004 #)	15.4<br>(15: f/m/aM/aM)	Early Onset; ADOS-2 (Autism, 6); CARS2-ST: 33; Normal language; Borderline IQ (WISC-IV, Full IQ 78); No epilepsy
17:7323905-C-T	*NEURL4*	-	NM_032442.3:c.2170G>A:p.G724S	0.000011/0.00001	DmisA (MPC=1.5)	LP<br>(PS2,PM1,PM2,PP3,BS4)	Likely pathogenic	0.36 (0.35)	-	knock-out mouse (Dickinson et al. 2016 §)	115.3<br>(115: f/m/aF/aM/aF)	Early Onset; ADOS-2 (Autism, 6); CARS2-ST: 36; Single words; Moderate ID (GMDS, CA 27 months, total AE 5.5 months); No epilepsy

17:75496430-C-T	*TMEM94*	-	NM_014738.6:c.3202C>T:p.R1068C	0.000062/0.0001	DmisA (MPC=1.2)	LP (PS2,PM1,BP1)	Likely benign	0.32 (0.3)	Intellectual developmental disorder with cardiac defects and dysmorphic facies (618316, AR)	knock-out mouse (Stephen et al. 2018 #)	101.3 (101: f/m/aM)	Early Onset; ADOS-2 (ASD, 4); CARS2-ST: 36.5; Absent speech; Moderate ID (GMDS, CA 43 months, total AE 21.5 months); No epilepsy
17:10528976-C-T	*MYH2*	-	NM_017534.6:c.3458G>A:p.S1153N	novel	DmisA (MPC=1.0)	LP (PS2,PM1,PM2,PP3,BS4)	Likely pathogenic	1 (1)	Congenital myopathy 6 with ophthalmoplegia (605637, AD/AR)	-	77.3 (77: f/m/aM/aF/uM/uFs)	Early Onset; ADOS-2 (Autism, 9); CARS2-ST: 41.5; Single words; Mild ID (Leiter-R, NA); No epilepsy
20:42677943-C-T	*PTPRT*	-	NM_007050.6:c.1076G>A:p.R359Q	0.000033/0.0000	DmisA (MPC=1.2)	LP (PS2,PM1)	Likely benign	1 (1)	-	-	72.3 (72: f/m/aF/uF)	Early Onset; ADOS-2 (Autism, 6); CARS2-ST: 34; Single words; Mild ID (Leiter-R, NA); No epilepsy
22:42693390-C-T	*A4GALT*	-	NM_017436.7:c.562G>A:p.G188S	0.000062/0.0005	DmisA (MPC=1.1)	LP (PS2,PM1,PM2,PP3)	Likely pathogenic	0.95 (0.94)	NOR polyagglutination syndrome / Blood group, P1Pk system, P(2) phenotype / Blood group, P1Pk system, p phenotype (111400)	-	90.3 (90: f/m/aF/dM)	Early Onset; ADOS-2 (Autism, 9); CARS2-ST: 41; Absent speech; Moderate ID (PEP-3, CA 4 years, cognitive-verbal AE 24 months); No epilepsy

De novo pdSNVs include PTVs in genes with LOEUF score <0.6 (PTV_{LOEUF}), missense variants with MPC score ≥2 (DmisB) and missense variants with 1≤MPC<2 (DmisA). [1] High-confidence ASD/NDD gene list classification according to Table S8; [2] Amino acid changes are reported according to the MANE isoform; [3] Classification of genetic variants according to the ACMG guidelines, together with detailed evidence codes, was performed using InterVar. Abbreviations: P, pathogenic; LP, likely pathogenic; VUS, variant of uncertain significance; [4] The reported brain pext score is the mean value of the 13 GTEx brain tissues; [5] Only established OMIM diseases are reported; [6] Probands are indicated with an identifier code formed by the family number and the individual number. In brackets, the family structure is reported with children listed sequentially after the father and the mother, according to the recruitment order. The affected individual heterozygote for the variant is underlined. Abbreviations: f, father; m, mother; r, relative (uncle); a, affected; u, unaffected; M, male; F, female; [7] Epilepsy is present only where it is explicitly indicated. Abbreviations: EO, Early Onset; NA, Not Assessed; V-IQ, Verbal IQ; P-IQ, Performance IQ; B-IQ, Brief IQ; FR, Fluid Reasoning; CA, chronological age; AE, age equivalent; GMDS, Griffiths Mental Development Scales; m, months; y, years; [8] Since DNA of the biological mother was unavailable, *de novo* status was only assumed; [9] This variant has been reported in gnomAD v3.1.2 in a single individual of African/African American origin belonging to the gnomAD “neuro” dataset, therefore the variant is not present in gnomAD v3.1.2 "non-neuro" dataset. [10] For this gene, RSEM assigns higher expression to non-coding transcripts than protein coding transcripts. This likely represents an artifact in the isoform expression quantification and results in a low pext value for all bases of this gene.

Supplementary Table 5: Gene ontology from 17 genes carrying the 19 most severe *de novo* pdSNVs identified in 144 cases

Term ID	GO biological process description	# genes (Reference list)	# genes (Our list)	Expected	Fold Enrichment	P value (Fisher's Exact)	FDR	Genes (Our list)
GO:0051049	regulation of transport	1771	10	1.46	6.84	0.00000025	0.00388	RAB11B, UBR5, BRSK2, SCN2A, SLC9A1, SCN3A, SHANK3, RAB8B, DYNC1H1, RAP1A
GO:0032879	regulation of localization	2126	10	1.76	5.7	0.00000137	0.0107	RAB11B, UBR5, BRSK2, SCN2A, SLC9A1, SCN3A, SHANK3, RAB8B, DYNC1H1, RAP1A

GO functional enrichment analysis performed using the Annotation Data Set "GO biological process". Only results for FDR $p < 0.05$ are displayed. The 19 most severe *de novo* pdSNVs include 9 PTV_{LOEUF} and 10 DmisB variants.

Supplementary Table 6 (List of de novo and inherited pdSNVs in cases) is available at the end of this file.

Supplementary Table 7: SynGO Gene Set Enrichment Analysis (GSEA) of genes carrying *de novo* and inherited pdSNVs in 144 cases
a) pdSNVs

GO term ID	GO domain	GO term name	GSEA 'gene cluster' FDR corrected p-value	GSEA count foreground/input	GSEA count background	Genes
GO:0045202	CC	synapse	1.12E-10	218	1082	FLOT1;CYFIP2;SLC18A1;AGRN;PABPC1;RPL5;RPL6;RPL3;RPS25;SLC6A11;NCAN;PPFIA3;PPFIA4;ARHGDIA;SLC3A2;CLU;DPYSL3;SPARC;SH3GL1;MADD;YWHAG;DVL3;CDH10;RPL13A;RPL23A;RPL30;RPS10;EIF4G3;RPS6KA4;ATP8A1;CDH8;SYT3;ITGB3;ITGB1;PTPRA;FBXO45;RAB8B;CACNB4;DPYSL2;CACNB1;CADPS2;CPEB2;APBB1;SEPTIN3;STXBP5;TNIK;HIP1;ADD2;NOS1AP;ARFGEF2;GRK2;GAD1;GAD2;NECAP1;LRFN2;GAP43;PRKCB;CTBP2;NSF;HTT;ANKS1B;CALB1;GDI1;C1QBP;UNC13A;CNTNAP1;ARHGAP44;CACNA1B;RIMS1;RIMS2;CTNNA2;CTNNB1;CTNND1;APBA1;ATP2B2;ATP2B4;CANX;GRM7;GRIA2;GRIA1;GRIA4;GRIN2B;LPAR2;KCNMA1;KCNJ8;GRM4;CACNA1D;NRXN1;NRG1;ITGA3;WDR7;PICK1;RAB3A;RAB11B;RAB4A;DOC2A;ATP6V1B1;SLC6A2;STX12;ATP6V0D1;CALCRL;KCNC4;CADM3;EPHB2;ADCY8;SCN2A;SLC6A1;SLC1A6;CACNA1C;SLC6A3;ADGRL1;GRIK5;SCN1A;CDH2;GABBR2;CHRM1;LPAR1;ERBB2;NGFR;ITPR3;ITPR1;LAMB2;NPTX1;C1QL2;ADAM10;BEGAIN;ELAVL1;NCKAP1;APC2;TRIM3;CPEB4;PSD2;EIF4B;PPP1R1B;EIF4G1;GIT1;SIPA1L1;ARHGAP39;CALD1;CNN3;SPTB;SPTAN1;MYO9A;AGAP2;MYO5A;PUM2;ARHGEF2;PLCB1;PLCB3;MTOR;DAG1;KIF5C;NEFH;MYH10;SYNPO;DLGAP3;DLGAP4;NOS1;HTR5A;CHRNA1;CHRNB1;KCND2;NLGN2;NLGN1;PRKCZ;HOMER2;DRP2;PAK3;SHANK2;SHANK3;MAGI2;CTNND2;ADGRA1;PLEKHA5;TSC1;HNRNPH1;CAPZB;RAPGEF4;ABL1;NUMB;LZTS3;DAPK1;AGAP3;IQSEC1;TANC2;SH3GL3;DLG2;MPP2;LZTS1;GRM1;ADCY1;PLPPR4;GRID1;CNIH2;LRFN4;LRRC4;LRRC4C;PTPRF;PTPRT;DRD3;SORCS3;CACNG7;KCNAB2;IQSEC3;IGSF9B;SH3GLB2;RAB11FIP3;CNTN2;TENM2;FBXO2;ADGRB1;NTRK3;DAGLA;GABRD;KCNN2;ITGA5;ITGB4;BCAN
SYNGO: synprocess	BP	process in the synapse	1.25E-10	187	874	CALB1;CACNA1B;CACNA1D;CACNB4;GRIK3;GRIK5;GRIA1;GRIA2;GRIA4;GRIN2B;KCNMA1;KCNC4;SCN2A;SCN1A;KCNJ8;NECAP1;NSF;STXBP5;NRXN1;ARHGDIA;RAPGEF4;LPAR1;CTNNB1;STX12;UNC13A;STX1B;PPFIA3;DOC2A;RIMS1;RIMS2;PRKCB;GIT1;WNT7A;FBXO45;CTBP2;RAB3A;CADPS2;ATP6V0D1;ATP6V1B1;CANX;KIAA1109;TBC1D2;SLC6A11;SLC1A6;SLC6A3;SLC6A1;SLC6A2;FLOT1;DRD3;SYNPO;MYO5A;ATP2B2;ITPR1;GRM1;CHRM1;NOS1;CHRNA1;CHRNB1;GABRD;KCND2;BEGAIN;NPTX1;CNIH2;NRXN3;CTNND1;CLPTM1;CTNND2;AGAP3;SNX27;DLG2;CACNG7;C1QL2;ERBB2;PRKCZ;DAG1;MAGI2;TNIK;ADAM10;RAP1A;PAK3;SACM1L;ITGB3;ITGB1;SH3GLB2;IQSEC1;NRG1;PICK1;NUMB;HIP1;ARRB2;NF1;ANKS1B;HTT;DAGLA;PLCB1;TENM2;TENM3;TENM4;PLG;ADCY8;ADCY1;EPHB1;CDH2;LRRC4;LRRC4C;LRFN2;PTPRA;NCAN;PACSIN2;SHANK3;DLGAP3;ARHGAP44;KCNN2;PAFAH1B1;APBA1;GRM4;GRM7;GRM2;NGFR;SORCS3;NEFH;IGF1R;APBB1;NLGN1;IQSEC3;ARHGAP39;ITGA3;SEMA3F;SHANK2;PTPRF;GRID1;MYO9A;CTNNA2;SPTB;MYH10;NCKAP1;AGRN;SPARC;CDH8;PTPRT;ACTN1;MPP2;LAMB2;IGSF9B;LRFN4;CDH10;MDGA1;PLXND1;ADD2;PPFIA4;EPHB2;NRG2;IL1RAPL2;NTRK3;NLGN2;LZTS1;LZTS3;NUMBL;CRK;GAP43;ABL1;SIPA1L1;PLXNC1;BCAN;ROCK2;CNTNAP1;PLXNA4;ARHGEF7;ADGRB1;NOS1AP;EZR;FBXO2;MTOR;CPEB2;TRIM3;GAD1;GAD2;RPL13A;RPL23A;RPL5;RPL6;RPS10;DPYSL2;KIF5A;KIF17;SFPQ;MADD
GO:0098794	CC	postsynapse	1.64E-10	143	621	ADAM10;BEGAIN;ELAVL1;NCKAP1;APC2;TRIM3;CPEB2;CPEB4;PSD2;EIF4B;PPP1R1B;EIF4G1;CALB1;GIT1;SIPA1L1;CACNB1;ADD2;NOS1AP;ARHGAP39;ARFGEF2;CALD1;CNN3;SPTB;SPTAN1;MYO9A;AGAP2;MYO5A;PUM2;NRG1;ARHGEF2;LRFN2;FBXO45;PLCB1;PLCB3;HTT;MTOR;DAG1;KIF5C;NEFH;MYH10;SYNPO;DLGAP3;DLGAP4;PICK1;NOS1;HTR5A;CHRNA1;CHRNB1;KCND2;CDH10;NLGN2;NLGN1;PRKCZ;HOMER2;DRP2;PAK3;SHANK2;SHANK3;MAGI2;ANKS1B;CTNND2;RPL5;ADGRA1;PLEKHA5;RPL30;TSC1;RPS25;HNRNPH1;RPL6;ARHGAP44;CAPZB;RAPGEF4;ABL1;NUMB;LZTS3;GRK2;DAPK1;AGAP3;CTNNA2;CTNNB1;IQSEC1;TNIK;TANC2;CTNND1;SH3GL1;SH3GL3;DLG2;MPP2;LZTS1;ATP2B2;GRM1;ADCY1;GRIA1;PLPPR4;GRIK5;GRIA2;GRIN2B;GRID1;CNIH2;LRFN4;LRRC4;LRRC4C;PTPRF;PTPRT;GRIA4;DRD3;CHRM1;SORCS3;CACNG7;KCNAB2;IQSEC3;IGSF9B;SH3GLB2;STX12;RAB11FIP3;HIP1;CNTN2;TENM2;FBXO2;KCNC4;EPHB2;CANX;CACNA1C;SLC6A1;SLC6A11;SLC1A6;SLC6A3;CDH2;GABBR2;ADGRB1;NTRK3;DAGLA;ITGB1;GABRD;KCNN2;ITGA3;ITGA5;ITGB4;LPAR1;RPL13A;RPL23A;RPS10;ITPR1
GO:0050808	BP	synapse organization	4.83E-09	84	302	APBB1;NLGN1;IQSEC3;ADAM10;ARHGAP39;ITGA3;SEMA3F;SHANK2;SHANK3;PTPRF;LRFN2;GRID1;WNT7A;RAPGEF4;PAK3;NF1;PAFAH1B1;NCAN;MYO9A;DLGAP3;ARHGAP44;CTNNA2;SPTB;MYH10;NCKAP1;AGRN;SPARC;DAG1;CDH8;PTPRT;RIMS1;RIMS2;CTBP2;ACTN1;NEFH;MAGI2;GIT1;MPP2;DLG2;CTNND2;LAMB2;IGSF9B;LRFN4;LRRC4;LRRC4C;NRXN1;NRG1;CDH10;MDGA1;PLXND1;ADD2;PPFIA3;PPFIA4;EPHB2;NRG2;CTNNB1;IL1RAPL2;NTRK3;NLGN2;NUMB;LZTS1;LZTS3;NUMBL;CRK;GAP43;ABL1;NPTX1;C1QL2;NRXN3;SIPA1L1;PLXNC1;ITGB1;BCAN;ROCK2;CNTNAP1;PLXNA4;TENM2;TENM3;TENM4;ITGB3;ARHGEF7;ADGRB1;NOS1AP;EZR
GO:0099572	CC	postsynaptic specialization	3.92E-06	74	311	DLGAP3;DLGAP4;PICK1;NOS1;HTR5A;CHRNA1;CHRNB1;KCND2;CDH10;NLGN2;NLGN1;PRKCZ;HOMER2;DRP2;PAK3;SHANK2;SHANK3;MAGI2;ANKS1B;CTNND2;RPL5;ADGRA1;PLEKHA5;RPL30;TSC1;RPS25;HNRNPH1;RPL6;ARHGAP44;CAPZB;RAPGEF4;ABL1;NUMB;LZTS3;GRK2;DAPK1;AGAP3;CTNNA2;CTNNB1;IQSEC1;ARHGEF2;TNIK;TANC2;CTNND1;SH3GL1;SH3GL3;DLG2;MPP2;LZTS1;ATP2B2;GRM1;ADCY1;GRIA1;PLPPR4;GRIK5;GRIA2;GRIN2B;GRID1;CNIH2;LRFN2;LRFN4;LRRC4;LRRC4C;PTPRF;PTPRT;GRIA4;DRD3;CHRM1;SORCS3;CACNG7;NRG1;KCNAB2;IQSEC3;IGSF9B
GO:0014069	CC	postsynaptic density	8.95E-06	62	251	PRKCZ;HOMER2;DRP2;PAK3;SHANK2;SHANK3;MAGI2;ANKS1B;CTNND2;RPL5;ADGRA1;PLEKHA5;RPL30;TSC1;RPS25;HNRNPH1;RPL6;ARHGAP44;CAPZB;RAPGEF4;ABL1;NUMB;LZTS3;GRK2;DAPK1;AGAP3;CTNNA2;CTNNB1;IQSEC1;ARHGEF2;TNIK;NOS1;TANC2;CTNND1;SH3GL1;SH3GL3;DLG2;MPP2;LZTS1;ATP2B2;GRM1;ADCY1;GRIA1;PLPPR4;GRIK5;GRIA2;GRIN2B;GRID1;CNIH2;LRFN2;LRFN4;LRRC4;LRRC4C;PTPRF;PTPRT;GRIA4;DRD3;CHRM1;SORCS3;CACNG7;NRG1;KCNAB2
GO:0048786	CC	presynaptic active zone	1.42E-05	31	89	FLOT1;C1QBP;UNC13A;CNTNAP1;ARHGAP44;PPFIA3;CACNA1B;RIMS1;CTBP2;RIMS2;CTNNA2;CTNNB1;CTNND1;APBA1;ATP2B2;ATP2B4;CANX;GRM7;GRIA2;GRIA1;GRIA4;GRIN2B;LPAR2;KCNMA1;KCNJ8;GRM4;CACNA1D;CDH10;NRXN1;NRG1;ITGA3
GO:0045211	CC	postsynaptic membrane	2.85E-05	35	114	CTNNB1;HIP1;CNTN2;MAGI2;TENM2;CTNNA2;FBXO2;ATP2B2;KCNC4;EPHB2;CANX;CACNA1C;SLC6A1;SLC6A11;SLC1A6;SLC6A3;CHRM1;CDH2;GRIA1;GRIA2;GRIK5;GRM1;GABBR2;ADGRB1;NTRK3;DAGLA;ITGB1;KCND2;GABRD;KCNN2;ITGA3;ITGA5;ITGB4;LPAR1;GRID1
SYNGO:postsynprocess	BP	process in the postsynapse	3.20E-05	56	218	SYNPO;MYO5A;ATP2B2;CALB1;ITPR1;GRM1;CHRM1;NOS1;GRIN2B;CHRNA1;CHRNB1;GRIA1;GRIA2;GABRD;GRIA4;GRIK5;KCNC4;KCND2;BEGAIN;NPTX1;CNIH2;NRXN3;CTNND1;CLPTM1;CTNND2;AGAP3;STX12;SNX27;GIT1;DLG2;CACNG7;C1QL2;ERBB2;PRKCZ;DAG1;MAGI2;TNIK;ADAM10;RAP1A;PAK3;RAPGEF4;SACM1L;ITGB3;ITGB1;SH3GLB2;IQSEC1;DRD3;NRG1;PICK1;NUMB;HIP1;LPAR1;ARRB2;NF1;ANKS1B;HTT
GO:0098793	CC	presynapse	2.37E-04	99	531	FBXO45;RAB8B;CACNB4;DPYSL2;CACNB1;CADPS2;CPEB2;APBB1;SEPTIN3;FLOT1;STXBP5;TNIK;HIP1;YWHAG;ADD2;NOS1AP;SH3GL1;ARFGEF2;GRK2;GAD1;GAD2;NECAP1;LRFN2;GAP43;PPFIA4;PRKCB;CTBP2;NSF;HTT;ANKS1B;CALB1;GDI1;C1QBP;UNC13A;CNTNAP1;ARHGAP44;PPFIA3;CACNA1B;RIMS1;RIMS2;CTNNA2;CTNNB1;CTNND1;APBA1;ATP2B2;ATP2B4;CANX;GRM7;GRIA2;GRIA1;GRIA4;GRIN2B;LPAR2;KCNMA1;KCNJ8;GRM4;CACNA1D;CDH10;NRXN1;NRG1;ITGA3;WDR7;PICK1;RAB3A;RAB11B;RAB4A;DOC2A;ATP6V1B1;SLC6A2;ATP8A1;STX12;ATP6V0D1;CALCRL;KCNC4;CADM3;EPHB2;ADCY8;SCN2A;SLC6A1;SLC6A11;SLC1A6;CACNA1C;SLC6A3;ADGRL1;GRIK5;SCN1A;CDH2;GABBR2;CHRM1;LPAR1;ERBB2;NGFR;RPL13A;RPL23A;RPL5;RPL6;RPS10;ITPR3;ITPR1
GO:0099055	CC	integral component of postsynaptic membrane	4.37E-04	28	97	ATP2B2;KCNC4;EPHB2;CANX;CACNA1C;SLC6A1;SLC6A11;SLC1A6;SLC6A3;CHRM1;CDH2;GRIA1;GRIA2;GRIK5;GRM1;GABBR2;ADGRB1;NTRK3;DAGLA;ITGB1;KCND2;GABRD;KCNN2;ITGA3;ITGA5;ITGB4;LPAR1;GRID1
GO:0099059	CC	integral component of presynaptic active zone membrane	6.84E-04	17	45	ATP2B2;ATP2B4;CANX;GRM7;GRIA2;GRIA1;GRIA4;GRIN2B;LPAR2;KCNMA1;KCNJ8;GRM4;CACNA1D;CDH10;NRXN1;NRG1;ITGA3
GO:0048787	CC	presynaptic active zone membrane	9.89E-04	19	57	APBA1;RIMS1;ATP2B2;ATP2B4;CANX;GRM7;GRIA2;GRIA1;GRIA4;GRIN2B;LPAR2;KCNMA1;KCNJ8;GRM4;CACNA1D;CDH10;NRXN1;NRG1;ITGA3
GO:0099061	CC	integral component of postsynaptic density membrane	1.76E-03	23	82	ATP2B2;GRM1;ADCY1;GRIA1;PLPPR4;GRIK5;GRIA2;GRIN2B;GRID1;CNIH2;LRFN2;LRFN4;LRRC4;LRRC4C;PTPRF;PTPRT;GRIA4;DRD3;CHRM1;SORCS3;CACNG7;NRG1;KCNAB2
GO:0098839	CC	postsynaptic density membrane	2.20E-03	26	101	DLG2;MPP2;LZTS1;ATP2B2;GRM1;ADCY1;GRIA1;PLPPR4;GRIK5;GRIA2;GRIN2B;GRID1;CNIH2;LRFN2;LRFN4;LRRC4;LRRC4C;PTPRF;PTPRT;GRIA4;DRD3;CHRM1;SORCS3;CACNG7;NRG1;KCNAB2
GO:0099072	BP	regulation of postsynaptic membrane neurotransmitter receptor levels	2.67E-03	32	121	NRXN3;CTNND1;CLPTM1;CTNND2;AGAP3;STX12;SNX27;GIT1;NPTX1;DLG2;CACNG7;C1QL2;ERBB2;PRKCZ;DAG1;MAGI2;TNIK;ADAM10;RAP1A;PAK3;RAPGEF4;SACM1L;ITGB3;ITGB1;SH3GLB2;IQSEC1;DRD3;NRG1;PICK1;NUMB;HIP1;LPAR1
GO:0007416	BP	synapse assembly	3.65E-03	26	92	ADD2;NRXN1;PPFIA3;PPFIA4;EPHB2;NRG2;CTNNB1;NRG1;IL1RAPL2;NTRK3;LRFN4;NLGN2;MDGA1;WNT7A;NUMB;LZTS1;LZTS3;NUMBL;NLGN1;CRK;GAP43;ABL1;NPTX1;C1QL2;NRXN3;SIPA1L1
GO:0042734	CC	presynaptic membrane	6.80E-03	30	136	CTNNB1;ATP2B2;STXBP5;CTNNA2;KCNC4;CADM3;EPHB2;ADCY8;CACNA1B;SCN2A;SLC6A1;SLC6A11;SLC1A6;CACNA1C;SLC6A3;SLC6A2;NRXN1;ADGRL1;GRIK5;GRIA1;GRIA2;SCN1A;GRIN2B;GRM7;CDH2;GABBR2;CHRM1;LPAR1;ERBB2;NGFR

The table shows all SynGO ontology terms enriched in our list of 2,156 genes harbouring pdSNVs in cases as compared to the “brain expressed" background set (18,035 unique genes including 1,225 SynGO annotated genes). 254/2156 genes from our gene list were mapped to 254 unique SynGO annotated genes. 13 Cellular Component (CC) and 5 Biological Processes (BP) terms are significantly enriched at 1% FDR.

b) novel pdSNVs

GO term ID	GO domain	GO term name	GSEA 'gene cluster' FDR corrected p-value	GSEA count foreground/input	GSEA count background	Genes
GO:0050808	BP	synapse organization	2.18E-07	41	302	ADAM10;SHANK2;SHANK3;WNT7A;RAPGEF4;PAK3;NF1;PAFAH1B1;NCAN;CTNNA2;SPTB;MYH10;RIMS1;RIMS2;NEFH;MAGI2;GIT1;LRFN4;LRRC4C;NRXN1;NRG1;PLXND1;ADD2;PPFIA3;PPFIA4;CTNNB1;NLGN2;CRK;GAP43;C1QL2;NRXN3;SIPA1L1;PLXNC1;ITGB1;BCAN;CNTNAP1;PLXNA4;TENM4;ARHGEF7;NOS1AP;EZR
GO:0045202	CC	synapse	1.85E-06	90	1082	FLOT1;SLC18A1;PABPC1;RPS25;SLC6A11;NCAN;PPFIA3;PPFIA4;ARHGDIA;SLC3A2;DPYSL3;MADD;RPL30;SYT3;ITGB1;CACNB1;SEPTIN3;ADD2;NOS1AP;ARFGEF2;GAD1;GAD2;GAP43;PRKCB;GDI1;UNC13A;CNTNAP1;RIMS1;RIMS2;CTNNA2;CTNNB1;ATP2B2;ATP2B4;CANX;GRM7;CACNA1D;NRXN1;NRG1;PICK1;RAB11B;ATP6V0D1;CALCRL;SCN2A;SLC1A6;ADGRL1;GRIK5;SCN1A;CDH2;ITPR1;C1QL2;ADAM10;BEGAIN;APC2;GIT1;SIPA1L1;CNN3;SPTB;SPTAN1;AGAP2;MYO5A;PLCB1;NEFH;MYH10;SYNPO;NOS1;KCND2;NLGN2;PAK3;SHANK2;SHANK3;MAGI2;ADGRA1;TSC1;HNRNPH1;RAPGEF4;TANC2;SH3GL3;GRM1;PLPPR4;LRFN4;LRRC4C;DRD3;KCNAB2;RAB11FIP3;CNTN2;FBXO2;GABRD;KCNN2;ITGA5;BCAN
SYNGO: synprocess	BP	process in the synapse	4.78E-06	76	874	CACNA1D;GRIK5;SCN2A;SCN1A;NRXN1;ARHGDIA;RAPGEF4;CTNNB1;UNC13A;PPFIA3;RIMS1;RIMS2;PRKCB;GIT1;WNT7A;ATP6V0D1;CANX;SLC6A11;SLC1A6;FLOT1;DRD3;SYNPO;MYO5A;ATP2B2;ITPR1;GRM1;NOS1;GABRD;KCND2;BEGAIN;NRXN3;C1QL2;MAGI2;ADAM10;RAP1A;PAK3;SACM1L;ITGB1;NRG1;PICK1;ARRB2;NF1;PLCB1;TENM4;CDH2;LRRC4C;NCAN;SHANK3;KCNN2;PAFAH1B1;GRM7;NEFH;SHANK2;CTNNA2;SPTB;MYH10;LRFN4;PLXND1;ADD2;PPFIA4;NLGN2;CRK;GAP43;SIPA1L1;PLXNC1;BCAN;CNTNAP1;PLXNA4;ARHGEF7;NOS1AP;EZR;FBXO2;GAD1;GAD2;SFPQ;MADD
GO:0098794	CC	postsynapse	1.52E-05	57	621	ADAM10;BEGAIN;APC2;GIT1;SIPA1L1;CACNB1;ADD2;NOS1AP;ARFGEF2;CNN3;SPTB;SPTAN1;AGAP2;MYO5A;NRG1;PLCB1;NEFH;MYH10;SYNPO;PICK1;NOS1;KCND2;NLGN2;PAK3;SHANK2;SHANK3;MAGI2;ADGRA1;RPL30;TSC1;RPS25;HNRNPH1;RAPGEF4;CTNNA2;CTNNB1;TANC2;SH3GL3;ATP2B2;GRM1;PLPPR4;GRIK5;LRFN4;LRRC4C;DRD3;KCNAB2;RAB11FIP3;CNTN2;FBXO2;CANX;SLC6A11;SLC1A6;CDH2;ITGB1;GABRD;KCNN2;ITGA5;ITPR1
GO:0048786	CC	presynaptic active zone	2.54E-04	15	89	FLOT1;UNC13A;CNTNAP1;PPFIA3;RIMS1;RIMS2;CTNNA2;CTNNB1;ATP2B2;ATP2B4;CANX;GRM7;CACNA1D;NRXN1;NRG1
GO:0045211	CC	postsynaptic membrane	2.54E-04	17	114	CTNNB1;CNTN2;MAGI2;CTNNA2;FBXO2;ATP2B2;CANX;SLC6A11;SLC1A6;CDH2;GRIK5;GRM1;ITGB1;KCND2;GABRD;KCNN2;ITGA5
SYNGO:postsynprocess	BP	process in the postsynapse	9.34E-04	25	218	SYNPO;MYO5A;ATP2B2;ITPR1;GRM1;NOS1;GABRD;GRIK5;KCND2;BEGAIN;NRXN3;GIT1;C1QL2;MAGI2;ADAM10;RAP1A;PAK3;RAPGEF4;SACM1L;ITGB1;DRD3;NRG1;PICK1;ARRB2;NF1
GO:0007416	BP	synapse assembly	2.22E-03	14	92	ADD2;NRXN1;PPFIA3;PPFIA4;CTNNB1;NRG1;LRFN4;NLGN2;WNT7A;CRK;GAP43;C1QL2;NRXN3;SIPA1L1
GO:0098698	BP	postsynaptic specialization assembly	2.43E-03	8	32	NLGN2;CRK;GAP43;NRXN1;C1QL2;NRXN3;SIPA1L1;LRFN4
GO:0014069	CC	postsynaptic density	3.69E-03	24	251	PAK3;SHANK2;SHANK3;MAGI2;ADGRA1;RPL30;TSC1;RPS25;HNRNPH1;RAPGEF4;CTNNA2;CTNNB1;NOS1;TANC2;SH3GL3;ATP2B2;GRM1;PLPPR4;GRIK5;LRFN4;LRRC4C;DRD3;NRG1;KCNAB2
GO:0099566	BP	regulation of postsynaptic cytosolic calcium levels	4.47E-03	5	12	SYNPO;MYO5A;ATP2B2;ITPR1;GRM1
GO:0099572	CC	postsynaptic specialization	5.74E-03	27	311	PICK1;NOS1;KCND2;NLGN2;PAK3;SHANK2;SHANK3;MAGI2;ADGRA1;RPL30;TSC1;RPS25;HNRNPH1;RAPGEF4;CTNNA2;CTNNB1;TANC2;SH3GL3;ATP2B2;GRM1;PLPPR4;GRIK5;LRFN4;LRRC4C;DRD3;NRG1;KCNAB2
GO:0099055	CC	integral component of postsynaptic membrane	7.32E-03	12	97	ATP2B2;CANX;SLC6A11;SLC1A6;CDH2;GRIK5;GRM1;ITGB1;KCND2;GABRD;KCNN2;ITGA5
GO:0099645	BP	neurotransmitter receptor localization to postsynaptic specialization membrane	8.42E-03	8	44	GIT1;C1QL2;NRXN3;MAGI2;ADAM10;RAP1A;PAK3;RAPGEF4
GO:0099175	BP	regulation of postsynapse organization	8.42E-03	9	54	SHANK2;SHANK3;WNT7A;RAPGEF4;ADAM10;PAK3;NF1;PAFAH1B1;NCAN
GO:0098918	BP	structural constituent of synapse	8.42E-03	7	34	RIMS1;RIMS2;NEFH;MAGI2;GIT1;SHANK3;SHANK2
GO:0099188	BP	postsynaptic cytoskeleton organization	8.42E-03	6	25	SIPA1L1;ARHGEF7;NOS1AP;MYH10;EZR;TENM4

The table shows all SynGO ontology terms enriched in our list of 823 genes harbouring novel pdSNVs in cases as compared to the “brain expressed" background set (18,035 unique genes including 1,225 SynGO annotated genes). 105/823 genes from our gene list were mapped to 105 unique SynGO annotated genes. 7 Cellular Component (CC) and 10 Biological Processes (BP) terms are significantly enriched at 1% FDR.

c) novel pdSNVs not shared with unaffected siblings

GO term ID	GO domain	GO term name	GSEA 'gene cluster' FDR corrected p-value	GSEA count foreground/input	GSEA count background	Genes
GO:0050808	BP	synapse organization	7.55E-07	34	302	ADAM10;SHANK2;SHANK3;WNT7A;RAPGEF4;NF1;PAFAH1B1;NCAN;SPTB;RIMS1;RIMS2;NEFH;MAGI2;GIT1;LRRC4C;NRXN1;NRG1;PLXND1;ADD2;PPFIA4;CTNNB1;NLGN2;CRK;GAP43;C1QL2;NRXN3;SIPA1L1;ITGB1;BCAN;PLXNA4;TENM4;ARHGEF7;NOS1AP;EZR
SYNGO: synprocess	BP	process in the synapse	1.19E-06	65	874	CACNA1D;GRIK5;SCN2A;SCN1A;NRXN1;ARHGDIA;RAPGEF4;CTNNB1;UNC13A;RIMS1;RIMS2;PRKCB;GIT1;WNT7A;CANX;SLC6A11;SLC1A6;FLOT1;SYNPO;MYO5A;ATP2B2;ITPR1;GABRD;KCND2;BEGAIN;NRXN3;C1QL2;MAGI2;ADAM10;RAP1A;SACM1L;ITGB1;NRG1;PICK1;ARRB2;NF1;PLCB1;TENM4;CDH2;LRRC4C;NCAN;SHANK3;KCNN2;PAFAH1B1;GRM7;NEFH;SHANK2;SPTB;PLXND1;ADD2;PPFIA4;NLGN2;CRK;GAP43;SIPA1L1;BCAN;PLXNA4;ARHGEF7;NOS1AP;EZR;FBXO2;GAD1;GAD2;SFPQ;MADD
GO:0045202	CC	synapse	2.89E-06	74	1082	FLOT1;SLC18A1;PABPC1;RPS25;SLC6A11;NCAN;PPFIA4;ARHGDIA;SLC3A2;DPYSL3;MADD;SYT3;ITGB1;CACNB1;SEPTIN3;ADD2;NOS1AP;ARFGEF2;GAD1;GAD2;GAP43;PRKCB;GDI1;UNC13A;RIMS1;RIMS2;CTNNB1;ATP2B2;ATP2B4;CANX;GRM7;CACNA1D;NRXN1;NRG1;PICK1;RAB11B;CALCRL;SCN2A;SLC1A6;GRIK5;SCN1A;CDH2;ITPR1;C1QL2;ADAM10;BEGAIN;APC2;GIT1;SIPA1L1;SPTB;SPTAN1;AGAP2;MYO5A;PLCB1;NEFH;SYNPO;KCND2;NLGN2;SHANK2;SHANK3;MAGI2;ADGRA1;TSC1;RAPGEF4;TANC2;SH3GL3;PLPPR4;LRRC4C;CNTN2;FBXO2;GABRD;KCNN2;ITGA5;BCAN
GO:0098794	CC	postsynapse	1.09E-04	45	621	ADAM10;BEGAIN;APC2;GIT1;SIPA1L1;CACNB1;ADD2;NOS1AP;ARFGEF2;SPTB;SPTAN1;AGAP2;MYO5A;NRG1;PLCB1;NEFH;SYNPO;PICK1;KCND2;NLGN2;SHANK2;SHANK3;MAGI2;ADGRA1;TSC1;RPS25;RAPGEF4;CTNNB1;TANC2;SH3GL3;ATP2B2;PLPPR4;GRIK5;LRRC4C;CNTN2;FBXO2;CANX;SLC6A11;SLC1A6;CDH2;ITGB1;GABRD;KCNN2;ITGA5;ITPR1
GO:0045211	CC	postsynaptic membrane	1.97E-04	15	114	CTNNB1;CNTN2;MAGI2;FBXO2;ATP2B2;CANX;SLC6A11;SLC1A6;CDH2;GRIK5;ITGB1;KCND2;GABRD;KCNN2;ITGA5
GO:0048786	CC	presynaptic active zone	7.01E-04	12	89	FLOT1;UNC13A;RIMS1;RIMS2;CTNNB1;ATP2B2;ATP2B4;CANX;GRM7;CACNA1D;NRXN1;NRG1
SYNGO:postsynprocess	BP	process in the postsynapse	1.01E-03	21	218	SYNPO;MYO5A;ATP2B2;ITPR1;GABRD;GRIK5;KCND2;BEGAIN;NRXN3;GIT1;C1QL2;MAGI2;ADAM10;RAP1A;RAPGEF4;SACM1L;ITGB1;NRG1;PICK1;ARRB2;NF1
GO:0007416	BP	synapse assembly	2.10E-03	12	92	ADD2;NRXN1;PPFIA4;CTNNB1;NRG1;NLGN2;WNT7A;CRK;GAP43;C1QL2;NRXN3;SIPA1L1
GO:0098698	BP	postsynaptic specialization assembly	2.26E-03	7	32	NLGN2;CRK;GAP43;NRXN1;C1QL2;NRXN3;SIPA1L1
GO:0098918	BP	structural constituent of synapse	2.59E-03	7	34	RIMS1;RIMS2;NEFH;MAGI2;GIT1;SHANK3;SHANK2
GO:0099055	CC	integral component of postsynaptic membrane	3.81E-03	11	97	ATP2B2;CANX;SLC6A11;SLC1A6;CDH2;GRIK5;ITGB1;KCND2;GABRD;KCNN2;ITGA5
GO:0048787	CC	presynaptic active zone membrane	4.21E-03	8	57	RIMS1;ATP2B2;ATP2B4;CANX;GRM7;CACNA1D;NRXN1;NRG1
GO:0099059	CC	integral component of presynaptic active zone membrane	4.21E-03	7	45	ATP2B2;ATP2B4;CANX;GRM7;CACNA1D;NRXN1;NRG1
GO:0098793	CC	presynapse	4.36E-03	33	531	CACNB1;SEPTIN3;FLOT1;ADD2;NOS1AP;ARFGEF2;GAD1;GAD2;GAP43;PPFIA4;PRKCB;GDI1;UNC13A;RIMS1;RIMS2;CTNNB1;ATP2B2;ATP2B4;CANX;GRM7;CACNA1D;NRXN1;NRG1;PICK1;RAB11B;CALCRL;SCN2A;SLC6A11;SLC1A6;GRIK5;SCN1A;CDH2;ITPR1
GO:0099175	BP	regulation of postsynapse organization	6.14E-03	8	54	SHANK2;SHANK3;WNT7A;RAPGEF4;ADAM10;NF1;PAFAH1B1;NCAN
GO:0099566	BP	regulation of postsynaptic cytosolic calcium levels	6.95E-03	4	12	SYNPO;MYO5A;ATP2B2;ITPR1
GO:0099645	BP	neurotransmitter receptor localization to postsynaptic specialization membrane	6.95E-03	7	44	GIT1;C1QL2;NRXN3;MAGI2;ADAM10;RAP1A;RAPGEF4
GO:0099186	BP	structural constituent of postsynapse	9.81E-03	5	24	NEFH;MAGI2;GIT1;SHANK3;SHANK2

The table shows all SynGO ontology terms enriched in our list of 645 genes harbouring novel pdSNVs not shared with unaffected siblings in cases as compared to the “brain expressed" background set (18,035 unique genes including 1,225 SynGO annotated genes). 88/645 genes from our gene list were mapped to 88 unique SynGO annotated genes. 8 Cellular Component (CC) and 10 Biological Processes (BP) terms are significantly enriched at 1% FDR.

Supplementary Table 8: List of high-confidence ASD/NDD genes

	Fu et al, 2022				Trost et al, 2022	This study					Leblond et al, 2021
Gene	ASD72 (FDR ≤0.001)	ASD185 (FDR ≤0.05)	NDD373 (FDR ≤0.001)	NDD664 (FDR ≤0.05)	ASD135 (FDR <0.1)	Gene category	Nr of rare pdSNVs in 144 cases	Nr of rare pdSNVs in 55 unaffected sibs	Nr of novel pdSNVs in 144 cases	Nr of novel pdSNVs in 55 unaffected sibs	GeneTrek classification
ANK2	TRUE	TRUE	TRUE	TRUE	TRUE	ASD	0	1	0	1	HC-NDD
ANKRD11	TRUE	TRUE	TRUE	TRUE	TRUE	ASD	1	0	0	0	HC-NDD
ARID1B	TRUE	TRUE	TRUE	TRUE	TRUE	ASD	1	0	0	0	HC-NDD
ASH1L	TRUE	TRUE	TRUE	TRUE	TRUE	ASD	1	0	0	0	HC-NDD
ATP2B2	TRUE	TRUE	TRUE	TRUE	TRUE	ASD	5	1	4	0	Candidate-NDD
BRSK2	TRUE	TRUE	TRUE	TRUE	TRUE	ASD	3	0	2	0	HC-NDD
CHD2	TRUE	TRUE	TRUE	TRUE	TRUE	ASD	2	0	0	0	HC-NDD
CHD8	TRUE	TRUE	TRUE	TRUE	TRUE	ASD	2	0	0	0	HC-NDD
CTCF	TRUE	TRUE	TRUE	TRUE	TRUE	ASD	2	2	2	2	HC-NDD
CTNNB1	TRUE	TRUE	TRUE	TRUE	TRUE	ASD	1	1	1	0	HC-NDD
DYNC1H1	TRUE	TRUE	TRUE	TRUE	TRUE	ASD	4	0	4	0	HC-NDD
EBF3	TRUE	TRUE	TRUE	TRUE	TRUE	ASD	1	0	0	0	HC-NDD
EHMT1	TRUE	TRUE	TRUE	TRUE	TRUE	ASD	1	2	0	0	HC-NDD
GRIN2B	TRUE	TRUE	TRUE	TRUE	TRUE	ASD	2	2	0	0	HC-NDD
KCNQ3	TRUE	TRUE	TRUE	TRUE	TRUE	ASD	1	0	0	0	HC-NDD
KMT2A	TRUE	TRUE	TRUE	TRUE	TRUE	ASD	1	2	0	0	HC-NDD
NF1	TRUE	TRUE	TRUE	TRUE	TRUE	ASD	1	0	1	0	HC-NDD
RFX3	TRUE	TRUE	TRUE	TRUE	TRUE	ASD	2	0	2	0	HC-NDD
RORB	TRUE	TRUE	TRUE	TRUE	TRUE	ASD	1	0	0	0	HC-NDD
SCN2A	TRUE	TRUE	TRUE	TRUE	TRUE	ASD	1	0	1	0	HC-NDD
SHANK3	TRUE	TRUE	TRUE	TRUE	TRUE	ASD	2	0	2	0	HC-NDD
SLC6A1	TRUE	TRUE	TRUE	TRUE	TRUE	ASD	1	1	0	0	HC-NDD
SMARCC2	TRUE	TRUE	TRUE	TRUE	TRUE	ASD	2	1	0	0	HC-NDD
TCF7L2	TRUE	TRUE	TRUE	TRUE	TRUE	ASD	0	1	0	0	HC-NDD
WAC	TRUE	TRUE	TRUE	TRUE	TRUE	ASD	0	1	0	0	HC-NDD
CASKIN1	TRUE	TRUE	TRUE	TRUE	FALSE	ASD	0	1	0	0	Candidate-NDD
DSCAM	TRUE	TRUE	TRUE	TRUE	FALSE	ASD	2	0	1	0	HC-NDD
KCNMA1	TRUE	TRUE	TRUE	TRUE	FALSE	ASD	1	1	0	0	HC-NDD
KMT2C	TRUE	TRUE	TRUE	TRUE	FALSE	ASD	1	0	1	0	HC-NDD
NIPBL	TRUE	TRUE	TRUE	TRUE	FALSE	ASD	1	0	0	0	HC-NDD
NRXN1	TRUE	TRUE	TRUE	TRUE	FALSE	ASD	2	0	1	0	HC-NDD
PLXNA1	TRUE	TRUE	TRUE	TRUE	FALSE	ASD	3	0	2	0	HC-NDD
SCN1A	TRUE	TRUE	TRUE	TRUE	FALSE	ASD	2	0	1	0	HC-NDD
ZBTB20	TRUE	TRUE	TRUE	TRUE	FALSE	ASD	1	0	0	0	HC-NDD
APBB1	FALSE	TRUE	FALSE	TRUE	TRUE	ASD	1	0	0	0	Candidate-NDD
CAPRIN1	FALSE	TRUE	TRUE	TRUE	TRUE	ASD	0	1	0	1	HC-NDD
DPYSL2	FALSE	TRUE	FALSE	TRUE	TRUE	ASD	1	0	0	0	HC-NDD
GRIA2	FALSE	TRUE	TRUE	TRUE	TRUE	ASD	1	0	0	0	HC-NDD
SHANK2	FALSE	TRUE	TRUE	TRUE	TRUE	ASD	1	0	1	0	HC-NDD
TLE3	FALSE	TRUE	TRUE	TRUE	TRUE	ASD	0	1	0	1	Candidate-NDD
TRIP12	FALSE	TRUE	TRUE	TRUE	TRUE	ASD	0	1	0	0	HC-NDD
TSC1	FALSE	TRUE	TRUE	TRUE	TRUE	ASD	3	0	3	0	HC-NDD
UBR1	FALSE	TRUE	FALSE	TRUE	TRUE	ASD	0	1	0	0	HC-NDD
ZMYND11	FALSE	TRUE	TRUE	TRUE	TRUE	ASD	1	0	0	0	HC-NDD
ZMYND8	FALSE	TRUE	TRUE	TRUE	TRUE	ASD	1	0	0	0	HC-NDD
ACVR2A	FALSE	TRUE	TRUE	TRUE	FALSE	ASD	1	0	1	0	Candidate-NDD
AGO4	FALSE	TRUE	FALSE	TRUE	FALSE	ASD	2	0	2	0	Candidate-NDD
AHDC1	FALSE	TRUE	TRUE	TRUE	FALSE	ASD	5	0	0	0	HC-NDD
ANKH	FALSE	TRUE	FALSE	TRUE	FALSE	ASD	1	0	0	0	HC-NDD
ATP4A	FALSE	TRUE	FALSE	TRUE	FALSE	ASD	0	2	0	0	.
BTG3	FALSE	TRUE	FALSE	TRUE	FALSE	ASD	0	1	0	0	Candidate-NDD
CACNA1D	FALSE	TRUE	FALSE	TRUE	FALSE	ASD	1	0	1	0	HC-NDD
CDK13	FALSE	TRUE	TRUE	TRUE	FALSE	ASD	1	0	1	0	HC-NDD
CELF1	FALSE	TRUE	FALSE	TRUE	FALSE	ASD	1	1	0	0	Candidate-NDD
CHD3	FALSE	TRUE	TRUE	TRUE	FALSE	ASD	2	2	1	1	HC-NDD
CTNNA2	FALSE	TRUE	TRUE	TRUE	FALSE	ASD	1	1	1	1	HC-NDD
CYFIP2	FALSE	TRUE	FALSE	TRUE	FALSE	ASD	1	0	0	0	HC-NDD
DIP2A	FALSE	TRUE	FALSE	TRUE	FALSE	ASD	2	2	1	1	HC-NDD
DIP2C	FALSE	TRUE	FALSE	TRUE	FALSE	ASD	0	1	0	1	Candidate-NDD
GABBR2	FALSE	TRUE	TRUE	TRUE	FALSE	ASD	1	0	0	0	HC-NDD
HDAC4	FALSE	TRUE	TRUE	TRUE	FALSE	ASD	1	0	0	0	HC-NDD
HECTD4	FALSE	TRUE	TRUE	TRUE	FALSE	ASD	2	1	1	0	HC-NDD
INTS3	FALSE	TRUE	FALSE	TRUE	FALSE	ASD	3	2	1	1	Candidate-NDD
ITPR3	FALSE	TRUE	FALSE	TRUE	FALSE	ASD	2	1	0	1	Candidate-NDD
KIF1B	FALSE	TRUE	FALSE	TRUE	FALSE	ASD	1	1	1	1	Candidate-NDD
MAPK8IP3	FALSE	TRUE	TRUE	TRUE	FALSE	ASD	3	1	2	1	HC-NDD
NCKAP1	FALSE	TRUE	FALSE	TRUE	FALSE	ASD	1	0	0	0	HC-NDD
NRXN3	FALSE	TRUE	FALSE	TRUE	FALSE	ASD	6	2	2	1	HC-NDD
OBSCN	FALSE	TRUE	FALSE	TRUE	FALSE	ASD	1	1	0	1	Candidate-NDD
PRDM16	FALSE	TRUE	FALSE	TRUE	FALSE	ASD	0	2	0	0	Candidate-NDD
PRR12	FALSE	TRUE	TRUE	TRUE	FALSE	ASD	2	0	1	0	HC-NDD
PSMD12	FALSE	TRUE	FALSE	TRUE	FALSE	ASD	1	0	0	0	HC-NDD
PTPRF	FALSE	TRUE	FALSE	TRUE	FALSE	ASD	1	0	0	0	Candidate-NDD
RAB11FIP4	FALSE	TRUE	FALSE	TRUE	FALSE	ASD	1	0	1	0	Candidate-NDD
RALGAPB	FALSE	TRUE	FALSE	TRUE	FALSE	ASD	2	0	0	0	HC-NDD
SIN3A	FALSE	TRUE	TRUE	TRUE	FALSE	ASD	1	0	0	0	HC-NDD
SLC6A2	FALSE	TRUE	FALSE	TRUE	FALSE	ASD	0	1	0	0	Candidate-NDD
SLC9A3	FALSE	TRUE	FALSE	TRUE	FALSE	ASD	3	1	1	0	Candidate-NDD
SMARCA2	FALSE	TRUE	TRUE	TRUE	FALSE	ASD	1	1	1	1	HC-NDD
SPEN	FALSE	TRUE	TRUE	TRUE	FALSE	ASD	1	1	0	0	HC-NDD
TAF4	FALSE	TRUE	FALSE	TRUE	FALSE	ASD	2	0	0	0	Candidate-NDD
UBR5	FALSE	TRUE	TRUE	TRUE	FALSE	ASD	3	0	3	0	Candidate-NDD
XPO1	FALSE	TRUE	TRUE	TRUE	FALSE	ASD	0	1	0	1	Candidate-NDD
YWHAG	FALSE	TRUE	TRUE	TRUE	FALSE	ASD	1	0	0	0	HC-NDD
CELF4	FALSE	FALSE	FALSE	TRUE	TRUE	ASD	1	0	1	0	HC-NDD
CORO2B	FALSE	FALSE	FALSE	FALSE	TRUE	ASD	1	0	0	0	Candidate-NDD
DMPK	FALSE	FALSE	FALSE	TRUE	TRUE	ASD	1	0	0	0	HC-NDD
EPOR	FALSE	FALSE	FALSE	FALSE	TRUE	ASD	2	0	0	0	.
ITGB4	FALSE	FALSE	FALSE	FALSE	TRUE	ASD	1	1	0	0	Candidate-NDD
KIF5C	FALSE	FALSE	TRUE	TRUE	TRUE	ASD	1	0	0	0	HC-NDD
PLXNB1	FALSE	FALSE	FALSE	FALSE	TRUE	ASD	3	0	2	0	Candidate-NDD
PSMD6	FALSE	FALSE	FALSE	FALSE	TRUE	ASD	2	1	1	0	Candidate-NDD
RUNX1T1	FALSE	FALSE	FALSE	TRUE	TRUE	ASD	1	0	1	0	Candidate-NDD
TANC2	FALSE	FALSE	TRUE	TRUE	TRUE	ASD	1	0	1	0	HC-NDD
TBCEL	FALSE	FALSE	FALSE	TRUE	TRUE	ASD	1	0	1	0	Candidate-NDD

TRAF7	FALSE	FALSE	TRUE	TRUE	TRUE	ASD	1	0	0	0	HC-NDD
WDFY3	FALSE	FALSE	FALSE	TRUE	TRUE	ASD	1	0	0	0	HC-NDD
ADNP	TRUE	TRUE	TRUE	TRUE	TRUE	ASD	0	0	0	0	HC-NDD
AP2S1	TRUE	TRUE	TRUE	TRUE	TRUE	ASD	0	0	0	0	HC-NDD
ASXL3	TRUE	TRUE	TRUE	TRUE	TRUE	ASD	0	0	0	0	HC-NDD
AUTS2	TRUE	TRUE	TRUE	TRUE	TRUE	ASD	0	0	0	0	HC-NDD
CUL3	TRUE	TRUE	TRUE	TRUE	TRUE	ASD	0	0	0	0	HC-NDD
DEAF1	TRUE	TRUE	TRUE	TRUE	TRUE	ASD	0	0	0	0	HC-NDD
DLG4	TRUE	TRUE	TRUE	TRUE	TRUE	ASD	0	0	0	0	HC-NDD
DNMT3A	TRUE	TRUE	TRUE	TRUE	TRUE	ASD	0	0	0	0	HC-NDD
DYRK1A	TRUE	TRUE	TRUE	TRUE	TRUE	ASD	0	0	0	0	HC-NDD
FOXP1	TRUE	TRUE	TRUE	TRUE	TRUE	ASD	0	0	0	0	HC-NDD
GABRA1	TRUE	TRUE	TRUE	TRUE	TRUE	ASD	0	0	0	0	HC-NDD
GIGYF1	TRUE	TRUE	TRUE	TRUE	TRUE	ASD	0	0	0	0	HC-NDD
IRF2BPL	TRUE	TRUE	TRUE	TRUE	TRUE	ASD	0	0	0	0	HC-NDD
KCNB1	TRUE	TRUE	TRUE	TRUE	TRUE	ASD	0	0	0	0	HC-NDD
KDM5B	TRUE	TRUE	TRUE	TRUE	TRUE	ASD	0	0	0	0	HC-NDD
KDM6B	TRUE	TRUE	TRUE	TRUE	TRUE	ASD	0	0	0	0	HC-NDD
MBD5	TRUE	TRUE	TRUE	TRUE	TRUE	ASD	0	0	0	0	HC-NDD
MED13	TRUE	TRUE	TRUE	TRUE	TRUE	ASD	0	0	0	0	HC-NDD
MED13L	TRUE	TRUE	TRUE	TRUE	TRUE	ASD	0	0	0	0	HC-NDD
MEIS2	TRUE	TRUE	TRUE	TRUE	TRUE	ASD	0	0	0	0	HC-NDD
NAA15	TRUE	TRUE	TRUE	TRUE	TRUE	ASD	0	0	0	0	HC-NDD
PHF21A	TRUE	TRUE	TRUE	TRUE	TRUE	ASD	0	0	0	0	HC-NDD
POGZ	TRUE	TRUE	TRUE	TRUE	TRUE	ASD	0	0	0	0	HC-NDD
PTEN	TRUE	TRUE	TRUE	TRUE	TRUE	ASD	0	0	0	0	HC-NDD
SATB2	TRUE	TRUE	TRUE	TRUE	TRUE	ASD	0	0	0	0	HC-NDD
SETD5	TRUE	TRUE	TRUE	TRUE	TRUE	ASD	0	0	0	0	HC-NDD
SKI	TRUE	TRUE	TRUE	TRUE	TRUE	ASD	0	0	0	0	HC-NDD
STXBP1	TRUE	TRUE	TRUE	TRUE	TRUE	ASD	0	0	0	0	HC-NDD
SUV420H1	TRUE	TRUE	TRUE	TRUE	TRUE	ASD	0	0	0	0	HC-NDD
SYNGAP1	TRUE	TRUE	TRUE	TRUE	TRUE	ASD	0	0	0	0	HC-NDD
TAOK1	TRUE	TRUE	TRUE	TRUE	TRUE	ASD	0	0	0	0	HC-NDD
TCF20	TRUE	TRUE	TRUE	TRUE	TRUE	ASD	0	0	0	0	HC-NDD
TLK2	TRUE	TRUE	TRUE	TRUE	TRUE	ASD	0	0	0	0	HC-NDD
VEZF1	TRUE	TRUE	TRUE	TRUE	TRUE	ASD	0	0	0	0	HC-NDD
DLL1	TRUE	TRUE	TRUE	TRUE	FALSE	ASD	0	0	0	0	HC-NDD
MED23	TRUE	TRUE	TRUE	TRUE	FALSE	ASD	0	0	0	0	HC-NDD
PIK3CA	TRUE	TRUE	TRUE	TRUE	FALSE	ASD	0	0	0	0	HC-NDD
SPAST	TRUE	TRUE	TRUE	TRUE	FALSE	ASD	0	0	0	0	HC-NDD
CAMTA2	FALSE	TRUE	TRUE	TRUE	TRUE	ASD	0	0	0	0	Candidate-NDD
CSNK2A1	FALSE	TRUE	TRUE	TRUE	TRUE	ASD	0	0	0	0	HC-NDD
HNRNPD	FALSE	TRUE	TRUE	TRUE	TRUE	ASD	0	0	0	0	HC-NDD
HNRNPUL2	FALSE	TRUE	FALSE	TRUE	TRUE	ASD	0	0	0	0	HC-NDD
MARK2	FALSE	TRUE	TRUE	TRUE	TRUE	ASD	0	0	0	0	Candidate-NDD
MYT1L	FALSE	TRUE	TRUE	TRUE	TRUE	ASD	0	0	0	0	HC-NDD
PAX5	FALSE	TRUE	TRUE	TRUE	TRUE	ASD	0	0	0	0	HC-NDD
PBX1	FALSE	TRUE	TRUE	TRUE	TRUE	ASD	0	0	0	0	HC-NDD
PHF12	FALSE	TRUE	TRUE	TRUE	TRUE	ASD	0	0	0	0	HC-NDD
PSMD11	FALSE	TRUE	FALSE	TRUE	TRUE	ASD	0	0	0	0	Candidate-NDD
RAI1	FALSE	TRUE	TRUE	TRUE	TRUE	ASD	0	0	0	0	HC-NDD
SRPR	FALSE	TRUE	TRUE	TRUE	TRUE	ASD	0	0	0	0	HC-NDD
SYNCRIP	FALSE	TRUE	TRUE	TRUE	TRUE	ASD	0	0	0	0	HC-NDD
SYT1	FALSE	TRUE	TRUE	TRUE	TRUE	ASD	0	0	0	0	HC-NDD
TBL1XR1	FALSE	TRUE	TRUE	TRUE	TRUE	ASD	0	0	0	0	HC-NDD
TBR1	FALSE	TRUE	TRUE	TRUE	TRUE	ASD	0	0	0	0	HC-NDD
TCF4	FALSE	TRUE	TRUE	TRUE	TRUE	ASD	0	0	0	0	HC-NDD
TNPO3	FALSE	TRUE	TRUE	TRUE	TRUE	ASD	0	0	0	0	Candidate-NDD
ACLY	FALSE	TRUE	FALSE	TRUE	FALSE	ASD	0	0	0	0	Candidate-NDD
ADCY5	FALSE	TRUE	TRUE	TRUE	FALSE	ASD	0	0	0	0	Candidate-NDD
ATG13	FALSE	TRUE	FALSE	TRUE	FALSE	ASD	0	0	0	0	Candidate-NDD
ATG9B	FALSE	TRUE	FALSE	TRUE	FALSE	ASD	0	0	0	0	.
ATP1A3	FALSE	TRUE	TRUE	TRUE	FALSE	ASD	0	0	0	0	HC-NDD
C16orf72	FALSE	TRUE	FALSE	TRUE	FALSE	ASD	0	0	0	0	Candidate-NDD
CACNA1E	FALSE	TRUE	TRUE	TRUE	FALSE	ASD	0	0	0	0	HC-NDD
CAMK2A	FALSE	TRUE	TRUE	TRUE	FALSE	ASD	0	0	0	0	HC-NDD
CELF2	FALSE	TRUE	TRUE	TRUE	FALSE	ASD	0	0	0	0	HC-NDD
CGREF1	FALSE	TRUE	FALSE	TRUE	FALSE	ASD	0	0	0	0	Candidate-NDD
CORO1A	FALSE	TRUE	FALSE	TRUE	FALSE	ASD	0	0	0	0	HC-NDD
CREBBP	FALSE	TRUE	TRUE	TRUE	FALSE	ASD	0	0	0	0	HC-NDD
CSNK1E	FALSE	TRUE	TRUE	TRUE	FALSE	ASD	0	0	0	0	Candidate-NDD
CYLD	FALSE	TRUE	FALSE	TRUE	FALSE	ASD	0	0	0	0	Candidate-NDD
DEDD	FALSE	TRUE	FALSE	TRUE	FALSE	ASD	0	0	0	0	Candidate-NDD
EFTUD2	FALSE	TRUE	TRUE	TRUE	FALSE	ASD	0	0	0	0	HC-NDD
FAM120A	FALSE	TRUE	FALSE	TRUE	FALSE	ASD	0	0	0	0	Candidate-NDD
FBXO11	FALSE	TRUE	TRUE	TRUE	FALSE	ASD	0	0	0	0	HC-NDD
GABRB3	FALSE	TRUE	TRUE	TRUE	FALSE	ASD	0	0	0	0	HC-NDD
H3F3A	FALSE	TRUE	TRUE	TRUE	FALSE	ASD	0	0	0	0	HC-NDD
INO80D	FALSE	TRUE	FALSE	TRUE	FALSE	ASD	0	0	0	0	Candidate-NDD
ITSN1	FALSE	TRUE	FALSE	TRUE	FALSE	ASD	0	0	0	0	HC-NDD
KCNH7	FALSE	TRUE	FALSE	TRUE	FALSE	ASD	0	0	0	0	.
KDM3B	FALSE	TRUE	TRUE	TRUE	FALSE	ASD	0	0	0	0	HC-NDD
KPNA1	FALSE	TRUE	FALSE	TRUE	FALSE	ASD	0	0	0	0	Candidate-NDD
MEF2C	FALSE	TRUE	TRUE	TRUE	FALSE	ASD	0	0	0	0	HC-NDD
MLXIP	FALSE	FALSE	FALSE	TRUE	FALSE	ASD	0	0	0	0	Candidate-NDD
MRFAP1	FALSE	TRUE	FALSE	TRUE	FALSE	ASD	1	0	0	0	.
MYO18A	FALSE	TRUE	FALSE	TRUE	FALSE	ASD	0	0	0	0	Candidate-NDD
NAA25	FALSE	TRUE	FALSE	TRUE	FALSE	ASD	0	0	0	0	Candidate-NDD
NACC1	FALSE	TRUE	TRUE	TRUE	FALSE	ASD	0	0	0	0	HC-NDD
NAV3	FALSE	TRUE	FALSE	TRUE	FALSE	ASD	0	0	0	0	Candidate-NDD
PACS1	FALSE	TRUE	TRUE	TRUE	FALSE	ASD	0	0	0	0	HC-NDD
PHF2	FALSE	TRUE	FALSE	TRUE	FALSE	ASD	0	0	0	0	HC-NDD
PPP1R9B	FALSE	TRUE	FALSE	TRUE	FALSE	ASD	0	0	0	0	HC-NDD
PRH1	FALSE	TRUE	FALSE	TRUE	FALSE	ASD	0	0	0	0	.
PTGDR2	FALSE	TRUE	FALSE	TRUE	FALSE	ASD	0	0	0	0	.
ROPN1	FALSE	TRUE	FALSE	TRUE	FALSE	ASD	0	0	0	0	.
SYK	FALSE	TRUE	FALSE	TRUE	FALSE	ASD	0	0	0	0	Candidate-NDD
TLE4	FALSE	TRUE	FALSE	TRUE	FALSE	ASD	0	0	0	0	Candidate-NDD
TRAF2	FALSE	TRUE	FALSE	TRUE	FALSE	ASD	0	0	0	0	Candidate-NDD
USP49	FALSE	TRUE	FALSE	TRUE	FALSE	ASD	0	0	0	0	Candidate-NDD
ZMYM2	FALSE	TRUE	TRUE	TRUE	FALSE	ASD	0	0	0	0	HC-NDD

ZNF292	FALSE	TRUE	TRUE	TRUE	FALSE	ASD	0	0	0	0	HC-NDD
ZNF462	FALSE	TRUE	TRUE	TRUE	FALSE	ASD	0	0	0	0	HC-NDD
ABCE1	FALSE	FALSE	FALSE	FALSE	TRUE	ASD	0	0	0	0	Candidate-NDD
ANP32A	FALSE	FALSE	FALSE	FALSE	TRUE	ASD	0	0	0	0	Candidate-NDD
ARF3	FALSE	FALSE	FALSE	TRUE	TRUE	ASD	0	0	0	0	Candidate-NDD
BCL11A	FALSE	FALSE	TRUE	TRUE	TRUE	ASD	0	0	0	0	HC-NDD
CASZ1	FALSE	FALSE	TRUE	TRUE	TRUE	ASD	0	0	0	0	HC-NDD
CNOT3	FALSE	FALSE	TRUE	TRUE	TRUE	ASD	0	0	0	0	HC-NDD
CPSF7	FALSE	FALSE	FALSE	TRUE	TRUE	ASD	0	0	0	0	Candidate-NDD
DMWD	FALSE	FALSE	FALSE	FALSE	TRUE	ASD	0	0	0	0	Candidate-NDD
EIF4E	FALSE	FALSE	FALSE	FALSE	TRUE	ASD	0	0	0	0	Candidate-NDD
ENC1	FALSE	FALSE	FALSE	FALSE	TRUE	ASD	0	0	0	0	Candidate-NDD
FUBP3	FALSE	FALSE	FALSE	FALSE	TRUE	ASD	0	0	0	0	.
G3BP1	FALSE	FALSE	FALSE	FALSE	TRUE	ASD	0	0	0	0	.
GNAI1	FALSE	FALSE	TRUE	TRUE	TRUE	ASD	0	0	0	0	HC-NDD
LDB1	FALSE	FALSE	FALSE	TRUE	TRUE	ASD	0	0	0	0	HC-NDD
MAP1A	FALSE	FALSE	FALSE	FALSE	TRUE	ASD	0	0	0	0	HC-NDD
MBD6	FALSE	FALSE	FALSE	FALSE	TRUE	ASD	0	0	0	0	Candidate-NDD
MIB1	FALSE	FALSE	TRUE	TRUE	TRUE	ASD	0	0	0	0	Candidate-NDD
MKX	FALSE	FALSE	FALSE	TRUE	TRUE	ASD	0	0	0	0	HC-NDD
MSL2	FALSE	FALSE	TRUE	TRUE	TRUE	ASD	0	0	0	0	Candidate-NDD
NR4A2	FALSE	FALSE	TRUE	TRUE	TRUE	ASD	0	0	0	0	HC-NDD
NUP155	FALSE	FALSE	FALSE	FALSE	TRUE	ASD	0	0	0	0	HC-NDD
PAPOLG	FALSE	FALSE	FALSE	TRUE	TRUE	ASD	0	0	0	0	Candidate-NDD
PHF3	FALSE	FALSE	FALSE	FALSE	TRUE	ASD	0	0	0	0	HC-NDD
PPP2R5D	FALSE	FALSE	TRUE	TRUE	TRUE	ASD	0	0	0	0	HC-NDD
PRKG2	FALSE	FALSE	FALSE	FALSE	TRUE	ASD	0	0	0	0	Candidate-NDD
PRR14L	FALSE	FALSE	FALSE	FALSE	TRUE	ASD	0	0	0	0	Candidate-NDD
QRICH1	FALSE	FALSE	TRUE	TRUE	TRUE	ASD	0	0	0	0	HC-NDD
SETD1A	FALSE	FALSE	TRUE	TRUE	TRUE	ASD	0	0	0	0	HC-NDD
SOX5	FALSE	FALSE	TRUE	TRUE	TRUE	ASD	0	0	0	0	HC-NDD
SPRY2	FALSE	FALSE	FALSE	TRUE	TRUE	ASD	0	0	0	0	Candidate-NDD
TERF2	FALSE	FALSE	FALSE	FALSE	TRUE	ASD	0	0	0	0	Candidate-NDD
TM9SF4	FALSE	FALSE	FALSE	TRUE	TRUE	ASD	0	0	0	0	HC-NDD
TSHZ1	FALSE	FALSE	FALSE	FALSE	TRUE	ASD	0	0	0	0	Candidate-NDD
ZBTB21	FALSE	FALSE	FALSE	TRUE	TRUE	ASD	0	0	0	0	Candidate-NDD
ABL1	FALSE	FALSE	TRUE	TRUE	FALSE	NDD	1	1	0	0	Candidate-NDD
AP3B2	FALSE	FALSE	TRUE	TRUE	FALSE	NDD	1	0	0	0	HC-NDD
ARID2	FALSE	FALSE	TRUE	TRUE	FALSE	NDD	2	0	1	0	HC-NDD
ASXL1	FALSE	FALSE	TRUE	TRUE	FALSE	NDD	1	0	1	0	HC-NDD
BRD4	FALSE	FALSE	TRUE	TRUE	FALSE	NDD	1	1	0	0	HC-NDD
BRPF1	FALSE	FALSE	TRUE	TRUE	FALSE	NDD	1	1	1	0	HC-NDD
CACNA1C	FALSE	FALSE	TRUE	TRUE	FALSE	NDD	1	0	0	0	HC-NDD
CACNA1G	FALSE	FALSE	TRUE	TRUE	FALSE	NDD	1	0	0	0	HC-NDD
CAMK2G	FALSE	FALSE	TRUE	TRUE	FALSE	NDD	2	0	1	0	Candidate-NDD
CAMTA1	FALSE	FALSE	TRUE	TRUE	FALSE	NDD	2	3	0	2	HC-NDD
CNOT1	FALSE	FALSE	TRUE	TRUE	FALSE	NDD	1	1	0	0	HC-NDD
COL4A1	FALSE	FALSE	TRUE	TRUE	FALSE	NDD	1	1	0	0	HC-NDD
CTNND1	FALSE	FALSE	TRUE	TRUE	FALSE	NDD	1	1	0	0	HC-NDD
CTR9	FALSE	FALSE	TRUE	TRUE	FALSE	NDD	1	1	1	1	Candidate-NDD
DHX30	FALSE	FALSE	TRUE	TRUE	FALSE	NDD	2	1	2	1	HC-NDD
DNM1L	FALSE	FALSE	TRUE	TRUE	FALSE	NDD	0	1	0	0	HC-NDD
ERF	FALSE	FALSE	TRUE	TRUE	FALSE	NDD	2	0	1	0	HC-NDD
FBN1	FALSE	FALSE	TRUE	TRUE	FALSE	NDD	2	1	2	0	Candidate-NDD
GLI3	FALSE	FALSE	TRUE	TRUE	FALSE	NDD	1	0	1	0	HC-NDD
HECW2	FALSE	FALSE	TRUE	TRUE	FALSE	NDD	3	0	2	0	HC-NDD
HIST1H4J	FALSE	FALSE	TRUE	TRUE	FALSE	NDD	1	0	0	0	Candidate-NDD
ITPR1	FALSE	FALSE	TRUE	TRUE	FALSE	NDD	1	1	1	0	HC-NDD
KANSL1	FALSE	FALSE	TRUE	TRUE	FALSE	NDD	1	0	0	0	HC-NDD
KAT6A	FALSE	FALSE	TRUE	TRUE	FALSE	NDD	1	0	0	0	HC-NDD
KAT6B	FALSE	FALSE	TRUE	TRUE	FALSE	NDD	0	1	0	0	HC-NDD
KBTBD7	FALSE	FALSE	TRUE	TRUE	FALSE	NDD	1	0	0	0	.
KIAA0100	FALSE	FALSE	TRUE	TRUE	FALSE	NDD	0	1	0	0	Candidate-NDD
KIF11	FALSE	FALSE	TRUE	TRUE	FALSE	NDD	1	1	1	1	HC-NDD
KIF1A	FALSE	FALSE	TRUE	TRUE	FALSE	NDD	2	0	0	0	HC-NDD
KIF2A	FALSE	FALSE	TRUE	TRUE	FALSE	NDD	0	1	0	0	HC-NDD
KMT2D	FALSE	FALSE	TRUE	TRUE	FALSE	NDD	2	2	1	0	HC-NDD
LONP1	FALSE	FALSE	TRUE	TRUE	FALSE	NDD	0	1	0	0	HC-NDD
MAP3K7	FALSE	FALSE	TRUE	TRUE	FALSE	NDD	1	0	0	0	HC-NDD
MORC2	FALSE	FALSE	TRUE	TRUE	FALSE	NDD	1	0	0	0	HC-NDD
MSI1	FALSE	FALSE	TRUE	TRUE	FALSE	NDD	1	1	1	1	Candidate-NDD
MTOR	FALSE	FALSE	TRUE	TRUE	FALSE	NDD	1	0	0	0	HC-NDD
MYCN	FALSE	FALSE	TRUE	TRUE	FALSE	NDD	0	1	0	1	HC-NDD
NALCN	FALSE	FALSE	TRUE	TRUE	FALSE	NDD	1	0	1	0	HC-NDD
NFIA	FALSE	FALSE	TRUE	TRUE	FALSE	NDD	1	0	1	0	HC-NDD
NFIB	FALSE	FALSE	TRUE	TRUE	FALSE	NDD	1	1	1	1	HC-NDD
NFIX	FALSE	FALSE	TRUE	TRUE	FALSE	NDD	1	0	1	0	HC-NDD
NLGN2	FALSE	FALSE	TRUE	TRUE	FALSE	NDD	4	0	1	0	HC-NDD
NOTCH1	FALSE	FALSE	TRUE	TRUE	FALSE	NDD	8	1	1	0	Candidate-NDD
NSD1	FALSE	FALSE	TRUE	TRUE	FALSE	NDD	1	0	1	0	HC-NDD
NTRK2	FALSE	FALSE	TRUE	TRUE	FALSE	NDD	0	1	0	0	HC-NDD
PAFAH1B1	FALSE	FALSE	TRUE	TRUE	FALSE	NDD	1	0	1	0	HC-NDD
PIK3R2	FALSE	FALSE	TRUE	TRUE	FALSE	NDD	1	1	0	1	HC-NDD
PPM1D	FALSE	FALSE	TRUE	TRUE	FALSE	NDD	2	0	0	0	HC-NDD
PRPF8	FALSE	FALSE	TRUE	TRUE	FALSE	NDD	1	0	1	0	Candidate-NDD
PSMC3	FALSE	FALSE	TRUE	TRUE	FALSE	NDD	1	0	1	0	Candidate-NDD
PTCH1	FALSE	FALSE	TRUE	TRUE	FALSE	NDD	4	1	1	0	HC-NDD
PTPN11	FALSE	FALSE	TRUE	TRUE	FALSE	NDD	1	0	0	0	HC-NDD
PUF60	FALSE	FALSE	TRUE	TRUE	FALSE	NDD	2	0	2	0	HC-NDD
RAB11B	FALSE	FALSE	TRUE	TRUE	FALSE	NDD	1	0	1	0	HC-NDD
RHOBTB2	FALSE	FALSE	TRUE	TRUE	FALSE	NDD	1	0	0	0	HC-NDD
RIT1	FALSE	FALSE	TRUE	TRUE	FALSE	NDD	1	0	0	0	HC-NDD
RYR2	FALSE	FALSE	TRUE	TRUE	FALSE	NDD	3	1	1	0	Candidate-NDD
SEMA6B	FALSE	FALSE	TRUE	TRUE	FALSE	NDD	2	1	2	1	HC-NDD
SETD1B	FALSE	FALSE	TRUE	TRUE	FALSE	NDD	5	2	1	0	HC-NDD
SETD2	FALSE	FALSE	TRUE	TRUE	FALSE	NDD	0	1	0	0	HC-NDD
SF3B2	FALSE	FALSE	TRUE	TRUE	FALSE	NDD	1	0	1	0	Candidate-NDD
SLC2A1	FALSE	FALSE	TRUE	TRUE	FALSE	NDD	1	0	0	0	HC-NDD
SMARCA4	FALSE	FALSE	TRUE	TRUE	FALSE	NDD	2	2	0	1	HC-NDD
SMARCE1	FALSE	FALSE	TRUE	TRUE	FALSE	NDD	0	1	0	0	HC-NDD
SOS1	FALSE	FALSE	TRUE	TRUE	FALSE	NDD	2	0	0	0	HC-NDD

SOX11	FALSE	FALSE	TRUE	TRUE	FALSE	NDD	1	0	0	0	HC-NDD
SOX2	FALSE	FALSE	TRUE	TRUE	FALSE	NDD	1	0	0	0	HC-NDD
SOX9	FALSE	FALSE	TRUE	TRUE	FALSE	NDD	1	1	0	0	Candidate-NDD
SPTAN1	FALSE	FALSE	TRUE	TRUE	FALSE	NDD	2	1	2	1	HC-NDD
TAB2	FALSE	FALSE	TRUE	TRUE	FALSE	NDD	0	1	0	0	HC-NDD
TNRC6B	FALSE	FALSE	TRUE	TRUE	FALSE	NDD	0	1	0	0	HC-NDD
TRPM3	FALSE	FALSE	TRUE	TRUE	FALSE	NDD	1	0	0	0	HC-NDD
TRRAP	FALSE	FALSE	TRUE	TRUE	FALSE	NDD	1	0	1	0	HC-NDD
UBTF	FALSE	FALSE	TRUE	TRUE	FALSE	NDD	1	1	1	1	HC-NDD
WDR26	FALSE	FALSE	TRUE	TRUE	FALSE	NDD	1	0	0	0	HC-NDD
WDR48	FALSE	FALSE	TRUE	TRUE	FALSE	NDD	0	1	0	0	Candidate-NDD
ZBTB10	FALSE	FALSE	TRUE	TRUE	FALSE	NDD	1	0	0	0	Candidate-NDD
ALDH18A1	FALSE	FALSE	FALSE	TRUE	FALSE	NDD	0	1	0	0	HC-NDD
ANK3	FALSE	FALSE	FALSE	TRUE	FALSE	NDD	1	1	0	0	HC-NDD
ARID5B	FALSE	FALSE	FALSE	TRUE	FALSE	NDD	1	0	0	0	Candidate-NDD
ATP1B1	FALSE	FALSE	FALSE	TRUE	FALSE	NDD	1	0	0	0	Candidate-NDD
ATP2B1	FALSE	FALSE	FALSE	TRUE	FALSE	NDD	0	1	0	1	Candidate-NDD
BRSK1	FALSE	FALSE	FALSE	TRUE	FALSE	NDD	1	0	0	0	Candidate-NDD
BRWD1	FALSE	FALSE	FALSE	TRUE	FALSE	NDD	1	0	0	0	Candidate-NDD
C3	FALSE	FALSE	FALSE	TRUE	FALSE	NDD	1	0	0	0	Candidate-NDD
CASKIN2	FALSE	FALSE	FALSE	TRUE	FALSE	NDD	1	0	0	0	Candidate-NDD
CDC42BPB	FALSE	FALSE	FALSE	TRUE	FALSE	NDD	3	0	1	0	HC-NDD
CLPTM1	FALSE	FALSE	FALSE	TRUE	FALSE	NDD	3	0	0	0	Candidate-NDD
CPD	FALSE	FALSE	FALSE	TRUE	FALSE	NDD	1	1	0	0	.
CSDE1	FALSE	FALSE	FALSE	TRUE	FALSE	NDD	1	0	0	0	HC-NDD
CUX2	FALSE	FALSE	FALSE	TRUE	FALSE	NDD	2	3	0	0	HC-NDD
DDB1	FALSE	FALSE	FALSE	TRUE	FALSE	NDD	2	2	2	2	HC-NDD
EIF4A2	FALSE	FALSE	FALSE	TRUE	FALSE	NDD	1	0	1	0	Candidate-NDD
ELAVL3	FALSE	FALSE	FALSE	TRUE	FALSE	NDD	1	0	0	0	HC-NDD
ERLIN1	FALSE	FALSE	FALSE	TRUE	FALSE	NDD	1	0	1	0	Candidate-NDD
FBN2	FALSE	FALSE	FALSE	TRUE	FALSE	NDD	0	1	0	0	Candidate-NDD
FRMD5	FALSE	FALSE	FALSE	TRUE	FALSE	NDD	1	2	0	0	Candidate-NDD
GAPVD1	FALSE	FALSE	FALSE	TRUE	FALSE	NDD	1	1	0	1	Candidate-NDD
HDLBP	FALSE	FALSE	FALSE	TRUE	FALSE	NDD	0	1	0	0	HC-NDD
HECTD1	FALSE	FALSE	FALSE	TRUE	FALSE	NDD	1	0	1	0	Candidate-NDD
HIC2	FALSE	FALSE	FALSE	TRUE	FALSE	NDD	1	0	1	0	Candidate-NDD
HNF4A	FALSE	FALSE	FALSE	TRUE	FALSE	NDD	1	0	0	0	Candidate-NDD
INHBA	FALSE	FALSE	FALSE	TRUE	FALSE	NDD	1	1	0	0	Candidate-NDD
JAG1	FALSE	FALSE	FALSE	TRUE	FALSE	NDD	1	1	0	0	Candidate-NDD
KBTBD6	FALSE	FALSE	FALSE	TRUE	FALSE	NDD	1	0	1	0	Candidate-NDD
KDM1A	FALSE	FALSE	FALSE	TRUE	FALSE	NDD	1	1	0	0	HC-NDD
KIAA1109	FALSE	FALSE	FALSE	TRUE	FALSE	NDD	1	0	0	0	HC-NDD
LARP1	FALSE	FALSE	FALSE	TRUE	FALSE	NDD	0	1	0	0	Candidate-NDD
LRFN3	FALSE	FALSE	FALSE	TRUE	FALSE	NDD	0	1	0	0	Candidate-NDD
MED24	FALSE	FALSE	FALSE	TRUE	FALSE	NDD	1	0	1	0	.
MSANTD2	FALSE	FALSE	FALSE	TRUE	FALSE	NDD	1	0	1	0	Candidate-NDD
MYH3	FALSE	FALSE	FALSE	TRUE	FALSE	NDD	2	0	0	0	Candidate-NDD
NPR1	FALSE	FALSE	FALSE	TRUE	FALSE	NDD	2	0	1	0	.
PAK1	FALSE	FALSE	FALSE	TRUE	FALSE	NDD	0	1	0	1	HC-NDD
PAX2	FALSE	FALSE	FALSE	TRUE	FALSE	NDD	1	0	1	0	Candidate-NDD
PDE10A	FALSE	FALSE	FALSE	TRUE	FALSE	NDD	1	0	0	0	Candidate-NDD
PRKCB	FALSE	FALSE	FALSE	TRUE	FALSE	NDD	3	0	3	0	Candidate-NDD
PUM2	FALSE	FALSE	FALSE	TRUE	FALSE	NDD	1	1	0	0	Candidate-NDD
RBBP4	FALSE	FALSE	FALSE	TRUE	FALSE	NDD	1	0	0	0	Candidate-NDD
SALL3	FALSE	FALSE	FALSE	TRUE	FALSE	NDD	3	2	0	0	.
SBNO2	FALSE	FALSE	FALSE	TRUE	FALSE	NDD	1	0	1	0	.
SCAF1	FALSE	FALSE	FALSE	TRUE	FALSE	NDD	2	1	1	0	Candidate-NDD
SCAPER	FALSE	FALSE	FALSE	TRUE	FALSE	NDD	1	0	1	0	HC-NDD
SCN3A	FALSE	FALSE	FALSE	TRUE	FALSE	NDD	2	1	2	1	HC-NDD
SCUBE3	FALSE	FALSE	FALSE	TRUE	FALSE	NDD	1	0	0	0	Candidate-NDD
SF1	FALSE	FALSE	FALSE	TRUE	FALSE	NDD	2	1	1	0	Candidate-NDD
SF3B3	FALSE	FALSE	FALSE	TRUE	FALSE	NDD	1	1	1	1	Candidate-NDD
SNRNP200	FALSE	FALSE	FALSE	TRUE	FALSE	NDD	4	1	2	0	Candidate-NDD
SOX4	FALSE	FALSE	FALSE	TRUE	FALSE	NDD	1	0	0	0	HC-NDD
STAT1	FALSE	FALSE	FALSE	TRUE	FALSE	NDD	1	0	1	0	Candidate-NDD
STX1B	FALSE	FALSE	FALSE	TRUE	FALSE	NDD	2	0	0	0	HC-NDD
SUPT6H	FALSE	FALSE	FALSE	TRUE	FALSE	NDD	1	1	1	1	Candidate-NDD
TAOK3	FALSE	FALSE	FALSE	TRUE	FALSE	NDD	2	1	0	0	Candidate-NDD
TBX3	FALSE	FALSE	FALSE	TRUE	FALSE	NDD	1	1	1	1	Candidate-NDD
TERF2IP	FALSE	FALSE	FALSE	TRUE	FALSE	NDD	0	1	0	0	Candidate-NDD
TNPO2	FALSE	FALSE	FALSE	TRUE	FALSE	NDD	1	0	1	0	HC-NDD
TNRC6A	FALSE	FALSE	FALSE	TRUE	FALSE	NDD	2	0	0	0	Candidate-NDD
ZBTB34	FALSE	FALSE	FALSE	TRUE	FALSE	NDD	1	0	0	0	Candidate-NDD
ZNF536	FALSE	FALSE	FALSE	TRUE	FALSE	NDD	1	3	0	1	Candidate-NDD
ABCC9	FALSE	FALSE	TRUE	TRUE	FALSE	NDD	0	0	0	0	HC-NDD
ACTL6B	FALSE	FALSE	TRUE	TRUE	FALSE	NDD	0	0	0	0	HC-NDD
AFF3	FALSE	FALSE	TRUE	TRUE	FALSE	NDD	0	0	0	0	Candidate-NDD
AGO1	FALSE	FALSE	TRUE	TRUE	FALSE	NDD	0	0	0	0	HC-NDD
ARF1	FALSE	FALSE	TRUE	TRUE	FALSE	NDD	0	0	0	0	HC-NDD
ARHGAP35	FALSE	FALSE	TRUE	TRUE	FALSE	NDD	0	0	0	0	Candidate-NDD
ARID1A	FALSE	FALSE	TRUE	TRUE	FALSE	NDD	0	0	0	0	HC-NDD
ARIH1	FALSE	FALSE	TRUE	TRUE	FALSE	NDD	0	0	0	0	Candidate-NDD
ASXL2	FALSE	FALSE	TRUE	TRUE	FALSE	NDD	0	0	0	0	HC-NDD
ATF2	FALSE	FALSE	TRUE	TRUE	FALSE	NDD	0	0	0	0	Candidate-NDD
ATP1A2	FALSE	FALSE	TRUE	TRUE	FALSE	NDD	0	0	0	0	HC-NDD
ATP6V0A1	FALSE	FALSE	TRUE	TRUE	FALSE	NDD	0	0	0	0	HC-NDD
ATP6V1A	FALSE	FALSE	TRUE	TRUE	FALSE	NDD	0	0	0	0	HC-NDD
BAI1	FALSE	FALSE	TRUE	TRUE	FALSE	NDD	0	0	0	0	Candidate-NDD
BAP1	FALSE	FALSE	TRUE	TRUE	FALSE	NDD	0	0	0	0	Candidate-NDD
BCL11B	FALSE	FALSE	TRUE	TRUE	FALSE	NDD	0	0	0	0	HC-NDD
BICD2	FALSE	FALSE	TRUE	TRUE	FALSE	NDD	0	0	0	0	Candidate-NDD
BRAF	FALSE	FALSE	TRUE	TRUE	FALSE	NDD	0	0	0	0	HC-NDD
BRD3	FALSE	FALSE	TRUE	TRUE	FALSE	NDD	0	0	0	0	Candidate-NDD
CACNA1A	FALSE	FALSE	TRUE	TRUE	FALSE	NDD	0	0	0	0	HC-NDD
CAMK2B	FALSE	FALSE	TRUE	TRUE	FALSE	NDD	0	0	0	0	HC-NDD
CBX5	FALSE	FALSE	TRUE	TRUE	FALSE	NDD	0	0	0	0	Candidate-NDD
CDC42	FALSE	FALSE	TRUE	TRUE	FALSE	NDD	0	0	0	0	HC-NDD
CDK8	FALSE	FALSE	TRUE	TRUE	FALSE	NDD	0	0	0	0	HC-NDD
CHAMP1	FALSE	FALSE	TRUE	TRUE	FALSE	NDD	0	0	0	0	HC-NDD
CHD4	FALSE	FALSE	TRUE	TRUE	FALSE	NDD	0	0	0	0	HC-NDD
CHD7	FALSE	FALSE	TRUE	TRUE	FALSE	NDD	0	0	0	0	HC-NDD

CIC	FALSE	FALSE	TRUE	TRUE	FALSE	NDD	0	0	0	0	HC-NDD
CLTC	FALSE	FALSE	TRUE	TRUE	FALSE	NDD	0	0	0	0	HC-NDD
COL2A1	FALSE	FALSE	TRUE	TRUE	FALSE	NDD	0	0	0	0	Candidate-NDD
COL4A3BP	FALSE	FALSE	TRUE	TRUE	FALSE	NDD	0	0	0	0	HC-NDD
CSNK2B	FALSE	FALSE	TRUE	TRUE	FALSE	NDD	0	0	0	0	HC-NDD
CTBP1	FALSE	FALSE	TRUE	TRUE	FALSE	NDD	0	0	0	0	HC-NDD
DDX23	FALSE	FALSE	TRUE	TRUE	FALSE	NDD	0	0	0	0	HC-NDD
DHDDS	FALSE	FALSE	TRUE	TRUE	FALSE	NDD	0	0	0	0	HC-NDD
DNM1	FALSE	FALSE	TRUE	TRUE	FALSE	NDD	0	0	0	0	HC-NDD
DPF2	FALSE	FALSE	TRUE	TRUE	FALSE	NDD	0	0	0	0	HC-NDD
EEF1A2	FALSE	FALSE	TRUE	TRUE	FALSE	NDD	0	0	0	0	HC-NDD
EIF5A	FALSE	FALSE	TRUE	TRUE	FALSE	NDD	0	0	0	0	HC-NDD
EP300	FALSE	FALSE	TRUE	TRUE	FALSE	NDD	0	0	0	0	HC-NDD
EXT1	FALSE	FALSE	TRUE	TRUE	FALSE	NDD	0	0	0	0	Candidate-NDD
FBXW7	FALSE	FALSE	TRUE	TRUE	FALSE	NDD	0	0	0	0	Candidate-NDD
FGFR1	FALSE	FALSE	TRUE	TRUE	FALSE	NDD	0	0	0	0	HC-NDD
FGFR2	FALSE	FALSE	TRUE	TRUE	FALSE	NDD	0	0	0	0	HC-NDD
FGFR3	FALSE	FALSE	TRUE	TRUE	FALSE	NDD	0	0	0	0	HC-NDD
FOXG1	FALSE	FALSE	TRUE	TRUE	FALSE	NDD	0	0	0	0	HC-NDD
FOXP2	FALSE	FALSE	TRUE	TRUE	FALSE	NDD	0	0	0	0	HC-NDD
GABRB2	FALSE	FALSE	TRUE	TRUE	FALSE	NDD	0	0	0	0	HC-NDD
GABRG2	FALSE	FALSE	TRUE	TRUE	FALSE	NDD	0	0	0	0	HC-NDD
GATA3	FALSE	FALSE	TRUE	TRUE	FALSE	NDD	0	0	0	0	Candidate-NDD
GATAD2B	FALSE	FALSE	TRUE	TRUE	FALSE	NDD	0	0	0	0	HC-NDD
GFAP	FALSE	FALSE	TRUE	TRUE	FALSE	NDD	0	0	0	0	HC-NDD
GLTSCR1	FALSE	FALSE	TRUE	TRUE	FALSE	NDD	0	0	0	0	HC-NDD
GLYR1	FALSE	FALSE	TRUE	TRUE	FALSE	NDD	0	0	0	0	Candidate-NDD
GNAO1	FALSE	FALSE	TRUE	TRUE	FALSE	NDD	0	0	0	0	HC-NDD
GNAS	FALSE	FALSE	TRUE	TRUE	FALSE	NDD	0	0	0	0	HC-NDD
GNB1	FALSE	FALSE	TRUE	TRUE	FALSE	NDD	0	0	0	0	HC-NDD
GNB2	FALSE	FALSE	TRUE	TRUE	FALSE	NDD	0	0	0	0	HC-NDD
GRIN1	FALSE	FALSE	TRUE	TRUE	FALSE	NDD	0	0	0	0	HC-NDD
GRIN2A	FALSE	FALSE	TRUE	TRUE	FALSE	NDD	0	0	0	0	HC-NDD
GSK3B	FALSE	FALSE	TRUE	TRUE	FALSE	NDD	0	0	0	0	Candidate-NDD
HDAC2	FALSE	FALSE	TRUE	TRUE	FALSE	NDD	0	0	0	0	Candidate-NDD
HIST1H1E	FALSE	FALSE	TRUE	TRUE	FALSE	NDD	0	0	0	0	HC-NDD
HIST1H2AC	FALSE	FALSE	TRUE	TRUE	FALSE	NDD	0	0	0	0	Candidate-NDD
HIST1H2AE	FALSE	FALSE	TRUE	TRUE	FALSE	NDD	0	0	0	0	.
HIVEP2	FALSE	FALSE	TRUE	TRUE	FALSE	NDD	0	0	0	0	HC-NDD
HK1	FALSE	FALSE	TRUE	TRUE	FALSE	NDD	0	0	0	0	HC-NDD
HNRNPK	FALSE	FALSE	TRUE	TRUE	FALSE	NDD	0	0	0	0	HC-NDD
HNRNPU	FALSE	FALSE	TRUE	TRUE	FALSE	NDD	0	0	0	0	HC-NDD
HRAS	FALSE	FALSE	TRUE	TRUE	FALSE	NDD	0	0	0	0	HC-NDD
KCNA2	FALSE	FALSE	TRUE	TRUE	FALSE	NDD	0	0	0	0	HC-NDD
KCND3	FALSE	FALSE	TRUE	TRUE	FALSE	NDD	0	0	0	0	Candidate-NDD
KCNH1	FALSE	FALSE	TRUE	TRUE	FALSE	NDD	0	0	0	0	HC-NDD
KCNQ2	FALSE	FALSE	TRUE	TRUE	FALSE	NDD	0	0	0	0	HC-NDD
KCNT1	FALSE	FALSE	TRUE	TRUE	FALSE	NDD	0	0	0	0	HC-NDD
KCNT2	FALSE	FALSE	TRUE	TRUE	FALSE	NDD	0	0	0	0	HC-NDD
KIDINS220	FALSE	FALSE	TRUE	TRUE	FALSE	NDD	0	0	0	0	HC-NDD
KLF7	FALSE	FALSE	TRUE	TRUE	FALSE	NDD	0	0	0	0	HC-NDD
KLHL20	FALSE	FALSE	TRUE	TRUE	FALSE	NDD	0	0	0	0	.
KMT2B	FALSE	FALSE	TRUE	TRUE	FALSE	NDD	0	0	0	0	HC-NDD
KMT2E	FALSE	FALSE	TRUE	TRUE	FALSE	NDD	0	0	0	0	HC-NDD
KRAS	FALSE	FALSE	TRUE	TRUE	FALSE	NDD	0	0	0	0	HC-NDD
LZTR1	FALSE	FALSE	TRUE	TRUE	FALSE	NDD	0	0	0	0	HC-NDD
MAGEL2	FALSE	FALSE	TRUE	TRUE	FALSE	NDD	0	0	0	0	HC-NDD
MAP2K1	FALSE	FALSE	TRUE	TRUE	FALSE	NDD	0	0	0	0	HC-NDD
MFN2	FALSE	FALSE	TRUE	TRUE	FALSE	NDD	0	0	0	0	Candidate-NDD
MN1	FALSE	FALSE	TRUE	TRUE	FALSE	NDD	0	0	0	0	HC-NDD
ODC1	FALSE	FALSE	TRUE	TRUE	FALSE	NDD	0	0	0	0	HC-NDD
OTX2	FALSE	FALSE	TRUE	TRUE	FALSE	NDD	0	0	0	0	HC-NDD
PACS2	FALSE	FALSE	TRUE	TRUE	FALSE	NDD	0	0	0	0	HC-NDD
PCBP2	FALSE	FALSE	TRUE	TRUE	FALSE	NDD	0	0	0	0	Candidate-NDD
PDE4D	FALSE	FALSE	TRUE	TRUE	FALSE	NDD	0	0	0	0	HC-NDD
PHIP	FALSE	FALSE	TRUE	TRUE	FALSE	NDD	0	0	0	0	HC-NDD
PHLDB1	FALSE	FALSE	TRUE	TRUE	FALSE	NDD	0	0	0	0	.
PIK3R1	FALSE	FALSE	TRUE	TRUE	FALSE	NDD	0	0	0	0	HC-NDD
PIP5K1C	FALSE	FALSE	TRUE	TRUE	FALSE	NDD	0	0	0	0	Candidate-NDD
POLR3B	FALSE	FALSE	TRUE	TRUE	FALSE	NDD	0	0	0	0	HC-NDD
POU3F3	FALSE	FALSE	TRUE	TRUE	FALSE	NDD	0	0	0	0	HC-NDD
PPP1CB	FALSE	FALSE	TRUE	TRUE	FALSE	NDD	0	0	0	0	HC-NDD
PPP2CA	FALSE	FALSE	TRUE	TRUE	FALSE	NDD	0	0	0	0	HC-NDD
PPP2R1A	FALSE	FALSE	TRUE	TRUE	FALSE	NDD	0	0	0	0	HC-NDD
PPP3CA	FALSE	FALSE	TRUE	TRUE	FALSE	NDD	0	0	0	0	HC-NDD
PRKAR1B	FALSE	FALSE	TRUE	TRUE	FALSE	NDD	0	0	0	0	HC-NDD
PSMC5	FALSE	FALSE	TRUE	TRUE	FALSE	NDD	0	0	0	0	Candidate-NDD
PTPRD	FALSE	FALSE	TRUE	TRUE	FALSE	NDD	0	0	0	0	Candidate-NDD
PURA	FALSE	FALSE	TRUE	TRUE	FALSE	NDD	0	0	0	0	HC-NDD
RAB14	FALSE	FALSE	TRUE	TRUE	FALSE	NDD	0	0	0	0	Candidate-NDD
RAC1	FALSE	FALSE	TRUE	TRUE	FALSE	NDD	0	0	0	0	HC-NDD
RALA	FALSE	FALSE	TRUE	TRUE	FALSE	NDD	0	0	0	0	HC-NDD
RFX7	FALSE	FALSE	TRUE	TRUE	FALSE	NDD	0	0	0	0	HC-NDD
RORA	FALSE	FALSE	TRUE	TRUE	FALSE	NDD	0	0	0	0	HC-NDD
SATB1	FALSE	FALSE	TRUE	TRUE	FALSE	NDD	0	0	0	0	HC-NDD
SCN8A	FALSE	FALSE	TRUE	TRUE	FALSE	NDD	0	0	0	0	HC-NDD
SET	FALSE	FALSE	TRUE	TRUE	FALSE	NDD	0	0	0	0	HC-NDD
SETBP1	FALSE	FALSE	TRUE	TRUE	FALSE	NDD	0	0	0	0	HC-NDD
SHOC2	FALSE	FALSE	TRUE	TRUE	FALSE	NDD	0	0	0	0	HC-NDD
SMAD4	FALSE	FALSE	TRUE	TRUE	FALSE	NDD	0	0	0	0	HC-NDD
SMAD6	FALSE	FALSE	TRUE	TRUE	FALSE	NDD	0	0	0	0	Candidate-NDD
SMARCB1	FALSE	FALSE	TRUE	TRUE	FALSE	NDD	0	0	0	0	HC-NDD
SMC3	FALSE	FALSE	TRUE	TRUE	FALSE	NDD	0	0	0	0	HC-NDD
SNAP25	FALSE	FALSE	TRUE	TRUE	FALSE	NDD	0	0	0	0	HC-NDD
SON	FALSE	FALSE	TRUE	TRUE	FALSE	NDD	0	0	0	0	HC-NDD
SOX10	FALSE	FALSE	TRUE	TRUE	FALSE	NDD	0	0	0	0	HC-NDD
SPOP	FALSE	FALSE	TRUE	TRUE	FALSE	NDD	0	0	0	0	HC-NDD
SPTBN1	FALSE	FALSE	TRUE	TRUE	FALSE	NDD	0	0	0	0	HC-NDD
SRCAP	FALSE	FALSE	TRUE	TRUE	FALSE	NDD	0	0	0	0	HC-NDD
SRP54	FALSE	FALSE	TRUE	TRUE	FALSE	NDD	0	0	0	0	HC-NDD
SRRM2	FALSE	FALSE	TRUE	TRUE	FALSE	NDD	0	0	0	0	Candidate-NDD

SRSF1	FALSE	FALSE	TRUE	TRUE	FALSE	NDD	0	0	0	0	Candidate-NDD
STAG1	FALSE	FALSE	TRUE	TRUE	FALSE	NDD	0	0	0	0	HC-NDD
TCF12	FALSE	FALSE	TRUE	TRUE	FALSE	NDD	0	0	0	0	HC-NDD
TCF3	FALSE	FALSE	TRUE	TRUE	FALSE	NDD	0	0	0	0	.
TFAP4	FALSE	FALSE	TRUE	TRUE	FALSE	NDD	0	0	0	0	Candidate-NDD
TOP2B	FALSE	FALSE	TRUE	TRUE	FALSE	NDD	0	0	0	0	Candidate-NDD
TRIM8	FALSE	FALSE	TRUE	TRUE	FALSE	NDD	0	0	0	0	HC-NDD
TRIO	FALSE	FALSE	TRUE	TRUE	FALSE	NDD	0	0	0	0	HC-NDD
U2AF2	FALSE	FALSE	TRUE	TRUE	FALSE	NDD	0	0	0	0	Candidate-NDD
UBE3A	FALSE	FALSE	TRUE	TRUE	FALSE	NDD	0	0	0	0	HC-NDD
UPF1	FALSE	FALSE	TRUE	TRUE	FALSE	NDD	0	0	0	0	Candidate-NDD
USP7	FALSE	FALSE	TRUE	TRUE	FALSE	NDD	0	0	0	0	HC-NDD
VAMP2	FALSE	FALSE	TRUE	TRUE	FALSE	NDD	0	0	0	0	HC-NDD
VCP	FALSE	FALSE	TRUE	TRUE	FALSE	NDD	0	0	0	0	Candidate-NDD
WHSC1	FALSE	FALSE	TRUE	TRUE	FALSE	NDD	0	0	0	0	HC-NDD
YY1	FALSE	FALSE	TRUE	TRUE	FALSE	NDD	0	0	0	0	HC-NDD
ZBTB18	FALSE	FALSE	TRUE	TRUE	FALSE	NDD	0	0	0	0	HC-NDD
ZBTB7A	FALSE	FALSE	TRUE	TRUE	FALSE	NDD	0	0	0	0	HC-NDD
ZEB2	FALSE	FALSE	TRUE	TRUE	FALSE	NDD	0	0	0	0	HC-NDD
ZFHX3	FALSE	FALSE	TRUE	TRUE	FALSE	NDD	0	0	0	0	Candidate-NDD
ZFHX4	FALSE	FALSE	TRUE	TRUE	FALSE	NDD	0	0	0	0	HC-NDD
ZMIZ1	FALSE	FALSE	TRUE	TRUE	FALSE	NDD	0	0	0	0	HC-NDD
ZNF148	FALSE	FALSE	TRUE	TRUE	FALSE	NDD	0	0	0	0	HC-NDD
ZNF865	FALSE	FALSE	TRUE	TRUE	FALSE	NDD	0	0	0	0	Candidate-NDD
ACTA2	FALSE	FALSE	FALSE	TRUE	FALSE	NDD	0	0	0	0	.
AKT3	FALSE	FALSE	FALSE	TRUE	FALSE	NDD	0	0	0	0	HC-NDD
AMH	FALSE	FALSE	FALSE	TRUE	FALSE	NDD	0	0	0	0	.
AMIGO1	FALSE	FALSE	FALSE	TRUE	FALSE	NDD	0	0	0	0	Candidate-NDD
ANKRD12	FALSE	FALSE	FALSE	TRUE	FALSE	NDD	0	0	0	0	Candidate-NDD
AP1G1	FALSE	FALSE	FALSE	TRUE	FALSE	NDD	0	0	0	0	HC-NDD
AP3D1	FALSE	FALSE	FALSE	TRUE	FALSE	NDD	0	0	0	0	Candidate-NDD
ARCN1	FALSE	FALSE	FALSE	TRUE	FALSE	NDD	0	0	0	0	HC-NDD
ARHGAP32	FALSE	FALSE	FALSE	TRUE	FALSE	NDD	0	0	0	0	HC-NDD
ATP6V1B2	FALSE	FALSE	FALSE	TRUE	FALSE	NDD	0	0	0	0	HC-NDD
B3GNT6	FALSE	FALSE	FALSE	TRUE	FALSE	NDD	0	0	0	0	.
BCAS3	FALSE	FALSE	FALSE	TRUE	FALSE	NDD	0	0	0	0	HC-NDD
BCL9	FALSE	FALSE	FALSE	TRUE	FALSE	NDD	0	0	0	0	.
BMPR1A	FALSE	FALSE	FALSE	TRUE	FALSE	NDD	0	0	0	0	.
BOD1L1	FALSE	FALSE	FALSE	TRUE	FALSE	NDD	0	0	0	0	Candidate-NDD
BPTF	FALSE	FALSE	FALSE	TRUE	FALSE	NDD	0	0	0	0	HC-NDD
BTBD10	FALSE	FALSE	FALSE	TRUE	FALSE	NDD	0	0	0	0	.
CACNA2D1	FALSE	FALSE	FALSE	TRUE	FALSE	NDD	0	0	0	0	Candidate-NDD
CALM1	FALSE	FALSE	FALSE	TRUE	FALSE	NDD	0	0	0	0	Candidate-NDD
CAMK2D	FALSE	FALSE	FALSE	TRUE	FALSE	NDD	0	0	0	0	.
CAMK4	FALSE	FALSE	FALSE	TRUE	FALSE	NDD	0	0	0	0	HC-NDD
CBL	FALSE	FALSE	FALSE	TRUE	FALSE	NDD	0	0	0	0	HC-NDD
CBLL1	FALSE	FALSE	FALSE	TRUE	FALSE	NDD	0	0	0	0	Candidate-NDD
CBX3	FALSE	FALSE	FALSE	TRUE	FALSE	NDD	0	0	0	0	Candidate-NDD
CDKN1C	FALSE	FALSE	FALSE	TRUE	FALSE	NDD	0	0	0	0	Candidate-NDD
CDKN2AIP	FALSE	FALSE	FALSE	TRUE	FALSE	NDD	0	0	0	0	Candidate-NDD
CENPE	FALSE	FALSE	FALSE	TRUE	FALSE	NDD	0	0	0	0	HC-NDD
CENPV	FALSE	FALSE	FALSE	TRUE	FALSE	NDD	0	0	0	0	.
CHD5	FALSE	FALSE	FALSE	TRUE	FALSE	NDD	0	0	0	0	HC-NDD
CHMP7	FALSE	FALSE	FALSE	TRUE	FALSE	NDD	0	0	0	0	.
CNOT2	FALSE	FALSE	FALSE	TRUE	FALSE	NDD	0	0	0	0	Candidate-NDD
CPSF6	FALSE	FALSE	FALSE	TRUE	FALSE	NDD	0	0	0	0	Candidate-NDD
CRIM1	FALSE	FALSE	FALSE	TRUE	FALSE	NDD	0	0	0	0	Candidate-NDD
DAZAP1	FALSE	FALSE	FALSE	TRUE	FALSE	NDD	0	0	0	0	Candidate-NDD
DCAF7	FALSE	FALSE	FALSE	TRUE	FALSE	NDD	0	0	0	0	Candidate-NDD
DCTN2	FALSE	FALSE	FALSE	TRUE	FALSE	NDD	0	0	0	0	Candidate-NDD
DDX6	FALSE	FALSE	FALSE	TRUE	FALSE	NDD	0	0	0	0	HC-NDD
DLK2	FALSE	FALSE	FALSE	TRUE	FALSE	NDD	0	0	0	0	.
DOPEY1	FALSE	FALSE	FALSE	TRUE	FALSE	NDD	0	0	0	0	Candidate-NDD
DOT1L	FALSE	FALSE	FALSE	TRUE	FALSE	NDD	0	0	0	0	Candidate-NDD
DPYSL5	FALSE	FALSE	FALSE	TRUE	FALSE	NDD	0	0	0	0	HC-NDD
DRD2	FALSE	FALSE	FALSE	TRUE	FALSE	NDD	0	0	0	0	Candidate-NDD
DUS1L	FALSE	FALSE	FALSE	TRUE	FALSE	NDD	0	0	0	0	.
DUSP16	FALSE	FALSE	FALSE	TRUE	FALSE	NDD	0	0	0	0	.
EIF3B	FALSE	FALSE	FALSE	TRUE	FALSE	NDD	0	0	0	0	Candidate-NDD
EIF3G	FALSE	FALSE	FALSE	TRUE	FALSE	NDD	0	0	0	0	HC-NDD
ELAVL4	FALSE	FALSE	FALSE	TRUE	FALSE	NDD	0	0	0	0	Candidate-NDD
EML6	FALSE	FALSE	FALSE	TRUE	FALSE	NDD	0	0	0	0	.
ESRRG	FALSE	FALSE	FALSE	TRUE	FALSE	NDD	0	0	0	0	.
ETF1	FALSE	FALSE	FALSE	TRUE	FALSE	NDD	0	0	0	0	Candidate-NDD
EZH2	FALSE	FALSE	FALSE	TRUE	FALSE	NDD	0	0	0	0	HC-NDD
FBXO28	FALSE	FALSE	FALSE	TRUE	FALSE	NDD	0	0	0	0	HC-NDD
FEZF2	FALSE	FALSE	FALSE	TRUE	FALSE	NDD	0	0	0	0	Candidate-NDD
FGF12	FALSE	FALSE	FALSE	TRUE	FALSE	NDD	0	0	0	0	HC-NDD
FOSL2	FALSE	FALSE	FALSE	TRUE	FALSE	NDD	0	0	0	0	Candidate-NDD
FUS	FALSE	FALSE	FALSE	TRUE	FALSE	NDD	0	0	0	0	Candidate-NDD
G3BP2	FALSE	FALSE	FALSE	TRUE	FALSE	NDD	0	0	0	0	Candidate-NDD
GABBR1	FALSE	FALSE	FALSE	TRUE	FALSE	NDD	0	0	0	0	Candidate-NDD
GAK	FALSE	FALSE	FALSE	TRUE	FALSE	NDD	0	0	0	0	Candidate-NDD
GGNBP2	FALSE	FALSE	FALSE	TRUE	FALSE	NDD	0	0	0	0	Candidate-NDD
GOLGA4	FALSE	FALSE	FALSE	TRUE	FALSE	NDD	0	0	0	0	.
GPR4	FALSE	FALSE	FALSE	TRUE	FALSE	NDD	0	0	0	0	.
GRIK2	FALSE	FALSE	FALSE	TRUE	FALSE	NDD	0	0	0	0	HC-NDD
GRN	FALSE	FALSE	FALSE	TRUE	FALSE	NDD	0	0	0	0	Candidate-NDD
HCN1	FALSE	FALSE	FALSE	TRUE	FALSE	NDD	0	0	0	0	HC-NDD
HNRNPA2B1	FALSE	FALSE	FALSE	TRUE	FALSE	NDD	0	0	0	0	Candidate-NDD
HSP90AB1	FALSE	FALSE	FALSE	TRUE	FALSE	NDD	0	0	0	0	Candidate-NDD
INF2	FALSE	FALSE	FALSE	TRUE	FALSE	NDD	0	0	0	0	Candidate-NDD
KAT5	FALSE	FALSE	FALSE	TRUE	FALSE	NDD	0	0	0	0	HC-NDD
KAT8	FALSE	FALSE	FALSE	TRUE	FALSE	NDD	0	0	0	0	HC-NDD
KCNA1	FALSE	FALSE	FALSE	TRUE	FALSE	NDD	0	0	0	0	HC-NDD
KCNC1	FALSE	FALSE	FALSE	TRUE	FALSE	NDD	0	0	0	0	HC-NDD
KCNJ6	FALSE	FALSE	FALSE	TRUE	FALSE	NDD	0	0	0	0	HC-NDD
KIAA1551	FALSE	FALSE	FALSE	TRUE	FALSE	NDD	0	0	0	0	.
KIF5B	FALSE	FALSE	FALSE	TRUE	FALSE	NDD	0	0	0	0	Candidate-NDD
KLHDC3	FALSE	FALSE	FALSE	TRUE	FALSE	NDD	0	0	0	0	Candidate-NDD
KLHL6	FALSE	FALSE	FALSE	TRUE	FALSE	NDD	0	0	0	0	.

LASP1	FALSE	FALSE	FALSE	TRUE	FALSE	NDD	0	0	0	0	Candidate-NDD
LEF1	FALSE	FALSE	FALSE	TRUE	FALSE	NDD	0	0	0	0	Candidate-NDD
LEO1	FALSE	FALSE	FALSE	TRUE	FALSE	NDD	0	0	0	0	HC-NDD
LHX2	FALSE	FALSE	FALSE	TRUE	FALSE	NDD	0	0	0	0	Candidate-NDD
LMNB1	FALSE	FALSE	FALSE	TRUE	FALSE	NDD	0	0	0	0	HC-NDD
LRRC4	FALSE	FALSE	FALSE	TRUE	FALSE	NDD	0	0	0	0	Candidate-NDD
MAEA	FALSE	FALSE	FALSE	TRUE	FALSE	NDD	0	0	0	0	.
MAFB	FALSE	FALSE	FALSE	TRUE	FALSE	NDD	0	0	0	0	Candidate-NDD
MAP2	FALSE	FALSE	FALSE	TRUE	FALSE	NDD	0	0	0	0	Candidate-NDD
MAP4K4	FALSE	FALSE	FALSE	TRUE	FALSE	NDD	0	0	0	0	Candidate-NDD
MAX	FALSE	FALSE	FALSE	TRUE	FALSE	NDD	0	0	0	0	.
MCOLN1	FALSE	FALSE	FALSE	TRUE	FALSE	NDD	0	0	0	0	HC-NDD
METTL2A	FALSE	FALSE	FALSE	TRUE	FALSE	NDD	0	0	0	0	.
MIP	FALSE	FALSE	FALSE	TRUE	FALSE	NDD	0	0	0	0	.
MKL1	FALSE	FALSE	FALSE	TRUE	FALSE	NDD	0	0	0	0	Candidate-NDD
MYRF	FALSE	FALSE	FALSE	TRUE	FALSE	NDD	0	0	0	0	Candidate-NDD
NACA	FALSE	FALSE	FALSE	TRUE	FALSE	NDD	0	0	0	0	Candidate-NDD
NAMPT	FALSE	FALSE	FALSE	TRUE	FALSE	NDD	0	0	0	0	Candidate-NDD
NARS	FALSE	FALSE	FALSE	TRUE	FALSE	NDD	0	0	0	0	HC-NDD
NCL	FALSE	FALSE	FALSE	TRUE	FALSE	NDD	0	0	0	0	Candidate-NDD
NEDD8	FALSE	FALSE	FALSE	TRUE	FALSE	NDD	0	0	0	0	Candidate-NDD
NEO1	FALSE	FALSE	FALSE	TRUE	FALSE	NDD	0	0	0	0	Candidate-NDD
NKX2-1	FALSE	FALSE	FALSE	TRUE	FALSE	NDD	0	0	0	0	HC-NDD
NOTCH2	FALSE	FALSE	FALSE	TRUE	FALSE	NDD	0	0	0	0	Candidate-NDD
NPTN	FALSE	FALSE	FALSE	TRUE	FALSE	NDD	0	0	0	0	Candidate-NDD
NR3C2	FALSE	FALSE	FALSE	TRUE	FALSE	NDD	0	0	0	0	HC-NDD
OTUD7A	FALSE	FALSE	FALSE	TRUE	FALSE	NDD	0	0	0	0	Candidate-NDD
PAPOLA	FALSE	FALSE	FALSE	TRUE	FALSE	NDD	0	0	0	0	Candidate-NDD
PBX3	FALSE	FALSE	FALSE	TRUE	FALSE	NDD	0	0	0	0	.
PCSK2	FALSE	FALSE	FALSE	TRUE	FALSE	NDD	0	0	0	0	Candidate-NDD
PISD	FALSE	FALSE	FALSE	TRUE	FALSE	NDD	0	0	0	0	HC-NDD
PLXNA2	FALSE	FALSE	FALSE	TRUE	FALSE	NDD	0	0	0	0	Candidate-NDD
PPARG	FALSE	FALSE	FALSE	TRUE	FALSE	NDD	0	0	0	0	.
PPFIA2	FALSE	FALSE	FALSE	TRUE	FALSE	NDD	0	0	0	0	Candidate-NDD
PRKAR1A	FALSE	FALSE	FALSE	TRUE	FALSE	NDD	0	0	0	0	Candidate-NDD
PRKCE	FALSE	FALSE	FALSE	TRUE	FALSE	NDD	0	0	0	0	Candidate-NDD
PRKD1	FALSE	FALSE	FALSE	TRUE	FALSE	NDD	0	0	0	0	HC-NDD
PRKG1	FALSE	FALSE	FALSE	TRUE	FALSE	NDD	0	0	0	0	Candidate-NDD
PROC	FALSE	FALSE	FALSE	TRUE	FALSE	NDD	0	0	0	0	.
PSMA3	FALSE	FALSE	FALSE	TRUE	FALSE	NDD	0	0	0	0	Candidate-NDD
RAB11A	FALSE	FALSE	FALSE	TRUE	FALSE	NDD	0	0	0	0	HC-NDD
RAB43	FALSE	FALSE	FALSE	TRUE	FALSE	NDD	0	0	0	0	Candidate-NDD
RAF1	FALSE	FALSE	FALSE	TRUE	FALSE	NDD	0	0	0	0	HC-NDD
RAP1B	FALSE	FALSE	FALSE	TRUE	FALSE	NDD	0	0	0	0	Candidate-NDD
RARA	FALSE	FALSE	FALSE	TRUE	FALSE	NDD	0	0	0	0	Candidate-NDD
RARB	FALSE	FALSE	FALSE	TRUE	FALSE	NDD	0	0	0	0	HC-NDD
RBM12	FALSE	FALSE	FALSE	TRUE	FALSE	NDD	0	0	0	0	.
RMND5A	FALSE	FALSE	FALSE	TRUE	FALSE	NDD	0	0	0	0	.
RPL10A	FALSE	FALSE	FALSE	TRUE	FALSE	NDD	0	0	0	0	Candidate-NDD
RPL19	FALSE	FALSE	FALSE	TRUE	FALSE	NDD	0	0	0	0	Candidate-NDD
RTN4RL2	FALSE	FALSE	FALSE	TRUE	FALSE	NDD	0	0	0	0	.
RUSC1	FALSE	FALSE	FALSE	TRUE	FALSE	NDD	0	0	0	0	Candidate-NDD
SCAF4	FALSE	FALSE	FALSE	TRUE	FALSE	NDD	0	0	0	0	HC-NDD
SCN4A	FALSE	FALSE	FALSE	TRUE	FALSE	NDD	0	0	0	0	Candidate-NDD
SEC16A	FALSE	FALSE	FALSE	TRUE	FALSE	NDD	0	0	0	0	Candidate-NDD
SF3B1	FALSE	FALSE	FALSE	TRUE	FALSE	NDD	0	0	0	0	Candidate-NDD
SF3B4	FALSE	FALSE	FALSE	TRUE	FALSE	NDD	0	0	0	0	Candidate-NDD
SLC15A3	FALSE	FALSE	FALSE	TRUE	FALSE	NDD	0	0	0	0	.
SLC39A8	FALSE	FALSE	FALSE	TRUE	FALSE	NDD	0	0	0	0	HC-NDD
SLC4A4	FALSE	FALSE	FALSE	TRUE	FALSE	NDD	0	0	0	0	HC-NDD
SOGA1	FALSE	FALSE	FALSE	TRUE	FALSE	NDD	0	0	0	0	Candidate-NDD
SOX6	FALSE	FALSE	FALSE	TRUE	FALSE	NDD	0	0	0	0	HC-NDD
SPI1	FALSE	FALSE	FALSE	TRUE	FALSE	NDD	0	0	0	0	Candidate-NDD
SPTBN2	FALSE	FALSE	FALSE	TRUE	FALSE	NDD	0	0	0	0	HC-NDD
SRRM3	FALSE	FALSE	FALSE	TRUE	FALSE	NDD	0	0	0	0	.
SRSF7	FALSE	FALSE	FALSE	TRUE	FALSE	NDD	0	0	0	0	Candidate-NDD
SUPT16H	FALSE	FALSE	FALSE	TRUE	FALSE	NDD	0	0	0	0	HC-NDD
SVEP1	FALSE	FALSE	FALSE	TRUE	FALSE	NDD	0	0	0	0	Candidate-NDD
TFAP2B	FALSE	FALSE	FALSE	TRUE	FALSE	NDD	0	0	0	0	Candidate-NDD
TMEM63B	FALSE	FALSE	FALSE	TRUE	FALSE	NDD	0	0	0	0	Candidate-NDD
TNFRSF11A	FALSE	FALSE	FALSE	TRUE	FALSE	NDD	0	0	0	0	.
TNFRSF21	FALSE	FALSE	FALSE	TRUE	FALSE	NDD	0	0	0	0	Candidate-NDD
TNNC2	FALSE	FALSE	FALSE	TRUE	FALSE	NDD	0	0	0	0	.
TSC2	FALSE	FALSE	FALSE	TRUE	FALSE	NDD	0	0	0	0	HC-NDD
UBA2	FALSE	FALSE	FALSE	TRUE	FALSE	NDD	0	0	0	0	HC-NDD
USP46	FALSE	FALSE	FALSE	TRUE	FALSE	NDD	0	0	0	0	Candidate-NDD
VPS4A	FALSE	FALSE	FALSE	TRUE	FALSE	NDD	0	0	0	0	HC-NDD
WAPAL	FALSE	FALSE	FALSE	TRUE	FALSE	NDD	0	0	0	0	Candidate-NDD
WDR37	FALSE	FALSE	FALSE	TRUE	FALSE	NDD	0	0	0	0	HC-NDD
ZBTB1	FALSE	FALSE	FALSE	TRUE	FALSE	NDD	0	0	0	0	Candidate-NDD
ZDBF2	FALSE	FALSE	FALSE	TRUE	FALSE	NDD	0	0	0	0	.
ZDHHC5	FALSE	FALSE	FALSE	TRUE	FALSE	NDD	0	0	0	0	Candidate-NDD
ZNF423	FALSE	FALSE	FALSE	TRUE	FALSE	NDD	0	0	0	0	Candidate-NDD
ZNF644	FALSE	FALSE	FALSE	TRUE	FALSE	NDD	0	0	0	0	Candidate-NDD

Supplementary Table 9: Comorbidities of probands with pdSNVs in the list of 684 high-confidence ASD/NDD genes

a) Rare pdSNVs

	***Rare de novo* pdSNVs in 684 ASD/NDD genes**				**Rare severe pdSNVs (*de novo* /inherited) in 684 ASD/NDD genes**			
	**Probands with *de novo* pdSNVs n =16 (11%)**	**Probands without *de novo* pdSNVs n =128 (89%)**	**OR**	**Fisher's Exact test p-value**	**Probands with severe pdSNVs n =49 (34%)**	**Probands without severe pdSNVs n =95 (66%)**	**OR**	**Fisher's Exact test p-value**
**Females**	4 (25%)	30 (23%)			12 (24%)	22 (23%)		
**SPX**	9 (56%)	80 (63%)			33 (67%)	56 (59%)		
**Severe ID**	5 (31%)	11 (9%)	4.83	0.018	10 (20%)	6 (6%)	3.80	0.022

b) Novel pdSNVs

	**Novel *de novo* pdSNVs in 684 ASD/NDD genes**				**Novel severe pdSNVs (*de novo* /inherited) in 684 ASD/NDD genes**			
	**Probands with *de novo* pdSNVs n =15 (10%)**	**Probands without *de novo* pdSNVs n =129 (90%)**	**OR**	**Fisher's Exact test p-value**	**Probands with severe pdSNVs n=43 (30%)**	**Probands without severe pdSNVs n=101 (70%)**	**OR**	**Fisher's Exact test p-value**
**Females**	4 (27%)	30 (23%)			12 (28%)	22 (22%)		
**SPX**	9 (60%)	80 (62%)			28 (65%)	61 (60%)		
**Severe ID**	5 (33%)	11 (9%)	5.36	0.014	9 (21%)	7 (7%)	3.55	0.021

De novo pdSNVs include high confidence PTVs in genes with LOEUF <0.6 (PTV_{LOEUF}), missense variants with MPC score ≥2 (DmisB) and missense variants with MPC score 1-2 (DmisA). Severe pdSNVs include high confidence PTVs in genes with LOEUF <0.6 (PTV_{LOEUF}) and missense variants with MPC score ≥2 (DmisB) likely pathogenic for AlphaMissense ($LP_{\alpha M}$). Severe ID: non verbal IQ<35

Supplementary Table 10: Rare genic CNVs in affected individuals and SLD siblings

CytoBand	Coordinates (hg38)	Length (bp)	CNV type	Available CNV data	Detected from SNP/GS data	Family	Proband	Segregation [1]	Inheritance	Genes (hg38.ncbiRefSeqCurated)	NDD genes (GeneTrek v2) [2]	pdCNVs (Table2)
1p36.32	chr1:3445692-3499148	53457	LOSS	SNP	SNP	Fam41	41.3	41: f/m/aM/uM	paternal	ARHGEF16,MEGF6	.	no
1p36.23	chr1:8917389-8978674	61286	GAIN	SNP/GS	SNP/GS	Fam10	10.3	10: f/m/aM/uF	maternal	CA6	CA6(cand)	no
1p36.13	chr1:18606250-19018007	411758	LOSS	SNP	SNP	Fam91	91.3	91: m/aM/dM/aM/dM	father N/A	ALDH4A1,IFFO2,MIR1290,MIR4695,PAX7,TAS1R2	ALDH4A1(HC),PAX7(cand)	yes
1p33	chr1:49251101-49466595	215495	LOSS	SNP/GS	SNP/GS	Fam78	78.3	78: f/m/aF/uM	paternal	AGBL4,AGBL4-AS1,AGBL4-IT1	AGBL4(cand)	no
1p33	chr1:49447941-49533260	85320	LOSS	SNP/GS	GS	Fam44	44.3	44: f/m/aM/uF	paternal	AGBL4,AGBL4-IT1	AGBL4(cand)	no
1p32.3	chr1:54657064-54667169	10106	LOSS	SNP/GS	GS	Fam48	48.3	48: f/m/aM/uM	maternal	MROH7-TTC4,MROH7	.	no
1p31.3	chr1:65126632-65158378	31747	GAIN	SNP/GS	GS	Fam107	107.3	107: f/m/aM	paternal	AK4	.	no
1p31.1	chr1:71519763-71888101	368339	LOSS	SNP/GS	SNP/GS	Fam108	108.3	108: f/m/aM	maternal	NEGR1,NEGR1-IT1	NEGR1(cand)	yes
1p21.1-p21.2	chr1:101559386-101806218	246833	GAIN	SNP	SNP	Fam115	115.4	115: f/m/aF/aM/aF	paternal	LINC01709,OLFM3	.	no
1p21.1	chr1:101825699-101875521	49823	LOSS	SNP/GS	SNP/GS	Fam75	75.3	75: f/m/aM	paternal	DNAJA1P5,OLFM3	.	no
1q24.1	chr1:167038425-167074452	36028	LOSS	SNP/GS	SNP/GS	Fam92	92.3	92: f/m/aM/raM	maternal	GPA33	.	no
1q24.2	chr1:170622490-170688738	66249	GAIN	SNP/GS	SNP/GS	Fam94	94.3	94: f/m/aM	maternal	PRRX1	PRRX1(cand)	no
1q32.1	chr1:200044020-200054031	10012	GAIN	SNP/GS	GS	Fam34	34.3	34: f/m/aM/uF	maternal	NR5A2	.	no
1q41	chr1:219913924-219941648	27725	GAIN	SNP/GS	SNP/GS	Fam12	12.3	12: f/m/aM/uM/aM	paternal	SLC30A10	SLC30A10(cand)	no
1q42.13	chr1:230095012-230188321	93310	GAIN	SNP	SNP	Fam2	2.3	2: f/m/aF	maternal	GALNT2	GALNT2(HC)	yes
1q43	chr1:239719407-240184673	465267	GAIN	SNP/GS	SNP/GS	Fam95	95.3	95: f/m/aM/aM/uF	maternal	CHRM3,FMN2,RPS7P5,CHRM3-AS1	CHRM3(cand),FMN2(HC)	no
1q44	chr1:245334797-245424055	89259	LOSS	SNP/GS	SNP/GS	Fam71	71.3	71: f/m/aF	maternal	KIF26B	KIF26B(cand)	yes
1q44	chr1:246965773-246989584	23812	LOSS	SNP/GS	SNP/GS	Fam51	51.3	51: f/m/aM/uF	paternal	ZNF670-ZNF695,ZNF695	.	no
2p25.3	chr2:196082-261610	65529	LOSS	SNP/GS	SNP/GS	Fam100	100.3	100: f/m/aF	paternal	SH3YL1	SH3YL1(cand)	yes
2p25.3	chr2:1736572-1846326	109755	GAIN	SNP/GS	GS	Fam84	84.3	84: f/m/aF	maternal	MYT1L,PXDN	MYT1L(HC),PXDN(cand)	yes
2p25.2	chr2:6833046-6845996	12951	LOSS	SNP/GS	GS	Fam113	113.4	113: f/m/aF/raM/uF	N/A	CMPK2,NRIR	CMPK2(cand)	yes
2p23.3	chr2:24969272-25033256	63985	GAIN	SNP/GS	GS	Fam17	17.3	17: f/m/aM/uM	paternal	DNAJC27-AS1,DNAJC27	.	no
2p21	chr2:44279660-44297873	18214	LOSS	SNP/GS	SNP/GS	Fam52	52.3	52: f/m/aM	paternal	SLC3A1	.	no
2p16.3	chr2:49962600-50756048	793449	LOSS	SNP	SNP	Fam2	2.3	2: f/m/aF	maternal	NRXN1,MIR8485	NRXN1(HC)	yes
2p16.2	chr2:54193480-54356405	162926	LOSS	SNP	SNP	Fam120	120.4	120: f/m/aM/**aM**	***de novo***	**ACYP2,C2orf73,TSPYL6**	**ACYP2(cand)**	yes
2p13.2	chr2:73156352-73212468	56117	GAIN	SNP/GS	SNP/GS	Fam51	51.3	51: f/m/aM/uF	maternal	NOTO	.	no
2p11.2	chr2:85382432-85420460	38029	LOSS	SNP/GS	SNP/GS	Fam113	113.3	113: f/m/aF/raM/uF	paternal	CAPG,ELMOD3,SH2D6	CAPG(cand)	no
2q13	chr2:110095476-110227154	131679	GAIN	SNP/GS	SNP/GS	Fam108	108.3	108: f/m/aM	maternal	MALL,MTLN,NPHP1	NPHP1(HC)	no
2q14.2	chr2:121327822-121451859	124038	GAIN	SNP/GS	GS	Fam101	101.3	101: f/m/aM	paternal	CLASP1	CLASP1(cand)	no
2q21.2	chr2:133265993-133292049	26057	LOSS	SNP/GS	GS	Fam7	7.4	7: f/m/aM/aM/uM	maternal	NCKAP5,NCKAP5-AS2	NCKAP5(cand)	no
2q37.2	chr2:236200550-236222477	21928	LOSS	SNP/GS	GS	Fam50	50.3	50: f/m/aF	paternal	ASB18	.	no
2q37.3	chr2:239926522-239970550	44029	GAIN	SNP/GS	GS	Fam107	107.3	107: f/m/aM	paternal	MIR4786,NDUFA10	NDUFA10(cand)	no
2q37.3	chr2:240684490-240770186	85697	GAIN	SNP/GS	GS	Fam51	51.3	51: f/m/aM/uF	maternal	AQP12A,KIF1A,LOC285191	KIF1A(HC)	no
2q37.3	chr2:240685836-240770462	84627	GAIN	SNP/GS	SNP/GS	Fam92	92.3	92: f/m/aM/raM	maternal	AQP12A,KIF1A,LOC285191	KIF1A(HC)	no
3p26.3	chr3:241763-276520	34758	LOSS	SNP/GS	SNP/GS	Fam9	9.4	9: f/m/aM/dM/uF	maternal	CHL1	CHL1(cand)	no
3p24.2	chr3:24849648-24866624	16977	LOSS	SNP/GS	GS	Fam29	29.3	29: f/m/aF/uM	paternal	RARB	RARB(HC)	no
3p22.1	chr3:40374287-40407387	33101	LOSS	SNP/GS	GS	Fam77	77.3	77: f/m/aM/aF/uM/uFs	maternal	ENTPD3,ENTPD3-AS1	ENTPD3(cand)	no
3q13.13	chr3:111537794-111552977	15184	LOSS	SNP/GS	SNP/GS	Fam56	56.3	56: f/m/aF/aM	maternal	CD96	CD96(cand)	no
3q13.32	chr3:119008223-119053775	45553	GAIN	SNP/GS	SNP/GS	Fam99	99.4	99: f/m/aM/aM	maternal	IGSF11	.	no
4p16.3	chr4:2984670-2996333	11664	LOSS	SNP/GS	SNP/GS	Fam14	14.3	14: f/m/aM/aM	maternal	GRK4	.	no
4p15.2	chr4:26884744-27056163	171420	GAIN	SNP/GS	SNP/GS	Fam82	82.3	82: f/m/aM/uF	paternal	STIM2	STIM2(cand)	no
4q28.2	chr4:128851635-129010147	158513	GAIN	SNP/GS	SNP/GS	Fam85	85.3	85: f/m/aM/aM	maternal	JADE1,SCLT1	JADE1(cand)	no
4q28.2	chr4:128852273-129006649	154377	GAIN	SNP	SNP	Fam8	8.3	8: f/m/aM/uF	paternal	JADE1,SCLT1	JADE1(cand)	no
4q31.3	chr4:150267053-150327676	60624	GAIN	SNP/GS	SNP/GS	Fam11	11.3	11: f/m/aM/uM	maternal	LRBA	LRBA(cand)	yes
4q32.3	chr4:168622846-168679424	56579	LOSS	SNP/GS	SNP/GS	Fam35	35.3	35: f/m/aF/uM	maternal	PALLD	PALLD(cand)	no
4q35.2	chr4:186408181-186610376	202196	GAIN	SNP/GS	SNP/GS	Fam1	1.3	1: f/m/aM	maternal	FAT1,F11-AS1,MTNR1A	FAT1(HC)	no
4q35.2	chr4:187715944-188681612	965669	LOSS	SNP/GS	SNP/GS	Fam70	70.3	70: f/m/aM	paternal	LINC01060,TRIML1,TRIML2,ZFP42	.	no
5p15.33	chr5:437001-462457	25457	GAIN	SNP/GS	GS	Fam64	64.3	64: f/m/aM	paternal	AHRR,EXOC3,EXOC3-AS1,PDCD6-AHRR	EXOC3(cand)	no
5p15.31	chr5:7238099-7468447	230349	GAIN	SNP	SNP	Fam91	91.3	91: m/aM/dM/aM/dM	maternal	ADCY2,LOC442132,LINC02123,LINC02142	ADCY2(cand)	no
5p15.31	chr5:7410483-7471380	60898	GAIN	SNP/GS	GS	Fam81	81.3	81: f/m/aM/uM	paternal	ADCY2	ADCY2(cand)	no
5p13.2	chr5:37453605-37629929	176325	LOSS	SNP/GS	SNP/GS	Fam61	61.3	61: f/m/aM	paternal	WDR70	.	no
5q11.2	chr5:56121218-56138759	17542	GAIN	SNP/GS	GS	Fam68	68.3	68: f/m/aF/uF/uM	paternal	ANKRD55	.	no
5q12.3	chr5:67060542-67176929	116388	GAIN	SNP/GS	SNP/GS	Fam23	23.3	23: f/m/aM/dF/uFs	paternal	MAST4	.	no

5q13.2	chr5:73633702-73844631	210930	GAIN	SNP/GS	SNP/GS	Fam23	23.3	23: f/m/aM/dF/uFs	paternal	ARHGEF28	.	no
5q14.1	chr5:79401623-79420576	18954	LOSS	SNP/GS	GS	Fam82	82.3	82: f/m/aM/uF	maternal	HOMER1	HOMER1(cand)	yes
5q21.2	chr5:103542040-103809577	267538	GAIN	SNP/GS	SNP/GS	Fam25	25.3	25: f/m/aM	maternal	NUDT12	.	no
5q21.3	chr5:108496181-109233820	737640	GAIN	SNP/GS	SNP/GS	Fam73	73.3	73: f/m/**aF**/uM	***de novo***	FER,LINC01023	.	yes
5q23.1	chr5:121953524-121970220	16697	GAIN	SNP/GS	GS	Fam52	52.3	52: f/m/aM	paternal	SRFBP1	.	no
5q31.2	chr5:138536201-138548992	12792	GAIN	SNP/GS	GS	Fam11	11.3	11: f/m/aM/uM	paternal	ETF1	ETF1(cand)	no
6p12.3	chr6:49462173-49480480	18308	GAIN	SNP/GS	GS	Fam56	56.4	56: f/m/aF/aM	paternal	MUT,CENPQ	MMUT(HC)	no
6q12	chr6:63563200-63698372	135173	LOSS	SNP	SNP	Fam117	117.3	117: f/m/aM/aF	maternal	PHF3,PTP4A1,LOC128125822	PHF3(HC),PTP4A1(cand)	yes
6q12	chr6:65218009-65333510	115502	LOSS	SNP/GS	SNP/GS	Fam105	105.3	105: f/m/aM	maternal	EYS,LOC441155	EYS(cand)	no
6q13	chr6:73746981-73766013	19033	GAIN	SNP/GS	GS	Fam19	19.3	19: f/m/aM	maternal	CD109	.	no
6q25.1	chr6:151534004-151591862	57859	LOSS	SNP/GS	SNP/GS	Fam86	86.3	86: f/m/aF	paternal	CCDC170	.	no
6q25.3	chr6:156870642-156885949	15308	LOSS	SNP/GS	SNP/GS	Fam56	56.3	56: f/m/aF/aM	maternal	ARID1B	ARID1B(HC)	yes
6q27	chr6:167104592-167114687	10096	LOSS	SNP/GS	GS	Fam28	28.3	28: f/m/aF/uF	paternal	CCR6	.	no
7p22.3	chr7:261054-472257	211204	GAIN	SNP/GS	GS	Fam53	53.3	53: f/m/aM	maternal	FOXL3,FOXL3-OT1,LOC442497,LOC116435278,LOC112267991	.	no
7p21.3	chr7:12351493-12562201	210709	GAIN	SNP/GS	SNP/GS	Fam86	86.3	86: f/m/aF	maternal	LOC102725191,VWDE	.	no
7q11.21	chr7:64690471-64937553	247083	LOSS	SNP	SNP	Fam41	41.3	41: f/m/aM/uM	paternal	ZNF107,ZNF138,ZNF273	.	no
7q11.22	chr7:72086040-72107051	21012	GAIN	SNP/GS	GS	Fam95	95.3	95: f/m/aM/aM/uF	maternal	CALN1	.	no
7q11.23	chr7:76046887-76062107	15221	GAIN	SNP/GS	GS	Fam22	22.3	22: f/m/aF/aF/uF	maternal	MDH2,STYXL1	MDH2(HC),STYXL1(cand)	no
7q22.3	chr7:107621000-107641898	20899	LOSS	SNP/GS	GS	Fam19	19.3	19: f/m/aM	paternal	BCAP29,DUS4L-BCAP29	.	no
7q31.1	chr7:112301121-112322776	21656	LOSS	SNP/GS	GS	Fam102	102.3	102: f/m/aM	paternal	ZNF277	.	no
7q31.2	chr7:117660201-117670913	10713	LOSS	SNP/GS	GS	Fam107	107.3	107: f/m/aM	maternal	CFTR	.	no
7q31.31	chr7:121174075-121269627	95553	LOSS	SNP/GS	SNP/GS	Fam79	79.3	79: f/m/aM/uM	maternal	CPED1	.	no
7q32.1	chr7:127719389-127968829	249441	LOSS	SNP/GS	SNP/GS	Fam109	109.3	109: f/m/aM	paternal	SND1	SND1(cand)	yes
7q32.1	chr7:128288062-128307786	19725	GAIN	SNP/GS	GS	Fam77	77.3	77: f/m/aM/aF/uM/uFs	paternal	RBM28	RBM28(HC)	no
7q36.3	chr7:157340148-157479781	139634	GAIN	SNP/GS	SNP/GS	Fam48	48.3	48: f/m/aM/uM	maternal	DNAJB6,LOC101927914	DNAJB6(cand)	no
8p23.3	chr8:460393-1203174	742782	GAIN	SNP/GS	SNP/GS	Fam106	106.3	106: f/m/aM	paternal	DLGAP2,ERICH1,FBXO25,LOC401442,LOC105377777,TDRP	DLGAP2(cand)	no
8p22	chr8:17471896-17509420	37525	LOSS	SNP/GS	GS	Fam57	57.3	57: f/m/aM/uF	paternal	SLC7A2	.	no
8q11.22	chr8:51586310-51596780	10471	LOSS	SNP/GS	GS	Fam67	67.3	67: f/m/aM/uF	maternal	PXDNL	.	no
8q13.3	chr8:70685463-70807482	122020	LOSS	SNP/GS	SNP/GS	Fam15	15.3	15: f/m/aM/aM	maternal	XKR9	.	no
8q21.13	chr8:76734172-76944750	210579	GAIN	SNP/GS	SNP/GS	Fam24	24.4	24: f/m/aM/aM	paternal	ZFHX4	ZFHX4(HC)	no
8q22.1	chr8:96235252-96246875	11624	LOSS	SNP/GS	GS	Fam87	87.3	87: f/m/aM/uM	paternal	MTERF3,UQCRB,UQCRB-AS1	UQCRB(cand)	no
8q22.3	chr8:104392687-104406426	13740	LOSS	SNP/GS	SNP/GS	Fam85	85.3	85: f/m/aM/aM	maternal	DPYS	DPYS(cand)	yes
8q24.3	chr8:144793746-144807498	13753	LOSS	SNP/GS	GS	Fam103	103.3	103: f/m/aM	maternal	ZNF517	ZNF517(cand)	no
8q24.3	chr8:144920754-144932476	11723	LOSS	SNP	SNP	Fam115	115.3	115: f/m/aF/aM/aF	maternal	ZNF16	.	no
9p24.2	chr9:2726669-2792829	66161	LOSS	SNP/GS	SNP/GS	Fam14	14.3	14: f/m/aM/aM	maternal	KCNV2	KCNV2(cand)	yes
9p24.2	chr9:3457204-3524371	67168	GAIN	SNP/GS	GS	Fam67	67.3	67: f/m/aM/uF	maternal	RFX3	RFX3(HC)	no
9p24.1	chr9:5379481-5787518	408038	LOSS	SNP/GS	SNP/GS	Fam114	114.3	114: f/m/aM	paternal	RIC1,ERMP1,PLGRKT,CD274,PDCD1LG2	RIC1(HC)	no
9p22.1	chr9:19372707-19422113	49407	GAIN	SNP/GS	GS	Fam47	47.3	47: f/m/aM	paternal	ACER2,DENND4C,RPS6	RPS6(cand)	no
9p21.1	chr9:28457113-28476232	19120	GAIN	SNP/GS	GS	Fam82	82.3	82: f/m/aM/uF	paternal	LINGO2	LINGO2(cand)	no
9p21.1-p13.3	chr9:33140780-33262543	121764	GAIN	SNP/GS	SNP/GS	Fam46	46.3	46: f/m/aM/uM	paternal	BAG1,B4GALT1,B4GALT1-AS1,SPINK4	BAG1(cand),B4GALT1(HC)	yes
9p13.3	chr9:34296619-34311569	14951	LOSS	SNP/GS	SNP/GS	Fam13	13.3	13: f/m/aM	maternal	KIF24	.	no
9q21.11	chr9:68415483-68758083	342601	GAIN	SNP/GS	SNP/GS	Fam65	65.3	65: f/m/aM/uF/uF	paternal	PIP5K1B,PGM5,TMEM252,LINC01506	.	no
9q22.32	chr9:95221201-95255683	34483	LOSS	SNP/GS	SNP/GS	Fam96	96.3	96: f/m/aM/uF	maternal	FANCC	FANCC(cand)	yes
9q31.1	chr9:102974937-102996386	21450	GAIN	SNP/GS	GS	Fam15	15.3	15: f/m/aM/aM	paternal	CYLC2	CYLC2(cand)	no
9q31.3	chr9:108926659-108990573	63915	GAIN	SNP/GS	SNP/GS	Fam56	56.3	56: f/m/aF/aM	paternal	ABITRAM,CTNNAL1,ELP1	ELP1(cand)	no
9q33.3	chr9:125219881-125231633	11753	LOSS	SNP/GS	GS	Fam60	60.3	60: f/m/aF	maternal	RABEPK	RABEPK(cand)	no
9q33.3	chr9:127380921-127434723	53803	GAIN	SNP/GS	SNP/GS	Fam42	42.3	42: f/m/aM/uF	paternal	GARNL3,SLC2A8,ZNF79	GARNL3(cand)	no
10p15.2	chr10:3040783-3121006	80224	LOSS	SNP/GS	SNP/GS	Fam17	17.3	17: f/m/aM/uM	maternal	PFKP,PFKP-DT	PFKP(cand)	yes
10p11.22	chr10:32788364-32815841	27478	LOSS	SNP/GS	GS	Fam56	56.3	56: f/m/aF/aM	maternal	CCDC7	.	no
10p11.21	chr10:34838959-35069381	230423	GAIN	SNP/GS	SNP/GS	Fam11	11.3	11: f/m/aM/uM	paternal	CUL2	CUL2(cand)	no
10q23.1	chr10:80563654-80575364	11711	LOSS	SNP/GS	GS	Fam28	28.3	28: f/m/aF/uF	paternal	SH2D4B	.	no
10q23.31	chr10:90706464-90845238	138775	GAIN	SNP/GS	SNP/GS	Fam74	74.3	74: f/m/aM/uM	maternal	HTR7	HTR7(cand)	no
10q24.1	chr10:95440563-95689410	248848	GAIN	SNP/GS	SNP/GS	Fam47	47.3	47: f/m/aM	maternal	ALDH18A1,SORBS1,TCTN3	ALDH18A1(HC),SORBS1(cand), TCTN3(HC)	yes
10q24.32	chr10:102640823-102666335	25513	GAIN	SNP/GS	GS	Fam67	67.3	67: f/m/aM/uF	maternal	TRIM8	TRIM8(HC)	yes

10q25.2-q25.3	chr10:113078246-113111305	33060	LOSS	SNP/GS	GS	Fam47	47.3	47: f/m/aM	maternal	TCF7L2	TCF7L2(HC)	yes
10q26.3	chr10:133323788-133366683	42896	GAIN	SNP/GS	SNP/GS	Fam89	89.3	89: f/m/aM	maternal	CALY,ECHS1,PRAP1,FUOM	CALY(cand),ECHS1(HC)	no
11q12.1	chr11:58778082-58909459	131378	GAIN	SNP/GS	GS	Fam84	84.3	84: f/m/aF	paternal	GLYATL1,GLYATL2	.	no
11q24.2	chr11:125024122-125040200	16079	GAIN	SNP/GS	GS	Fam113	113.4	113: f/m/aF/raM/uF	N/A	CCDC15	.	no
12p13.33	chr12:662992-688533	25542	GAIN	SNP/GS	SNP/GS	Fam31	31.3	31: f/m/aM/uF	paternal	NINJ2	NINJ2(cand)	no
12p12.2	chr12:20863827-20884104	20278	LOSS	SNP/GS	SNP/GS	Fam108	108.3	108: f/m/aM	paternal	SLCO1B3,SLCO1B3-SLCO1B7	SLCO1B3(cand)	no
12p12.1	chr12:21412398-21434012	21615	LOSS	SNP/GS	GS	Fam26	26.3	26: f/m/aM/uM	maternal	SLCO1A2	SLCO1A2(cand)	no
12q13.13	chr12:52692556-52744846	52291	GAIN	SNP	SNP	Fam118	118.4	118: f/m/aM/aF/uM	paternal	KRT77	.	no
12q13.13	chr12:52755682-52770967	15286	LOSS	SNP/GS	SNP/GS	Fam56	56.3	56: f/m/aF/aM	maternal	KRT76	.	no
12q13.3	chr12:56941756-56981828	40073	GAIN	SNP/GS	GS	Fam16	16.3	16: f/m/aM/aM	paternal	RDH16	.	no
12q21.1	chr12:75214941-75283497	68557	GAIN	SNP/GS	GS	Fam92	92.3	92: f/m/aM/raM	paternal	CAPS2,LOC100130268	CAPS2(cand)	no
12q24.13	chr12:112304842-112441663	136822	GAIN	SNP/GS	GS	Fam22	22.3	22: f/m/aF/aF/uF	maternal	HECTD4,PTPN11,RPL6	HECTD4(HC),PTPN11(HC),RPL6 (cand)	no
12q24.31-q24.23	chr12:120295260-120318770	23511	LOSS	SNP/GS	GS	Fam77	77.3	77: f/m/aM/aF/uM/uFs	maternal	SIRT4	.	no
12q24.31	chr12:122261800-122275427	13628	GAIN	SNP/GS	GS	Fam12	12.3	12: f/m/aM/uM/aM	maternal	CLIP1,VPS33A	CLIP1(cand)	no
13q12.12	chr13:24621058-24764295	143238	GAIN	SNP	SNP	Fam93	93.3	93: f/m/aM/uM	paternal	ATP12A,RNF17	RNF17(cand)	no
13q12.12	chr13:24640872-24736612	95741	GAIN	SNP/GS	SNP/GS	Fam29	29.3	29: f/m/aF/uM	maternal	ATP12A	.	no
13q21.32	chr13:66774853-66860306	85454	LOSS	SNP/GS	SNP/GS	Fam97	97.3	97: f/m/aM/aF	maternal	PCDH9,PCDH9-AS2	PCDH9(cand)	no
13q31.3	chr13:93533137-93587893	54757	GAIN	SNP/GS	SNP/GS	Fam94	94.3	94: f/m/aM	paternal	GPC6	GPC6(cand)	yes
13q34	chr13:113639887-113736598	96712	GAIN	SNP/GS	GS	Fam105	105.3	105: f/m/aM	maternal	ATP4B,GRK1,TFDP1	TFDP1(cand)	no
13q34	chr13:113808856-113835183	26328	GAIN	SNP/GS	GS	Fam71	71.3	71: f/m/aF	maternal	TMEM255B,GAS6,GAS6-AS1	.	no
14q11.2	chr14:20498474-20517970	19497	GAIN	SNP/GS	GS	Fam95	95.4	95: f/m/aM/aM/uF	maternal	RNASE10	.	no
14q23.2	chr14:63305126-63398877	93752	GAIN	SNP/GS	GS	Fam29	29.3	29: f/m/aF/uM	maternal	PPP2R5E,GPHB5	PPP2R5E(cand)	no
14q32.32	chr14:102798110-102852223	54114	GAIN	SNP/GS	GS	Fam43	43.3	43: f/m/aM	paternal	TRAF3	TRAF3(cand)	no
14q32.33	chr14:103979304-104040169	60866	GAIN	SNP/GS	GS	Fam28	28.3	28: f/m/aF/uF	maternal	TDRD9	.	no
15q11.2	chr15:22636123-23102073	465951	GAIN	SNP/GS	SNP/GS	Fam99	99.4	99: f/m/aM/aM	paternal	CYFIP1,LOC283683,NIPA1,NIPA2,TUBGCP5,WHAMMP3	CYFIP1(cand),NIPA1(cand),NIPA2(cand),TUBGCP5(cand)	yes
15q11.2	chr15:22636123-23117882	481760	LOSS	SNP	SNP	Fam117	117.3	117: f/m/aM/aF	paternal	CYFIP1,LOC283683,NIPA1,NIPA2,TUBGCP5,WHAMMP3	CYFIP1(cand),NIPA1(cand),NIPA2(cand),TUBGCP5(cand)	yes
15q13.2-q13.3	chr15:30626840-32222140	1595301	LOSS	SNP	SNP	Fam115	115.3	115: f/m/aF/aM/aF	paternal	ARHGAP11B,CHRNA7,FAN1,HERC2P10,KLF13,LINC02352,LINC03034,LOC100288637,MIR211,MTMR10,OTUD7A,TRPM1	ARHGAP11B(cand),CHRNA7(cand),FAN1(cand),OTUD7A(cand),TRPM1(cand)	yes
15q13.2-q13.3	chr15:30635159-32222140	1586982	GAIN	SNP/GS	SNP/GS	Fam81	81.3	81: f/m/aM/uM	maternal	ARHGAP11B,CHRNA7,FAN1,HERC2P10,KLF13,LINC02352,LINC03034,LOC100288637,MIR211,MTMR10,OTUD7A,TRPM1	ARHGAP11B(cand),CHRNA7(cand),FAN1(cand),OTUD7A(cand),TRPM1(cand)	yes
15q15.1	chr15:41919964-41930311	10348	LOSS	SNP/GS	GS	Fam68	68.3	68: f/m/aF/uF/uM	paternal	EHD4,EHD4-AS1	.	no
15q22.31	chr15:64083030-64104271	21242	GAIN	SNP/GS	GS	Fam19	19.3	19: f/m/aM	maternal	SNX1,CIAO2A	SNX1(cand)	no
15q25.2	chr15:82742884-82782598	39715	GAIN	SNP/GS	SNP/GS	Fam22	22.3	22: f/m/aF/aF/uF	maternal	FSD2,SCARNA15,SNHG21	.	no
15q26.1	chr15:89676153-89694595	18443	LOSS	SNP/GS	SNP/GS	Fam52	52.3	52: f/m/aM	paternal	PEX11A,PLIN1,WDR93	PLIN1(cand),WDR93(cand)	no
15q26.3	chr15:101467741-101859739	391999	GAIN	SNP/GS	SNP/GS	Fam66	66.3	66: f/m/aM	paternal	LINC02348,OR4F13P,OR4F15,OR4F6,PCSK6,TARS3,TM2D3,UBE2Q2P13	TM2D3(cand)	no
15q26.3	chr15:101486854-101610964	124111	GAIN	SNP/GS	SNP/GS	Fam90	90.3	90: f/m/aF/dM	maternal	LINC02348,PCSK6	.	no
16p13.3	chr16:3053034-3071915	18882	LOSS	SNP/GS	GS	Fam94	94.3	94: f/m/aM	maternal	MMP25-AS1,MMP25,IL32	.	no
16p13.3	chr16:3055942-3075375	19434	LOSS	SNP/GS	GS	Fam28	28.3	28: f/m/aF/uF	paternal	MMP25-AS1,MMP25,IL32	.	no
16p13.2	chr16:8446703-8794331	347629	LOSS	SNP/GS	SNP/GS	Fam105	105.3	105: f/m/aM	paternal	ABAT,TMEM114,METTL22	ABAT(HC),TMEM114(cand)	yes
16p11.2	chr16:29584162-30188392	604231	LOSS	SNP	SNP	Fam118	118.4	118: f/m/aM/**aF**/uM	***de novo***	**ALDOA,ASPHD1,C16orf54,C16orf92,CDIPT,CDIPTOSP,CORO1A,DOC2A,GDPD3,HIRIP3,INO80E,KCTD13,KIF22,LOC112694756,MAPK3,MAZ,MIR3680-2,MVP,PAGR1,PPP4C,PRRT2,QPRT,SEZ6L2,SMG1P2,SPN,TAOK2,TBX6,TLCD3B,TMEM219,YPEL3,YPEL3-DT,ZG16**	**ALDOA(cand),ASPHD1(cand),CDIPT(cand),CORO1A(HC),KCTD13(cand),KIF22(cand),MAPK3(cand),MAZ(cand),PRRT2(HC),QPRT(cand),SEZ6L2(cand),TAOK2(cand),TBX6(cand)**	yes
16p11.2	chr16:30409802-30523250	113449	GAIN	SNP/GS	SNP/GS	Fam87	87.3	87: f/m/aM/uM	paternal	DCTPP1,ITGAL,MIR4518,SEPHS2,SNORA80C,ZNF771	.	no
16q23.1	chr16:78256339-78314555	58217	LOSS	SNP/GS	SNP/GS	Fam45	45.3	45: f/m/aM/uF	paternal	WWOX	WWOX(HC)	yes
16q23.2-q23.3	chr16:81527671-82084386	556716	GAIN	SNP/GS	SNP/GS	Fam107	107.3	107: f/m/aM	maternal	CMIP,HSD17B2,LOC100129617,MIR6504,MIR7854,PLCG2,SDR42E1	CMIP(cand),PLCG2(cand)	no
16q24.1	chr16:84238533-84314440	75908	GAIN	SNP/GS	SNP/GS	Fam22	22.3	22: f/m/aF/aF/uF	paternal	KCNG4,WFDC1	WFDC1(cand)	no
17p13.3	chr17:238137-310625	72489	GAIN	SNP/GS	SNP/GS	Fam101	101.3	101: f/m/aM	maternal	RPH3AL	.	no

17p13.3	chr17:1065582-1342604	277023	GAIN	SNP/GS	SNP/GS	Fam12	12.5	12: f/m/aM/uM/aM	maternal	ABR,BHLHA9,TRARG1	ABR(cand),BHLHA9(cand)	no
17p13.3	chr17:1923825-1948136	24312	GAIN	SNP/GS	GS	Fam44	44.3	44: f/m/aM/uF	paternal	RTN4RL1	RTN4RL1(cand)	no
17p13.1	chr17:9904545-9997527	92983	GAIN	SNP/GS	GS	Fam110	110.4	110: f/m/aM/aM/uMs	maternal	GAS7,RCVRN	GAS7(cand)	no
17p11.2	chr17:18904539-18931127	26589	GAIN	SNP/GS	GS	Fam12	12.3	12: f/m/aM/uM/aM	maternal	PRPSAP2	.	no
17q21.31	chr17:43324355-43702997	378643	GAIN	SNP/GS	SNP/GS	Fam94	94.3	94: f/m/aM	maternal	ARL4,DDHX8,ETV4,LINC00910,MEOX1,MIR2117,MIR2117HG	MEOX1(cand)	no
17q21.31	chr17:45882068-45980967	98900	GAIN	SNP/GS	SNP/GS	Fam39	39.3	39: f/m/aM	paternal	MAPT,MAPT-AS1,MAPT-IT1	MAPT(cand)	no
17q23.3	chr17:63872557-63906241	33685	LOSS	SNP	SNP	Fam121	121.4	121: f/m/aF/aM	maternal	CSH1,CSH2,GH2	.	no
17q25.1	chr17:75895283-75908604	13322	LOSS	SNP/GS	GS	Fam25	25.3	25: f/m/aM	paternal	MRPL38,TRIM65	.	no
17q25.3	chr17:81464264-81510148	45885	GAIN	SNP/GS	SNP/GS	Fam106	106.3	106: f/m/aM	maternal	ACTG1,BAHCC1,LINC01971	ACTG1(HC)	no
18p11.23	chr18:7200444-7737735	537292	GAIN	SNP/GS	SNP/GS	Fam25	25.3	25: f/m/aM	maternal	LRRC30,LOC112577592,PTPRM	PTPRM(cand)	no
18q12.2	chr18:36897900-36960200	62301	LOSS	SNP/GS	GS	Fam85	85.4	85: f/m/aM/aM	paternal	LOC105372069,KIAA1328	.	no
18q21.1	chr18:46643136-46684359	41224	GAIN	SNP/GS	GS	Fam96	96.3	96: f/m/aM/uF	maternal	ST8SIA5,LOXHD1	.	no
19p13.3	chr19:364427-384382	19956	LOSS	SNP/GS	SNP/GS	Fam43	43.3	43: f/m/aM	paternal	THEG	.	no
19p13.3	chr19:747510-779577	32068	GAIN	SNP/GS	GS	Fam110	110.3	110: f/m/aM/aM/uMs	paternal	PALM,MISP	PALM(cand)	no
19p13.3	chr19:2892921-2948514	55594	LOSS	SNP/GS	SNP/GS	Fam32	32.3	32: f/m/aM/uM	paternal	ZNF57,ZNF77	.	no
19p13.3+169:195	chr19:3804703-3831783	27081	GAIN	SNP	SNP	Fam115	115.4	115: f/m/aF/aM/aF	maternal	ZFR2	.	no
19p13.2	chr19:7250375-7305938	55564	GAIN	SNP/GS	GS	Fam49	49.3	49: f/m/aM	paternal	INSR	INSR(cand)	no
19p13.2	chr19:8257238-8270817	13580	GAIN	SNP/GS	GS	Fam7	7.4	7: f/m/aM/aM/uM	paternal	CERS4	CERS4(cand)	no
19q13.2	chr19:38670314-38770582	100269	GAIN	SNP/GS	SNP/GS	Fam13	13.3	13: f/m/aM	paternal	ACTN4,CAPN12	ACTN4(cand),CAPN12(cand)	no
19q13.32	chr19:44912872-44927662	14791	LOSS	SNP/GS	GS	Fam110	110.3	110: f/m/aM/aM/uMs	paternal	APOC1P1,APOC1	.	no
19q13.33	chr19:50449126-50484568	35443	LOSS	SNP/GS	GS	Fam7	7.4	7: f/m/aM/aM/uM	paternal	EMC10,GARIN5A,MYBPC2	EMC10(HC)	no
19q13.42	chr19:53892638-53932412	39775	GAIN	SNP/GS	GS	Fam55	55.4	55: f/m/aM/aM/dM	paternal	CACNG7,PRKCG	CACNG7(cand),PRKCG(HC)	no
19q13.43	chr19:56650622-56670856	20235	GAIN	SNP/GS	SNP/GS	Fam36	36.3	36: f/m/aM/uM	maternal	SMIM17,ZNF835,ZNF71-SMIM17	.	no
20q11.21	chr20:33154027-33178869	24843	GAIN	SNP/GS	GS	Fam22	22.3	22: f/m/aF/aF/uF	paternal	BPIFA2	.	no
20q13.12	chr20:45722456-45749534	27079	LOSS	SNP	SNP	Fam8	8.3	8: f/m/aM/uF	maternal	SPINT4	.	no
20q13.33	chr20:62821525-62833156	11632	LOSS	SNP/GS	SNP/GS	Fam96	96.3	96: f/m/aM/uF	paternal	COL9A3	COL9A3(cand)	yes
21q22.11	chr21:31165520-31653872	488353	LOSS	SNP/GS	SNP/GS	Fam55	55.3	55: f/m/aM/aM/dM	paternal	TIAM1,TIAM1-AS1	TIAM1(cand)	yes
21q22.2	chr21:39375414-39412460	37047	GAIN	SNP/GS	SNP/GS	Fam57	57.3	57: f/m/aM/uF	maternal	GET1,GET1-SH3BGR,LCA5L	GET1(cand)	no
21q22.3	chr21:46140262-46168679	28418	LOSS	SNP/GS	SNP/GS	Fam64	64.3	64: f/m/aM	paternal	FTCD,FTCD-AS1,SPATC1L	FTCD(HC)	yes
22q11.23	chr22:24678665-24784399	105735	GAIN	SNP/GS	SNP/GS	Fam113	113.4	113: f/m/aF/raM/uF	N/A	PIWIL3,TOP1P2	.	no
22q12.2	chr22:29932161-29970614	38454	LOSS	SNP/GS	GS	Fam31	31.3	31: f/m/aM/uF	maternal	MTMR3	MTMR3(cand)	no
22q12.2	chr22:30600940-30795509	194570	GAIN	SNP/GS	SNP/GS	Fam86	86.3	86: f/m/aF	maternal	DUSP18,MIR3200,OSBP2,PES1,SLC35E4,TCN2	TCN2(HC)	no
18p11.22-p11.21	chr18:9291609-12509914	3218306	GAIN	SNP/GS	SNP/GS	Fam9	9.4	9: f/m/aM/**dM**/uF	***de novo***	AFG3L2,ANKRD62,APCDD1,C18orf61,CHMP1B,CIDEA,GNAL,IMPA2,LINC01254,LINC01255,LINC01887,LINC01928,LOC101927410,LOC105371998,MIR6788,MIR7153,MPPE1,NAPG,PIEZO2,PPP4R1,PPP4R1-AS1,PRELID3A,RAB31,RALBP1,SLC35G4,SPIRE1,TUBB6,TWSG1,TWSG1-DT,TXNDC2,VAPA	NAPG(cand),PIEZO2(HC),PPP4R1(cand),RAB31(cand),RALBP1(cand), VAPA(cand),GNAL(cand),AFG3L2(cand)	yes
Xp22.33	chrX:254466-287975	33510	LOSS	SNP/GS	GS	Fam97	97.4	97: f/m/aM/aF	maternal	PLCXD1	.	no
Xp22.33	chrX:1167810-1197997	30188	LOSS	SNP/GS	GS	Fam21	21.3	21: f/m/aM/uM	maternal	CRLF2	.	no
Xp22.33	chrX:1315131-1566172	251042	GAIN	SNP/GS	GS	Fam33	33.3	33: f/m/aM/uM	maternal	ASMTL,ASMTL-AS1,CSF2RA,IL3RA,LINC00106,P2RY8,SLC25A6	ASMTL(cand),SLC25A6(cand)	no
Xp22.31	chrX:6532399-8168495	1636097	GAIN	SNP/GS	SNP/GS	Fam23	23.4	23: f/m/aM/dF/uFs	paternal	MIR4767,PUDP,STS,VCX3A,PNPLA4,MIR651,VCX	STS(cand)	no
Xp22.11	chrX:23384110-24802539	1418430	GAIN	SNP/GS	SNP/GS	Fam15	15.4	15: f/m/aM/aM	maternal	ACOT9,APOO,CXorf58,EIF2S3,KLHL15,LOC127933115,PCYT1B,PCYT1B-AS1,PDK3,POLA1,PRDX4,PTCHD1,SAT1,SAT1-DT,SCARNA23,SUPT20HL1,SUPT20HL2,ZFX,ZFX-AS1	APOO(cand),EIF2S3(HC),KLHL15(HC),POLA1(HC),PRDX4(cand),PTCHD1(HC),SAT1(cand),ZFX(cand)	no
Xp22.11	chrX:23831645-23851788	20144	GAIN	SNP/GS	GS	Fam85	85.3	85: f/m/aM/aM	maternal	APOO	APOO(cand)	no
Xp11.4	chrX:40513917-40631198	117282	GAIN	SNP/GS	SNP/GS	Fam33	33.3	33: f/m/aM/uM	maternal	ATP6AP2,CXorf38,MPC1L	ATP6AP2(HC)	no
Xp11.21	chrX:57252079-57397441	145363	LOSS	SNP/GS	SNP/GS	Fam113	113.3	113: f/m/aF/raM/uF	paternal	FAAH2	FAAH2(cand)	no
Xq13.3	chrX:75784933-76167591	382659	GAIN	SNP	SNP	Fam117	117.4	117: f/m/aM/aF	maternal	MAGEE2	.	no

[1] Children are listed sequentially after the father and the mother, according to recruitment order. Family members carrying the CNV are underlined; if the CNV is a *de novo* variant, the heterozygous is also indicated in bold. Abbreviations: a, affected; u, unaffected; d, specific learning disability; M, male; F, female; f, father; m, mother; r, relative (uncle); [2] NDD genes reported in GeneTrek (https://genetrek.pasteur.fr/) are indicated as "HC" (high-confidence) or "cand" (candidates) in brackets.

Supplementary Table 11: Rare homozygous and compound heterozygous pdSNVs

Chr	Position (hg38)	Ref	Alt	Gene	Amino acid change [1]	Missense classification (MPC score)	Event type	Individual	Segregation [2]	Nr homozygotes (gnomADv2.1.1/v 3.1.2)	GnomAD pRec score [3]	Mode of inheritance in the Genomics England neurology and NDD panel	GeneTrek classification	High-confidence ASD/NDD gene list [4]
**chr1**	**18691870**	**G**	**A**	***PAX7***	**NM_001135254.2:c.703G>A:p.A235T**	**DMisA (1.07)**	**Compound heterozygous (SNV/CNV) [5]**	**91.3**	**91: m/aM/dM/aM/dM**	**0/0**	**0.8546**	**biallelic**	**Candidate -NDD**	**.**
chr2	70087994	A	G	*PCBP1*	NM_006196.4:c.251A>G:p.N84S	DMisA (1.13)	Homozygous	99.3	99: f/m/aM/aM	1 Male/0	0.089934	.	Candidate -NDD	.
chr3	48412897	C	T	*PLXNB1*	NM_001130082.3:c.4699G>A:p.D1567N	DMisA (1.47)	Compound heterozygous	116.4	116: f/m/aM/aF	1 Female/0	0.99962	.	Candidate -NDD	ASD
chr3	48406876	T	G	*PLXNB1*	NM_001130082.3:c.6175A>C:p.T2059P	DMisA (1.27)	Compound heterozygous	116.4	116: f/m/aM/aF	0/0	0.99962	.	Candidate -NDD	ASD
chr12	6492309	G	A	*MRPL51*	NM_016497.4:c.349C>T:p.L117F	DMisA (1.27)	Homozygous	1.3	1: f/m/aM	1 Female/1 Male	0.83089	.	.	.
**chr14**	**102016376**	**T**	**C**	***DYNC1H1***	**NM_001376.5:c.7501T>C:p.S2501P**	**DMisB (2.52)**	**Possible compound heterozygous**	**122.3**	**122: f/m/aM**	**novel**	**1.2281E-29**	**monoallelic**	**HC-NDD**	**ASD**
**chr14**	**101988838**	**G**	**C**	***DYNC1H1***	**NM_001376.5:c.2854G>C:p.E952Q**	**DMisA (1.18)**	**Possible compound heterozygous**	**122.3**	**122: f/m/aM**	**novel**	**1.2281E-29**	**monoallelic**	**HC-NDD**	**ASD**
chr19	49595846	G	C	*PRR12*	NM_020719.3:c.1511G>C:p.G504A	DMisA (1.23)	Compound heterozygous	53.3	53: f/m/aM	0/0	7.6646E-10	monoallelic	HC-NDD	ASD
chr19	49596935	G	A	*PRR12*	NM_020719.3:c.2600G>A:p.R867H	DMisA (1.22)	Compound heterozygous	53.3	53: f/m/aM	0/2 Female	7.6646E-10	monoallelic	HC-NDD	ASD
**chr21**	**40369136**	**C**	**A**	***DSCAM***	**NM_001389.5:c.618G>T:p.E206D**	**DMisA (1.40)**	**Compound heterozygous**	**120.4**	**120: f/m/aM/aM**	**novel**	**2.8346E-08**	**Not set**	**HC-NDD**	**ASD**
**chr21**	**40189118**	**C**	**T**	***DSCAM***	**NM_001389.5:exon12:c.G2477A:p.R826Q**	**DMisA (1.36)**	**Compound heterozygous**	**120.4**	**120: f/m/aM/aM**	**0/0**	**2.8346E-08**	**Not set**	**HC-NDD**	**ASD**

The table lists rare homozygous and compound heterozygous pdSNVs (MAF ≤1% in reference databases). Homozygous and compound heterozygous pdSNVs retained after restrincting the analysis to novel variants are indicated in bold.

[1] Amino acid changes are reported according to the MANE isoform.

[2] In the segregation column, children are listed sequentially after the father and the mother, according to the recruitment order. Family member carrying the variant are underlined; if the variant is homozygous, the carrier is also indicated in blue. *De novo* variants are indicated in red. Abbreviations: f, father; m, mother; a, affected; u, unaffected; d, specific learning disability; M, male; F, female.

[3] GnomAD pRec score: Probability that transcript falls into distribution of recessive genes (~46% o/e pLoF ratio; computed from gnomAD data). A pRec score > 0.5 indicates genes with a higher probability of intolerance to biallelic loss of function.

[4] High-confidence ASD/NDD gene list classification according to Supplementary Table 8.

[5] The PAX7 deletion identified in family 91 is reported in Table 2.

Supplementary Table 12: Rare hemizygous pdSNVs

Chr	Position (hg38)	Ref	Alt	Gene	Amino acid change [1]	Missense classification (MPC score)	ASD Male(s)	Segregation [2]	Nr hemizygotes (gnomAD non-neuro v2.1.1/v3.1.2) [3]	GeneTrek classification
chrX	10067293	C	G	WWC3	NM_015691.5:exon3:c.23C>G:p.T8S	DMisA (1.009)	69.3	69: f/m/aM	0/NF	Candidate -NDD
chrX	18941608	A	C	PHKA2	NM_000292.3:exon8:c.785T>G:p.I262S	DMisA (1.858)	66.3	66: f/m/aM	11/15	.
chrX	21655991	C	T	KLHL34	NM_153270.3:c.1798G>A:p.D600N	DMisA (1.278)	114.3	114: f/m/aM	1/NF	.
chrX	41224766	T	C	USP9X	NM_001039591.3:c.6776T>C:p.F2259S	DMisA (1.563)		91: m/aM/dM/aM/dM	novel	HC-NDD
chrX	41474066	C	G	NYX	NM_001378477.3:c.598C>G:p.L200V	DMisA (1.237)	51.3	51: f/m/aM/uF	novel	Candidate -NDD
chrX	45051714	A	T	KDM6A	NM_001291415.2:c.660A>T:p.K220N	DMisA (1.884)	118.3	118: f/m/aM/aF/uM	0/1	HC-NDD
chrX	47626101	G	A	CFP	NM_001145252.3:c.1201C>T:p.R401C	DMisA (1.914)	53.3	53: f/m/aM	0/NF	.
chrX	48601844	G	T	WDR13	NM_001347217.2:c.892G>T:p.G298W	DMisB (2.355)	15.4	15: f/m/aM/aM	8/3	Candidate -NDD
chrX	50086401	G	A	CLCN5	NM_001127898.4:c.1088G>A:p.R363H	DMisA (1.583)	119.3	119: f/m/aM/aM	0/2	Candidate -NDD
chrX	55452768	C	T	MAGEH1	NM_014061.5:c.394C>T:p.P132S	DMisA (1.534)	92.3	92: f/m/aM/raM	novel	Candidate -NDD
chrX	65518271	G	C	LAS1L	NM_031206.7:c.1643C>G:p.S548C	DMisA (1.057)	121.4	121: f/m/aF/aM	novel	HC-NDD
chrX	65729651	G	A	MSN	NM_002444.3:exon4:c.406G>A:p.D136N	DMisA (1.900)	92.3	92: f/m/aM/raM	2/NF	Candidate -NDD
chrX	69529693	T	C	NALF2	NM_015686.3:c.1156T>C:p.F386L	DMisA (1.201)	34.3	34: f/m/aM/uF	2/1	.
chrX	105767516	A	G	IL1RAPL2	NM_017416.2:c.1916A>G:p.H639R	DMisA (1.063)	91.3, 91.5	91: m/aM/dM/aM/dM	4/3	Candidate -NDD
chrX	106928284	T	C	CLDN2	NM_020384.4:c.56T>C:p.L19S	DMisA (1.193)	39.3	39: f/m/aM	12/2	.
chrX	106986166	G	T	MORC4	NM_024657.5:c.343C>A:p.Q115K	DMisA (1.083)	105.3	105: f/m/aM	4/2	Candidate -NDD
chrX	106986167	G	C	MORC4	NM_024657.5:c.342C>G:p.S114R	DMisA (1.151)	105.3	105: f/m/aM	4/2	Candidate -NDD
chrX	109665485	T	C	ACSL4	NM_001318510.2:c.1325A>G:p.Y442C	DMisA (1.635)	9.3	9: f/m/aM/dM/uF	34/15	HC-NDD
chrX	110317627	G	A	AMMECR1	NM_015365.3:c.445C>T:p.R149W	DMisA (1.764)	81.3	81: f/m/aM/uM	novel	Candidate -NDD
chrX	129565827	G	A	OCRL	NM_000276.4:c.1300G>A:p.E434K	DMisB (2.017)	56.4	56: f/m/aF/aM	novel	HC-NDD
chrX	129788035	G	A	SASH3	NM_018990.4:c.118G>A:p.V40M	DMisA (1.296)	23.3	23: f/m/aM/dF/uFs	1/0	.
chrX	129793748	G	C	SASH3	NM_018990.4:c.1059G>C:p.E353D	DMisA (1.248)	5.3	5: f/m/aM	novel	.
chrX	134413584	A	T	PHF6	NM_001015877.2:c.512A>T:p.N171I	DMisA (1.325)	39.3	39: f/m/aM	novel	HC-NDD
chrX	135552001	G	A	INTS6L	NM_001351601.3:c.914G>A:p.R305Q	DMisA (1.378)	21.3	21: f/m/aM/uM	novel	Candidate -NDD
chrX	139802242	A	C	ATP11C	NM_001353812.2:c.653T>G:p.L218R	DMisA (1.623)	47.3	47: f/m/aM	0/1	Candidate -NDD
chrX	153725316	G	A	ABCD1	NM_000033.4:c.50G>A:p.R17H	DMisA (1.404)	44.3	44: f/m/aM/uF	3/4	HC-NDD
chrX	153785963	G	A	IDH3G	NM_004135.4:c.1091C>T:p.P364L	DMisA (1.241)	92.4	92: f/m/aM/raM	0/0	Candidate -NDD
chrX	153905849	G	A	AVPR2	NM_000054.7:c.343G>A:p.V115M	DMisA (1.399)	51.3	51: f/m/aM/uF	2/1	Candidate -NDD
chrX	153951650	G	A	HCFC1	NM_005334.3:c.5318C>T:p.A1773V	DMisA (1.450)	53.3	53: f/m/aM	novel	HC-NDD
chrX	154442150	C	T	GDI1	NM_001493.3:c.1031C>T:p.A344V	DMisB (2.277)	39.3	39: f/m/aM	novel	HC-NDD
chrX	155046562	G	A	FUNDC2	NM_023934.4:c.338G>A:p.G113E	DMisA (1.027)	20.3	20: f/m/aM/uF	novel	.
chrX	155077198	A	G	BRCC3	NM_001018055.3:c.224A>G:p.H75R	DMisB (2.221)	16.3, 16.4	16: f/m/aM/aM	0/0	.

List of hemizygous pdSNVs identified in male probands and absent in unaffected brother(s).

[1] Amino acid changes are reported according to the MANE isoform.

[2] In the segregation column, children are listed sequentially after the father and the mother, according to the recruitment order. Family member carrying the variant are underlined. Abbreviations: f, father; m, mother; r, relative (uncle); a, affected: u, unaffected; d, specific learning disability; M, male; F, female.

[3] Number of hemizygotes: 0, the variant is present in GnomAD but no hemizygotes are listed; NF, the variant is not found in the specific release of GnomAD; novel, the variant is absent in both GnomAD releases.

Supplementary Table 6: List of *de novo* and inherited pdSNVs in cases

Chr	Start (hg38)	End (hg38)	Ref	Alt	Gene (RefSeq)	Exonic/ splicing	Consequence	Amino acid change	LOEUF score (gnomAD v4)	MPC score	gnomADv2.1 (genome, non neuro sample, AF popmax)	gnomADv2.1 (exome, non neuro sample, AF popmax)	gnomADv3.0 (AF total)	Family	Genotypes
chr1	1042075	1042075	G	A	AGRN	exonic	nonsynonymous SNV	AGRN:NM_198576:exon7:c.G1297A:p.G433R	.	1.405	0.0002	9.91E-05	2.09E-05	Fam91_m_aM_dM_aM_dM	0/1;0/1;0/0;0/0;0/0
chr1	1232763	1232763	G	A	B3GALT6	exonic	nonsynonymous SNV	B3GALT6:NM_080605:exon1:c.G485A:p.R162Q	.	1.982	0.0002	0.0004	0.0004	Fam12_f_m_aM_uM_aM	0/0;0/1;0/1;0/0;0/1
chr1	1314329	1314329	C	T	INTS11	exonic	nonsynonymous SNV	INTS11:NM_001256462:exon6:c.G445A:p.E149K,INTS11:NM_001256463:exon6:c.G436A:p.E146K,INTS11:NM_017871:exon8:c.G739A:p.E247K,INTS11:NM_001256460:exon9:c.G652A:p.E218K,INTS11:NM_001256456:exon10:c.G757A:p.E253K	.	1.568	.	6.94E-05	6.98E-06	Fam30_f_m_aM_uM	0/0;0/1;0/1;0/1
chr1	1623505	1623505	G	A	MIB2	exonic	nonsynonymous SNV	MIB2:NM_001170686:exon3:c.G227A:p.G76D,MIB2:NM_001170687:exon3:c.G185A:p.G62D,MIB2:NM_001170688:exon3:c.G398A:p.G133D,MIB2:NM_001170689:exon3:c.G53A:p.G18D,MIB2:NM_080875:exon3:c.G227A:p.G76D	.	1.502	.	.	.	Fam56_f_m_aF_aM	0/0;0/1;0/1;0/0
chr1	1623873	1623873	C	T	MIB2	exonic	nonsynonymous SNV	MIB2:NM_001170686:exon4:c.C521T:p.T174M,MIB2:NM_001170687:exon4:c.C479T:p.T160M,MIB2:NM_001170688:exon4:c.C692T:p.T231M,MIB2:NM_001170689:exon4:c.C347T:p.T116M,MIB2:NM_080875:exon4:c.C521T:p.T174M	.	1.319	.	7.76E-05	6.98E-06	Fam108_f_m_aM	0/0;0/1;0/1
chr1	1628693	1628693	G	C	MIB2	exonic	nonsynonymous SNV	MIB2:NM_001170688:exon15:c.G2323C:p.D775H,MIB2:NM_001170689:exon15:c.G1978C:p.D660H,MIB2:NM_001170686:exon16:c.G2335C:p.D779H,MIB2:NM_001170687:exon16:c.G2305C:p.D769H,MIB2:NM_080875:exon16:c.G2347C:p.D783H	.	1.545	7.35E-05	0.0005	0.0002	Fam110_f_m_aM_aM_uMs	0/0;0/1;0/1;0/1;0/0
chr1	2029673	2029673	G	A	GABRD	exonic	nonsynonymous SNV	GABRD:NM_000815:exon8:c.G970A:p.A324T	.	1.8	.	.	.	Fam88_f_m_aF	0/1;0/0;0/1
chr1	2173968	2173968	A	G	PRKCZ	exonic	nonsynonymous SNV	PRKCZ:NM_001033581:exon11:c.A808G:p.I270V,PRKCZ:NM_001033582:exon11:c.A808G:p.I270V,PRKCZ:NM_001242874:exon11:c.A1045G:p.I349V,PRKCZ:NM_001350803:exon11:c.A832G:p.I278V,PRKCZ:NM_001350805:exon11:c.A646G:p.I216V,PRKCZ:NM_001350806:exon11:c.A646G:p.I216V,PRKCZ:NM_001350804:exon12:c.A832G:p.I278V,PRKCZ:NM_002744:exon14:c.A1357G:p.I453V	.	1.049	7.34E-05	3.37E-05	3.49E-05	Fam94_f_m_aM	0/1;0/0;0/1
chr1	2526560	2526560	C	T	PANK4	exonic	nonsynonymous SNV	PANK4:NM_018216:exon1:c.G28A:p.G10R	.	1.037	.	.	6.98E-06	Fam6_f_m_aM	0/1;0/0;0/1
chr1	4655448	4655448	G	A	AJAP1	exonic	nonsynonymous SNV	AJAP1:NM_001042478:exon1:c.G23A:p.G8E,AJAP1:NM_018836:exon1:c.G23A:p.G8E	.	1.062	0.0003	1.29E-05	1.40E-05	Fam52_f_m_aM	0/0;0/1;0/1
chr1	6051603	6051603	C	G	KCNAB2	exonic	nonsynonymous SNV	KCNAB2:NM_001199862:exon2:c.C67G:p.L23V	.	1.026	.	.	.	Fam12_f_m_aM_uM_aM	0/1;0/0;0/1;0/1;0/1
chr1	6245169	6245169	G	C	HES3	exonic	nonsynonymous SNV	HES3:NM_001024598:exon4:c.G223C:p.G75R	.	2.081	.	.	.	Fam31_f_m_aM_uF	0/1;0/0;0/1;0/0
chr1	6249397	6249397	G	C	GPR153	exonic	nonsynonymous SNV	GPR153:NM_207370:exon6:c.C1771G:p.P591A	.	1.007	.	.	.	Fam57_f_m_aM_uF	0/0;0/1;0/1;0/0
chr1	6253852	6253852	C	T	GPR153	exonic	nonsynonymous SNV	GPR153:NM_207370:exon3:c.G652A:p.V218M	.	1.034	0.0002	0.0001	9.07E-05	Fam97_f_m_aM_aF	0/1;0/0;0/0;0/1
chr1	6253891	6253891	G	A	GPR153	exonic	nonsynonymous SNV	GPR153:NM_207370:exon3:c.C613T:p.R205C	.	1.084	0.0004	0.0004	0.0002	Fam18_f_m_aM_uF	0/0;0/1;0/1;0/1
chr1	6418916	6418916	G	A	HES2	exonic	nonsynonymous SNV	HES2:NM_019089:exon4:c.C479T:p.P160L	.	1.77	.	.	6.98E-06	Fam60_f_m_aF	0/1;0/0;0/1
chr1	7664621	7664621	A	T	CAMTA1	exonic	nonsynonymous SNV	CAMTA1:NM_001349608:exon8:c.A1984T:p.M662L,CAMTA1:NM_001349612:exon8:c.A1984T:p.M662L,CAMTA1:NM_001349609:exon9:c.A2074T:p.M692L,CAMTA1:NM_001349610:exon9:c.A2074T:p.M692L,CAMTA1:NM_015215:exon9:c.A2074T:p.M692L	.	1.139	.	3.28E-05	6.98E-06	Fam121_f_m_aF_aM	0/0;0/1;0/0;0/1
chr1	7737538	7737538	A	G	CAMTA1	exonic	nonsynonymous SNV	CAMTA1:NM_001349613:exon5:c.A755G:p.K252R,CAMTA1:NM_001349615:exon5:c.A698G:p.K233R,CAMTA1:NM_001349616:exon5:c.A698G:p.K233R,CAMTA1:NM_001349618:exon5:c.A698G:p.K233R,CAMTA1:NM_001349619:exon5:c.A698G:p.K233R,CAMTA1:NM_001349622:exon5:c.A698G:p.K233R,CAMTA1:NM_001349624:exon5:c.A698G:p.K233R,CAMTA1:NM_001349626:exon5:c.A698G:p.K233R,CAMTA1:NM_001349614:exon7:c.A698G:p.K233R,CAMTA1:NM_001349617:exon7:c.A698G:p.K233R,CAMTA1:NM_001349620:exon7:c.A698G:p.K233R,CAMTA1:NM_001349621:exon7:c.A698G:p.K233R,CAMTA1:NM_001349623:exon7:c.A698G:p.K233R,CAMTA1:NM_001349625:exon7:c.A698G:p.K233R,CAMTA1:NM_001349608:exon14:c.A3536G:p.K1179R,CAMTA1:NM_001349612:exon14:c.A3536G:p.K1179R,CAMTA1:NM_001349609:exon15:c.A3626G:p.K1209R,CAMTA1:NM_001349610:exon15:c.A3626G:p.K1209R,CAMTA1:NM_015215:exon15:c.A3626G:p.K1209R	.	1.245	7.34E-05	0.0003	0.0002	Fam18_f_m_aM_uF	0/1;0/0;0/1;0/1
chr1	9718765	9718765	C	A	PIK3CD	exonic	nonsynonymous SNV	PIK3CD:NM_001350235:exon8:c.C1005A:p.S335R,PIK3CD:NM_001350234:exon9:c.C1092A:p.S364R,PIK3CD:NM_005026:exon9:c.C1092A:p.S364R	.	1.324	.	1.12E-05	1.40E-05	Fam53_f_m_aM	0/1;0/0;0/1
chr1	10304537	10304537	C	T	KIF1B	exonic	stopgain	KIF1B:NM_001365953:exon21:c.C3352T:p.Q1118X,KIF1B:NM_183416:exon21:c.C3352T:p.Q1118X	0.385	.	.	.	.	Fam4_f_m_aM	0/1;0/0;0/1
chr1	11055069	11055069	C	T	SRM	exonic	nonsynonymous SNV	SRM:NM_003132:exon7:c.G781A:p.E261K	.	1.181	0.0005	0.0005	0.0003	Fam88_f_m_aF	0/1;0/0;0/1

chr1	11069564	11069564	A	G	EXOSC10	exonic	nonsynonymous SNV	EXOSC10:NM_002685:exon21:c.T2408C:p.F803S,EXOSC10:NM_001001998:exon22:c.T2483C:p.F828S	.	1.115	.	.	.	Fam37_f_m_aF_uM	0/1;0/0;0/1;0/1
chr1	11133109	11133109	C	T	MTOR	exonic	nonsynonymous SNV	MTOR:NM_004958:exon38:c.G5335A:p.A1779T	.	1.201	.	3.28E-05	.	Fam60_f_m_aF	0/1;0/0;0/1
chr1	11515488	11515488	G	A	DISP3	exonic	nonsynonymous SNV	DISP3:NM_020780:exon5:c.G1573A:p.V525M	.	1.15	0.0003	4.64E-05	6.98E-05	Fam68_f_m_aF_uF_uM	0/1;0/0;0/1;0/0;0/0
chr1	11649833	11649833	C	T	FBXO2	exonic	nonsynonymous SNV	FBXO2:NM_012168:exon4:c.G563A:p.G188D	.	1.141	.	.	.	Fam56_f_m_aF_aM	0/1;0/0;0/1;0/1
chr1	12192518	12192518	A	G	TNFRSF1B	exonic	nonsynonymous SNV	TNFRSF1B:NM_001066:exon5:c.A545G:p.H182R	.	1.221	.	.	.	Fam99_f_m_aM_aM	0/1;0/0;0/1;0/0
chr1	15044062	15044062	G	A	KAZN	exonic	nonsynonymous SNV	KAZN:NM_001017999:exon4:c.G347A:p.R116H,KAZN:NM_001018000:exon4:c.G611A:p.R204H,KAZN:NM_001018001:exon4:c.G347A:p.R116H,KAZN:NM_001370229:exon4:c.G602A:p.R201H,KAZN:NM_001370230:exon4:c.G434A:p.R145H,KAZN:NM_015209:exon4:c.G629A:p.R210H,KAZN:NM_201628:exon4:c.G629A:p.R210H,KAZN:NM_001370231:exon5:c.G626A:p.R209H	.	1.9	7.37E-05	5.65E-05	9.08E-05	Fam72_f_m_aF_uF	0/1;0/0;0/1;0/1
chr1	15578976	15578976	G	T	AGMAT	exonic	nonsynonymous SNV	AGMAT:NM_024758:exon4:c.C603A:p.H201Q	.	1.034	0.001	0.0008	0.0006	Fam29_f_m_aF_uM	0/0;0/1;0/1;0/0
chr1	15617745	15617745	C	G	DDI2	exonic	nonsynonymous SNV	DDI2:NM_032341:exon1:c.C75G:p.F25L	.	1.131	.	.	.	Fam89_f_m_aM	0/1;0/0;0/1
chr1	15930486	15930486	C	T	SPEN	exonic	nonsynonymous SNV	SPEN:NM_015001:exon11:c.C4246T:p.R1416C	.	1.011	.	3.27E-05	.	Fam89_f_m_aM	0/1;0/0;0/1
chr1	15946317	15946317	C	G	ZBTB17	splicing	.	.	0.472	.	.	0.0002	4.19E-05	Fam51_f_m_aM_uF	0/1;0/0;0/1;0/1
chr1	16251656	16251656	G	A	FBXO42	exonic	nonsynonymous SNV	FBXO42:NM_018994:exon10:c.C1168T:p.R390C	.	1.05	7.35E-05	0.0002	2.10E-05	Fam102_f_m_aM	0/1;0/0;0/1
chr1	17623068	17623068	C	T	ARHGEF10L	exonic	nonsynonymous SNV	ARHGEF10L:NM_001319838:exon4:c.C427T:p.R143C,ARHGEF10L:NM_001328124:exon4:c.C427T:p.R143C,ARHGEF10L:NM_001011722:exon10:c.C976T:p.R326C,ARHGEF10L:NM_001319837:exon10:c.C976T:p.R326C,ARHGEF10L:NM_018125:exon12:c.C1093T:p.R365C	.	1.681	.	0.0002	5.58E-05	Fam90_f_m_aF_dM	0/0;0/1;0/1;0/1
chr1	17697234	17697234	C	T	ARHGEF10L	exonic	nonsynonymous SNV	ARHGEF10L:NM_001319838:exon18:c.C2803T:p.R935C,ARHGEF10L:NM_001328124:exon21:c.C3028T:p.R1010C,ARHGEF10L:NM_001319837:exon26:c.C3562T:p.R1188C,ARHGEF10L:NM_001011722:exon27:c.C3577T:p.R1193C,ARHGEF10L:NM_018125:exon29:c.C3694T:p.R1232C	.	1.111	0.0003	0.0003	0.0001	Fam28_f_m_aF_uF	0/0;0/1;0/1;0/0
chr1	18691870	18691870	G	A	PAX7	exonic	nonsynonymous SNV	PAX7:NM_001135254:exon5:c.G703A:p.A235T,PAX7:NM_002584:exon5:c.G703A:p.A235T,PAX7:NM_013945:exon5:c.G697A:p.A233T	.	1.067	0.0009	0.0009	0.0003	Fam91_m_aM_dM_aM_dM	0/1;1/1;0/0;0/0;0/0
chr1	18703204	18703204	C	T	PAX7	exonic	nonsynonymous SNV	PAX7:NM_001135254:exon7:c.C1063T:p.R355C,PAX7:NM_002584:exon7:c.C1063T:p.R355C,PAX7:NM_013945:exon7:c.C1057T:p.R353C	.	1.466	.	3.27E-05	.	Fam118_f_m_aM_aF_uM	0/1;0/0;0/1;0/0;0/1
chr1	19095606	19095606	C	T	UBR4	exonic	nonsynonymous SNV	UBR4:NM_020765:exon93:c.G13565A:p.R4522Q	.	1.927	0.0001	0.0001	8.38E-05	Fam75_f_m_aM	0/0;0/1;0/1
chr1	19105073	19105073	G	A	UBR4	exonic	nonsynonymous SNV	UBR4:NM_020765:exon85:c.C12620T:p.P4207L	.	1.82	0.0003	0.0008	0.0001	Fam6_f_m_aM	0/0;0/1;0/1
chr1	19317726	19317726	G	A	SLC66A1	exonic	nonsynonymous SNV	SLC66A1:NM_001040125:exon2:c.G49A:p.G17S,SLC66A1:NM_017765:exon3:c.G49A:p.G17S	.	1.064	.	0.0003	5.58E-05	Fam92_f_m_aM_raM	0/0;0/0;0/0;0/1
chr1	19357433	19357433	C	T	CAPZB	exonic	nonsynonymous SNV	CAPZB:NM_001206540:exon5:c.G460A:p.V154I,CAPZB:NM_001282162:exon5:c.G547A:p.V183I,CAPZB:NM_004930:exon5:c.G460A:p.V154I,CAPZB:NM_001206541:exon6:c.G538A:p.V180I	.	2.298	.	0.0005	.	Fam97_f_m_aM_aF	0/0;0/1;0/1;0/0
chr1	19666013	19666013	C	G	HTR6	exonic	nonsynonymous SNV	HTR6:NM_000871:exon1:c.C260G:p.A87G	.	1.112	.	2.24E-05	.	Fam77_f_m_aM_aF_uM_uFs	0/0;0/1;0/0;0/1;0/1;0/1
chr1	19907682	19907682	G	T	OTUD3	exonic	nonsynonymous SNV	OTUD3:NM_015207:exon8:c.G1133T:p.R378I	.	1.247	.	.	.	Fam107_f_m_aM	0/1;0/0;0/1
chr1	20672181	20672181	C	G	KIF17	exonic	nonsynonymous SNV	KIF17:NM_001122819:exon12:c.G2479C:p.V827L,KIF17:NM_001287212:exon12:c.G2179C:p.V727L,KIF17:NM_020816:exon12:c.G2479C:p.V827L	.	1.098	.	3.28E-05	.	Fam43_f_m_aM	0/0;0/1;0/1
chr1	20879466	20879466	T	A	EIF4G3	exonic	nonsynonymous SNV	EIF4G3:NM_003760:exon15:c.A2311T:p.N771Y,EIF4G3:NM_001198801:exon18:c.A2419T:p.N807Y,EIF4G3:NM_001198802:exon18:c.A2329T:p.N777Y	.	1.797	.	1.24E-05	.	Fam47_f_m_aM	0/0;0/1;0/1
chr1	21247309	21247309	C	T	ECE1	exonic	nonsynonymous SNV	ECE1:NM_001113347:exon7:c.G1039A:p.V347M,ECE1:NM_001113349:exon8:c.G1066A:p.V356M,ECE1:NM_001113348:exon9:c.G1027A:p.V343M,ECE1:NM_001397:exon9:c.G1075A:p.V359M	.	1.512	.	6.17E-05	.	Fam26_f_m_aM_uM	0/0;0/1;0/1;0/0
chr1	21695151	21695151	C	T	USP48	exonic	nonsynonymous SNV	USP48:NM_001350164:exon22:c.G2597A:p.R866H,USP48:NM_001350166:exon23:c.G2792A:p.R931H,USP48:NM_001350167:exon23:c.G2795A:p.R932H,USP48:NM_001350168:exon23:c.G2795A:p.R932H,USP48:NM_032236:exon23:c.G2798A:p.R933H	.	2.516	.	.	.	Fam66_f_m_aM	0/0;0/1;0/1
chr1	22129640	22129640	C	T	WNT4	exonic	nonsynonymous SNV	WNT4:NM_030761:exon2:c.G289A:p.V97I	.	1.102	.	0.0001	.	Fam83_f_m_aF	0/0;0/1;0/1
chr1	22129681	22129681	C	T	WNT4	exonic	nonsynonymous SNV	WNT4:NM_030761:exon2:c.G248A:p.R83Q	.	1.289	7.35E-05	6.56E-05	1.40E-05	Fam94_f_m_aM	0/1;0/0;0/1
chr1	22864889	22864889	C	T	EPHB2	exonic	nonsynonymous SNV	EPHB2:NM_001309192:exon5:c.C980T:p.A327V,EPHB2:NM_001309193:exon5:c.C980T:p.A327V,EPHB2:NM_004442:exon5:c.C980T:p.A327V,EPHB2:NM_017449:exon5:c.C980T:p.A327V	.	1.293	.	0.0002	1.40E-05	Fam47_f_m_aM	0/0;0/1;0/1
chr1	23072185	23072185	C	T	KDM1A	exonic	nonsynonymous SNV	KDM1A:NM_001363654:exon12:c.C1538T:p.A513V,KDM1A:NM_015013:exon12:c.C1538T:p.A513V,KDM1A:NM_001009999:exon14:c.C1610T:p.A537V	.	1.645	7.34E-05	0.0002	0.0001	Fam118_f_m_aM_aF_uM	0/1;0/0;0/1;0/0;0/1
chr1	26114435	26114435	G	A	PDIK1L	exonic	nonsynonymous SNV	PDIK1L:NM_001243532:exon2:c.G127A:p.A43T,PDIK1L:NM_152835:exon2:c.G127A:p.A43T,PDIK1L:NM_001243533:exon3:c.G127A:p.A43T	.	1.081	.	.	.	Fam112_f_m_aM	0/1;0/0;0/1
chr1	26950738	26950738	G	A	KDF1	exonic	nonsynonymous SNV	KDF1:NM_152365:exon3:c.C1058T:p.S353F	.	1.036	.	.	.	Fam81_f_m_aM_uM	0/0;0/0;0/1;0/0
chr1	27006417	27006417	G	A	TENT5B	exonic	nonsynonymous SNV	TENT5B:NM_052943:exon2:c.C805T:p.R269C	.	1.342	.	.	.	Fam79_f_m_aM_uM	0/1;0/0;0/1;0/1
chr1	27107758	27107758	T	C	SLC9A1	exonic	nonsynonymous SNV	SLC9A1:NM_003047:exon4:c.A1172G:p.E391G	.	2.299	.	.	.	Fam98_f_m_aM	0/0;0/0;0/1

chr1	27109732	27109732	C	T	SLC9A1	exonic	nonsynonymous SNV	SLC9A1:NM_003047:exon3:c.G859A:p.G287S	.	1.005	0.0003	0.0004	0.0003	Fam92_f_m_aM_raM	0/0;0/1;0/1;0/0
chr1	27547531	27547531	G	A	AHDC1	exonic	nonsynonymous SNV	AHDC1:NM_001029882:exon6:c.C4585T:p.R1529C	.	1.661	0.0006	0.0006	0.0003	Fam5_f_m_aM	0/0;0/1;0/1
chr1	27547531	27547531	G	A	AHDC1	exonic	nonsynonymous SNV	AHDC1:NM_001029882:exon6:c.C4585T:p.R1529C	.	1.661	0.0006	0.0006	0.0003	Fam75_f_m_aM	0/1;0/0;0/1
chr1	27549810	27549810	G	A	AHDC1	exonic	nonsynonymous SNV	AHDC1:NM_001029882:exon6:c.C2306T:p.A769V	.	1.23	7.37E-05	.	6.99E-06	Fam52_f_m_aM	0/1;0/0;0/1
chr1	27551044	27551044	G	A	AHDC1	exonic	nonsynonymous SNV	AHDC1:NM_001029882:exon6:c.C1072T:p.P358S	.	1.371	0.0001	0.0002	7.68E-05	Fam112_f_m_aM	0/1;0/0;0/1
chr1	27551467	27551467	C	T	AHDC1	exonic	nonsynonymous SNV	AHDC1:NM_001029882:exon6:c.G649A:p.G217R	.	1.699	.	0.0002	2.09E-05	Fam55_f_m_aM_aM_dM	0/0;0/1;0/0;0/1;0/1
chr1	27759546	27759546	T	G	FAM76A	exonic	nonsynonymous SNV	FAM76A:NM_001143915:exon6:c.T516G:p.D172E,FAM76A:NM_001143914:exon7:c.T669G:p.D223E,FAM76A:NM_001143913:exon8:c.T771G:p.D257E,FAM76A:NM_152660:exon8:c.T756G:p.D252E,FAM76A:NM_001143912:exon9:c.T858G:p.D286E	.	1.656	.	.	6.99E-06	Fam64_f_m_aM	0/0;0/1;0/1
chr1	27801678	27801678	C	T	STX12	exonic	nonsynonymous SNV	STX12:NM_177424:exon4:c.C289T:p.R97C	.	1.172	.	1.42E-05	6.98E-06	Fam55_f_m_aM_aM_dM	0/0;0/1;0/0;0/1;0/1
chr1	27882976	27882976	C	T	THEMIS2	exonic	nonsynonymous SNV	THEMIS2:NM_001105556:exon4:c.C1652T:p.P551L,THEMIS2:NM_001286115:exon4:c.C1064T:p.P355L,THEMIS2:NM_001286113:exon5:c.C1265T:p.P422L	.	1.172	.	1.15E-05	.	Fam52_f_m_aM	0/1;0/0;0/1
chr1	28208404	28208404	G	C	DNAJC8	exonic	nonsynonymous SNV	DNAJC8:NM_014280:exon6:c.C409G:p.R137G	.	1.149	.	.	.	Fam12_f_m_aM_uM_aM	0/0;0/1;0/1;0/1;0/0
chr1	28279140	28279140	C	T	SESN2	exonic	nonsynonymous SNV	SESN2:NM_031459:exon9:c.C1255T:p.R419W	.	1.348	7.34E-05	9.83E-05	3.49E-05	Fam42_f_m_aM_uF	0/1;0/0;0/1;0/1
chr1	28714324	28714324	G	A	GMEB1	exonic	nonsynonymous SNV	GMEB1:NM_001319674:exon10:c.G1243A:p.A415T,GMEB1:NM_006582:exon10:c.G1273A:p.A425T,GMEB1:NM_024482:exon10:c.G1243A:p.A415T	.	1.121	.	2.23E-05	.	Fam71_f_m_aF	0/1;0/0;0/1
chr1	29303926	29303926	C	T	PTPRU	exonic	nonsynonymous SNV	PTPRU:NM_001195001:exon16:c.C2548T:p.R850W,PTPRU:NM_133177:exon16:c.C2548T:p.R850W,PTPRU:NM_133178:exon16:c.C2548T:p.R850W,PTPRU:NM_005704:exon17:c.C2578T:p.R860W	.	1.232	0.0003	0.0004	0.0002	Fam86_f_m_aF	0/0;0/1;0/1
chr1	31730883	31730883	G	A	ADGRB2	exonic	nonsynonymous SNV	ADGRB2:NM_001294336:exon28:c.C4198T:p.R1400C,ADGRB2:NM_001294335:exon29:c.C4297T:p.R1433C,ADGRB2:NM_001364857:exon29:c.C4297T:p.R1433C	.	1.69	0.0003	3.97E-05	4.19E-05	Fam15_f_m_aM_aM	0/1;0/0;0/0;0/1
chr1	31740453	31740453	C	T	ADGRB2	exonic	nonsynonymous SNV	ADGRB2:NM_001294335:exon12:c.G1883A:p.R628Q,ADGRB2:NM_001294336:exon12:c.G1883A:p.R628Q,ADGRB2:NM_001364857:exon12:c.G1883A:p.R628Q	.	1.108	0.0001	0.0001	4.88E-05	Fam56_f_m_aF_aM	0/0;0/1;0/0;0/1
chr1	31741895	31741895	C	T	ADGRB2	exonic	nonsynonymous SNV	ADGRB2:NM_001294335:exon9:c.G1490A:p.R497H,ADGRB2:NM_001294336:exon9:c.G1490A:p.R497H,ADGRB2:NM_001364857:exon9:c.G1490A:p.R497H	.	1.892	7.35E-05	4.49E-05	2.79E-05	Fam45_f_m_aM_uF	0/1;0/0;0/1;0/1
chr1	32037957	32037957	C	T	KHDRBS1	exonic	nonsynonymous SNV	KHDRBS1:NM_001271878:exon5:c.C911T:p.P304L,KHDRBS1:NM_006559:exon6:c.C1028T:p.P343L	.	2.11	.	.	.	Fam51_f_m_aM_uF	0/0;0/1;0/1;0/1
chr1	32181461	32181461	A	G	TXLNA	exonic	nonsynonymous SNV	TXLNA:NM_175852:exon3:c.A389G:p.N130S	.	1.077	0.0002	0.0005	0.0003	Fam85_f_m_aM_aM	0/1;0/0;0/1;0/0
chr1	32332201	32332201	A	G	HDAC1	exonic	nonsynonymous SNV	HDAC1:NM_004964:exon12:c.A1331G:p.K444R	.	1.596	.	0.0003	6.98E-06	Fam116_f_m_aM_aF	0/0;0/1;0/0;0/1
chr1	32593092	32593092	C	T	ZBTB8A	exonic	nonsynonymous SNV	ZBTB8A:NM_001040441:exon3:c.C161T:p.S54F,ZBTB8A:NM_001291496:exon3:c.C161T:p.S54F	.	1.219	.	.	.	Fam14_f_m_aM_aM	0/0;0/1;0/0;0/1
chr1	32668329	32668329	A	G	RBBP4	exonic	nonsynonymous SNV	RBBP4:NM_001135255:exon4:c.A412G:p.I138V,RBBP4:NM_001135256:exon4:c.A310G:p.I104V,RBBP4:NM_005610:exon4:c.A415G:p.I139V	.	1.33	.	6.17E-05	6.98E-06	Fam16_f_m_aM_aM	0/1;0/0;0/1;0/0
chr1	33307028	33307028	T	C	A3GALT2	exonic	nonsynonymous SNV	A3GALT2:NM_001080438:exon5:c.A761G:p.H254R	.	2.898	.	.	.	Fam97_f_m_aM_aF	0/1;0/0;0/0;0/1
chr1	33332398	33332398	G	A	PHC2	exonic	nonsynonymous SNV	PHC2:NM_004427:exon3:c.C160T:p.R54C,PHC2:NM_001330488:exon9:c.C1681T:p.R561C,PHC2:NM_198040:exon10:c.C1765T:p.R589C	.	1.346	.	3.28E-05	6.98E-06	Fam85_f_m_aM_aM	0/1;0/0;0/1;0/0
chr1	34868751	34868751	C	T	DLGAP3	exonic	nonsynonymous SNV	DLGAP3:NM_001080418:exon9:c.G2339A:p.R780H	.	1.748	.	3.49E-05	6.98E-06	Fam105_f_m_aM	0/0;0/1;0/1
chr1	34868821	34868821	C	G	DLGAP3	exonic	nonsynonymous SNV	DLGAP3:NM_001080418:exon9:c.G2269C:p.G757R	.	1.716	0.0003	8.71E-05	3.49E-05	Fam28_f_m_aF_uF	0/0;0/1;0/1;0/0
chr1	35191512	35191512	C	G	SFPQ	exonic	nonsynonymous SNV	SFPQ:NM_005066:exon2:c.G846C:p.L282F	.	2.117	.	.	.	Fam76_f_m_aM_uM	0/1;0/0;0/1;0/0
chr1	35562545	35562545	G	A	NCDN	exonic	nonsynonymous SNV	NCDN:NM_001014841:exon4:c.G1246A:p.E416K,NCDN:NM_014284:exon4:c.G1297A:p.E433K,NCDN:NM_001014839:exon5:c.G1297A:p.E433K	.	1.108	.	1.12E-05	1.40E-05	Fam51_f_m_aM_uF	0/1;0/0;0/1;0/1
chr1	35563398	35563398	C	T	NCDN	exonic	nonsynonymous SNV	NCDN:NM_001014841:exon5:c.C1531T:p.L511F,NCDN:NM_014284:exon5:c.C1582T:p.L528F,NCDN:NM_001014839:exon6:c.C1582T:p.L528F	.	1.106	.	.	.	Fam19_f_m_aM	0/0;0/1;0/1
chr1	35745535	35745535	A	C	CLSPN	exonic	stopgain	CLSPN:NM_001190481:exon15:c.T2690G:p.L897X,CLSPN:NM_001330490:exon16:c.T2882G:p.L961X,CLSPN:NM_022111:exon16:c.T2882G:p.L961X	0.511	.	.	.	.	Fam57_f_m_aM_uF	0/0;0/1;0/1;0/1
chr1	35825696	35825696	G	A	AGO4	exonic	nonsynonymous SNV	AGO4:NM_017629:exon5:c.G506A:p.R169H	.	1.337	.	.	.	Fam112_f_m_aM	0/0;0/1;0/1
chr1	35841701	35841701	T	C	AGO4	exonic	nonsynonymous SNV	AGO4:NM_017629:exon15:c.T2126C:p.V709A	.	2.04	.	.	.	Fam89_f_m_aM	0/0;0/1;0/1
chr1	36013751	36013751	G	A	AGO3	exonic	nonsynonymous SNV	AGO3:NM_177422:exon8:c.G569A:p.R190Q,AGO3:NM_024852:exon10:c.G1271A:p.R424Q	.	2.166	.	1.12E-05	6.98E-06	Fam54_f_m_aM_uF	0/1;0/0;0/1;0/1
chr1	36098014	36098014	A	G	COL8A2	exonic	nonsynonymous SNV	COL8A2:NM_001294347:exon4:c.T1472C:p.L491P,COL8A2:NM_005202:exon4:c.T1667C:p.L556P	.	1.305	.	0.0001	7.68E-05	Fam51_f_m_aM_uF	0/1;0/0;0/1;0/1
chr1	36471475	36471475	C	T	CSF3R	exonic	nonsynonymous SNV	CSF3R:NM_000760:exon10:c.G1243A:p.G415R,CSF3R:NM_156039:exon10:c.G1243A:p.G415R,CSF3R:NM_172313:exon10:c.G1243A:p.G415R	.	1.013	.	4.47E-05	1.40E-05	Fam52_f_m_aM	0/0;0/1;0/1
chr1	36802012	36802012	G	A	GRIK3	exonic	nonsynonymous SNV	GRIK3:NM_000831:exon16:c.C2599T:p.R867C	.	1.772	.	0.0001	6.98E-06	Fam26_f_m_aM_uM	0/0;0/1;0/1;0/1
chr1	36817157	36817157	C	T	GRIK3	exonic	nonsynonymous SNV	GRIK3:NM_000831:exon13:c.G1994A:p.R665H	.	1.86	.	7.45E-05	1.40E-05	Fam24_f_m_aM_aM	0/1;0/0;0/1;0/0
chr1	36841894	36841894	C	T	GRIK3	exonic	nonsynonymous SNV	GRIK3:NM_000831:exon10:c.G1372A:p.G458R	.	1.831	0.0006	3.27E-05	5.58E-05	Fam119_f_m_aM_aM	0/0;0/1;0/0;0/1
chr1	37794647	37794647	C	G	MANEAL	exonic	nonsynonymous SNV	MANEAL:NM_001031740:exon1:c.C465G:p.S155R,MANEAL:NM_001113482:exon1:c.C465G:p.S155R	.	2.4	.	.	.	Fam26_f_m_aM_uM	0/1;0/0;0/1;0/0
chr1	37809012	37809012	C	T	C1orf122	exonic	nonsynonymous SNV	C1orf122:NM_001142726:exon3:c.C83T:p.P28L,C1orf122:NM_198446:exon3:c.C272T:p.P91L	.	2.054	.	1.14E-05	.	Fam43_f_m_aM	0/0;0/1;0/1

chr1	39569632	39569632	C	G	PABPC4	exonic	nonsynonymous SNV	PABPC4:NM_001135653:exon5:c.G701C:p.G234A,PABPC4:NM_001135654:exon5:c.G701C:p.G234A,PABPC4:NM_003819:exon5:c.G701C:p.G234A	.	1.569	.	.	.	Fam112_f_m_aM	0/1;0/0;0/1
chr1	39661180	39661180	C	T	NT5C1A	exonic	nonsynonymous SNV	NT5C1A:NM_032526:exon5:c.G640A:p.A214T	.	1.17	0.0001	9.80E-05	2.79E-05	Fam7_f_m_aM_aM_uM	0/1;0/0;0/0;0/1;0/1
chr1	39848085	39848085	C	T	TRIT1	exonic	nonsynonymous SNV	TRIT1:NM_001312692:exon4:c.G470A:p.R157H,TRIT1:NM_001312691:exon6:c.G716A:p.R239H,TRIT1:NM_017646:exon6:c.G716A:p.R239H	.	1.073	0.0003	6.17E-05	0.0001	Fam113_f_m_aF_raM_uF	0/0;0/1;0/1;0/0;0/1
chr1	40413039	40413039	A	T	SMAP2	exonic	nonsynonymous SNV	SMAP2:NM_001198978:exon5:c.A336T:p.K112N,SMAP2:NM_001198979:exon5:c.A411T:p.K137N,SMAP2:NM_001198980:exon5:c.A186T:p.K62N,SMAP2:NM_022733:exon5:c.A426T:p.K142N	.	1.233	.	3.29E-05	.	Fam85_f_m_aM_aM	0/0;0/1;0/1;0/1
chr1	40416183	40416183	G	A	SMAP2	exonic	nonsynonymous SNV	SMAP2:NM_001198978:exon8:c.G599A:p.G200D,SMAP2:NM_001198979:exon8:c.G674A:p.G225D,SMAP2:NM_001198980:exon8:c.G449A:p.G150D,SMAP2:NM_022733:exon8:c.G689A:p.G230D	.	1.171	.	.	.	Fam55_f_m_aM_aM_dM	0/0;0/1;0/1;0/0;0/0
chr1	41010238	41010238	A	G	CTPS1	exonic	nonsynonymous SNV	CTPS1:NM_001301237:exon14:c.A1301G:p.H434R,CTPS1:NM_001905:exon18:c.A1769G:p.H590R	.	1.14	7.34E-05	6.57E-05	2.79E-05	Fam77_f_m_aM_aF_uM_uFs	0/1;0/0;0/0;0/1;0/0;0/0
chr1	42928972	42928972	G	A	SLC2A1	exonic	nonsynonymous SNV	SLC2A1:NM_006516:exon8:c.C1034T:p.A345V	.	2.031	.	0.0004	4.19E-05	Fam121_f_m_aF_aM	0/1;0/0;0/1;0/0
chr1	43307256	43307256	G	C	TIE1	exonic	nonsynonymous SNV	TIE1:NM_001253357:exon5:c.G620C:p.G207A,TIE1:NM_005424:exon5:c.G755C:p.G252A	.	1.119	.	2.24E-05	1.40E-05	Fam2_f_m_aF	0/0;0/1;0/1
chr1	43313257	43313257	G	C	TIE1	exonic	nonsynonymous SNV	TIE1:NM_001253357:exon13:c.G1915C:p.A639P,TIE1:NM_005424:exon13:c.G2050C:p.A684P	.	1.201	.	5.59E-05	2.79E-05	Fam88_f_m_aF	0/1;0/0;0/1
chr1	43317663	43317663	G	A	TIE1	exonic	nonsynonymous SNV	TIE1:NM_001253357:exon16:c.G2585A:p.C862Y,TIE1:NM_005424:exon16:c.G2720A:p.C907Y	.	1.387	.	1.23E-05	.	Fam56_f_m_aF_aM	0/1;0/0;0/1;0/1
chr1	43359267	43359267	G	A	CDC20	exonic	nonsynonymous SNV	CDC20:NM_001255:exon2:c.G52A:p.A18T	.	1.168	.	.	.	Fam36_f_m_aM_uM	0/0;0/1;0/1;0/0
chr1	43435330	43435330	G	A	SZT2	splicing	.	.	0.582	.	.	.	.	Fam76_f_m_aM_uM	0/1;0/0;0/1;0/0
chr1	43591218	43591218	C	T	PTPRF	exonic	nonsynonymous SNV	PTPRF:NM_001329139:exon9:c.C1196T:p.P399L,PTPRF:NM_001329140:exon9:c.C1196T:p.P399L,PTPRF:NM_002840:exon9:c.C1196T:p.P399L,PTPRF:NM_130440:exon9:c.C1196T:p.P399L,PTPRF:NM_001329137:exon11:c.C1214T:p.P405L,PTPRF:NM_001329138:exon12:c.C1226T:p.P409L	.	1.038	0.0002	0.0003	0.0003	Fam105_f_m_aM	0/0;0/1;0/1
chr1	43981392	43981392	G	A	B4GALT2	exonic	nonsynonymous SNV	B4GALT2:NM_001005417:exon2:c.G232A:p.G78R,B4GALT2:NM_003780:exon2:c.G232A:p.G78R,B4GALT2:NM_030587:exon2:c.G319A:p.G107R	.	1.066	0.0007	0.0007	0.0004	Fam81_f_m_aM_uM	0/0;0/1;0/1;0/1
chr1	44218615	44218615	C	T	DMAP1	exonic	nonsynonymous SNV	DMAP1:NM_019100:exon5:c.C580T:p.R194W,DMAP1:NM_001034023:exon6:c.C580T:p.R194W,DMAP1:NM_001034024:exon6:c.C580T:p.R194W	.	1.361	.	6.17E-05	2.09E-05	Fam81_f_m_aM_uM	0/0;0/1;0/1;0/1
chr1	44786159	44786159	G	C	BEST4	exonic	nonsynonymous SNV	BEST4:NM_153274:exon4:c.C551G:p.P184R	.	2.032	.	.	.	Fam60_f_m_aF	0/1;0/0;0/1
chr1	44803014	44803014	G	A	PLK3	exonic	nonsynonymous SNV	PLK3:NM_004073:exon7:c.G809A:p.R270H	.	1.345	.	0.0001	1.40E-05	Fam74_f_m_aM_uM	0/0;0/1;0/1;0/1
chr1	45005999	45005999	G	C	HECTD3	exonic	nonsynonymous SNV	HECTD3:NM_024602:exon14:c.C1843G:p.L615V	.	1.303	.	1.12E-05	.	Fam97_f_m_aM_aF	0/1;0/0;0/1;0/0
chr1	46023803	46023803	C	T	MAST2	exonic	nonsynonymous SNV	MAST2:NM_001319245:exon15:c.C1603T:p.R535W,MAST2:NM_001324321:exon15:c.C1141T:p.R381W,MAST2:NM_015112:exon15:c.C1603T:p.R535W,MAST2:NM_001324320:exon16:c.C1624T:p.R542W	.	2.121	0.0001	0.0002	6.99E-05	Fam91_m_aM_dM_aM_dM	0/0;0/1;0/1;0/0;0/1
chr1	46023899	46023899	G	A	MAST2	exonic	nonsynonymous SNV	MAST2:NM_001319245:exon15:c.G1699A:p.A567T,MAST2:NM_001324321:exon15:c.G1237A:p.A413T,MAST2:NM_015112:exon15:c.G1699A:p.A567T,MAST2:NM_001324320:exon16:c.G1720A:p.A574T	.	1.419	.	1.12E-05	.	Fam91_m_aM_dM_aM_dM	0/0;0/1;0/1;0/0;0/1
chr1	46035582	46035582	C	-	MAST2	exonic	frameshift deletion	MAST2:NM_001319245:exon29:c.4910delC:p.T1639Pfs*14,MAST2:NM_001324321:exon29:c.4451delC:p.T1486Pfs*14,MAST2:NM_015112:exon29:c.4913delC:p.T1640Pfs*14,MAST2:NM_001324320:exon30:c.4934delC:p.T1647Pfs*14	0.576	.	.	6.57E-05	.	Fam87_f_m_aM_uM	0/1;0/0;0/1;0/1
chr1	46285824	46285824	G	A	LRRC41	exonic	nonsynonymous SNV	LRRC41:NM_006369:exon4:c.C1033T:p.P345S	.	1.393	.	1.22E-05	.	Fam56_f_m_aF_aM	0/1;0/0;0/1;0/1
chr1	47225690	47225690	C	T	TAL1	exonic	nonsynonymous SNV	TAL1:NM_001287347:exon3:c.G199A:p.G67R,TAL1:NM_001290403:exon3:c.G199A:p.G67R,TAL1:NM_001290405:exon3:c.G199A:p.G67R,TAL1:NM_001290404:exon4:c.G199A:p.G67R,TAL1:NM_003189:exon4:c.G199A:p.G67R	.	1.863	.	.	.	Fam63_f_m_aF	0/1;0/0;0/1
chr1	48759098	48759098	A	G	BEND5	exonic	nonsynonymous SNV	BEND5:NM_001349793:exon2:c.T40C:p.Y14H,BEND5:NM_001302082:exon3:c.T40C:p.Y14H,BEND5:NM_024603:exon3:c.T547C:p.Y183H,BEND5:NM_001349794:exon4:c.T40C:p.Y14H,BEND5:NM_001349795:exon5:c.T292C:p.Y98H	.	1.924	.	.	.	Fam7_f_m_aM_aM_uM	0/0;0/1;0/1;0/1;0/1
chr1	50655447	50655447	C	T	FAF1	exonic	nonsynonymous SNV	FAF1:NM_007051:exon8:c.G739A:p.D247N	.	1.027	.	6.54E-05	1.40E-05	Fam29_f_m_aF_uM	0/1;0/0;0/1;0/0
chr1	51269957	51269957	A	G	RNF11	exonic	nonsynonymous SNV	RNF11:NM_014372:exon2:c.A125G:p.E42G	.	1.231	.	.	.	Fam13_f_m_aM	0/1;0/0;0/1
chr1	51288881	51288881	T	C	TTC39A	exonic	nonsynonymous SNV	TTC39A:NM_001297667:exon5:c.A299G:p.Q100R,TTC39A:NM_001297666:exon6:c.A500G:p.Q167R,TTC39A:NM_001080494:exon17:c.A1571G:p.Q524R,TTC39A:NM_001144832:exon17:c.A1580G:p.Q527R,TTC39A:NM_001297663:exon17:c.A1568G:p.Q523R,TTC39A:NM_001297664:exon17:c.A1487G:p.Q496R,TTC39A:NM_001297665:exon17:c.A1676G:p.Q559R	.	1.046	.	.	.	Fam94_f_m_aM	0/1;0/0;0/1
chr1	51834152	51834152	A	C	NRDC	exonic	nonsynonymous SNV	NRDC:NM_001101662:exon4:c.T731G:p.L244W,NRDC:NM_001242361:exon6:c.T539G:p.L180W,NRDC:NM_002525:exon6:c.T935G:p.L312W	.	1.865	0.0002	0.0006	0.0003	Fam28_f_m_aF_uF	0/0;0/1;0/1;0/1
chr1	52033586	52033586	C	T	KTI12	exonic	nonsynonymous SNV	KTI12:NM_138417:exon1:c.G176A:p.R59H	.	1.692	.	.	.	Fam16_f_m_aM_aM	0/0;0/0;0/0;0/1

chr1	52340227	52340227	C	G	ZFYVE9	exonic	nonsynonymous SNV	ZFYVE9:NM_007324:exon16:c.C3758G:p.T1253R,ZFYVE9:NM_004799:exon17:c.C3935G:p.T1312R	.	1.765	.	1.12E-05	.	Fam77_f_m_aM_aF_uM_uFs	0/1;0/0;0/1;0/0;0/1;0/0
chr1	53080870	53080870	T	C	PODN	exonic	nonsynonymous SNV	PODN:NM_153703:exon9:c.T1799C:p.F600S,PODN:NM_001199081:exon10:c.T1742C:p.F581S,PODN:NM_001199080:exon11:c.T1742C:p.F581S	.	1.499	7.35E-05	0.0002	9.77E-05	Fam38_f_m_aM	0/1;0/0;0/1
chr1	53257382	53257383	GG	-	LRP8	exonic	frameshift deletion	LRP8:NM_033300:exon13:c.1781_1782del:p.T594Sfs*25,LRP8:NM_001018054:exon15:c.2291_2292del:p.T764Sfs*25,LRP8:NM_004631:exon15:c.2291_2292del:p.T764Sfs*25	0.525	.	.	.	.	Fam77_f_m_aM_aF_uM_uFs	0/1;0/0;0/1;0/0;0/1;0/0
chr1	53271034	53271034	C	A	LRP8	exonic	nonsynonymous SNV	LRP8:NM_033300:exon6:c.G736T:p.A246S,LRP8:NM_017522:exon7:c.G859T:p.A287S,LRP8:NM_001018054:exon8:c.G1246T:p.A416S,LRP8:NM_004631:exon8:c.G1246T:p.A416S	.	1.126	7.34E-05	0.0002	6.29E-05	Fam84_f_m_aF	0/1;0/0;0/1
chr1	53327842	53327851	AGCA	-	LRP8	exonic	frameshift deletion	LRP8:NM_001018054:exon1:c.62_71del:p.L21Rfs*50,LRP8:NM_004631:exon1:c.62_71del:p.L21Rfs*50,LRP8:NM_017522:exon1:c.62_71del:p.L21Rfs*50,LRP8:NM_033300:exon1:c.62_71del:p.L21Rfs*50	0.525	.	0.0003	0.0006	0.0002	Fam108_f_m_aM	0/0;0/1;0/1
chr1	54782432	54782432	C	A	TTC22	exonic	nonsynonymous SNV	TTC22:NM_001114108:exon6:c.G1066T:p.G356C	.	1.019	0.0001	0.0001	0.0002	Fam108_f_m_aM	0/1;0/0;0/1
chr1	54871547	54871547	C	T	DHCR24	exonic	nonsynonymous SNV	DHCR24:NM_014762:exon5:c.G679A:p.A227T	.	1.174	7.35E-05	0.0002	0.0001	Fam71_f_m_aF	0/1;0/0;0/1
chr1	56537035	56537035	T	C	PLPP3	exonic	nonsynonymous SNV	PLPP3:NM_003713:exon2:c.A217G:p.I73V	.	1.154	.	.	.	Fam85_f_m_aM_aM	0/1;0/0;0/1;0/0
chr1	56696021	56696021	A	T	PRKAA2	exonic	nonsynonymous SNV	PRKAA2:NM_006252:exon6:c.A650T:p.E217V	.	1.311	0.0003	0.0003	0.0001	Fam20_f_m_aM_uF	0/1;0/0;0/1;0/1
chr1	61406578	61406578	A	C	NFIA	exonic	nonsynonymous SNV	NFIA:NM_001134673:exon9:c.A1271C:p.Q424P,NFIA:NM_001145511:exon9:c.A1247C:p.Q416P,NFIA:NM_005595:exon9:c.A1271C:p.Q424P,NFIA:NM_001145512:exon10:c.A1406C:p.Q469P	.	2.114	.	.	0	Fam113_f_m_aF_raM_uF	0/0;0/0;0/0;0/1;0/0
chr1	63415884	63415884	C	T	ALG6	exonic	nonsynonymous SNV	ALG6:NM_013339:exon11:c.C914T:p.T305M	.	1.006	7.36E-05	6.58E-05	1.40E-05	Fam57_f_m_aM_uF	0/1;0/0;0/1;0/0
chr1	64845593	64845593	T	G	JAK1	exonic	nonsynonymous SNV	JAK1:NM_001321852:exon15:c.A2035C:p.M679L,JAK1:NM_001321856:exon15:c.A2035C:p.M679L,JAK1:NM_001321857:exon15:c.A2032C:p.M678L,JAK1:NM_002227:exon15:c.A2035C:p.M679L,JAK1:NM_001320923:exon16:c.A2035C:p.M679L,JAK1:NM_001321854:exon16:c.A2035C:p.M679L,JAK1:NM_001321855:exon16:c.A2035C:p.M679L,JAK1:NM_001321853:exon17:c.A2035C:p.M679L	.	1.364	.	5.61E-05	1.40E-05	Fam117_f_m_aM_aF	0/0;0/1;0/0;0/1
chr1	65190745	65190745	A	T	AK4	exonic	nonsynonymous SNV	AK4:NM_001330616:exon2:c.A25T:p.S9C,AK4:NM_013410:exon2:c.A181T:p.S61C,AK4:NM_001005353:exon3:c.A181T:p.S61C,AK4:NM_203464:exon3:c.A181T:p.S61C	.	1.251	7.35E-05	3.35E-05	6.98E-06	Fam42_f_m_aM_uF	0/0;0/1;0/1;0/0
chr1	70354051	70354051	C	T	ANKRD13C	exonic	nonsynonymous SNV	ANKRD13C:NM_030816:exon1:c.G358A:p.V120I	.	1.141	0.0009	0.0006	0.0002	Fam46_f_m_aM_uM	0/1;0/0;0/1;0/0
chr1	75791527	75791527	G	A	RABGGTB	exonic	nonsynonymous SNV	RABGGTB:NM_004582:exon6:c.G535A:p.G179S	.	1.84	.	3.28E-05	1.40E-05	Fam50_f_m_aF	0/0;0/1;0/1
chr1	77063063	77063063	C	T	ST6GALNAC5	exonic	nonsynonymous SNV	ST6GALNAC5:NM_030965:exon5:c.C868T:p.R290C	.	1.286	0.0001	6.17E-05	6.99E-05	Fam34_f_m_aM_uF	0/0;0/1;0/1;0/0
chr1	83890439	83890439	G	A	TTLL7	exonic	nonsynonymous SNV	TTLL7:NM_001350215:exon18:c.C2170T:p.R724C,TTLL7:NM_024686:exon19:c.C2251T:p.R751C,TTLL7:NM_001350214:exon20:c.C2251T:p.R751C	.	1.603	0.0006	0.0002	0.0001	Fam10_f_m_aM_uF	0/1;0/0;0/1;0/0
chr1	83917606	83917606	T	C	TTLL7	exonic	nonsynonymous SNV	TTLL7:NM_001350215:exon13:c.A1504G:p.K502E,TTLL7:NM_024686:exon14:c.A1585G:p.K529E,TTLL7:NM_001350214:exon15:c.A1585G:p.K529E	.	1.448	.	.	.	Fam38_f_m_aM	0/0;0/1;0/1
chr1	85582508	85582508	T	C	CCN1	exonic	nonsynonymous SNV	CCN1:NM_001554:exon4:c.T727C:p.C243R	.	1.324	.	1.12E-05	.	Fam56_f_m_aF_aM	0/1;0/0;0/1;0/1
chr1	87332097	87332097	G	A	LMO4	exonic	nonsynonymous SNV	LMO4:NM_001369491:exon2:c.G82A:p.G28S,LMO4:NM_006769:exon2:c.G82A:p.G28S	.	2.181	.	1.13E-05	.	Fam24_f_m_aM_aM	0/0;0/1;0/1;0/0
chr1	89712973	89712973	A	G	LRRC8C	exonic	nonsynonymous SNV	LRRC8C:NM_032270:exon3:c.A403G:p.T135A	.	1.349	.	.	.	Fam47_f_m_aM	0/0;0/1;0/1
chr1	92841780	92841780	A	G	RPL5	exonic	nonsynonymous SNV	RPL5:NM_000969:exon8:c.A809G:p.K270R	.	1.142	.	1.13E-05	6.98E-06	Fam60_f_m_aF	0/1;0/0;0/1
chr1	93533006	93533006	C	A	FNBP1L	exonic	nonsynonymous SNV	FNBP1L:NM_001024948:exon8:c.C724A:p.P242T,FNBP1L:NM_001164473:exon8:c.C724A:p.P242T,FNBP1L:NM_017737:exon8:c.C724A:p.P242T	.	1.226	.	.	.	Fam10_f_m_aM_uF	0/0;0/1;0/1;0/0
chr1	94897830	94897830	T	C	CNN3	exonic	nonsynonymous SNV	CNN3:NM_001286055:exon6:c.A764G:p.Q255R,CNN3:NM_001286056:exon7:c.A779G:p.Q260R,CNN3:NM_001839:exon7:c.A902G:p.Q301R	.	1.376	.	.	.	Fam9_f_m_aM_dM_uF	0/1;0/0;0/1;0/0;0/1
chr1	98953177	98953177	T	C	PLPPR5	exonic	nonsynonymous SNV	PLPPR5:NM_001010861:exon3:c.A514G:p.S172G,PLPPR5:NM_001037317:exon3:c.A514G:p.S172G	.	1.914	.	.	6.98E-06	Fam97_f_m_aM_aF	0/0;0/1;0/1;0/1
chr1	99306498	99306498	A	G	PLPPR4	exonic	nonsynonymous SNV	PLPPR4:NM_001166252:exon6:c.A1606G:p.K536E,PLPPR4:NM_014839:exon7:c.A1780G:p.K594E	.	1.055	.	.	.	Fam2_f_m_aF	0/1;0/0;0/1
chr1	100068083	100068083	C	T	MFSD14A	exonic	nonsynonymous SNV	MFSD14A:NM_033055:exon6:c.C593T:p.A198V	.	1.998	.	.	.	Fam6_f_m_aM	0/0;0/1;0/1
chr1	101239135	101239135	G	A	S1PR1	exonic	nonsynonymous SNV	S1PR1:NM_001320730:exon2:c.G151A:p.V51M,S1PR1:NM_001400:exon2:c.G151A:p.V51M	.	1.067	7.34E-05	5.58E-05	1.40E-05	Fam26_f_m_aM_uM	0/1;0/0;0/1;0/1
chr1	102962199	102962199	C	A	COL11A1	exonic	stopgain	COL11A1:NM_080630:exon38:c.G2743T:p.E915X,COL11A1:NM_001190709:exon39:c.G2974T:p.E992X,COL11A1:NM_001854:exon40:c.G3091T:p.E1031X,COL11A1:NM_080629:exon40:c.G3127T:p.E1043X	0.344	.	.	.	.	Fam14_f_m_aM_aM	0/0;0/1;0/1;0/0
chr1	109074996	109074996	A	G	TAF13	exonic	nonsynonymous SNV	TAF13:NM_005645:exon2:c.T97C:p.S33P	.	1.262	.	4.67E-05	.	Fam82_f_m_aM_uF	0/0;0/1;0/1;0/1
chr1	109107982	109107982	C	T	C1orf194	exonic	nonsynonymous SNV	C1orf194:NM_001122961:exon2:c.G101A:p.R34Q,C1orf194:NM_001245025:exon2:c.G137A:p.R46Q,C1orf194:NM_001366200:exon2:c.G131A:p.R44Q	.	1.355	0.0003	0.0002	0.0001	Fam121_f_m_aF_aM	0/0;0/1;0/1;0/1
chr1	109251694	109251694	C	T	CELSR2	exonic	nonsynonymous SNV	CELSR2:NM_001408:exon1:c.C1615T:p.R539C	.	1.393	0.0004	0.0007	0.0004	Fam4_f_m_aM	0/1;0/0;0/1
chr1	109262350	109262350	G	A	CELSR2	exonic	nonsynonymous SNV	CELSR2:NM_001408:exon6:c.G4450A:p.V1484M	.	1.168	.	0.0001	2.79E-05	Fam49_f_m_aM	0/0;0/1;0/1
chr1	109269434	109269434	C	T	CELSR2	exonic	nonsynonymous SNV	CELSR2:NM_001408:exon21:c.C6823T:p.R2275C	.	1.388	.	6.18E-05	.	Fam67_f_m_aM_uF	0/0;0/1;0/1;0/1
chr1	109603362	109603362	G	A	GNAT2	exonic	nonsynonymous SNV	GNAT2:NM_005272:exon8:c.C1057T:p.L353F	.	1.104	0.0001	0.0001	8.37E-05	Fam82_f_m_aM_uF	0/1;0/0;0/1;0/0

chr1	109668462	109668462	A	T	GSTM2	exonic	nonsynonymous SNV	GSTM2:NM_000848:exon2:c.A74T:p.D25V,GSTM2:NM_001142368:exon2:c.A74T:p.D25V	.	1.277	.	.	.	Fam75_f_m_aM	0/0;0/1;0/1
chr1	110053882	110053882	G	C	STRIP1	exonic	nonsynonymous SNV	STRIP1:NM_001270768:exon21:c.G2199C:p.K733N,STRIP1:NM_033088:exon21:c.G2484C:p.K828N	.	1.237	0.0002	0.0001	8.37E-05	Fam26_f_m_aM_uM	0/1;0/0;0/1;0/0
chr1	110223282	110223282	G	A	KCNC4	exonic	nonsynonymous SNV	KCNC4:NM_001039574:exon2:c.G997A:p.G333S,KCNC4:NM_004978:exon2:c.G997A:p.G333S	.	1.535	0.0006	0.0007	0.0004	Fam28_f_m_aF_uF	0/0;0/1;0/1;0/1
chr1	111695356	111695356	C	T	RAP1A	exonic	stopgain	RAP1A:NM_001291896:exon3:c.C73T:p.Q25X,RAP1A:NM_001370216:exon3:c.C73T:p.Q25X,RAP1A:NM_001370217:exon3:c.C73T:p.Q25X,RAP1A:NM_002884:exon3:c.C73T:p.Q25X,RAP1A:NM_001010935:exon4:c.C73T:p.Q25X	0.433	.	.	.	.	Fam69_f_m_aM	0/0;0/0;0/1
chr1	112715181	112715181	C	T	PPM1J	exonic	nonsynonymous SNV	PPM1J:NM_005167:exon1:c.G121A:p.E41K	.	1.343	.	.	.	Fam10_f_m_aM_uF	0/0;0/1;0/1;0/0
chr1	114510883	114510883	C	A	TRIM33	exonic	nonsynonymous SNV	TRIM33:NM_015906:exon1:c.G194T:p.G65V,TRIM33:NM_033020:exon1:c.G194T:p.G65V	.	1.498	.	.	.	Fam79_f_m_aM_uM	0/0;0/1;0/1;0/0
chr1	114726316	114726316	C	T	CSDE1	exonic	nonsynonymous SNV	CSDE1:NM_001242893:exon12:c.G1442A:p.R481H,CSDE1:NM_001242892:exon13:c.G1535A:p.R512H,CSDE1:NM_007158:exon13:c.G1442A:p.R481H,CSDE1:NM_001007553:exon14:c.G1535A:p.R512H,CSDE1:NM_001130523:exon14:c.G1580A:p.R527H,CSDE1:NM_001242891:exon15:c.G1673A:p.R558H	.	1.169	.	7.46E-05	2.79E-05	Fam53_f_m_aM	0/1;0/0;0/1
chr1	116388729	116388729	G	A	ATP1A1	exonic	nonsynonymous SNV	ATP1A1:NM_000701:exon6:c.G593A:p.R198Q,ATP1A1:NM_001160233:exon6:c.G593A:p.R198Q,ATP1A1:NM_001160234:exon6:c.G500A:p.R167Q	.	1.665	.	.	.	Fam78_f_m_aF_uM	0/0;0/1;0/1;0/1
chr1	116577455	116577455	G	A	IGSF3	exonic	nonsynonymous SNV	IGSF3:NM_001007237:exon11:c.C3442T:p.R1148C,IGSF3:NM_001542:exon12:c.C3502T:p.R1168C	.	1.765	.	0.0003	2.09E-05	Fam56_f_m_aF_aM	0/0;0/1;0/0;0/1
chr1	116585047	116585047	G	A	IGSF3	exonic	nonsynonymous SNV	IGSF3:NM_001007237:exon9:c.C2446T:p.R816C,IGSF3:NM_001542:exon10:c.C2506T:p.R836C	.	1.663	.	1.48E-05	.	Fam19_f_m_aM	0/0;0/1;0/1
chr1	116941990	116941990	G	T	PTGFRN	exonic	nonsynonymous SNV	PTGFRN:NM_020440:exon2:c.G325T:p.V109F	.	1.382	.	2.23E-05	.	Fam57_f_m_aM_uF	0/0;0/1;0/1;0/1
chr1	116984868	116984868	C	G	PTGFRN	exonic	nonsynonymous SNV	PTGFRN:NM_020440:exon8:c.C2356G:p.Q786E	.	1.033	0.0002	0.0004	4.89E-05	Fam118_f_m_aM_aF_uM	0/1;0/0;0/1;0/1;0/1
chr1	116984980	116984980	T	C	PTGFRN	exonic	nonsynonymous SNV	PTGFRN:NM_020440:exon8:c.T2468C:p.M823T	.	1.36	.	.	.	Fam20_f_m_aM_uF	0/0;0/1;0/1;0/1
chr1	117112400	117112400	A	G	TRIM45	exonic	nonsynonymous SNV	TRIM45:NM_001145635:exon6:c.T1594C:p.W532R,TRIM45:NM_025188:exon6:c.T1648C:p.W550R	.	1.165	7.34E-05	3.35E-05	1.40E-05	Fam85_f_m_aM_aM	0/1;0/0;0/1;0/1
chr1	119623897	119623897	A	G	ZNF697	exonic	nonsynonymous SNV	ZNF697:NM_001080470:exon3:c.T446C:p.L149P	.	1.97	.	.	.	Fam118_f_m_aM_aF_uM	0/1;0/0;0/1;0/1;0/1
chr1	149887357	149887357	G	C	HIST2H2AC	exonic	nonsynonymous SNV	HIST2H2AC:NM_003517:exon1:c.G383C:p.S128T	.	1.126	.	3.38E-05	3.49E-05	Fam42_f_m_aM_uF	0/0;0/1;0/1;0/1
chr1	149943871	149943871	C	-	OTUD7B	exonic	frameshift deletion	OTUD7B:NM_020205:exon12:c.2518delG:p.V840Cfs*10	0.526	.	.	.	.	Fam100_f_m_aF	0/1;0/0;0/1
chr1	150338280	150338280	G	A	PRPF3	exonic	nonsynonymous SNV	PRPF3:NM_004698:exon8:c.G1156A:p.E386K,PRPF3:NM_001350529:exon9:c.G751A:p.E251K	.	1.225	.	.	.	Fam22_f_m_aF_aF_uF	0/0;0/1;0/1;0/1;0/1
chr1	150471742	150471742	A	T	RPRD2	exonic	stopgain	RPRD2:NM_001297673:exon10:c.A2716T:p.K906X	0.376	0.712	.	.	.	Fam95_f_m_aM_aM_uF	0/0;0/1;0/1;0/1;0/0
chr1	150487499	150487499	G	C	TARS2	exonic	nonsynonymous SNV	TARS2:NM_001271895:exon1:c.G49C:p.A17P,TARS2:NM_001271896:exon1:c.G49C:p.A17P,TARS2:NM_025150:exon1:c.G49C:p.A17P	.	1.063	.	.	.	Fam116_f_m_aM_aF	0/1;0/0;0/1;0/0
chr1	150648506	150648506	G	A	GOLPH3L	exonic	nonsynonymous SNV	GOLPH3L:NM_018178:exon5:c.C673T:p.L225F	.	1.038	.	.	6.98E-06	Fam7_f_m_aM_aM_uM	0/0;0/1;0/1;0/1;0/0
chr1	150834584	150834584	T	C	ARNT	exonic	nonsynonymous SNV	ARNT:NM_001197325:exon7:c.A712G:p.M238V,ARNT:NM_178427:exon7:c.A712G:p.M238V,ARNT:NM_001286036:exon8:c.A757G:p.M253V,ARNT:NM_001350225:exon8:c.A754G:p.M252V,ARNT:NM_001350226:exon8:c.A751G:p.M251V,ARNT:NM_001668:exon8:c.A757G:p.M253V,ARNT:NM_001286035:exon9:c.A730G:p.M244V,ARNT:NM_001350224:exon9:c.A730G:p.M244V	.	1.195	.	.	.	Fam30_f_m_aM_uM	0/1;0/0;0/1;0/0
chr1	151047905	151047905	G	A	C1orf56	exonic	nonsynonymous SNV	C1orf56:NM_017860:exon1:c.G58A:p.A20T	.	1.504	.	.	.	Fam43_f_m_aM	0/0;0/1;0/1
chr1	151048062	151048062	C	G	C1orf56	exonic	nonsynonymous SNV	C1orf56:NM_017860:exon1:c.C215G:p.A72G	.	1.487	.	2.27E-05	.	Fam2_f_m_aF	0/1;0/0;0/1
chr1	151137041	151137041	G	A	SEMA6C	exonic	nonsynonymous SNV	SEMA6C:NM_001178062:exon10:c.C670T:p.R224C,SEMA6C:NM_001178061:exon11:c.C790T:p.R264C,SEMA6C:NM_030913:exon11:c.C790T:p.R264C	.	1.323	0.001	0.0007	0.0004	Fam72_f_m_aF_uF	0/0;0/1;0/1;0/0
chr1	151158920	151158920	C	T	TNFAIP8L2	exonic	nonsynonymous SNV	TNFAIP8L2:NM_024575:exon2:c.C223T:p.R75C	.	1.064	.	3.36E-05	2.09E-05	Fam2_f_m_aF	0/0;0/1;0/1
chr1	151158968	151158968	C	T	TNFAIP8L2	exonic	nonsynonymous SNV	TNFAIP8L2:NM_024575:exon2:c.C271T:p.R91C	.	1.071	7.34E-05	3.27E-05	1.40E-05	Fam92_f_m_aM_raM	0/0;0/1;0/1;0/0
chr1	151224262	151224262	A	G	PIP5K1A	exonic	nonsynonymous SNV	PIP5K1A:NM_001135636:exon2:c.A103G:p.R35G,PIP5K1A:NM_001135637:exon2:c.A100G:p.R34G,PIP5K1A:NM_001135638:exon2:c.A103G:p.R35G,PIP5K1A:NM_001330689:exon2:c.A103G:p.R35G,PIP5K1A:NM_003557:exon2:c.A100G:p.R34G	.	1.191	.	.	.	Fam28_f_m_aF_uF	0/1;0/0;0/1;0/0
chr1	151232657	151232657	A	G	PIP5K1A	exonic	nonsynonymous SNV	PIP5K1A:NM_001135636:exon6:c.A557G:p.H186R,PIP5K1A:NM_001135637:exon6:c.A554G:p.H185R,PIP5K1A:NM_001330689:exon6:c.A557G:p.H186R,PIP5K1A:NM_003557:exon6:c.A554G:p.H185R,PIP5K1A:NM_001135638:exon7:c.A593G:p.H198R	.	1.738	.	.	.	Fam94_f_m_aM	0/1;0/0;0/1
chr1	151662236	151662236	T	A	SNX27	exonic	nonsynonymous SNV	SNX27:NM_001330723:exon5:c.T872A:p.V291D,SNX27:NM_030918:exon5:c.T872A:p.V291D	.	1.944	.	3.36E-05	.	Fam67_f_m_aM_uF	0/1;0/0;0/1;0/0
chr1	153686655	153686655	G	A	NPR1	exonic	nonsynonymous SNV	NPR1:NM_000906:exon11:c.G1768A:p.V590M	.	1.725	0.0003	0.0005	0.0004	Fam24_f_m_aM_aM	0/0;0/1;0/0;0/1
chr1	153688089	153688089	C	A	NPR1	exonic	nonsynonymous SNV	NPR1:NM_000906:exon15:c.C2285A:p.P762H	.	1.615	.	.	.	Fam59_f_m_aF_uM	0/1;0/0;0/1;0/0
chr1	153747025	153747025	A	G	INTS3	exonic	nonsynonymous SNV	INTS3:NM_023015:exon4:c.A387G:p.I129M,INTS3:NM_001324475:exon5:c.A387G:p.I129M	.	1.283	.	0.0002	.	Fam54_f_m_aM_uF	0/1;0/0;0/1;0/1
chr1	153770242	153770242	C	A	INTS3	exonic	nonsynonymous SNV	INTS3:NM_023015:exon24:c.C2434A:p.Q812K,INTS3:NM_001324475:exon25:c.C2434A:p.Q812K	.	2.452	.	.	.	Fam7_f_m_aM_aM_uM	0/0;0/1;0/0;0/1;0/1
chr1	153773233	153773233	G	A	INTS3	exonic	nonsynonymous SNV	INTS3:NM_023015:exon30:c.G3092A:p.R1031Q,INTS3:NM_001324475:exon31:c.G3092A:p.R1031Q	.	2.305	.	7.46E-05	.	Fam99_f_m_aM_aM	0/1;0/0;0/0;0/1

chr1	154212459	154212459	C	T	C1orf43	exonic	nonsynonymous SNV	C1orf43:NM_138740:exon3:c.G350A:p.G117E,C1orf43:NM_001297717:exon4:c.G452A:p.G151E,C1orf43:NM_001297720:exon4:c.G452A:p.G151E,C1orf43:NM_001297721:exon4:c.G404A:p.G135E,C1orf43:NM_015449:exon4:c.G404A:p.G135E,C1orf43:NM_001098616:exon5:c.G506A:p.G169E,C1orf43:NM_001297718:exon5:c.G401A:p.G134E,C1orf43:NM_001297723:exon5:c.G506A:p.G169E	.	1.233	7.34E-05	2.23E-05	1.40E-05	Fam86_f_m_aF	0/0;0/1;0/1
chr1	154584918	154584918	C	T	ADAR	exonic	nonsynonymous SNV	ADAR:NM_001025107:exon15:c.G2684A:p.R895H,ADAR:NM_001111:exon15:c.G3569A:p.R1190H,ADAR:NM_001193495:exon15:c.G2684A:p.R895H,ADAR:NM_001365045:exon15:c.G3596A:p.R1199H,ADAR:NM_001365048:exon15:c.G2684A:p.R895H,ADAR:NM_015840:exon15:c.G3491A:p.R1164H,ADAR:NM_015841:exon15:c.G3434A:p.R1145H,ADAR:NM_001365046:exon16:c.G2684A:p.R895H,ADAR:NM_001365047:exon16:c.G2684A:p.R895H,ADAR:NM_001365049:exon16:c.G2606A:p.R869H	.	1.148	.	.	.	Fam89_f_m_aM	0/0;0/1;0/1
chr1	154708235	154708235	T	C	KCNN3	exonic	nonsynonymous SNV	KCNN3:NM_001365838:exon8:c.A998G:p.N333S,KCNN3:NM_002249:exon8:c.A1937G:p.N646S,KCNN3:NM_170782:exon8:c.A1022G:p.N341S,KCNN3:NM_001204087:exon9:c.A1982G:p.N661S,KCNN3:NM_001365837:exon9:c.A1043G:p.N348S	.	1.757	0.0003	7.45E-05	2.09E-05	Fam117_f_m_aM_aF	0/1;0/0;0/1;0/1
chr1	154869490	154869490	T	C	KCNN3	exonic	nonsynonymous SNV	KCNN3:NM_001204087:exon1:c.A475G:p.S159G,KCNN3:NM_002249:exon1:c.A475G:p.S159G	.	1.801	.	.	.	Fam118_f_m_aM_aF_uM	0/0;0/1;0/1;0/1;0/0
chr1	155178125	155178125	C	T	TRIM46	exonic	nonsynonymous SNV	TRIM46:NM_001282378:exon5:c.C655T:p.R219C,TRIM46:NM_001256599:exon6:c.C964T:p.R322C,TRIM46:NM_001256601:exon6:c.C994T:p.R332C,TRIM46:NM_001282379:exon6:c.C1033T:p.R345C,TRIM46:NM_025058:exon6:c.C1033T:p.R345C	.	1.26	.	8.98E-05	4.89E-05	Fam77_f_m_aM_aF_uM_uFs	0/0;0/1;0/1;0/0;0/0;0/1
chr1	155197062	155197062	C	T	THBS3	exonic	nonsynonymous SNV	THBS3:NM_001252608:exon19:c.G2291A:p.R764Q,THBS3:NM_001252607:exon20:c.G2624A:p.R875Q,THBS3:NM_007112:exon21:c.G2651A:p.R884Q	.	1.23	.	6.71E-05	1.40E-05	Fam7_f_m_aM_aM_uM	0/0;0/1;0/1;0/0;0/0
chr1	155250347	155250347	G	A	FAM189B	exonic	nonsynonymous SNV	FAM189B:NM_198264:exon6:c.C1151T:p.P384L,FAM189B:NM_001267608:exon8:c.C1385T:p.P462L,FAM189B:NM_006589:exon9:c.C1439T:p.P480L	.	1.568	0.0003	4.41E-05	6.98E-06	Fam87_f_m_aM_uM	0/0;0/1;0/1;0/0
chr1	155269499	155269499	G	A	CLK2	exonic	nonsynonymous SNV	CLK2:NM_001294338:exon3:c.C388T:p.R130C,CLK2:NM_001363704:exon3:c.C385T:p.R129C,CLK2:NM_003993:exon3:c.C388T:p.R130C	.	1.229	.	0.0004	1.40E-05	Fam6_f_m_aM	0/0;0/1;0/1
chr1	155269501	155269501	C	T	CLK2	exonic	nonsynonymous SNV	CLK2:NM_001294338:exon3:c.G386A:p.S129N,CLK2:NM_001363704:exon3:c.G383A:p.S128N,CLK2:NM_003993:exon3:c.G386A:p.S129N	.	1.153	.	.	.	Fam11_f_m_aM_uM	0/0;0/1;0/1;0/1
chr1	155349338	155349338	G	A	ASH1L	exonic	nonsynonymous SNV	ASH1L:NM_001366177:exon19:c.C7558T:p.R2520W,ASH1L:NM_018489:exon19:c.C7543T:p.R2515W	.	1.833	.	0.0001	.	Fam108_f_m_aM	0/0;0/1;0/1
chr1	155775119	155775119	G	T	GON4L	exonic	nonsynonymous SNV	GON4L:NM_001282856:exon17:c.C2233A:p.P745T,GON4L:NM_001282858:exon17:c.C2233A:p.P745T,GON4L:NM_001282860:exon17:c.C2233A:p.P745T,GON4L:NM_001282861:exon17:c.C2233A:p.P745T,GON4L:NM_032292:exon17:c.C2233A:p.P745T	.	1.826	0.0004	0.001	0.0003	Fam56_f_m_aF_aM	0/1;0/0;0/1;0/0
chr1	155904373	155904373	G	A	RIT1	exonic	nonsynonymous SNV	RIT1:NM_001256820:exon4:c.C259T:p.R87C,RIT1:NM_001256821:exon5:c.C418T:p.R140C,RIT1:NM_006912:exon5:c.C367T:p.R123C	.	1.062	.	.	2.09E-05	Fam72_f_m_aF_uF	0/0;0/1;0/1;0/0
chr1	155925668	155925668	C	G	KHDC4	exonic	nonsynonymous SNV	KHDC4:NM_014949:exon7:c.G857C:p.G286A	.	1.166	.	.	.	Fam63_f_m_aF	0/1;0/0;0/1
chr1	155963003	155963003	C	T	ARHGEF2	exonic	nonsynonymous SNV	ARHGEF2:NM_001162383:exon8:c.G905A:p.R302H,ARHGEF2:NM_001162384:exon8:c.G902A:p.R301H,ARHGEF2:NM_001350110:exon8:c.G824A:p.R275H,ARHGEF2:NM_001350111:exon8:c.G824A:p.R275H,ARHGEF2:NM_001350112:exon8:c.G851A:p.R284H,ARHGEF2:NM_004723:exon8:c.G821A:p.R274H	.	1.572	.	0.0001	2.79E-05	Fam43_f_m_aM	0/0;0/1;0/1
chr1	156011873	156011873	A	T	SSR2	exonic	nonsynonymous SNV	SSR2:NM_003145:exon5:c.T378A:p.S126R	.	1.401	.	1.12E-05	.	Fam61_f_m_aM	0/0;0/1;0/1
chr1	156114949	156114949	C	T	LMNA	exonic	nonsynonymous SNV	LMNA:NM_001282626:exon1:c.C31T:p.R11C,LMNA:NM_005572:exon1:c.C31T:p.R11C,LMNA:NM_170707:exon1:c.C31T:p.R11C,LMNA:NM_170708:exon1:c.C31T:p.R11C,LMNA:NM_001282625:exon4:c.C31T:p.R11C	.	1.988	0.0003	.	.	Fam33_f_m_aM_uM	0/0;0/1;0/1;0/0
chr1	156253492	156253492	C	T	SMG5	exonic	nonsynonymous SNV	SMG5:NM_001323614:exon16:c.G2300A:p.R767H,SMG5:NM_001323615:exon16:c.G2321A:p.R774H,SMG5:NM_015327:exon17:c.G2459A:p.R820H,SMG5:NM_001323616:exon18:c.G2261A:p.R754H,SMG5:NM_001323617:exon18:c.G2261A:p.R754H	.	1.091	.	0.0001	.	Fam1_f_m_aM	0/1;0/0;0/1
chr1	156624051	156624051	C	G	HAPLN2	exonic	nonsynonymous SNV	HAPLN2:NM_021817:exon4:c.C330G:p.D110E	.	1.81	.	9.38E-05	6.98E-06	Fam26_f_m_aM_uM	0/1;0/0;0/1;0/0
chr1	156624106	156624106	G	A	HAPLN2	exonic	nonsynonymous SNV	HAPLN2:NM_021817:exon4:c.G385A:p.E129K	.	1.848	.	.	.	Fam95_f_m_aM_aM_uF	0/1;0/0;0/1;0/1;0/0
chr1	156646888	156646888	A	G	BCAN	exonic	nonsynonymous SNV	BCAN:NM_021948:exon3:c.A179G:p.H60R,BCAN:NM_198427:exon3:c.A179G:p.H60R	.	1.398	7.37E-05	.	1.40E-05	Fam54_f_m_aM_uF	0/0;0/1;0/1;0/1
chr1	156646899	156646899	C	T	BCAN	exonic	nonsynonymous SNV	BCAN:NM_021948:exon3:c.C190T:p.P64S,BCAN:NM_198427:exon3:c.C190T:p.P64S	.	1.665	.	.	.	Fam48_f_m_aM_uM	0/1;0/0;0/1;0/0
chr1	156676960	156676960	C	G	NES	exonic	nonsynonymous SNV	NES:NM_006617:exon1:c.G305C:p.R102P	.	2.447	.	.	.	Fam78_f_m_aF_uM	0/1;0/0;0/1;0/1
chr1	156955732	156955732	A	G	ARHGEF11	exonic	nonsynonymous SNV	ARHGEF11:NM_014784:exon19:c.T1619C:p.I540T,ARHGEF11:NM_198236:exon20:c.T1739C:p.I580T	.	1.323	.	.	6.98E-06	Fam114_f_m_aM	0/0;0/1;0/1
chr1	157099189	157099189	C	T	ETV3L	exonic	nonsynonymous SNV	ETV3L:NM_001004341:exon2:c.G248A:p.R83K	.	2.022	.	3.36E-05	.	Fam98_f_m_aM	0/1;0/0;0/1
chr1	157099211	157099211	C	A	ETV3L	exonic	nonsynonymous SNV	ETV3L:NM_001004341:exon2:c.G226T:p.V76L	.	1.837	.	.	.	Fam73_f_m_aF_dM	0/0;0/1;0/1;0/0
chr1	157125614	157125614	C	T	ETV3	exonic	nonsynonymous SNV	ETV3:NM_001145312:exon5:c.G766A:p.G256R	.	2.039	.	0.0005	0.0001	Fam121_f_m_aF_aM	0/1;0/0;0/1;0/1

chr1	158076124	158076124	C	T	KIRREL1	exonic	nonsynonymous SNV	KIRREL1:NM_018240:exon2:c.C64T:p.R22C	.	1.838	.	0.0002	2.79E-05	Fam57_f_m_aM_uF	0/0;0/1;0/1;0/0
chr1	158093381	158093381	C	T	KIRREL1	exonic	nonsynonymous SNV	KIRREL1:NM_001286349:exon10:c.C1214T:p.A405V,KIRREL1:NM_018240:exon12:c.C1514T:p.A505V	.	1.075	0.0001	0.0003	6.98E-05	Fam40_f_m_aM_aM	0/1;0/0;0/0;0/1
chr1	158681526	158681526	C	A	SPTA1	splicing	.	.	0.592	.	.	.	.	Fam38_f_m_aM	0/1;0/0;0/1
chr1	159199867	159199867	C	T	CADM3	exonic	nonsynonymous SNV	CADM3:NM_001127173:exon8:c.C1069T:p.R357W,CADM3:NM_001346510:exon8:c.C931T:p.R311W,CADM3:NM_021189:exon9:c.C1171T:p.R391W	.	1.281	.	2.24E-05	1.40E-05	Fam16_f_m_aM_aM	0/0;0/1;0/1;0/0
chr1	160240062	160240062	G	A	DCAF8	exonic	nonsynonymous SNV	DCAF8:NM_015726:exon4:c.C358T:p.R120C	.	1.166	.	0.0001	6.98E-05	Fam121_f_m_aF_aM	0/1;0/0;0/1;0/1
chr1	160296140	160296140	G	C	COPA	exonic	nonsynonymous SNV	COPA:NM_001098398:exon22:c.C2300G:p.A767G,COPA:NM_004371:exon22:c.C2273G:p.A758G	.	1.363	.	.	6.98E-06	Fam66_f_m_aM	0/1;0/0;0/1
chr1	160297429	160297429	C	A	COPA	exonic	nonsynonymous SNV	COPA:NM_001098398:exon21:c.G2204T:p.R735I,COPA:NM_004371:exon21:c.G2177T:p.R726I	.	1.012	.	.	.	Fam30_f_m_aM_uM	0/1;0/0;0/1;0/0
chr1	160419212	160419212	C	T	VANGL2	exonic	nonsynonymous SNV	VANGL2:NM_020335:exon4:c.C403T:p.R135W	.	1.426	.	0.0002	4.89E-05	Fam14_f_m_aM_aM	0/1;0/0;0/1;0/1
chr1	160421109	160421109	G	A	VANGL2	exonic	nonsynonymous SNV	VANGL2:NM_020335:exon6:c.G995A:p.R332Q	.	1.417	.	.	0.0001	Fam62_f_m_aM_aM	0/0;0/1;0/1;0/1
chr1	161168559	161168559	G	C	PPOX	exonic	nonsynonymous SNV	PPOX:NM_001350128:exon5:c.G500C:p.G167A,PPOX:NM_001350131:exon5:c.G113C:p.G38A,PPOX:NM_001365400:exon5:c.G191C:p.G64A,PPOX:NM_001365401:exon5:c.G113C:p.G38A,PPOX:NM_000309:exon6:c.G599C:p.G200A,PPOX:NM_001122764:exon6:c.G599C:p.G200A,PPOX:NM_001350129:exon6:c.G191C:p.G64A,PPOX:NM_001350130:exon6:c.G113C:p.G38A,PPOX:NM_001365398:exon6:c.G599C:p.G200A,PPOX:NM_001365399:exon6:c.G599C:p.G200A	.	1.363	.	.	.	Fam5_f_m_aM	0/0;0/1;0/1
chr1	161306450	161306450	C	G	MPZ	exonic	nonsynonymous SNV	MPZ:NM_000530:exon4:c.G463C:p.G155R	.	1.645	.	.	.	Fam83_f_m_aF	0/1;0/0;0/1
chr1	161365011	161365011	G	A	CFAP126	exonic	nonsynonymous SNV	CFAP126:NM_001013625:exon5:c.C488T:p.P163L	.	1.505	.	.	.	Fam36_f_m_aM_uM	0/1;0/0;0/1;0/0
chr1	162287428	162287428	A	G	NOS1AP	exonic	nonsynonymous SNV	NOS1AP:NM_001164757:exon3:c.A262G:p.K88E,NOS1AP:NM_014697:exon3:c.A262G:p.K88E	.	2.782	.	.	.	Fam19_f_m_aM	0/0;0/1;0/1
chr1	162778688	162778688	A	G	DDR2	exonic	nonsynonymous SNV	DDR2:NM_001354982:exon17:c.A2392G:p.I798V,DDR2:NM_001354983:exon17:c.A2392G:p.I798V,DDR2:NM_006182:exon17:c.A2392G:p.I798V,DDR2:NM_001014796:exon18:c.A2392G:p.I798V	.	1.188	.	.	.	Fam65_f_m_aM_uF_dF	0/0;0/1;0/1;0/0;0/1
chr1	163343845	163343845	C	G	NUF2	exonic	nonsynonymous SNV	NUF2:NM_031423:exon10:c.C782G:p.T261R,NUF2:NM_145697:exon10:c.C782G:p.T261R	.	1.192	.	.	.	Fam44_f_m_aM_uF	0/0;0/1;0/1;0/0
chr1	163347863	163347863	A	G	NUF2	exonic	nonsynonymous SNV	NUF2:NM_031423:exon12:c.A1049G:p.E350G,NUF2:NM_145697:exon12:c.A1049G:p.E350G	.	1.062	.	.	.	Fam13_f_m_aM	0/1;0/0;0/1
chr1	166849066	166849066	G	C	POGK	exonic	nonsynonymous SNV	POGK:NM_001314014:exon3:c.G133C:p.E45Q,POGK:NM_017542:exon5:c.G487C:p.E163Q	.	1.947	.	1.12E-05	.	Fam119_f_m_aM_aM	0/1;0/0;0/1;0/0
chr1	166849985	166849985	T	C	POGK	exonic	nonsynonymous SNV	POGK:NM_001314014:exon3:c.T1052C:p.I351T,POGK:NM_017542:exon5:c.T1406C:p.I469T	.	2.164	.	.	.	Fam33_f_m_aM_uM	0/1;0/0;0/1;0/1
chr1	167415585	167415585	C	A	POU2F1	exonic	nonsynonymous SNV	POU2F1:NM_001198786:exon15:c.C1887A:p.N629K,POU2F1:NM_001198783:exon16:c.C2043A:p.N681K,POU2F1:NM_002697:exon16:c.C2076A:p.N692K,POU2F1:NM_001365849:exon17:c.C2001A:p.N667K,POU2F1:NM_001365848:exon18:c.C2001A:p.N667K	.	1.208	.	.	.	Fam61_f_m_aM	0/1;0/0;0/1
chr1	168096613	168096613	G	A	GPR161	exonic	nonsynonymous SNV	GPR161:NM_001267613:exon2:c.C760T:p.R254C,GPR161:NM_001267614:exon2:c.C652T:p.R218C,GPR161:NM_001349635:exon2:c.C598T:p.R200C,GPR161:NM_001267611:exon3:c.C1045T:p.R349C,GPR161:NM_001267612:exon3:c.C598T:p.R200C,GPR161:NM_001349633:exon3:c.C994T:p.R332C,GPR161:NM_001267609:exon4:c.C1054T:p.R352C,GPR161:NM_001267610:exon4:c.C994T:p.R332C,GPR161:NM_001349632:exon5:c.C994T:p.R332C,GPR161:NM_001349634:exon5:c.C994T:p.R332C,GPR161:NM_153832:exon5:c.C994T:p.R332C	.	1.1	.	6.55E-05	.	Fam96_f_m_aM_uF	0/1;0/0;0/1;0/1
chr1	168096853	168096853	T	A	GPR161	exonic	nonsynonymous SNV	GPR161:NM_001267613:exon2:c.A520T:p.R174W,GPR161:NM_001267614:exon2:c.A412T:p.R138W,GPR161:NM_001349635:exon2:c.A358T:p.R120W,GPR161:NM_001267611:exon3:c.A805T:p.R269W,GPR161:NM_001267612:exon3:c.A358T:p.R120W,GPR161:NM_001349633:exon3:c.A754T:p.R252W,GPR161:NM_001267609:exon4:c.A814T:p.R272W,GPR161:NM_001267610:exon4:c.A754T:p.R252W,GPR161:NM_001349632:exon5:c.A754T:p.R252W,GPR161:NM_001349634:exon5:c.A754T:p.R252W,GPR161:NM_153832:exon5:c.A754T:p.R252W	.	1.073	.	1.12E-05	6.98E-06	Fam91_m_aM_dM_aM_dM	0/1;0/1;0/1;0/1;0/1
chr1	168096969	168096969	C	T	GPR161	exonic	nonsynonymous SNV	GPR161:NM_001267613:exon2:c.G404A:p.R135H,GPR161:NM_001267614:exon2:c.G296A:p.R99H,GPR161:NM_001349635:exon2:c.G242A:p.R81H,GPR161:NM_001267611:exon3:c.G689A:p.R230H,GPR161:NM_001267612:exon3:c.G242A:p.R81H,GPR161:NM_001349633:exon3:c.G638A:p.R213H,GPR161:NM_001267609:exon4:c.G698A:p.R233H,GPR161:NM_001267610:exon4:c.G638A:p.R213H,GPR161:NM_001349632:exon5:c.G638A:p.R213H,GPR161:NM_001349634:exon5:c.G638A:p.R213H,GPR161:NM_153832:exon5:c.G638A:p.R213H	.	1.158	.	6.54E-05	2.09E-05	Fam51_f_m_aM_uF	0/1;0/0;0/1;0/1
chr1	168231591	168231591	C	G	SFT2D2	exonic	nonsynonymous SNV	SFT2D2:NM_199344:exon2:c.C141G:p.C47W	.	1.046	7.35E-05	1.12E-05	6.98E-06	Fam17_f_m_aM_uM	0/1;0/0;0/1;0/0
chr1	169111490	169111490	C	T	ATP1B1	exonic	nonsynonymous SNV	ATP1B1:NM_001677:exon2:c.C218T:p.A73V	.	1.397	.	.	2.79E-05	Fam119_f_m_aM_aM	0/1;0/0;0/1;0/0
chr1	173481390	173481390	G	A	PRDX6	exonic	nonsynonymous SNV	PRDX6:NM_004905:exon2:c.G160A:p.A54T	.	1.302	.	.	.	Fam91_m_aM_dM_aM_dM	0/0;0/1;0/1;0/0;0/1

chr1	173836959	173836959	T	A	DARS2	exonic	nonsynonymous SNV	DARS2:NM_001365212:exon8:c.T683A:p.V228E,DARS2:NM_001365213:exon8:c.T683A:p.V228E,DARS2:NM_018122:exon8:c.T683A:p.V228E	.	1.124	.	.	.	Fam117_f_m_aM_aF	0/0;0/1;0/1;0/0
chr1	173836959	173836959	T	A	DARS2	exonic	nonsynonymous SNV	DARS2:NM_001365212:exon8:c.T683A:p.V228E,DARS2:NM_001365213:exon8:c.T683A:p.V228E,DARS2:NM_018122:exon8:c.T683A:p.V228E	.	1.124	.	.	.	Fam39_f_m_aM	0/1;0/0;0/1
chr1	173914726	173914726	G	A	SERPINC1	exonic	nonsynonymous SNV	SERPINC1:NM_000488:exon2:c.C235T:p.R79C,SERPINC1:NM_001365052:exon3:c.C91T:p.R31C	.	1.331	.	0.0004	4.88E-05	Fam4_f_m_aM	0/1;0/0;0/1
chr1	174219296	174219296	G	A	RABGAP1L	splicing	.	.	0.586	.	.	9.37E-05	3.50E-05	Fam14_f_m_aM_aM	0/1;0/0;0/1;0/1
chr1	178457852	178457852	C	T	RASAL2	exonic	stopgain	RASAL2:NM_004841:exon12:c.C2137T:p.Q713X,RASAL2:NM_170692:exon14:c.C2560T:p.Q854X	0.552	.	.	.	.	Fam19_f_m_aM	0/0;0/0;0/1
chr1	180825282	180825282	T	C	XPR1	exonic	nonsynonymous SNV	XPR1:NM_001135669:exon9:c.T1072C:p.F358L,XPR1:NM_001328662:exon9:c.T1072C:p.F358L,XPR1:NM_004736:exon9:c.T1072C:p.F358L	.	1.133	.	0.0002	6.28E-05	Fam44_f_m_aM_uF	0/0;0/1;0/1;0/0
chr1	182858557	182858557	C	T	DHX9	exonic	nonsynonymous SNV	DHX9:NM_001357:exon9:c.C817T:p.P273S	.	1.965	.	.	.	Fam23_f_m_aM_dF_uFs	0/1;0/0;0/1;0/1;0/0
chr1	183560120	183560120	C	T	NCF2	exonic	nonsynonymous SNV	NCF2:NM_001190789:exon12:c.G1201A:p.D401N,NCF2:NM_001190794:exon13:c.G1309A:p.D437N,NCF2:NM_000433:exon14:c.G1444A:p.D482N,NCF2:NM_001127651:exon15:c.G1444A:p.D482N	.	1.028	.	1.12E-05	.	Fam92_f_m_aM_raM	0/0;0/0;0/0;0/1
chr1	183590226	183590226	G	A	NCF2	exonic	nonsynonymous SNV	NCF2:NM_000433:exon1:c.C104T:p.P35L,NCF2:NM_001190789:exon1:c.C104T:p.P35L,NCF2:NM_001190794:exon1:c.C104T:p.P35L,NCF2:NM_001127651:exon2:c.C104T:p.P35L	.	1.051	.	.	.	Fam43_f_m_aM	0/0;0/1;0/1
chr1	183916655	183916655	G	A	RGL1	exonic	nonsynonymous SNV	RGL1:NM_001297672:exon15:c.G1871A:p.R624H,RGL1:NM_001297670:exon16:c.G1952A:p.R651H,RGL1:NM_001297671:exon16:c.G1958A:p.R653H,RGL1:NM_001297669:exon17:c.G1952A:p.R651H,RGL1:NM_015149:exon17:c.G2063A:p.R688H	.	1.116	.	7.46E-05	6.99E-06	Fam24_f_m_aM_aM	0/1;0/0;0/1;0/0
chr1	185300308	185300308	G	C	IVNS1ABP	exonic	nonsynonymous SNV	IVNS1ABP:NM_006469:exon12:c.C1278G:p.H426Q	.	1.385	0.0003	0.0004	0.0001	Fam25_f_m_aM	0/0;0/1;0/1
chr1	190226207	190226207	C	T	BRINP3	exonic	stopgain	BRINP3:NM_001317188:exon5:c.G530A:p.W177X,BRINP3:NM_199051:exon6:c.G836A:p.W279X	0.364	.	.	.	.	Fam108_f_m_aM	0/0;0/1;0/1
chr1	200407539	200407539	A	G	ZNF281	exonic	nonsynonymous SNV	ZNF281:NM_001281293:exon2:c.T2167C:p.S723P,ZNF281:NM_012482:exon2:c.T2167C:p.S723P,ZNF281:NM_001281294:exon3:c.T2059C:p.S687P	.	1.616	.	.	.	Fam85_f_m_aM_aM	0/0;0/1;0/1;0/1
chr1	200739961	200739961	G	A	CAMSAP2	exonic	nonsynonymous SNV	CAMSAP2:NM_001297707:exon1:c.G134A:p.G45E,CAMSAP2:NM_001297708:exon1:c.G134A:p.G45E,CAMSAP2:NM_203459:exon1:c.G134A:p.G45E	.	1.132	.	2.23E-05	.	Fam47_f_m_aM	0/1;0/0;0/1
chr1	200832336	200832336	T	C	CAMSAP2	exonic	nonsynonymous SNV	CAMSAP2:NM_001297708:exon5:c.T782C:p.L261S,CAMSAP2:NM_203459:exon5:c.T782C:p.L261S,CAMSAP2:NM_001297707:exon6:c.T815C:p.L272S	.	1.128	0.0004	0.0002	0.0002	Fam62_f_m_aM_aM	0/0;0/1;0/1;0/1
chr1	200979556	200979556	G	A	KIF21B	exonic	nonsynonymous SNV	KIF21B:NM_001252103:exon29:c.C4100T:p.A1367V,KIF21B:NM_017596:exon29:c.C4100T:p.A1367V,KIF21B:NM_001252100:exon30:c.C4139T:p.A1380V,KIF21B:NM_001252102:exon30:c.C4139T:p.A1380V	.	1.41	.	.	1.40E-05	Fam34_f_m_aM_uF	0/1;0/0;0/1;0/0
chr1	201135809	201135809	G	A	TMEM9	exonic	nonsynonymous SNV	TMEM9:NM_001288565:exon5:c.C406T:p.R136C,TMEM9:NM_001288564:exon6:c.C406T:p.R136C,TMEM9:NM_001288566:exon6:c.C406T:p.R136C,TMEM9:NM_001288567:exon6:c.C406T:p.R136C,TMEM9:NM_001288568:exon6:c.C406T:p.R136C,TMEM9:NM_001288569:exon6:c.C406T:p.R136C,TMEM9:NM_001288570:exon6:c.C415T:p.R139C,TMEM9:NM_001288571:exon6:c.C481T:p.R161C,TMEM9:NM_016456:exon6:c.C406T:p.R136C	.	1.293	7.35E-05	7.08E-05	1.40E-05	Fam11_f_m_aM_uM	0/0;0/1;0/1;0/1
chr1	201359245	201359245	G	A	TNNT2	exonic	nonsynonymous SNV	TNNT2:NM_001001432:exon14:c.C814T:p.R272C,TNNT2:NM_001001431:exon15:c.C823T:p.R275C,TNNT2:NM_001276346:exon15:c.C733T:p.R245C,TNNT2:NM_000364:exon16:c.C853T:p.R285C,TNNT2:NM_001001430:exon16:c.C832T:p.R278C,TNNT2:NM_001276347:exon16:c.C832T:p.R278C,TNNT2:NM_001276345:exon17:c.C862T:p.R288C	.	1.675	0.0005	0.0007	0.0004	Fam31_f_m_aM_uF	0/1;0/0;0/1;0/0
chr1	201648708	201648708	G	C	NAV1	exonic	nonsynonymous SNV	NAV1:NM_020443:exon1:c.G40C:p.E14Q	.	1.656	0.0001	.	1.40E-05	Fam105_f_m_aM	0/0;0/1;0/1
chr1	201648979	201648979	T	C	NAV1	exonic	nonsynonymous SNV	NAV1:NM_020443:exon1:c.T311C:p.L104P	.	2.339	.	.	.	Fam12_f_m_aM_uM_aM	0/0;0/1;0/0;0/1;0/1
chr1	201788549	201788549	G	T	NAV1	exonic	nonsynonymous SNV	NAV1:NM_001167738:exon8:c.G1904T:p.S635I,NAV1:NM_020443:exon10:c.G3077T:p.S1026I	.	1.014	.	8.94E-05	3.49E-05	Fam25_f_m_aM	0/1;0/0;0/1
chr1	202280797	202280797	C	T	LGR6	exonic	nonsynonymous SNV	LGR6:NM_001017403:exon6:c.C661T:p.R221C,LGR6:NM_021636:exon6:c.C505T:p.R169C	.	1.001	.	7.46E-05	.	Fam37_f_m_aF_uM	0/0;0/1;0/1;0/0
chr1	202318462	202318462	A	G	LGR6	exonic	nonsynonymous SNV	LGR6:NM_001017404:exon16:c.A1742G:p.Y581C,LGR6:NM_001017403:exon18:c.A2159G:p.Y720C,LGR6:NM_021636:exon18:c.A2003G:p.Y668C	.	1.018	.	.	.	Fam91_m_aM_dM_aM_dM	0/0;0/1;0/1;0/0;0/1
chr1	202919843	202919843	T	G	KLHL12	exonic	nonsynonymous SNV	KLHL12:NM_001303051:exon3:c.A375C:p.L125F,KLHL12:NM_001303109:exon3:c.A261C:p.L87F,KLHL12:NM_021633:exon3:c.A261C:p.L87F	.	1.82	.	.	.	Fam97_f_m_aM_aF	0/0;0/1;0/1;0/1
chr1	203022942	203022942	C	T	TMEM183A	exonic	nonsynonymous SNV	TMEM183A:NM_001322958:exon7:c.C943T:p.R315C,TMEM183A:NM_001322959:exon7:c.C940T:p.R314C,TMEM183A:NM_001349859:exon7:c.C736T:p.R246C,TMEM183A:NM_001349862:exon7:c.C733T:p.R245C,TMEM183A:NM_001322955:exon8:c.C1022T:p.A341V,TMEM183A:NM_001322956:exon8:c.C1030T:p.R344C,TMEM183A:NM_001322957:exon8:c.C1025T:p.A342V,TMEM183A:NM_138391:exon8:c.C1033T:p.R345C	.	2.372	.	9.83E-05	7.02E-06	Fam96_f_m_aM_uF	0/1;0/0;0/1;0/1
chr1	203060284	203060284	T	C	PPFIA4	exonic	nonsynonymous SNV	PPFIA4:NM_001304331:exon22:c.T2651C:p.M884T,PPFIA4:NM_001304332:exon22:c.T2651C:p.M884T	.	1.417	.	.	.	Fam54_f_m_aM_uF	0/0;0/1;0/1;0/0

chr1	203700266	203700266	G	C	ATP2B4	exonic	nonsynonymous SNV	ATP2B4:NM_001001396:exon5:c.G710C:p.S237T,ATP2B4:NM_001365783:exon5:c.G710C:p.S237T,ATP2B4:NM_001365784:exon5:c.G710C:p.S237T,ATP2B4:NM_001684:exon5:c.G710C:p.S237T	.	1.229	.	.	.	Fam71_f_m_aF	0/0;0/1;0/1
chr1	203700809	203700809	A	G	ATP2B4	exonic	nonsynonymous SNV	ATP2B4:NM_001001396:exon6:c.A787G:p.M263V,ATP2B4:NM_001365783:exon6:c.A787G:p.M263V,ATP2B4:NM_001365784:exon6:c.A787G:p.M263V,ATP2B4:NM_001684:exon6:c.A787G:p.M263V	.	1.29	.	1.12E-05	.	Fam44_f_m_aM_uF	0/1;0/0;0/1;0/0
chr1	204449901	204449901	C	T	PIK3C2B	exonic	nonsynonymous SNV	PIK3C2B:NM_002646:exon14:c.G2183A:p.R728Q	.	1.072	.	0.0001	4.89E-05	Fam34_f_m_aM_uF	0/0;0/1;0/1;0/1
chr1	204988708	204988708	C	T	NFASC	exonic	nonsynonymous SNV	NFASC:NM_001365986:exon22:c.C2657T:p.T886M,NFASC:NM_001005388:exon23:c.C2669T:p.T890M,NFASC:NM_001160331:exon23:c.C3023T:p.T1008M,NFASC:NM_001160332:exon24:c.C2978T:p.T993M,NFASC:NM_015090:exon24:c.C2978T:p.T993M	.	1.024	7.34E-05	6.17E-05	2.09E-05	Fam94_f_m_aM	0/1;0/0;0/1
chr1	205061970	205061970	G	A	CNTN2	exonic	stopgain	CNTN2:NM_001346083:exon9:c.G1079A:p.W360X,CNTN2:NM_005076:exon9:c.G1079A:p.W360X	0.542	.	.	.	.	Fam81_f_m_aM_uM	0/1;0/0;0/1;0/0
chr1	205211501	205211501	G	A	DSTYK	exonic	nonsynonymous SNV	DSTYK:NM_015375:exon1:c.C35T:p.P12L,DSTYK:NM_199462:exon1:c.C35T:p.P12L	.	1.141	0.0002	0.0002	4.89E-05	Fam107_f_m_aM	0/0;0/1;0/1
chr1	205269052	205269052	C	G	TMCC2	exonic	nonsynonymous SNV	TMCC2:NM_001297611:exon2:c.C175G:p.R59G,TMCC2:NM_001297613:exon2:c.C130G:p.R44G,TMCC2:NM_001331034:exon2:c.C265G:p.R89G,TMCC2:NM_001242925:exon3:c.C616G:p.R206G,TMCC2:NM_014858:exon3:c.C850G:p.R284G	.	1.756	.	.	.	Fam5_f_m_aM	0/0;0/1;0/1
chr1	205343543	205343543	C	T	KLHDC8A	exonic	nonsynonymous SNV	KLHDC8A:NM_018203:exon2:c.G62A:p.R21Q,KLHDC8A:NM_001271864:exon3:c.G62A:p.R21Q,KLHDC8A:NM_001271865:exon4:c.G62A:p.R21Q,KLHDC8A:NM_001271863:exon5:c.G62A:p.R21Q	.	1.386	.	.	.	Fam103_f_m_aM	0/1;0/0;0/1
chr1	205599225	205599225	A	G	MFSD4A	exonic	nonsynonymous SNV	MFSD4A:NM_181644:exon9:c.A1463G:p.H488R	.	1.068	.	0.0003	4.19E-05	Fam11_f_m_aM_uM	0/1;0/0;0/1;0/0
chr1	205663534	205663534	C	T	SLC45A3	exonic	nonsynonymous SNV	SLC45A3:NM_033102:exon3:c.G257A:p.R86Q	.	1.223	.	1.16E-05	.	Fam115_f_m_aF_aM_aF	0/1;0/0;0/1;0/0;0/1
chr1	205791709	205791709	G	C	SLC41A1	exonic	nonsynonymous SNV	SLC41A1:NM_173854:exon11:c.C1366G:p.L456V	.	1.014	7.35E-05	0.0001	4.89E-05	Fam82_f_m_aM_uF	0/0;0/1;0/1;0/0
chr1	205798975	205798975	T	C	SLC41A1	exonic	nonsynonymous SNV	SLC41A1:NM_173854:exon5:c.A679G:p.I227V	.	1.01	.	7.46E-05	6.99E-06	Fam22_f_m_aF_aF_uF	0/1;0/0;0/1;0/1;0/1
chr1	206595791	206595791	T	C	EIF2D	exonic	nonsynonymous SNV	EIF2D:NM_001201478:exon11:c.A1064G:p.Q355R,EIF2D:NM_006893:exon13:c.A1436G:p.Q479R	.	1.086	.	.	1.40E-05	Fam44_f_m_aM_uF	0/1;0/0;0/1;0/0
chr1	206934647	206934647	T	G	PIGR	exonic	nonsynonymous SNV	PIGR:NM_002644:exon6:c.A1478C:p.K493T	.	1.128	.	.	.	Fam48_f_m_aM_uM	0/0;0/1;0/1;0/1
chr1	207072267	207072267	A	G	PFKFB2	exonic	nonsynonymous SNV	PFKFB2:NM_006212:exon15:c.A1414G:p.S472G	.	1.043	.	4.47E-05	.	Fam100_f_m_aF	0/1;0/0;0/1
chr1	211271253	211271253	A	G	RCOR3	exonic	nonsynonymous SNV	RCOR3:NM_018254:exon2:c.A71G:p.K24R,RCOR3:NM_001136223:exon3:c.A245G:p.K82R,RCOR3:NM_001136224:exon3:c.A245G:p.K82R,RCOR3:NM_001136225:exon3:c.A245G:p.K82R,RCOR3:NM_001350069:exon4:c.A341G:p.K114R	.	1.714	.	.	.	Fam34_f_m_aM_uF	0/1;0/0;0/1;0/0
chr1	212348492	212348492	G	A	PPP2R5A	exonic	nonsynonymous SNV	PPP2R5A:NM_001199756:exon7:c.G697A:p.A233T,PPP2R5A:NM_006243:exon7:c.G868A:p.A290T	.	1.686	.	4.57E-05	.	Fam18_f_m_aM_uF	0/0;0/1;0/1;0/1
chr1	212859084	212859084	C	T	FLVCR1	exonic	nonsynonymous SNV	FLVCR1:NM_014053:exon1:c.C632T:p.S211L	.	1.804	.	.	.	Fam47_f_m_aM	0/0;0/1;0/1
chr1	213998177	213998177	G	A	PROX1	exonic	nonsynonymous SNV	PROX1:NM_001270616:exon2:c.G1642A:p.E548K,PROX1:NM_002763:exon2:c.G1642A:p.E548K	.	1.5	.	.	.	Fam119_f_m_aM_aM	0/0;0/1;0/1;0/1
chr1	215086460	215086460	C	T	KCNK2	exonic	nonsynonymous SNV	KCNK2:NM_001017424:exon2:c.C127T:p.R43W,KCNK2:NM_001017425:exon2:c.C139T:p.R47W,KCNK2:NM_014217:exon2:c.C94T:p.R32W	.	1.575	.	.	.	Fam25_f_m_aM	0/0;0/1;0/1
chr1	215604162	215604162	A	G	KCTD3	exonic	nonsynonymous SNV	KCTD3:NM_001319294:exon13:c.A1169G:p.Y390C,KCTD3:NM_001319295:exon13:c.A863G:p.Y288C,KCTD3:NM_016121:exon13:c.A1169G:p.Y390C	.	2.087	.	.	.	Fam103_f_m_aM	0/0;0/1;0/1
chr1	218405222	218405222	G	A	TGFB2	exonic	nonsynonymous SNV	TGFB2:NM_003238:exon2:c.G400A:p.V134I,TGFB2:NM_001135599:exon3:c.G484A:p.V162I	.	1.575	7.34E-05	3.35E-05	2.79E-05	Fam25_f_m_aM	0/1;0/0;0/1
chr1	219928172	219928172	G	A	SLC30A10	exonic	nonsynonymous SNV	SLC30A10:NM_018713:exon1:c.C269T:p.T90I	.	1.756	.	0.001	0.0002	Fam106_f_m_aM	0/1;0/0;0/1
chr1	220074002	220074002	G	A	BPNT1	exonic	nonsynonymous SNV	BPNT1:NM_001286149:exon2:c.C25T:p.R9W,BPNT1:NM_001286151:exon2:c.C25T:p.R9W,BPNT1:NM_001286150:exon3:c.C190T:p.R64W,BPNT1:NM_006085:exon3:c.C190T:p.R64W	.	1.024	0.0003	0.0005	0.0002	Fam63_f_m_aF	0/1;0/0;0/1
chr1	220696723	220696723	G	A	C1orf115	exonic	nonsynonymous SNV	C1orf115:NM_024709:exon2:c.G421A:p.V141M	.	1.032	.	0.0004	5.58E-05	Fam57_f_m_aM_uF	0/1;0/0;0/1;0/0
chr1	220880356	220880356	C	T	HLX	exonic	nonsynonymous SNV	HLX:NM_021958:exon1:c.C499T:p.P167S	.	1.306	7.35E-05	0.0002	0.0002	Fam71_f_m_aF	0/0;0/1;0/1
chr1	224393929	224393929	C	T	WDR26	exonic	nonsynonymous SNV	WDR26:NM_001115113:exon13:c.G1811A:p.S604N,WDR26:NM_025160:exon13:c.G1859A:p.S620N	.	1.988	.	6.17E-05	6.98E-06	Fam117_f_m_aM_aF	0/1;0/0;0/0;0/1
chr1	226154826	226154826	A	G	ACBD3	exonic	nonsynonymous SNV	ACBD3:NM_022735:exon6:c.T911C:p.L304S	.	1.767	.	3.53E-05	1.40E-05	Fam55_f_m_aM_aM_dM	0/0;0/1;0/0;0/1;0/0
chr1	226161618	226161618	C	T	ACBD3	exonic	nonsynonymous SNV	ACBD3:NM_022735:exon4:c.G641A:p.R214H	.	1.182	7.34E-05	0.0004	0.0002	Fam23_f_m_aM_dF_uFs	0/1;0/0;0/1;0/0;0/0
chr1	227028708	227028708	G	A	CDC42BPA	exonic	stopgain	CDC42BPA:NM_001366010:exon28:c.C4192T:p.R1398X,CDC42BPA:NM_014826:exon28:c.C4033T:p.R1345X,CDC42BPA:NM_001366011:exon29:c.C4216T:p.R1406X,CDC42BPA:NM_003607:exon29:c.C4276T:p.R1426X,CDC42BPA:NM_001366019:exon30:c.C4315T:p.R1439X	0.455	.	.	1.14E-05	6.98E-06	Fam18_f_m_aM_uF	0/1;0/0;0/1;0/0
chr1	228147476	228147476	C	T	GUK1	exonic	nonsynonymous SNV	GUK1:NM_001159390:exon5:c.C385T:p.R129W,GUK1:NM_001159391:exon5:c.C322T:p.R108W,GUK1:NM_001242839:exon5:c.C322T:p.R108W,GUK1:NM_001242840:exon5:c.C385T:p.R129W,GUK1:NM_000858:exon6:c.C322T:p.R108W	.	1.021	.	7.46E-05	1.40E-05	Fam94_f_m_aM	0/0;0/1;0/1
chr1	228212318	228212318	G	A	OBSCN	exonic	nonsynonymous SNV	OBSCN:NM_001098623:exon2:c.G535A:p.G179S,OBSCN:NM_001271223:exon2:c.G535A:p.G179S,OBSCN:NM_052843:exon2:c.G535A:p.G179S	.	1.156	0.0003	.	2.16E-05	Fam66_f_m_aM	0/0;0/1;0/1

chr1	229295894	229295894	G	A	RAB4A	exonic	nonsynonymous SNV	RAB4A:NM_004578:exon4:c.G274A:p.V92I	.	1.255	7.34E-05	6.70E-05	5.59E-05	Fam1_f_m_aM	0/0;0/1;0/1
chr1	229547600	229547600	G	A	ABCB10	exonic	nonsynonymous SNV	ABCB10:NM_012089:exon3:c.C820T:p.R274C	.	1.362	.	2.23E-05	6.98E-06	Fam5_f_m_aM	0/0;0/1;0/1
chr1	229549344	229549344	G	T	ABCB10	exonic	nonsynonymous SNV	ABCB10:NM_012089:exon2:c.C608A:p.P203H	.	1.162	.	3.28E-05	6.98E-06	Fam55_f_m_aM_aM_dM	0/0;0/1;0/1;0/0;0/1
chr1	229594314	229594314	C	G	TAF5L	exonic	nonsynonymous SNV	TAF5L:NM_014409:exon5:c.G1753C:p.E585Q	.	1.45	.	.	6.98E-06	Fam95_f_m_aM_aM_uF	0/0;0/1;0/1;0/1;0/0
chr1	230262614	230262614	A	C	GALNT2	exonic	nonsynonymous SNV	GALNT2:NM_001291866:exon12:c.A1064C:p.K355T,GALNT2:NM_004481:exon12:c.A1178C:p.K393T	.	1.61	.	.	.	Fam6_f_m_aM	0/1;0/0;0/1
chr1	230911483	230911483	A	G	TTC13	exonic	nonsynonymous SNV	TTC13:NM_001122835:exon18:c.T2134C:p.S712P,TTC13:NM_024525:exon20:c.T2296C:p.S766P	.	1.078	.	1.15E-05	6.98E-06	Fam123_f_aF	0/1;0/1
chr1	231209075	231209075	G	A	TRIM67	exonic	nonsynonymous SNV	TRIM67:NM_001004342:exon8:c.G1948A:p.V650M,TRIM67:NM_001300889:exon10:c.G1762A:p.V588M	.	1.318	.	7.87E-05	2.09E-05	Fam121_f_m_aF_aM	0/0;0/1;0/1;0/1
chr1	231209202	231209202	T	C	TRIM67	exonic	nonsynonymous SNV	TRIM67:NM_001004342:exon8:c.T2075C:p.V692A,TRIM67:NM_001300889:exon10:c.T1889C:p.V630A	.	1.692	.	2.36E-05	6.98E-06	Fam101_f_m_aM	0/1;0/0;0/1
chr1	233328707	233328707	C	G	MAP3K21	exonic	nonsynonymous SNV	MAP3K21:NM_032435:exon1:c.C679G:p.R227G	.	3.099	.	.	.	Fam6_f_m_aM	0/1;0/0;0/1
chr1	233353838	233353838	G	A	MAP3K21	exonic	nonsynonymous SNV	MAP3K21:NM_032435:exon3:c.G1018A:p.G340R	.	1.935	7.34E-05	0.0002	0.0001	Fam90_f_m_aF_dM	0/0;0/1;0/1;0/0
chr1	233666730	233666730	C	T	KCNK1	exonic	nonsynonymous SNV	KCNK1:NM_002245:exon2:c.C491T:p.P164L	.	1.177	7.35E-05	1.12E-05	.	Fam80_f_m_aM_uM	0/0;0/1;0/1;0/0
chr1	234478931	234478931	G	C	TARBP1	exonic	nonsynonymous SNV	TARBP1:NM_005646:exon1:c.C173G:p.P58R	.	1.219	.	5.46E-05	1.41E-05	Fam15_f_m_aM_aM	0/1;0/0;0/1;0/0
chr1	234479064	234479064	G	A	TARBP1	exonic	nonsynonymous SNV	TARBP1:NM_005646:exon1:c.C40T:p.R14W	.	1.745	.	0.0001	1.40E-05	Fam31_f_m_aM_uF	0/0;0/1;0/1;0/1
chr1	236409255	236409255	G	A	EDARADD	exonic	nonsynonymous SNV	EDARADD:NM_080738:exon2:c.G71A:p.S24N,EDARADD:NM_145861:exon2:c.G101A:p.S34N	.	1.525	7.35E-05	.	2.79E-05	Fam123_f_aF	0/0;0/1
chr1	236835586	236835586	G	C	MTR	exonic	nonsynonymous SNV	MTR:NM_001291940:exon13:c.G7C:p.A3P,MTR:NM_000254:exon14:c.G1228C:p.A410P,MTR:NM_001291939:exon14:c.G1228C:p.A410P	.	1.423	.	.	7.12E-06	Fam9_f_m_aM_dM_uF	0/1;0/0;0/1;0/0;0/0
chr1	237445445	237445445	G	T	RYR2	exonic	nonsynonymous SNV	RYR2:NM_001035:exon14:c.G1215T:p.L405F	.	1.169	.	.	.	Fam114_f_m_aM	0/1;0/0;0/1
chr1	237506807	237506807	A	G	RYR2	exonic	nonsynonymous SNV	RYR2:NM_001035:exon23:c.A2711G:p.Y904C	.	1.286	0.0003	0.0001	0.0002	Fam78_f_m_aF_uM	0/0;0/1;0/1;0/1
chr1	237617395	237617395	T	G	RYR2	exonic	nonsynonymous SNV	RYR2:NM_001035:exon38:c.T5825G:p.F1942C	.	1.264	.	6.74E-05	.	Fam63_f_m_aF	0/1;0/0;0/1
chr1	246724442	246724442	C	T	SCCPDH	exonic	nonsynonymous SNV	SCCPDH:NM_016002:exon1:c.C20T:p.P7L	.	1.295	0.0005	0.001	0.0007	Fam61_f_m_aM	0/0;0/1;0/1
chr1	246724442	246724442	C	T	SCCPDH	exonic	nonsynonymous SNV	SCCPDH:NM_016002:exon1:c.C20T:p.P7L	.	1.295	0.0005	0.001	0.0007	Fam33_f_m_aM_uM	0/1;0/0;0/1;0/1
chr1	246903977	246903977	C	T	AHCTF1	exonic	nonsynonymous SNV	AHCTF1:NM_001323342:exon7:c.G938A:p.C313Y,AHCTF1:NM_001323343:exon7:c.G938A:p.C313Y,AHCTF1:NM_015446:exon7:c.G965A:p.C322Y	.	1.13	.	.	.	Fam60_f_m_aF	0/1;0/0;0/1
chr1	247329616	247329616	C	G	ZNF496	splicing	.	.	0.3	.	0.0001	6.91E-05	6.28E-05	Fam31_f_m_aM_uF	0/0;0/1;0/1;0/0
chr1	247424347	247424347	G	T	NLRP3	exonic	nonsynonymous SNV	NLRP3:NM_001127462:exon3:c.G904T:p.D302Y,NLRP3:NM_001243133:exon3:c.G898T:p.D300Y,NLRP3:NM_004895:exon3:c.G904T:p.D302Y,NLRP3:NM_183395:exon3:c.G904T:p.D302Y,NLRP3:NM_001127461:exon4:c.G904T:p.D302Y,NLRP3:NM_001079821:exon5:c.G904T:p.D302Y	.	1.497	.	.	.	Fam65_f_m_aM_uF_dF	0/1;0/0;0/1;0/0;0/0
chr1	248812298	248812298	G	T	SH3BP5L	exonic	nonsynonymous SNV	SH3BP5L:NM_001322463:exon7:c.C427A:p.R143S,SH3BP5L:NM_001322464:exon7:c.C283A:p.R95S,SH3BP5L:NM_030645:exon7:c.C784A:p.R262S,SH3BP5L:NM_001322462:exon9:c.C427A:p.R143S	.	1.392	7.36E-05	2.24E-05	.	Fam66_f_m_aM	0/0;0/1;0/1
chr10	247438	247438	G	A	ZMYND11	exonic	nonsynonymous SNV	ZMYND11:NM_001370122:exon9:c.G893A:p.R298H,ZMYND11:NM_001370124:exon9:c.G728A:p.R243H,ZMYND11:NM_001202465:exon10:c.G944A:p.R315H,ZMYND11:NM_001202467:exon10:c.G1037A:p.R346H,ZMYND11:NM_001370110:exon10:c.G944A:p.R315H,ZMYND11:NM_001370120:exon10:c.G971A:p.R324H,ZMYND11:NM_001370121:exon10:c.G917A:p.R306H,ZMYND11:NM_001370123:exon10:c.G842A:p.R281H,ZMYND11:NM_001202464:exon11:c.G1037A:p.R346H,ZMYND11:NM_001202466:exon11:c.G1034A:p.R345H,ZMYND11:NM_001202468:exon11:c.G1199A:p.R400H,ZMYND11:NM_001370103:exon11:c.G1037A:p.R346H,ZMYND11:NM_001370104:exon11:c.G1037A:p.R346H,ZMYND11:NM_001370105:exon11:c.G1037A:p.R346H,ZMYND11:NM_001370106:exon11:c.G1037A:p.R346H,ZMYND11:NM_001370107:exon11:c.G1037A:p.R346H,ZMYND11:NM_001370108:exon11:c.G1037A:p.R346H,ZMYND11:NM_001370109:exon11:c.G1037A:p.R346H,ZMYND11:NM_001370111:exon11:c.G1034A:p.R345H,ZMYND11:NM_001370113:exon11:c.G1106A:p.R369H,ZMYND11:NM_001370114:exon11:c.G1106A:p.R369H,ZMYND11:NM_001370116:exon11:c.G1133A:p.R378H,ZMYND11:NM_001370118:exon11:c.G1079A:p.R360H,ZMYND11:NM_001370097:exon12:c.G1199A:p.R400H,ZMYND11:NM_001370098:exon12:c.G1199A:p.R400H,ZMYND11:NM_001370099:exon12:c.G1199A:p.R400H,ZMYND11:NM_001370100:exon12:c.G1199A:p.R400H,ZMYND11:NM_001370101:exon12:c.G1199A:p.R400H,ZMYND11:NM_001370102:exon12:c.G1199A:p.R400H,ZMYND11:NM_001370115:exon12:c.G1196A:p.R399H,ZMYND11:NM_006624:exon12:c.G1199A:p.R400H,ZMYND11:NM_212479:exon12:c.G1196A:p.R399H,ZMYND11:NM_001330057:exon13:c.G1148A:p.R383H,ZMYND11:NM_001370112:exon13:c.G1148A:p.R383H	.	2.047	0.0003	1.12E-05	2.79E-05	Fam66_f_m_aM	0/0;0/1;0/1
chr10	1185019	1185019	G	A	ADARB2	exonic	nonsynonymous SNV	ADARB2:NM_018702:exon9:c.C1885T:p.R629C	.	1.151	0.0005	0.0006	0.0006	Fam13_f_m_aM	0/0;0/1;0/1

chr10	5452371	5452371	G	A	NET1	exonic	nonsynonymous SNV	NET1:NM_005863:exon3:c.G215A:p.R72H,NET1:NM_001047160:exon5:c.G377A:p.R126H	.	1.019	0.0008	0.0008	0.0004	Fam99_f_m_aM_aM	0/1;0/0;0/1;0/1
chr10	5936572	5936572	G	C	FBH1	exonic	nonsynonymous SNV	FBH1:NM_178150:exon20:c.G2946C:p.K982N,FBH1:NM_001258452:exon21:c.G2775C:p.K925N,FBH1:NM_001258453:exon21:c.G2724C:p.K908N,FBH1:NM_032807:exon21:c.G3099C:p.K1033N	.	1.822	0.0001	5.58E-05	8.38E-05	Fam110_f_m_aM_aM_uMs	0/1;0/0;0/0;0/1;0/0
chr10	6224199	6224199	C	T	PFKFB3	exonic	nonsynonymous SNV	PFKFB3:NM_001145443:exon13:c.C1267T:p.R423W,PFKFB3:NM_001282630:exon13:c.C1369T:p.R457W,PFKFB3:NM_001314063:exon13:c.C1327T:p.R443W,PFKFB3:NM_001323016:exon13:c.C1267T:p.R423W,PFKFB3:NM_001323017:exon13:c.C778T:p.R260W,PFKFB3:NM_001363545:exon13:c.C1327T:p.R443W,PFKFB3:NM_004566:exon13:c.C1327T:p.R443W	.	1.208	.	6.17E-05	5.58E-05	Fam59_f_m_aF_uM	0/1;0/0;0/1;0/0
chr10	6498444	6498444	G	C	PRKCQ	exonic	nonsynonymous SNV	PRKCQ:NM_001282645:exon4:c.C119G:p.T40S,PRKCQ:NM_001323266:exon4:c.C119G:p.T40S,PRKCQ:NM_001242413:exon5:c.C494G:p.T165S,PRKCQ:NM_001282644:exon5:c.C386G:p.T129S,PRKCQ:NM_001323265:exon5:c.C494G:p.T165S,PRKCQ:NM_001323267:exon5:c.C386G:p.T129S,PRKCQ:NM_006257:exon5:c.C494G:p.T165S	.	1.203	.	6.53E-05	3.49E-05	Fam32_f_m_aM_uM	0/1;0/0;0/1;0/0
chr10	7171013	7171013	T	C	SFMBT2	exonic	nonsynonymous SNV	SFMBT2:NM_001018039:exon20:c.A2459G:p.N820S,SFMBT2:NM_001029880:exon20:c.A2459G:p.N820S	.	1.993	.	3.28E-05	6.98E-06	Fam110_f_m_aM_aM_uMs	0/1;0/0;0/1;0/0;0/1
chr10	12828812	12828812	G	-	CAMK1D	exonic	frameshift deletion	CAMK1D:NM_153498:exon11:c.1083delG:p.V363Sfs*15	0.283	.	.	8.95E-05	2.79E-05	Fam101_f_m_aM	0/1;0/0;0/1
chr10	13656896	13656896	G	A	FRMD4A	exonic	nonsynonymous SNV	FRMD4A:NM_001318338:exon11:c.C1766T:p.P589L,FRMD4A:NM_001318336:exon21:c.C2741T:p.P914L,FRMD4A:NM_001318337:exon21:c.C2792T:p.P931L,FRMD4A:NM_018027:exon22:c.C2693T:p.P898L	.	1.088	0.0003	5.14E-05	5.61E-05	Fam31_f_m_aM_uF	0/0;0/1;0/1;0/0
chr10	13656914	13656914	T	A	FRMD4A	exonic	nonsynonymous SNV	FRMD4A:NM_001318338:exon11:c.A1748T:p.D583V,FRMD4A:NM_001318336:exon21:c.A2723T:p.D908V,FRMD4A:NM_001318337:exon21:c.A2774T:p.D925V,FRMD4A:NM_018027:exon22:c.A2675T:p.D892V	.	1.078	.	.	.	Fam64_f_m_aM	0/1;0/0;0/1
chr10	15214593	15214593	C	T	FAM171A1	exonic	nonsynonymous SNV	FAM171A1:NM_001010924:exon8:c.G995A:p.C332Y	.	1.196	.	0.0002	.	Fam29_f_m_aF_uM	0/1;0/0;0/1;0/1
chr10	22329127	22329127	G	A	BMI1;COMMD3-B	exonic	nonsynonymous SNV	BMI1:NM_005180:exon9:c.G650A:p.R217K,COMMD3-BMI1:NM_001204062:exon13:c.G1079A:p.R360K	.	1.226	.	.	.	Fam118_f_m_aM_aF_uM	0/0;0/1;0/1;0/1;0/1
chr10	26292956	26292956	G	A	GAD2	exonic	nonsynonymous SNV	GAD2:NM_000818:exon15:c.G1549A:p.E517K,GAD2:NM_001134366:exon15:c.G1549A:p.E517K	.	1.264	.	.	.	Fam34_f_m_aM_uF	0/0;0/1;0/1;0/0
chr10	27114547	27114547	C	T	YME1L1	exonic	nonsynonymous SNV	YME1L1:NM_001253866:exon17:c.G1882A:p.E628K,YME1L1:NM_014263:exon18:c.G1981A:p.E661K,YME1L1:NM_139312:exon19:c.G2152A:p.E718K	.	1.993	.	.	.	Fam117_f_m_aM_aF	0/0;0/0;0/1;0/0
chr10	30451706	30451706	G	A	MAP3K8	exonic	nonsynonymous SNV	MAP3K8:NM_001244134:exon5:c.G835A:p.E279K,MAP3K8:NM_001320961:exon5:c.G835A:p.E279K,MAP3K8:NM_005204:exon6:c.G835A:p.E279K	.	1.758	.	3.34E-05	.	Fam79_f_m_aM_uM	0/0;0/1;0/1;0/1
chr10	32923650	32923650	T	C	ITGB1	exonic	nonsynonymous SNV	ITGB1:NM_033668:exon6:c.A877G:p.I293V,ITGB1:NM_002211:exon7:c.A877G:p.I293V,ITGB1:NM_133376:exon7:c.A877G:p.I293V	.	1.455	.	.	.	Fam44_f_m_aM_uF	0/0;0/1;0/1;0/0
chr10	35566130	35566130	A	T	CCNY	exonic	nonsynonymous SNV	CCNY:NM_001282854:exon7:c.A455T:p.N152I,CCNY:NM_001282852:exon8:c.A779T:p.N260I,CCNY:NM_145012:exon9:c.A854T:p.N285I,CCNY:NM_001282853:exon10:c.A692T:p.N231I,CCNY:NM_181698:exon11:c.A692T:p.N231I	.	1.759	.	.	.	Fam110_f_m_aM_aM_uMs	0/0;0/1;0/0;0/1;0/0
chr10	43102402	43102402	G	A	RET	exonic	nonsynonymous SNV	RET:NM_020630:exon3:c.G398A:p.R133H,RET:NM_020975:exon3:c.G398A:p.R133H	.	1.571	0.0003	0.0007	0.0001	Fam24_f_m_aM_aM	0/0;0/1;0/1;0/1
chr10	43201929	43201929	A	G	RASGEF1A	exonic	nonsynonymous SNV	RASGEF1A:NM_001282862:exon4:c.T362C:p.F121S,RASGEF1A:NM_145313:exon4:c.T338C:p.F113S	.	1.3	.	.	.	Fam88_f_m_aF	0/1;0/0;0/1
chr10	59806926	59806926	C	T	CCDC6	exonic	nonsynonymous SNV	CCDC6:NM_005436:exon6:c.G1000A:p.E334K	.	2.216	.	.	.	Fam6_f_m_aM	0/0;0/1;0/1
chr10	60166650	60166650	C	T	ANK3	exonic	nonsynonymous SNV	ANK3:NM_001204404:exon23:c.G2504A:p.R835H,ANK3:NM_001320874:exon23:c.G2555A:p.R852H,ANK3:NM_020987:exon23:c.G2555A:p.R852H,ANK3:NM_001204403:exon24:c.G2537A:p.R846H	.	1.49	0.0001	0.0008	0.0002	Fam85_f_m_aM_aM	0/0;0/1;0/1;0/0
chr10	62091385	62091385	C	T	ARID5B	exonic	nonsynonymous SNV	ARID5B:NM_001244638:exon7:c.C1193T:p.A398V,ARID5B:NM_032199:exon10:c.C1922T:p.A641V	.	1.021	7.34E-05	3.27E-05	2.09E-05	Fam112_f_m_aM	0/0;0/1;0/1
chr10	68231512	68231512	G	A	ATOH7	exonic	nonsynonymous SNV	ATOH7:NM_145178:exon1:c.C166T:p.L56F	.	1.984	7.51E-05	3.37E-05	4.92E-05	Fam35_f_m_aF_uM	0/1;0/0;0/1;0/0
chr10	68673044	68673044	A	G	TET1	exonic	nonsynonymous SNV	TET1:NM_030625:exon8:c.A4823G:p.H1608R	.	2.268	.	2.24E-05	6.98E-06	Fam67_f_m_aM_uF	0/1;0/0;0/1;0/1
chr10	68913553	68913553	T	C	DDX50	exonic	nonsynonymous SNV	DDX50:NM_024045:exon6:c.T920C:p.I307T	.	1.578	7.34E-05	.	1.40E-05	Fam121_f_m_aF_aM	0/1;0/0;0/1;0/1
chr10	69200449	69200449	G	A	SUPV3L1	exonic	nonsynonymous SNV	SUPV3L1:NM_001323587:exon10:c.G481A:p.D161N,SUPV3L1:NM_001323588:exon10:c.G481A:p.D161N,SUPV3L1:NM_001323584:exon11:c.G1075A:p.D359N,SUPV3L1:NM_003171:exon11:c.G1468A:p.D490N,SUPV3L1:NM_001301683:exon12:c.G1075A:p.D359N,SUPV3L1:NM_001323586:exon12:c.G1105A:p.D369N,SUPV3L1:NM_001323585:exon13:c.G1105A:p.D369N	.	1.002	.	.	.	Fam48_f_m_aM_uM	0/1;0/0;0/1;0/0
chr10	69504460	69504460	G	A	TSPAN15	exonic	nonsynonymous SNV	TSPAN15:NM_001351263:exon4:c.G332A:p.C111Y,TSPAN15:NM_012339:exon6:c.G593A:p.C198Y	.	1.516	.	.	.	Fam47_f_m_aM	0/1;0/0;0/1
chr10	73345190	73345190	G	C	CFAP70	exonic	nonsynonymous SNV	CFAP70:NM_001367801:exon6:c.C484G:p.Q162E	.	1.011	.	.	.	Fam12_f_m_aM_uM_aM	0/1;0/0;0/1;0/0;0/1
chr10	73760821	73760821	G	A	SEC24C	exonic	nonsynonymous SNV	SEC24C:NM_198597:exon6:c.G959A:p.R320H,SEC24C:NM_004922:exon7:c.G959A:p.R320H	.	1.442	.	.	6.98E-06	Fam106_f_m_aM	0/1;0/0;0/1
chr10	73782170	73782170	G	C	CHCHD1	exonic	nonsynonymous SNV	CHCHD1:NM_203298:exon1:c.G95C:p.R32P	.	1.337	.	6.54E-05	1.40E-05	Fam84_f_m_aF	0/0;0/1;0/1

chr10	73791072	73791072	G	A	ZSWIM8	exonic	nonsynonymous SNV	ZSWIM8:NM_001242487:exon8:c.G1039A:p.V347I,ZSWIM8:NM_001242488:exon8:c.G1039A:p.V347I,ZSWIM8:NM_001367799:exon8:c.G1039A:p.V347I,ZSWIM8:NM_015037:exon8:c.G1039A:p.V347I	.	1.139	0.0003	1.13E-05	1.40E-05	Fam49_f_m_aM	0/0;0/1;0/1
chr10	73794564	73794564	C	T	ZSWIM8	exonic	nonsynonymous SNV	ZSWIM8:NM_001242487:exon14:c.C2818T:p.P940S,ZSWIM8:NM_001242488:exon14:c.C2818T:p.P940S,ZSWIM8:NM_001367799:exon14:c.C2833T:p.P945S,ZSWIM8:NM_015037:exon14:c.C2833T:p.P945S	.	1.024	.	.	.	Fam77_f_m_aM_aF_uM_uFs	0/1;0/0;0/1;0/1;0/1;0/0
chr10	73801121	73801121	G	A	ZSWIM8	exonic	nonsynonymous SNV	ZSWIM8:NM_001242487:exon25:c.G5236A:p.A1746T,ZSWIM8:NM_001242488:exon25:c.G5144A:p.R1715H,ZSWIM8:NM_001367799:exon25:c.G5227A:p.A1743T,ZSWIM8:NM_015037:exon25:c.G5251A:p.A1751T	.	1.171	0.001	0.0004	0.0003	Fam77_f_m_aM_aF	0/1;0/0;0/1;0/1
chr10	73805652	73805652	G	A	NDST2	exonic	nonsynonymous SNV	NDST2:NM_001330107:exon8:c.C1681T:p.P561S,NDST2:NM_003635:exon8:c.C1681T:p.P561S	.	1.279	.	.	.	Fam34_f_m_aM_uF	0/1;0/0;0/1;0/0
chr10	73808060	73808060	C	T	NDST2	exonic	nonsynonymous SNV	NDST2:NM_001330107:exon3:c.G329A:p.R110H,NDST2:NM_003635:exon3:c.G329A:p.R110H	.	1.427	7.34E-05	0.0001	2.79E-05	Fam97_f_m_aM_aF	0/0;0/1;0/1;0/1
chr10	73817551	73817551	C	T	CAMK2G	exonic	nonsynonymous SNV	CAMK2G:NM_001367538:exon14:c.G776A:p.R259Q,CAMK2G:NM_001367537:exon15:c.G890A:p.R297Q,CAMK2G:NM_001367540:exon15:c.G422A:p.R141Q,CAMK2G:NM_001222:exon16:c.G1088A:p.R363Q,CAMK2G:NM_001367541:exon16:c.G1088A:p.R363Q,CAMK2G:NM_001367516:exon17:c.G1157A:p.R386Q,CAMK2G:NM_001367517:exon17:c.G1157A:p.R386Q,CAMK2G:NM_001367519:exon17:c.G1121A:p.R374Q,CAMK2G:NM_001367528:exon17:c.G1202A:p.R401Q,CAMK2G:NM_001367529:exon17:c.G1157A:p.R386Q,CAMK2G:NM_001367530:exon17:c.G1121A:p.R374Q,CAMK2G:NM_001367532:exon17:c.G1133A:p.R378Q,CAMK2G:NM_001367539:exon17:c.G1025A:p.R342Q,CAMK2G:NM_001367545:exon17:c.G1202A:p.R401Q,CAMK2G:NM_172170:exon17:c.G1157A:p.R386Q,CAMK2G:NM_172173:exon17:c.G1115A:p.R372Q,CAMK2G:NM_001204492:exon18:c.G1220A:p.R407Q,CAMK2G:NM_001367514:exon18:c.G1247A:p.R416Q,CAMK2G:NM_001367518:exon18:c.G1235A:p.R412Q,CAMK2G:NM_001367520:exon18:c.G1229A:p.R410Q,CAMK2G:NM_001367521:exon18:c.G1220A:p.R407Q,CAMK2G:NM_001367522:exon18:c.G1235A:p.R412Q,CAMK2G:NM_001367523:exon18:c.G1184A:p.R395Q,CAMK2G:NM_001367524:exon18:c.G1052A:p.R351Q,CAMK2G:NM_001367525:exon18:c.G1247A:p.R416Q,CAMK2G:NM_001367527:exon18:c.G1265A:p.R422Q,CAMK2G:NM_001367531:exon18:c.G1190A:p.R397Q,CAMK2G:NM_001367533:exon18:c.G1229A:p.R410Q,CAMK2G:NM_001367535:exon18:c.G1243A:p.E415K,CAMK2G:NM_001367536:exon18:c.G1208A:p.R403Q,CAMK2G:NM_172169:exon18:c.G1184A:p.R395Q,CAMK2G:NM_172171:exon18:c.G1271A:p.R424Q,CAMK2G:NM_001320898:exon19:c.G1298A:p.R433Q,CAMK2G:NM_001367526:exon19:c.G1330A:p.E444K,CAMK2G:NM_001367543:exon19:c.G1253A:p.R418Q,CAMK2G:NM_001367544:exon19:c.G1334A:p.R445Q,CAMK2G:NM_001367546:exon19:c.G1298A:p.R433Q,CAMK2G:NM_001367547:exon19:c.G1298A:p.R433Q,CAMK2G:NM_001367534:exon20:c.G1367A:p.R456Q,CAMK2G:NM_001367542:exon20:c.G581A:p.R194Q,CAMK2G:NM_001367548:exon20:c.G1325A:p.R442Q	.	1.802	.	3.28E-05	1.40E-05	Fam52_f_m_aM	0/1;0/0;0/1
chr10	73824085	73824085	C	G	CAMK2G	splicing	.	.	0.396	.	.	.	.	Fam57_f_m_aM_uF	0/1;0/0;0/1;0/0
chr10	73912966	73912966	G	A	PLAU	exonic	nonsynonymous SNV	PLAU:NM_001145031:exon4:c.G185A:p.R62Q,PLAU:NM_002658:exon5:c.G236A:p.R79Q	.	1.045	0.0005	0.0005	0.0001	Fam74_f_m_aM_uM	0/1;0/0;0/1;0/1
chr10	73913604	73913604	C	T	PLAU	exonic	nonsynonymous SNV	PLAU:NM_001145031:exon6:c.C475T:p.R159C,PLAU:NM_001319191:exon6:c.C268T:p.R90C,PLAU:NM_002658:exon7:c.C526T:p.R176C	.	1.141	.	0.0001	6.28E-05	Fam73_f_m_aF_dM	0/0;0/1;0/1;0/1
chr10	75037945	75037945	G	A	DUPD1	exonic	nonsynonymous SNV	DUPD1:NM_001003892:exon3:c.C554T:p.P185L	.	1.144	.	6.71E-05	1.40E-05	Fam112_f_m_aM	0/0;0/1;0/1
chr10	76887420	76887420	C	T	KCNMA1	exonic	nonsynonymous SNV	KCNMA1:NM_001271518:exon27:c.G3233A:p.R1078Q,KCNMA1:NM_001322832:exon27:c.G3383A:p.R1128Q,KCNMA1:NM_002247:exon27:c.G3383A:p.R1128Q,KCNMA1:NM_001014797:exon28:c.G3395A:p.R1132Q,KCNMA1:NM_001161352:exon28:c.G3557A:p.R1186Q,KCNMA1:NM_001161353:exon28:c.G3506A:p.R1169Q,KCNMA1:NM_001322829:exon28:c.G3392A:p.R1131Q,KCNMA1:NM_001322836:exon28:c.G3392A:p.R1131Q,KCNMA1:NM_001271519:exon29:c.G3473A:p.R1158Q,KCNMA1:NM_001322835:exon29:c.G3476A:p.R1159Q,KCNMA1:NM_001322837:exon29:c.G3476A:p.R1159Q,KCNMA1:NM_001322838:exon29:c.G2930A:p.R977Q,KCNMA1:NM_001322830:exon30:c.G3485A:p.R1162Q	.	1.074	.	3.28E-05	.	Fam28_f_m_aF_uF	0/0;0/1;0/1;0/1
chr10	79386348	79386348	C	A	ZCCHC24	exonic	nonsynonymous SNV	ZCCHC24:NM_153367:exon4:c.G723T:p.Q241H	.	1.935	.	.	.	Fam64_f_m_aM	0/1;0/0;0/1
chr10	87094567	87094567	T	G	GLUD1	exonic	nonsynonymous SNV	GLUD1:NM_005271:exon1:c.A203C:p.K68T	.	1.361	.	1.12E-05	.	Fam11_f_m_aM_uM	0/1;0/0;0/1;0/0
chr10	91482997	91482997	G	A	HECTD2	exonic	nonsynonymous SNV	HECTD2:NM_001284274:exon8:c.G742A:p.V248I,HECTD2:NM_001348365:exon8:c.G421A:p.V141I,HECTD2:NM_182765:exon8:c.G742A:p.V248I	.	1.025	.	.	.	Fam30_f_m_aM_uM	0/0;0/0;0/1;0/0

chr10	91842212	91842212	C	T	TNKS2	exonic	nonsynonymous SNV	TNKS2:NM_025235:exon16:c.C1880T:p.T627I	.	1.343	.	0.0003	4.89E-05	Fam109_f_m_aM	0/1;0/0;0/1
chr10	92609103	92609103	A	C	KIF11	exonic	nonsynonymous SNV	KIF11:NM_004523:exon5:c.A471C:p.K157N	.	2.377	.	.	.	Fam113_f_m_aF_raM_uF	0/0;0/1;0/1;0/0;0/1
chr10	93065956	93065956	G	A	CYP26C1	exonic	nonsynonymous SNV	CYP26C1:NM_183374:exon5:c.G862A:p.E288K	.	1.833	0.0007	0.0005	0.0004	Fam79_f_m_aM_uM	0/1;0/0;0/1;0/1
chr10	93601674	93601674	G	-	RBP4	exonic	frameshift deletion	RBP4:NM_001323518:exon1:c.43delC:p.Q15Rfs*8	0.575	.	.	6.66E-05	.	Fam116_f_m_aM_aF	0/1;0/0;0/0;0/1
chr10	94403046	94403046	G	C	TBC1D12	exonic	nonsynonymous SNV	TBC1D12:NM_015188:exon1:c.G433C:p.D145H	.	1.335	.	.	.	Fam101_f_m_aM	0/1;0/0;0/1
chr10	94588313	94588313	C	T	HELLS	exonic	nonsynonymous SNV	HELLS:NM_001289070:exon11:c.C1117T:p.P373S,HELLS:NM_001289072:exon11:c.C1021T:p.P341S,HELLS:NM_001289073:exon12:c.C997T:p.P333S,HELLS:NM_001289075:exon12:c.C193T:p.P65S,HELLS:NM_001289068:exon13:c.C1363T:p.P455S,HELLS:NM_001289069:exon13:c.C1315T:p.P439S,HELLS:NM_018063:exon13:c.C1411T:p.P471S,HELLS:NM_001289067:exon14:c.C1549T:p.P517S,HELLS:NM_001289071:exon14:c.C1039T:p.P347S,HELLS:NM_001289074:exon14:c.C328T:p.P110S	.	1.578	.	2.24E-05	4.19E-05	Fam77_f_m_aM_aF_uM_uFs	0/1;0/0;0/1;0/1;0/1;0/0
chr10	96551263	96551263	A	G	TM9SF3	exonic	nonsynonymous SNV	TM9SF3:NM_020123:exon7:c.T941C:p.I314T	.	1.221	.	.	.	Fam12_f_m_aM_uM_aM	0/0;0/0;0/0;0/0;0/1
chr10	96586780	96586780	A	G	TM9SF3	exonic	nonsynonymous SNV	TM9SF3:NM_020123:exon1:c.T56C:p.L19P	.	1.481	7.56E-05	0.0002	0.0001	Fam23_f_m_aM_dF_uFs	0/1;0/0;0/1;0/1;0/0
chr10	96651633	96651633	A	T	PIK3AP1	exonic	nonsynonymous SNV	PIK3AP1:NM_152309:exon5:c.T731A:p.V244D	.	1.882	.	.	.	Fam29_f_m_aF_uM	0/0;0/1;0/1;0/0
chr10	97001118	97001118	G	T	SLIT1	exonic	stopgain	SLIT1:NM_003061:exon37:c.C4599A:p.C1533X	0.432	.	.	1.13E-05	6.98E-06	Fam43_f_m_aM	0/1;0/0;0/1
chr10	97046694	97046694	G	A	SLIT1	exonic	nonsynonymous SNV	SLIT1:NM_003061:exon18:c.C1813T:p.R605W	.	1.332	.	0.0005	4.19E-05	Fam43_f_m_aM	0/1;0/0;0/1
chr10	97056433	97056433	G	A	SLIT1	exonic	nonsynonymous SNV	SLIT1:NM_003061:exon13:c.C1189T:p.R397W	.	1.34	.	0.0003	2.09E-05	Fam92_f_m_aM_raM	0/1;0/0;0/1;0/1
chr10	97319820	97319820	G	A	FRAT1	exonic	nonsynonymous SNV	FRAT1:NM_005479:exon1:c.G367A:p.G123S	.	2.146	.	.	.	Fam34_f_m_aM_uF	0/1;0/0;0/1;0/0
chr10	97334272	97334272	C	G	FRAT2	exonic	nonsynonymous SNV	FRAT2:NM_012083:exon1:c.G301C:p.E101Q	.	2.012	0.0003	.	3.57E-05	Fam25_f_m_aM	0/0;0/1;0/1
chr10	97401168	97401168	G	A	RRP12	exonic	nonsynonymous SNV	RRP12:NM_001145114:exon1:c.C64T:p.H22Y,RRP12:NM_001284337:exon1:c.C64T:p.H22Y,RRP12:NM_015179:exon1:c.C64T:p.H22Y	.	1.7	.	2.24E-05	1.40E-05	Fam107_f_m_aM	0/1;0/0;0/1
chr10	97673642	97673642	C	T	PI4K2A	exonic	nonsynonymous SNV	PI4K2A:NM_018425:exon9:c.C1340T:p.P447L	.	1.47	.	1.12E-05	.	Fam117_f_m_aM_aF	0/1;0/0;0/1;0/0
chr10	97917581	97917581	C	T	CRTAC1	exonic	nonsynonymous SNV	CRTAC1:NM_001206528:exon5:c.G634A:p.D212N,CRTAC1:NM_018058:exon5:c.G634A:p.D212N	.	1.062	.	1.12E-05	.	Fam9_f_m_aM_dM_uF	0/0;0/1;0/1;0/1;0/0
chr10	100156163	100156163	G	T	ERLIN1	exonic	nonsynonymous SNV	ERLIN1:NM_001347858:exon8:c.C247A:p.R83S,ERLIN1:NM_001347856:exon9:c.C475A:p.R159S,ERLIN1:NM_006459:exon9:c.C727A:p.R243S,ERLIN1:NM_001100626:exon10:c.C727A:p.R243S,ERLIN1:NM_001347857:exon10:c.C727A:p.R243S,ERLIN1:NM_001347859:exon10:c.C727A:p.R243S,ERLIN1:NM_001347860:exon10:c.C727A:p.R243S,ERLIN1:NM_001347861:exon10:c.C727A:p.R243S	.	1.527	.	.	.	Fam98_f_m_aM	0/1;0/0;0/1
chr10	100222882	100222882	C	T	CHUK	exonic	nonsynonymous SNV	CHUK:NM_001278:exon3:c.G299A:p.G100E,CHUK:NM_001320928:exon3:c.G299A:p.G100E	.	1.038	.	.	.	Fam29_f_m_aF_uM	0/0;0/1;0/1;0/0
chr10	100482643	100482643	G	A	WNT8B	exonic	nonsynonymous SNV	WNT8B:NM_003393:exon6:c.G883A:p.G295R	.	2.121	.	1.24E-05	.	Fam96_f_m_aM_uF	0/1;0/0;0/1;0/0
chr10	100545058	100545058	C	G	HIF1AN	exonic	nonsynonymous SNV	HIF1AN:NM_017902:exon4:c.C685G:p.P229A	.	1.807	.	8.93E-05	6.98E-05	Fam15_f_m_aM_aM	0/1;0/0;0/1;0/1
chr10	100824669	100824669	C	T	PAX2	exonic	nonsynonymous SNV	PAX2:NM_000278:exon8:c.C941T:p.T314I,PAX2:NM_003988:exon8:c.C941T:p.T314I,PAX2:NM_003989:exon8:c.C941T:p.T314I,PAX2:NM_001304569:exon9:c.C1034T:p.T345I,PAX2:NM_003987:exon9:c.C1010T:p.T337I,PAX2:NM_003990:exon9:c.C1010T:p.T337I	.	1.009	.	.	.	Fam115_f_m_aF_aM_aF	0/1;0/0;0/0;0/0;0/1
chr10	101975268	101975268	C	T	ARMH3	exonic	nonsynonymous SNV	ARMH3:NM_024541:exon20:c.G1439A:p.C480Y	.	1.817	.	.	.	Fam6_f_m_aM	0/0;0/1;0/1
chr10	102067290	102067290	C	T	HPS6	exonic	nonsynonymous SNV	HPS6:NM_024747:exon1:c.C1816T:p.R606C	.	1.604	.	4.58E-05	2.09E-05	Fam109_f_m_aM	0/1;0/0;0/1
chr10	102421137	102421137	C	T	FBXL15	exonic	nonsynonymous SNV	FBXL15:NM_024326:exon1:c.C8T:p.P3L	.	1.09	7.34E-05	0.0005	9.07E-05	Fam37_f_m_aF_uM	0/1;0/0;0/1;0/1
chr10	103373496	103373496	G	A	TAF5	exonic	nonsynonymous SNV	TAF5:NM_006951:exon2:c.G698A:p.R233Q,TAF5:NM_139052:exon2:c.G698A:p.R233Q	.	1.318	.	1.12E-05	.	Fam91_m_aM_dM_aM_dM	0/1;0/1;0/1;0/0;0/0
chr10	103388215	103388215	C	T	TAF5	exonic	nonsynonymous SNV	TAF5:NM_139052:exon10:c.C2230T:p.P744S,TAF5:NM_006951:exon11:c.C2395T:p.P799S	.	1.164	.	1.12E-05	.	Fam123_f_aF	0/1;0/1
chr10	103584907	103584907	A	C	NEURL1	exonic	nonsynonymous SNV	NEURL1:NM_004210:exon4:c.A1021C:p.K341Q	.	1.203	.	.	.	Fam43_f_m_aM	0/0;0/1;0/1
chr10	103585006	103585006	C	T	NEURL1	exonic	nonsynonymous SNV	NEURL1:NM_004210:exon4:c.C1120T:p.P374S	.	1.387	0.0001	1.47E-05	2.79E-05	Fam99_f_m_aM_aM	0/1;0/0;0/0;0/1
chr10	105223139	105223139	C	T	SORCS3	exonic	stopgain	SORCS3:NM_014978:exon20:c.C2758T:p.R920X	0.515	.	7.35E-05	6.19E-05	1.40E-05	Fam16_f_m_aM_aM	0/1;0/0;0/0;0/1
chr10	115334327	115334327	G	T	ATRNL1	exonic	nonsynonymous SNV	ATRNL1:NM_207303:exon19:c.G3083T:p.C1028F	.	1.049	.	.	.	Fam48_f_m_aM_uM	0/0;0/1;0/1;0/1
chr10	116681194	116681194	G	A	HSPA12A	exonic	nonsynonymous SNV	HSPA12A:NM_025015:exon9:c.C985T:p.R329W,HSPA12A:NM_001330164:exon10:c.C1036T:p.R346W	.	1.206	0.0007	0.0007	0.0005	Fam89_f_m_aM	0/0;0/1;0/1
chr10	117318960	117318960	C	T	PDZD8	exonic	nonsynonymous SNV	PDZD8:NM_173791:exon3:c.G1010A:p.G337E	.	2.059	.	.	.	Fam76_f_m_aM_uM	0/1;0/0;0/1;0/1
chr10	118594362	118594362	G	A	PRLHR	exonic	nonsynonymous SNV	PRLHR:NM_004248:exon2:c.C883T:p.H295Y	.	1.231	.	.	.	Fam51_f_m_aM_uF	0/0;0/1;0/1;0/1
chr10	118594899	118594899	G	A	PRLHR	exonic	nonsynonymous SNV	PRLHR:NM_004248:exon2:c.C346T:p.L116F	.	1.01	.	1.14E-05	.	Fam15_f_m_aM_aM	0/0;0/1;0/0;0/1
chr10	122429922	122429922	C	T	PLEKHA1	exonic	nonsynonymous SNV	PLEKHA1:NM_001001974:exon12:c.C1199T:p.P400L,PLEKHA1:NM_021622:exon12:c.C1199T:p.P400L	.	1.151	0.0007	0.0004	0.0003	Fam119_f_m_aM_aM	0/0;0/1;0/1;0/0
chr10	123150059	123150059	A	G	HMX2	exonic	nonsynonymous SNV	HMX2:NM_005519:exon2:c.A758G:p.Y253C	.	1.81	.	.	6.98E-06	Fam31_f_m_aM_uF	0/1;0/0;0/1;0/1
chr10	124994680	124994680	C	T	CTBP2	exonic	nonsynonymous SNV	CTBP2:NM_001363508:exon5:c.G773A:p.R258H,CTBP2:NM_022802:exon5:c.G2189A:p.R730H,CTBP2:NM_001321013:exon6:c.G569A:p.R190H,CTBP2:NM_001083914:exon7:c.G569A:p.R190H,CTBP2:NM_001290214:exon7:c.G569A:p.R190H,CTBP2:NM_001290215:exon7:c.G569A:p.R190H,CTBP2:NM_001321012:exon7:c.G569A:p.R190H,CTBP2:NM_001321014:exon7:c.G569A:p.R190H,CTBP2:NM_001329:exon7:c.G569A:p.R190H	.	1.445	7.34E-05	7.45E-05	4.88E-05	Fam45_f_m_aM_uF	0/1;0/0;0/1;0/1

chr10	125003083	125003083	C	A	CTBP2	exonic	nonsynonymous SNV	CTBP2:NM_001363508:exon3:c.G439T:p.A147S,CTBP2:NM_022802:exon3:c.G1855T:p.A619S,CTBP2:NM_001321013:exon4:c.G235T:p.A79S,CTBP2:NM_001083914:exon5:c.G235T:p.A79S,CTBP2:NM_001290214:exon5:c.G235T:p.A79S,CTBP2:NM_001290215:exon5:c.G235T:p.A79S,CTBP2:NM_001321012:exon5:c.G235T:p.A79S,CTBP2:NM_001321014:exon5:c.G235T:p.A79S,CTBP2:NM_001329:exon5:c.G235T:p.A79S	.	1.882	7.34E-05	0.0002	6.28E-05	Fam66_f_m_aM	0/1;0/0;0/1
chr10	127738940	127738940	A	G	FOXI2	exonic	nonsynonymous SNV	FOXI2:NM_207426:exon2:c.A932G:p.Y311C	.	1.682	7.36E-05	5.88E-05	2.79E-05	Fam37_f_m_aF_uM	0/1;0/0;0/1;0/0
chr10	129841021	129841021	G	A	EBF3	exonic	nonsynonymous SNV	EBF3:NM_001005463:exon14:c.C1357T:p.R453C	.	1.88	.	6.82E-05	7.28E-06	Fam88_f_m_aF	0/1;0/0;0/1
chr10	132200891	132200891	C	A	DPYSL4	exonic	nonsynonymous SNV	DPYSL4:NM_006426:exon10:c.C1018A:p.Q340K	.	1.065	.	.	.	Fam55_f_m_aM_aM_dM	0/0;0/1;0/1;0/1;0/1
chr10	132226817	132226817	G	A	STK32C	exonic	nonsynonymous SNV	STK32C:NM_001318878:exon4:c.C661T:p.R221C,STK32C:NM_001318879:exon4:c.C271T:p.R91C,STK32C:NM_173575:exon4:c.C622T:p.R208C	.	1.634	.	3.27E-05	6.98E-06	Fam115_f_m_aF_aM_aF	0/1;0/0;0/1;0/0;0/0
chr10	132405368	132405368	G	A	PWWP2B	exonic	nonsynonymous SNV	PWWP2B:NM_001098637:exon2:c.G868A:p.G290S,PWWP2B:NM_138499:exon2:c.G868A:p.G290S	.	1.182	.	7.86E-05	.	Fam22_f_m_aF_aF_uF	0/0;0/1;0/1;0/1;0/0
chr10	132749518	132749518	C	T	INPP5A	exonic	nonsynonymous SNV	INPP5A:NM_001321042:exon7:c.C542T:p.T181M,INPP5A:NM_005539:exon10:c.C734T:p.T245M	.	1.581	7.34E-05	6.54E-05	6.98E-06	Fam72_f_m_aF_uF	0/0;0/1;0/1;0/1
chr10	133128724	133128724	G	C	ADGRA1	exonic	nonsynonymous SNV	ADGRA1:NM_001291085:exon4:c.G605C:p.G202A,ADGRA1:NM_001083909:exon7:c.G896C:p.G299A	.	1.295	.	.	.	Fam66_f_m_aM	0/0;0/1;0/1
chr11	244126	244126	-	G	PSMD13	exonic	frameshift insertion	PSMD13:NM_175932:exon2:c.182dupG:p.R62Afs*26	0.268	.	.	2.24E-05	.	Fam110_f_m_aM_aM_uMs	0/1;0/0;0/1;0/1;0/0
chr11	460270	460270	C	T	PTDSS2	exonic	nonsynonymous SNV	PTDSS2:NM_001329544:exon2:c.C266T:p.T89M,PTDSS2:NM_001329548:exon2:c.C86T:p.T29M,PTDSS2:NM_030783:exon2:c.C266T:p.T89M	.	1.308	.	3.36E-05	6.98E-06	Fam83_f_m_aF	0/1;0/0;0/1
chr11	607064	607064	A	G	PHRF1	splicing	.	.	0.574	.	.	6.81E-05	4.88E-05	Fam78_f_m_aF_uM	0/0;0/1;0/1;0/1
chr11	792367	792367	C	T	SLC25A22	exonic	nonsynonymous SNV	SLC25A22:NM_001191060:exon8:c.G679A:p.V227M,SLC25A22:NM_001191061:exon8:c.G679A:p.V227M,SLC25A22:NM_024698:exon8:c.G679A:p.V227M	.	1.048	7.36E-05	0.0001	0.0002	Fam47_f_m_aM	0/1;0/0;0/1
chr11	1442548	1442548	G	A	BRSK2	exonic	nonsynonymous SNV	BRSK2:NM_001256627:exon5:c.G472A:p.A158T,BRSK2:NM_001256629:exon5:c.G472A:p.A158T,BRSK2:NM_001256630:exon5:c.G610A:p.A204T,BRSK2:NM_001282218:exon5:c.G292A:p.A98T,BRSK2:NM_003957:exon5:c.G472A:p.A158T	.	1.988	.	.	6.98E-06	Fam14_f_m_aM_aM	0/0;0/0;0/0;0/1
chr11	1454559	1454560	AC	-	BRSK2	exonic	frameshift deletion	BRSK2:NM_001256627:exon16:c.1619_1620del:p.D540Efs*9,BRSK2:NM_001256629:exon16:c.1619_1620del:p.D540Efs*9,BRSK2:NM_001256630:exon16:c.1757_1758del:p.D586Efs*9,BRSK2:NM_001282218:exon16:c.1439_1440del:p.D480Efs*9,BRSK2:NM_003957:exon16:c.1619_1620del:p.D540Efs*9	0.297	.	.	.	.	Fam32_f_m_aM_uM	0/0;0/0;0/1;0/0
chr11	1459237	1459239	GTG	-	BRSK2	exonic	stopgain	BRSK2:NM_001256627:exon19:c.1985_1987del:p.G663del,BRSK2:NM_001256630:exon19:c.2123_2125del:p.C708_D709delinsY,BRSK2:NM_003957:exon19:c.1985_1987del:p.C662_S668del	0.297	.	.	3.27E-05	.	Fam5_f_m_aM	0/0;0/1;0/1
chr11	1563929	1563929	C	T	DUSP8	exonic	nonsynonymous SNV	DUSP8:NM_004420:exon3:c.G292A:p.V98M	.	2.011	.	.	6.98E-06	Fam15_f_m_aM_aM	0/1;0/0;0/1;0/0
chr11	1565654	1565654	C	T	DUSP8	exonic	nonsynonymous SNV	DUSP8:NM_004420:exon2:c.G173A:p.R58Q	.	2.267	0.0003	0.0001	4.19E-05	Fam102_f_m_aM	0/0;0/1;0/1
chr11	1932480	1932480	C	T	TNNT3	exonic	nonsynonymous SNV	TNNT3:NM_001367850:exon6:c.C80T:p.P27L,TNNT3:NM_001367844:exon7:c.C113T:p.P38L,TNNT3:NM_001367849:exon7:c.C125T:p.P42L,TNNT3:NM_001042780:exon8:c.C113T:p.P38L,TNNT3:NM_001042782:exon8:c.C113T:p.P38L,TNNT3:NM_001367842:exon8:c.C131T:p.P44L,TNNT3:NM_001367843:exon8:c.C131T:p.P44L,TNNT3:NM_001367845:exon8:c.C113T:p.P38L,TNNT3:NM_001042781:exon9:c.C131T:p.P44L,TNNT3:NM_001297646:exon9:c.C113T:p.P38L,TNNT3:NM_001367847:exon9:c.C146T:p.P49L,TNNT3:NM_001367848:exon9:c.C134T:p.P45L,TNNT3:NM_006757:exon9:c.C137T:p.P46L,TNNT3:NM_001363561:exon10:c.C146T:p.P49L,TNNT3:NM_001367846:exon11:c.C170T:p.P57L	.	1.086	.	.	.	Fam97_f_m_aM_aF	0/0;0/1;0/1;0/0
chr11	2270184	2270184	G	C	ASCL2	exonic	nonsynonymous SNV	ASCL2:NM_005170:exon1:c.C149G:p.A50G	.	2.237	.	7.63E-05	2.80E-05	Fam38_f_m_aM	0/0;0/1;0/1
chr11	3823950	3823950	G	A	PGAP2	exonic	nonsynonymous SNV	PGAP2:NM_001256239:exon3:c.G233A:p.R78H,PGAP2:NM_001256240:exon3:c.G233A:p.R78H,PGAP2:NM_001145438:exon4:c.G404A:p.R135H,PGAP2:NM_001256237:exon4:c.G404A:p.R135H,PGAP2:NM_001256238:exon4:c.G233A:p.R78H,PGAP2:NM_001283038:exon4:c.G404A:p.R135H,PGAP2:NM_001346397:exon4:c.G386A:p.R129H,PGAP2:NM_001346398:exon4:c.G233A:p.R78H,PGAP2:NM_001346400:exon4:c.G233A:p.R78H,PGAP2:NM_001346404:exon4:c.G233A:p.R78H,PGAP2:NM_001346405:exon4:c.G233A:p.R78H,PGAP2:NM_014489:exon4:c.G416A:p.R139H,PGAP2:NM_001256235:exon5:c.G287A:p.R96H,PGAP2:NM_001256236:exon5:c.G587A:p.R196H,PGAP2:NM_001346402:exon5:c.G353A:p.R118H,PGAP2:NM_001346403:exon5:c.G416A:p.R139H	.	1.287	.	6.55E-05	1.40E-05	Fam78_f_m_aF_uM	0/0;0/1;0/1;0/1
chr11	4091584	4091584	G	A	STIM1	exonic	nonsynonymous SNV	STIM1:NM_001277961:exon12:c.G2162A:p.R721H,STIM1:NM_003156:exon12:c.G1844A:p.R615H	.	1.415	0.0006	0.0002	5.58E-05	Fam37_f_m_aF_uM	0/1;0/0;0/1;0/0
chr11	6217802	6217802	C	T	FAM160A2	exonic	nonsynonymous SNV	FAM160A2:NM_001098794:exon9:c.G1784A:p.R595H,FAM160A2:NM_032127:exon9:c.G1826A:p.R609H	.	1.198	.	7.47E-05	7.01E-06	Fam117_f_m_aM_aF	0/1;0/0;0/1;0/1
chr11	6269797	6269797	C	T	CCKBR	exonic	nonsynonymous SNV	CCKBR:NM_001363552:exon2:c.C280T:p.L94F,CCKBR:NM_176875:exon2:c.C280T:p.L94F	.	1.033	.	2.23E-05	.	Fam68_f_m_aF_uF_uM	0/0;0/1;0/1;0/1;0/1

chr11	6402113	6402113	G	A	APBB1	exonic	nonsynonymous SNV	APBB1:NM_001257319:exon7:c.C691T:p.R231C,APBB1:NM_001257320:exon7:c.C574T:p.R192C,APBB1:NM_001257323:exon7:c.C691T:p.R231C,APBB1:NM_001257325:exon7:c.C646T:p.R216C,APBB1:NM_001257326:exon7:c.C574T:p.R192C,APBB1:NM_001164:exon8:c.C1351T:p.R451C,APBB1:NM_001257321:exon8:c.C574T:p.R192C,APBB1:NM_145689:exon8:c.C1351T:p.R451C	.	1.225	.	0.0002	6.98E-05	Fam98_f_m_aM	0/1;0/0;0/1
chr11	6456425	6456425	C	T	TRIM3	exonic	nonsynonymous SNV	TRIM3:NM_001248007:exon5:c.G944A:p.R315H,TRIM3:NM_001248006:exon6:c.G1301A:p.R434H,TRIM3:NM_033278:exon6:c.G1301A:p.R434H,TRIM3:NM_006458:exon7:c.G1301A:p.R434H	.	2.443	.	4.46E-05	2.79E-05	Fam92_f_m_aM_raM	0/1;0/0;0/1;0/0
chr11	6458261	6458261	G	A	TRIM3	exonic	nonsynonymous SNV	TRIM3:NM_001248006:exon3:c.C167T:p.T56M,TRIM3:NM_033278:exon3:c.C167T:p.T56M,TRIM3:NM_006458:exon4:c.C167T:p.T56M	.	2.221	.	.	6.98E-06	Fam66_f_m_aM	0/0;0/1;0/1
chr11	6630176	6630176	T	C	DCHS1	exonic	nonsynonymous SNV	DCHS1:NM_003737:exon10:c.A4618G:p.N1540D	.	2.825	.	.	.	Fam88_f_m_aF	0/1;0/0;0/1
chr11	6632580	6632580	G	A	DCHS1	exonic	nonsynonymous SNV	DCHS1:NM_003737:exon6:c.C2932T:p.R978C	.	1.095	0.0003	0.0005	0.0002	Fam26_f_m_aM_uM	0/0;0/1;0/1;0/1
chr11	6640838	6640838	C	T	DCHS1	exonic	nonsynonymous SNV	DCHS1:NM_003737:exon2:c.G776A:p.R259H	.	1.102	.	7.46E-05	6.98E-06	Fam89_f_m_aM	0/0;0/1;0/1
chr11	10502734	10502734	A	C	AMPD3	exonic	nonsynonymous SNV	AMPD3:NM_001172431:exon12:c.A1379C:p.Q460P,AMPD3:NM_000480:exon13:c.A1883C:p.Q628P,AMPD3:NM_001025389:exon13:c.A1856C:p.Q619P,AMPD3:NM_001025390:exon13:c.A1877C:p.Q626P,AMPD3:NM_001172430:exon13:c.A1856C:p.Q619P	.	1.038	.	1.12E-05	1.40E-05	Fam35_f_m_aF_uM	0/1;0/0;0/1;0/0
chr11	10772564	10772564	G	A	CTR9	exonic	nonsynonymous SNV	CTR9:NM_001346279:exon19:c.G2417A:p.R806Q,CTR9:NM_014633:exon20:c.G2489A:p.R830Q	.	2.284	.	.	.	Fam77_f_m_aM_aF_uM_uFs	0/1;0/0;0/1;0/0;0/1;0/0
chr11	11332792	11332792	C	T	GALNT18	exonic	nonsynonymous SNV	GALNT18:NM_001363464:exon7:c.G1132A:p.A378T,GALNT18:NM_198516:exon8:c.G1318A:p.A440T	.	1.047	.	4.47E-05	3.49E-05	Fam12_f_m_aM_uM_aM	0/0;0/1;0/1;0/1;0/1
chr11	12008428	12008428	C	A	DKK3	exonic	nonsynonymous SNV	DKK3:NM_001018057:exon1:c.G155T:p.R52L,DKK3:NM_001330220:exon2:c.G155T:p.R52L,DKK3:NM_013253:exon2:c.G155T:p.R52L,DKK3:NM_015881:exon2:c.G155T:p.R52L	.	1.192	.	.	6.98E-06	Fam69_f_m_aM	0/1;0/0;0/1
chr11	12162246	12162246	G	A	MICAL2	exonic	nonsynonymous SNV	MICAL2:NM_001282666:exon1:c.G91A:p.A31T,MICAL2:NM_001282667:exon1:c.G91A:p.A31T,MICAL2:NM_001282664:exon2:c.G91A:p.A31T,MICAL2:NM_001282665:exon2:c.G91A:p.A31T,MICAL2:NM_001346292:exon2:c.G91A:p.A31T,MICAL2:NM_001346294:exon2:c.G91A:p.A31T,MICAL2:NM_001346298:exon2:c.G91A:p.A31T,MICAL2:NM_001282663:exon3:c.G91A:p.A31T,MICAL2:NM_001282668:exon3:c.G91A:p.A31T,MICAL2:NM_001346293:exon3:c.G91A:p.A31T,MICAL2:NM_001346295:exon3:c.G91A:p.A31T,MICAL2:NM_001346296:exon3:c.G91A:p.A31T,MICAL2:NM_001346297:exon3:c.G91A:p.A31T,MICAL2:NM_001346299:exon3:c.G91A:p.A31T,MICAL2:NM_014632:exon3:c.G91A:p.A31T	.	1.386	0.0004	0.0006	0.0006	Fam77_f_m_aM_aF_uM_uFs	0/0;0/1;0/1;0/0;0/0;0/1
chr11	12162246	12162246	G	A	MICAL2	exonic	nonsynonymous SNV	MICAL2:NM_001282666:exon1:c.G91A:p.A31T,MICAL2:NM_001282667:exon1:c.G91A:p.A31T,MICAL2:NM_001282664:exon2:c.G91A:p.A31T,MICAL2:NM_001282665:exon2:c.G91A:p.A31T,MICAL2:NM_001346292:exon2:c.G91A:p.A31T,MICAL2:NM_001346294:exon2:c.G91A:p.A31T,MICAL2:NM_001346298:exon2:c.G91A:p.A31T,MICAL2:NM_001282663:exon3:c.G91A:p.A31T,MICAL2:NM_001282668:exon3:c.G91A:p.A31T,MICAL2:NM_001346293:exon3:c.G91A:p.A31T,MICAL2:NM_001346295:exon3:c.G91A:p.A31T,MICAL2:NM_001346296:exon3:c.G91A:p.A31T,MICAL2:NM_001346297:exon3:c.G91A:p.A31T,MICAL2:NM_001346299:exon3:c.G91A:p.A31T,MICAL2:NM_014632:exon3:c.G91A:p.A31T	.	1.386	0.0004	0.0006	0.0006	Fam40_f_m_aM_aM	0/1;0/0;0/1;0/1
chr11	14294515	14294515	T	C	RRAS2	exonic	nonsynonymous SNV	RRAS2:NM_001102669:exon4:c.A133G:p.M45V,RRAS2:NM_001177314:exon4:c.A259G:p.M87V,RRAS2:NM_001177315:exon4:c.A133G:p.M45V,RRAS2:NM_012250:exon4:c.A364G:p.M122V	.	1.041	.	1.15E-05	.	Fam66_f_m_aM	0/0;0/1;0/1
chr11	17719844	17719844	G	C	MYOD1	exonic	nonsynonymous SNV	MYOD1:NM_002478:exon1:c.G62C:p.C21S	.	1.49	0.0002	0.0003	0.0001	Fam118_f_m_aM_aF_uM	0/0;0/1;0/0;0/1;0/0
chr11	18732615	18732615	G	A	PTPN5	exonic	nonsynonymous SNV	PTPN5:NM_001278238:exon11:c.C1234T:p.R412W,PTPN5:NM_001278239:exon11:c.C1138T:p.R380W,PTPN5:NM_001039970:exon12:c.C1210T:p.R404W,PTPN5:NM_001278236:exon12:c.C1210T:p.R404W,PTPN5:NM_006906:exon12:c.C1306T:p.R436W,PTPN5:NM_032781:exon12:c.C1306T:p.R436W	.	1.018	7.34E-05	0.0001	3.49E-05	Fam42_f_m_aM_uF	0/0;0/1;0/1;0/1
chr11	19224693	19224693	G	A	E2F8	exonic	stopgain	E2F8:NM_001256371:exon13:c.C2569T:p.R857X,E2F8:NM_001256372:exon13:c.C2569T:p.R857X,E2F8:NM_024680:exon13:c.C2569T:p.R857X	0.539	.	.	0.0002	9.77E-05	Fam118_f_m_aM_aF_uM	0/0;0/1;0/0;0/1;0/0
chr11	20083059	20083059	C	T	NAV2	exonic	nonsynonymous SNV	NAV2:NM_001111019:exon16:c.C2570T:p.A857V,NAV2:NM_001111018:exon26:c.C5186T:p.A1729V,NAV2:NM_145117:exon26:c.C5378T:p.A1793V,NAV2:NM_182964:exon26:c.C5387T:p.A1796V,NAV2:NM_001244963:exon28:c.C5555T:p.A1852V	.	1.244	.	3.35E-05	6.98E-06	Fam26_f_m_aM_uM	0/1;0/0;0/1;0/1
chr11	20101014	20101014	C	A	NAV2	exonic	nonsynonymous SNV	NAV2:NM_001111019:exon22:c.C3451A:p.L1151I,NAV2:NM_001111018:exon32:c.C6067A:p.L2023I,NAV2:NM_145117:exon32:c.C6259A:p.L2087I,NAV2:NM_182964:exon32:c.C6268A:p.L2090I,NAV2:NM_001244963:exon34:c.C6436A:p.L2146I	.	1.286	.	.	.	Fam26_f_m_aM_uM	0/1;0/0;0/1;0/1

chr11	31806404	31806404	T	C	PAX6	exonic	nonsynonymous SNV	PAX6:NM_001310159:exon2:c.A8G:p.N3S,PAX6:NM_001258465:exon3:c.A8G:p.N3S,PAX6:NM_001368889:exon3:c.A8G:p.N3S,PAX6:NM_001368891:exon3:c.A8G:p.N3S,PAX6:NM_001368892:exon3:c.A8G:p.N3S,PAX6:NM_001368913:exon3:c.A8G:p.N3S,PAX6:NM_001368916:exon3:c.A8G:p.N3S,PAX6:NM_001368918:exon3:c.A8G:p.N3S,PAX6:NM_001368920:exon3:c.A8G:p.N3S,PAX6:NM_001368924:exon3:c.A8G:p.N3S,PAX6:NM_001368925:exon3:c.A8G:p.N3S,PAX6:NM_001368928:exon3:c.A8G:p.N3S,PAX6:NM_000280:exon4:c.A8G:p.N3S,PAX6:NM_001127612:exon4:c.A8G:p.N3S,PAX6:NM_001258462:exon4:c.A8G:p.N3S,PAX6:NM_001258463:exon4:c.A8G:p.N3S,PAX6:NM_001258464:exon4:c.A8G:p.N3S,PAX6:NM_001310158:exon4:c.A8G:p.N3S,PAX6:NM_001368887:exon4:c.A8G:p.N3S,PAX6:NM_001368888:exon4:c.A8G:p.N3S,PAX6:NM_001368890:exon4:c.A8G:p.N3S,PAX6:NM_001368894:exon4:c.A8G:p.N3S,PAX6:NM_001368912:exon4:c.A8G:p.N3S,PAX6:NM_001368914:exon4:c.A8G:p.N3S,PAX6:NM_001368915:exon4:c.A8G:p.N3S,PAX6:NM_001368917:exon4:c.A8G:p.N3S,PAX6:NM_001368919:exon4:c.A8G:p.N3S,PAX6:NM_001368921:exon4:c.A8G:p.N3S,PAX6:NM_001368922:exon4:c.A8G:p.N3S,PAX6:NM_001368923:exon4:c.A8G:p.N3S,PAX6:NM_001368926:exon4:c.A8G:p.N3S,PAX6:NM_001368927:exon4:c.A8G:p.N3S,PAX6:NM_001604:exon4:c.A8G:p.N3S,PAX6:NM_001368893:exon5:c.A8G:p.N3S	.	1.501	.	.	.	Fam122_f_m_aM	0/1;0/0;0/1
chr11	33141645	33141645	-	AAAA	CSTF3	exonic	frameshift insertion	CSTF3:NM_001033505:exon3:c.246_247insATTTT:p.F83Ifs*21	0.377	.	0.0002	0.0003	0.0003	Fam49_f_m_aM	0/0;0/1;0/1
chr11	33751545	33751545	T	G	FBXO3	exonic	nonsynonymous SNV	FBXO3:NM_012175:exon7:c.A787C:p.I263L,FBXO3:NM_033406:exon7:c.A787C:p.I263L	.	1.526	.	0.0001	.	Fam27_f_m_aF_uM_uM	0/0;0/1;0/1;0/0;0/0
chr11	33751545	33751545	T	G	FBXO3	exonic	nonsynonymous SNV	FBXO3:NM_012175:exon7:c.A787C:p.I263L,FBXO3:NM_033406:exon7:c.A787C:p.I263L	.	1.526	.	0.0001	.	Fam59_f_m_aF_uM	0/1;0/0;0/1;0/1
chr11	34883386	34883386	G	A	APIP	exonic	nonsynonymous SNV	APIP:NM_015957:exon6:c.C580T:p.R194C	.	1.244	.	2.24E-05	2.10E-05	Fam102_f_m_aM	0/0;0/1;0/1
chr11	36462525	36462525	T	A	PRR5L	exonic	nonsynonymous SNV	PRR5L:NM_001160168:exon6:c.T512A:p.I171N,PRR5L:NM_001160167:exon9:c.T896A:p.I299N,PRR5L:NM_024841:exon10:c.T896A:p.I299N	.	1.283	.	1.18E-05	.	Fam72_f_m_aF_uF	0/1;0/0;0/1;0/0
chr11	40115766	40115766	C	G	LRRC4C	exonic	nonsynonymous SNV	LRRC4C:NM_020929:exon5:c.G527C:p.R176P,LRRC4C:NM_001258419:exon7:c.G527C:p.R176P	.	1.732	.	.	.	Fam103_f_m_aM	0/0;0/1;0/1
chr11	40319722	40319722	C	T	LRRC4C	splicing	.	.	0.297	.	.	.	1.40E-05	Fam7_f_m_aM_aM_uM	0/1;0/0;0/1;0/1;0/0
chr11	45650489	45650489	G	T	CHST1	exonic	nonsynonymous SNV	CHST1:NM_003654:exon4:c.C435A:p.N145K	.	1.89	.	.	.	Fam92_f_m_aM_raM	0/0;0/0;0/0;0/1
chr11	45650659	45650659	G	T	CHST1	exonic	nonsynonymous SNV	CHST1:NM_003654:exon4:c.C265A:p.L89M	.	2.095	.	2.23E-05	.	Fam29_f_m_aF_uM	0/1;0/0;0/1;0/0
chr11	46542621	46542621	C	T	AMBRA1	exonic	nonsynonymous SNV	AMBRA1:NM_001300731:exon7:c.G1396A:p.A466T,AMBRA1:NM_001367468:exon7:c.G1396A:p.A466T,AMBRA1:NM_001267782:exon8:c.G1126A:p.A376T,AMBRA1:NM_001267783:exon8:c.G1126A:p.A376T,AMBRA1:NM_001367469:exon8:c.G1126A:p.A376T,AMBRA1:NM_001367470:exon8:c.G589A:p.A197T,AMBRA1:NM_001367471:exon8:c.G1126A:p.A376T,AMBRA1:NM_017749:exon8:c.G1126A:p.A376T	.	1.418	.	.	.	Fam27_f_m_aF_uM_uM	0/0;0/1;0/1;0/0;0/1
chr11	46739293	46739293	T	C	F2	exonic	nonsynonymous SNV	F2:NM_000506:exon14:c.T1754C:p.M585T,F2:NM_001311257:exon14:c.T1706C:p.M569T	.	1.483	0.0006	0.0001	6.28E-05	Fam95_f_m_aM_aM_uF	0/1;0/0;0/1;0/1;0/1
chr11	46899461	46899461	C	A	LRP4	exonic	nonsynonymous SNV	LRP4:NM_002334:exon5:c.G473T:p.G158V	.	1.677	.	.	.	Fam61_f_m_aM	0/0;0/1;0/1
chr11	47175289	47175289	A	G	ARFGAP2	exonic	nonsynonymous SNV	ARFGAP2:NM_001242832:exon4:c.T289C:p.C97R,ARFGAP2:NM_032389:exon4:c.T289C:p.C97R	.	1.053	0.0002	6.70E-05	4.19E-05	Fam87_f_m_aM_uM	0/0;0/1;0/1;0/0
chr11	47267933	47267933	A	C	NR1H3	exonic	nonsynonymous SNV	NR1H3:NM_001363595:exon6:c.A694C:p.N232H,NR1H3:NM_001130101:exon7:c.A829C:p.N277H,NR1H3:NM_001130102:exon7:c.A874C:p.N292H,NR1H3:NM_001251934:exon8:c.A1027C:p.N343H,NR1H3:NM_001251935:exon8:c.A1027C:p.N343H,NR1H3:NM_005693:exon8:c.A1009C:p.N337H	.	1.343	7.34E-05	0.0001	0.0002	Fam61_f_m_aM	0/1;0/0;0/1
chr11	47276780	47276780	G	A	MADD	exonic	nonsynonymous SNV	MADD:NM_001135943:exon5:c.G1012A:p.A338T,MADD:NM_001135944:exon5:c.G1012A:p.A338T,MADD:NM_003682:exon5:c.G1012A:p.A338T,MADD:NM_130470:exon5:c.G1012A:p.A338T,MADD:NM_130471:exon5:c.G1012A:p.A338T,MADD:NM_130472:exon5:c.G1012A:p.A338T,MADD:NM_130473:exon5:c.G1012A:p.A338T,MADD:NM_130474:exon5:c.G1012A:p.A338T,MADD:NM_130475:exon5:c.G1012A:p.A338T,MADD:NM_130476:exon5:c.G1012A:p.A338T	.	1.1	.	.	.	Fam121_f_m_aF_aM	0/0;0/1;0/1;0/1
chr11	47419145	47419145	C	T	PSMC3	exonic	nonsynonymous SNV	PSMC3:NM_002804:exon11:c.G1180A:p.A394T	.	2.082	.	.	.	Fam106_f_m_aM	0/0;0/0;0/1
chr11	47499490	47499490	T	C	CELF1	exonic	nonsynonymous SNV	CELF1:NM_001172639:exon3:c.A34G:p.M12V,CELF1:NM_001330272:exon3:c.A34G:p.M12V	.	1.027	.	4.64E-05	1.40E-05	Fam33_f_m_aM_uM	0/0;0/1;0/1;0/1
chr11	47808452	47808456	ATAA	-	NUP160	exonic	frameshift deletion	NUP160:NM_015231:exon18:c.2315_2319del:p.S772fs	0.596	.	.	1.12E-05	1.40E-05	Fam86_f_m_aF	0/1;0/0;0/1
chr11	57659981	57659981	G	A	CLP1	exonic	nonsynonymous SNV	CLP1:NM_006831:exon2:c.G505A:p.E169K	.	1.206	.	.	.	Fam25_f_m_aM	0/1;0/0;0/1
chr11	57661062	57661062	C	G	CLP1	exonic	nonsynonymous SNV	CLP1:NM_001142597:exon3:c.C712G:p.R238G,CLP1:NM_006831:exon3:c.C904G:p.R302G	.	1.783	.	.	.	Fam22_f_m_aF_aF_uF	0/0;0/1;0/1;0/1;0/0

chr11	57801881	57801881	C	T	CTNND1	exonic	nonsynonymous SNV	CTNND1:NM_001085469:exon4:c.C802T:p.P268S,CTNND1:NM_001085463:exon5:c.C802T:p.P268S,CTNND1:NM_001085464:exon5:c.C802T:p.P268S,CTNND1:NM_001085465:exon5:c.C802T:p.P268S,CTNND1:NM_001085466:exon5:c.C802T:p.P268S,CTNND1:NM_001085467:exon5:c.C802T:p.P268S,CTNND1:NM_001085468:exon5:c.C802T:p.P268S,CTNND1:NM_001206890:exon5:c.C802T:p.P268S,CTNND1:NM_001085462:exon6:c.C1105T:p.P369S,CTNND1:NM_001206883:exon6:c.C943T:p.P315S,CTNND1:NM_001206884:exon6:c.C943T:p.P315S,CTNND1:NM_001206886:exon6:c.C943T:p.P315S,CTNND1:NM_001206887:exon6:c.C943T:p.P315S,CTNND1:NM_001206888:exon6:c.C943T:p.P315S,CTNND1:NM_001085458:exon7:c.C1105T:p.P369S,CTNND1:NM_001085459:exon7:c.C1105T:p.P369S,CTNND1:NM_001085460:exon7:c.C1105T:p.P369S,CTNND1:NM_001085461:exon7:c.C1105T:p.P369S,CTNND1:NM_001206885:exon7:c.C1105T:p.P369S,CTNND1:NM_001206889:exon7:c.C943T:p.P315S,CTNND1:NM_001206891:exon7:c.C943T:p.P315S,CTNND1:NM_001331:exon7:c.C1105T:p.P369S	.	1.004	.		2.25E-05	1.40E-05	Fam13_f_m_aM	0/1;0/0;0/1
chr11	60936080	60936080	C	T	TMEM132A	exonic	nonsynonymous SNV	TMEM132A:NM_017870:exon11:c.C2248T:p.R750C,TMEM132A:NM_178031:exon11:c.C2245T:p.R749C	.	1.109	.		0.0001	.	Fam79_f_m_aM_uM	0/1;0/0;0/1;0/0
chr11	61009638	61009638	G	A	CD6	exonic	nonsynonymous SNV	CD6:NM_001254750:exon5:c.G848A:p.R283Q,CD6:NM_001254751:exon5:c.G848A:p.R283Q,CD6:NM_006725:exon5:c.G848A:p.R283Q	.	1.514	.		6.18E-05	2.79E-05	Fam12_f_m_aM_uM_aM	0/0;0/1;0/0;0/0;0/1
chr11	61325657	61325657	T	C	DDB1	exonic	nonsynonymous SNV	DDB1:NM_001923:exon6:c.A716G:p.Y239C	.	2.21	.	.	.		Fam77_f_m_aM_aF_uM_uFs	0/0;0/1;0/1;0/1;0/1;0/1
chr11	61731407	61731407	G	A	DAGLA	exonic	nonsynonymous SNV	DAGLA:NM_006133:exon9:c.G940A:p.A314T	.	1.035	.		7.45E-05	.	Fam21_f_m_aM_uM	0/0;0/1;0/1;0/0
chr11	61965497	61965497	C	T	FTH1	exonic	nonsynonymous SNV	FTH1:NM_002032:exon2:c.G133A:p.D45N	.	1.636	.	.	.		Fam24_f_m_aM_aM	0/0;0/1;0/1;0/1
chr11	62566920	62566920	C	A	EEF1G	exonic	nonsynonymous SNV	EEF1G:NM_001404:exon7:c.G743T:p.R248L	.	1.094	.	.		6.98E-06	Fam17_f_m_aM_uM	0/0;0/1;0/1;0/1
chr11	62571680	62571680	T	G	EEF1G	exonic	nonsynonymous SNV	EEF1G:NM_001404:exon4:c.A238C:p.S80R	.	1.933	.	.	.		Fam15_f_m_aM_aM	0/1;0/0;0/1;0/1
chr11	62575191	62575191	A	G	TUT1	exonic	nonsynonymous SNV	TUT1:NM_022830:exon9:c.T2528C:p.L843P	.	1.015	.	.	.		Fam71_f_m_aF	0/0;0/1;0/1
chr11	62649509	62649509	C	T	INTS5	exonic	nonsynonymous SNV	INTS5:NM_030628:exon2:c.G571A:p.D191N	.	1.125	.		4.47E-05	2.09E-05	Fam107_f_m_aM	0/0;0/1;0/1
chr11	62676909	62676909	C	T	UBXN1	exonic	nonsynonymous SNV	UBXN1:NM_001286078:exon6:c.G571A:p.E191K,UBXN1:NM_001286077:exon8:c.G748A:p.E250K,UBXN1:NM_015853:exon8:c.G748A:p.E250K	.	1.003	7.35E-05		1.12E-05	1.40E-05	Fam90_f_m_aF_dM	0/0;0/1;0/1;0/1
chr11	62752410	62752410	C	T	ZBTB3	exonic	nonsynonymous SNV	ZBTB3:NM_001363108:exon2:c.G1255A:p.E419K,ZBTB3:NM_001363109:exon2:c.G1255A:p.E419K,ZBTB3:NM_001370809:exon2:c.G1255A:p.E419K,ZBTB3:NM_024784:exon2:c.G1405A:p.E469K	.	1.142	.		3.35E-05	6.98E-06	Fam42_f_m_aM_uF	0/1;0/0;0/1;0/0
chr11	62834496	62834496	C	T	WDR74	exonic	nonsynonymous SNV	WDR74:NM_001307977:exon7:c.G650A:p.R217H,WDR74:NM_001369447:exon7:c.G692A:p.R231H,WDR74:NM_001369450:exon7:c.G650A:p.R217H,WDR74:NM_001369448:exon8:c.G245A:p.R82H,WDR74:NM_001369451:exon8:c.G650A:p.R217H,WDR74:NM_001369453:exon8:c.G650A:p.R217H,WDR74:NM_018093:exon8:c.G650A:p.R217H,WDR74:NM_001369449:exon9:c.G245A:p.R82H	.	1.247	7.36E-05		7.76E-05	1.41E-05	Fam55_f_m_aM_aM_dM	0/0;0/1;0/1;0/0;0/1
chr11	62856328	62856328	C	T	SLC3A2	exonic	nonsynonymous SNV	SLC3A2:NM_001012662:exon1:c.C59T:p.P20L,SLC3A2:NM_001012664:exon1:c.C59T:p.P20L,SLC3A2:NM_002394:exon1:c.C59T:p.P20L	.	1.408	.	.	.		Fam106_f_m_aM	0/0;0/1;0/1
chr11	62856340	62856340	C	T	SLC3A2	exonic	nonsynonymous SNV	SLC3A2:NM_001012662:exon1:c.C71T:p.S24L,SLC3A2:NM_001012664:exon1:c.C71T:p.S24L,SLC3A2:NM_002394:exon1:c.C71T:p.S24L	.	1.267	7.34E-05		7.84E-05	2.79E-05	Fam47_f_m_aM	0/1;0/0;0/1
chr11	62881346	62881346	G	C	SLC3A2	exonic	nonsynonymous SNV	SLC3A2:NM_001013251:exon1:c.G323C:p.R108P,SLC3A2:NM_001012664:exon2:c.G440C:p.R147P,SLC3A2:NM_001012662:exon4:c.G629C:p.R210P,SLC3A2:NM_002394:exon4:c.G626C:p.R209P	.	1.689	0.0006		3.59E-05	3.49E-05	Fam95_f_m_aM_aM_uF	0/0;0/1;0/1;0/1;0/0
chr11	62885185	62885185	T	C	SLC3A2	exonic	nonsynonymous SNV	SLC3A2:NM_001013251:exon6:c.T827C:p.I276T,SLC3A2:NM_001012664:exon7:c.T944C:p.I315T,SLC3A2:NM_001012662:exon9:c.T1133C:p.I378T,SLC3A2:NM_002394:exon9:c.T1130C:p.I377T	.	1.582	.		3.35E-05	6.98E-06	Fam31_f_m_aM_uF	0/0;0/1;0/1;0/0
chr11	62909824	62909824	C	T	CHRM1	exonic	nonsynonymous SNV	CHRM1:NM_000738:exon2:c.G1277A:p.R426Q	.	1.694	.		4.47E-05	.	Fam24_f_m_aM_aM	0/1;0/0;0/1;0/0
chr11	63014853	63014853	G	T	SLC22A8	exonic	nonsynonymous SNV	SLC22A8:NM_001184732:exon2:c.C106A:p.L36M,SLC22A8:NM_004254:exon2:c.C106A:p.L36M	.	1.056	7.34E-05		0.0001	4.19E-05	Fam120_f_m_aM_aM	0/1;0/0;0/1;0/1
chr11	63952522	63952522	C	T	NAA40	exonic	nonsynonymous SNV	NAA40:NM_001300800:exon5:c.C304T:p.H102Y,NAA40:NM_024771:exon5:c.C367T:p.H123Y	.	1.274	0.0001		0.0004	0.0002	Fam94_f_m_aM	0/1;0/0;0/1
chr11	64185962	64185962	G	A	STIP1	exonic	stopgain	STIP1:NM_001282652:exon1:c.G138A:p.W46X	0.306	.	.		0.0003	1.40E-05	Fam108_f_m_aM	0/0;0/1;0/1
chr11	64195749	64195749	C	A	STIP1	exonic	nonsynonymous SNV	STIP1:NM_001282652:exon5:c.C749A:p.P250H,STIP1:NM_001282653:exon5:c.C536A:p.P179H,STIP1:NM_006819:exon5:c.C608A:p.P203H	.	1.077	.		2.23E-05	6.98E-06	Fam16_f_m_aM_aM	0/0;0/1;0/1;0/1
chr11	64197918	64197918	T	C	STIP1	exonic	nonsynonymous SNV	STIP1:NM_001282652:exon8:c.T1108C:p.Y370H,STIP1:NM_001282653:exon8:c.T895C:p.Y299H,STIP1:NM_006819:exon8:c.T967C:p.Y323H	.	1.601	.		3.35E-05	6.98E-06	Fam115_f_m_aF_aM_aF	0/1;0/0;0/1;0/1;0/1

chr11	64242551	64242551	A	T	FKBP2	exonic	nonsynonymous SNV	FKBP2:NM_001135208:exon2:c.A164T:p.H55L,FKBP2:NM_001370360:exon2:c.A164T:p.H55L,FKBP2:NM_001370362:exon2:c.A164T:p.H55L,FKBP2:NM_001370363:exon2:c.A164T:p.H55L,FKBP2:NM_001370365:exon2:c.A251T:p.H84L,FKBP2:NM_001370367:exon2:c.A5T:p.H2L,FKBP2:NM_004470:exon2:c.A164T:p.H55L,FKBP2:NM_057092:exon2:c.A164T:p.H55L	.	1.665	.	.	.	Fam57_f_m_aM_uF	0/1;0/0;0/1;0/1
chr11	64262726	64262726	C	T	PLCB3	exonic	nonsynonymous SNV	PLCB3:NM_001184883:exon17:c.C2072T:p.T691M,PLCB3:NM_000932:exon19:c.C2273T:p.T758M,PLCB3:NM_001316314:exon19:c.C2273T:p.T758M	.	1.95	.	1.12E-05	6.98E-06	Fam16_f_m_aM_aM	0/0;0/1;0/1;0/1
chr11	64368504	64368504	C	T	RPS6KA4	exonic	nonsynonymous SNV	RPS6KA4:NM_001006944:exon11:c.C1219T:p.R407W,RPS6KA4:NM_001300802:exon11:c.C1216T:p.R406W,RPS6KA4:NM_001318361:exon11:c.C1048T:p.R350W,RPS6KA4:NM_003942:exon11:c.C1237T:p.R413W	.	2.035	.	1.80E-05	2.09E-05	Fam12_f_m_aM_uM_aM	0/0;0/1;0/1;0/1;0/0
chr11	64368504	64368504	C	T	RPS6KA4	exonic	nonsynonymous SNV	RPS6KA4:NM_001006944:exon11:c.C1219T:p.R407W,RPS6KA4:NM_001300802:exon11:c.C1216T:p.R406W,RPS6KA4:NM_001318361:exon11:c.C1048T:p.R350W,RPS6KA4:NM_003942:exon11:c.C1237T:p.R413W	.	2.035	.	1.80E-05	2.09E-05	Fam21_f_m_aM_uM	0/1;0/0;0/1;0/0
chr11	64770355	64770355	C	G	SF1	exonic	nonsynonymous SNV	SF1:NM_001178030:exon4:c.G665C:p.R222P,SF1:NM_001178031:exon4:c.G212C:p.R71P,SF1:NM_001346363:exon4:c.G290C:p.R97P,SF1:NM_001346364:exon4:c.G290C:p.R97P,SF1:NM_004630:exon4:c.G290C:p.R97P,SF1:NM_201995:exon4:c.G290C:p.R97P,SF1:NM_201997:exon4:c.G290C:p.R97P,SF1:NM_201998:exon4:c.G290C:p.R97P	.	2.74	.	.	.	Fam51_f_m_aM_uF	0/0;0/1;0/1;0/0
chr11	64778203	64778203	G	A	SF1	exonic	nonsynonymous SNV	SF1:NM_001178030:exon1:c.C190T:p.P64S	.	1.653	7.72E-05	.	7.80E-05	Fam62_f_m_aM_aM	0/0;0/1;0/0;0/1
chr11	64808019	64808019	C	A	MEN1	exonic	nonsynonymous SNV	MEN1:NM_000244:exon3:c.G541T:p.A181S,MEN1:NM_001370251:exon3:c.G526T:p.A176S,MEN1:NM_001370259:exon3:c.G526T:p.A176S,MEN1:NM_001370260:exon3:c.G526T:p.A176S,MEN1:NM_001370261:exon3:c.G526T:p.A176S,MEN1:NM_001370262:exon3:c.G526T:p.A176S,MEN1:NM_001370263:exon3:c.G526T:p.A176S,MEN1:NM_130799:exon3:c.G526T:p.A176S,MEN1:NM_130800:exon3:c.G541T:p.A181S,MEN1:NM_130801:exon3:c.G541T:p.A181S,MEN1:NM_130802:exon3:c.G541T:p.A181S,MEN1:NM_130803:exon3:c.G541T:p.A181S,MEN1:NM_130804:exon4:c.G541T:p.A181S	.	2.079	.	1.12E-05	1.40E-05	Fam92_f_m_aM_raM	0/1;0/0;0/1;0/1
chr11	65041339	65041339	C	T	SAC3D1	exonic	nonsynonymous SNV	SAC3D1:NM_001367486:exon1:c.C47T:p.P16L,SAC3D1:NM_001367488:exon1:c.C47T:p.P16L,SAC3D1:NM_013299:exon1:c.C47T:p.P16L,SAC3D1:NM_001367485:exon2:c.C47T:p.P16L,SAC3D1:NM_001367487:exon2:c.C47T:p.P16L	.	1.252	.	5.42E-05	6.98E-06	Fam85_f_m_aM_aM	0/1;0/0;0/1;0/1
chr11	65041527	65041527	G	A	SAC3D1	exonic	nonsynonymous SNV	SAC3D1:NM_001367486:exon1:c.G235A:p.V79M,SAC3D1:NM_001367488:exon1:c.G235A:p.V79M,SAC3D1:NM_013299:exon1:c.G235A:p.V79M,SAC3D1:NM_001367485:exon2:c.G235A:p.V79M,SAC3D1:NM_001367487:exon2:c.G235A:p.V79M	.	1.241	7.36E-05	3.66E-05	1.40E-05	Fam79_f_m_aM_uM	0/0;0/1;0/1;0/0
chr11	65088034	65088034	C	T	ZFPL1	exonic	nonsynonymous SNV	ZFPL1:NM_006782:exon8:c.C853T:p.R285C	.	1.004	.	.	6.98E-06	Fam115_f_m_aF_aM_aF	0/1;0/0;0/1;0/1;0/1
chr11	65109443	65109443	C	T	VPS51	exonic	nonsynonymous SNV	VPS51:NM_013265:exon6:c.C1607T:p.T536M	.	1.545	7.35E-05	0.0002	7.68E-05	Fam15_f_m_aM_aM	0/0;0/1;0/0;0/1
chr11	65584987	65584987	T	C	EHBP1L1	exonic	nonsynonymous SNV	EHBP1L1:NM_001351087:exon11:c.T1055C:p.V352A,EHBP1L1:NM_001099409:exon12:c.T3329C:p.V1110A	.	1.808	0.0002	0.0003	9.07E-05	Fam89_f_m_aM	0/1;0/0;0/1
chr11	65590165	65590165	C	T	EHBP1L1	exonic	nonsynonymous SNV	EHBP1L1:NM_001351087:exon14:c.C1864T:p.R622W,EHBP1L1:NM_001099409:exon15:c.C4138T:p.R1380W	.	1.692	0.0002	0.0004	9.77E-05	Fam117_f_m_aM_aF	0/0;0/1;0/1;0/0
chr11	65646775	65646775	C	G	SIPA1	exonic	nonsynonymous SNV	SIPA1:NM_006747:exon8:c.C1741G:p.L581V,SIPA1:NM_153253:exon8:c.C1741G:p.L581V	.	1.809	.	.	.	Fam114_f_m_aM	0/0;0/1;0/1
chr11	65647531	65647531	C	T	SIPA1	exonic	nonsynonymous SNV	SIPA1:NM_006747:exon9:c.C2179T:p.R727C,SIPA1:NM_153253:exon9:c.C2179T:p.R727C	.	3	.	.	.	Fam61_f_m_aM	0/0;0/1;0/1
chr11	65720349	65720349	C	G	RNASEH2C	exonic	nonsynonymous SNV	RNASEH2C:NM_032193:exon2:c.G241C:p.G81R	.	1.355	.	.	.	Fam90_f_m_aF_dM	0/0;0/1;0/1;0/1
chr11	65961910	65961910	G	A	SART1	exonic	nonsynonymous SNV	SART1:NM_005146:exon1:c.G130A:p.G44S	.	2.5	.	.	.	Fam28_f_m_aF_uF	0/0;0/1;0/1;0/1
chr11	66013323	66013323	C	T	CST6	exonic	nonsynonymous SNV	CST6:NM_001323:exon3:c.C373T:p.R125C	.	1.467	.	9.83E-05	4.89E-05	Fam121_f_m_aF_aM	0/0;0/1;0/1;0/1
chr11	66063530	66063530	C	A	SF3B2	exonic	nonsynonymous SNV	SF3B2:NM_006842:exon18:c.C2216A:p.T739N	.	2.155	.	.	.	Fam122_f_m_aM	0/0;0/1;0/1
chr11	66266103	66266103	G	C	KLC2	exonic	nonsynonymous SNV	KLC2:NM_001134774:exon13:c.G1382C:p.G461A,KLC2:NM_001134775:exon14:c.G1613C:p.G538A,KLC2:NM_001134776:exon14:c.G1613C:p.G538A,KLC2:NM_001318734:exon14:c.G1613C:p.G538A,KLC2:NM_022822:exon14:c.G1613C:p.G538A	.	1.433	.	3.28E-05	.	Fam36_f_m_aM_uM	0/1;0/0;0/1;0/0
chr11	66282286	66282286	G	A	CNIH2	exonic	nonsynonymous SNV	CNIH2:NM_182553:exon2:c.G109A:p.D37N	.	2.459	.	1.13E-05	6.98E-06	Fam47_f_m_aM	0/1;0/0;0/1
chr11	66644124	66644124	G	A	RBM14-RBM4;RBI	exonic	nonsynonymous SNV	RBM14-RBM4:NM_001198845:exon2:c.G1012A:p.A338T,RBM4:NM_002896:exon3:c.G1087A:p.A363T	.	1.3	.	1.14E-05	.	Fam9_f_m_aM_dM_uF	0/1;0/0;0/1;0/0;0/0
chr11	66845018	66845018	C	G	RCE1	exonic	nonsynonymous SNV	RCE1:NM_001032279:exon5:c.C289G:p.P97A,RCE1:NM_005133:exon5:c.C601G:p.P201A	.	1.378	.	.	.	Fam68_f_m_aF_uF_uM	0/0;0/1;0/1;0/0;0/1
chr11	66845206	66845206	G	C	RCE1	exonic	nonsynonymous SNV	RCE1:NM_001032279:exon6:c.G348C:p.Q116H,RCE1:NM_005133:exon6:c.G660C:p.Q220H	.	1.099	.	.	.	Fam73_f_m_aF_dM	0/1;0/0;0/1;0/0
chr11	66858165	66858165	G	A	LRFN4	exonic	nonsynonymous SNV	LRFN4:NM_024036:exon1:c.G421A:p.D141N,LRFN4:NM_001363524:exon2:c.G421A:p.D141N	.	1.845	.	.	.	Fam9_f_m_aM_dM_uF	0/0;0/1;0/1;0/1;0/1
chr11	66870337	66870337	G	A	PC	exonic	nonsynonymous SNV	PC:NM_022172:exon7:c.C868T:p.R290W,PC:NM_000920:exon8:c.C868T:p.R290W,PC:NM_001040716:exon9:c.C868T:p.R290W	.	1.789	7.36E-05	2.26E-05	1.40E-05	Fam74_f_m_aM_uM	0/1;0/0;0/1;0/0

chr11	67283739	67283739	G	T	GRK2	exonic	nonsynonymous SNV	GRK2:NM_001619:exon16:c.G1361T:p.R454L		.	1.079	.		6.72E-05	.	Fam75_f_m_aM	0/1;0/0;0/1
chr11	67290442	67290442	G	A	ANKRD13D	exonic	nonsynonymous SNV	ANKRD13D:NM_207354:exon3:c.G347A:p.R116H,ANKRD13D:NM_001347901:exon4:c.G86A:p.R29H		.	1.337	.		8.50E-05	1.40E-05	Fam79_f_m_aM_uM	0/0;0/1;0/1;0/0
chr11	67365588	67365588	G	A	CLCF1	exonic	nonsynonymous SNV	CLCF1:NM_001166212:exon3:c.C196T:p.P66S,CLCF1:NM_013246:exon3:c.C226T:p.P76S		.	1.114	.	.		1.40E-05	Fam66_f_m_aM	0/0;0/1;0/1
chr11	67451967	67451967	T	C	GPR152	exonic	nonsynonymous SNV	GPR152:NM_206997:exon1:c.A758G:p.Y253C		.	1.079	.		3.38E-05	.	Fam81_f_m_aM_uM	0/0;0/1;0/1;0/0
chr11	67451967	67451967	T	C	GPR152	exonic	nonsynonymous SNV	GPR152:NM_206997:exon1:c.A758G:p.Y253C		.	1.079	.		3.38E-05	.	Fam84_f_m_aF	0/1;0/0;0/1
chr11	67493965	67493965	G	A	PITPNM1	exonic	nonsynonymous SNV	PITPNM1:NM_001130848:exon20:c.C2962T:p.R988C,PITPNM1:NM_004910:exon20:c.C2965T:p.R989C		.	1.775	7.34E-05		3.45E-05	6.98E-06	Fam40_f_m_aM_aM	0/1;0/0;0/1;0/1
chr11	67497324	67497324	G	A	PITPNM1	exonic	nonsynonymous SNV	PITPNM1:NM_001130848:exon14:c.C2053T:p.R685C,PITPNM1:NM_004910:exon14:c.C2053T:p.R685C		.	1.215	.		7.98E-05	2.79E-05	Fam65_f_m_aM_uF_dF	0/1;0/0;0/1;0/0;0/0
chr11	68213475	68213475	A	G	KMT5B	splicing	.	.	0.19	.		.	.		7.36E-06	Fam12_f_m_aM_uM_aM	0/1;0/0;0/1;0/1;0/0
chr11	68438611	68438611	A	G	LRP5	exonic	nonsynonymous SNV	LRP5:NM_001291902:exon20:c.A2534G:p.Y845C,LRP5:NM_002335:exon20:c.A4277G:p.Y1426C		.	1.237	.		1.13E-05	.	Fam89_f_m_aM	0/1;0/0;0/1
chr11	68761621	68761621	T	C	CPT1A	exonic	nonsynonymous SNV	CPT1A:NM_001031847:exon16:c.A1942G:p.M648V,CPT1A:NM_001876:exon16:c.A1942G:p.M648V		.	1.164	.	.		.	Fam55_f_m_aM_aM_dM	0/0;0/1;0/1;0/0;0/1
chr11	68761624	68761624	C	T	CPT1A	exonic	nonsynonymous SNV	CPT1A:NM_001031847:exon16:c.G1939A:p.A647T,CPT1A:NM_001876:exon16:c.G1939A:p.A647T		.	1.19	.		3.35E-05	6.98E-06	Fam79_f_m_aM_uM	0/0;0/1;0/1;0/0
chr11	69005634	69005634	A	G	MRGPRF	exonic	nonsynonymous SNV	MRGPRF:NM_001098515:exon3:c.T676C:p.C226R,MRGPRF:NM_145015:exon3:c.T676C:p.C226R		.	1.746	.	.		6.98E-06	Fam5_f_m_aM	0/0;0/1;0/1
chr11	69005846	69005846	C	T	MRGPRF	exonic	nonsynonymous SNV	MRGPRF:NM_001098515:exon3:c.G464A:p.R155Q,MRGPRF:NM_145015:exon3:c.G464A:p.R155Q		.	1.382	7.35E-05		4.11E-05	2.09E-05	Fam37_f_m_aF_uM	0/1;0/0;0/1;0/0
chr11	70485650	70485650	G	T	SHANK2	exonic	nonsynonymous SNV	SHANK2:NM_133266:exon10:c.C2879A:p.P960H,SHANK2:NM_012309:exon24:c.C4643A:p.P1548H		.	1.346	.	.		.	Fam14_f_m_aM_aM	0/1;0/0;0/0;0/1
chr11	72004298	72004298	C	T	NUMA1	exonic	nonsynonymous SNV	NUMA1:NM_001286561:exon25:c.G6008A:p.R2003Q,NUMA1:NM_006185:exon25:c.G6050A:p.R2017Q		.	1.053	.		9.81E-05	1.40E-05	Fam123_f_aF	0/1;0/1
chr11	72006074	72006074	G	A	NUMA1	exonic	nonsynonymous SNV	NUMA1:NM_001286561:exon22:c.C5611T:p.R1871C,NUMA1:NM_006185:exon22:c.C5653T:p.R1885C		.	1.15	0.0003		0.0004	0.0001	Fam110_f_m_aM_aM_uMs	0/0;0/1;0/0;0/1;0/0
chr11	72007230	72007230	G	A	NUMA1	exonic	nonsynonymous SNV	NUMA1:NM_001286561:exon21:c.C5380T:p.R1794C,NUMA1:NM_006185:exon21:c.C5422T:p.R1808C		.	1.101	.		6.73E-05	2.79E-05	Fam23_f_m_aM_dF_uFs	0/0;0/1;0/1;0/0;0/0
chr11	72013241	72013241	C	T	NUMA1	exonic	nonsynonymous SNV	NUMA1:NM_006185:exon15:c.G4262A:p.R1421Q,NUMA1:NM_001286561:exon16:c.G4262A:p.R1421Q		.	1.054	.		1.13E-05	.	Fam28_f_m_aF_uF	0/0;0/1;0/1;0/1
chr11	72293398	72293398	A	G	CLPB	exonic	nonsynonymous SNV	CLPB:NM_001258393:exon15:c.T1916C:p.L639P,CLPB:NM_001258392:exon16:c.T2003C:p.L668P,CLPB:NM_030813:exon17:c.T2093C:p.L698P,CLPB:NM_001258394:exon18:c.T1958C:p.L653P		.	1.404	.	.		.	Fam45_f_m_aM_uF	0/1;0/0;0/1;0/0
chr11	72705855	72705855	C	T	ARAP1	exonic	nonsynonymous SNV	ARAP1:NM_001135190:exon11:c.G1024A:p.V342M,ARAP1:NM_001369489:exon11:c.G1024A:p.V342M,ARAP1:NM_015242:exon11:c.G1024A:p.V342M,ARAP1:NM_001040118:exon13:c.G1759A:p.V587M		.	1.06	7.35E-05		0.0002	9.07E-05	Fam44_f_m_aM_uF	0/1;0/0;0/1;0/0
chr11	73296697	73296697	G	T	P2RY6	exonic	nonsynonymous SNV	P2RY6:NM_001277208:exon2:c.G482T:p.R161L,P2RY6:NM_001277204:exon3:c.G179T:p.R60L,P2RY6:NM_001277206:exon3:c.G179T:p.R60L,P2RY6:NM_001277207:exon3:c.G179T:p.R60L,P2RY6:NM_176797:exon3:c.G179T:p.R60L,P2RY6:NM_001277205:exon4:c.G179T:p.R60L,P2RY6:NM_176796:exon4:c.G179T:p.R60L,P2RY6:NM_176798:exon4:c.G179T:p.R60L		.	1.282	.		0.0002	9.08E-05	Fam88_f_m_aF	0/0;0/1;0/1
chr11	73296910	73296910	C	T	P2RY6	exonic	nonsynonymous SNV	P2RY6:NM_001277208:exon2:c.C695T:p.P232L,P2RY6:NM_001277204:exon3:c.C392T:p.P131L,P2RY6:NM_001277206:exon3:c.C392T:p.P131L,P2RY6:NM_001277207:exon3:c.C392T:p.P131L,P2RY6:NM_176797:exon3:c.C392T:p.P131L,P2RY6:NM_001277205:exon4:c.C392T:p.P131L,P2RY6:NM_176796:exon4:c.C392T:p.P131L,P2RY6:NM_176798:exon4:c.C392T:p.P131L		.	1.159	.		3.28E-05	1.40E-05	Fam119_f_m_aM_aM	0/1;0/0;0/1;0/1
chr11	73365499	73365499	A	G	ARHGEF17	exonic	nonsynonymous SNV	ARHGEF17:NM_014786:exon19:c.A5660G:p.H1887R		.	1.262	0.0003		0.0001	3.49E-05	Fam81_f_m_aM_uM	0/0;0/1;0/1;0/1
chr11	76140830	76140830	G	A	UVRAG	exonic	nonsynonymous SNV	UVRAG:NM_003369:exon15:c.G1517A:p.R506Q		.	1.015	.		0.0004	3.49E-05	Fam26_f_m_aM_uM	0/0;0/1;0/1;0/0
chr11	76187078	76187078	T	G	WNT11	exonic	nonsynonymous SNV	WNT11:NM_004626:exon5:c.A1052C:p.Y351S		.	1.237	.		3.27E-05	1.40E-05	Fam10_f_m_aM_uF	0/0;0/1;0/1;0/1
chr11	77676889	77676889	C	T	RSF1	exonic	nonsynonymous SNV	RSF1:NM_016578:exon13:c.G3244A:p.A1082T		.	1.614	7.34E-05		0.0002	4.19E-05	Fam80_f_m_aM_uM	0/0;0/1;0/1;0/1
chr11	78676202	78676202	G	A	TENM4	exonic	nonsynonymous SNV	TENM4:NM_001098816:exon30:c.C5446T:p.R1816C		.	1.117	.	.		.	Fam100_f_m_aF	0/0;0/1;0/1
chr11	82733674	82733674	C	G	FAM181B	exonic	nonsynonymous SNV	FAM181B:NM_175885:exon1:c.G56C:p.G19A		.	1.885	.		9.97E-05	6.99E-06	Fam82_f_m_aM_uF	0/0;0/1;0/1;0/0
chr11	83541708	83541708	C	A	DLG2	exonic	nonsynonymous SNV	DLG2:NM_001142702:exon3:c.G222T:p.E74D,DLG2:NM_001142700:exon12:c.G1467T:p.E489D,DLG2:NM_001206769:exon14:c.G1893T:p.E631D,DLG2:NM_001300983:exon15:c.G1776T:p.E592D,DLG2:NM_001351276:exon15:c.G1833T:p.E611D,DLG2:NM_001364:exon15:c.G1776T:p.E592D,DLG2:NM_001351275:exon18:c.G2124T:p.E708D,DLG2:NM_001351274:exon19:c.G2127T:p.E709D,DLG2:NM_001142699:exon20:c.G2091T:p.E697D		.	1.59	.		0.0005	1.40E-05	Fam116_f_m_aM_aF	0/0;0/1;0/0;0/1
chr11	86559712	86559712	G	A	ME3	exonic	nonsynonymous SNV	ME3:NM_001014811:exon2:c.C295T:p.R99W,ME3:NM_001161586:exon3:c.C295T:p.R99W,ME3:NM_001351934:exon3:c.C295T:p.R99W,ME3:NM_006680:exon3:c.C295T:p.R99W		.	1.142	.		7.82E-05	4.19E-05	Fam101_f_m_aM	0/1;0/0;0/1
chr11	87309530	87309530	G	A	TMEM135	exonic	nonsynonymous SNV	TMEM135:NM_001168724:exon9:c.G728A:p.G243E,TMEM135:NM_022918:exon10:c.G794A:p.G265E		.	1.34	.	.		.	Fam31_f_m_aM_uF	0/1;0/0;0/1;0/1
chr11	95026444	95026444	G	A	KDM4E	exonic	nonsynonymous SNV	KDM4E:NM_001161630:exon1:c.G887A:p.R296Q		.	1.215	.		0.0002	3.49E-05	Fam20_f_m_aM_uF	0/1;0/0;0/1;0/1
chr11	101489055	101489055	T	C	TRPC6	exonic	nonsynonymous SNV	TRPC6:NM_004621:exon4:c.A1175G:p.Y392C		.	1.136	.		3.27E-05	.	Fam56_f_m_aF_aM	0/0;0/1;0/0;0/1
chr11	105924660	105924660	G	A	GRIA4	exonic	nonsynonymous SNV	GRIA4:NM_000829:exon12:c.G1738A:p.D580N,GRIA4:NM_001077243:exon12:c.G1738A:p.D580N		.	2.02	0.0002		0.0002	7.68E-05	Fam87_f_m_aM_uM	0/0;0/1;0/1;0/1

chr11	108094413	108094413	A	T	CUL5	exonic	nonsynonymous SNV	CUL5:NM_003478:exon14:c.A1466T:p.Y489F	.	1.119	.		3.50E-05	2.09E-05	Fam70_f_m_aM	0/0;0/1;0/1
chr11	111840702	111840702	A	G	ALG9	exonic	nonsynonymous SNV	ALG9:NM_001352409:exon9:c.T613C:p.Y205H,ALG9:NM_001352418:exon9:c.T1003C:p.Y335H,ALG9:NM_001352423:exon9:c.T490C:p.Y164H,ALG9:NM_001077690:exon10:c.T1126C:p.Y376H,ALG9:NM_001077691:exon10:c.T613C:p.Y205H,ALG9:NM_001077692:exon10:c.T613C:p.Y205H,ALG9:NM_001352410:exon10:c.T613C:p.Y205H,ALG9:NM_001352411:exon10:c.T613C:p.Y205H,ALG9:NM_001352412:exon10:c.T613C:p.Y205H,ALG9:NM_001352413:exon10:c.T613C:p.Y205H,ALG9:NM_001352414:exon10:c.T613C:p.Y205H,ALG9:NM_001352415:exon10:c.T613C:p.Y205H,ALG9:NM_001352416:exon10:c.T613C:p.Y205H,ALG9:NM_001352417:exon10:c.T1126C:p.Y376H,ALG9:NM_001352419:exon10:c.T613C:p.Y205H,ALG9:NM_001352420:exon10:c.T613C:p.Y205H,ALG9:NM_001352421:exon10:c.T613C:p.Y205H,ALG9:NM_024740:exon10:c.T1126C:p.Y376H,ALG9:NM_001352422:exon11:c.T538C:p.Y180H	.	1.176	.	.	.		Fam121_f_m_aF_aM	0/1;0/0;0/0;0/1
chr11	111911661	111911661	G	A	CRYAB	exonic	nonsynonymous SNV	CRYAB:NM_001289808:exon1:c.C64T:p.R22C,CRYAB:NM_001289807:exon2:c.C64T:p.R22C,CRYAB:NM_001368245:exon2:c.C64T:p.R22C,CRYAB:NM_001885:exon2:c.C64T:p.R22C	.	1.079	.		6.88E-05	6.98E-06	Fam114_f_m_aM	0/1;0/0;0/1
chr11	116790601	116790601	G	A	APOA5	exonic	nonsynonymous SNV	APOA5:NM_001166598:exon4:c.C628T:p.H210Y,APOA5:NM_052968:exon4:c.C628T:p.H210Y	.	1.486	.	.	.		Fam118_f_m_aM_aF_uM	0/0;0/1;0/1;0/0;0/0
chr11	117189361	117189361	A	T	SIDT2	exonic	nonsynonymous SNV	SIDT2:NM_001040455:exon15:c.A1379T:p.Y460F	.	1.411	.	.	.		Fam45_f_m_aM_uF	0/0;0/1;0/1;0/0
chr11	117518612	117518612	C	T	DSCAML1	exonic	nonsynonymous SNV	DSCAML1:NM_001367904:exon7:c.G1364A:p.R455H,DSCAML1:NM_001367905:exon7:c.G956A:p.R319H,DSCAML1:NM_020693:exon7:c.G1364A:p.R455H	.	1.123		0.0003	0.0005	0.0002	Fam45_f_m_aM_uF	0/1;0/0;0/1;0/0
chr11	117776868	117776868	T	C	DSCAML1	exonic	nonsynonymous SNV	DSCAML1:NM_001367904:exon3:c.A434G:p.K145R,DSCAML1:NM_001367905:exon3:c.A26G:p.K9R,DSCAML1:NM_020693:exon3:c.A434G:p.K145R	.	1.367	.	.	.		Fam49_f_m_aM	0/0;0/1;0/1
chr11	118373576	118373576	T	C	UBE4A	exonic	nonsynonymous SNV	UBE4A:NM_001204077:exon8:c.T1007C:p.I336T,UBE4A:NM_004788:exon8:c.T1028C:p.I343T	.	1.068	.		0.0006	0.0001	Fam40_f_m_aM_aM	0/0;0/1;0/0;0/1
chr11	118472150	118472150	C	G	KMT2A	exonic	nonsynonymous SNV	KMT2A:NM_001197104:exon3:c.C991G:p.R331G,KMT2A:NM_005933:exon3:c.C991G:p.R331G	.	2.108		0.0003	6.51E-05	2.10E-05	Fam46_f_m_aM_uM	0/1;0/0;0/1;0/1
chr11	119017482	119017482	A	G	RPS25	exonic	nonsynonymous SNV	RPS25:NM_001028:exon3:c.T163C:p.Y55H	.	1.598	.	.	.		Fam94_f_m_aM	0/1;0/0;0/1
chr11	119113637	119113637	G	T	C2CD2L	exonic	nonsynonymous SNV	C2CD2L:NM_001290474:exon11:c.G1414T:p.V472L,C2CD2L:NM_014807:exon11:c.G1414T:p.V472L	.	1.371	.	.	.		Fam110_f_m_aM_aM_uMs	0/1;0/0;0/0;0/1;0/1
chr11	119336177	119336177	G	A	RNF26	exonic	nonsynonymous SNV	RNF26:NM_032015:exon1:c.G1055A:p.G352D	.	1.234	.		2.24E-05	6.98E-06	Fam66_f_m_aM	0/0;0/1;0/1
chr11	121045706	121045706	G	A	TBCEL	exonic	nonsynonymous SNV	TBCEL:NM_001130047:exon2:c.G16A:p.G6R,TBCEL:NM_152715:exon2:c.G16A:p.G6R,TBCEL:NM_001363644:exon3:c.G16A:p.G6R	.	1.946	.	.	.		Fam116_f_m_aM_aF	0/0;0/1;0/0;0/1
chr11	121145969	121145969	C	T	TECTA	exonic	nonsynonymous SNV	TECTA:NM_005422:exon11:c.C3958T:p.P1320S	.	1.074		7.35E-05	2.23E-05	6.98E-06	Fam35_f_m_aF_uM	0/1;0/0;0/1;0/1
chr11	121168904	121168904	G	A	TECTA	exonic	nonsynonymous SNV	TECTA:NM_005422:exon19:c.G5978A:p.R1993Q	.	1.146	.		0.0002	2.10E-05	Fam17_f_m_aM_uM	0/0;0/1;0/1;0/0
chr11	121605150	121605150	G	C	SORL1	exonic	nonsynonymous SNV	SORL1:NM_003105:exon34:c.G4689C:p.W1563C	.	1.151		0.0003	0.0007	0.0003	Fam56_f_m_aF_aM	0/1;0/0;0/1;0/1
chr11	123618706	123618706	G	T	GRAMD1B	exonic	nonsynonymous SNV	GRAMD1B:NM_001286564:exon18:c.G1795T:p.V599F,GRAMD1B:NM_001330396:exon18:c.G1783T:p.V595F,GRAMD1B:NM_001367418:exon18:c.G1984T:p.V662F,GRAMD1B:NM_020716:exon18:c.G1915T:p.V639F,GRAMD1B:NM_001286563:exon19:c.G1936T:p.V646F,GRAMD1B:NM_001367419:exon19:c.G1996T:p.V666F,GRAMD1B:NM_001367420:exon19:c.G1996T:p.V666F,GRAMD1B:NM_001367421:exon19:c.G2200T:p.V734F	.	1.346	.		0.0004	4.89E-05	Fam30_f_m_aM_uM	0/0;0/1;0/1;0/0
chr11	124774775	124774775	T	A	MSANTD2	exonic	nonsynonymous SNV	MSANTD2:NM_001312920:exon1:c.A20T:p.E7V,MSANTD2:NM_001308027:exon2:c.A710T:p.E237V,MSANTD2:NM_001312921:exon2:c.A20T:p.E7V,MSANTD2:NM_024631:exon2:c.A554T:p.E185V,MSANTD2:NM_001352400:exon3:c.A20T:p.E7V,MSANTD2:NM_001352401:exon3:c.A20T:p.E7V	.	1.82	.	.	.		Fam95_f_m_aM_aM_uF	0/1;0/0;0/1;0/1;0/0
chr11	124921191	124921191	C	T	HEPACAM	exonic	nonsynonymous SNV	HEPACAM:NM_152722:exon7:c.G1198A:p.V400M	.	2.801		0.0004	0.0005	0.0002	Fam70_f_m_aM	0/0;0/1;0/1
chr11	125397932	125397932	A	C	PKNOX2	exonic	nonsynonymous SNV	PKNOX2:NM_022062:exon7:c.A458C:p.E153A	.	1.037	.	.	.		Fam110_f_m_aM_aM_uMs	0/1;0/0;0/1;0/0;0/0
chr11	125575315	125575315	G	A	EI24	exonic	nonsynonymous SNV	EI24:NM_001290135:exon3:c.G53A:p.R18Q,EI24:NM_004879:exon3:c.G95A:p.R32Q,EI24:NM_001330419:exon4:c.G95A:p.R32Q	.	1.017		7.34E-05	0.0003	6.98E-06	Fam74_f_m_aM_uM	0/1;0/0;0/1;0/0
chr11	125606415	125606415	T	C	STT3A	exonic	nonsynonymous SNV	STT3A:NM_001278504:exon7:c.T454C:p.Y152H,STT3A:NM_152713:exon8:c.T730C:p.Y244H,STT3A:NM_001278503:exon9:c.T730C:p.Y244H	.	2.102	.	.	.		Fam76_f_m_aM_uM	0/0;0/1;0/1;0/0
chr11	126414021	126414021	G	C	ST3GAL4	exonic	nonsynonymous SNV	ST3GAL4:NM_001254759:exon9:c.G973C:p.A325P,ST3GAL4:NM_001254757:exon11:c.G976C:p.A326P,ST3GAL4:NM_001254758:exon11:c.G976C:p.A326P,ST3GAL4:NM_001348398:exon11:c.G882C:p.E294D,ST3GAL4:NM_001348399:exon11:c.G976C:p.A326P,ST3GAL4:NM_001348400:exon11:c.G964C:p.A322P,ST3GAL4:NM_006278:exon11:c.G964C:p.A322P,ST3GAL4:NM_001348396:exon12:c.G1039C:p.A347P,ST3GAL4:NM_001348397:exon12:c.G1039C:p.A347P	.	1.386	.	.	.		Fam74_f_m_aM_uM	0/0;0/1;0/1;0/1
chr11	128480256	128480256	C	T	ETS1	exonic	nonsynonymous SNV	ETS1:NM_001162422:exon2:c.G278A:p.R93Q,ETS1:NM_005238:exon6:c.G926A:p.R309Q,ETS1:NM_001143820:exon8:c.G1058A:p.R353Q	.	1.228	.	.		1.40E-05	Fam43_f_m_aM	0/0;0/1;0/1

chr11	128486122	128486122	T	A	ETS1	exonic	nonsynonymous SNV	ETS1:NM_001330451:exon4:c.A428T:p.N143I,ETS1:NM_005238:exon4:c.A428T:p.N143I,ETS1:NM_001143820:exon6:c.A560T:p.N187I	.	1.073	.	.	.	Fam77_f_m_aM_aF_uM_uFs	0/0;0/1;0/0;0/1;0/1;0/0
chr11	128758299	128758299	G	T	FLI1	exonic	nonsynonymous SNV	FLI1:NM_002017:exon2:c.G203T:p.R68L,FLI1:NM_001167681:exon3:c.G104T:p.R35L	.	1.02	0.0005	0.0005	0.0003	Fam5_f_m_aM	0/1;0/0;0/1
chr11	129914738	129914738	T	C	PRDM10	exonic	nonsynonymous SNV	PRDM10:NM_001367898:exon13:c.A2549G:p.Q850R,PRDM10:NM_199439:exon13:c.A2549G:p.Q850R,PRDM10:NM_001367896:exon14:c.A1958G:p.Q653R,PRDM10:NM_001367899:exon14:c.A2561G:p.Q854R,PRDM10:NM_199438:exon14:c.A2561G:p.Q854R,PRDM10:NM_001367895:exon15:c.A1958G:p.Q653R,PRDM10:NM_001367897:exon15:c.A1970G:p.Q657R,PRDM10:NM_001367890:exon16:c.A2459G:p.Q820R,PRDM10:NM_001367891:exon16:c.A2747G:p.Q916R,PRDM10:NM_001367894:exon16:c.A2459G:p.Q820R,PRDM10:NM_199437:exon17:c.A2807G:p.Q936R,PRDM10:NM_001367892:exon18:c.A2753G:p.Q918R,PRDM10:NM_001367893:exon18:c.A2819G:p.Q940R,PRDM10:NM_020228:exon18:c.A2819G:p.Q940R	.	1.283	.	.	6.98E-06	Fam16_f_m_aM_aM	0/1;0/0;0/1;0/1
chr11	133921108	133921108	G	A	IGSF9B	exonic	nonsynonymous SNV	IGSF9B:NM_001277285:exon18:c.C2617T:p.R873C	.	2.045	0.0001	0.0002	0.0001	Fam52_f_m_aM	0/1;0/0;0/1
chr11	134383897	134383897	C	T	B3GAT1	exonic	nonsynonymous SNV	B3GAT1:NM_054025:exon3:c.G404A:p.R135H,B3GAT1:NM_001367973:exon4:c.G443A:p.R148H,B3GAT1:NM_018644:exon4:c.G404A:p.R135H	.	1.063	.	.	2.09E-05	Fam95_f_m_aM_aM_uF	0/0;0/1;0/1;0/1;0/1
chr12	157548	157548	A	G	IQSEC3	exonic	nonsynonymous SNV	IQSEC3:NM_015232:exon6:c.A1388G:p.N463S,IQSEC3:NM_001170738:exon7:c.A2297G:p.N766S	.	1.048	.	7.88E-05	6.98E-06	Fam10_f_m_aM_uF	0/0;0/1;0/1;0/1
chr12	1632849	1632849	G	A	WNT5B	exonic	nonsynonymous SNV	WNT5B:NM_030775:exon3:c.G272A:p.R91Q,WNT5B:NM_032642:exon3:c.G272A:p.R91Q	.	1.372	.	.	1.40E-05	Fam73_f_m_aF_dM	0/1;0/0;0/1;0/0
chr12	1646037	1646037	C	A	WNT5B	exonic	nonsynonymous SNV	WNT5B:NM_030775:exon5:c.C865A:p.L289M,WNT5B:NM_032642:exon5:c.C865A:p.L289M	.	1.13	.	4.50E-05	.	Fam47_f_m_aM	0/1;0/0;0/1
chr12	2493262	2493262	C	T	CACNA1C	exonic	nonsynonymous SNV	CACNA1C:NM_000719:exon7:c.C989T:p.T330M,CACNA1C:NM_001129827:exon7:c.C989T:p.T330M,CACNA1C:NM_001129829:exon7:c.C989T:p.T330M,CACNA1C:NM_001129830:exon7:c.C989T:p.T330M,CACNA1C:NM_001129831:exon7:c.C989T:p.T330M,CACNA1C:NM_001129832:exon7:c.C989T:p.T330M,CACNA1C:NM_001129833:exon7:c.C989T:p.T330M,CACNA1C:NM_001129834:exon7:c.C989T:p.T330M,CACNA1C:NM_001129835:exon7:c.C989T:p.T330M,CACNA1C:NM_001129836:exon7:c.C989T:p.T330M,CACNA1C:NM_001129837:exon7:c.C989T:p.T330M,CACNA1C:NM_001129838:exon7:c.C989T:p.T330M,CACNA1C:NM_001129839:exon7:c.C989T:p.T330M,CACNA1C:NM_001129840:exon7:c.C989T:p.T330M,CACNA1C:NM_001129841:exon7:c.C989T:p.T330M,CACNA1C:NM_001129842:exon7:c.C989T:p.T330M,CACNA1C:NM_001129843:exon7:c.C989T:p.T330M,CACNA1C:NM_001129844:exon7:c.C980T:p.T327M,CACNA1C:NM_001129846:exon7:c.C989T:p.T330M,CACNA1C:NM_001167623:exon7:c.C989T:p.T330M,CACNA1C:NM_001167624:exon7:c.C989T:p.T330M,CACNA1C:NM_001167625:exon7:c.C989T:p.T330M,CACNA1C:NM_199460:exon7:c.C989T:p.T330M	.	2.088	.	3.27E-05	2.09E-05	Fam25_f_m_aM	0/0;0/1;0/1
chr12	5045554	5045554	C	A	KCNA5	exonic	nonsynonymous SNV	KCNA5:NM_002234:exon1:c.C1407A:p.D469E	.	1.234	.	1.12E-05	.	Fam67_f_m_aM_uF	0/1;0/0;0/1;0/1
chr12	6375573	6375573	C	-	LTBR	exonic	frameshift deletion	LTBR:NM_001270987:exon1:c.18delC:p.S7Hfs*2	0.502	.	.	.	.	Fam1_f_m_aM	0/0;0/1;0/1
chr12	6638287	6638287	C	T	ACRBP	exonic	nonsynonymous SNV	ACRBP:NM_032489:exon10:c.G1627A:p.G543R	.	1.127	0.0003	4.48E-05	2.79E-05	Fam45_f_m_aM_uF	0/1;0/0;0/1;0/0
chr12	6748976	6748976	G	A	MLF2	exonic	nonsynonymous SNV	MLF2:NM_005439:exon8:c.C566T:p.A189V	.	2.346	0.0006	2.60E-05	6.98E-06	Fam117_f_m_aM_aF	0/1;0/0;0/1;0/0
chr12	6937516	6937516	C	T	ATN1	exonic	nonsynonymous SNV	ATN1:NM_001007026:exon5:c.C2249T:p.P750L,ATN1:NM_001940:exon5:c.C2249T:p.P750L	.	1.887	.	.	.	Fam22_f_m_aF_aF_uF	0/0;0/1;0/1;0/1;0/0
chr12	6937955	6937955	A	G	ATN1	exonic	nonsynonymous SNV	ATN1:NM_001007026:exon6:c.A2405G:p.K802R,ATN1:NM_001940:exon6:c.A2405G:p.K802R	.	1.077	.	1.64E-05	.	Fam13_f_m_aM	0/0;0/1;0/1
chr12	6955457	6955457	C	T	PTPN6	exonic	nonsynonymous SNV	PTPN6:NM_002831:exon6:c.C719T:p.A240V,PTPN6:NM_080548:exon6:c.C725T:p.A242V,PTPN6:NM_080549:exon6:c.C719T:p.A240V	.	1.211	.	2.24E-05	.	Fam14_f_m_aM_aM	0/1;0/0;0/1;0/1
chr12	8095673	8095673	A	T	NECAP1	exonic	nonsynonymous SNV	NECAP1:NM_015509:exon7:c.A749T:p.D250V	.	1.625	.	.	6.98E-06	Fam12_f_m_aM_uM_aM	0/1;0/0;0/1;0/0;0/1
chr12	8921649	8921649	A	G	PHC1	exonic	nonsynonymous SNV	PHC1:NM_004426:exon5:c.A355G:p.S119G	.	1.568	.	.	.	Fam21_f_m_aM_uM	0/0;0/1;0/1;0/1
chr12	13611737	13611737	C	T	GRIN2B	exonic	nonsynonymous SNV	GRIN2B:NM_000834:exon8:c.G1768A:p.A590T	.	1.596	0.0002	0.0003	6.28E-05	Fam64_f_m_aM	0/0;0/1;0/1
chr12	13866019	13866019	C	T	GRIN2B	exonic	nonsynonymous SNV	GRIN2B:NM_000834:exon2:c.G190A:p.V64M	.	1.2	0.0004	7.82E-05	0.0001	Fam74_f_m_aM_uM	0/1;0/0;0/1;0/1
chr12	19253987	19253987	C	T	PLEKHA5	exonic	nonsynonymous SNV	PLEKHA5:NM_001143821:exon4:c.C275T:p.P92L,PLEKHA5:NM_001256470:exon4:c.C275T:p.P92L,PLEKHA5:NM_019012:exon4:c.C275T:p.P92L	.	1.847	.	3.38E-05	6.98E-06	Fam32_f_m_aM_uM	0/1;0/0;0/1;0/1
chr12	20680052	20680052	A	C	PDE3A	exonic	nonsynonymous SNV	PDE3A:NM_001244683:exon15:c.A2241C:p.R747S,PDE3A:NM_000921:exon16:c.A3207C:p.R1069S	.	1.047	.	.	.	Fam52_f_m_aM	0/0;0/1;0/1
chr12	21773354	21773354	G	C	KCNJ8	exonic	nonsynonymous SNV	KCNJ8:NM_004982:exon2:c.C263G:p.A88G	.	2.229	0.0004	0.0005	0.0002	Fam83_f_m_aF	0/0;0/1;0/1
chr12	26711246	26711246	C	T	ITPR2	exonic	nonsynonymous SNV	ITPR2:NM_002223:exon9:c.G878A:p.R293H	.	2.187	.	6.47E-05	6.98E-06	Fam46_f_m_aM_uM	0/0;0/1;0/1;0/0
chr12	30639602	30639602	A	G	IPO8	exonic	nonsynonymous SNV	IPO8:NM_001190995:exon17:c.T1787C:p.L596S,IPO8:NM_006390:exon21:c.T2402C:p.L801S	.	1.059	.	.	.	Fam55_f_m_aM_aM_dM	0/0;0/1;0/1;0/0;0/0
chr12	30669222	30669222	A	G	IPO8	exonic	nonsynonymous SNV	IPO8:NM_001190995:exon6:c.T490C:p.W164R,IPO8:NM_006390:exon10:c.T1105C:p.W369R	.	1.264	.	0.0002	.	Fam39_f_m_aM	0/0;0/1;0/1

chr12	31387713	31387713	C	A	DENND5B	exonic	nonsynonymous SNV	DENND5B:NM_144973:exon21:c.G3715T:p.A1239S,DENND5B:NM_001308339:exon23:c.G3820T:p.A1274S	.	1.192	0.0003	7.70E-05	3.49E-05	Fam115_f_m_aF_aM_aF	0/0;0/1;0/0;0/1;0/0
chr12	32216346	32216346	T	C	BICD1	exonic	nonsynonymous SNV	BICD1:NM_001003398:exon2:c.T313C:p.Y105H,BICD1:NM_001354186:exon2:c.T313C:p.Y105H,BICD1:NM_001354187:exon2:c.T313C:p.Y105H,BICD1:NM_001354188:exon2:c.T313C:p.Y105H,BICD1:NM_001354189:exon2:c.T313C:p.Y105H,BICD1:NM_001363603:exon2:c.T313C:p.Y105H,BICD1:NM_001714:exon2:c.T313C:p.Y105H	.	1.454	.	5.58E-05	6.98E-06	Fam99_f_m_aM_aM	0/1;0/0;0/1;0/1
chr12	32327563	32327563	C	T	BICD1	exonic	nonsynonymous SNV	BICD1:NM_001003398:exon5:c.C1108T:p.R370W,BICD1:NM_001354186:exon5:c.C1108T:p.R370W,BICD1:NM_001354187:exon5:c.C1108T:p.R370W,BICD1:NM_001354188:exon5:c.C1108T:p.R370W,BICD1:NM_001354189:exon5:c.C1108T:p.R370W,BICD1:NM_001363603:exon5:c.C1108T:p.R370W,BICD1:NM_001714:exon5:c.C1108T:p.R370W	.	1.182	.	0.0003	6.98E-06	Fam116_f_m_aM_aF	0/1;0/0;0/1;0/0
chr12	39604902	39604902	C	T	ABCD2	exonic	nonsynonymous SNV	ABCD2:NM_005164:exon4:c.G1265A:p.R422Q	.	1.541	.	.	.	Fam33_f_m_aM_uM	0/1;0/0;0/1;0/1
chr12	39618808	39618808	C	T	ABCD2	exonic	nonsynonymous SNV	ABCD2:NM_005164:exon1:c.G808A:p.V270M	.	1.225	.	.	.	Fam110_f_m_aM_aM_uMs	0/1;0/0;0/1;0/0;0/1
chr12	41189035	41189035	G	A	PDZRN4	exonic	nonsynonymous SNV	PDZRN4:NM_001164595:exon1:c.G580A:p.E194K	.	1.001	7.40E-05	.	1.40E-05	Fam42_f_m_aM_uF	0/1;0/0;0/1;0/0
chr12	43799442	43799442	G	C	TWF1	exonic	nonsynonymous SNV	TWF1:NM_001242397:exon5:c.C439G:p.L147V,TWF1:NM_002822:exon5:c.C439G:p.L147V	.	1.107	0.0001	4.51E-05	6.99E-06	Fam26_f_m_aM_uM	0/1;0/0;0/1;0/1
chr12	45730067	45730067	C	A	ARID2	exonic	nonsynonymous SNV	ARID2:NM_001347839:exon2:c.C116A:p.A39E,ARID2:NM_152641:exon2:c.C116A:p.A39E	.	1.847	.	6.20E-05	3.49E-05	Fam53_f_m_aM	0/0;0/1;0/1
chr12	45836783	45836783	C	A	ARID2	exonic	nonsynonymous SNV	ARID2:NM_001347839:exon8:c.C815A:p.P272Q,ARID2:NM_152641:exon8:c.C815A:p.P272Q	.	2.19	.	.	.	Fam46_f_m_aM_uM	0/0;0/1;0/1;0/0
chr12	48071723	48071723	T	C	SENP1	splicing	.	.	0.427	.	.	2.29E-05	6.98E-06	Fam107_f_m_aM	0/1;0/0;0/1
chr12	48131341	48131341	G	A	PFKM	exonic	nonsynonymous SNV	PFKM:NM_001166688:exon3:c.G185A:p.G62E,PFKM:NM_001354747:exon3:c.G35A:p.G12E,PFKM:NM_001354748:exon3:c.G35A:p.G12E,PFKM:NM_000289:exon4:c.G185A:p.G62E,PFKM:NM_001166687:exon4:c.G185A:p.G62E,PFKM:NM_001354740:exon4:c.G329A:p.G110E,PFKM:NM_001354742:exon4:c.G185A:p.G62E,PFKM:NM_001354743:exon4:c.G185A:p.G62E,PFKM:NM_001354744:exon4:c.G185A:p.G62E,PFKM:NM_001354746:exon4:c.G185A:p.G62E,PFKM:NM_001363619:exon4:c.G185A:p.G62E,PFKM:NM_001354741:exon5:c.G209A:p.G70E,PFKM:NM_001354745:exon5:c.G98A:p.G33E,PFKM:NM_001166686:exon6:c.G398A:p.G133E,PFKM:NM_001354737:exon6:c.G398A:p.G133E,PFKM:NM_001354738:exon6:c.G398A:p.G133E,PFKM:NM_001354739:exon6:c.G398A:p.G133E,PFKM:NM_001354735:exon7:c.G494A:p.G165E,PFKM:NM_001354736:exon7:c.G494A:p.G165E	.	1.605	.	1.12E-05	6.98E-06	Fam117_f_m_aM_aF	0/0;0/1;0/1;0/1
chr12	48716570	48716570	A	G	CCNT1	exonic	nonsynonymous SNV	CCNT1:NM_001240:exon1:c.T106C:p.S36P,CCNT1:NM_001277842:exon1:c.T106C:p.S36P	.	1.449	0.0003	0.0002	7.67E-05	Fam26_f_m_aM_uM	0/0;0/1;0/1;0/1
chr12	48771724	48771724	C	T	ADCY6	exonic	nonsynonymous SNV	ADCY6:NM_015270:exon19:c.G3037A:p.A1013T	.	1.152	0.0006	9.83E-05	6.98E-06	Fam24_f_m_aM_aM	0/1;0/0;0/1;0/0
chr12	48783221	48783221	G	A	ADCY6	exonic	nonsynonymous SNV	ADCY6:NM_015270:exon2:c.C214T:p.R72W	.	1.05	0.0003	6.56E-05	2.09E-05	Fam59_f_m_aF_uM	0/0;0/1;0/1;0/1
chr12	49024934	49024934	C	T	KMT2D	exonic	nonsynonymous SNV	KMT2D:NM_003482:exon49:c.G15797A:p.R5266H	.	1.186	0.0007	0.0008	0.0005	Fam10_f_m_aM_uF	0/0;0/1;0/1;0/1
chr12	49048748	49048748	G	A	KMT2D	exonic	nonsynonymous SNV	KMT2D:NM_003482:exon13:c.C4042T:p.P1348S	.	2.458	.	.	.	Fam84_f_m_aF	0/1;0/0;0/1
chr12	49094349	49094349	C	G	DHH	exonic	nonsynonymous SNV	DHH:NM_021044:exon1:c.G164C:p.R55P	.	2.081	.	.	.	Fam18_f_m_aM_uF	0/1;0/0;0/1;0/0
chr12	49094433	49094433	C	T	DHH	exonic	nonsynonymous SNV	DHH:NM_021044:exon1:c.G80A:p.R27Q	.	1.845	.	0.0006	4.19E-05	Fam65_f_m_aM_uF_dF	0/1;0/0;0/1;0/1;0/0
chr12	49103801	49103801	G	A	LMBR1L	exonic	nonsynonymous SNV	LMBR1L:NM_001352162:exon4:c.C274T:p.R92W,LMBR1L:NM_001352163:exon5:c.C67T:p.R23W,LMBR1L:NM_001352164:exon5:c.C67T:p.R23W,LMBR1L:NM_001352166:exon5:c.C67T:p.R23W,LMBR1L:NM_001300751:exon6:c.C448T:p.R150W,LMBR1L:NM_001352161:exon6:c.C448T:p.R150W,LMBR1L:NM_001352165:exon6:c.C67T:p.R23W,LMBR1L:NM_018113:exon6:c.C448T:p.R150W,LMBR1L:NM_001300750:exon7:c.C433T:p.R145W	.	1.021	.	7.48E-05	.	Fam39_f_m_aM	0/1;0/0;0/1
chr12	49296985	49296985	G	A	PRPH	exonic	nonsynonymous SNV	PRPH:NM_006262:exon4:c.G799A:p.D267N	.	1.487	0.0001	5.62E-05	4.19E-05	Fam61_f_m_aM	0/0;0/1;0/1
chr12	49543449	49543449	C	T	KCNH3	exonic	nonsynonymous SNV	KCNH3:NM_001314030:exon5:c.C574T:p.R192W,KCNH3:NM_012284:exon5:c.C754T:p.R252W	.	1.978	.	7.54E-05	2.09E-05	Fam112_f_m_aM	0/0;0/1;0/1
chr12	49550174	49550174	G	A	KCNH3	exonic	nonsynonymous SNV	KCNH3:NM_001314030:exon10:c.G1583A:p.R528H,KCNH3:NM_012284:exon10:c.G1763A:p.R588H	.	2.517	.	9.83E-05	2.09E-05	Fam108_f_m_aM	0/0;0/1;0/1
chr12	50737085	50737085	C	T	DIP2B	exonic	nonsynonymous SNV	DIP2B:NM_173602:exon35:c.C4151T:p.P1384L	.	1.576	.	9.81E-05	6.98E-06	Fam121_f_m_aF_aM	0/1;0/0;0/0;0/1
chr12	51109158	51109158	T	G	TFCP2	exonic	nonsynonymous SNV	TFCP2:NM_001173452:exon6:c.A680C:p.H227P,TFCP2:NM_005653:exon6:c.A680C:p.H227P	.	2.003	.	.	.	Fam88_f_m_aF	0/0;0/1;0/1
chr12	51475184	51475184	C	T	SLC4A8	exonic	nonsynonymous SNV	SLC4A8:NM_001039960:exon16:c.C2150T:p.T717M,SLC4A8:NM_001258401:exon16:c.C1991T:p.T664M,SLC4A8:NM_001258403:exon16:c.C1991T:p.T664M	.	1.724	.	.	1.40E-05	Fam122_f_m_aM	0/0;0/1;0/1
chr12	51920786	51920786	C	T	ACVRL1	exonic	nonsynonymous SNV	ACVRL1:NM_001077401:exon9:c.C1405T:p.R469W,ACVRL1:NM_000020:exon10:c.C1405T:p.R469W	.	1.359	7.35E-05	7.46E-05	2.09E-05	Fam81_f_m_aM_uM	0/1;0/0;0/1;0/0
chr12	52054515	52054515	G	C	NR4A1	exonic	nonsynonymous SNV	NR4A1:NM_173157:exon2:c.G187C:p.E63Q,NR4A1:NM_001202233:exon3:c.G226C:p.E76Q,NR4A1:NM_001202234:exon3:c.G349C:p.E117Q,NR4A1:NM_002135:exon3:c.G187C:p.E63Q	.	1.132	.	3.27E-05	.	Fam70_f_m_aM	0/0;0/1;0/1
chr12	52365134	52365134	T	A	KRT85	exonic	nonsynonymous SNV	KRT85:NM_002283:exon2:c.A457T:p.T153S	.	1.028	.	.	.	Fam15_f_m_aM_aM	0/1;0/0;0/0;0/1

chr12	52900649	52900649	A	T	KRT8	exonic	nonsynonymous SNV	KRT8:NM_002273:exon4:c.T629A:p.L210Q,KRT8:NM_001256282:exon5:c.T713A:p.L238Q,KRT8:NM_001256293:exon5:c.T629A:p.L210Q	.	1.426	.	3.27E-05	.	Fam15_f_m_aM_aM	0/0;0/1;0/0;0/1
chr12	53016553	53016553	A	G	EIF4B	exonic	nonsynonymous SNV	EIF4B:NM_001300821:exon2:c.A94G:p.T32A,EIF4B:NM_001330654:exon2:c.A94G:p.T32A,EIF4B:NM_001417:exon2:c.A94G:p.T32A	.	1.084	0.0002	0.0004	0.0004	Fam91_m_aM_dM_aM_dM	0/0;0/1;0/0;0/0;0/0
chr12	53517183	53517183	T	G	ATF7	exonic	nonsynonymous SNV	ATF7:NM_001366561:exon11:c.A1241C:p.Q414P,ATF7:NM_001130060:exon12:c.A1343C:p.Q448P,ATF7:NM_001366555:exon12:c.A1439C:p.Q480P,ATF7:NM_001366556:exon12:c.A1406C:p.Q469P,ATF7:NM_001366558:exon12:c.A1406C:p.Q469P,ATF7:NM_006856:exon12:c.A1406C:p.Q469P	.	1.309	.	9.81E-05	2.09E-05	Fam77_f_m_aM_aF_uM_uFs	0/1;0/0;0/1;0/1;0/1;0/0
chr12	54411882	54411882	A	G	ITGA5	exonic	nonsynonymous SNV	ITGA5:NM_002205:exon2:c.T301C:p.W101R	.	1.407	7.36E-05	7.11E-05	2.80E-05	Fam12_f_m_aM_uM_aM	0/1;0/0;0/0;0/0;0/1
chr12	54419037	54419037	C	-	ITGA5	exonic	frameshift deletion	ITGA5:NM_002205:exon1:c.162delG:p.P56Rfs*114	0.455	.	.	.	.	Fam53_f_m_aM	0/1;0/0;0/1
chr12	54575631	54575631	A	G	PDE1B	exonic	nonsynonymous SNV	PDE1B:NM_001315535:exon9:c.A654G:p.I218M,PDE1B:NM_001165975:exon11:c.A1206G:p.I402M,PDE1B:NM_001288769:exon11:c.A1143G:p.I381M,PDE1B:NM_001315534:exon11:c.A855G:p.I285M,PDE1B:NM_000924:exon12:c.A1266G:p.I422M,PDE1B:NM_001288768:exon12:c.A855G:p.I285M	.	1.292	.	1.12E-05	1.40E-05	Fam32_f_m_aM_uM	0/0;0/1;0/1;0/1
chr12	54582761	54582761	T	G	PPP1R1A	exonic	nonsynonymous SNV	PPP1R1A:NM_006741:exon4:c.A218C:p.K73T	.	1.127	.	.	.	Fam91_m_aM_dM_aM_dM	0/0;0/0;0/1;0/1;0/1
chr12	56169868	56169868	T	C	SMARCC2	exonic	nonsynonymous SNV	SMARCC2:NM_003075:exon23:c.A2363G:p.E788G,SMARCC2:NM_001130420:exon24:c.A2456G:p.E819G,SMARCC2:NM_001330288:exon24:c.A2456G:p.E819G,SMARCC2:NM_139067:exon24:c.A2456G:p.E819G	.	1.506	0.0003	0.0005	0.0002	Fam90_f_m_aF_dM	0/0;0/1;0/1;0/1
chr12	56169904	56169904	C	T	SMARCC2	exonic	nonsynonymous SNV	SMARCC2:NM_003075:exon23:c.G2327A:p.R776Q,SMARCC2:NM_001130420:exon24:c.G2420A:p.R807Q,SMARCC2:NM_001330288:exon24:c.G2420A:p.R807Q,SMARCC2:NM_139067:exon24:c.G2420A:p.R807Q	.	1.293	0.0006	7.45E-05	3.49E-05	Fam95_f_m_aM_aM_uF	0/0;0/1;0/1;0/1;0/1
chr12	56214032	56214032	T	C	RNF41	exonic	nonsynonymous SNV	RNF41:NM_001242826:exon3:c.A16G:p.T6A,RNF41:NM_005785:exon3:c.A16G:p.T6A,RNF41:NM_194359:exon3:c.A16G:p.T6A	.	1.23	0.0004	0.0004	0.0002	Fam98_f_m_aM	0/1;0/0;0/1
chr12	56245548	56245548	C	T	ANKRD52	exonic	nonsynonymous SNV	ANKRD52:NM_173595:exon21:c.G2233A:p.A745T	.	1.863	.	1.14E-05	.	Fam114_f_m_aM	0/1;0/0;0/1
chr12	56315157	56315157	G	A	CNPY2	exonic	nonsynonymous SNV	CNPY2:NM_001190991:exon2:c.C61T:p.R21W,CNPY2:NM_014255:exon2:c.C61T:p.R21W	.	1.256	.	2.23E-05	6.99E-06	Fam56_f_m_aF_aM	0/0;0/1;0/1;0/1
chr12	57091718	57091718	C	G	NAB2	exonic	nonsynonymous SNV	NAB2:NM_001330305:exon2:c.C677G:p.A226G,NAB2:NM_005967:exon2:c.C677G:p.A226G	.	1.52	.	4.50E-05	6.98E-06	Fam44_f_m_aM_uF	0/0;0/1;0/1;0/1
chr12	57254975	57254975	C	A	R3HDM2	exonic	nonsynonymous SNV	R3HDM2:NM_001351217:exon22:c.G2675T:p.G892V,R3HDM2:NM_001351213:exon23:c.G2777T:p.G926V,R3HDM2:NM_001351214:exon23:c.G2777T:p.G926V,R3HDM2:NM_001351215:exon23:c.G2777T:p.G926V,R3HDM2:NM_001351216:exon23:c.G2765T:p.G922V,R3HDM2:NM_001351218:exon23:c.G2675T:p.G892V,R3HDM2:NM_001330122:exon24:c.G2831T:p.G944V,R3HDM2:NM_001330123:exon24:c.G2699T:p.G900V,R3HDM2:NM_001351208:exon24:c.G2873T:p.G958V,R3HDM2:NM_001351209:exon24:c.G2825T:p.G942V,R3HDM2:NM_001351212:exon24:c.G2777T:p.G926V,R3HDM2:NM_014925:exon24:c.G2729T:p.G910V,R3HDM2:NM_001330121:exon25:c.G2831T:p.G944V,R3HDM2:NM_001351205:exon25:c.G2927T:p.G976V,R3HDM2:NM_001351206:exon25:c.G2927T:p.G976V,R3HDM2:NM_001351211:exon25:c.G2795T:p.G932V,R3HDM2:NM_001351204:exon26:c.G2927T:p.G976V,R3HDM2:NM_001351207:exon27:c.G2897T:p.G966V	.	1.058	.	3.28E-05	.	Fam85_f_m_aM_aM	0/0;0/1;0/1;0/0
chr12	57575130	57575130	G	A	KIF5A	exonic	nonsynonymous SNV	KIF5A:NM_001354705:exon13:c.G1496A:p.R499Q,KIF5A:NM_004984:exon16:c.G1763A:p.R588Q	.	1.571	.	3.28E-05	.	Fam91_m_aM_dM_aM_dM	0/0;0/1;0/0;0/0;0/0
chr12	57730901	57730901	A	G	AGAP2	exonic	nonsynonymous SNV	AGAP2:NM_001122772:exon11:c.T2198C:p.V733A,AGAP2:NM_014770:exon11:c.T1190C:p.V397A	.	1.564	.	.	.	Fam116_f_m_aM_aF	0/1;0/0;0/0;0/1
chr12	57737484	57737484	C	A	AGAP2	exonic	nonsynonymous SNV	AGAP2:NM_001122772:exon1:c.G763T:p.G255W	.	1.542	7.35E-05	3.75E-05	3.49E-05	Fam71_f_m_aF	0/1;0/0;0/1
chr12	61754898	61754898	G	A	TAFA2	exonic	nonsynonymous SNV	TAFA2:NM_178539:exon3:c.C233T:p.T78M	.	1.592	.	0.0008	3.49E-05	Fam97_f_m_aM_aF	0/0;0/1;0/1;0/1
chr12	62302792	62302792	G	A	USP15	exonic	nonsynonymous SNV	USP15:NM_001252078:exon3:c.G220A:p.G74S,USP15:NM_001252079:exon3:c.G220A:p.G74S,USP15:NM_001351161:exon3:c.G220A:p.G74S,USP15:NM_006313:exon3:c.G220A:p.G74S	.	1.054	.	.	6.99E-06	Fam11_f_m_aM_uM	0/0;0/1;0/1;0/0
chr12	68856465	68856465	A	T	CPM	exonic	nonsynonymous SNV	CPM:NM_001005502:exon9:c.T1304A:p.V435E,CPM:NM_001874:exon9:c.T1304A:p.V435E,CPM:NM_198320:exon9:c.T1304A:p.V435E	.	1.133	.	.	6.98E-06	Fam115_f_m_aF_aM_aF	0/0;0/1;0/1;0/0;0/1
chr12	70521494	70521494	G	T	PTPRB	exonic	nonsynonymous SNV	PTPRB:NM_001206971:exon31:c.C5719A:p.H1907N,PTPRB:NM_001206972:exon31:c.C5719A:p.H1907N,PTPRB:NM_002837:exon32:c.C5989A:p.H1997N,PTPRB:NM_001330204:exon33:c.C6379A:p.H2127N,PTPRB:NM_001109754:exon34:c.C6643A:p.H2215N	.	1.308	.	0.0001	1.40E-05	Fam36_f_m_aM_uM	0/1;0/0;0/1;0/0
chr12	72031568	72031568	C	A	TPH2	exonic	nonsynonymous SNV	TPH2:NM_173353:exon11:c.C1346A:p.P449H	.	1.158	7.34E-05	1.12E-05	.	Fam79_f_m_aM_uM	0/0;0/1;0/1;0/0
chr12	72473116	72473116	A	T	TRHDE	exonic	nonsynonymous SNV	TRHDE:NM_013381:exon5:c.A1385T:p.K462M	.	1.197	.	.	.	Fam119_f_m_aM_aM	0/1;0/0;0/0;0/1
chr12	76031432	76031432	C	A	PHLDA1	exonic	nonsynonymous SNV	PHLDA1:NM_007350:exon1:c.G310T:p.G104C	.	1.693	.	4.42E-05	6.98E-06	Fam108_f_m_aM	0/1;0/0;0/1
chr12	76809823	76809823	C	T	ZDHHC17	exonic	nonsynonymous SNV	ZDHHC17:NM_001359626:exon5:c.C479T:p.T160I,ZDHHC17:NM_015336:exon5:c.C509T:p.T170I	.	1.26	7.35E-05	.	6.99E-06	Fam38_f_m_aM	0/0;0/1;0/1
chr12	79806164	79806164	A	G	PPP1R12A	splicing	.	.	0.158	.	0.0003	0.0002	0.0001	Fam48_f_m_aM_uM	0/1;0/0;0/1;0/1

chr12	80708033	80708033	G	C	MYF6	exonic	nonsynonymous SNV	MYF6:NM_002469:exon1:c.G314C:p.R105T	.	1.408	.	.	6.98E-06	Fam50_f_m_aF	0/0;0/1;0/1
chr12	94149660	94149660	G	A	PLXNC1	exonic	nonsynonymous SNV	PLXNC1:NM_005761:exon1:c.G689A:p.G230D	.	1.799	.	.	.	Fam87_f_m_aM_uM	0/1;0/0;0/1;0/1
chr12	94581940	94581940	G	T	TMCC3	exonic	nonsynonymous SNV	TMCC3:NM_001301036:exon2:c.C584A:p.S195Y,TMCC3:NM_020698:exon2:c.C677A:p.S226Y	.	1.04	.	.	.	Fam60_f_m_aF	0/0;0/1;0/1
chr12	94582210	94582210	T	C	TMCC3	exonic	nonsynonymous SNV	TMCC3:NM_001301036:exon2:c.A314G:p.Y105C,TMCC3:NM_020698:exon2:c.A407G:p.Y136C	.	1.081	.	5.58E-05	6.98E-06	Fam100_f_m_aF	0/0;0/1;0/1
chr12	96018846	96018846	A	T	LTA4H	exonic	nonsynonymous SNV	LTA4H:NM_000895:exon8:c.T769A:p.Y257N,LTA4H:NM_001256643:exon8:c.T697A:p.Y233N,LTA4H:NM_001256644:exon8:c.T697A:p.Y233N	.	1.199	.	.	6.98E-06	Fam17_f_m_aM_uM	0/1;0/0;0/1;0/1
chr12	98735585	98735586	GT	-	ANKS1B	exonic	stopgain	ANKS1B:NM_001352216:exon9:c.514_515del:p.T172*,ANKS1B:NM_001204081:exon10:c.739_740del:p.T247*,ANKS1B:NM_001352213:exon10:c.514_515del:p.T172*,ANKS1B:NM_001352214:exon10:c.514_515del:p.T172*,ANKS1B:NM_001352223:exon11:c.814_815del:p.T272*,ANKS1B:NM_020140:exon11:c.1219_1220del:p.T407*,ANKS1B:NM_001352197:exon12:c.1399_1400del:p.T467*,ANKS1B:NM_001352196:exon13:c.1471_1472del:p.T491*,ANKS1B:NM_152788:exon26:c.3721_3722del:p.T1241*	0.582	.	.	0.0001	2.09E-05	Fam62_f_m_aM_aM	0/0;0/1;0/0;0/1
chr12	101110433	101110433	G	C	ANO4	exonic	nonsynonymous SNV	ANO4:NM_001286616:exon22:c.G2179C:p.V727L,ANO4:NM_178826:exon22:c.G2074C:p.V692L,ANO4:NM_001286615:exon23:c.G2179C:p.V727L	.	1.457	.	.	.	Fam113_f_m_aF_raM_uF	0/0;0/0;0/0;0/1;0/0
chr12	102958692	102958692	G	T	ASCL1	exonic	nonsynonymous SNV	ASCL1:NM_004316:exon1:c.G448T:p.A150S	.	1.007	.	.	6.98E-06	Fam60_f_m_aF	0/1;0/0;0/1
chr12	103943796	103943796	G	C	HSP90B1	exonic	nonsynonymous SNV	HSP90B1:NM_003299:exon14:c.G1949C:p.S650T	.	1.162	.	.	.	Fam6_f_m_aM	0/1;0/0;0/1
chr12	103946665	103946665	C	T	HSP90B1	exonic	nonsynonymous SNV	HSP90B1:NM_003299:exon15:c.C2075T:p.P692L	.	1.293	.	0.0003	0.0002	Fam91_m_aM_dM_aM_dM	0/0;0/1;0/1;0/0;0/1
chr12	103980906	103980906	C	T	TDG	exonic	nonsynonymous SNV	TDG:NM_003211:exon4:c.C422T:p.P141L	.	1.508	.	.	6.98E-06	Fam106_f_m_aM	0/1;0/0;0/1
chr12	104757034	104757034	G	A	CHST11	exonic	nonsynonymous SNV	CHST11:NM_001173982:exon3:c.G275A:p.R92H,CHST11:NM_018413:exon3:c.G290A:p.R97H	.	1.847	0.0006	0.0002	6.98E-05	Fam28_f_m_aF_uF	0/0;0/1;0/1;0/1
chr12	104757400	104757400	G	A	CHST11	exonic	nonsynonymous SNV	CHST11:NM_001173982:exon3:c.G641A:p.R214H,CHST11:NM_018413:exon3:c.G656A:p.R219H	.	1.884	.	3.35E-05	.	Fam97_f_m_aM_aF	0/1;0/0;0/1;0/0
chr12	106066896	106066896	C	T	NUAK1	exonic	nonsynonymous SNV	NUAK1:NM_014840:exon7:c.G1892A:p.R631Q	.	1.411	0.0001	0.0001	0.0006	Fam86_f_m_aF	0/0;0/1;0/1
chr12	107657647	107657647	G	T	BTBD11	exonic	nonsynonymous SNV	BTBD11:NM_001017523:exon15:c.G1855T:p.D619Y,BTBD11:NM_001347943:exon15:c.G2887T:p.D963Y,BTBD11:NM_001347944:exon15:c.G1789T:p.D597Y,BTBD11:NM_001018072:exon17:c.G3244T:p.D1082Y	.	1.081	.	.	.	Fam21_f_m_aM_uM	0/1;0/0;0/1;0/1
chr12	108240370	108240370	C	T	WSCD2	exonic	nonsynonymous SNV	WSCD2:NM_014653:exon8:c.C1171T:p.R391C,WSCD2:NM_001304447:exon9:c.C1171T:p.R391C	.	1.253	7.34E-05	3.27E-05	6.98E-06	Fam89_f_m_aM	0/0;0/1;0/1
chr12	108538193	108538193	A	C	SART3	exonic	nonsynonymous SNV	SART3:NM_014706:exon8:c.T1073G:p.L358R	.	1.013	.	.	.	Fam82_f_m_aM_uF	0/0;0/1;0/1;0/1
chr12	109085066	109085066	G	A	USP30	exonic	nonsynonymous SNV	USP30:NM_032663:exon12:c.G1282A:p.D428N,USP30:NM_001301175:exon15:c.G1189A:p.D397N	.	1.068	.	3.45E-05	1.40E-05	Fam114_f_m_aM	0/1;0/0;0/1
chr12	109085786	109085786	G	A	USP30	exonic	nonsynonymous SNV	USP30:NM_032663:exon13:c.G1409A:p.S470N,USP30:NM_001301175:exon16:c.G1316A:p.S439N	.	1.081	.	.	.	Fam86_f_m_aF	0/0;0/1;0/1
chr12	109180019	109180019	C	T	ACACB	exonic	nonsynonymous SNV	ACACB:NM_001093:exon11:c.C1750T:p.R584C	.	1.027	.	0.0001	2.79E-05	Fam75_f_m_aM	0/0;0/1;0/1
chr12	109460713	109460713	C	G	KCTD10	exonic	nonsynonymous SNV	KCTD10:NM_001317395:exon3:c.G310C:p.E104Q,KCTD10:NM_001317399:exon3:c.G310C:p.E104Q,KCTD10:NM_031954:exon3:c.G310C:p.E104Q	.	1.186	7.35E-05	0.0001	2.09E-05	Fam5_f_m_aM	0/1;0/0;0/1
chr12	109586086	109586086	G	A	MVK	exonic	nonsynonymous SNV	MVK:NM_001301182:exon5:c.G436A:p.G146R,MVK:NM_000431:exon6:c.G592A:p.G198R,MVK:NM_001114185:exon6:c.G592A:p.G198R	.	1.249	.	6.54E-05	.	Fam30_f_m_aM_uM	0/1;0/0;0/1;0/0
chr12	110651307	110651307	C	T	HVCN1	exonic	nonsynonymous SNV	HVCN1:NM_001256413:exon5:c.G493A:p.D165N,HVCN1:NM_001040107:exon6:c.G553A:p.D185N,HVCN1:NM_032369:exon6:c.G553A:p.D185N	.	1.472	7.35E-05	0.0002	3.49E-05	Fam7_f_m_aM_aM_uM	0/1;0/0;0/1;0/0;0/1
chr12	111320353	111320353	G	A	CUX2	exonic	nonsynonymous SNV	CUX2:NM_001370598:exon17:c.G2158A:p.A720T,CUX2:NM_015267:exon17:c.G2344A:p.A782T	.	1.614	7.36E-05	0.0008	6.98E-05	Fam51_f_m_aM_uF	0/0;0/1;0/1;0/1
chr12	111341911	111341911	G	A	CUX2	exonic	nonsynonymous SNV	CUX2:NM_001370598:exon21:c.G3331A:p.V1111M,CUX2:NM_015267:exon21:c.G3517A:p.V1173M	.	1.691	.	.	6.98E-06	Fam27_f_m_aF_uM_uM	0/1;0/0;0/1;0/0;0/1
chr12	111418609	111418609	C	T	SH2B3	exonic	nonsynonymous SNV	SH2B3:NM_005475:exon2:c.C464T:p.P155L	.	1.118	0.0004	0.0006	0.0003	Fam20_f_m_aM_uF	0/1;0/0;0/1;0/0
chr12	112265242	112265242	C	T	HECTD4	exonic	nonsynonymous SNV	HECTD4:NM_001109662:exon16:c.G2552A:p.C851Y	.	1.467	.	1.13E-05	.	Fam51_f_m_aM_uF	0/1;0/0;0/1;0/1
chr12	112279335	112279335	T	C	HECTD4	exonic	nonsynonymous SNV	HECTD4:NM_001109662:exon9:c.A1580G:p.Y527C	.	1.268	.	.	.	Fam85_f_m_aM_aM	0/0;0/1;0/0;0/1
chr12	112405899	112405899	C	T	RPL6	exonic	nonsynonymous SNV	RPL6:NM_000970:exon6:c.G668A:p.R223Q,RPL6:NM_001024662:exon6:c.G668A:p.R223Q,RPL6:NM_001320137:exon6:c.G668A:p.R223Q,RPL6:NM_001320138:exon6:c.G668A:p.R223Q,RPL6:NM_001320139:exon6:c.G668A:p.R223Q,RPL6:NM_001320140:exon6:c.G668A:p.R223Q,RPL6:NM_001320142:exon6:c.G335A:p.R112Q,RPL6:NM_001320141:exon8:c.G668A:p.R223Q	.	1.001	0.0004	0.0004	0.0003	Fam2_f_m_aF	0/0;0/1;0/1
chr12	112454594	112454594	C	T	PTPN11	exonic	nonsynonymous SNV	PTPN11:NM_001330437:exon5:c.C556T:p.R186W,PTPN11:NM_002834:exon5:c.C556T:p.R186W,PTPN11:NM_080601:exon5:c.C556T:p.R186W	.	1.85	.	0.0002	2.79E-05	Fam61_f_m_aM	0/1;0/0;0/1
chr12	113158957	113158957	G	A	DDX54	exonic	nonsynonymous SNV	DDX54:NM_001111322:exon20:c.C2569T:p.R857C,DDX54:NM_024072:exon20:c.C2566T:p.R856C	.	1.07	.	6.91E-05	2.09E-05	Fam114_f_m_aM	0/0;0/1;0/1
chr12	113180977	113180977	G	A	DDX54	exonic	nonsynonymous SNV	DDX54:NM_001111322:exon2:c.C256T:p.R86C,DDX54:NM_024072:exon2:c.C256T:p.R86C	.	1.194	.	0.0002	4.89E-05	Fam105_f_m_aM	0/1;0/0;0/1
chr12	114674352	114674352	C	G	TBX3	exonic	nonsynonymous SNV	TBX3:NM_005996:exon6:c.G1523C:p.G508A,TBX3:NM_016569:exon7:c.G1583C:p.G528A	.	1.2	.	.	.	Fam96_f_m_aM_uF	0/1;0/0;0/1;0/1

chr12	117218154	117218154	C	T	NOS1	exonic	nonsynonymous SNV	NOS1:NM_001204213:exon27:c.G3173A:p.R1058Q,NOS1:NM_001204214:exon27:c.G3173A:p.R1058Q,NOS1:NM_000620:exon28:c.G4181A:p.R1394Q,NOS1:NM_001204218:exon29:c.G4283A:p.R1428Q	.	1.618	0.0003	0.0004	0.0001	Fam15_f_m_aM_aM	0/0;0/1;0/1;0/0
chr12	117247461	117247461	C	G	NOS1	exonic	nonsynonymous SNV	NOS1:NM_001204213:exon17:c.G1702C:p.V568L,NOS1:NM_001204214:exon17:c.G1702C:p.V568L,NOS1:NM_000620:exon18:c.G2710C:p.V904L,NOS1:NM_001204218:exon19:c.G2812C:p.V938L	.	1.005	.	.	.	Fam27_f_m_aF_uM_uM	0/1;0/0;0/1;0/0;0/1
chr12	118172615	118172615	G	A	TAOK3	exonic	nonsynonymous SNV	TAOK3:NM_001346494:exon4:c.C361T:p.R121W,TAOK3:NM_001346496:exon4:c.C361T:p.R121W,TAOK3:NM_001346495:exon5:c.C361T:p.R121W,TAOK3:NM_001346497:exon5:c.C361T:p.R121W,TAOK3:NM_001346487:exon17:c.C1768T:p.R590W,TAOK3:NM_001346489:exon17:c.C1741T:p.R581W,TAOK3:NM_016281:exon17:c.C1741T:p.R581W,TAOK3:NM_001346488:exon18:c.C1741T:p.R581W,TAOK3:NM_001346490:exon18:c.C1258T:p.R420W,TAOK3:NM_001346492:exon18:c.C1231T:p.R411W,TAOK3:NM_001346493:exon18:c.C1231T:p.R411W,TAOK3:NM_001346491:exon19:c.C1231T:p.R411W	.	1.039	.	5.58E-05	2.79E-05	Fam22_f_m_aF_aF_uF	0/0;0/1;0/1;0/1;0/1
chr12	118235611	118235611	A	C	TAOK3	exonic	nonsynonymous SNV	TAOK3:NM_001346487:exon8:c.T498G:p.F166L,TAOK3:NM_001346489:exon8:c.T498G:p.F166L,TAOK3:NM_016281:exon8:c.T498G:p.F166L,TAOK3:NM_001346488:exon9:c.T498G:p.F166L	.	1.271	.	0.0003	0.0002	Fam27_f_m_aF_uM_uM	0/0;0/1;0/1;0/0;0/0
chr12	118386143	118386143	A	G	SUDS3	exonic	nonsynonymous SNV	SUDS3:NM_022491:exon4:c.A298G:p.K100E	.	1.179	.	.	.	Fam120_f_m_aM_aM	0/1;0/0;0/1;0/0
chr12	119156605	119156605	G	T	SRRM4	exonic	nonsynonymous SNV	SRRM4:NM_194286:exon13:c.G1643T:p.S548I	.	1.123	7.35E-05	3.58E-05	1.40E-05	Fam110_f_m_aM_aM_uMs	0/1;0/0;0/1;0/1;0/1
chr12	119697827	119697827	C	T	CIT	exonic	nonsynonymous SNV	CIT:NM_007174:exon45:c.G5588A:p.R1863Q,CIT:NM_001206999:exon46:c.G5714A:p.R1905Q	.	1.569	7.34E-05	3.27E-05	.	Fam9_f_m_aM_dM_uF	0/0;0/1;0/1;0/0;0/1
chr12	119752172	119752172	G	C	CIT	exonic	nonsynonymous SNV	CIT:NM_007174:exon22:c.C2656G:p.R886G,CIT:NM_001206999:exon23:c.C2782G:p.R928G	.	1.747	.	.	.	Fam48_f_m_aM_uM	0/0;0/1;0/1;0/1
chr12	120151180	120151180	G	A	GCN1	exonic	nonsynonymous SNV	GCN1:NM_006836:exon34:c.C4274T:p.A1425V	.	1.609	.	.	6.98E-06	Fam119_f_m_aM_aM	0/0;0/1;0/1;0/0
chr12	120364731	120364731	G	A	MSI1	exonic	nonsynonymous SNV	MSI1:NM_002442:exon5:c.C292T:p.R98W	.	2.037	.	.	.	Fam12_f_m_aM_uM_aM	0/1;0/0;0/0;0/1;0/1
chr12	120462116	120462116	T	C	SRSF9	exonic	nonsynonymous SNV	SRSF9:NM_003769:exon4:c.A569G:p.Y190C	.	2.244	.	.	0	Fam119_f_m_aM_aM	0/0;0/1;0/0;0/1
chr12	120579825	120579825	C	T	POP5	exonic	nonsynonymous SNV	POP5:NM_015918:exon3:c.G262A:p.E88K	.	1.348	0.0005	0.0004	0.0002	Fam82_f_m_aM_uF	0/0;0/1;0/1;0/1
chr12	120710481	120710481	G	T	UNC119B	exonic	nonsynonymous SNV	UNC119B:NM_001080533:exon1:c.G7T:p.G3W	.	1.661	0.0001	.	4.19E-05	Fam123_f_aF	0/0;0/1
chr12	121245171	121245171	G	A	CAMKK2	exonic	nonsynonymous SNV	CAMKK2:NM_001270486:exon14:c.C1522T:p.R508C,CAMKK2:NM_153500:exon14:c.C1393T:p.R465C,CAMKK2:NM_172215:exon14:c.C1393T:p.R465C,CAMKK2:NM_172216:exon14:c.C1393T:p.R465C,CAMKK2:NM_001270485:exon15:c.C1522T:p.R508C,CAMKK2:NM_006549:exon15:c.C1522T:p.R508C,CAMKK2:NM_153499:exon15:c.C1522T:p.R508C,CAMKK2:NM_172214:exon15:c.C1522T:p.R508C,CAMKK2:NM_172226:exon15:c.C1522T:p.R508C	.	1.209	.	0.0008	8.37E-05	Fam90_f_m_aF_dM	0/1;0/0;0/1;0/0
chr12	121808279	121808279	G	C	SETD1B	exonic	nonsynonymous SNV	SETD1B:NM_001353345:exon4:c.G616C:p.E206Q	.	2.317	.	2.04E-05	7.01E-06	Fam31_f_m_aM_uF	0/0;0/1;0/1;0/0
chr12	121808279	121808279	G	C	SETD1B	exonic	nonsynonymous SNV	SETD1B:NM_001353345:exon4:c.G616C:p.E206Q	.	2.317	.	2.04E-05	7.01E-06	Fam57_f_m_aM_uF	0/0;0/1;0/1;0/1
chr12	121817895	121817895	T	C	SETD1B	exonic	nonsynonymous SNV	SETD1B:NM_001353345:exon9:c.T3409C:p.S1137P	.	1.623	.	.	.	Fam1_f_m_aM	0/1;0/0;0/1
chr12	121823438	121823438	C	T	SETD1B	exonic	nonsynonymous SNV	SETD1B:NM_001353345:exon11:c.C4859T:p.P1620L	.	1.355	0.0001	0.0003	9.82E-05	Fam17_f_m_aM_uM	0/1;0/0;0/1;0/1
chr12	121825349	121825349	C	A	SETD1B	exonic	nonsynonymous SNV	SETD1B:NM_001353345:exon12:c.C5320A:p.P1774T	.	1.464	.	4.85E-05	6.98E-06	Fam121_f_m_aF_aM	0/1;0/0;0/1;0/1
chr12	122354478	122354478	G	C	CLIP1	exonic	nonsynonymous SNV	CLIP1:NM_001247997:exon7:c.C1282G:p.L428V,CLIP1:NM_002956:exon7:c.C1282G:p.L428V,CLIP1:NM_198240:exon7:c.C1282G:p.L428V	.	1.241	.	.	6.98E-06	Fam9_f_m_aM_dM_uF	0/1;0/0;0/1;0/0;0/1
chr12	122987336	122987336	C	T	PITPNM2	exonic	nonsynonymous SNV	PITPNM2:NM_001300801:exon22:c.G3340A:p.V1114M,PITPNM2:NM_020845:exon22:c.G3358A:p.V1120M	.	2.152	0.0001	0.0002	0.0001	Fam85_f_m_aM_aM	0/1;0/0;0/1;0/0
chr12	122994873	122994873	C	T	PITPNM2	exonic	nonsynonymous SNV	PITPNM2:NM_001300801:exon14:c.G2161A:p.D721N,PITPNM2:NM_020845:exon14:c.G2161A:p.D721N	.	1.456	0.0001	0.0003	4.19E-05	Fam92_f_m_aM_raM	0/0;0/1;0/1;0/0
chr12	123465695	123465695	C	T	SNRNP35	exonic	nonsynonymous SNV	SNRNP35:NM_022717:exon2:c.C155T:p.T52I,SNRNP35:NM_180699:exon2:c.C170T:p.T57I	.	1.187	.	0.0002	1.40E-05	Fam97_f_m_aM_aF	0/0;0/1;0/1;0/0
chr12	124012603	124012603	G	T	ZNF664	exonic	nonsynonymous SNV	ZNF664:NM_001204298:exon5:c.G459T:p.R153S,ZNF664:NM_152437:exon5:c.G459T:p.R153S	.	1.08	.	.	.	Fam22_f_m_aF_aF_uF	0/0;0/1;0/0;0/1;0/1
chr12	130346489	130346489	C	T	PIWIL1	exonic	nonsynonymous SNV	PIWIL1:NM_001190971:exon5:c.C436T:p.R146C,PIWIL1:NM_004764:exon5:c.C436T:p.R146C	.	1.851	.	1.12E-05	6.98E-06	Fam21_f_m_aM_uM	0/0;0/1;0/1;0/1
chr12	131711370	131711370	C	G	SFSWAP	exonic	nonsynonymous SNV	SFSWAP:NM_001261411:exon1:c.C141G:p.D47E,SFSWAP:NM_004592:exon1:c.C141G:p.D47E	.	1.847	.	.	.	Fam115_f_m_aF_aM_aF	0/1;0/0;0/0;0/1;0/1
chr12	131714878	131714878	G	A	SFSWAP	exonic	nonsynonymous SNV	SFSWAP:NM_001261411:exon3:c.G445A:p.V149M,SFSWAP:NM_004592:exon3:c.G445A:p.V149M	.	1.836	7.34E-05	0.0002	4.19E-05	Fam103_f_m_aM	0/1;0/0;0/1
chr12	131753245	131753245	G	A	SFSWAP	exonic	nonsynonymous SNV	SFSWAP:NM_001261411:exon8:c.G1204A:p.V402M,SFSWAP:NM_004592:exon8:c.G1204A:p.V402M	.	1.293	.	9.83E-05	5.58E-05	Fam45_f_m_aM_uF	0/1;0/0;0/1;0/1
chr12	132657389	132657389	G	C	POLE	exonic	nonsynonymous SNV	POLE:NM_006231:exon28:c.C3419G:p.A1140G	.	1.572	.	.	.	Fam2_f_m_aF	0/1;0/0;0/1
chr12	132661621	132661621	G	A	POLE	exonic	nonsynonymous SNV	POLE:NM_006231:exon24:c.C2770T:p.R924C	.	1.805	0.0003	6.17E-05	3.49E-05	Fam77_f_m_aM_aF_uM_uFs	0/1;0/0;0/0;0/1;0/1;0/0
chr13	19179518	19179518	C	T	TUBA3C	exonic	nonsynonymous SNV	TUBA3C:NM_006001:exon2:c.G49A:p.G17S	.	1.594	7.35E-05	0.0001	5.58E-05	Fam5_f_m_aM	0/1;0/0;0/1
chr13	20979722	20979722	A	G	LATS2	exonic	nonsynonymous SNV	LATS2:NM_014572:exon7:c.T2741C:p.L914S	.	1.851	.	.	.	Fam105_f_m_aM	0/0;0/1;0/1

chr13	24286296	24286296	C	A	SPATA13	exonic	nonsynonymous SNV	SPATA13:NM_001286793:exon3:c.C275A:p.A92D,SPATA13:NM_001286795:exon3:c.C275A:p.A92D,SPATA13:NM_001286794:exon4:c.C341A:p.A114D,SPATA13:NM_153023:exon5:c.C509A:p.A170D,SPATA13:NM_001166271:exon6:c.C2384A:p.A795D,SPATA13:NM_001286792:exon8:c.C2570A:p.A857D	.	1.247	.	.	.	Fam38_f_m_aM	0/1;0/0;0/1
chr13	24297502	24297502	G	A	SPATA13	exonic	nonsynonymous SNV	SPATA13:NM_001286793:exon7:c.G1055A:p.R352H,SPATA13:NM_001286795:exon8:c.G1241A:p.R414H,SPATA13:NM_001286794:exon9:c.G1307A:p.R436H,SPATA13:NM_153023:exon10:c.G1475A:p.R492H,SPATA13:NM_001166271:exon11:c.G3350A:p.R1117H,SPATA13:NM_001286792:exon13:c.G3536A:p.R1179H	.	1.098	0.0004	0.0005	0.0003	Fam9_f_m_aM_dM_uF	0/1;0/0;0/1;0/0;0/1
chr13	26759091	26759091	G	A	GPR12	exonic	nonsynonymous SNV	GPR12:NM_005288:exon2:c.C737T:p.T246I	.	1.929	.	.	.	Fam61_f_m_aM	0/1;0/0;0/1
chr13	28138761	28138761	G	T	PAN3	exonic	nonsynonymous SNV	PAN3:NM_175854:exon1:c.G104T:p.G35V	.	2.095	.	0.0002	1.42E-05	Fam117_f_m_aM_aF	0/0;0/1;0/0;0/1
chr13	28272064	28272064	A	G	PAN3	exonic	nonsynonymous SNV	PAN3:NM_175854:exon14:c.A2042G:p.Q681R	.	1.795	.	.	6.98E-06	Fam116_f_m_aM_aF	0/0;0/1;0/0;0/1
chr13	28277252	28277252	T	C	PAN3	exonic	nonsynonymous SNV	PAN3:NM_175854:exon15:c.T2065C:p.S689P	.	2.032	.	.	.	Fam37_f_m_aF_uM	0/1;0/0;0/1;0/0
chr13	28427880	28427880	T	C	FLT1	exonic	nonsynonymous SNV	FLT1:NM_001159920:exon9:c.A1148G:p.Y383C,FLT1:NM_001160030:exon9:c.A1148G:p.Y383C,FLT1:NM_001160031:exon9:c.A1148G:p.Y383C,FLT1:NM_002019:exon9:c.A1148G:p.Y383C	.	1.078	.	5.59E-05	3.49E-05	Fam90_f_m_aF_dM	0/0;0/1;0/1;0/1
chr13	29536121	29536121	C	T	SLC7A1	exonic	nonsynonymous SNV	SLC7A1:NM_003045:exon3:c.G68A:p.R23Q	.	1.016	7.35E-05	9.80E-05	2.09E-05	Fam24_f_m_aM_aM	0/1;0/0;0/0;0/1
chr13	29772186	29772186	T	C	UBL3	exonic	nonsynonymous SNV	UBL3:NM_007106:exon3:c.A149G:p.E50G	.	1.692	.	.	.	Fam13_f_m_aM	0/1;0/0;0/1
chr13	31150046	31150046	C	G	HSPH1	exonic	nonsynonymous SNV	HSPH1:NM_001286505:exon6:c.G817C:p.A273P,HSPH1:NM_001286503:exon8:c.G1045C:p.A349P,HSPH1:NM_001286504:exon8:c.G1051C:p.A351P,HSPH1:NM_001349704:exon8:c.G1045C:p.A349P,HSPH1:NM_006644:exon8:c.G1045C:p.A349P	.	1.409	.	.	.	Fam87_f_m_aM_uM	0/1;0/0;0/1;0/1
chr13	32185125	32185125	C	T	FRY	exonic	nonsynonymous SNV	FRY:NM_023037:exon26:c.C3296T:p.A1099V	.	1.2	.	.	1.40E-05	Fam88_f_m_aF	0/1;0/0;0/1
chr13	33017246	33017246	A	G	KL	exonic	nonsynonymous SNV	KL:NM_004795:exon1:c.A806G:p.H269R	.	2.298	.	.	6.98E-06	Fam36_f_m_aM_uM	0/0;0/1;0/1;0/1
chr13	36126085	36126085	G	A	DCLK1	exonic	nonsynonymous SNV	DCLK1:NM_001330071:exon2:c.C53T:p.A18V,DCLK1:NM_001330072:exon2:c.C53T:p.A18V,DCLK1:NM_004734:exon2:c.C53T:p.A18V	.	1.212	.	1.12E-05	.	Fam40_f_m_aM_aM	0/1;0/0;0/0;0/1
chr13	40665962	40665962	G	A	FOXO1	exonic	nonsynonymous SNV	FOXO1:NM_002015:exon1:c.C251T:p.P84L	.	1.719	0.001	.	0.0007	Fam63_f_m_aF	0/0;0/1;0/1
chr13	40665962	40665962	G	A	FOXO1	exonic	nonsynonymous SNV	FOXO1:NM_002015:exon1:c.C251T:p.P84L	.	1.719	0.001	.	0.0007	Fam62_f_m_aM_aM	0/1;0/0;0/1;0/1
chr13	40666031	40666031	G	A	FOXO1	exonic	nonsynonymous SNV	FOXO1:NM_002015:exon1:c.C182T:p.A61V	.	1.696	7.41E-05	.	4.20E-05	Fam106_f_m_aM	0/0;0/1;0/1
chr13	41131191	41131191	C	G	KBTBD6	exonic	nonsynonymous SNV	KBTBD6:NM_152903:exon1:c.G1321C:p.G441R	.	2.305	.	.	.	Fam88_f_m_aF	0/1;0/0;0/1
chr13	41192409	41192409	G	A	KBTBD7	exonic	nonsynonymous SNV	KBTBD7:NM_032138:exon1:c.C1849T:p.R617C	.	1.936	.	0.0001	2.79E-05	Fam40_f_m_aM_aM	0/0;0/0;0/0;0/1
chr13	43322494	43322494	C	T	ENOX1	exonic	nonsynonymous SNV	ENOX1:NM_001347963:exon10:c.G1256A:p.R419Q,ENOX1:NM_001347965:exon10:c.G1151A:p.R384Q,ENOX1:NM_001347967:exon10:c.G1151A:p.R384Q,ENOX1:NM_001347970:exon10:c.G1034A:p.R345Q,ENOX1:NM_001347971:exon10:c.G1034A:p.R345Q,ENOX1:NM_001127615:exon11:c.G1151A:p.R384Q,ENOX1:NM_001242863:exon11:c.G1151A:p.R384Q,ENOX1:NM_001347969:exon11:c.G1151A:p.R384Q,ENOX1:NM_017993:exon11:c.G1151A:p.R384Q,ENOX1:NM_001347964:exon12:c.G1151A:p.R384Q,ENOX1:NM_001347966:exon12:c.G1151A:p.R384Q,ENOX1:NM_001347968:exon12:c.G1151A:p.R384Q	.	1.64	7.34E-05	0.0001	4.19E-05	Fam101_f_m_aM	0/1;0/0;0/1
chr13	45411392	45411392	C	T	SLC25A30	exonic	nonsynonymous SNV	SLC25A30:NM_001010875:exon2:c.G34A:p.G12R	.	1.048	.	6.55E-05	.	Fam16_f_m_aM_aM	0/1;0/0;0/0;0/1
chr13	45481237	45481237	T	A	COG3	exonic	nonsynonymous SNV	COG3:NM_031431:exon5:c.T557A:p.L186H	.	1.063	.	.	.	Fam77_f_m_aM_aF_uM_uFs	0/0;0/1;0/1;0/1;0/1;0/0
chr13	46064720	46064720	G	A	CPB2	exonic	nonsynonymous SNV	CPB2:NM_001278541:exon7:c.C613T:p.R205C,CPB2:NM_001872:exon8:c.C724T:p.R242C	.	1.402	.	1.12E-05	.	Fam35_f_m_aF_uM	0/1;0/0;0/1;0/0
chr13	46154854	46154854	A	G	LCP1	exonic	nonsynonymous SNV	LCP1:NM_002298:exon6:c.T524C:p.I175T	.	1.249	7.34E-05	3.27E-05	1.40E-05	Fam16_f_m_aM_aM	0/0;0/1;0/0;0/1
chr13	49931134	49931134	C	T	SPRYD7	exonic	nonsynonymous SNV	SPRYD7:NM_020456:exon2:c.G107A:p.G36E	.	1.575	7.36E-05	0.0006	0.0001	Fam109_f_m_aM	0/1;0/0;0/1
chr13	50843361	50843361	C	G	DLEU7	exonic	nonsynonymous SNV	DLEU7:NM_001306135:exon1:c.G286C:p.G96R,DLEU7:NM_198989:exon1:c.G286C:p.G96R	.	2.082	.	.	1.40E-05	Fam5_f_m_aM	0/0;0/1;0/1
chr13	75299365	75299365	G	A	TBC1D4	exonic	nonsynonymous SNV	TBC1D4:NM_001286659:exon15:c.C2932T:p.R978C,TBC1D4:NM_001286658:exon16:c.C3097T:p.R1033C,TBC1D4:NM_014832:exon17:c.C3121T:p.R1041C	.	1.284	7.34E-05	3.27E-05	2.79E-05	Fam81_f_m_aM_uM	0/1;0/0;0/1;0/1
chr13	75481643	75481643	C	T	TBC1D4	exonic	nonsynonymous SNV	TBC1D4:NM_001286658:exon1:c.G125A:p.G42E,TBC1D4:NM_001286659:exon1:c.G125A:p.G42E,TBC1D4:NM_014832:exon1:c.G125A:p.G42E	.	1.252	.	.	.	Fam6_f_m_aM	0/1;0/0;0/1
chr13	77007282	77007282	C	T	FBXL3	exonic	nonsynonymous SNV	FBXL3:NM_012158:exon5:c.G1150A:p.G384S	.	2.024	.	.	.	Fam44_f_m_aM_uF	0/1;0/0;0/1;0/0
chr13	77067627	77067627	C	T	MYCBP2	exonic	nonsynonymous SNV	MYCBP2:NM_015057:exon71:c.G12409A:p.G4137S	.	1.891	.	.	.	Fam116_f_m_aM_aF	0/1;0/0;0/1;0/1
chr13	77158091	77158091	C	T	MYCBP2	exonic	nonsynonymous SNV	MYCBP2:NM_015057:exon45:c.G6616A:p.G2206R	.	1.066	.	.	.	Fam113_f_m_aF_raM_uF	0/0;0/0;0/0;0/1;0/0
chr13	79344303	79344303	T	C	RBM26	exonic	nonsynonymous SNV	RBM26:NM_001286632:exon15:c.A2132G:p.Q711R,RBM26:NM_022118:exon15:c.A2123G:p.Q708R,RBM26:NM_001286631:exon16:c.A2210G:p.Q737R,RBM26:NM_001366735:exon16:c.A2204G:p.Q735R	.	1.2	.	1.12E-05	.	Fam112_f_m_aM	0/1;0/0;0/1
chr13	79481278	79481278	G	C	NDFIP2	exonic	nonsynonymous SNV	NDFIP2:NM_001161407:exon1:c.G75C:p.E25D,NDFIP2:NM_019080:exon1:c.G75C:p.E25D	.	1.042	.	.	.	Fam23_f_m_aM_dF_uFs	0/1;0/0;0/1;0/1;0/0
chr13	87676500	87676500	C	A	SLITRK5	exonic	nonsynonymous SNV	SLITRK5:NM_015567:exon2:c.C1112A:p.P371H	.	1.256	.	5.59E-05	2.09E-05	Fam100_f_m_aF	0/1;0/0;0/1

chr13	95641820	95641820	G	T	DZIP1	exonic	nonsynonymous SNV	DZIP1:NM_014934:exon5:c.C72A:p.S24R,DZIP1:NM_198968:exon5:c.C72A:p.S24R	.	1.16	.	.	2.10E-05	Fam43_f_m_aM	0/1;0/0;0/1
chr13	98014064	98014064	A	T	IPO5	exonic	nonsynonymous SNV	IPO5:NM_002271:exon22:c.A2175T:p.E725D	.	1.873	0.0005	0.001	0.0005	Fam19_f_m_aM	0/0;0/1;0/1
chr13	98902435	98902435	A	C	DOCK9	exonic	nonsynonymous SNV	DOCK9:NM_001130049:exon12:c.T1236G:p.I412M,DOCK9:NM_001130050:exon12:c.T1233G:p.I411M,DOCK9:NM_001366677:exon12:c.T1269G:p.I423M,DOCK9:NM_001366679:exon12:c.T1233G:p.I411M,DOCK9:NM_001366680:exon12:c.T1233G:p.I411M,DOCK9:NM_001366681:exon12:c.T1233G:p.I411M,DOCK9:NM_001366682:exon12:c.T1233G:p.I411M,DOCK9:NM_001366683:exon12:c.T1233G:p.I411M,DOCK9:NM_001366684:exon12:c.T1233G:p.I411M	.	1.035		3.37E-05	.	Fam80_f_m_aM_uM	0/0;0/1;0/1;0/0
chr13	99529570	99529570	T	C	TM9SF2	exonic	nonsynonymous SNV	TM9SF2:NM_004800:exon4:c.T437C:p.M146T	.	1.423	.	0.0001	1.40E-05	Fam98_f_m_aM	0/0;0/1;0/1
chr13	101143192	101143192	C	A	NALCN	exonic	nonsynonymous SNV	NALCN:NM_001350750:exon16:c.G1919T:p.R640L,NALCN:NM_001350751:exon16:c.G1919T:p.R640L,NALCN:NM_001350748:exon17:c.G2006T:p.R669L,NALCN:NM_001350749:exon17:c.G2006T:p.R669L,NALCN:NM_052867:exon17:c.G2006T:p.R669L	.	1.228	.	.	.	Fam18_f_m_aM_uF	0/1;0/0;0/1;0/0
chr13	101567725	101567725	T	C	ITGBL1	exonic	nonsynonymous SNV	ITGBL1:NM_001271756:exon2:c.T64C:p.C22R,ITGBL1:NM_004791:exon3:c.T343C:p.C115R	.	1.058	.	.	.	Fam38_f_m_aM	0/1;0/0;0/1
chr13	110170577	110170577	G	A	COL4A1	exonic	nonsynonymous SNV	COL4A1:NM_001845:exon42:c.C3712T:p.R1238C	.	1.408	0.0004	0.0005	0.0001	Fam100_f_m_aF	0/0;0/1;0/1
chr13	110457425	110457425	G	C	COL4A2	exonic	nonsynonymous SNV	COL4A2:NM_001846:exon21:c.G1422C:p.K474N	.	1.087	.	.	.	Fam110_f_m_aM_aM_uMs	0/0;0/1;0/0;0/1;0/0
chr13	110503184	110503184	G	A	COL4A2	exonic	nonsynonymous SNV	COL4A2:NM_001846:exon42:c.G3941A:p.G1314E	.	1.16	.	.	.	Fam77_f_m_aM_aF_uM_uFs	0/1;0/0;0/1;0/0;0/1;0/0
chr13	110561447	110561447	A	G	RAB20	exonic	nonsynonymous SNV	RAB20:NM_017817:exon1:c.T73C:p.Y25H	.	1.557	0.0005	0.0006	0.0003	Fam94_f_m_aM	0/0;0/1;0/1
chr13	111217851	111217851	G	C	ARHGEF7	exonic	nonsynonymous SNV	ARHGEF7:NM_001320851:exon3:c.G170C:p.S57T,ARHGEF7:NM_001330598:exon3:c.G170C:p.S57T,ARHGEF7:NM_001354047:exon3:c.G425C:p.S142T,ARHGEF7:NM_001354053:exon3:c.G170C:p.S57T,ARHGEF7:NM_001354056:exon3:c.G170C:p.S57T,ARHGEF7:NM_001113512:exon4:c.G554C:p.S185T,ARHGEF7:NM_001113513:exon4:c.G170C:p.S57T,ARHGEF7:NM_001320853:exon4:c.G395C:p.S132T,ARHGEF7:NM_001354050:exon4:c.G170C:p.S57T,ARHGEF7:NM_001354051:exon4:c.G170C:p.S57T,ARHGEF7:NM_001354060:exon4:c.G170C:p.S57T,ARHGEF7:NM_003899:exon4:c.G170C:p.S57T,ARHGEF7:NM_001320852:exon5:c.G641C:p.S214T,ARHGEF7:NM_001330597:exon5:c.G170C:p.S57T,ARHGEF7:NM_001354046:exon5:c.G641C:p.S214T,ARHGEF7:NM_001354048:exon5:c.G170C:p.S57T,ARHGEF7:NM_001354049:exon5:c.G170C:p.S57T,ARHGEF7:NM_001354052:exon5:c.G170C:p.S57T,ARHGEF7:NM_001354054:exon5:c.G170C:p.S57T,ARHGEF7:NM_001354057:exon5:c.G170C:p.S57T,ARHGEF7:NM_145735:exon5:c.G641C:p.S214T,ARHGEF7:NM_001113511:exon6:c.G704C:p.S235T,ARHGEF7:NM_001354058:exon6:c.G170C:p.S57T,ARHGEF7:NM_001354059:exon6:c.G170C:p.S57T	.	1.44	.	.	.	Fam70_f_m_aM	0/1;0/0;0/1
chr13	111292198	111292198	A	G	ARHGEF7	exonic	nonsynonymous SNV	ARHGEF7:NM_001320854:exon14:c.A1510G:p.S504G,ARHGEF7:NM_001354055:exon14:c.A1510G:p.S504G,ARHGEF7:NM_001354061:exon15:c.A1372G:p.S458G,ARHGEF7:NM_001330598:exon17:c.A1744G:p.S582G,ARHGEF7:NM_001354047:exon17:c.A1999G:p.S667G,ARHGEF7:NM_001354056:exon17:c.A1744G:p.S582G,ARHGEF7:NM_001113512:exon18:c.A2128G:p.S710G,ARHGEF7:NM_001354050:exon18:c.A1744G:p.S582G,ARHGEF7:NM_001354051:exon18:c.A1744G:p.S582G,ARHGEF7:NM_001354060:exon18:c.A1744G:p.S582G,ARHGEF7:NM_001330597:exon19:c.A1744G:p.S582G,ARHGEF7:NM_001354046:exon19:c.A2215G:p.S739G,ARHGEF7:NM_001354048:exon19:c.A1744G:p.S582G,ARHGEF7:NM_001354049:exon19:c.A1744G:p.S582G,ARHGEF7:NM_001354057:exon19:c.A1744G:p.S582G,ARHGEF7:NM_145735:exon19:c.A2215G:p.S739G,ARHGEF7:NM_001113511:exon20:c.A2278G:p.S760G,ARHGEF7:NM_001354058:exon20:c.A1744G:p.S582G,ARHGEF7:NM_001354059:exon20:c.A1744G:p.S582G	.	1.432	0.0001	3.35E-05	2.09E-05	Fam85_f_m_aM_aM	0/1;0/0;0/1;0/0
chr13	112856059	112856059	C	T	ATP11A	exonic	nonsynonymous SNV	ATP11A:NM_015205:exon20:c.C2392T:p.R798C,ATP11A:NM_032189:exon20:c.C2392T:p.R798C	.	1.182	.	9.85E-05	2.09E-05	Fam37_f_m_aF_uM	0/1;0/0;0/1;0/0
chr13	114018110	114018110	C	T	RASA3	exonic	nonsynonymous SNV	RASA3:NM_001320822:exon11:c.G989A:p.R330Q,RASA3:NM_007368:exon11:c.G1085A:p.R362Q	.	1.284	.	2.18E-05	4.19E-05	Fam42_f_m_aM_uF	0/1;0/0;0/1;0/1
chr14	20460788	20460788	G	C	PIP4P1	exonic	nonsynonymous SNV	PIP4P1:NM_001100814:exon2:c.C221G:p.P74R,PIP4P1:NM_144568:exon2:c.C200G:p.P67R	.	1.598	0.0006	0.0008	0.0002	Fam15_f_m_aM_aM	0/1;0/0;0/1;0/1
chr14	20996636	20996636	G	A	METTL17	exonic	nonsynonymous SNV	METTL17:NM_001029991:exon13:c.G1190A:p.R397H,METTL17:NM_022734:exon13:c.G1190A:p.R397H	.	1.1	7.34E-05	0.0003	4.19E-05	Fam88_f_m_aF	0/1;0/0;0/1
chr14	21093244	21093244	G	A	ZNF219	exonic	nonsynonymous SNV	ZNF219:NM_001101672:exon3:c.C53T:p.P18L,ZNF219:NM_001102454:exon3:c.C53T:p.P18L,ZNF219:NM_016423:exon3:c.C53T:p.P18L	.	1.871	.	1.23E-05	.	Fam121_f_m_aF_aM	0/0;0/1;0/1;0/0
chr14	21403633	21403633	C	T	CHD8	exonic	nonsynonymous SNV	CHD8:NM_001170629:exon17:c.G3338A:p.R1113H,CHD8:NM_020920:exon17:c.G2501A:p.R834H	.	2.622	.	.	6.99E-06	Fam91_m_aM_dM_aM_dM	0/0;0/0;0/0;0/1;0/0
chr14	21409849	21409849	A	G	CHD8	splicing	.	.	0.144	.	.	9.92E-05	2.09E-05	Fam62_f_m_aM_aM	0/1;0/0;0/1;0/1
chr14	22842475	22842475	G	A	MMP14	exonic	nonsynonymous SNV	MMP14:NM_004995:exon4:c.G446A:p.R149H	.	1.472	0.0006	0.0002	5.58E-05	Fam119_f_m_aM_aM	0/1;0/0;0/1;0/1

chr14	22988015	22988015	C	T	C14orf93	exonic	nonsynonymous SNV	C14orf93:NM_001130708:exon6:c.G1085A:p.G362E,C14orf93:NM_001282970:exon6:c.G1085A:p.G362E,C14orf93:NM_021944:exon6:c.G1085A:p.G362E,C14orf93:NM_001130706:exon7:c.G1085A:p.G362E,C14orf93:NM_001282968:exon7:c.G545A:p.G182E,C14orf93:NM_001282969:exon7:c.G545A:p.G182E	.	1.216	.	5.60E-05	6.99E-06	Fam53_f_m_aM	0/1;0/0;0/1
chr14	22996193	22996193	G	T	C14orf93	exonic	nonsynonymous SNV	C14orf93:NM_001130708:exon3:c.C673A:p.P225T,C14orf93:NM_001282970:exon3:c.C673A:p.P225T,C14orf93:NM_021944:exon3:c.C673A:p.P225T,C14orf93:NM_001130706:exon4:c.C673A:p.P225T,C14orf93:NM_001282968:exon4:c.C133A:p.P45T,C14orf93:NM_001282969:exon4:c.C133A:p.P45T	.	1.119	.	.	.	Fam121_f_m_aF_aM	0/1;0/0;0/1;0/1
chr14	23053548	23053548	G	C	CDH24	exonic	nonsynonymous SNV	CDH24:NM_022478:exon7:c.C1174G:p.L392V,CDH24:NM_144985:exon7:c.C1174G:p.L392V	.	1.032	.	0.0001	2.79E-05	Fam62_f_m_aM_aM	0/1;0/0;0/1;0/1
chr14	23061333	23061333	C	A	ACIN1	exonic	nonsynonymous SNV	ACIN1:NM_001164816:exon10:c.G1382T:p.R461L,ACIN1:NM_001164817:exon11:c.G1289T:p.R430L,ACIN1:NM_001164815:exon16:c.G3443T:p.R1148L,ACIN1:NM_001164814:exon17:c.G3524T:p.R1175L,ACIN1:NM_014977:exon17:c.G3563T:p.R1188L	.	2.298	0.0001	0.0002	0.0002	Fam54_f_m_aM_uF	0/1;0/0;0/1;0/0
chr14	23061348	23061348	C	T	ACIN1	exonic	nonsynonymous SNV	ACIN1:NM_001164816:exon10:c.G1367A:p.R456H,ACIN1:NM_001164817:exon11:c.G1274A:p.R425H,ACIN1:NM_001164815:exon16:c.G3428A:p.R1143H,ACIN1:NM_001164814:exon17:c.G3509A:p.R1170H,ACIN1:NM_014977:exon17:c.G3548A:p.R1183H	.	2.299	.	6.56E-05	2.79E-05	Fam85_f_m_aM_aM	0/1;0/0;0/0;0/1
chr14	23307895	23307895	A	G	BCL2L2;BCL2L2-PA	exonic	nonsynonymous SNV	BCL2L2:NM_001199839:exon3:c.A128G:p.H43R,BCL2L2-PABPN1:NM_001199864:exon3:c.A128G:p.H43R,BCL2L2:NM_004050:exon3:c.A128G:p.H43R	.	1.536	0.0001	0.0002	0.0002	Fam64_f_m_aM	0/0;0/1;0/1
chr14	23324237	23324237	C	T	BCL2L2-PABPN1;P	exonic	nonsynonymous SNV	PABPN1:NM_001360552:exon5:c.C445T:p.R149C,PABPN1:NM_001360551:exon6:c.C829T:p.R277C,PABPN1:NM_004643:exon6:c.C829T:p.R277C,BCL2L2-PABPN1:NM_001199864:exon8:c.C910T:p.R304C	.	2.133	0.0001	1.12E-05	6.98E-06	Fam108_f_m_aM	0/0;0/1;0/1
chr14	23393730	23393730	T	A	MYH6	exonic	nonsynonymous SNV	MYH6:NM_002471:exon22:c.A2864T:p.D955V	.	1.357	.	.	.	Fam64_f_m_aM	0/0;0/1;0/1
chr14	23403422	23403422	A	T	MYH6	exonic	nonsynonymous SNV	MYH6:NM_002471:exon10:c.T824A:p.I275N	.	1.113	0.0001	0.0002	0.0002	Fam72_f_m_aF_uF	0/1;0/0;0/1;0/0
chr14	23416063	23416063	C	T	MYH7	exonic	nonsynonymous SNV	MYH7:NM_000257:exon34:c.G4894A:p.A1632T	.	1.522	.	0.0002	2.79E-05	Fam49_f_m_aM	0/0;0/1;0/1
chr14	23418234	23418234	C	T	MYH7	exonic	nonsynonymous SNV	MYH7:NM_000257:exon30:c.G4145A:p.R1382Q	.	1.674	.	6.17E-05	1.40E-05	Fam65_f_m_aM_uF_dF	0/1;0/0;0/1;0/0;0/1
chr14	23421008	23421008	C	A	MYH7	exonic	nonsynonymous SNV	MYH7:NM_000257:exon26:c.G3286T:p.D1096Y	.	1.278	0.0002	0.0003	0.0001	Fam48_f_m_aM_uM	0/1;0/0;0/1;0/1
chr14	24055701	24055701	G	T	CARMIL3	exonic	nonsynonymous SNV	CARMIL3:NM_138360:exon10:c.G682T:p.G228C	.	1.061	.	6.55E-05	1.40E-05	Fam121_f_m_aF_aM	0/0;0/1;0/1;0/0
chr14	24060044	24060044	C	T	CARMIL3	exonic	nonsynonymous SNV	CARMIL3:NM_138360:exon23:c.C1943T:p.T648I	.	1.053	.	.	.	Fam77_f_m_aM_aF_uM_uFs	0/1;0/0;0/0;0/1;0/0;0/0
chr14	24155272	24155272	G	A	RNF31	exonic	nonsynonymous SNV	RNF31:NM_001310332:exon12:c.G1793A:p.R598H,RNF31:NM_017999:exon12:c.G2246A:p.R749H	.	1.773	.	0.0002	6.98E-06	Fam56_f_m_aF_aM	0/1;0/0;0/0;0/1
chr14	24427853	24427853	C	T	CBLN3	exonic	nonsynonymous SNV	CBLN3:NM_001039771:exon3:c.G554A:p.R185Q	.	1.134	0.0003	0.0005	9.78E-05	Fam46_f_m_aM_uM	0/0;0/1;0/1;0/0
chr14	30719365	30719365	G	A	SCFD1	exonic	nonsynonymous SNV	SCFD1:NM_001283033:exon18:c.G1169A:p.R390Q,SCFD1:NM_001257376:exon19:c.G1448A:p.R483Q,SCFD1:NM_001283031:exon20:c.G1169A:p.R390Q,SCFD1:NM_001283032:exon20:c.G1547A:p.R516Q,SCFD1:NM_182835:exon20:c.G1523A:p.R508Q,SCFD1:NM_016106:exon21:c.G1724A:p.R575Q	.	1.182	7.36E-05	7.49E-05	2.10E-05	Fam28_f_m_aF_uF	0/1;0/0;0/1;0/1
chr14	31109504	31109504	C	G	HECTD1	exonic	nonsynonymous SNV	HECTD1:NM_015382:exon36:c.G6373C:p.V2125L	.	1.344	.	1.12E-05	.	Fam119_f_m_aM_aM	0/0;0/1;0/1;0/0
chr14	31144223	31144223	A	G	HECTD1	exonic	nonsynonymous SNV	HECTD1:NM_015382:exon17:c.T2666C:p.M889T	.	1.133	.	.	.	Fam38_f_m_aM	0/0;0/1;0/1
chr14	34783829	34783829	C	T	BAZ1A	exonic	nonsynonymous SNV	BAZ1A:NM_182648:exon14:c.G1834A:p.A612T,BAZ1A:NM_013448:exon15:c.G1930A:p.A644T	.	1.187	.	.	.	Fam77_f_m_aM_aF_uM_uFs	0/0;0/1;0/0;0/1;0/0;0/1
chr14	35116626	35116626	G	A	PPP2R3C	exonic	nonsynonymous SNV	PPP2R3C:NM_017917:exon2:c.C170T:p.P57L	.	1.236	.	.	.	Fam96_f_m_aM_uF	0/0;0/1;0/1;0/1
chr14	35672956	35672956	T	A	RALGAPA1	exonic	nonsynonymous SNV	RALGAPA1:NM_001330075:exon24:c.A4843T:p.M1615L,RALGAPA1:NM_001346243:exon24:c.A3466T:p.M1156L,RALGAPA1:NM_001346246:exon24:c.A3466T:p.M1156L,RALGAPA1:NM_001346248:exon24:c.A4843T:p.M1615L,RALGAPA1:NM_014990:exon24:c.A3466T:p.M1156L,RALGAPA1:NM_194301:exon24:c.A3466T:p.M1156L,RALGAPA1:NM_001283043:exon25:c.A3505T:p.M1169L,RALGAPA1:NM_001283044:exon25:c.A3607T:p.M1203L,RALGAPA1:NM_001346245:exon25:c.A3607T:p.M1203L,RALGAPA1:NM_001346247:exon25:c.A3607T:p.M1203L,RALGAPA1:NM_001346249:exon25:c.A4984T:p.M1662L	.	1.578	.	1.20E-05	2.09E-05	Fam113_f_m_aF_raM_uF	0/0;0/1;0/1;0/0;0/0
chr14	35684947	35684947	C	G	RALGAPA1	exonic	nonsynonymous SNV	RALGAPA1:NM_001330075:exon19:c.G4135C:p.D1379H,RALGAPA1:NM_001346243:exon19:c.G2758C:p.D920H,RALGAPA1:NM_001346246:exon19:c.G2758C:p.D920H,RALGAPA1:NM_001346248:exon19:c.G4135C:p.D1379H,RALGAPA1:NM_014990:exon19:c.G2758C:p.D920H,RALGAPA1:NM_194301:exon19:c.G2758C:p.D920H,RALGAPA1:NM_001283043:exon20:c.G2797C:p.D933H,RALGAPA1:NM_001283044:exon20:c.G2899C:p.D967H,RALGAPA1:NM_001346245:exon20:c.G2899C:p.D967H,RALGAPA1:NM_001346247:exon20:c.G2899C:p.D967H,RALGAPA1:NM_001346249:exon20:c.G4276C:p.D1426H	.	1.484	.	3.36E-05	6.98E-06	Fam63_f_m_aF	0/1;0/0;0/1
chr14	38210311	38210311	T	C	SSTR1	exonic	nonsynonymous SNV	SSTR1:NM_001049:exon3:c.T922C:p.S308P	.	1.552	.	1.12E-05	.	Fam56_f_m_aF_aM	0/1;0/0;0/1;0/1
chr14	38255379	38255379	A	G	CLEC14A	exonic	nonsynonymous SNV	CLEC14A:NM_175060:exon1:c.T644C:p.L215P	.	1.733	.	.	.	Fam122_f_m_aM	0/1;0/0;0/1

chr14	50635366	50635366	C	A	SAV1	exonic	nonsynonymous SNV	SAV1:NM_021818:exon5:c.G969T:p.K323N	.	1.003	.	6.54E-05	.		Fam116_f_m_aM_aF	0/0;0/1;0/1;0/1
chr14	52268375	52268375	G	T	PTGDR	exonic	nonsynonymous SNV	PTGDR:NM_000953:exon1:c.G561T:p.M187I,PTGDR:NM_001281469:exon1:c.G561T:p.M187I	.	1.123	.	3.27E-05	.		Fam120_f_m_aM_aM	0/1;0/0;0/1;0/1
chr14	52274846	52274846	G	C	PTGDR	exonic	nonsynonymous SNV	PTGDR:NM_000953:exon2:c.G962C:p.W321S	.	1.377	.	.	.		Fam88_f_m_aF	0/0;0/1;0/1
chr14	53054576	53054576	G	A	DDHD1	exonic	nonsynonymous SNV	DDHD1:NM_001160148:exon11:c.C2299T:p.R767C,DDHD1:NM_030637:exon11:c.C2299T:p.R767C,DDHD1:NM_001160147:exon12:c.C2320T:p.R774C	.	1.515	.	3.28E-05	.		Fam115_f_m_aF_aM_aF	0/1;0/0;0/1;0/0;0/1
chr14	53152509	53152509	A	G	DDHD1	exonic	nonsynonymous SNV	DDHD1:NM_001160147:exon1:c.T590C:p.L197P,DDHD1:NM_001160148:exon1:c.T590C:p.L197P,DDHD1:NM_030637:exon1:c.T590C:p.L197P	.	1.622	.	.	.		Fam37_f_m_aF_uM	0/1;0/0;0/1;0/0
chr14	54737225	54737225	A	T	SAMD4A	exonic	nonsynonymous SNV	SAMD4A:NM_015589:exon3:c.A917T:p.E306V	.	1.179	.	.	.		Fam84_f_m_aF	0/0;0/1;0/1
chr14	54760329	54760329	G	T	SAMD4A	exonic	nonsynonymous SNV	SAMD4A:NM_001161577:exon2:c.G118T:p.D40Y,SAMD4A:NM_001161576:exon5:c.G1081T:p.D361Y,SAMD4A:NM_015589:exon6:c.G1345T:p.D449Y	.	1.27	.	3.40E-05	6.98E-06		Fam97_f_m_aM_aF	0/1;0/0;0/0;0/1
chr14	55380648	55380648	A	G	ATG14	exonic	nonsynonymous SNV	ATG14:NM_014924:exon7:c.T920C:p.L307P	.	1.249	.	.	.		Fam60_f_m_aF	0/1;0/0;0/1
chr14	59352581	59352581	A	G	DAAM1	exonic	nonsynonymous SNV	DAAM1:NM_001270520:exon18:c.A2216G:p.H739R,DAAM1:NM_014992:exon18:c.A2246G:p.H749R	.	1.051	.	1.12E-05	6.98E-06		Fam14_f_m_aM_aM	0/0;0/1;0/1;0/1
chr14	60245952	60245956	AAGA	-	PPM1A	exonic	frameshift deletion	PPM1A:NM_177952:exon1:c.105_109del:p.K40Gfs*8	0.396	.	0.0006	8.99E-05	6.98E-06		Fam32_f_m_aM_uM	0/0;0/1;0/1;0/1
chr14	60724049	60724049	T	C	SIX4	exonic	nonsynonymous SNV	SIX4:NM_017420:exon1:c.A26G:p.Q9R	.	1.23	.	.	.		Fam5_f_m_aM	0/0;0/1;0/1
chr14	61450860	61450860	G	A	PRKCH	exonic	nonsynonymous SNV	PRKCH:NM_006255:exon6:c.G721A:p.G241R	.	1.46	.	1.12E-05	.		Fam14_f_m_aM_aM	0/1;0/0;0/0;0/1
chr14	61549752	61549752	T	C	PRKCH	exonic	nonsynonymous SNV	PRKCH:NM_006255:exon14:c.T1973C:p.I658T	.	1.144	.	.	.		Fam99_f_m_aM_aM	0/0;0/1;0/1;0/0
chr14	61721533	61721533	T	G	HIF1A	exonic	nonsynonymous SNV	HIF1A:NM_001243084:exon3:c.T323G:p.M108R,HIF1A:NM_001530:exon3:c.T251G:p.M84R,HIF1A:NM_181054:exon3:c.T251G:p.M84R	.	1.367	0.0003	6.56E-05	2.09E-05		Fam15_f_m_aM_aM	0/0;0/1;0/1;0/0
chr14	62779855	62779855	C	T	KCNH5	exonic	nonsynonymous SNV	KCNH5:NM_139318:exon10:c.G1892A:p.R631Q	.	1.866	.	.	6.98E-06		Fam110_f_m_aM_aM_uMs	0/1;0/0;0/1;0/1;0/1
chr14	63392013	63392013	T	C	PPP2R5E	exonic	nonsynonymous SNV	PPP2R5E:NM_001282181:exon8:c.A634G:p.I212V,PPP2R5E:NM_001282182:exon8:c.A634G:p.I212V,PPP2R5E:NM_001282179:exon9:c.A862G:p.I288V,PPP2R5E:NM_001282180:exon9:c.A862G:p.I288V,PPP2R5E:NM_006246:exon9:c.A862G:p.I288V	.	1.024	0.0001	3.45E-05	6.98E-06		Fam82_f_m_aM_uF	0/0;0/1;0/1;0/1
chr14	64418025	64418025	G	A	MTHFD1	splicing	.	.	0.511	.	.	.	.		Fam59_f_m_aF_uM	0/0;0/1;0/1;0/0
chr14	64454823	64454823	G	A	MTHFD1	exonic	nonsynonymous SNV	MTHFD1:NM_001364837:exon26:c.G2666A:p.R889H,MTHFD1:NM_005956:exon26:c.G2666A:p.R889H	.	1.267	7.35E-05	6.17E-05	3.49E-05		Fam78_f_m_aF_uM	0/0;0/1;0/1;0/0
chr14	64801821	64801821	T	A	SPTB	exonic	nonsynonymous SNV	SPTB:NM_001024858:exon5:c.A580T:p.N194Y,SPTB:NM_001355436:exon6:c.A580T:p.N194Y,SPTB:NM_001355437:exon6:c.A580T:p.N194Y	.	1.812	.	.	.		Fam70_f_m_aM	0/1;0/0;0/1
chr14	68880003	68880003	G	C	ACTN1	exonic	nonsynonymous SNV	ACTN1:NM_001102:exon18:c.C2239G:p.Q747E,ACTN1:NM_001130004:exon18:c.C2239G:p.Q747E,ACTN1:NM_001130005:exon18:c.C2239G:p.Q747E	.	1.92	0.0001	0.0001	6.98E-05		Fam77_f_m_aM_aF_uM_uFs	0/1;0/0;0/1;0/1;0/0;0/0
chr14	69055254	69055254	C	T	DCAF5	exonic	nonsynonymous SNV	DCAF5:NM_001284206:exon9:c.G1429A:p.D477N,DCAF5:NM_001284207:exon9:c.G1186A:p.D396N,DCAF5:NM_003861:exon9:c.G1432A:p.D478N	.	1.873	7.35E-05	7.46E-05	.		Fam37_f_m_aF_uM	0/0;0/1;0/1;0/0
chr14	69338665	69338665	G	T	GALNT16	exonic	nonsynonymous SNV	GALNT16:NM_001168368:exon10:c.G982T:p.V328L,GALNT16:NM_020692:exon10:c.G982T:p.V328L	.	1.108	0.0001	4.47E-05	4.19E-05		Fam76_f_m_aM_uM	0/1;0/0;0/1;0/0
chr14	70801008	70801008	C	T	MAP3K9	exonic	nonsynonymous SNV	MAP3K9:NM_001284230:exon2:c.G479A:p.R160H,MAP3K9:NM_033141:exon2:c.G479A:p.R160H	.	1.107	0.0003	0.0002	0.0001		Fam7_f_m_aM_aM_uM	0/1;0/0;0/0;0/1;0/0
chr14	70808885	70808885	G	A	MAP3K9	exonic	nonsynonymous SNV	MAP3K9:NM_001284230:exon1:c.C287T:p.T96I,MAP3K9:NM_033141:exon1:c.C287T:p.T96I	.	1.031	.	2.54E-05	1.40E-05		Fam45_f_m_aM_uF	0/0;0/1;0/1;0/0
chr14	71588320	71588320	G	A	SIPA1L1	exonic	nonsynonymous SNV	SIPA1L1:NM_001284246:exon2:c.G448A:p.D150N,SIPA1L1:NM_001284247:exon4:c.G448A:p.D150N,SIPA1L1:NM_001354285:exon5:c.G448A:p.D150N,SIPA1L1:NM_001354287:exon5:c.G448A:p.D150N,SIPA1L1:NM_001354288:exon5:c.G448A:p.D150N,SIPA1L1:NM_001354286:exon6:c.G448A:p.D150N,SIPA1L1:NM_001284245:exon7:c.G448A:p.D150N,SIPA1L1:NM_015556:exon7:c.G448A:p.D150N	.	1.055	.	0.0002	7.68E-05		Fam55_f_m_aM_aM_dM	0/0;0/1;0/1;0/1;0/1
chr14	71671187	71671187	C	G	SIPA1L1	exonic	nonsynonymous SNV	SIPA1L1:NM_001284246:exon8:c.C2324G:p.P775R,SIPA1L1:NM_001284247:exon10:c.C2324G:p.P775R,SIPA1L1:NM_001354285:exon11:c.C2324G:p.P775R,SIPA1L1:NM_001354287:exon11:c.C2324G:p.P775R,SIPA1L1:NM_001354288:exon11:c.C2324G:p.P775R,SIPA1L1:NM_001354289:exon11:c.C842G:p.P281R,SIPA1L1:NM_001354286:exon12:c.C2324G:p.P775R,SIPA1L1:NM_001284245:exon13:c.C2324G:p.P775R,SIPA1L1:NM_015556:exon13:c.C2324G:p.P775R	.	1.13	.	.	.		Fam116_f_m_aM_aF	0/1;0/0;0/1;0/0

chr14	72536225	72536225	C	T	RGS6	exonic	nonsynonymous SNV	RGS6:NM_001370286:exon14:c.C1108T:p.R370C,RGS6:NM_001370294:exon14:c.C1081T:p.R361C,RGS6:NM_001204419:exon15:c.C1207T:p.R403C,RGS6:NM_001204420:exon15:c.C1207T:p.R403C,RGS6:NM_001204421:exon15:c.C1207T:p.R403C,RGS6:NM_001204422:exon15:c.C1207T:p.R403C,RGS6:NM_001204423:exon15:c.C1213T:p.R405C,RGS6:NM_001370280:exon15:c.C1192T:p.R398C,RGS6:NM_001370283:exon15:c.C1207T:p.R403C,RGS6:NM_001370292:exon15:c.C1207T:p.R403C,RGS6:NM_001370293:exon15:c.C1207T:p.R403C,RGS6:NM_001204416:exon16:c.C1318T:p.R440C,RGS6:NM_001204417:exon16:c.C1318T:p.R440C,RGS6:NM_001204418:exon16:c.C1318T:p.R440C,RGS6:NM_001204424:exon16:c.C1318T:p.R440C,RGS6:NM_001370270:exon16:c.C1318T:p.R440C,RGS6:NM_001370271:exon16:c.C1318T:p.R440C,RGS6:NM_001370272:exon16:c.C1318T:p.R440C,RGS6:NM_001370273:exon16:c.C1318T:p.R440C,RGS6:NM_001370274:exon16:c.C1318T:p.R440C,RGS6:NM_001370275:exon16:c.C1318T:p.R440C,RGS6:NM_001370276:exon16:c.C1318T:p.R440C,RGS6:NM_001370277:exon16:c.C1318T:p.R440C,RGS6:NM_001370278:exon16:c.C1318T:p.R440C,RGS6:NM_001370279:exon16:c.C1309T:p.R437C,RGS6:NM_001370281:exon16:c.C1318T:p.R440C,RGS6:NM_001370282:exon16:c.C1318T:p.R440C,RGS6:NM_001370284:exon16:c.C1318T:p.R440C,RGS6:NM_001370287:exon16:c.C1318T:p.R440C,RGS6:NM_001370288:exon16:c.C1318T:p.R440C,RGS6:NM_001370289:exon16:c.C1318T:p.R440C,RGS6:NM_001370290:exon16:c.C1318T:p.R440C,RGS6:NM_001370291:exon16:c.C1318T:p.R440C,RGS6:NM_004296:exon16:c.C1318T:p.R440C	.	1.349	.	3.28E-05	.	Fam75_f_m_aM	0/1;0/0;0/1
chr14	72753327	72753327	G	A	DPF3	exonic	nonsynonymous SNV	DPF3:NM_001280542:exon3:c.C238T:p.R80C,DPF3:NM_001280544:exon3:c.C403T:p.R135C,DPF3:NM_012074:exon3:c.C238T:p.R80C,DPF3:NM_001280543:exon4:c.C268T:p.R90C	.	1.374	0.0006	9.01E-05	0.0001	Fam15_f_m_aM_aM	0/1;0/0;0/1;0/1
chr14	73103376	73103376	G	A	RBM25	exonic	nonsynonymous SNV	RBM25:NM_021239:exon10:c.G1052A:p.R351H	.	1.292	.	3.36E-05	.	Fam60_f_m_aF	0/0;0/0;0/1
chr14	73287209	73287209	G	A	NUMB	exonic	nonsynonymous SNV	NUMB:NM_001005745:exon8:c.C523T:p.R175W,NUMB:NM_003744:exon8:c.C523T:p.R175W,NUMB:NM_001005743:exon9:c.C556T:p.R186W,NUMB:NM_001005744:exon9:c.C556T:p.R186W,NUMB:NM_001320114:exon9:c.C556T:p.R186W	.	1.847	.	7.45E-05	.	Fam115_f_m_aF_aM_aF	0/1;0/0;0/0;0/1;0/0
chr14	73738627	73738627	G	T	ELMSAN1	exonic	nonsynonymous SNV	ELMSAN1:NM_001043318:exon2:c.C1382A:p.S461Y,ELMSAN1:NM_001367710:exon2:c.C1382A:p.S461Y,ELMSAN1:NM_194278:exon2:c.C1382A:p.S461Y	.	1.371	.	.	.	Fam86_f_m_aF	0/0;0/1;0/1
chr14	73739146	73739146	A	C	ELMSAN1	exonic	nonsynonymous SNV	ELMSAN1:NM_001043318:exon2:c.T863G:p.F288C,ELMSAN1:NM_001367710:exon2:c.T863G:p.F288C,ELMSAN1:NM_194278:exon2:c.T863G:p.F288C	.	1.364	0.0002	0.0008	0.0001	Fam120_f_m_aM_aM	0/1;0/0;0/1;0/1
chr14	74494896	74494896	G	T	ISCA2	exonic	nonsynonymous SNV	ISCA2:NM_194279:exon4:c.G361T:p.V121L	.	1.153	.	0.0001	9.77E-05	Fam69_f_m_aM	0/0;0/1;0/1
chr14	74674069	74674069	G	A	AREL1	exonic	nonsynonymous SNV	AREL1:NM_001039479:exon9:c.C1123T:p.R375C	.	1.11	.	9.86E-05	8.38E-05	Fam92_f_m_aM_raM	0/0;0/1;0/1;0/0
chr14	74817234	74817234	G	T	YLPM1	exonic	nonsynonymous SNV	YLPM1:NM_019589:exon15:c.G5903T:p.G1968V	.	1.555	.	7.24E-05	2.09E-05	Fam91_m_aM_dM_aM_dM	0/0;0/1;0/1;0/1;0/0
chr14	76776177	76776177	G	C	VASH1	exonic	nonsynonymous SNV	VASH1:NM_014909:exon5:c.G816C:p.Q272H	.	1.463	.	.	6.98E-06	Fam26_f_m_aM_uM	0/0;0/1;0/1;0/1
chr14	77133828	77133828	C	T	ZDHHC22	exonic	nonsynonymous SNV	ZDHHC22:NM_001364172:exon3:c.G647A:p.R216H,ZDHHC22:NM_174976:exon3:c.G647A:p.R216H	.	1.122	.	.	.	Fam33_f_m_aM_uM	0/0;0/1;0/1;0/1
chr14	77248804	77248804	C	T	TMEM63C	exonic	nonsynonymous SNV	TMEM63C:NM_020431:exon20:c.C1802T:p.A601V	.	1.231	0.0002	6.46E-05	6.28E-05	Fam112_f_m_aM	0/0;0/1;0/1
chr14	78709371	78709371	G	C	NRXN3	exonic	nonsynonymous SNV	NRXN3:NM_004796:exon4:c.G257C:p.S86T,NRXN3:NM_001330195:exon7:c.G1376C:p.S459T,NRXN3:NM_001366425:exon7:c.G1376C:p.S459T,NRXN3:NM_001366426:exon8:c.G1388C:p.S463T	.	1.111	.	1.12E-05	6.98E-06	Fam85_f_m_aM_aM	0/0;0/1;0/1;0/1
chr14	78714917	78714917	G	A	NRXN3	exonic	nonsynonymous SNV	NRXN3:NM_004796:exon5:c.G703A:p.V235M,NRXN3:NM_001330195:exon8:c.G1822A:p.V608M,NRXN3:NM_001366425:exon8:c.G1822A:p.V608M,NRXN3:NM_001366426:exon9:c.G1834A:p.V612M	.	1.687	.	.	.	Fam87_f_m_aM_uM	0/1;0/0;0/1;0/1
chr14	78957260	78957260	T	C	NRXN3	exonic	nonsynonymous SNV	NRXN3:NM_004796:exon8:c.T1175C:p.L392S,NRXN3:NM_001330195:exon11:c.T2294C:p.L765S,NRXN3:NM_001366425:exon11:c.T2294C:p.L765S,NRXN3:NM_001366426:exon12:c.T2306C:p.L769S	.	1.959	.	.	6.98E-06	Fam31_f_m_aM_uF	0/1;0/0;0/1;0/1
chr14	78957262	78957262	T	C	NRXN3	exonic	nonsynonymous SNV	NRXN3:NM_004796:exon8:c.T1177C:p.Y393H,NRXN3:NM_001330195:exon11:c.T2296C:p.Y766H,NRXN3:NM_001366425:exon11:c.T2296C:p.Y766H,NRXN3:NM_001366426:exon12:c.T2308C:p.Y770H	.	1.898	.	.	.	Fam28_f_m_aF_uF	0/0;0/1;0/1;0/0
chr14	78966073	78966073	C	T	NRXN3	exonic	nonsynonymous SNV	NRXN3:NM_004796:exon9:c.C1325T:p.T442M,NRXN3:NM_001330195:exon12:c.C2444T:p.T815M,NRXN3:NM_001366425:exon12:c.C2444T:p.T815M,NRXN3:NM_001366426:exon13:c.C2456T:p.T819M	.	1.697	.	0.0002	2.79E-05	Fam121_f_m_aF_aM	0/0;0/1;0/1;0/1
chr14	78967269	78967269	C	T	NRXN3	exonic	nonsynonymous SNV	NRXN3:NM_004796:exon10:c.C1720T:p.R574C,NRXN3:NM_001330195:exon13:c.C2839T:p.R947C,NRXN3:NM_001366425:exon13:c.C2839T:p.R947C,NRXN3:NM_001366426:exon14:c.C2851T:p.R951C	.	1.942	.	6.56E-05	2.10E-05	Fam106_f_m_aM	0/1;0/0;0/1
chr14	81487891	81487891	C	T	SEL1L	exonic	nonsynonymous SNV	SEL1L:NM_005065:exon15:c.G1447A:p.V483M	.	1.256	7.34E-05	6.17E-05	6.98E-06	Fam96_f_m_aM_uF	0/0;0/1;0/1;0/0
chr14	88517219	88517219	G	A	PTPN21	exonic	nonsynonymous SNV	PTPN21:NM_007039:exon3:c.C223T:p.R75C	.	1.135	0.0003	0.0004	0.0001	Fam118_f_m_aM_aF_uM	0/1;0/0;0/1;0/1;0/0

chr14	91234368	91234368	C	T	GPR68	exonic	nonsynonymous SNV	GPR68:NM_001177676:exon2:c.G683A:p.R228Q,GPR68:NM_003485:exon2:c.G683A:p.R228Q,GPR68:NM_001348437:exon3:c.G683A:p.R228Q	.	1.079	0.0001	0.0001	8.37E-05	Fam72_f_m_aF_uF	0/1;0/0;0/1;0/0
chr14	91475932	91475932	G	A	PPP4R3A	exonic	nonsynonymous SNV	PPP4R3A:NM_001284281:exon5:c.C428T:p.T143I,PPP4R3A:NM_001284280:exon7:c.C1145T:p.T382I,PPP4R3A:NM_001366432:exon7:c.C1145T:p.T382I	.	1.716	.	1.14E-05	2.10E-05	Fam68_f_m_aF_uF_uM	0/0;0/1;0/1;0/1;0/0
chr14	91937063	91937063	-	CC	FBLN5	exonic	frameshift insertion	FBLN5:NM_006329:exon4:c.262_263insGG:p.Y88Wfs*81	0.581	.	.	.	.	Fam89_f_m_aM	0/0;0/1;0/1
chr14	91937064	91937064	A	C	FBLN5	exonic	nonsynonymous SNV	FBLN5:NM_006329:exon4:c.T262G:p.Y88D	.	1.245	.	.	.	Fam89_f_m_aM	0/0;0/1;0/1
chr14	92993993	92993993	T	C	ITPK1	exonic	nonsynonymous SNV	ITPK1:NM_001142593:exon5:c.A251G:p.Y84C,ITPK1:NM_001142594:exon5:c.A251G:p.Y84C,ITPK1:NM_014216:exon5:c.A251G:p.Y84C	.	1.458	.	.	.	Fam122_f_m_aM	0/0;0/1;0/1
chr14	93571980	93571980	T	A	UNC79	exonic	nonsynonymous SNV	UNC79:NM_001346218:exon15:c.T1842A:p.N614K,UNC79:NM_020818:exon15:c.T1311A:p.N437K	.	1.152	.	.	.	Fam113_f_m_aF_raM_uF	0/1;0/0;0/1;0/0;0/1
chr14	93939434	93939434	G	T	ASB2	exonic	nonsynonymous SNV	ASB2:NM_016150:exon6:c.C1147A:p.L383M,ASB2:NM_001202429:exon8:c.C1291A:p.L431M	.	2.181	.	.	1.40E-05	Fam78_f_m_aF_uM	0/0;0/1;0/1;0/1
chr14	99398996	99398996	G	A	SETD3	exonic	nonsynonymous SNV	SETD3:NM_032233:exon13:c.C1468T:p.R490C	.	1.108	7.34E-05	0.0006	0.0001	Fam54_f_m_aM_uF	0/0;0/1;0/1;0/1
chr14	99399086	99399086	G	A	SETD3	exonic	nonsynonymous SNV	SETD3:NM_032233:exon13:c.C1378T:p.R460C	.	1.339	0.0001	9.11E-05	4.89E-05	Fam62_f_m_aM_aM	0/1;0/0;0/1;0/0
chr14	100123554	100123554	G	A	EVL	exonic	nonsynonymous SNV	EVL:NM_001330221:exon4:c.G368A:p.R123H,EVL:NM_016337:exon4:c.G374A:p.R125H	.	1.324	.	5.59E-05	4.19E-05	Fam40_f_m_aM_aM	0/0;0/1;0/1;0/1
chr14	100123554	100123554	G	A	EVL	exonic	nonsynonymous SNV	EVL:NM_001330221:exon4:c.G368A:p.R123H,EVL:NM_016337:exon4:c.G374A:p.R125H	.	1.324	.	5.59E-05	4.19E-05	Fam64_f_m_aM	0/1;0/0;0/1
chr14	100538718	100538718	C	G	BEGAIN	exonic	nonsynonymous SNV	BEGAIN:NM_001159531:exon6:c.G1033C:p.G345R,BEGAIN:NM_020836:exon7:c.G1033C:p.G345R	.	1.101	.	.	.	Fam56_f_m_aF_aM	0/0;0/1;0/1;0/0
chr14	100538723	100538723	T	C	BEGAIN	exonic	nonsynonymous SNV	BEGAIN:NM_001159531:exon6:c.A1028G:p.Y343C,BEGAIN:NM_020836:exon7:c.A1028G:p.Y343C	.	1.346	.	4.48E-05	1.40E-05	Fam36_f_m_aM_uM	0/0;0/1;0/1;0/1
chr14	100883646	100883646	C	A	RTL1	exonic	nonsynonymous SNV	RTL1:NM_001134888:exon1:c.G1143T:p.W381C	.	1.847	.	9.70E-05	9.79E-05	Fam4_f_m_aM	0/1;0/0;0/1
chr14	100884710	100884710	C	T	RTL1	exonic	nonsynonymous SNV	RTL1:NM_001134888:exon1:c.G79A:p.E27K	.	1.674	7.35E-05	9.85E-05	9.08E-05	Fam91_m_aM_dM_aM_dM	0/0;0/1;0/1;0/0;0/0
chr14	101988838	101988838	G	C	DYNC1H1	exonic	nonsynonymous SNV	DYNC1H1:NM_001376:exon10:c.G2854C:p.E952Q	.	1.181	.	.	.	Fam122_f_m_aM	0/0;0/1;0/1
chr14	102016376	102016376	T	C	DYNC1H1	exonic	nonsynonymous SNV	DYNC1H1:NM_001376:exon37:c.T7501C:p.S2501P	.	2.521	.	.	.	Fam122_f_m_aM	0/0;0/0;0/1
chr14	102026607	102026607	G	A	DYNC1H1	exonic	nonsynonymous SNV	DYNC1H1:NM_001376:exon44:c.G8671A:p.D2891N	.	1.739	.	.	.	Fam97_f_m_aM_aF	0/1;0/0;0/1;0/1
chr14	102042720	102042720	G	T	DYNC1H1	exonic	nonsynonymous SNV	DYNC1H1:NM_001376:exon69:c.G12485T:p.S4162I	.	1.415	.	1.12E-05	.	Fam119_f_m_aM_aM	0/0;0/1;0/0;0/1
chr14	102438034	102438034	T	C	TECPR2	exonic	nonsynonymous SNV	TECPR2:NM_001172631:exon10:c.T2407C:p.W803R,TECPR2:NM_014844:exon10:c.T2407C:p.W803R	.	1.394	.	1.12E-05	.	Fam77_f_m_aM_aF_uM_uFs	0/1;0/0;0/1;0/1;0/1;0/0
chr14	102450629	102450629	C	A	TECPR2	exonic	nonsynonymous SNV	TECPR2:NM_001172631:exon15:c.C3386A:p.S1129Y,TECPR2:NM_014844:exon15:c.C3386A:p.S1129Y	.	1.064	0.0001	0.0006	0.0003	Fam115_f_m_aF_aM_aF	0/0;0/1;0/0;0/1;0/1
chr14	102507549	102507549	C	A	ANKRD9	exonic	nonsynonymous SNV	ANKRD9:NM_001348652:exon3:c.G341T:p.G114V,ANKRD9:NM_001348651:exon4:c.G341T:p.G114V,ANKRD9:NM_152326:exon4:c.G341T:p.G114V	.	1.822	0.0003	.	6.37E-05	Fam2_f_m_aF	0/1;0/0;0/1
chr14	102945689	102945689	G	A	CDC42BPB	exonic	nonsynonymous SNV	CDC42BPB:NM_006035:exon29:c.C3784T:p.L1262F	.	1.335	.	0.0003	0.0001	Fam39_f_m_aM	0/1;0/0;0/1
chr14	102972016	102972016	C	T	CDC42BPB	exonic	nonsynonymous SNV	CDC42BPB:NM_006035:exon13:c.G1787A:p.R596Q	.	1.374	7.35E-05	3.28E-05	2.09E-05	Fam108_f_m_aM	0/1;0/0;0/1
chr14	102983706	102983706	G	C	CDC42BPB	exonic	nonsynonymous SNV	CDC42BPB:NM_006035:exon7:c.C741G:p.I247M	.	2.492	.	.	.	Fam92_f_m_aM_raM	0/0;0/0;0/0;0/1
chr14	103521318	103521318	G	A	CKB	exonic	nonsynonymous SNV	CKB:NM_001362531:exon4:c.C670T:p.P224S,CKB:NM_001823:exon5:c.C598T:p.P200S	.	1.474	7.35E-05	.	6.98E-06	Fam13_f_m_aM	0/1;0/0;0/1
chr14	103740147	103740147	C	A	PPP1R13B	exonic	nonsynonymous SNV	PPP1R13B:NM_015316:exon12:c.G2269T:p.D757Y	.	1.305	.	.	.	Fam82_f_m_aM_uF	0/0;0/1;0/1;0/0
chr14	103749910	103749910	G	C	PPP1R13B	exonic	nonsynonymous SNV	PPP1R13B:NM_015316:exon8:c.C853G:p.Q285E	.	1.224	.	8.99E-05	4.19E-05	Fam68_f_m_aF_uF_uM	0/1;0/0;0/1;0/1;0/1
chr14	105529754	105529754	C	T	TMEM121	exonic	nonsynonymous SNV	TMEM121:NM_001331238:exon2:c.C920T:p.P307L,TMEM121:NM_025268:exon2:c.C920T:p.P307L	.	1.219	.	0.0001	5.58E-05	Fam115_f_m_aF_aM_aF	0/1;0/0;0/0;0/0;0/1
chr15	23686343	23686343	G	C	NDN	exonic	nonsynonymous SNV	NDN:NM_002487:exon1:c.C875G:p.S292C	.	1.509	.	.	.	Fam62_f_m_aM_aM	0/0;0/1;0/1;0/0
chr15	24976412	24976412	A	G	SNRPN	exonic	nonsynonymous SNV	SNRPN:NM_001349464:exon6:c.A263G:p.K88R,SNRPN:NM_003097:exon6:c.A263G:p.K88R,SNRPN:NM_001349463:exon7:c.A263G:p.K88R,SNRPN:NM_001349465:exon7:c.A263G:p.K88R,SNRPN:NM_001349459:exon8:c.A263G:p.K88R,SNRPN:NM_022805:exon8:c.A263G:p.K88R,SNRPN:NM_022806:exon8:c.A263G:p.K88R,SNRPN:NM_022808:exon8:c.A263G:p.K88R,SNRPN:NM_001349455:exon9:c.A263G:p.K88R,SNRPN:NM_001349456:exon9:c.A263G:p.K88R,SNRPN:NM_001349458:exon9:c.A263G:p.K88R,SNRPN:NM_001349461:exon9:c.A263G:p.K88R,SNRPN:NM_001349462:exon9:c.A263G:p.K88R,SNRPN:NM_022807:exon9:c.A263G:p.K88R,SNRPN:NM_001349454:exon10:c.A263G:p.K88R,SNRPN:NM_001349457:exon10:c.A263G:p.K88R,SNRPN:NM_001349460:exon11:c.A263G:p.K88R	.	1.091	.	.	.	Fam40_f_m_aM_aM	0/0;0/1;0/1;0/0
chr15	25863014	25863014	C	T	ATP10A	exonic	nonsynonymous SNV	ATP10A:NM_024490:exon1:c.G83A:p.R28H	.	1.651	.	.	.	Fam12_f_m_aM_uM_aM	0/1;0/0;0/1;0/1;0/0
chr15	28113577	28113577	G	A	HERC2	exonic	nonsynonymous SNV	HERC2:NM_004667:exon91:c.C14015T:p.T4672M	.	1.759	7.34E-05	0.0003	0.0001	Fam101_f_m_aM	0/0;0/1;0/1
chr15	28141791	28141791	T	C	HERC2	exonic	nonsynonymous SNV	HERC2:NM_004667:exon77:c.A11756G:p.H3919R	.	1.957	.	.	6.98E-06	Fam9_f_m_aM_dM_uF	0/1;0/0;0/1;0/0;0/1
chr15	28142369	28142369	G	C	HERC2	exonic	nonsynonymous SNV	HERC2:NM_004667:exon76:c.C11569G:p.L3857V	.	1.735	.	.	.	Fam22_f_m_aF_aF_uF	0/1;0/0;0/1;0/1;0/1
chr15	28144801	28144801	C	A	HERC2	exonic	nonsynonymous SNV	HERC2:NM_004667:exon72:c.G11012T:p.R3671L	.	2.008	.	.	.	Fam123_f_aF	0/0;0/1
chr15	28238731	28238731	C	A	HERC2	exonic	nonsynonymous SNV	HERC2:NM_004667:exon24:c.G3619T:p.V1207L	.	1.165	.	2.24E-05	.	Fam24_f_m_aM_aM	0/0;0/1;0/1;0/0
chr15	29268926	29268926	C	G	NSMCE3	exonic	nonsynonymous SNV	NSMCE3:NM_138704:exon1:c.G780C:p.K260N	.	1.399	.	.	.	Fam76_f_m_aM_uM	0/0;0/1;0/1;0/1
chr15	30633569	30633569	C	T	ARHGAP11B	exonic	nonsynonymous SNV	ARHGAP11B:NM_001039841:exon3:c.C280T:p.R94C	.	1.58	.	5.60E-05	6.99E-05	Fam44_f_m_aM_uF	0/0;0/1;0/1;0/0

chr15	32616250	32616250	G	T	ARHGAP11A;ARH	exonic	nonsynonymous SNV	ARHGAP11A-SCG5:NM_001368319:exon1:c.G39T:p.Q13H,ARHGAP11A:NM_014783:exon1:c.G39T:p.Q13H,ARHGAP11A:NM_199357:exon1:c.G39T:p.Q13H	.	2.284	.	0.0001	4.19E-05		Fam11_f_m_aM_uM	0/1;0/0;0/1;0/0
chr15	34103574	34103574	C	A	PGBD4	exonic	nonsynonymous SNV	PGBD4:NM_152595:exon1:c.C1043A:p.T348N	.	1.311	.	7.82E-05	2.10E-05		Fam48_f_m_aM_uM	0/0;0/1;0/1;0/1
chr15	34242206	34242206	C	T	SLC12A6	exonic	nonsynonymous SNV	SLC12A6:NM_001042497:exon15:c.G2013A:p.M671I,SLC12A6:NM_005135:exon16:c.G1905A:p.M635I,SLC12A6:NM_133647:exon16:c.G2058A:p.M686I,SLC12A6:NM_001042494:exon17:c.G1881A:p.M627I,SLC12A6:NM_001042495:exon17:c.G1881A:p.M627I,SLC12A6:NM_001042496:exon17:c.G2031A:p.M677I,SLC12A6:NM_001365088:exon17:c.G2058A:p.M686I	.	1.143	.	.	.		Fam108_f_m_aM	0/0;0/1;0/1
chr15	34365142	34365142	C	T	LPCAT4	exonic	nonsynonymous SNV	LPCAT4:NM_153613:exon3:c.G344A:p.R115Q	.	1.799	0.0006	0.0001	6.98E-06		Fam25_f_m_aM	0/1;0/0;0/1
chr15	38299531	38299531	G	A	SPRED1	exonic	nonsynonymous SNV	SPRED1:NM_152594:exon2:c.G191A:p.R64Q	.	1.189	.	.	.		Fam32_f_m_aM_uM	0/0;0/1;0/1;0/0
chr15	38507809	38507809	A	G	RASGRP1	exonic	nonsynonymous SNV	RASGRP1:NM_001128602:exon9:c.T1159C:p.Y387H,RASGRP1:NM_001306086:exon9:c.T1159C:p.Y387H,RASGRP1:NM_005739:exon9:c.T1159C:p.Y387H	.	1.146	7.35E-05	0.0002	8.38E-05		Fam123_f_aF	0/0;0/1
chr15	39592745	39592745	C	T	THBS1	exonic	nonsynonymous SNV	THBS1:NM_003246:exon17:c.C2710T:p.P904S	.	1.303	.	.	.		Fam42_f_m_aM_uF	0/0;0/1;0/1;0/0
chr15	40367062	40367062	G	A	DISP2	exonic	nonsynonymous SNV	DISP2:NM_033510:exon8:c.G950A:p.R317H	.	1.334	0.0003	0.0002	0.0001		Fam32_f_m_aM_uM	0/1;0/0;0/1;0/0
chr15	40369548	40369548	C	T	DISP2	exonic	nonsynonymous SNV	DISP2:NM_033510:exon8:c.C3436T:p.R1146C	.	1.305	.	0.0003	1.40E-05		Fam22_f_m_aF_aF_uF	0/1;0/0;0/1;0/1;0/0
chr15	40472009	40472009	T	C	CHST14	exonic	nonsynonymous SNV	CHST14:NM_130468:exon1:c.T796C:p.Y266H	.	1.765	0.0002	7.46E-05	8.37E-05		Fam19_f_m_aM	0/0;0/1;0/1
chr15	40931610	40931610	C	T	DLL4	exonic	nonsynonymous SNV	DLL4:NM_019074:exon4:c.C502T:p.R168C	.	2.555	.	.	.		Fam36_f_m_aM_uM	0/0;0/1;0/1;0/0
chr15	40936645	40936645	G	A	DLL4	exonic	nonsynonymous SNV	DLL4:NM_019074:exon9:c.G1658A:p.R553Q	.	1.138	0.0001	9.48E-05	7.68E-05		Fam113_f_m_aF_raM_uF	0/0;0/1;0/1;0/0;0/1
chr15	41573715	41573715	C	T	TYRO3	exonic	nonsynonymous SNV	TYRO3:NM_001330264:exon18:c.C2047T:p.R683C,TYRO3:NM_006293:exon18:c.C2182T:p.R728C	.	1.019	.	4.47E-05	6.98E-05		Fam24_f_m_aM_aM	0/1;0/0;0/1;0/1
chr15	41900716	41900716	C	T	EHD4	exonic	nonsynonymous SNV	EHD4:NM_139265:exon6:c.G1555A:p.E519K	.	1.071	.	3.28E-05	.		Fam19_f_m_aM	0/0;0/1;0/1
chr15	43420659	43420659	G	A	TP53BP1	exonic	nonsynonymous SNV	TP53BP1:NM_001141979:exon21:c.C4327T:p.R1443C,TP53BP1:NM_001141980:exon21:c.C4327T:p.R1443C,TP53BP1:NM_005657:exon21:c.C4312T:p.R1438C,TP53BP1:NM_001355001:exon22:c.C1447T:p.R483C	.	1.999	.	.	.		Fam15_f_m_aM_aM	0/0;0/1;0/1;0/0
chr15	43421047	43421047	G	A	TP53BP1	exonic	nonsynonymous SNV	TP53BP1:NM_001141979:exon20:c.C4228T:p.P1410S,TP53BP1:NM_001141980:exon20:c.C4228T:p.P1410S,TP53BP1:NM_005657:exon20:c.C4213T:p.P1405S,TP53BP1:NM_001355001:exon21:c.C1348T:p.P450S	.	1.72	.	3.30E-05	.		Fam110_f_m_aM_aM_uMs	0/0;0/1;0/1;0/1;0/0
chr15	43793080	43793080	A	G	SERF2	exonic	nonsynonymous SNV	SERF2:NM_001018108:exon2:c.A113G:p.Q38R,SERF2:NM_001199875:exon2:c.A113G:p.Q38R,SERF2:NM_001199876:exon2:c.A113G:p.Q38R,SERF2:NM_001199878:exon2:c.A71G:p.Q24R,SERF2:NM_001199877:exon4:c.A113G:p.Q38R	.	1.687	0.0001	.	6.98E-06		Fam66_f_m_aM	0/1;0/0;0/1
chr15	43905874	43905874	C	T	FRMD5	exonic	nonsynonymous SNV	FRMD5:NM_001322951:exon5:c.G400A:p.E134K,FRMD5:NM_001322949:exon6:c.G505A:p.E169K,FRMD5:NM_001322950:exon6:c.G505A:p.E169K,FRMD5:NM_032892:exon6:c.G505A:p.E169K,FRMD5:NM_001286490:exon7:c.G238A:p.E80K	.	2.065	0.0006	0.0008	0.0005		Fam77_f_m_aM_aF	0/1;0/0;0/0;0/1
chr15	44403030	44403030	G	A	CASC4	exonic	nonsynonymous SNV	CASC4:NM_138423:exon9:c.G1216A:p.D406N	.	1.261	.	.	.		Fam95_f_m_aM_aM_uF	0/0;0/1;0/1;0/1;0/1
chr15	45160849	45160849	G	A	DUOX1	exonic	nonsynonymous SNV	DUOX1:NM_175940:exon29:c.G3715A:p.G1239S,DUOX1:NM_017434:exon30:c.G3715A:p.G1239S	.	1.013	7.35E-05	.	6.98E-06		Fam75_f_m_aM	0/0;0/1;0/1
chr15	47763853	47763853	G	A	SEMA6D	exonic	nonsynonymous SNV	SEMA6D:NM_001358351:exon10:c.G751A:p.V251M,SEMA6D:NM_001358352:exon10:c.G751A:p.V251M,SEMA6D:NM_020858:exon10:c.G751A:p.V251M,SEMA6D:NM_024966:exon10:c.G751A:p.V251M,SEMA6D:NM_153616:exon10:c.G751A:p.V251M,SEMA6D:NM_153617:exon10:c.G751A:p.V251M,SEMA6D:NM_153618:exon10:c.G751A:p.V251M,SEMA6D:NM_153619:exon10:c.G751A:p.V251M,SEMA6D:NM_001198999:exon13:c.G751A:p.V251M	.	1.127	0.0006	0.0007	0.0004		Fam4_f_m_aM	0/0;0/1;0/1
chr15	47764025	47764025	G	A	SEMA6D	exonic	nonsynonymous SNV	SEMA6D:NM_001358351:exon10:c.G923A:p.G308D,SEMA6D:NM_001358352:exon10:c.G923A:p.G308D,SEMA6D:NM_020858:exon10:c.G923A:p.G308D,SEMA6D:NM_024966:exon10:c.G923A:p.G308D,SEMA6D:NM_153616:exon10:c.G923A:p.G308D,SEMA6D:NM_153617:exon10:c.G923A:p.G308D,SEMA6D:NM_153618:exon10:c.G923A:p.G308D,SEMA6D:NM_153619:exon10:c.G923A:p.G308D,SEMA6D:NM_001198999:exon13:c.G923A:p.G308D	.	1.913	.	1.12E-05	.		Fam78_f_m_aF_uM	0/0;0/1;0/1;0/0
chr15	48427606	48427606	G	A	FBN1	exonic	nonsynonymous SNV	FBN1:NM_000138:exon58:c.C7165T:p.L2389F	.	1.39	.	.	.		Fam72_f_m_aF_uF	0/0;0/1;0/1;0/0
chr15	48515404	48515404	A	G	FBN1	exonic	nonsynonymous SNV	FBN1:NM_000138:exon12:c.T1451C:p.L484P	.	1.632	.	.	.		Fam25_f_m_aM	0/1;0/0;0/1
chr15	50958794	50958794	-	GTAA	AP4E1	exonic	frameshift insertion	AP4E1:NM_001252127:exon14:c.1626_1627insGTAA:p.D544Sfs*14,AP4E1:NM_007347:exon14:c.1851_1852insGTAA:p.D619Sfs*14	0.577	.	0.0001	0.0008	4.89E-05		Fam10_f_m_aM_uF	0/0;0/1;0/1;0/0
chr15	52112705	52112705	G	A	BCL2L10	exonic	nonsynonymous SNV	BCL2L10:NM_001306168:exon1:c.C22T:p.R8C,BCL2L10:NM_020396:exon1:c.C22T:p.R8C	.	1.278	.	0.0006	0.0002		Fam27_f_m_aF_uM_uM	0/1;0/0;0/1;0/0;0/0
chr15	52416277	52416277	G	C	MYO5A	exonic	nonsynonymous SNV	MYO5A:NM_000259:exon5:c.C480G:p.I160M,MYO5A:NM_001142495:exon5:c.C480G:p.I160M	.	1.132	.	.	.		Fam14_f_m_aM_aM	0/1;0/0;0/1;0/0
chr15	52433186	52433186	C	T	MYO5A	exonic	nonsynonymous SNV	MYO5A:NM_000259:exon2:c.G127A:p.E43K,MYO5A:NM_001142495:exon2:c.G127A:p.E43K	.	1.078	.	1.12E-05	2.10E-05		Fam44_f_m_aM_uF	0/1;0/0;0/1;0/1
chr15	52789080	52789080	G	C	ONECUT1	exonic	nonsynonymous SNV	ONECUT1:NM_004498:exon1:c.C805G:p.R269G	.	1.709	0.0006	0.0009	0.0006		Fam67_f_m_aM_uF	0/0;0/1;0/1;0/1

chr15	58014245	58014245	C	T	ALDH1A2	exonic	nonsynonymous SNV	ALDH1A2:NM_003888:exon2:c.G154A:p.G52R,ALDH1A2:NM_170696:exon2:c.G154A:p.G52R,ALDH1A2:NM_001206897:exon3:c.G91A:p.G31R	.	1.284	.	.	.		Fam23_f_m_aM_dF_uFs	0/1;0/0;0/1;0/0;0/0
chr15	58611843	58611843	C	G	ADAM10	exonic	nonsynonymous SNV	ADAM10:NM_001320570:exon11:c.G1567C:p.D523H,ADAM10:NM_001110:exon12:c.G1660C:p.D554H	.	1.365	.	.	.		Fam109_f_m_aM	0/1;0/0;0/1
chr15	58887385	58887385	C	T	SLTM	exonic	nonsynonymous SNV	SLTM:NM_001013843:exon18:c.G2477A:p.R826Q,SLTM:NM_024755:exon18:c.G2531A:p.R844Q	.	1.197	0.0001	0.0002	9.79E-05		Fam14_f_m_aM_aM	0/1;0/0;0/0;0/1
chr15	63061245	63061245	T	G	TPM1	exonic	nonsynonymous SNV	TPM1:NM_001330344:exon5:c.T503G:p.L168W,TPM1:NM_001330351:exon5:c.T503G:p.L168W,TPM1:NM_001365781:exon5:c.T503G:p.L168W,TPM1:NM_000366:exon6:c.T611G:p.L204W,TPM1:NM_001018006:exon6:c.T611G:p.L204W,TPM1:NM_001018020:exon6:c.T611G:p.L204W	.	3.119	.	.	.		Fam10_f_m_aM_uF	0/0;0/1;0/1;0/0
chr15	63249686	63249686	C	T	RAB8B	exonic	nonsynonymous SNV	RAB8B:NM_016530:exon3:c.C227T:p.A76V	.	2.081	.	.	1.40E-05		Fam85_f_m_aM_aM	0/0;0/0;0/1;0/0
chr15	63654211	63654211	G	A	HERC1	exonic	nonsynonymous SNV	HERC1:NM_003922:exon51:c.C10198T:p.R3400C	.	1.278	0.0001	4.50E-05	2.09E-05		Fam88_f_m_aF	0/1;0/0;0/1
chr15	63672581	63672581	T	C	HERC1	exonic	nonsynonymous SNV	HERC1:NM_003922:exon39:c.A7960G:p.T2654A	.	1.064	.	3.43E-05	.		Fam97_f_m_aM_aF	0/1;0/0;0/0;0/1
chr15	63680710	63680710	G	C	HERC1	exonic	nonsynonymous SNV	HERC1:NM_003922:exon35:c.C6292G:p.H2098D	.	1.227	.	.	.		Fam79_f_m_aM_uM	0/1;0/0;0/1;0/0
chr15	63758210	63758210	G	A	HERC1	exonic	nonsynonymous SNV	HERC1:NM_003922:exon4:c.C1186T:p.P396S	.	1.732	.	.	.		Fam35_f_m_aF_uM	0/1;0/0;0/1;0/0
chr15	64156789	64156789	G	A	PPIB	exonic	nonsynonymous SNV	PPIB:NM_000942:exon4:c.C464T:p.T155M	.	1.068	.	6.17E-05	1.40E-05		Fam64_f_m_aM	0/0;0/1;0/1
chr15	64162872	64162872	C	A	PPIB	exonic	nonsynonymous SNV	PPIB:NM_000942:exon1:c.G115T:p.G39W	.	1.131	.	2.32E-05	.		Fam2_f_m_aF	0/0;0/1;0/1
chr15	65055203	65055203	C	T	RASL12	exonic	nonsynonymous SNV	RASL12:NM_001307930:exon4:c.G440A:p.C147Y,RASL12:NM_016563:exon5:c.G497A:p.C166Y	.	1.078	.	3.31E-05	2.09E-05		Fam24_f_m_aM_aM	0/1;0/0;0/1;0/1
chr15	65077356	65077356	G	T	KBTBD13	exonic	nonsynonymous SNV	KBTBD13:NM_001101362:exon1:c.G541T:p.D181Y	.	1.64	.	0.0001	.		Fam97_f_m_aM_aF	0/1;0/0;0/0;0/1
chr15	65395923	65395923	A	C	IGDCC4	exonic	nonsynonymous SNV	IGDCC4:NM_020962:exon7:c.T1238G:p.M413R	.	2.092	.	.	.		Fam9_f_m_aM_dM_uF	0/0;0/1;0/1;0/1;0/0
chr15	68208360	68208360	A	G	CLN6	exonic	nonsynonymous SNV	CLN6:NM_017882:exon7:c.T716C:p.F239S	.	1.385	.	.	.		Fam15_f_m_aM_aM	0/0;0/1;0/1;0/0
chr15	68218598	68218598	C	T	CLN6	exonic	nonsynonymous SNV	CLN6:NM_017882:exon2:c.G136A:p.D46N	.	1.009	7.35E-05	6.53E-05	3.49E-05		Fam117_f_m_aM_aF	0/1;0/0;0/0;0/1
chr15	68710778	68710778	C	T	CORO2B	exonic	nonsynonymous SNV	CORO2B:NM_001190456:exon4:c.C365T:p.T122M,CORO2B:NM_001190457:exon4:c.C365T:p.T122M,CORO2B:NM_001324015:exon4:c.C365T:p.T122M,CORO2B:NM_006091:exon4:c.C380T:p.T127M,CORO2B:NM_001324014:exon5:c.C365T:p.T122M	.	1.117	7.34E-05	0.0001	4.19E-05		Fam15_f_m_aM_aM	0/0;0/1;0/0;0/1
chr15	72046188	72046188	G	A	MYO9A	exonic	nonsynonymous SNV	MYO9A:NM_006901:exon2:c.C376T:p.R126W	.	1.143	0.0001	0.0003	0.0001		Fam74_f_m_aM_uM	0/0;0/1;0/1;0/0
chr15	73984005	73984005	G	A	STOML1	exonic	nonsynonymous SNV	STOML1:NM_001256673:exon6:c.C979T:p.R327W,STOML1:NM_001256674:exon6:c.C976T:p.R326W,STOML1:NM_001256675:exon6:c.C916T:p.R306W,STOML1:NM_001324226:exon6:c.C658T:p.R220W,STOML1:NM_001324229:exon6:c.C661T:p.R221W,STOML1:NM_001256672:exon7:c.C1126T:p.R376W,STOML1:NM_001256676:exon7:c.C865T:p.R289W,STOML1:NM_001324227:exon7:c.C868T:p.R290W,STOML1:NM_001324228:exon7:c.C868T:p.R290W,STOML1:NM_004809:exon7:c.C1129T:p.R377W,STOML1:NM_001256677:exon8:c.C1000T:p.R334W,STOML1:NM_001324230:exon8:c.C1003T:p.R335W	.	1.105	0.0006	7.46E-05	2.79E-05		Fam83_f_m_aF	0/1;0/0;0/1
chr15	74410761	74410761	G	A	SEMA7A	exonic	nonsynonymous SNV	SEMA7A:NM_001146029:exon13:c.C1822T:p.R608C,SEMA7A:NM_001146030:exon14:c.C1369T:p.R457C,SEMA7A:NM_003612:exon14:c.C1864T:p.R622C	.	1.447	0.0006	0.0004	0.0005		Fam94_f_m_aM	0/0;0/1;0/1
chr15	75381678	75381678	C	T	SIN3A	exonic	nonsynonymous SNV	SIN3A:NM_001145357:exon18:c.G3223A:p.V1075I,SIN3A:NM_001145358:exon18:c.G3223A:p.V1075I,SIN3A:NM_015477:exon18:c.G3223A:p.V1075I	.	1	.	1.12E-05	.		Fam15_f_m_aM_aM	0/0;0/1;0/1;0/0
chr15	75639903	75639903	A	C	IMP3	exonic	nonsynonymous SNV	IMP3:NM_018285:exon1:c.T266G:p.V89G	.	1.089	.	.	.		Fam94_f_m_aM	0/0;0/1;0/1
chr15	75657046	75657046	G	A	SNX33	exonic	nonsynonymous SNV	SNX33:NM_153271:exon2:c.G1556A:p.R519H,SNX33:NM_001318146:exon3:c.G1334A:p.R445H	.	1.445	.	3.28E-05	.		Fam51_f_m_aM_uF	0/0;0/1;0/1;0/1
chr15	75675693	75675693	G	A	CSPG4	exonic	nonsynonymous SNV	CSPG4:NM_001897:exon10:c.C6826T:p.R2276C	.	1.282	.	0.0005	2.09E-05		Fam47_f_m_aM	0/0;0/1;0/1
chr15	76665774	76665774	T	C	SCAPER	exonic	nonsynonymous SNV	SCAPER:NM_001145923:exon21:c.A1786G:p.K596E,SCAPER:NM_020843:exon21:c.A2524G:p.K842E,SCAPER:NM_001353009:exon22:c.A2542G:p.K848E,SCAPER:NM_001353010:exon22:c.A2122G:p.K708E,SCAPER:NM_001353011:exon22:c.A2140G:p.K714E,SCAPER:NM_001353012:exon22:c.A2122G:p.K708E	.	1.177	.	.	.		Fam56_f_m_aF_aM	0/0;0/1;0/0;0/1
chr15	78999766	78999766	C	T	RASGRF1	exonic	nonsynonymous SNV	RASGRF1:NM_153815:exon4:c.G419A:p.R140Q,RASGRF1:NM_001145648:exon17:c.G2723A:p.R908Q,RASGRF1:NM_002891:exon18:c.G2771A:p.R924Q	.	1.104	.	3.28E-05	6.98E-06		Fam95_f_m_aM_aM_uF	0/1;0/0;0/1;0/1;0/0
chr15	79970918	79970918	T	C	BCL2A1	exonic	nonsynonymous SNV	BCL2A1:NM_001114735:exon1:c.A202G:p.R68G,BCL2A1:NM_004049:exon1:c.A202G:p.R68G	.	1.344	.	.	.		Fam48_f_m_aM_uM	0/0;0/1;0/1;0/1
chr15	80404543	80404543	C	T	ARNT2	exonic	nonsynonymous SNV	ARNT2:NM_014862:exon1:c.C28T:p.P10S	.	1.63	7.93E-05	0.0004	8.60E-05		Fam109_f_m_aM	0/0;0/1;0/1
chr15	80925650	80925650	C	T	CEMIP	exonic	nonsynonymous SNV	CEMIP:NM_018689:exon18:c.C2315T:p.P772L,CEMIP:NM_001293298:exon19:c.C2315T:p.P772L,CEMIP:NM_001293304:exon19:c.C2315T:p.P772L	.	1.148	0.0001	0.0003	0.0002		Fam36_f_m_aM_uM	0/0;0/1;0/1;0/1
chr15	80937826	80937826	C	A	CEMIP	exonic	nonsynonymous SNV	CEMIP:NM_018689:exon24:c.C3254A:p.P1085Q,CEMIP:NM_001293298:exon25:c.C3254A:p.P1085Q,CEMIP:NM_001293304:exon25:c.C3254A:p.P1085Q	.	1.117	.	1.12E-05	.		Fam118_f_m_aM_aF_uM	0/1;0/0;0/1;0/1;0/0
chr15	81003077	81003077	A	C	TLNRD1	exonic	nonsynonymous SNV	TLNRD1:NM_022566:exon1:c.A806C:p.Q269P	.	1.062	.	.	.		Fam56_f_m_aF_aM	0/1;0/0;0/0;0/1
chr15	82666816	82666816	G	A	AP3B2	exonic	nonsynonymous SNV	AP3B2:NM_001278511:exon14:c.C1687T:p.L563F,AP3B2:NM_001278512:exon15:c.C1783T:p.L595F,AP3B2:NM_004644:exon15:c.C1783T:p.L595F	.	1.38	7.35E-05	5.63E-05	1.40E-05		Fam16_f_m_aM_aM	0/0;0/1;0/1;0/0

chr15	83572657	83572657	C	T	SH3GL3	exonic	nonsynonymous SNV	SH3GL3:NM_001301108:exon5:c.C217T:p.P73S,SH3GL3:NM_001324182:exon5:c.C424T:p.P142S,SH3GL3:NM_001324187:exon5:c.C217T:p.P73S,SH3GL3:NM_003027:exon5:c.C424T:p.P142S,SH3GL3:NM_001324183:exon6:c.C448T:p.P150S,SH3GL3:NM_001324185:exon6:c.C217T:p.P73S,SH3GL3:NM_001324186:exon6:c.C319T:p.P107S,SH3GL3:NM_001324184:exon7:c.C319T:p.P107S,SH3GL3:NM_001301109:exon8:c.C448T:p.P150S	.	1.271	.	.	.		Fam78_f_m_aF_uM	0/1;0/0;0/1;0/0
chr15	83576723	83576723	T	A	SH3GL3	exonic	nonsynonymous SNV	SH3GL3:NM_001301108:exon6:c.T399A:p.F133L,SH3GL3:NM_001324182:exon6:c.T606A:p.F202L,SH3GL3:NM_001324187:exon6:c.T399A:p.F133L,SH3GL3:NM_003027:exon6:c.T606A:p.F202L,SH3GL3:NM_001324183:exon7:c.T630A:p.F210L,SH3GL3:NM_001324185:exon7:c.T399A:p.F133L,SH3GL3:NM_001324186:exon7:c.T501A:p.F167L,SH3GL3:NM_001324184:exon8:c.T501A:p.F167L,SH3GL3:NM_001301109:exon9:c.T630A:p.F210L	.	1.365	.	.	.		Fam26_f_m_aM_uM	0/0;0/1;0/1;0/1
chr15	87877077	87877077	C	T	NTRK3	exonic	nonsynonymous SNV	NTRK3:NM_001243101:exon18:c.G2312A:p.R771Q,NTRK3:NM_002530:exon19:c.G2336A:p.R779Q,NTRK3:NM_001012338:exon20:c.G2378A:p.R793Q	.	1.417	0.0003	0.0007	6.98E-05		Fam116_f_m_aM_aF	0/1;0/0;0/1;0/0
chr15	88032953	88032953	C	T	NTRK3	exonic	nonsynonymous SNV	NTRK3:NM_001320134:exon12:c.G1489A:p.G497R,NTRK3:NM_001320135:exon12:c.G1195A:p.G399R,NTRK3:NM_001243101:exon13:c.G1465A:p.G489R,NTRK3:NM_001007156:exon14:c.G1489A:p.G497R,NTRK3:NM_001012338:exon14:c.G1489A:p.G497R,NTRK3:NM_002530:exon14:c.G1489A:p.G497R	.	1.237	0.0003	0.0001	8.41E-05		Fam110_f_m_aM_aM_uMs	0/1;0/0;0/1;0/1;0/0
chr15	89104046	89104046	T	A	ABHD2	splicing	.	.	0.571	.	0.0006	.	2.79E-05		Fam95_f_m_aM_aM_uF	0/0;0/1;0/1;0/1;0/1
chr15	90087189	90087189	A	-	IDH2	exonic	frameshift deletion	IDH2:NM_001290114:exon5:c.500delT:p.V167Afs*33,IDH2:NM_001289910:exon7:c.734delT:p.V245Afs*33,IDH2:NM_002168:exon7:c.890delT:p.V297Afs*33	0.502	.	.	.	.		Fam47_f_m_aM	0/0;0/1;0/1
chr15	90088364	90088364	C	T	IDH2	exonic	nonsynonymous SNV	IDH2:NM_001290114:exon3:c.G283A:p.D95N,IDH2:NM_001289910:exon5:c.G517A:p.D173N,IDH2:NM_002168:exon5:c.G673A:p.D225N	.	1.418	0.0004	0.0004	0.0003		Fam99_f_m_aM_aM	0/1;0/0;0/0;0/1
chr15	90476810	90476810	T	G	IQGAP1	exonic	nonsynonymous SNV	IQGAP1:NM_003870:exon24:c.T2932G:p.L978V	.	1.786	.	.	6.98E-06		Fam87_f_m_aM_uM	0/0;0/1;0/1;0/1
chr15	90893134	90893134	C	T	FES	exonic	nonsynonymous SNV	FES:NM_001143783:exon13:c.C1687T:p.R563C,FES:NM_001143784:exon13:c.C1651T:p.R551C,FES:NM_001143785:exon13:c.C1477T:p.R493C,FES:NM_002005:exon15:c.C1861T:p.R621C	.	1.182	.	3.27E-05	.		Fam71_f_m_aF	0/1;0/0;0/1
chr15	90905279	90905279	T	C	MAN2A2	exonic	nonsynonymous SNV	MAN2A2:NM_006122:exon3:c.T161C:p.I54T,MAN2A2:NM_001320977:exon4:c.T161C:p.I54T	.	1.07	.	3.28E-05	6.98E-06		Fam71_f_m_aF	0/1;0/0;0/1
chr15	90957272	90957272	C	T	RCCD1	exonic	nonsynonymous SNV	RCCD1:NM_001017919:exon3:c.C326T:p.P109L,RCCD1:NM_033544:exon4:c.C326T:p.P109L	.	1.925	.	.	.		Fam17_f_m_aM_uM	0/0;0/1;0/1;0/1
chr15	91916183	91916183	C	G	SLCO3A1	exonic	nonsynonymous SNV	SLCO3A1:NM_001145044:exon2:c.C371G:p.A124G,SLCO3A1:NM_013272:exon2:c.C371G:p.A124G	.	2.419	.	.	.		Fam95_f_m_aM_aM_uF	0/1;0/0;0/1;0/1;0/0
chr15	92984426	92984426	G	C	CHD2	exonic	nonsynonymous SNV	CHD2:NM_001271:exon25:c.G3163C:p.V1055L	.	1.3	.	1.12E-05	.		Fam65_f_m_aM_uF_dF	0/1;0/0;0/1;0/0;0/0
chr15	92985627	92985627	G	T	CHD2	exonic	nonsynonymous SNV	CHD2:NM_001271:exon26:c.G3367T:p.V1123L	.	1.339	.	.	6.98E-06		Fam110_f_m_aM_aM_uMs	0/0;0/1;0/1;0/1;0/0
chr15	98916752	98916752	G	A	IGF1R	exonic	nonsynonymous SNV	IGF1R:NM_000875:exon10:c.G2077A:p.G693S,IGF1R:NM_001291858:exon10:c.G2077A:p.G693S	.	1.39	7.34E-05	0.0002	6.28E-05		Fam87_f_m_aM_uM	0/1;0/0;0/1;0/0
chr16	58672	58672	G	A	RHBDF1	exonic	nonsynonymous SNV	RHBDF1:NM_022450:exon18:c.C2236T:p.R746C	.	1.337	.	0.0002	6.98E-06		Fam115_f_m_aF_aM_aF	0/1;0/0;0/1;0/1;0/1
chr16	426164	426164	C	T	RAB11FIP3	exonic	nonsynonymous SNV	RAB11FIP3:NM_001370401:exon1:c.C158T:p.P53L,RAB11FIP3:NM_014700:exon1:c.C158T:p.P53L	.	1.038	.	.	.		Fam72_f_m_aF_uF	0/1;0/0;0/1;0/1
chr16	676999	676999	G	A	RHBDL1	exonic	nonsynonymous SNV	RHBDL1:NM_001278721:exon3:c.G230A:p.R77H,RHBDL1:NM_001318733:exon3:c.G650A:p.R217H,RHBDL1:NM_001278720:exon4:c.G455A:p.R152H	.	1.42	.	7.91E-05	2.79E-05		Fam114_f_m_aM	0/0;0/1;0/1
chr16	685982	685982	A	G	WDR24	exonic	nonsynonymous SNV	WDR24:NM_032259:exon5:c.T1460C:p.L487P	.	1.464	.	9.80E-05	6.98E-06		Fam22_f_m_aF_aF_uF	0/1;0/0;0/1;0/1;0/1
chr16	689238	689238	C	G	WDR24	exonic	nonsynonymous SNV	WDR24:NM_032259:exon1:c.G403C:p.V135L	.	1.175	.	.	.		Fam112_f_m_aM	0/1;0/0;0/1
chr16	1413848	1413848	G	C	UNKL	exonic	nonsynonymous SNV	UNKL:NM_001037125:exon2:c.C285G:p.D95E,UNKL:NM_001193388:exon2:c.C285G:p.D95E	.	1.262	.	.	.		Fam51_f_m_aM_uF	0/0;0/1;0/1;0/1
chr16	1669109	1669109	A	G	CRAMP1	exonic	nonsynonymous SNV	CRAMP1:NM_020825:exon18:c.A3443G:p.H1148R	.	1.037	.	1.16E-05	.		Fam9_f_m_aM_dM_uF	0/1;0/0;0/1;0/0;0/1
chr16	1766766	1766766	C	T	MAPK8IP3	exonic	nonsynonymous SNV	MAPK8IP3:NM_001040439:exon23:c.C2962T:p.H988Y,MAPK8IP3:NM_001318852:exon24:c.C2983T:p.H995Y,MAPK8IP3:NM_015133:exon24:c.C2980T:p.H994Y	.	1.206	7.35E-05	1.14E-05	6.98E-06		Fam108_f_m_aM	0/1;0/0;0/1
chr16	1767211	1767211	C	T	MAPK8IP3	exonic	nonsynonymous SNV	MAPK8IP3:NM_001040439:exon25:c.C3130T:p.R1044C,MAPK8IP3:NM_001318852:exon26:c.C3151T:p.R1051C,MAPK8IP3:NM_015133:exon26:c.C3148T:p.R1050C	.	2.034	.	.	.		Fam2_f_m_aF	0/1;0/0;0/1
chr16	1767223	1767223	G	A	MAPK8IP3	exonic	nonsynonymous SNV	MAPK8IP3:NM_001040439:exon25:c.G3142A:p.V1048I,MAPK8IP3:NM_001318852:exon26:c.G3163A:p.V1055I,MAPK8IP3:NM_015133:exon26:c.G3160A:p.V1054I	.	1.245	.	.	.		Fam7_f_m_aM_aM_uM	0/1;0/0;0/0;0/1;0/1
chr16	2171598	2171598	G	C	TRAF7	exonic	nonsynonymous SNV	TRAF7:NM_032271:exon7:c.G468C:p.L156F	.	1.243	0.0001	0.0005	6.28E-05		Fam70_f_m_aM	0/0;0/1;0/1
chr16	2206544	2206544	C	T	MLST8	exonic	nonsynonymous SNV	MLST8:NM_001352059:exon3:c.C31T:p.P11S,MLST8:NM_001352060:exon3:c.C31T:p.P11S,MLST8:NM_001199173:exon4:c.C229T:p.P77S,MLST8:NM_001199174:exon4:c.C229T:p.P77S,MLST8:NM_001199175:exon4:c.C226T:p.P76S,MLST8:NM_001352057:exon4:c.C247T:p.P83S,MLST8:NM_022372:exon4:c.C229T:p.P77S	.	1.448	.	1.12E-05	6.98E-06		Fam48_f_m_aM_uM	0/1;0/0;0/1;0/1
chr16	2233094	2233094	C	G	E4F1	exonic	nonsynonymous SNV	E4F1:NM_001288776:exon7:c.C967G:p.L323V,E4F1:NM_001288778:exon7:c.C967G:p.L323V,E4F1:NM_004424:exon7:c.C967G:p.L323V	.	1.101	.	2.24E-05	1.40E-05		Fam56_f_m_aF_aM	0/1;0/0;0/1;0/1
chr16	2472326	2472326	C	T	NTN3	exonic	nonsynonymous SNV	NTN3:NM_006181:exon1:c.C625T:p.P209S	.	1.017	.	1.16E-05	6.98E-06		Fam91_m_aM_dM_aM_dM	0/1;0/1;0/1;0/0;0/1

chr16	2967053	2967053	C	T	KREMEN2	exonic	nonsynonymous SNV	KREMEN2:NM_001253725:exon6:c.C667T:p.L223F,KREMEN2:NM_001253726:exon6:c.C667T:p.L223F,KREMEN2:NM_024507:exon6:c.C784T:p.L262F,KREMEN2:NM_172229:exon6:c.C784T:p.L262F	.	1.431	.	7.21E-05	.		Fam86_f_m_aF	0/1;0/0;0/1
chr16	2967059	2967059	G	C	KREMEN2	exonic	nonsynonymous SNV	KREMEN2:NM_001253725:exon6:c.G673C:p.E225Q,KREMEN2:NM_001253726:exon6:c.G673C:p.E225Q,KREMEN2:NM_024507:exon6:c.G790C:p.E264Q,KREMEN2:NM_172229:exon6:c.G790C:p.E264Q	.	1.532	.	.	.	Fam60_f_m_aF	0/1;0/0;0/1
chr16	3021791	3021791	G	A	TNFRSF12A	exonic	nonsynonymous SNV	TNFRSF12A:NM_016639:exon4:c.G355A:p.G119R	.	1.365	.	0.0003	2.79E-05	Fam28_f_m_aF_uF	0/1;0/0;0/1;0/0
chr16	3140979	3140979	A	G	ZNF213	exonic	nonsynonymous SNV	ZNF213:NM_001134655:exon6:c.A1012G:p.T338A,ZNF213:NM_004220:exon6:c.A1012G:p.T338A	.	1.007	.	.	6.98E-06	Fam51_f_m_aM_uF	0/1;0/0;0/1;0/0
chr16	4694095	4694095	C	G	NUDT16L1	exonic	nonsynonymous SNV	NUDT16L1:NM_001193452:exon2:c.C271G:p.L91V,NUDT16L1:NM_001370585:exon2:c.C265G:p.L89V,NUDT16L1:NM_001370586:exon2:c.C265G:p.L89V,NUDT16L1:NM_001370587:exon2:c.C271G:p.L91V,NUDT16L1:NM_032349:exon2:c.C271G:p.L91V	.	1.529	.	.	.	Fam82_f_m_aM_uF	0/1;0/0;0/1;0/0
chr16	4695104	4695104	G	C	NUDT16L1	exonic	nonsynonymous SNV	NUDT16L1:NM_001370588:exon2:c.G300C:p.K100N,NUDT16L1:NM_001370585:exon3:c.G555C:p.K185N,NUDT16L1:NM_001370587:exon3:c.G441C:p.K147N,NUDT16L1:NM_032349:exon3:c.G561C:p.K187N	.	1.31	.	0.0001	4.19E-05	Fam91_m_aM_dM_aM_dM	0/1;0/1;0/1;0/0;0/1
chr16	10941798	10941798	G	T	DEXI	exonic	nonsynonymous SNV	DEXI:NM_014015:exon1:c.C208A:p.L70I	.	1.824	7.35E-05	.	1.40E-05	Fam86_f_m_aF	0/1;0/0;0/1
chr16	11886558	11886558	T	C	GSPT1	exonic	nonsynonymous SNV	GSPT1:NM_001130006:exon9:c.A1163G:p.N388S,GSPT1:NM_001130007:exon9:c.A752G:p.N251S,GSPT1:NM_002094:exon9:c.A1166G:p.N389S	.	1.087	.	0.0002	3.49E-05	Fam84_f_m_aF	0/1;0/0;0/1
chr16	15612561	15612561	A	G	MARF1	exonic	nonsynonymous SNV	MARF1:NM_001184998:exon17:c.T3470C:p.L1157S,MARF1:NM_001184999:exon17:c.T3461C:p.L1154S,MARF1:NM_014647:exon17:c.T3470C:p.L1157S	.	1.682	.	3.28E-05	.	Fam35_f_m_aF_uM	0/0;0/1;0/1;0/0
chr16	15741818	15741818	T	A	MYH11	exonic	nonsynonymous SNV	MYH11:NM_002474:exon21:c.A2594T:p.K865M,MYH11:NM_022844:exon21:c.A2594T:p.K865M,MYH11:NM_001040113:exon22:c.A2615T:p.K872M,MYH11:NM_001040114:exon22:c.A2615T:p.K872M	.	1.81	.	.	.	Fam5_f_m_aM	0/0;0/1;0/1
chr16	16114878	16114878	T	G	ABCC1	exonic	nonsynonymous SNV	ABCC1:NM_004996:exon23:c.T3192G:p.F1064L	.	1.188	.	.	6.98E-06	Fam46_f_m_aM_uM	0/0;0/1;0/1;0/1
chr16	16136619	16136619	T	A	ABCC1	exonic	nonsynonymous SNV	ABCC1:NM_004996:exon29:c.T4267A:p.C1423S	.	1.22	0.0009	0.0005	0.0003	Fam114_f_m_aM	0/1;0/0;0/1
chr16	18795529	18795529	T	G	ARL6IP1	exonic	nonsynonymous SNV	ARL6IP1:NM_001313858:exon4:c.A256C:p.T86P,ARL6IP1:NM_015161:exon4:c.A343C:p.T115P	.	1.554	.	.	.	Fam17_f_m_aM_uM	0/1;0/0;0/1;0/1
chr16	19627783	19627783	G	A	VPS35L	exonic	nonsynonymous SNV	VPS35L:NM_001300743:exon14:c.G1160A:p.C387Y,VPS35L:NM_001365294:exon14:c.G1160A:p.C387Y,VPS35L:NM_001365293:exon16:c.G1361A:p.C454Y,VPS35L:NM_020314:exon16:c.G1361A:p.C454Y,VPS35L:NM_001365295:exon17:c.G473A:p.C158Y	.	1.012	.	1.12E-05	1.40E-05	Fam40_f_m_aM_aM	0/0;0/1;0/0;0/1
chr16	19872287	19872287	G	A	GPRC5B	exonic	nonsynonymous SNV	GPRC5B:NM_001304771:exon2:c.C952T:p.R318C,GPRC5B:NM_016235:exon2:c.C559T:p.R187C	.	1.465	.	.	.	Fam68_f_m_aF_uF_uM	0/0;0/1;0/1;0/0;0/1
chr16	22313637	22313637	C	T	POLR3E	exonic	nonsynonymous SNV	POLR3E:NM_001258034:exon6:c.C274T:p.P92S,POLR3E:NM_001258035:exon6:c.C274T:p.P92S,POLR3E:NM_001258033:exon7:c.C382T:p.P128S,POLR3E:NM_001258036:exon7:c.C382T:p.P128S,POLR3E:NM_018119:exon7:c.C382T:p.P128S	.	1.209	.	3.28E-05	6.98E-06	Fam36_f_m_aM_uM	0/0;0/1;0/1;0/0
chr16	22915429	22915429	G	A	HS3ST2	exonic	nonsynonymous SNV	HS3ST2:NM_006043:exon2:c.G971A:p.R324Q	.	2.192	.	.	7.00E-06	Fam1_f_m_aM	0/0;0/1;0/1
chr16	23406239	23406239	T	C	COG7	exonic	nonsynonymous SNV	COG7:NM_153603:exon12:c.A1499G:p.Y500C	.	1.052	7.34E-05	0.0007	0.0002	Fam56_f_m_aF_aM	0/0;0/1;0/0;0/1
chr16	23410314	23410314	C	T	COG7	exonic	nonsynonymous SNV	COG7:NM_153603:exon11:c.G1456A:p.E486K	.	1	0.0006	.	2.09E-05	Fam9_f_m_aM_dM_uF	0/0;0/1;0/1;0/1;0/0
chr16	24092883	24092883	A	G	PRKCB	exonic	nonsynonymous SNV	PRKCB:NM_002738:exon6:c.A622G:p.S208G,PRKCB:NM_212535:exon6:c.A622G:p.S208G	.	1.01	.	.	.	Fam13_f_m_aM	0/1;0/0;0/1
chr16	24113032	24113032	G	A	PRKCB	exonic	nonsynonymous SNV	PRKCB:NM_002738:exon8:c.G881A:p.G294E,PRKCB:NM_212535:exon8:c.G881A:p.G294E	.	1.365	0.0003	0.0006	0.0003	Fam71_f_m_aF	0/0;0/1;0/1
chr16	24113068	24113068	A	C	PRKCB	exonic	nonsynonymous SNV	PRKCB:NM_002738:exon8:c.A917C:p.E306A,PRKCB:NM_212535:exon8:c.A917C:p.E306A	.	1.325	.	.	.	Fam95_f_m_aM_aM_uF	0/1;0/0;0/1;0/1;0/0
chr16	24816854	24816854	A	G	TNRC6A	exonic	nonsynonymous SNV	TNRC6A:NM_001330520:exon19:c.A4723G:p.I1575V,TNRC6A:NM_001351850:exon19:c.A4750G:p.I1584V,TNRC6A:NM_014494:exon20:c.A4870G:p.I1624V	.	1.322	.	3.29E-05	6.98E-06	Fam117_f_m_aM_aF	0/1;0/0;0/1;0/1
chr16	24820246	24820246	C	G	TNRC6A	exonic	nonsynonymous SNV	TNRC6A:NM_001330520:exon21:c.C5041G:p.P1681A,TNRC6A:NM_001351850:exon21:c.C5068G:p.P1690A,TNRC6A:NM_014494:exon22:c.C5188G:p.P1730A	.	1.462	.	5.58E-05	2.10E-05	Fam107_f_m_aM	0/1;0/0;0/1
chr16	27493242	27493242	G	A	GTF3C1	exonic	nonsynonymous SNV	GTF3C1:NM_001286242:exon17:c.C2833T:p.R945C,GTF3C1:NM_001520:exon17:c.C2833T:p.R945C	.	1.267	.	9.83E-05	1.40E-05	Fam119_f_m_aM_aM	0/0;0/1;0/1;0/0
chr16	28956306	28956306	G	A	NFATC2IP	exonic	nonsynonymous SNV	NFATC2IP:NM_032815:exon5:c.G815A:p.R272Q	.	1.001	.	.	.	Fam36_f_m_aM_uM	0/0;0/1;0/1;0/1
chr16	28983930	28983930	C	T	SPNS1	exonic	nonsynonymous SNV	SPNS1:NM_001142449:exon9:c.C1243T:p.R415C,SPNS1:NM_001142451:exon10:c.C1309T:p.R437C,SPNS1:NM_032038:exon11:c.C1465T:p.R489C,SPNS1:NM_001142448:exon12:c.C1465T:p.R489C,SPNS1:NM_001142450:exon12:c.C1246T:p.R416C	.	1.556	.	7.60E-05	.	Fam53_f_m_aM	0/1;0/0;0/1
chr16	29695171	29695171	A	T	QPRT	exonic	nonsynonymous SNV	QPRT:NM_014298:exon2:c.A521T:p.H174L	.	1.588	.	0.0003	5.58E-05	Fam97_f_m_aM_aF	0/1;0/0;0/0;0/1
chr16	29695171	29695171	A	T	QPRT	exonic	nonsynonymous SNV	QPRT:NM_014298:exon2:c.A521T:p.H174L	.	1.588	.	0.0003	5.58E-05	Fam25_f_m_aM	0/1;0/0;0/1
chr16	29840203	29840203	C	T	MVP	exonic	nonsynonymous SNV	MVP:NM_001293204:exon7:c.C935T:p.P312L,MVP:NM_001293205:exon7:c.C935T:p.P312L,MVP:NM_005115:exon8:c.C935T:p.P312L,MVP:NM_017458:exon8:c.C935T:p.P312L	.	1.131	.	1.12E-05	.	Fam89_f_m_aM	0/1;0/0;0/1

chr16	29840254	29840254	A	C	MVP	exonic	nonsynonymous SNV	MVP:NM_001293204:exon7:c.A986C:p.Q329P,MVP:NM_001293205:exon7:c.A986C:p.Q329P,MVP:NM_005115:exon8:c.A986C:p.Q329P,MVP:NM_017458:exon8:c.A986C:p.Q329P	.	1.226	.	.	.	Fam79_f_m_aM_uM	0/0;0/1;0/1;0/1
chr16	29841656	29841656	G	A	MVP	exonic	nonsynonymous SNV	MVP:NM_001293204:exon8:c.G1252A:p.E418K,MVP:NM_001293205:exon8:c.G1252A:p.E418K,MVP:NM_005115:exon9:c.G1252A:p.E418K,MVP:NM_017458:exon9:c.G1252A:p.E418K	.	1.176	0.0002	0.0003	6.98E-05	Fam110_f_m_aM_aM_uMs	0/0;0/1;0/1;0/0;0/0
chr16	30009243	30009243	C	T	DOC2A	exonic	nonsynonymous SNV	DOC2A:NM_001282068:exon4:c.G376A:p.D126N,DOC2A:NM_003586:exon4:c.G376A:p.D126N,DOC2A:NM_001282062:exon5:c.G376A:p.D126N,DOC2A:NM_001282063:exon5:c.G376A:p.D126N	.	1.117	.	9.33E-05	.	Fam26_f_m_aM_uM	0/1;0/0;0/1;0/0
chr16	30370015	30370015	C	G	TBC1D10B	exonic	nonsynonymous SNV	TBC1D10B:NM_015527:exon1:c.G169C:p.E57Q	.	1.533	7.41E-05	.	3.49E-05	Fam110_f_m_aM_aM_uMs	0/0;0/1;0/0;0/1;0/0
chr16	30418102	30418102	C	T	ZNF771	exonic	nonsynonymous SNV	ZNF771:NM_001142305:exon3:c.C689T:p.P230L,ZNF771:NM_016643:exon3:c.C689T:p.P230L	.	3.171	.	.	.	Fam112_f_m_aM	0/1;0/0;0/1
chr16	30496190	30496190	G	A	ITGAL	exonic	nonsynonymous SNV	ITGAL:NM_001114380:exon12:c.G1348A:p.G450S,ITGAL:NM_002209:exon14:c.G1597A:p.G533S	.	1.014	.	0.0007	4.19E-05	Fam22_f_m_aF_aF_uF	0/0;0/1;0/1;0/1;0/1
chr16	30525322	30525322	C	A	ZNF768	exonic	nonsynonymous SNV	ZNF768:NM_024671:exon2:c.G818T:p.G273V	.	1.876	.	.	6.98E-06	Fam107_f_m_aM	0/0;0/1;0/1
chr16	30662847	30662847	T	A	FBRS	exonic	nonsynonymous SNV	FBRS:NM_001105079:exon6:c.T1043A:p.L348H	.	2.271	.	0.0001	6.28E-05	Fam91_m_aM_dM_aM_dM	0/0;0/1;0/0;0/0;0/1
chr16	30766503	30766503	G	A	RNF40	exonic	nonsynonymous SNV	RNF40:NM_001207033:exon10:c.G1238A:p.R413Q,RNF40:NM_001286572:exon10:c.G1238A:p.R413Q,RNF40:NM_014771:exon10:c.G1238A:p.R413Q	.	1.565	7.35E-05	7.51E-05	2.09E-05	Fam13_f_m_aM	0/0;0/1;0/1
chr16	31001112	31001112	C	A	STX1B	exonic	nonsynonymous SNV	STX1B:NM_052874:exon3:c.G187T:p.A63S	.	1.094	.	1.12E-05	6.98E-06	Fam29_f_m_aF_uM	0/1;0/0;0/1;0/0
chr16	31001159	31001159	G	A	STX1B	exonic	nonsynonymous SNV	STX1B:NM_052874:exon3:c.C140T:p.S47L	.	1.326	.	3.35E-05	6.98E-06	Fam108_f_m_aM	0/0;0/1;0/1
chr16	31061150	31061150	G	A	ZNF668	exonic	nonsynonymous SNV	ZNF668:NM_001172668:exon3:c.C1778T:p.T593I,ZNF668:NM_001172670:exon3:c.C1778T:p.T593I,ZNF668:NM_024706:exon3:c.C1778T:p.T593I,ZNF668:NM_001172669:exon4:c.C1847T:p.T616I	.	2.032	.	0.0003	3.49E-05	Fam23_f_m_aM_dF_uFs	0/1;0/0;0/1;0/0;0/0
chr16	31321615	31321615	C	T	ITGAM	exonic	nonsynonymous SNV	ITGAM:NM_000632:exon16:c.C1990T:p.R664W,ITGAM:NM_001145808:exon16:c.C1993T:p.R665W	.	1.009	.	0.0001	4.19E-05	Fam40_f_m_aM_aM	0/1;0/0;0/1;0/0
chr16	31466484	31466484	G	T	ARMC5	exonic	nonsynonymous SNV	ARMC5:NM_001105247:exon6:c.G2403T:p.W801C,ARMC5:NM_001301820:exon7:c.G2499T:p.W833C,ARMC5:NM_001288767:exon8:c.G2688T:p.W896C	.	1.79	0.0001	5.67E-05	3.49E-05	Fam39_f_m_aM	0/1;0/0;0/1
chr16	46909852	46909852	C	T	GPT2	exonic	nonsynonymous SNV	GPT2:NM_001142466:exon6:c.C445T:p.R149W,GPT2:NM_133443:exon6:c.C745T:p.R249W	.	1.266	0.0006	0.0003	0.0002	Fam75_f_m_aM	0/0;0/1;0/1
chr16	46918675	46918675	G	A	GPT2	exonic	nonsynonymous SNV	GPT2:NM_001142466:exon8:c.G655A:p.V219M,GPT2:NM_133443:exon8:c.G955A:p.V319M	.	1.281	0.0001	0.0002	0.0002	Fam113_f_m_aF_raM_uF	0/0;0/0;0/0;0/1;0/0
chr16	47161796	47161796	T	G	ITFG1	exonic	nonsynonymous SNV	ITFG1:NM_001305002:exon16:c.A1276C:p.N426H,ITFG1:NM_030790:exon16:c.A1615C:p.N539H	.	1.249	.	.	1.40E-05	Fam2_f_m_aF	0/1;0/0;0/1
chr16	47454023	47454023	A	C	ITFG1	exonic	nonsynonymous SNV	ITFG1:NM_001305002:exon3:c.T78G:p.N26K,ITFG1:NM_030790:exon3:c.T417G:p.N139K	.	1.296	.	0.0006	4.19E-05	Fam116_f_m_aM_aF	0/0;0/1;0/0;0/1
chr16	50304495	50304495	C	T	ADCY7	exonic	nonsynonymous SNV	ADCY7:NM_001114:exon11:c.C1504T:p.H502Y,ADCY7:NM_001286057:exon11:c.C1504T:p.H502Y	.	1.331	.	.	3.49E-05	Fam55_f_m_aM_aM_dM	0/0;0/1;0/0;0/1;0/0
chr16	50308352	50308352	G	A	ADCY7	exonic	nonsynonymous SNV	ADCY7:NM_001114:exon16:c.G1876A:p.G626R,ADCY7:NM_001286057:exon16:c.G1876A:p.G626R	.	1.468	0.0001	0.0001	2.79E-05	Fam46_f_m_aM_uM	0/0;0/1;0/1;0/1
chr16	54931226	54931226	C	T	IRX5	exonic	stopgain	IRX5:NM_001252197:exon1:c.C28T:p.Q10X,IRX5:NM_005853:exon1:c.C28T:p.Q10X	0.417	.	.	.	.	Fam102_f_m_aM	0/0;0/0;0/1
chr16	55485759	55485759	G	A	MMP2	exonic	nonsynonymous SNV	MMP2:NM_001127891:exon5:c.G664A:p.G222S,MMP2:NM_001302508:exon5:c.G586A:p.G196S,MMP2:NM_001302509:exon5:c.G586A:p.G196S,MMP2:NM_001302510:exon5:c.G586A:p.G196S,MMP2:NM_004530:exon5:c.G814A:p.G272S	.	1.014	.	.	.	Fam64_f_m_aM	0/0;0/1;0/1
chr16	55498306	55498306	T	C	MMP2	exonic	nonsynonymous SNV	MMP2:NM_001127891:exon11:c.T1477C:p.Y493H,MMP2:NM_001302508:exon11:c.T1399C:p.Y467H,MMP2:NM_001302509:exon11:c.T1399C:p.Y467H,MMP2:NM_001302510:exon11:c.T1399C:p.Y467H,MMP2:NM_004530:exon11:c.T1627C:p.Y543H	.	1.184	0.0002	0.0002	9.77E-05	Fam74_f_m_aM_uM	0/0;0/1;0/1;0/0
chr16	56834232	56834232	C	T	NUP93	exonic	nonsynonymous SNV	NUP93:NM_001242795:exon12:c.C1273T:p.L425F,NUP93:NM_001242796:exon12:c.C1273T:p.L425F,NUP93:NM_014669:exon14:c.C1642T:p.L548F	.	1.124	.	0.0001	5.58E-05	Fam66_f_m_aM	0/1;0/0;0/1
chr16	57216984	57216984	T	G	RSPRY1	exonic	nonsynonymous SNV	RSPRY1:NM_001305163:exon8:c.T850G:p.Y284D,RSPRY1:NM_001305164:exon8:c.T850G:p.Y284D,RSPRY1:NM_133368:exon8:c.T850G:p.Y284D	.	2.213	.	.	.	Fam65_f_m_aM_uF_dF	0/0;0/1;0/1;0/0;0/1
chr16	57470012	57470012	A	G	POLR2C	exonic	nonsynonymous SNV	POLR2C:NM_032940:exon7:c.A491G:p.Y164C	.	1.842	7.35E-05	0.0004	2.79E-05	Fam57_f_m_aM_uF	0/0;0/1;0/1;0/0
chr16	57470122	57470122	G	A	POLR2C	exonic	nonsynonymous SNV	POLR2C:NM_032940:exon7:c.G601A:p.E201K	.	1.331	.	1.12E-05	6.98E-06	Fam118_f_m_aM_aF_uM	0/0;0/0;0/1;0/1;0/0
chr16	58543534	58543537	AGAC	-	CNOT1	exonic	frameshift deletion	CNOT1:NM_206999:exon31:c.4504_4507del:p.V1502Ifs*18	0.064	.	0.0003	0.0001	6.99E-05	Fam21_f_m_aM_uM	0/1;0/0;0/1;0/0
chr16	58546700	58546700	G	C	CNOT1	exonic	nonsynonymous SNV	CNOT1:NM_001265612:exon28:c.C3785G:p.A1262G,CNOT1:NM_016284:exon28:c.C3800G:p.A1267G,CNOT1:NM_206999:exon28:c.C3800G:p.A1267G	.	1.257	.	3.28E-05	6.98E-06	Fam34_f_m_aM_uF	0/1;0/0;0/1;0/1
chr16	61653663	61653663	C	T	CDH8	exonic	nonsynonymous SNV	CDH8:NM_001796:exon12:c.G2345A:p.R782H	.	1.422	.	6.19E-05	2.79E-05	Fam40_f_m_aM_aM	0/0;0/1;0/1;0/1
chr16	66517140	66517140	G	A	TK2	exonic	nonsynonymous SNV	TK2:NM_001172644:exon7:c.C539T:p.P180L,TK2:NM_001172645:exon7:c.C560T:p.P187L,TK2:NM_001172643:exon8:c.C521T:p.P174L,TK2:NM_001272050:exon8:c.C323T:p.P108L,TK2:NM_004614:exon8:c.C614T:p.P205L,TK2:NM_001271934:exon9:c.C467T:p.P156L	.	1.118	.	6.17E-05	5.58E-05	Fam35_f_m_aF_uM	0/1;0/0;0/1;0/1
chr16	66922156	66922156	C	G	RRAD	exonic	nonsynonymous SNV	RRAD:NM_001128850:exon5:c.G847C:p.G283R,RRAD:NM_004165:exon5:c.G847C:p.G283R	.	1.799	.	0.0001	5.58E-05	Fam77_f_m_aM_aF_uM_uFs	0/0;0/1;0/0;0/1;0/1;0/0
chr16	66923600	66923600	G	A	RRAD	exonic	nonsynonymous SNV	RRAD:NM_001128850:exon4:c.C565T:p.R189C,RRAD:NM_004165:exon4:c.C565T:p.R189C	.	1.925	7.37E-05	3.27E-05	1.40E-05	Fam10_f_m_aM_uF	0/0;0/1;0/1;0/1

chr16	67149444	67149444	G	A	B3GNT9	exonic	nonsynonymous SNV	B3GNT9:NM_033309:exon2:c.C1042T:p.P348S	.	1.818	.	.	.		Fam35_f_m_aF_uM	0/0;0/1;0/1;0/0
chr16	67150026	67150026	A	C	B3GNT9	exonic	nonsynonymous SNV	B3GNT9:NM_033309:exon2:c.T460G:p.F154V	.	2.078	.	.	.		Fam118_f_m_aM_aF_uM	0/1;0/0;0/1;0/0;0/0
chr16	67163215	67163215	C	G	FBXL8	exonic	nonsynonymous SNV	FBXL8:NM_018378:exon3:c.C520G:p.L174V	.	1.279	.	.	.		Fam84_f_m_aF	0/1;0/0;0/1
chr16	67238250	67238250	G	A	FHOD1	exonic	nonsynonymous SNV	FHOD1:NM_001318202:exon5:c.C499T:p.R167C,FHOD1:NM_013241:exon5:c.C499T:p.R167C	.	1.665	7.35E-05	6.53E-05	2.79E-05		Fam71_f_m_aF	0/1;0/0;0/1
chr16	67249085	67249085	C	T	SLC9A5	exonic	nonsynonymous SNV	SLC9A5:NM_001323971:exon1:c.C71T:p.P24L,SLC9A5:NM_001323972:exon1:c.C71T:p.P24L,SLC9A5:NM_001323973:exon1:c.C71T:p.P24L,SLC9A5:NM_004594:exon1:c.C71T:p.P24L	.	1.491	.	.	.		Fam35_f_m_aF_uM	0/0;0/1;0/1;0/0
chr16	67438551	67438551	C	T	ATP6V0D1	exonic	nonsynonymous SNV	ATP6V0D1:NM_004691:exon8:c.G1033A:p.D345N	.	1.044	.	.	.		Fam118_f_m_aM_aF_uM	0/0;0/1;0/0;0/1;0/1
chr16	67540119	67540119	C	T	RIPOR1	exonic	nonsynonymous SNV	RIPOR1:NM_001193522:exon7:c.C493T:p.R165C,RIPOR1:NM_001193523:exon7:c.C541T:p.R181C,RIPOR1:NM_001193524:exon7:c.C523T:p.R175C,RIPOR1:NM_024519:exon7:c.C481T:p.R161C	.	1.767	7.35E-05	6.70E-05	0.0002		Fam82_f_m_aM_uF	0/0;0/1;0/1;0/1
chr16	67540192	67540192	G	A	RIPOR1	exonic	nonsynonymous SNV	RIPOR1:NM_001193522:exon7:c.G566A:p.R189H,RIPOR1:NM_001193523:exon7:c.G614A:p.R205H,RIPOR1:NM_001193524:exon7:c.G596A:p.R199H,RIPOR1:NM_024519:exon7:c.G554A:p.R185H	.	1.664	7.35E-05	0.0002	0.0002		Fam99_f_m_aM_aM	0/0;0/1;0/1;0/1
chr16	67628404	67628404	C	G	CTCF	exonic	nonsynonymous SNV	CTCF:NM_001191022:exon7:c.C569G:p.T190S,CTCF:NM_001363916:exon9:c.C1553G:p.T518S,CTCF:NM_006565:exon9:c.C1553G:p.T518S	.	1.903	.	.	.		Fam33_f_m_aM_uM	0/0;0/1;0/1;0/1
chr16	67629412	67629412	A	T	CTCF	exonic	nonsynonymous SNV	CTCF:NM_001191022:exon8:c.A732T:p.R244S,CTCF:NM_001363916:exon10:c.A1716T:p.R572S,CTCF:NM_006565:exon10:c.A1716T:p.R572S	.	3.211	.	.	.		Fam45_f_m_aM_uF	0/1;0/0;0/1;0/1
chr16	67649936	67649936	C	T	CARMIL2	exonic	nonsynonymous SNV	CARMIL2:NM_001013838:exon21:c.C2050T:p.R684C,CARMIL2:NM_001317026:exon22:c.C1942T:p.R648C	.	1.233	.	0.0003	3.49E-05		Fam98_f_m_aM	0/0;0/1;0/1
chr16	67668021	67668021	G	A	C16orf86	exonic	nonsynonymous SNV	C16orf86:NM_001012984:exon3:c.G476A:p.R159H	.	1.108	.	1.13E-05	6.98E-06		Fam91_m_aM_dM_aM_dM	0/0;0/1;0/1;0/0;0/1
chr16	67729990	67729990	G	A	RANBP10	exonic	nonsynonymous SNV	RANBP10:NM_001320238:exon7:c.C595T:p.R199C,RANBP10:NM_001320239:exon7:c.C778T:p.R260C,RANBP10:NM_020850:exon8:c.C946T:p.R316C,RANBP10:NM_001320240:exon11:c.C259T:p.R87C	.	1.228	.	9.80E-05	2.79E-05		Fam14_f_m_aM_aM	0/1;0/0;0/0;0/1
chr16	67806443	67806443	C	T	RANBP10	exonic	nonsynonymous SNV	RANBP10:NM_001320239:exon1:c.G94A:p.E32K,RANBP10:NM_020850:exon1:c.G94A:p.E32K	.	1.215	0.0001	0.0004	0.0003		Fam37_f_m_aF_uM	0/0;0/1;0/1;0/0
chr16	67806454	67806454	G	C	RANBP10	exonic	nonsynonymous SNV	RANBP10:NM_001320239:exon1:c.C83G:p.P28R,RANBP10:NM_020850:exon1:c.C83G:p.P28R	.	1.111	.	8.18E-05	6.28E-05		Fam69_f_m_aM	0/1;0/0;0/1
chr16	67945180	67945180	G	A	SLC12A4	exonic	nonsynonymous SNV	SLC12A4:NM_001145962:exon22:c.C3079T:p.L1027F,SLC12A4:NM_001145961:exon23:c.C3055T:p.L1019F,SLC12A4:NM_001145963:exon23:c.C3055T:p.L1019F,SLC12A4:NM_001145964:exon23:c.C2980T:p.L994F,SLC12A4:NM_005072:exon23:c.C3073T:p.L1025F	.	1.032	.	1.25E-05	2.10E-05		Fam40_f_m_aM_aM	0/0;0/1;0/1;0/1
chr16	67947331	67947331	C	T	SLC12A4	exonic	nonsynonymous SNV	SLC12A4:NM_001145962:exon15:c.G2078A:p.R693Q,SLC12A4:NM_001145961:exon16:c.G2072A:p.R691Q,SLC12A4:NM_001145963:exon16:c.G2054A:p.R685Q,SLC12A4:NM_001145964:exon16:c.G1979A:p.R660Q,SLC12A4:NM_005072:exon16:c.G2072A:p.R691Q	.	1.078	.	0.0001	3.49E-05		Fam64_f_m_aM	0/0;0/1;0/1
chr16	68255795	68255795	C	T	PLA2G15	exonic	nonsynonymous SNV	PLA2G15:NM_012320:exon5:c.C532T:p.R178C	.	1.331	.	0.0003	4.89E-05		Fam80_f_m_aM_uM	0/1;0/0;0/1;0/1
chr16	69109607	69109607	G	A	HAS3	exonic	nonsynonymous SNV	HAS3:NM_001199280:exon2:c.G212A:p.R71Q,HAS3:NM_005329:exon2:c.G212A:p.R71Q,HAS3:NM_138612:exon2:c.G212A:p.R71Q	.	1.168	0.0006	0.0005	0.0002		Fam75_f_m_aM	0/1;0/0;0/1
chr16	70251474	70251474	G	C	EXOSC6	exonic	nonsynonymous SNV	EXOSC6:NM_058219:exon1:c.C427G:p.Q143E	.	1.2	7.54E-05	.	1.41E-05		Fam28_f_m_aF_uF	0/0;0/1;0/1;0/0
chr16	70251621	70251621	C	A	EXOSC6	exonic	nonsynonymous SNV	EXOSC6:NM_058219:exon1:c.G280T:p.G94C	.	1.662	.	.	7.21E-06		Fam102_f_m_aM	0/0;0/1;0/1
chr16	70329381	70329381	C	A	DDX19B	exonic	nonsynonymous SNV	DDX19B:NM_001257175:exon5:c.C244A:p.P82T,DDX19B:NM_001014449:exon6:c.C370A:p.P124T,DDX19B:NM_001257174:exon6:c.C370A:p.P124T,DDX19B:NM_001014451:exon7:c.C604A:p.P202T,DDX19B:NM_001257172:exon7:c.C619A:p.P207T,DDX19B:NM_001257173:exon7:c.C370A:p.P124T,DDX19B:NM_001363938:exon8:c.C712A:p.P238T,DDX19B:NM_007242:exon8:c.C697A:p.P233T	.	1.051	7.37E-05	3.35E-05	4.19E-05		Fam60_f_m_aF	0/1;0/0;0/1
chr16	70544474	70544474	T	C	SF3B3	exonic	nonsynonymous SNV	SF3B3:NM_012426:exon10:c.T1270C:p.Y424H	.	2.487	.	.	.		Fam78_f_m_aF_uM	0/0;0/1;0/1;0/1
chr16	72107486	72107486	C	T	DHX38	exonic	nonsynonymous SNV	DHX38:NM_014003:exon20:c.C2747T:p.P916L	.	1.583	.	.	.		Fam65_f_m_aM_uF_dF	0/1;0/0;0/1;0/0;0/1
chr16	74774578	74774578	C	T	FA2H	exonic	nonsynonymous SNV	FA2H:NM_024306:exon1:c.G178A:p.A60T	.	1.943	.	0.0002	.		Fam22_f_m_aF_aF_uF	0/0;0/1;0/1;0/1;0/1
chr16	74999883	74999883	T	C	ZNRF1	exonic	nonsynonymous SNV	ZNRF1:NM_032268:exon1:c.T212C:p.L71P	.	1.303	.	.	.		Fam69_f_m_aM	0/1;0/0;0/1
chr16	74999994	74999994	G	C	ZNRF1	exonic	nonsynonymous SNV	ZNRF1:NM_032268:exon1:c.G323C:p.G108A	.	1.139	0.0001	0.0002	0.0001		Fam89_f_m_aM	0/1;0/0;0/1
chr16	75648499	75648499	C	A	TERF2IP	exonic	nonsynonymous SNV	TERF2IP:NM_018975:exon1:c.C617A:p.S206Y	.	1.927	0.0003	0.0001	0.0002		Fam77_f_m_aM_aF_uM_uFs	0/0;0/1;0/0;0/1;0/1;0/0
chr16	77194781	77194781	C	G	MON1B	exonic	nonsynonymous SNV	MON1B:NM_001286640:exon2:c.C484G:p.L162V,MON1B:NM_001286639:exon3:c.C595G:p.L199V,MON1B:NM_014940:exon4:c.C922G:p.L308V	.	1.105	7.35E-05	0.0002	7.67E-05		Fam113_f_m_aF_raM_uF	0/0;0/1;0/1;0/0;0/0
chr16	83899245	83899245	G	T	MLYCD	exonic	nonsynonymous SNV	MLYCD:NM_012213:exon1:c.G101T:p.G34V	.	1.545	.	.	.		Fam45_f_m_aM_uF	0/1;0/0;0/1;0/1
chr16	87343657	87343657	T	C	FBXO31	exonic	nonsynonymous SNV	FBXO31:NM_024735:exon4:c.A598G:p.K200E,FBXO31:NM_001282683:exon5:c.A82G:p.K28E	.	1.532	7.34E-05	0.0001	4.19E-05		Fam110_f_m_aM_aM_uMs	0/1;0/0;0/1;0/0;0/1
chr16	87851784	87851784	C	T	SLC7A5	exonic	nonsynonymous SNV	SLC7A5:NM_003486:exon2:c.G604A:p.A202T	.	1.753	.	.	6.98E-06		Fam36_f_m_aM_uM	0/1;0/0;0/1;0/0
chr16	88433270	88433270	C	T	ZNF469	exonic	stopgain	ZNF469:NM_001367624:exon1:c.C5800T:p.R1934X	0.552	.	.	.	.		Fam40_f_m_aM_aM	0/0;0/1;0/1;0/0
chr16	89290777	89290777	G	A	ANKRD11	exonic	nonsynonymous SNV	ANKRD11:NM_001256183:exon6:c.C449T:p.T150M,ANKRD11:NM_013275:exon6:c.C449T:p.T150M,ANKRD11:NM_001256182:exon7:c.C449T:p.T150M	.	1.991	7.35E-05	7.45E-05	1.40E-05		Fam62_f_m_aM_aM	0/0;0/1;0/0;0/1

chr17	1437104	1437104	G	A	CRK	exonic	nonsynonymous SNV	CRK:NM_005206:exon2:c.C293T:p.P98L,CRK:NM_016823:exon2:c.C293T:p.P98L	.	1.86	.	.	.	Fam38_f_m_aM	0/0;0/1;0/1
chr17	1538889	1538889	G	C	PITPNA	exonic	nonsynonymous SNV	PITPNA:NM_006224:exon7:c.C436G:p.R146G	.	1.889	0.0001	9.86E-05	2.09E-05	Fam99_f_m_aM_aM	0/0;0/1;0/1;0/0
chr17	1591428	1591428	T	A	SLC43A2	exonic	nonsynonymous SNV	SLC43A2:NM_001284499:exon4:c.A361T:p.T121S,SLC43A2:NM_001284498:exon8:c.A772T:p.T258S,SLC43A2:NM_001321364:exon8:c.A772T:p.T258S,SLC43A2:NM_001321365:exon8:c.A772T:p.T258S,SLC43A2:NM_152346:exon8:c.A772T:p.T258S	.	1.089	7.36E-05	1.12E-05	6.98E-06	Fam101_f_m_aM	0/0;0/1;0/1
chr17	1676690	1676690	T	C	PRPF8	exonic	nonsynonymous SNV	PRPF8:NM_006445:exon16:c.A2203G:p.I735V	.	1.312	.	.	.	Fam109_f_m_aM	0/1;0/0;0/1
chr17	1731222	1731222	T	C	WDR81	exonic	nonsynonymous SNV	WDR81:NM_001163673:exon4:c.T512C:p.L171P,WDR81:NM_001163809:exon4:c.T4121C:p.L1374P,WDR81:NM_001163811:exon4:c.T440C:p.L147P,WDR81:NM_152348:exon5:c.T968C:p.L323P	.	1.563	.	.	.	Fam71_f_m_aF	0/1;0/0;0/1
chr17	2670194	2670194	G	A	PAFAH1B1	exonic	nonsynonymous SNV	PAFAH1B1:NM_000430:exon6:c.G431A:p.R144Q	.	1.59	.	.	.	Fam15_f_m_aM_aM	0/0;0/0;0/0;0/1
chr17	3664211	3664211	C	T	TAX1BP3	exonic	nonsynonymous SNV	TAX1BP3:NM_014604:exon3:c.G221A:p.G74E	.	1.448	0.0001	3.36E-05	2.79E-05	Fam24_f_m_aM_aM	0/1;0/0;0/1;0/1
chr17	3813159	3813159	-	C	NCBP3	exonic	frameshift insertion	NCBP3:NM_001114118:exon13:c.1747dupG:p.A583Gfs*4	0.404	.	.	.	6.98E-06	Fam25_f_m_aM	0/0;0/1;0/1
chr17	3947675	3947675	C	A	ATP2A3	exonic	nonsynonymous SNV	ATP2A3:NM_005173:exon8:c.G811T:p.V271L,ATP2A3:NM_174953:exon8:c.G811T:p.V271L,ATP2A3:NM_174954:exon8:c.G811T:p.V271L,ATP2A3:NM_174955:exon8:c.G811T:p.V271L,ATP2A3:NM_174956:exon8:c.G811T:p.V271L,ATP2A3:NM_174957:exon8:c.G811T:p.V271L,ATP2A3:NM_174958:exon8:c.G811T:p.V271L	.	1.463	.	0.0002	.	Fam56_f_m_aF_aM	0/1;0/0;0/1;0/0
chr17	3951265	3951265	A	G	ATP2A3	exonic	nonsynonymous SNV	ATP2A3:NM_005173:exon5:c.T449C:p.I150T,ATP2A3:NM_174953:exon5:c.T449C:p.I150T,ATP2A3:NM_174954:exon5:c.T449C:p.I150T,ATP2A3:NM_174955:exon5:c.T449C:p.I150T,ATP2A3:NM_174956:exon5:c.T449C:p.I150T,ATP2A3:NM_174957:exon5:c.T449C:p.I150T,ATP2A3:NM_174958:exon5:c.T449C:p.I150T	.	1.631	0.0003	0.0002	0.0002	Fam80_f_m_aM_uM	0/1;0/0;0/1;0/1
chr17	4513256	4513256	T	C	SPNS2	exonic	nonsynonymous SNV	SPNS2:NM_001124758:exon2:c.T380C:p.L127P	.	1.29	.	.	.	Fam122_f_m_aM	0/1;0/0;0/1
chr17	4720429	4720429	C	A	ARRB2	exonic	nonsynonymous SNV	ARRB2:NM_001330064:exon11:c.C462A:p.H154Q,ARRB2:NM_001257331:exon12:c.C993A:p.H331Q,ARRB2:NM_199004:exon12:c.C993A:p.H331Q,ARRB2:NM_001257328:exon13:c.C1101A:p.H367Q,ARRB2:NM_001257329:exon13:c.C907A:p.P303T,ARRB2:NM_001257330:exon13:c.C1038A:p.H346Q,ARRB2:NM_004313:exon13:c.C1038A:p.H346Q	.	1.945	.	.	.	Fam110_f_m_aM_aM_uMs	0/0;0/1;0/0;0/1;0/0
chr17	4896211	4896211	C	T	MINK1	exonic	nonsynonymous SNV	MINK1:NM_015716:exon28:c.C3373T:p.H1125Y,MINK1:NM_001024937:exon29:c.C3424T:p.H1142Y,MINK1:NM_001321236:exon29:c.C3208T:p.H1070Y,MINK1:NM_153827:exon29:c.C3484T:p.H1162Y,MINK1:NM_170663:exon29:c.C3397T:p.H1133Y	.	1.951	7.35E-05	6.02E-05	3.49E-05	Fam12_f_m_aM_uM_aM	0/0;0/1;0/1;0/0;0/0
chr17	5001351	5001351	T	C	KIF1C	exonic	nonsynonymous SNV	KIF1C:NM_006612:exon5:c.T313C:p.Y105H	.	1.105	.	.	.	Fam119_f_m_aM_aM	0/1;0/0;0/0;0/1
chr17	5438130	5438130	C	T	C1QBP	exonic	nonsynonymous SNV	C1QBP:NM_001212:exon2:c.G376A:p.G126R	.	1.141	.	0.0005	7.68E-05	Fam86_f_m_aF	0/0;0/1;0/1
chr17	6471163	6471163	C	G	PITPNM3	exonic	nonsynonymous SNV	PITPNM3:NM_001165966:exon11:c.G1514C:p.R505P,PITPNM3:NM_031220:exon12:c.G1622C:p.R541P	.	1.097	.	.	1.40E-05	Fam69_f_m_aM	0/1;0/0;0/1
chr17	6483736	6483736	C	T	PITPNM3	exonic	nonsynonymous SNV	PITPNM3:NM_001165966:exon5:c.G260A:p.R87H,PITPNM3:NM_031220:exon6:c.G368A:p.R123H	.	1.067	7.35E-05	6.82E-05	6.98E-06	Fam26_f_m_aM_uM	0/1;0/0;0/1;0/1
chr17	7026613	7026613	T	C	BCL6B	exonic	nonsynonymous SNV	BCL6B:NM_181844:exon6:c.T1046C:p.V349A	.	1.291	0.0001	0.0001	2.79E-05	Fam4_f_m_aM	0/1;0/0;0/1
chr17	7323503	7323503	T	C	NEURL4	exonic	nonsynonymous SNV	NEURL4:NM_001005408:exon14:c.A2399G:p.Y800C,NEURL4:NM_032442:exon14:c.A2399G:p.Y800C	.	1.589	0.0004	0.0001	0.0001	Fam45_f_m_aM_uF	0/1;0/0;0/1;0/0
chr17	7323905	7323905	C	T	NEURL4	exonic	nonsynonymous SNV	NEURL4:NM_001005408:exon12:c.G2170A:p.G724S,NEURL4:NM_032442:exon12:c.G2170A:p.G724S	.	1.464	.	1.12E-05	6.98E-06	Fam115_f_m_aF_aM_aF	0/0;0/0;0/1;0/0;0/0
chr17	7344077	7344077	G	A	ACAP1	exonic	nonsynonymous SNV	ACAP1:NM_014716:exon9:c.G698A:p.R233Q	.	1.269	.	1.31E-05	.	Fam116_f_m_aM_aF	0/0;0/1;0/1;0/1
chr17	7408418	7408418	C	T	NLGN2	exonic	nonsynonymous SNV	NLGN2:NM_020795:exon1:c.C163T:p.R55W	.	2.265	.	4.37E-05	.	Fam88_f_m_aF	0/0;0/1;0/1
chr17	7415791	7415791	G	A	NLGN2	exonic	nonsynonymous SNV	NLGN2:NM_020795:exon6:c.G1318A:p.D440N	.	2.298	7.34E-05	7.82E-05	4.88E-05	Fam48_f_m_aM_uM	0/1;0/0;0/1;0/0
chr17	7417174	7417174	G	A	NLGN2	exonic	nonsynonymous SNV	NLGN2:NM_020795:exon7:c.G1883A:p.R628H	.	1.363	7.37E-05	0.0002	0.0002	Fam43_f_m_aM	0/1;0/0;0/1
chr17	7417323	7417323	G	A	NLGN2	exonic	nonsynonymous SNV	NLGN2:NM_020795:exon7:c.G2032A:p.V678I	.	2.015	.	.	.	Fam81_f_m_aM_uM	0/1;0/0;0/1;0/0
chr17	7446929	7446929	G	A	CHRNB1	exonic	nonsynonymous SNV	CHRNB1:NM_000747:exon4:c.G340A:p.V114M	.	1.236	0.0007	0.0005	0.0002	Fam4_f_m_aM	0/0;0/1;0/1
chr17	7556873	7556873	C	T	TNFSF12;TNFSF12	exonic	nonsynonymous SNV	TNFSF12:NM_003809:exon6:c.C469T:p.R157W,TNFSF12-TNFSF13:NM_172089:exon6:c.C469T:p.R157W	.	1.312	7.35E-05	0.0001	2.79E-05	Fam63_f_m_aF	0/0;0/1;0/1
chr17	7777504	7777504	G	A	DNAH2	exonic	nonsynonymous SNV	DNAH2:NM_020877:exon33:c.G5117A:p.R1706Q	.	1.016	0.0006	0.0008	0.0002	Fam30_f_m_aM_uM	0/0;0/1;0/1;0/0
chr17	7819231	7819231	G	A	DNAH2	exonic	nonsynonymous SNV	DNAH2:NM_020877:exon72:c.G10838A:p.R3613Q	.	1.025	.	3.27E-05	1.40E-05	Fam16_f_m_aM_aM	0/0;0/1;0/0;0/1
chr17	7905904	7905904	A	G	CHD3	exonic	nonsynonymous SNV	CHD3:NM_001005271:exon28:c.A4450G:p.M1484V,CHD3:NM_001005273:exon28:c.A4273G:p.M1425V,CHD3:NM_005852:exon28:c.A4273G:p.M1425V	.	1.534	.	.	.	Fam67_f_m_aM_uF	0/0;0/1;0/1;0/1
chr17	7910572	7910572	C	T	CHD3	exonic	nonsynonymous SNV	CHD3:NM_005852:exon37:c.C5633T:p.T1878M,CHD3:NM_001005271:exon38:c.C5912T:p.T1971M,CHD3:NM_001005273:exon38:c.C5735T:p.T1912M	.	1.224	0.0008	0.0005	0.0003	Fam53_f_m_aM	0/1;0/0;0/1
chr17	8015999	8015999	T	C	GUCY2D	exonic	nonsynonymous SNV	GUCY2D:NM_000180:exon17:c.T3116C:p.L1039P	.	1.818	.	.	6.98E-06	Fam13_f_m_aM	0/1;0/0;0/1
chr17	8542210	8542210	A	G	MYH10	exonic	nonsynonymous SNV	MYH10:NM_005964:exon13:c.T1472C:p.M491T,MYH10:NM_001256012:exon14:c.T1502C:p.M501T,MYH10:NM_001256095:exon14:c.T1499C:p.M500T	.	1.122	.	.	.	Fam72_f_m_aF_uF	0/0;0/1;0/1;0/1
chr17	8623221	8623221	T	C	MYH10	exonic	nonsynonymous SNV	MYH10:NM_001256012:exon2:c.A26G:p.D9G,MYH10:NM_001256095:exon2:c.A26G:p.D9G,MYH10:NM_005964:exon2:c.A26G:p.D9G	.	2.4	7.34E-05	3.50E-05	1.40E-05	Fam27_f_m_aF_uM_uM	0/1;0/0;0/1;0/1;0/1

chr17	8909156	8909156	C	G	PIK3R5	exonic	nonsynonymous SNV	PIK3R5:NM_001142633:exon3:c.G122C:p.C41S,PIK3R5:NM_014308:exon3:c.G122C:p.C41S	.	1.38	.	.	.		Fam121_f_m_aF_aM	0/0;0/1;0/1;0/1
chr17	10528976	10528976	C	T	MYH2	exonic	nonsynonymous SNV	MYH2:NM_001100112:exon27:c.G3458A:p.S1153N,MYH2:NM_017534:exon27:c.G3458A:p.S1153N	.	1.033	.	.	.		Fam77_f_m_aM_aF_uM_uFs	0/0;0/0;0/1;0/0;0/0;0/0
chr17	10640363	10640363	C	T	MYH3	exonic	nonsynonymous SNV	MYH3:NM_002470:exon21:c.G2396A:p.R799H	.	2.1	.	9.80E-05	6.98E-06		Fam25_f_m_aM	0/0;0/1;0/1
chr17	10640375	10640375	C	G	MYH3	exonic	nonsynonymous SNV	MYH3:NM_002470:exon21:c.G2384C:p.G795A	.	1.971	7.34E-05	0.0001	3.49E-05		Fam4_f_m_aM	0/0;0/1;0/1
chr17	10729805	10729805	A	G	TMEM220	exonic	nonsynonymous SNV	TMEM220:NM_001004313:exon1:c.T47C:p.F16S,TMEM220:NM_001330139:exon1:c.T47C:p.F16S,TMEM220:NM_001330140:exon1:c.T47C:p.F16S,TMEM220:NM_001359647:exon1:c.T47C:p.F16S	.	1.525	.	.	.		Fam36_f_m_aM_uM	0/1;0/0;0/1;0/0
chr17	10729806	10729806	A	C	TMEM220	exonic	nonsynonymous SNV	TMEM220:NM_001004313:exon1:c.T46G:p.F16V,TMEM220:NM_001330139:exon1:c.T46G:p.F16V,TMEM220:NM_001330140:exon1:c.T46G:p.F16V,TMEM220:NM_001359647:exon1:c.T46G:p.F16V	.	1.296	.	.	.		Fam36_f_m_aM_uM	0/1;0/0;0/1;0/0
chr17	12915955	12915955	C	T	ARHGAP44	exonic	nonsynonymous SNV	ARHGAP44:NM_001321164:exon5:c.C331T:p.H111Y,ARHGAP44:NM_001321166:exon5:c.C331T:p.H111Y,ARHGAP44:NM_001321167:exon5:c.C331T:p.H111Y,ARHGAP44:NM_014859:exon5:c.C331T:p.H111Y	.	1.449	7.34E-05	9.00E-05	1.40E-05		Fam82_f_m_aM_uF	0/1;0/0;0/1;0/0
chr17	16064885	16064885	C	G	NCOR1	exonic	nonsynonymous SNV	NCOR1:NM_001190440:exon33:c.G5134C:p.A1712P,NCOR1:NM_006311:exon34:c.G5086C:p.A1696P	.	1.101	7.34E-05	0.0003	0.0002		Fam117_f_m_aM_aF	0/0;0/1;0/0;0/1
chr17	16064885	16064885	C	G	NCOR1	exonic	nonsynonymous SNV	NCOR1:NM_001190440:exon33:c.G5134C:p.A1712P,NCOR1:NM_006311:exon34:c.G5086C:p.A1696P	.	1.101	7.34E-05	0.0003	0.0002		Fam12_f_m_aM_uM_aM	0/0;0/1;0/1;0/1;0/0
chr17	16065554	16065554	G	A	NCOR1	exonic	nonsynonymous SNV	NCOR1:NM_001190440:exon32:c.C4930T:p.R1644C,NCOR1:NM_006311:exon33:c.C4882T:p.R1628C	.	1.129	7.34E-05	.	6.98E-06		Fam67_f_m_aM_uF	0/0;0/1;0/1;0/1
chr17	16171876	16171876	C	T	NCOR1	exonic	nonsynonymous SNV	NCOR1:NM_001190440:exon3:c.G362A:p.R121H,NCOR1:NM_006311:exon4:c.G362A:p.R121H	.	1.923	.	3.31E-05	.		Fam25_f_m_aM	0/1;0/0;0/1
chr17	17221617	17221617	G	A	FLCN	exonic	nonsynonymous SNV	FLCN:NM_144606:exon8:c.C791T:p.A264V,FLCN:NM_144997:exon8:c.C791T:p.A264V,FLCN:NM_001353230:exon9:c.C791T:p.A264V,FLCN:NM_001353231:exon9:c.C791T:p.A264V,FLCN:NM_001353229:exon10:c.C845T:p.A282V	.	1.145	.	9.81E-05	6.98E-06		Fam75_f_m_aM	0/1;0/0;0/1
chr17	17346937	17346937	G	A	NT5M	exonic	nonsynonymous SNV	NT5M:NM_020201:exon5:c.G677A:p.R226Q	.	1.125	.	7.47E-05	6.98E-06		Fam1_f_m_aM	0/0;0/1;0/1
chr17	17813391	17813391	C	T	SREBF1	exonic	nonsynonymous SNV	SREBF1:NM_001321096:exon18:c.G3119A:p.R1040Q,SREBF1:NM_004176:exon18:c.G3191A:p.R1064Q,SREBF1:NM_001005291:exon19:c.G3281A:p.R1094Q	.	1.089	.	0.0001	4.89E-05		Fam122_f_m_aM	0/1;0/0;0/1
chr17	18264154	18264154	G	A	MIEF2	exonic	nonsynonymous SNV	MIEF2:NM_139162:exon4:c.G755A:p.R252H,MIEF2:NM_148886:exon4:c.G788A:p.R263H	.	1.038	0.0003	0.0003	0.0003		Fam118_f_m_aM_aF_uM	0/0;0/1;0/1;0/0;0/1
chr17	18889849	18889849	G	A	PRPSAP2	exonic	nonsynonymous SNV	PRPSAP2:NM_001243936:exon6:c.G436A:p.V146M,PRPSAP2:NM_001243941:exon6:c.G298A:p.V100M,PRPSAP2:NM_001353103:exon6:c.G298A:p.V100M,PRPSAP2:NM_001353104:exon6:c.G298A:p.V100M,PRPSAP2:NM_001243940:exon7:c.G556A:p.V186M,PRPSAP2:NM_001243942:exon7:c.G298A:p.V100M,PRPSAP2:NM_001353101:exon7:c.G556A:p.V186M,PRPSAP2:NM_001353105:exon7:c.G556A:p.V186M,PRPSAP2:NM_001353107:exon7:c.G556A:p.V186M,PRPSAP2:NM_001353096:exon8:c.G421A:p.V141M,PRPSAP2:NM_001353097:exon8:c.G421A:p.V141M,PRPSAP2:NM_001353098:exon8:c.G718A:p.V240M,PRPSAP2:NM_001353099:exon8:c.G421A:p.V141M,PRPSAP2:NM_001353100:exon8:c.G298A:p.V100M,PRPSAP2:NM_001353102:exon8:c.G556A:p.V186M,PRPSAP2:NM_001353106:exon8:c.G556A:p.V186M,PRPSAP2:NM_002767:exon8:c.G556A:p.V186M	.	1.887	0.0001	0.0003	5.59E-05		Fam66_f_m_aM	0/1;0/0;0/1
chr17	19415190	19415190	C	A	RNF112	exonic	nonsynonymous SNV	RNF112:NM_007148:exon11:c.C1279A:p.L427I	.	1.09	.	0.0001	1.40E-05		Fam22_f_m_aF_aF_uF	0/1;0/0;0/1;0/1;0/0
chr17	27597293	27597293	T	C	KSR1	exonic	nonsynonymous SNV	KSR1:NM_001367810:exon9:c.T1325C:p.L442P,KSR1:NM_014238:exon10:c.T914C:p.L305P	.	1.495	7.36E-05	0.0005	7.68E-05		Fam19_f_m_aM	0/0;0/1;0/1
chr17	28681890	28681890	G	T	SUPT6H	exonic	nonsynonymous SNV	SUPT6H:NM_003170:exon13:c.G1507T:p.D503Y,SUPT6H:NM_001320755:exon14:c.G1507T:p.D503Y	.	1.021	.	.	.		Fam96_f_m_aM_uF	0/0;0/1;0/1;0/1
chr17	28722809	28722809	T	C	RPL23A	exonic	nonsynonymous SNV	RPL23A:NM_000984:exon3:c.T296C:p.I99T	.	1.928	0.0005	0.0006	0.0003		Fam95_f_m_aM_aM_uF	0/1;0/0;0/1;0/1;0/0
chr17	28749267	28749267	G	A	TRAF4	exonic	nonsynonymous SNV	TRAF4:NM_004295:exon7:c.G1103A:p.R368H	.	1.935	.	7.45E-05	3.49E-05		Fam63_f_m_aF	0/1;0/0;0/1
chr17	29577706	29577706	G	A	GIT1	exonic	nonsynonymous SNV	GIT1:NM_014030:exon10:c.C920T:p.T307I,GIT1:NM_001085454:exon11:c.C947T:p.T316I	.	1.349	.	.	.		Fam123_f_aF	0/1;0/1
chr17	29610746	29610746	A	G	ANKRD13B	exonic	nonsynonymous SNV	ANKRD13B:NM_152345:exon8:c.A884G:p.Q295R	.	1.732	.	.	.		Fam95_f_m_aM_aM_uF	0/0;0/1;0/1;0/1;0/0
chr17	29611975	29611975	C	T	ANKRD13B	exonic	nonsynonymous SNV	ANKRD13B:NM_152345:exon10:c.C1069T:p.R357C	.	2.223	7.35E-05	3.28E-05	6.98E-06		Fam30_f_m_aM_uM	0/0;0/1;0/1;0/0
chr17	29650713	29650713	T	G	SSH2	exonic	nonsynonymous SNV	SSH2:NM_033389:exon12:c.A1086C:p.E362D,SSH2:NM_001282129:exon13:c.A1167C:p.E389D	.	1.978	.	1.12E-05	6.98E-06		Fam17_f_m_aM_uM	0/0;0/1;0/1;0/0
chr17	29677698	29677698	T	A	SSH2	exonic	nonsynonymous SNV	SSH2:NM_001282131:exon5:c.A463T:p.T155S,SSH2:NM_001282130:exon6:c.A442T:p.T148S,SSH2:NM_033389:exon6:c.A442T:p.T148S,SSH2:NM_001282129:exon7:c.A523T:p.T175S	.	1.736	.	.	.		Fam110_f_m_aM_aM_uMs	0/0;0/1;0/0;0/1;0/0
chr17	30449576	30449576	G	A	CPD	exonic	nonsynonymous SNV	CPD:NM_001199775:exon13:c.G2156A:p.R719H,CPD:NM_001304:exon13:c.G2897A:p.R966H	.	1	.	0.0001	6.99E-06		Fam36_f_m_aM_uM	0/0;0/1;0/1;0/1
chr17	30926914	30926914	T	C	ADAP2	exonic	nonsynonymous SNV	ADAP2:NM_001346712:exon3:c.T331C:p.C111R,ADAP2:NM_001346714:exon3:c.T313C:p.C105R,ADAP2:NM_001346716:exon3:c.T313C:p.C105R,ADAP2:NM_018404:exon3:c.T313C:p.C105R	.	1.567	.	.	.		Fam47_f_m_aM	0/1;0/0;0/1
chr17	31201469	31201469	A	G	NF1	exonic	nonsynonymous SNV	NF1:NM_000267:exon11:c.A1244G:p.H415R,NF1:NM_001042492:exon11:c.A1244G:p.H415R,NF1:NM_001128147:exon11:c.A1244G:p.H415R	.	1.055	.	.	.		Fam61_f_m_aM	0/0;0/1;0/1

chr17	31434092	31434092	C	A	RAB11FIP4	exonic	stopgain	RAB11FIP4:NM_032932:exon3:c.C306A:p.C102X	0.5	.	.	.	.	Fam109_f_m_aM	0/1;0/0;0/1
chr17	31988443	31988443	G	A	SUZ12	exonic	nonsynonymous SNV	SUZ12:NM_001321207:exon9:c.G1078A:p.E360K,SUZ12:NM_015355:exon10:c.G1147A:p.E383K	.	1.196	.	.	.	Fam54_f_m_aM_uF	0/0;0/1;0/1;0/0
chr17	32288819	32288819	C	T	RHBDL3	exonic	nonsynonymous SNV	RHBDL3:NM_001363834:exon2:c.C139T:p.R47C,RHBDL3:NM_001330181:exon3:c.C298T:p.R100C,RHBDL3:NM_001363835:exon4:c.C322T:p.R108C,RHBDL3:NM_001363836:exon4:c.C28T:p.R10C,RHBDL3:NM_138328:exon4:c.C322T:p.R108C	.	1.028	.	0.0003	2.09E-05	Fam77_f_m_aM_aF_uM_uFs	0/1;0/0;0/1;0/1;0/1;0/0
chr17	32365414	32365414	C	G	ZNF207	exonic	nonsynonymous SNV	ZNF207:NM_003457:exon7:c.C707G:p.P236R,ZNF207:NM_001032293:exon8:c.C755G:p.P252R,ZNF207:NM_001098507:exon8:c.C755G:p.P252R	.	1.111	.	.	.	Fam101_f_m_aM	0/0;0/1;0/1
chr17	33292010	33292010	C	T	ASIC2	exonic	nonsynonymous SNV	ASIC2:NM_183377:exon1:c.G106A:p.G36R	.	1.389	.	.	.	Fam47_f_m_aM	0/0;0/1;0/1
chr17	34637852	34637852	G	A	TMEM132E	exonic	nonsynonymous SNV	TMEM132E:NM_001304438:exon9:c.G2845A:p.E949K	.	1.163	.	1.16E-05	1.40E-05	Fam64_f_m_aM	0/1;0/0;0/1
chr17	34961724	34961724	G	T	ZNF830	exonic	nonsynonymous SNV	ZNF830:NM_052857:exon1:c.G158T:p.C53F	.	1.527	.	9.80E-05	.	Fam73_f_m_aF_dM	0/1;0/0;0/1;0/1
chr17	34961747	34961747	A	G	ZNF830	exonic	nonsynonymous SNV	ZNF830:NM_052857:exon1:c.A181G:p.K61E	.	1.451	.	1.12E-05	2.09E-05	Fam4_f_m_aM	0/1;0/0;0/1
chr17	34962634	34962634	A	C	ZNF830	exonic	nonsynonymous SNV	ZNF830:NM_052857:exon1:c.A1068C:p.E356D	.	1.153	.	.	.	Fam116_f_m_aM_aF	0/0;0/1;0/1;0/1
chr17	35136183	35136183	A	G	NLE1	exonic	nonsynonymous SNV	NLE1:NM_001014445:exon8:c.T121C:p.Y41H,NLE1:NM_018096:exon9:c.T997C:p.Y333H	.	1.2	.	.	.	Fam31_f_m_aM_uF	0/0;0/1;0/1;0/0
chr17	39175200	39175200	A	T	CACNB1	exonic	nonsynonymous SNV	CACNB1:NM_000723:exon14:c.T1790A:p.I597N	.	1.024	.	.	.	Fam22_f_m_aF_aF_uF	0/1;0/0;0/1;0/1;0/0
chr17	39419878	39419878	T	G	MED1	exonic	nonsynonymous SNV	MED1:NM_004774:exon14:c.A1136C:p.D379A	.	1.052	.	3.35E-05	.	Fam5_f_m_aM	0/1;0/0;0/1
chr17	39633892	39633892	G	A	PPP1R1B	exonic	nonsynonymous SNV	PPP1R1B:NM_001242464:exon5:c.G143A:p.R48H,PPP1R1B:NM_032192:exon5:c.G251A:p.R84H,PPP1R1B:NM_181505:exon5:c.G143A:p.R48H	.	1.369	7.34E-05	6.54E-05	2.79E-05	Fam106_f_m_aM	0/1;0/0;0/1
chr17	39715792	39715792	C	T	ERBB2	exonic	nonsynonymous SNV	ERBB2:NM_001289937:exon12:c.C1366T:p.R456C,ERBB2:NM_004448:exon12:c.C1366T:p.R456C,ERBB2:NM_001005862:exon15:c.C1276T:p.R426C,ERBB2:NM_001289938:exon15:c.C1276T:p.R426C,ERBB2:NM_001289936:exon16:c.C1321T:p.R441C	.	1.305	.	0.0001	2.09E-05	Fam80_f_m_aM_uM	0/0;0/1;0/1;0/0
chr17	39730199	39730199	C	T	MIEN1	exonic	nonsynonymous SNV	MIEN1:NM_001330206:exon2:c.G182A:p.G61D,MIEN1:NM_032339:exon2:c.G182A:p.G61D	.	1.292	.	2.32E-05	1.40E-05	Fam52_f_m_aM	0/1;0/0;0/1
chr17	40027940	40027940	A	T	MED24	exonic	nonsynonymous SNV	MED24:NM_001079518:exon14:c.T1377A:p.N459K,MED24:NM_001267797:exon14:c.T1377A:p.N459K,MED24:NM_014815:exon15:c.T1416A:p.N472K,MED24:NM_001330211:exon16:c.T1473A:p.N491K	.	3.339	.	.	.	Fam70_f_m_aM	0/0;0/1;0/1
chr17	40129538	40129538	C	T	MSL1	exonic	nonsynonymous SNV	MSL1:NM_001365919:exon3:c.C1286T:p.T429I,MSL1:NM_001365920:exon3:c.C1286T:p.T429I,MSL1:NM_001365921:exon3:c.C1286T:p.T429I,MSL1:NM_001012241:exon4:c.C497T:p.T166I	.	1.021	.	.	.	Fam116_f_m_aM_aF	0/1;0/0;0/1;0/0
chr17	40363019	40363019	G	C	GJD3	exonic	nonsynonymous SNV	GJD3:NM_152219:exon1:c.C797G:p.A266G	.	2.491	.	.	.	Fam70_f_m_aM	0/1;0/0;0/1
chr17	40363508	40363508	T	C	GJD3	exonic	nonsynonymous SNV	GJD3:NM_152219:exon1:c.A308G:p.E103G	.	1.452	.	.	.	Fam64_f_m_aM	0/0;0/1;0/1
chr17	40555118	40555118	C	T	CCR7	exonic	nonsynonymous SNV	CCR7:NM_001301714:exon2:c.G572A:p.R191H,CCR7:NM_001301716:exon3:c.G743A:p.R248H,CCR7:NM_001301717:exon3:c.G743A:p.R248H,CCR7:NM_001301718:exon3:c.G743A:p.R248H,CCR7:NM_001838:exon3:c.G761A:p.R254H	.	1.104	.	0.0001	.	Fam6_f_m_aM	0/0;0/1;0/1
chr17	41525219	41525219	G	A	KRT19	exonic	nonsynonymous SNV	KRT19:NM_002276:exon2:c.C475T:p.R159C	.	1.164	7.35E-05	0.0001	7.68E-05	Fam67_f_m_aM_uF	0/0;0/1;0/1;0/1
chr17	41968488	41968488	C	G	CNP	exonic	nonsynonymous SNV	CNP:NM_001330216:exon2:c.C364G:p.Q122E,CNP:NM_033133:exon2:c.C424G:p.Q142E	.	1.408	.	.	.	Fam63_f_m_aF	0/1;0/0;0/1
chr17	41973590	41973590	C	T	CNP	exonic	nonsynonymous SNV	CNP:NM_001330216:exon4:c.C872T:p.P291L,CNP:NM_033133:exon4:c.C932T:p.P311L	.	1.026	.	3.28E-05	1.40E-05	Fam39_f_m_aM	0/0;0/1;0/1
chr17	41997236	41997236	A	G	DNAJC7	exonic	startloss	DNAJC7:NM_001144766:exon3:c.T2C:p.M1?,DNAJC7:NM_003315:exon3:c.T170C:p.M57T	0.393	0.716	7.36E-05	9.04E-05	6.28E-05	Fam85_f_m_aM_aM	0/1;0/0;0/0;0/1
chr17	42111835	42111835	T	A	DHX58	exonic	nonsynonymous SNV	DHX58:NM_024119:exon3:c.A58T:p.I20F	.	1.069	.	.	.	Fam115_f_m_aF_aM_aF	0/0;0/1;0/1;0/1;0/0
chr17	42113734	42113734	G	A	KAT2A	exonic	nonsynonymous SNV	KAT2A:NM_021078:exon18:c.C2429T:p.P810L	.	2.044	.	3.31E-05	2.79E-05	Fam109_f_m_aM	0/1;0/0;0/1
chr17	42201778	42201778	C	T	STAT5B	exonic	nonsynonymous SNV	STAT5B:NM_012448:exon19:c.G2324A:p.R775Q	.	2.023	.	0.0002	6.98E-06	Fam15_f_m_aM_aM	0/1;0/0;0/1;0/0
chr17	42564465	42564465	T	C	COASY	exonic	nonsynonymous SNV	COASY:NM_025233:exon3:c.T935C:p.L312S,COASY:NM_001042529:exon4:c.T935C:p.L312S,COASY:NM_001042532:exon5:c.T1022C:p.L341S	.	1.032	.	1.12E-05	6.98E-06	Fam64_f_m_aM	0/0;0/1;0/1
chr17	42567129	42567129	C	T	MLX	exonic	nonsynonymous SNV	MLX:NM_170607:exon1:c.C5T:p.T2M,MLX:NM_198204:exon1:c.C5T:p.T2M,MLX:NM_198205:exon1:c.C5T:p.T2M	.	1.028	.	.	.	Fam112_f_m_aM	0/0;0/1;0/1
chr17	42613856	42613856	C	T	TUBG1	exonic	nonsynonymous SNV	TUBG1:NM_001070:exon8:c.C701T:p.T234I	.	2.198	.	.	.	Fam28_f_m_aF_uF	0/1;0/0;0/1;0/1
chr17	42670124	42670124	G	C	PLEKHH3	exonic	nonsynonymous SNV	PLEKHH3:NM_024927:exon11:c.C1807G:p.R603G	.	1.82	.	.	7.18E-06	Fam114_f_m_aM	0/0;0/1;0/1
chr17	42680401	42680401	G	C	CCR10	exonic	nonsynonymous SNV	CCR10:NM_016602:exon2:c.C241G:p.L81V	.	2.26	7.34E-05	0.0001	6.98E-05	Fam82_f_m_aM_uF	0/1;0/0;0/1;0/0
chr17	42687886	42687886	G	C	CNTNAP1	exonic	nonsynonymous SNV	CNTNAP1:NM_003632:exon8:c.G1211C:p.G404A	.	1.078	.	3.27E-05	6.98E-06	Fam7_f_m_aM_aM_uM	0/0;0/1;0/0;0/1;0/1
chr17	42688507	42688507	C	T	CNTNAP1	exonic	nonsynonymous SNV	CNTNAP1:NM_003632:exon9:c.C1352T:p.A451V	.	1.245	.	.	.	Fam91_m_aM_dM_aM_dM	0/0;0/1;0/1;0/1;0/0
chr17	42692715	42692715	A	G	CNTNAP1	exonic	nonsynonymous SNV	CNTNAP1:NM_003632:exon17:c.A2747G:p.Y916C	.	1.036	.	0.0001	5.59E-05	Fam21_f_m_aM_uM	0/1;0/0;0/1;0/1
chr17	43018696	43018696	T	G	VAT1	exonic	nonsynonymous SNV	VAT1:NM_006373:exon2:c.A491C:p.E164A	.	1.162	.	.	.	Fam85_f_m_aM_aM	0/0;0/1;0/1;0/1

chr17	43504798	43504798	C	G	DHX8	exonic	nonsynonymous SNV	DHX8:NM_001322217:exon11:c.C1428G:p.I476M,DHX8:NM_001302623:exon12:c.C1701G:p.I567M,DHX8:NM_001322216:exon12:c.C876G:p.I292M,DHX8:NM_001322218:exon12:c.C1695G:p.I565M,DHX8:NM_001322219:exon12:c.C1701G:p.I567M,DHX8:NM_001322220:exon12:c.C1701G:p.I567M,DHX8:NM_001322221:exon12:c.C1695G:p.I565M,DHX8:NM_004941:exon12:c.C1701G:p.I567M	.	2.561	.	.	.		Fam20_f_m_aM_uF	0/0;0/1;0/1;0/0
chr17	43811181	43811181	T	C	MPP3	exonic	nonsynonymous SNV	MPP3:NM_001330233:exon17:c.A1355G:p.H452R,MPP3:NM_001932:exon17:c.A1280G:p.H427R,MPP3:NM_001353080:exon18:c.A1304G:p.H435R	.	1.221	0.0004	0.0007	0.0003	Fam92_f_m_aM_raM	0/0;0/0;0/0;0/1
chr17	43879892	43879892	G	A	MPP2	exonic	nonsynonymous SNV	MPP2:NM_001278371:exon10:c.C1210T:p.R404C,MPP2:NM_001278373:exon10:c.C1210T:p.R404C,MPP2:NM_001278374:exon10:c.C826T:p.R276C,MPP2:NM_001278375:exon10:c.C1210T:p.R404C,MPP2:NM_001278376:exon10:c.C1294T:p.R432C,MPP2:NM_001278370:exon11:c.C1378T:p.R460C,MPP2:NM_005374:exon11:c.C1243T:p.R415C,MPP2:NM_001278372:exon12:c.C1315T:p.R439C,MPP2:NM_001278381:exon12:c.C1243T:p.R415C	.	1.773	.	0.0001	6.99E-06	Fam74_f_m_aM_uM	0/1;0/0;0/1;0/1
chr17	44007460	44007460	C	T	NAGS	exonic	nonsynonymous SNV	NAGS:NM_153006:exon5:c.C1234T:p.R412C	.	2.017	0.0003	0.0003	0.0001	Fam81_f_m_aM_uM	0/0;0/1;0/1;0/0
chr17	44007460	44007460	C	T	NAGS	exonic	nonsynonymous SNV	NAGS:NM_153006:exon5:c.C1234T:p.R412C	.	2.017	0.0003	0.0003	0.0001	Fam107_f_m_aM	0/1;0/0;0/1
chr17	44007635	44007635	G	T	NAGS	exonic	nonsynonymous SNV	NAGS:NM_153006:exon6:c.G1313T:p.G438V	.	2.034	.	.	.	Fam87_f_m_aM_uM	0/1;0/0;0/1;0/0
chr17	44074230	44074230	G	T	G6PC3	exonic	nonsynonymous SNV	G6PC3:NM_001319945:exon2:c.G289T:p.V97F,G6PC3:NM_138387:exon2:c.G289T:p.V97F	.	1	.	.	.	Fam106_f_m_aM	0/0;0/1;0/1
chr17	44091461	44091461	G	A	HDAC5	exonic	nonsynonymous SNV	HDAC5:NM_001015053:exon11:c.C1199T:p.A400V,HDAC5:NM_005474:exon11:c.C1196T:p.A399V	.	1.038	.	.	.	Fam52_f_m_aM	0/1;0/0;0/1
chr17	44176980	44176980	T	G	ASB16	exonic	nonsynonymous SNV	ASB16:NM_080863:exon3:c.T812G:p.L271R	.	1.258	.	.	.	Fam15_f_m_aM_aM	0/1;0/0;0/0;0/1
chr17	44210433	44210433	G	A	UBTF	exonic	nonsynonymous SNV	UBTF:NM_001076683:exon13:c.C1289T:p.S430L,UBTF:NM_001076684:exon13:c.C1289T:p.S430L,UBTF:NM_014233:exon14:c.C1400T:p.S467L	.	1.314	.	.	.	Fam21_f_m_aM_uM	0/1;0/0;0/1;0/1
chr17	44259173	44259173	A	G	SLC4A1	exonic	nonsynonymous SNV	SLC4A1:NM_000342:exon9:c.T866C:p.M289T	.	1.182	.	3.28E-05	6.98E-06	Fam65_f_m_aM_uF_dF	0/0;0/1;0/1;0/0;0/1
chr17	44259320	44259320	G	A	SLC4A1	exonic	nonsynonymous SNV	SLC4A1:NM_000342:exon9:c.C719T:p.P240L	.	1.125	7.35E-05	0.0002	3.49E-05	Fam99_f_m_aM_aM	0/0;0/1;0/1;0/0
chr17	44773366	44773366	C	T	ADAM11	exonic	nonsynonymous SNV	ADAM11:NM_001318933:exon11:c.C331T:p.R111W,ADAM11:NM_002390:exon11:c.C931T:p.R311W	.	1.258	0.0001	8.94E-05	0.0001	Fam16_f_m_aM_aM	0/1;0/0;0/0;0/1
chr17	44776903	44776903	G	A	ADAM11	exonic	nonsynonymous SNV	ADAM11:NM_001318933:exon20:c.G1022A:p.R341H,ADAM11:NM_002390:exon20:c.G1622A:p.R541H	.	1.018	.	3.28E-05	.	Fam59_f_m_aF_uM	0/0;0/1;0/1;0/0
chr17	44805429	44805429	G	A	GJC1	exonic	nonsynonymous SNV	GJC1:NM_001080383:exon3:c.C389T:p.T130M,GJC1:NM_005497:exon3:c.C389T:p.T130M	.	1.038	.	9.83E-05	2.79E-05	Fam15_f_m_aM_aM	0/0;0/1;0/1;0/1
chr17	45149768	45149768	T	G	HEXIM1	exonic	nonsynonymous SNV	HEXIM1:NM_006460:exon1:c.T578G:p.M193R	.	1.762	7.35E-05	0.0001	8.38E-05	Fam25_f_m_aM	0/0;0/1;0/1
chr17	45243961	45243961	C	T	FMNL1	exonic	nonsynonymous SNV	FMNL1:NM_005892:exon18:c.C2384T:p.P795L	.	1.369	0.0006	0.0008	6.28E-05	Fam20_f_m_aM_uF	0/1;0/0;0/1;0/0
chr17	45245274	45245274	C	T	FMNL1	exonic	nonsynonymous SNV	FMNL1:NM_005892:exon22:c.C2750T:p.A917V	.	1.506	0.0001	0.0001	9.07E-05	Fam105_f_m_aM	0/1;0/0;0/1
chr17	45245327	45245327	C	T	FMNL1	exonic	nonsynonymous SNV	FMNL1:NM_005892:exon22:c.C2803T:p.R935W	.	1.587	.	0.0001	1.40E-05	Fam65_f_m_aM_uF_dF	0/1;0/0;0/1;0/1;0/0
chr17	45398035	45398035	C	G	ARHGAP27	exonic	nonsynonymous SNV	ARHGAP27:NM_001282290:exon10:c.G1756C:p.D586H,ARHGAP27:NM_199282:exon10:c.G733C:p.D245H	.	1.292	0.0001	0.0003	9.77E-05	Fam47_f_m_aM	0/0;0/1;0/1
chr17	45834628	45834628	G	A	CRHR1;LINC0221C	exonic	nonsynonymous SNV	CRHR1:NM_001303018:exon11:c.G587A:p.R196H,CRHR1:NM_001145147:exon12:c.G992A:p.R331H,CRHR1:NM_001145148:exon12:c.G1070A:p.R357H,CRHR1:NM_001303020:exon12:c.G809A:p.R270H,LINC02210-CRHR1:NM_001303016:exon13:c.G673A:p.V225I,CRHR1:NM_004382:exon13:c.G1112A:p.R371H,CRHR1:NM_001145146:exon14:c.G1199A:p.R400H,LINC02210-CRHR1:NM_001256299:exon15:c.G587A:p.R196H	.	1.204	7.36E-05	0.0001	3.49E-05	Fam14_f_m_aM_aM	0/0;0/1;0/0;0/1
chr17	46038621	46038621	T	C	KANSL1	exonic	nonsynonymous SNV	KANSL1:NM_001193466:exon10:c.A2458G:p.S820G,KANSL1:NM_015443:exon10:c.A2458G:p.S820G,KANSL1:NM_001193465:exon11:c.A2458G:p.S820G	.	1.28	.	2.23E-05	.	Fam56_f_m_aF_aM	0/0;0/1;0/0;0/1
chr17	46711008	46711008	G	A	NSF	exonic	nonsynonymous SNV	NSF:NM_006178:exon14:c.G1516A:p.G506S	.	1.148	.	9.38E-05	6.98E-06	Fam23_f_m_aM_dF_uFs	0/0;0/1;0/1;0/0;0/1
chr17	47283375	47283375	C	T	ITGB3	exonic	nonsynonymous SNV	ITGB3:NM_000212:exon3:c.C187T:p.R63C	.	1.457	0.0001	7.45E-05	2.09E-05	Fam40_f_m_aM_aM	0/0;0/1;0/1;0/0
chr17	47300558	47300558	T	C	ITGB3	exonic	nonsynonymous SNV	ITGB3:NM_000212:exon12:c.T1994C:p.I665T	.	1.37	.	3.28E-05	.	Fam55_f_m_aM_aM_dM	0/1;0/0;0/0;0/1;0/0
chr17	47310197	47310197	G	T	ITGB3	exonic	nonsynonymous SNV	ITGB3:NM_000212:exon15:c.G2360T:p.G787V	.	1.398	.	3.35E-05	.	Fam37_f_m_aF_uM	0/1;0/0;0/1;0/0
chr17	47605523	47605523	G	T	NPEPPS	exonic	nonsynonymous SNV	NPEPPS:NM_006310:exon17:c.G2066T:p.R689I,NPEPPS:NM_001330257:exon18:c.G2054T:p.R685I	.	1.534	.	.	.	Fam7_f_m_aM_aM_uM	0/0;0/1;0/1;0/0;0/0
chr17	47650433	47650433	G	A	KPNB1	exonic	nonsynonymous SNV	KPNB1:NM_002265:exon2:c.G88A:p.V30M	.	1.477	.	.	.	Fam12_f_m_aM_uM_aM	0/1;0/0;0/1;0/1;0/1
chr17	47696191	47696191	C	T	TBKBP1	exonic	nonsynonymous SNV	TBKBP1:NM_014726:exon1:c.C79T:p.P27S	.	1.186	.	3.29E-05	.	Fam43_f_m_aM	0/0;0/1;0/1
chr17	47699459	47699459	G	T	TBKBP1	exonic	nonsynonymous SNV	TBKBP1:NM_014726:exon5:c.G774T:p.Q258H	.	1.105	.	2.77E-05	.	Fam85_f_m_aM_aM	0/0;0/1;0/1;0/1
chr17	47848101	47848101	G	T	SP6	exonic	nonsynonymous SNV	SP6:NM_001258248:exon2:c.C329A:p.P110Q,SP6:NM_199262:exon2:c.C329A:p.P110Q	.	1.432	.	.	6.98E-06	Fam65_f_m_aM_uF_dF	0/0;0/1;0/1;0/0;0/1
chr17	47916262	47916262	C	T	SP2	exonic	nonsynonymous SNV	SP2:NM_003110:exon3:c.C191T:p.P64L	.	1.405	0.0001	0.0001	7.68E-05	Fam120_f_m_aM_aM	0/1;0/0;0/1;0/0
chr17	47916262	47916262	C	T	SP2	exonic	nonsynonymous SNV	SP2:NM_003110:exon3:c.C191T:p.P64L	.	1.405	0.0001	0.0001	7.68E-05	Fam14_f_m_aM_aM	0/1;0/0;0/1;0/1
chr17	48058980	48058980	G	A	NFE2L1	exonic	nonsynonymous SNV	NFE2L1:NM_001330262:exon5:c.G1568A:p.R523H,NFE2L1:NM_001330261:exon6:c.G1625A:p.R542H,NFE2L1:NM_003204:exon6:c.G1658A:p.R553H	.	1.374	0.0007	0.0006	0.0002	Fam110_f_m_aM_aM_uMs	0/0;0/1;0/1;0/0;0/0
chr17	48608002	48608002	C	G	HOXB7	exonic	nonsynonymous SNV	HOXB7:NM_004502:exon2:c.G494C:p.R165P	.	1.167	.	0.0002	.	Fam22_f_m_aF_aF_uF	0/1;0/0;0/1;0/1;0/0
chr17	48623124	48623124	C	T	HOXB9	exonic	nonsynonymous SNV	HOXB9:NM_024017:exon2:c.G529A:p.A177T	.	1.032	0.0001	0.0003	0.0001	Fam85_f_m_aM_aM	0/0;0/1;0/1;0/1

chr17	48912894	48912894	G	A	UBE2Z	exonic	nonsynonymous SNV	UBE2Z:NM_023079:exon3:c.G451A:p.V151M	.	1.589	.		.	.	Fam65_f_m_aM_uF_dF	0/1;0/0;0/1;0/1;0/0
chr17	49512954	49512954	G	A	NGFR	exonic	nonsynonymous SNV	NGFR:NM_002507:exon6:c.G1229A:p.R410Q	.	1.583	.		6.72E-05	2.79E-05	Fam80_f_m_aM_uM	0/0;0/1;0/1;0/0
chr17	49792035	49792035	-	A	KAT7	splicing	.	.	0.275	.		7.36E-05	0.0003	5.59E-05	Fam70_f_m_aM	0/0;0/1;0/1
chr17	50068222	50068222	C	T	ITGA3	exonic	nonsynonymous SNV	ITGA3:NM_002204:exon4:c.C581T:p.T194M	.	1.131	.		9.83E-05	1.40E-05	Fam84_f_m_aF	0/1;0/0;0/1
chr17	50191856	50191856	C	T	COL1A1	exonic	nonsynonymous SNV	COL1A1:NM_000088:exon31:c.G2059A:p.V687M	.	1.494	.		3.55E-05	.	Fam35_f_m_aF_uM	0/1;0/0;0/1;0/1
chr17	50356622	50356622	G	A	XYLT2	exonic	nonsynonymous SNV	XYLT2:NM_022167:exon8:c.G1594A:p.A532T	.	1.133	.		.	.	Fam97_f_m_aM_aF	0/1;0/0;0/1;0/1
chr17	50468549	50468549	C	G	CHAD	exonic	nonsynonymous SNV	CHAD:NM_001267:exon1:c.G265C:p.E89Q	.	1.2	.		6.55E-05	.	Fam91_m_aM_dM_aM_dM	0/0;0/1;0/1;0/1;0/0
chr17	50569291	50569291	A	T	CACNA1G	exonic	nonsynonymous SNV	CACNA1G:NM_001256324:exon3:c.A481T:p.I161F,CACNA1G:NM_001256325:exon3:c.A481T:p.I161F,CACNA1G:NM_001256326:exon3:c.A481T:p.I161F,CACNA1G:NM_001256327:exon3:c.A481T:p.I161F,CACNA1G:NM_001256328:exon3:c.A481T:p.I161F,CACNA1G:NM_001256329:exon3:c.A481T:p.I161F,CACNA1G:NM_001256330:exon3:c.A481T:p.I161F,CACNA1G:NM_001256331:exon3:c.A481T:p.I161F,CACNA1G:NM_001256332:exon3:c.A481T:p.I161F,CACNA1G:NM_001256333:exon3:c.A481T:p.I161F,CACNA1G:NM_001256334:exon3:c.A481T:p.I161F,CACNA1G:NM_001256359:exon3:c.A481T:p.I161F,CACNA1G:NM_001256360:exon3:c.A481T:p.I161F,CACNA1G:NM_001256361:exon3:c.A481T:p.I161F,CACNA1G:NM_018896:exon3:c.A481T:p.I161F,CACNA1G:NM_198376:exon3:c.A481T:p.I161F,CACNA1G:NM_198377:exon3:c.A481T:p.I161F,CACNA1G:NM_198378:exon3:c.A481T:p.I161F,CACNA1G:NM_198379:exon3:c.A481T:p.I161F,CACNA1G:NM_198380:exon3:c.A481T:p.I161F,CACNA1G:NM_198382:exon3:c.A481T:p.I161F,CACNA1G:NM_198383:exon3:c.A481T:p.I161F,CACNA1G:NM_198384:exon3:c.A481T:p.I161F,CACNA1G:NM_198385:exon3:c.A481T:p.I161F,CACNA1G:NM_198386:exon3:c.A481T:p.I161F,CACNA1G:NM_198387:exon3:c.A481T:p.I161F,CACNA1G:NM_198388:exon3:c.A481T:p.I161F,CACNA1G:NM_198396:exon3:c.A481T:p.I161F	.	2.84	.		3.37E-05	.	Fam48_f_m_aM_uM	0/1;0/0;0/1;0/0
chr17	58206479	58206479	A	C	MKS1	exonic	nonsynonymous SNV	MKS1:NM_001321268:exon15:c.T867G:p.C289W,MKS1:NM_001165927:exon16:c.T1446G:p.C482W,MKS1:NM_017777:exon16:c.T1476G:p.C492W	.	1.052	.		4.49E-05	2.79E-05	Fam83_f_m_aF	0/0;0/1;0/1
chr17	58272772	58272772	G	A	MPO	exonic	nonsynonymous SNV	MPO:NM_000250:exon10:c.C1768T:p.R590C	.	1.323		7.35E-05	0.0001	9.07E-05	Fam9_f_m_aM_dM_uF	0/1;0/0;0/1;0/0;0/0
chr17	58358335	58358335	T	C	RNF43	exonic	nonsynonymous SNV	RNF43:NM_001305545:exon8:c.A1060G:p.N354D,RNF43:NM_001305544:exon9:c.A1441G:p.N481D,RNF43:NM_017763:exon9:c.A1441G:p.N481D	.	1.324	.		.	.	Fam53_f_m_aM	0/0;0/1;0/1
chr17	58511501	58511501	C	T	MTMR4	exonic	nonsynonymous SNV	MTMR4:NM_004687:exon5:c.G221A:p.R74Q	.	1.691		7.35E-05	0.0002	5.58E-05	Fam39_f_m_aM	0/1;0/0;0/1
chr17	58521077	58521077	G	A	SEPTIN4	exonic	nonsynonymous SNV	SEPTIN4:NM_001363803:exon9:c.C901T:p.R301W,SEPTIN4:NM_001198713:exon10:c.C1174T:p.R392W,SEPTIN4:NM_001256822:exon10:c.C757T:p.R253W,SEPTIN4:NM_004574:exon10:c.C1198T:p.R400W,SEPTIN4:NM_080416:exon10:c.C1141T:p.R381W,SEPTIN4:NM_001256782:exon11:c.C1243T:p.R415W,SEPTIN4:NM_001368771:exon12:c.C2752T:p.R918W,SEPTIN4:NM_001368772:exon12:c.C1177T:p.R393W	.	1.244		0.0001	0.0003	0.0002	Fam78_f_m_aF_uM	0/1;0/0;0/1;0/0
chr17	58955677	58955677	T	G	PPM1E	exonic	nonsynonymous SNV	PPM1E:NM_014906:exon2:c.T493G:p.L165V	.	1.854	.		.	.	Fam24_f_m_aM_aM	0/1;0/0;0/1;0/1
chr17	59605560	59605560	T	G	DHX40	exonic	nonsynonymous SNV	DHX40:NM_001166301:exon16:c.T1855G:p.L619V,DHX40:NM_024612:exon17:c.T2086G:p.L696V	.	1.435	.		.	.	Fam55_f_m_aM_aM_dM	0/1;0/0;0/0;0/1;0/0
chr17	59765035	59765035	C	T	VMP1	exonic	nonsynonymous SNV	VMP1:NM_001329400:exon4:c.C77T:p.P26L,VMP1:NM_001329396:exon5:c.C368T:p.P123L,VMP1:NM_001329398:exon5:c.C188T:p.P63L,VMP1:NM_001329399:exon5:c.C188T:p.P63L,VMP1:NM_001329394:exon6:c.C479T:p.P160L,VMP1:NM_001329395:exon6:c.C479T:p.P160L,VMP1:NM_030938:exon6:c.C479T:p.P160L	.	1.665	.		.	.	Fam94_f_m_aM	0/0;0/1;0/1
chr17	60301646	60301646	A	G	USP32	exonic	nonsynonymous SNV	USP32:NM_032582:exon3:c.T245C:p.V82A	.	1.001		7.34E-05	0.0002	6.98E-06	Fam77_f_m_aM_aF_uM_uFs	0/0;0/1;0/0;0/1;0/0;0/1
chr17	60466371	60466371	T	C	APPBP2	exonic	nonsynonymous SNV	APPBP2:NM_001282476:exon4:c.A379G:p.K127E,APPBP2:NM_006380:exon5:c.A592G:p.K198E	.	1.165	.		0.0007	6.98E-06	Fam22_f_m_aF_aF_uF	0/0;0/1;0/1;0/1;0/1
chr17	60600542	60600542	G	A	PPM1D	exonic	nonsynonymous SNV	PPM1D:NM_003620:exon1:c.G128A:p.R43Q	.	1.364		0.0001	0.0003	7.68E-05	Fam102_f_m_aM	0/1;0/0;0/1
chr17	60600542	60600542	G	A	PPM1D	exonic	nonsynonymous SNV	PPM1D:NM_003620:exon1:c.G128A:p.R43Q	.	1.364		0.0001	0.0003	7.68E-05	Fam70_f_m_aM	0/1;0/0;0/1
chr17	63418356	63418356	C	G	TANC2	exonic	nonsynonymous SNV	TANC2:NM_025185:exon25:c.C3965G:p.P1322R	.	1.021	.		.	.	Fam102_f_m_aM	0/0;0/1;0/1
chr17	63706670	63706670	C	T	STRADA	exonic	nonsynonymous SNV	STRADA:NM_001003786:exon8:c.G712A:p.G238S,STRADA:NM_001165969:exon8:c.G736A:p.G246S,STRADA:NM_001165970:exon8:c.G691A:p.G231S,STRADA:NM_001363787:exon8:c.G736A:p.G246S,STRADA:NM_001363789:exon8:c.G712A:p.G238S,STRADA:NM_153335:exon8:c.G712A:p.G238S,STRADA:NM_001003788:exon9:c.G649A:p.G217S,STRADA:NM_001363790:exon9:c.G649A:p.G217S,STRADA:NM_001363791:exon9:c.G649A:p.G217S,STRADA:NM_001003787:exon10:c.G823A:p.G275S,STRADA:NM_001363786:exon10:c.G799A:p.G267S,STRADA:NM_001363788:exon10:c.G823A:p.G275S	.	1.283	.		6.54E-05	.	Fam118_f_m_aM_aF_uM	0/0;0/1;0/0;0/1;0/1

chr17	63707293	63707293	T	C	STRADA	exonic	nonsynonymous SNV	STRADA:NM_001003786:exon7:c.A596G:p.Y199C,STRADA:NM_001165969:exon7:c.A620G:p.Y207C,STRADA:NM_001165970:exon7:c.A575G:p.Y192C,STRADA:NM_001363787:exon7:c.A620G:p.Y207C,STRADA:NM_001363789:exon7:c.A596G:p.Y199C,STRADA:NM_153335:exon7:c.A596G:p.Y199C,STRADA:NM_001003788:exon8:c.A533G:p.Y178C,STRADA:NM_001363790:exon8:c.A533G:p.Y178C,STRADA:NM_001363791:exon8:c.A533G:p.Y178C,STRADA:NM_001003787:exon9:c.A707G:p.Y236C,STRADA:NM_001363786:exon9:c.A683G:p.Y228C,STRADA:NM_001363788:exon9:c.A707G:p.Y236C	.	1.412	.	1.12E-05	.	Fam7_f_m_aM_aM_uM	0/0;0/1;0/1;0/1;0/1
chr17	64188359	64188359	G	A	TEX2	exonic	nonsynonymous SNV	TEX2:NM_001288732:exon5:c.C2233T:p.R745C,TEX2:NM_001288733:exon5:c.C2233T:p.R745C,TEX2:NM_018469:exon5:c.C2254T:p.R752C	.	1.119	.	0.0002	8.38E-05	Fam12_f_m_aM_uM_aM	0/0;0/1;0/1;0/0;0/1
chr17	67160319	67160319	T	G	HELZ	exonic	nonsynonymous SNV	HELZ:NM_001330447:exon17:c.A2122C:p.I708L,HELZ:NM_014877:exon17:c.A2119C:p.I707L	.	1.632	.	.	.	Fam34_f_m_aM_uF	0/1;0/0;0/1;0/1
chr17	67178722	67178722	T	C	HELZ	exonic	nonsynonymous SNV	HELZ:NM_001330447:exon13:c.A1367G:p.Y456C,HELZ:NM_014877:exon13:c.A1367G:p.Y456C	.	1.854	.	6.47E-05	2.09E-05	Fam27_f_m_aF_uM_uM	0/0;0/1;0/1;0/1;0/1
chr17	67342248	67342248	C	T	PSMD12	exonic	nonsynonymous SNV	PSMD12:NM_174871:exon9:c.G1039A:p.A347T,PSMD12:NM_002816:exon10:c.G1099A:p.A367T,PSMD12:NM_001316341:exon12:c.G922A:p.A308T	.	1.545	7.34E-05	6.18E-05	3.49E-05	Fam78_f_m_aF_uM	0/1;0/0;0/1;0/0
chr17	68274038	68274038	C	T	SLC16A6	exonic	nonsynonymous SNV	SLC16A6:NM_004694:exon3:c.G265A:p.G89R,SLC16A6:NM_001174166:exon4:c.G265A:p.G89R	.	1.458	0.0004	0.0004	0.0002	Fam27_f_m_aF_uM_uM	0/0;0/1;0/1;0/1;0/1
chr17	68274038	68274038	C	T	SLC16A6	exonic	nonsynonymous SNV	SLC16A6:NM_004694:exon3:c.G265A:p.G89R,SLC16A6:NM_001174166:exon4:c.G265A:p.G89R	.	1.458	0.0004	0.0004	0.0002	Fam7_f_m_aM_aM_uM	0/0;0/1;0/1;0/1;0/1
chr17	68343669	68343669	G	A	ARSG	exonic	nonsynonymous SNV	ARSG:NM_001267727:exon3:c.G284A:p.R95Q,ARSG:NM_001352899:exon3:c.G284A:p.R95Q,ARSG:NM_001352900:exon3:c.G284A:p.R95Q,ARSG:NM_001352901:exon3:c.G284A:p.R95Q,ARSG:NM_001352902:exon3:c.G284A:p.R95Q,ARSG:NM_001352903:exon3:c.G284A:p.R95Q,ARSG:NM_001352904:exon3:c.G284A:p.R95Q,ARSG:NM_001352905:exon3:c.G284A:p.R95Q,ARSG:NM_001352906:exon3:c.G284A:p.R95Q,ARSG:NM_001352907:exon3:c.G284A:p.R95Q,ARSG:NM_001352909:exon3:c.G284A:p.R95Q,ARSG:NM_001352910:exon3:c.G284A:p.R95Q,ARSG:NM_014960:exon3:c.G284A:p.R95Q	.	1.148	7.35E-05	1.12E-05	2.79E-05	Fam35_f_m_aF_uM	0/1;0/0;0/1;0/1
chr17	69519349	69519349	C	T	MAP2K6	exonic	nonsynonymous SNV	MAP2K6:NM_001330450:exon5:c.C115T:p.R39W,MAP2K6:NM_002758:exon5:c.C283T:p.R95W	.	2.236	7.34E-05	7.47E-05	2.79E-05	Fam6_f_m_aM	0/0;0/1;0/1
chr17	72122721	72122721	T	A	SOX9	exonic	nonsynonymous SNV	SOX9:NM_000346:exon2:c.T434A:p.L145H	.	2.212	.	1.13E-05	.	Fam87_f_m_aM_uM	0/1;0/0;0/1;0/0
chr17	73424094	73424094	T	A	SDK2	splicing	.	.	0.555	.	.	.	.	Fam101_f_m_aM	0/1;0/0;0/1
chr17	75321750	75321750	T	C	GRB2	exonic	nonsynonymous SNV	GRB2:NM_203506:exon4:c.A254G:p.N85S,GRB2:NM_002086:exon5:c.A377G:p.N126S	.	1.073	.	0.0002	.	Fam92_f_m_aM_raM	0/0;0/0;0/0;0/1
chr17	75321750	75321750	T	C	GRB2	exonic	nonsynonymous SNV	GRB2:NM_203506:exon4:c.A254G:p.N85S,GRB2:NM_002086:exon5:c.A377G:p.N126S	.	1.073	.	0.0002	.	Fam91_m_aM_dM_aM_dM	0/1;0/0;0/0;0/1;0/0
chr17	75493717	75493717	C	G	TMEM94	exonic	nonsynonymous SNV	TMEM94:NM_001321148:exon17:c.C2238G:p.F746L,TMEM94:NM_001351203:exon17:c.C2238G:p.F746L,TMEM94:NM_001321149:exon18:c.C2220G:p.F740L,TMEM94:NM_001351202:exon18:c.C2160G:p.F720L,TMEM94:NM_014738:exon18:c.C2208G:p.F736L	.	1.304	.	3.35E-05	2.09E-05	Fam98_f_m_aM	0/0;0/1;0/1
chr17	75496430	75496430	C	T	TMEM94	exonic	nonsynonymous SNV	TMEM94:NM_001321148:exon23:c.C3232T:p.R1078C,TMEM94:NM_001351203:exon23:c.C3229T:p.R1077C,TMEM94:NM_001321149:exon24:c.C3214T:p.R1072C,TMEM94:NM_001351202:exon24:c.C3154T:p.R1052C,TMEM94:NM_014738:exon24:c.C3202T:p.R1068C	.	1.199	.	6.20E-05	2.79E-05	Fam101_f_m_aM	0/0;0/0;0/1
chr17	75507123	75507123	C	T	CASKIN2	exonic	nonsynonymous SNV	CASKIN2:NM_001142643:exon4:c.G5A:p.R2H,CASKIN2:NM_020753:exon5:c.G251A:p.R84H	.	1.707	7.36E-05	9.97E-05	6.98E-06	Fam103_f_m_aM	0/1;0/0;0/1
chr17	75742587	75742587	C	T	ITGB4	exonic	nonsynonymous SNV	ITGB4:NM_001005619:exon24:c.C2788T:p.R930W,ITGB4:NM_000213:exon25:c.C2788T:p.R930W,ITGB4:NM_001005731:exon25:c.C2788T:p.R930W,ITGB4:NM_001321123:exon25:c.C2788T:p.R930W	.	1.002	.	0.0001	4.19E-05	Fam31_f_m_aM_uF	0/0;0/1;0/1;0/0
chr17	75754594	75754594	G	A	ITGB4	exonic	nonsynonymous SNV	ITGB4:NM_001005619:exon32:c.G4127A:p.R1376Q,ITGB4:NM_001005731:exon33:c.G4127A:p.R1376Q,ITGB4:NM_001321123:exon33:c.G4127A:p.R1376Q,ITGB4:NM_000213:exon34:c.G4337A:p.R1446Q	.	1.084	0.0001	7.89E-05	4.19E-05	Fam103_f_m_aM	0/0;0/1;0/1
chr17	75955858	75955858	G	A	ACOX1	exonic	nonsynonymous SNV	ACOX1:NM_001185039:exon5:c.C514T:p.R172C,ACOX1:NM_004035:exon5:c.C628T:p.R210C,ACOX1:NM_007292:exon5:c.C628T:p.R210C	.	1.942	7.34E-05	0.0001	6.99E-05	Fam17_f_m_aM_uM	0/1;0/0;0/1;0/1
chr17	75960313	75960313	A	G	ACOX1	exonic	nonsynonymous SNV	ACOX1:NM_001185039:exon3:c.T218C:p.L73S,ACOX1:NM_004035:exon3:c.T332C:p.L111S	.	1.901	.	.	.	Fam64_f_m_aM	0/1;0/0;0/1
chr17	75979058	75979058	G	A	ACOX1	exonic	nonsynonymous SNV	ACOX1:NM_004035:exon1:c.C16T:p.R6C,ACOX1:NM_007292:exon1:c.C16T:p.R6C	.	1.307	.	0.0002	7.68E-05	Fam24_f_m_aM_aM	0/1;0/0;0/0;0/1
chr17	75979058	75979058	G	A	ACOX1	exonic	nonsynonymous SNV	ACOX1:NM_004035:exon1:c.C16T:p.R6C,ACOX1:NM_007292:exon1:c.C16T:p.R6C	.	1.307	.	0.0002	7.68E-05	Fam114_f_m_aM	0/1;0/0;0/1
chr17	76002371	76002371	G	A	CDK3	exonic	nonsynonymous SNV	CDK3:NM_001258:exon5:c.G439A:p.G147S	.	1.221	0.0006	3.28E-05	7.00E-06	Fam113_f_m_aF_raM_uF	0/0;0/0;0/0;0/1;0/0
chr17	76070403	76070403	G	A	SRP68	exonic	nonsynonymous SNV	SRP68:NM_001260502:exon2:c.C226T:p.R76W,SRP68:NM_014230:exon2:c.C226T:p.R76W	.	1.525	.	6.17E-05	.	Fam17_f_m_aM_uM	0/0;0/1;0/1;0/1
chr17	76313853	76313853	C	T	PRPSAP1	exonic	nonsynonymous SNV	PRPSAP1:NM_001330503:exon7:c.G511A:p.G171R,PRPSAP1:NM_001366236:exon8:c.G511A:p.G171R,PRPSAP1:NM_002766:exon8:c.G820A:p.G274R	.	1.298	.	3.35E-05	6.98E-06	Fam70_f_m_aM	0/1;0/0;0/1
chr17	76391541	76391541	T	C	UBE2O	exonic	nonsynonymous SNV	UBE2O:NM_022066:exon18:c.A3281G:p.Y1094C	.	1.266	.	.	.	Fam37_f_m_aF_uM	0/1;0/0;0/1;0/0

chr17	76926616	76926616	G	A	MGAT5B	exonic	nonsynonymous SNV	MGAT5B:NM_198955:exon9:c.G1210A:p.D404N,MGAT5B:NM_001199172:exon10:c.G1177A:p.D393N,MGAT5B:NM_144677:exon10:c.G1177A:p.D393N	.	2.032	.	.	6.98E-06	Fam15_f_m_aM_aM	0/0;0/0;0/1;0/0
chr17	76940526	76940526	G	A	MGAT5B	exonic	nonsynonymous SNV	MGAT5B:NM_198955:exon12:c.G1736A:p.R579Q,MGAT5B:NM_001199172:exon13:c.G1709A:p.R570Q,MGAT5B:NM_144677:exon13:c.G1703A:p.R568Q	.	2.136	.	9.87E-05	6.98E-06	Fam42_f_m_aM_uF	0/1;0/0;0/1;0/1
chr17	77190846	77190846	T	C	SEC14L1	exonic	nonsynonymous SNV	SEC14L1:NM_001144001:exon2:c.T5C:p.F2S,SEC14L1:NM_001039573:exon4:c.T107C:p.F36S,SEC14L1:NM_001143998:exon4:c.T107C:p.F36S,SEC14L1:NM_001143999:exon4:c.T107C:p.F36S,SEC14L1:NM_003003:exon4:c.T107C:p.F36S,SEC14L1:NM_001204410:exon5:c.T107C:p.F36S,SEC14L1:NM_001204408:exon6:c.T107C:p.F36S	.	1.905	.	.	.	Fam54_f_m_aM_uF	0/0;0/1;0/1;0/1
chr17	77190924	77190924	A	T	SEC14L1	exonic	nonsynonymous SNV	SEC14L1:NM_001144001:exon2:c.A83T:p.D28V,SEC14L1:NM_001039573:exon4:c.A185T:p.D62V,SEC14L1:NM_001143998:exon4:c.A185T:p.D62V,SEC14L1:NM_001143999:exon4:c.A185T:p.D62V,SEC14L1:NM_003003:exon4:c.A185T:p.D62V,SEC14L1:NM_001204410:exon5:c.A185T:p.D62V,SEC14L1:NM_001204408:exon6:c.A185T:p.D62V	.	1.786	.	2.23E-05	6.98E-06	Fam113_f_m_aF_raM_uF	0/0;0/0;0/0;0/1;0/0
chr17	77488806	77488806	C	T	SEPTIN9	exonic	nonsynonymous SNV	SEPTIN9:NM_001113495:exon5:c.C868T:p.R290C,SEPTIN9:NM_001113496:exon5:c.C451T:p.R151C,SEPTIN9:NM_001293696:exon5:c.C532T:p.R178C,SEPTIN9:NM_001293697:exon5:c.C451T:p.R151C,SEPTIN9:NM_001293698:exon5:c.C451T:p.R151C,SEPTIN9:NM_001113493:exon6:c.C1183T:p.R395C,SEPTIN9:NM_001113494:exon6:c.C712T:p.R238C,SEPTIN9:NM_001293695:exon6:c.C1147T:p.R383C,SEPTIN9:NM_006640:exon6:c.C1150T:p.R384C,SEPTIN9:NM_001113491:exon7:c.C1204T:p.R402C,SEPTIN9:NM_001113492:exon7:c.C712T:p.R238C	.	1.82	.	7.51E-05	2.79E-05	Fam101_f_m_aM	0/0;0/1;0/1
chr17	78049486	78049486	G	A	TNRC6C	exonic	nonsynonymous SNV	TNRC6C:NM_001142640:exon4:c.G424A:p.G142R,TNRC6C:NM_018996:exon4:c.G424A:p.G142R	.	1.061	0.0007	0.0008	0.0003	Fam80_f_m_aM_uM	0/0;0/1;0/1;0/1
chr17	78049486	78049486	G	A	TNRC6C	exonic	nonsynonymous SNV	TNRC6C:NM_001142640:exon4:c.G424A:p.G142R,TNRC6C:NM_018996:exon4:c.G424A:p.G142R	.	1.061	0.0007	0.0008	0.0003	Fam38_f_m_aM	0/1;0/0;0/1
chr17	79047988	79047988	G	A	C1QTNF1	exonic	nonsynonymous SNV	C1QTNF1:NM_198594:exon3:c.G500A:p.R167H,C1QTNF1:NM_030968:exon4:c.G746A:p.R249H,C1QTNF1:NM_153372:exon4:c.G746A:p.R249H,C1QTNF1:NM_198593:exon4:c.G746A:p.R249H	.	1.094	0.0004	0.0004	0.0003	Fam19_f_m_aM	0/1;0/0;0/1
chr17	80332437	80332437	C	A	RNF213	exonic	nonsynonymous SNV	RNF213:NM_001256071:exon21:c.C3949A:p.L1317M	.	1.059	7.34E-05	0.0002	7.68E-05	Fam42_f_m_aM_uF	0/0;0/1;0/1;0/0
chr17	80345918	80345918	G	A	RNF213	exonic	nonsynonymous SNV	RNF213:NM_001256071:exon29:c.G7583A:p.R2528Q	.	1.161	0.0003	0.0006	0.0001	Fam115_f_m_aF_aM_aF	0/1;0/0;0/1;0/0;0/0
chr17	80347189	80347189	G	A	RNF213	exonic	nonsynonymous SNV	RNF213:NM_001256071:exon29:c.G8854A:p.G2952R	.	1.185	0.0001	7.47E-05	4.89E-05	Fam88_f_m_aF	0/1;0/0;0/1
chr17	80473250	80473250	C	G	NPTX1	exonic	nonsynonymous SNV	NPTX1:NM_002522:exon3:c.G847C:p.V283L	.	1.576	0.0001	6.56E-05	8.37E-05	Fam70_f_m_aM	0/1;0/0;0/1
chr17	81715482	81715482	C	A	SLC25A10	exonic	nonsynonymous SNV	SLC25A10:NM_001270888:exon3:c.C218A:p.T73N,SLC25A10:NM_001270953:exon3:c.C218A:p.T73N,SLC25A10:NM_012140:exon3:c.C218A:p.T73N	.	1.121	.	.	.	Fam6_f_m_aM	0/0;0/1;0/1
chr17	81869353	81869353	A	G	ARHGDIA	exonic	nonsynonymous SNV	ARHGDIA:NM_001301243:exon3:c.T463C:p.Y155H,ARHGDIA:NM_001185077:exon4:c.T328C:p.Y110H,ARHGDIA:NM_001185078:exon4:c.T328C:p.Y110H,ARHGDIA:NM_001301240:exon4:c.T328C:p.Y110H,ARHGDIA:NM_001301241:exon4:c.T328C:p.Y110H,ARHGDIA:NM_001301242:exon4:c.T328C:p.Y110H,ARHGDIA:NM_004309:exon4:c.T328C:p.Y110H	.	1.93	.	.	.	Fam6_f_m_aM	0/0;0/1;0/1
chr17	81922891	81922891	C	T	MAFG	exonic	nonsynonymous SNV	MAFG:NM_002359:exon3:c.G203A:p.C68Y,MAFG:NM_032711:exon3:c.G203A:p.C68Y	.	2.504	.	.	.	Fam61_f_m_aM	0/0;0/1;0/1
chr17	81956919	81956919	G	A	NOTUM	exonic	nonsynonymous SNV	NOTUM:NM_178493:exon7:c.C851T:p.T284M	.	1.113	0.0003	0.0001	0.0001	Fam106_f_m_aM	0/1;0/0;0/1
chr17	81960825	81960825	G	C	NOTUM	exonic	nonsynonymous SNV	NOTUM:NM_178493:exon1:c.C85G:p.R29G	.	1.503	.	2.43E-05	1.40E-05	Fam83_f_m_aF	0/0;0/1;0/1
chr17	81960825	81960825	G	C	NOTUM	exonic	nonsynonymous SNV	NOTUM:NM_178493:exon1:c.C85G:p.R29G	.	1.503	.	2.43E-05	1.40E-05	Fam113_f_m_aF_raM_uF	0/0;0/1;0/1;0/0;0/0
chr17	82050717	82050717	C	G	RFNG	exonic	nonsynonymous SNV	RFNG:NM_002917:exon3:c.G364C:p.A122P	.	1.226	.	.	.	Fam97_f_m_aM_aF	0/1;0/0;0/0;0/1
chr17	82418479	82418479	G	A	OGFOD3	exonic	nonsynonymous SNV	OGFOD3:NM_024648:exon1:c.C7T:p.P3S,OGFOD3:NM_175902:exon1:c.C7T:p.P3S	.	1.725	0.0002	0.0002	9.11E-05	Fam45_f_m_aM_uF	0/0;0/1;0/1;0/0
chr18	3457467	3457467	A	G	TGIF1	exonic	nonsynonymous SNV	TGIF1:NM_001278682:exon3:c.A355G:p.K119E,TGIF1:NM_003244:exon3:c.A346G:p.K116E,TGIF1:NM_170695:exon3:c.A733G:p.K245E,TGIF1:NM_173207:exon3:c.A388G:p.K130E,TGIF1:NM_173209:exon3:c.A286G:p.K96E,TGIF1:NM_173210:exon3:c.A286G:p.K96E,TGIF1:NM_173211:exon3:c.A286G:p.K96E,TGIF1:NM_001278684:exon4:c.A346G:p.K116E,TGIF1:NM_001278686:exon4:c.A286G:p.K96E,TGIF1:NM_173208:exon4:c.A346G:p.K116E,TGIF1:NM_174886:exon4:c.A286G:p.K96E	.	1.29	.	1.12E-05	.	Fam117_f_m_aM_aF	0/0;0/1;0/1;0/0
chr18	8380398	8380398	G	A	PTPRM	exonic	nonsynonymous SNV	PTPRM:NM_002845:exon27:c.G3850A:p.V1284I,PTPRM:NM_001105244:exon29:c.G3889A:p.V1297I	.	1.1	.	0.0003	.	Fam116_f_m_aM_aF	0/0;0/1;0/1;0/0
chr18	10715717	10715717	C	T	PIEZO2	exonic	nonsynonymous SNV	PIEZO2:NM_022068:exon35:c.G5015A:p.R1672K	.	1.016	.	.	.	Fam63_f_m_aF	0/1;0/0;0/1
chr18	10784824	10784824	G	A	PIEZO2	exonic	nonsynonymous SNV	PIEZO2:NM_022068:exon17:c.C2452T:p.L818F	.	1.064	.	.	.	Fam34_f_m_aM_uF	0/1;0/0;0/1;0/1
chr18	11851548	11851548	T	A	CHMP1B	exonic	nonsynonymous SNV	CHMP1B:NM_020412:exon1:c.T37A:p.F13I	.	1.059	7.34E-05	4.65E-05	4.88E-05	Fam87_f_m_aM_uM	0/0;0/1;0/1;0/0
chr18	11851819	11851819	T	G	CHMP1B	exonic	nonsynonymous SNV	CHMP1B:NM_020412:exon1:c.T308G:p.L103W	.	1.995	.	.	.	Fam80_f_m_aM_uM	0/0;0/1;0/1;0/1
chr18	12308299	12308299	G	C	TUBB6	exonic	nonsynonymous SNV	TUBB6:NM_001303525:exon1:c.G7C:p.E3Q,TUBB6:NM_001303526:exon1:c.G7C:p.E3Q,TUBB6:NM_032525:exon1:c.G7C:p.E3Q,TUBB6:NM_001303524:exon2:c.G7C:p.E3Q	.	2.091	.	.	7.03E-06	Fam87_f_m_aM_uM	0/1;0/0;0/1;0/1

chr18	12358723	12358723	T	G	AFG3L2	exonic	nonsynonymous SNV	AFG3L2:NM_006796:exon8:c.A973C:p.N325H	.	2.051	.	1.12E-05	6.98E-06	Fam48_f_m_aM_uM	0/0;0/1;0/1;0/1
chr18	23234645	23234645	G	A	CABLES1	exonic	nonsynonymous SNV	CABLES1:NM_001100619:exon5:c.G1126A:p.A376T,CABLES1:NM_001256438:exon5:c.G145A:p.A49T,CABLES1:NM_138375:exon5:c.G331A:p.A111T	.	1.238	.	.	.	Fam62_f_m_aM_aM	0/0;0/1;0/1;0/0
chr18	23524453	23524453	A	T	RMC1	exonic	nonsynonymous SNV	RMC1:NM_001318707:exon9:c.A560T:p.Q187L,RMC1:NM_001276342:exon10:c.A887T:p.Q296L,RMC1:NM_001318709:exon10:c.A887T:p.Q296L,RMC1:NM_001318708:exon11:c.A560T:p.Q187L,RMC1:NM_013326:exon12:c.A1031T:p.Q344L	.	1.135	7.35E-05	6.70E-05	4.19E-05	Fam117_f_m_aM_aF	0/0;0/1;0/1;0/1
chr18	24123917	24123917	C	A	TTC39C	exonic	nonsynonymous SNV	TTC39C:NM_001292030:exon4:c.C349A:p.Q117K,TTC39C:NM_001135993:exon9:c.C1270A:p.Q424K,TTC39C:NM_153211:exon9:c.C1087A:p.Q363K	.	1.535	.	.	.	Fam110_f_m_aM_aM_uMs	0/0;0/1;0/0;0/1;0/0
chr18	24178089	24178089	T	C	OSBPL1A	exonic	nonsynonymous SNV	OSBPL1A:NM_018030:exon7:c.A478G:p.I160V,OSBPL1A:NM_001242508:exon9:c.A871G:p.I291V,OSBPL1A:NM_080597:exon21:c.A2017G:p.I673V	.	1.057	0.0001	0.0001	7.01E-05	Fam24_f_m_aM_aM	0/0;0/1;0/0;0/1
chr18	28013904	28013904	A	G	CDH2	exonic	nonsynonymous SNV	CDH2:NM_001308176:exon2:c.T85C:p.F29L,CDH2:NM_001792:exon3:c.T178C:p.F60L	.	1.092	.	.	.	Fam80_f_m_aM_uM	0/0;0/1;0/1;0/0
chr18	31595212	31595212	A	T	TTR	exonic	nonsynonymous SNV	TTR:NM_000371:exon3:c.A293T:p.Y98F	.	1.049	.	.	6.98E-06	Fam26_f_m_aM_uM	0/1;0/0;0/1;0/1
chr18	31645366	31645366	G	T	B4GALT6	exonic	nonsynonymous SNV	B4GALT6:NM_001330570:exon3:c.C343A:p.P115T,B4GALT6:NM_004775:exon4:c.C460A:p.P154T	.	1.631	0.0001	0.0002	0.0001	Fam45_f_m_aM_uF	0/0;0/1;0/1;0/0
chr18	32769882	32769882	G	A	KLHL14	exonic	nonsynonymous SNV	KLHL14:NM_020805:exon2:c.C710T:p.A237V	.	1.145	.	6.76E-05	5.58E-05	Fam91_m_aM_dM_aM_dM	0/0;0/1;0/1;0/0;0/1
chr18	32769906	32769906	G	C	KLHL14	exonic	nonsynonymous SNV	KLHL14:NM_020805:exon2:c.C686G:p.P229R	.	1.294	0.0003	0.0003	0.0002	Fam20_f_m_aM_uF	0/0;0/1;0/1;0/1
chr18	34875333	34875333	C	T	DTNA	exonic	nonsynonymous SNV	DTNA:NM_001198943:exon8:c.C827T:p.P276L,DTNA:NM_001198944:exon9:c.C713T:p.P238L,DTNA:NM_032980:exon9:c.C701T:p.P234L,DTNA:NM_001198942:exon11:c.C884T:p.P295L,DTNA:NM_001198938:exon16:c.C1577T:p.P526L,DTNA:NM_001198940:exon16:c.C1577T:p.P526L,DTNA:NM_001198939:exon17:c.C1598T:p.P533L,DTNA:NM_001390:exon17:c.C1757T:p.P586L,DTNA:NM_032975:exon17:c.C1586T:p.P529L	.	1.064	7.34E-05	0.0002	0.0001	Fam45_f_m_aM_uF	0/1;0/0;0/1;0/1
chr18	36067385	36067385	G	A	RPRD1A	exonic	nonsynonymous SNV	RPRD1A:NM_001303413:exon1:c.C20T:p.A7V,RPRD1A:NM_018170:exon1:c.C20T:p.A7V	.	1.568	.	3.57E-05	1.40E-05	Fam89_f_m_aM	0/0;0/1;0/1
chr18	37565592	37565592	C	T	CELF4	exonic	nonsynonymous SNV	CELF4:NM_001025087:exon1:c.G50A:p.S17N,CELF4:NM_001025088:exon1:c.G50A:p.S17N,CELF4:NM_001025089:exon1:c.G50A:p.S17N,CELF4:NM_001330603:exon1:c.G50A:p.S17N,CELF4:NM_001353695:exon1:c.G50A:p.S17N,CELF4:NM_001353696:exon1:c.G50A:p.S17N,CELF4:NM_001353697:exon1:c.G50A:p.S17N,CELF4:NM_001353698:exon1:c.G50A:p.S17N,CELF4:NM_001353699:exon1:c.G50A:p.S17N,CELF4:NM_001353700:exon1:c.G50A:p.S17N,CELF4:NM_001353701:exon1:c.G50A:p.S17N,CELF4:NM_001353702:exon1:c.G50A:p.S17N,CELF4:NM_001353703:exon1:c.G50A:p.S17N,CELF4:NM_001353705:exon1:c.G50A:p.S17N,CELF4:NM_001353706:exon1:c.G50A:p.S17N,CELF4:NM_001353707:exon1:c.G50A:p.S17N,CELF4:NM_001353708:exon1:c.G50A:p.S17N,CELF4:NM_001353709:exon1:c.G50A:p.S17N,CELF4:NM_001353710:exon1:c.G50A:p.S17N,CELF4:NM_001353711:exon1:c.G50A:p.S17N,CELF4:NM_001353712:exon1:c.G50A:p.S17N,CELF4:NM_001353713:exon1:c.G50A:p.S17N,CELF4:NM_001353714:exon1:c.G50A:p.S17N,CELF4:NM_001353715:exon1:c.G50A:p.S17N,CELF4:NM_001353716:exon1:c.G50A:p.S17N,CELF4:NM_001353717:exon1:c.G50A:p.S17N,CELF4:NM_001353718:exon1:c.G50A:p.S17N,CELF4:NM_001353719:exon1:c.G50A:p.S17N,CELF4:NM_001353720:exon1:c.G50A:p.S17N,CELF4:NM_001353721:exon1:c.G50A:p.S17N,CELF4:NM_001353722:exon1:c.G50A:p.S17N,CELF4:NM_001353723:exon1:c.G50A:p.S17N,CELF4:NM_001353724:exon1:c.G50A:p.S17N,CELF4:NM_001353725:exon1:c.G50A:p.S17N,CELF4:NM_001353726:exon1:c.G50A:p.S17N,CELF4:NM_001353727:exon1:c.G50A:p.S17N,CELF4:NM_001353728:exon1:c.G50A:p.S17N,CELF4:NM_001353729:exon1:c.G50A:p.S17N,CELF4:NM_001353730:exon1:c.G50A:p.S17N,CELF4:NM_001353731:exon1:c.G50A:p.S17N,CELF4:NM_001353732:exon1:c.G50A:p.S17N,CELF4:NM_001353733:exon1:c.G50A:p.S17N,CELF4:NM_001353734:exon1:c.G50A:p.S17N,CELF4:NM_001353735:exon1:c.G50A:p.S17N,CELF4:NM_001353736:exon1:c.G50A:p.S17N,CELF4:NM_001353737:exon1:c.G50A:p.S17N,CELF4:NM_001353738:exon1:c.G50A:p.S17N,CELF4:NM_001353739:exon1:c.G50A:p.S17N,CELF4:NM_001353740:exon1:c.G50A:p.S17N,CELF4:NM_001353741:exon1:c.G50A:p.S17N,CELF4:[illegible]	.	1.136	.	.	.	Fam88_f_m_aF	0/1;0/0;0/1
chr18	46433215	46433215	T	A	RNF165	exonic	nonsynonymous SNV	RNF165:NM_152470:exon2:c.T87A:p.H29Q	.	1.59	7.36E-05	1.22E-05	.	Fam62_f_m_aM_aM	0/0;0/1;0/1;0/1
chr18	46435303	46435303	G	A	RNF165	exonic	nonsynonymous SNV	RNF165:NM_152470:exon3:c.G392A:p.R131H	.	1.695	7.36E-05	0.0003	1.40E-05	Fam35_f_m_aF_uM	0/0;0/1;0/1;0/0

chr18	46890997	46890997	G	A	PIAS2	exonic	nonsynonymous SNV	PIAS2:NM_001324058:exon2:c.C82T:p.R28W,PIAS2:NM_001324060:exon2:c.C94T:p.R32W,PIAS2:NM_001354034:exon2:c.C82T:p.R28W,PIAS2:NM_004671:exon2:c.C82T:p.R28W,PIAS2:NM_173206:exon2:c.C82T:p.R28W,PIAS2:NM_001324047:exon3:c.C55T:p.R19W,PIAS2:NM_001324051:exon3:c.C55T:p.R19W,PIAS2:NM_001324054:exon3:c.C55T:p.R19W,PIAS2:NM_001354036:exon3:c.C55T:p.R19W,PIAS2:NM_001354037:exon3:c.C55T:p.R19W,PIAS2:NM_001324046:exon4:c.C55T:p.R19W,PIAS2:NM_001324048:exon4:c.C55T:p.R19W,PIAS2:NM_001324055:exon4:c.C55T:p.R19W,PIAS2:NM_001324059:exon4:c.C55T:p.R19W,PIAS2:NM_001354033:exon4:c.C55T:p.R19W,PIAS2:NM_001354039:exon4:c.C55T:p.R19W,PIAS2:NM_001324049:exon5:c.C55T:p.R19W,PIAS2:NM_001324052:exon5:c.C55T:p.R19W,PIAS2:NM_001324053:exon5:c.C55T:p.R19W,PIAS2:NM_001324057:exon5:c.C55T:p.R19W,PIAS2:NM_001354035:exon5:c.C55T:p.R19W,PIAS2:NM_001354038:exon5:c.C55T:p.R19W	.	1.071	.	6.40E-05	.	Fam91_m_aM_dM_aM_dM	0/0;0/0;0/0;0/1;0/0
chr18	47247568	47247568	C	T	SKOR2	exonic	nonsynonymous SNV	SKOR2:NM_001278063:exon2:c.G1616A:p.G539D	.	1.215	.	.	.	Fam36_f_m_aM_uM	0/0;0/1;0/1;0/1
chr18	47248859	47248859	G	A	SKOR2	exonic	nonsynonymous SNV	SKOR2:NM_001037802:exon2:c.C325T:p.P109S,SKOR2:NM_001278063:exon2:c.C325T:p.P109S	.	2.128	.	.	.	Fam63_f_m_aF	0/0;0/1;0/1
chr18	48040120	48040120	C	T	ZBTB7C	exonic	nonsynonymous SNV	ZBTB7C:NM_001039360:exon2:c.G988A:p.D330N,ZBTB7C:NM_001318841:exon4:c.G988A:p.D330N,ZBTB7C:NM_001371286:exon4:c.G988A:p.D330N,ZBTB7C:NM_001371288:exon4:c.G988A:p.D330N,ZBTB7C:NM_001371285:exon5:c.G988A:p.D330N,ZBTB7C:NM_001371287:exon5:c.G988A:p.D330N,ZBTB7C:NM_001371284:exon6:c.G988A:p.D330N,ZBTB7C:NM_001371291:exon6:c.G988A:p.D330N,ZBTB7C:NM_001371290:exon9:c.G988A:p.D330N	.	1.218	.	7.46E-05	1.40E-05	Fam1_f_m_aM	0/0;0/1;0/1
chr18	48040381	48040381	C	T	ZBTB7C	exonic	nonsynonymous SNV	ZBTB7C:NM_001039360:exon2:c.G727A:p.D243N,ZBTB7C:NM_001318841:exon4:c.G727A:p.D243N,ZBTB7C:NM_001371286:exon4:c.G727A:p.D243N,ZBTB7C:NM_001371288:exon4:c.G727A:p.D243N,ZBTB7C:NM_001371285:exon5:c.G727A:p.D243N,ZBTB7C:NM_001371287:exon5:c.G727A:p.D243N,ZBTB7C:NM_001371284:exon6:c.G727A:p.D243N,ZBTB7C:NM_001371291:exon6:c.G727A:p.D243N,ZBTB7C:NM_001371290:exon9:c.G727A:p.D243N	.	1.189	.	0.0002	0.0001	Fam118_f_m_aM_aF_uM	0/0;0/1;0/1;0/0;0/1
chr18	48040867	48040867	G	C	ZBTB7C	exonic	nonsynonymous SNV	ZBTB7C:NM_001039360:exon2:c.C241G:p.Q81E,ZBTB7C:NM_001318841:exon4:c.C241G:p.Q81E,ZBTB7C:NM_001371286:exon4:c.C241G:p.Q81E,ZBTB7C:NM_001371288:exon4:c.C241G:p.Q81E,ZBTB7C:NM_001371285:exon5:c.C241G:p.Q81E,ZBTB7C:NM_001371287:exon5:c.C241G:p.Q81E,ZBTB7C:NM_001371284:exon6:c.C241G:p.Q81E,ZBTB7C:NM_001371291:exon6:c.C241G:p.Q81E,ZBTB7C:NM_001371290:exon9:c.C241G:p.Q81E	.	1.147	.	.	.	Fam80_f_m_aM_uM	0/0;0/1;0/1;0/1
chr18	48950327	48950327	C	T	SMAD7	exonic	nonsynonymous SNV	SMAD7:NM_001190821:exon1:c.G98A:p.G33E,SMAD7:NM_005904:exon1:c.G98A:p.G33E	.	1.312	.	0.0002	6.99E-06	Fam95_f_m_aM_aM_uF	0/1;0/0;0/1;0/1;0/0
chr18	50274338	50274338	G	T	MBD1	exonic	nonsynonymous SNV	MBD1:NM_001204141:exon10:c.C844A:p.R282S,MBD1:NM_001204151:exon10:c.C925A:p.R309S,MBD1:NM_001323953:exon10:c.C418A:p.R140S,MBD1:NM_015845:exon10:c.C925A:p.R309S,MBD1:NM_015847:exon10:c.C847A:p.R283S,MBD1:NM_001204136:exon11:c.C994A:p.R332S,MBD1:NM_001204139:exon11:c.C994A:p.R332S,MBD1:NM_001204142:exon11:c.C994A:p.R332S,MBD1:NM_001323950:exon11:c.C991A:p.R331S,MBD1:NM_001323951:exon11:c.C994A:p.R332S,MBD1:NM_015846:exon11:c.C994A:p.R332S,MBD1:NM_001204137:exon12:c.C1069A:p.R357S,MBD1:NM_001204138:exon12:c.C1069A:p.R357S,MBD1:NM_001323942:exon12:c.C1069A:p.R357S,MBD1:NM_001323947:exon12:c.C1069A:p.R357S,MBD1:NM_001323949:exon12:c.C562A:p.R188S	.	1.334	.	2.28E-05	.	Fam113_f_m_aF_raM_uF	0/0;0/0;0/0;0/1;0/0
chr18	50282702	50282702	T	C	CXXC1	exonic	nonsynonymous SNV	CXXC1:NM_001101654:exon15:c.A1874G:p.N625S,CXXC1:NM_014593:exon15:c.A1862G:p.N621S	.	2.355	.	2.23E-05	.	Fam74_f_m_aM_uM	0/1;0/0;0/1;0/1
chr18	50285208	50285208	G	A	CXXC1	exonic	nonsynonymous SNV	CXXC1:NM_001101654:exon7:c.C706T:p.R236C,CXXC1:NM_014593:exon7:c.C706T:p.R236C	.	1.096	7.35E-05	0.0001	2.09E-05	Fam98_f_m_aM	0/0;0/1;0/1
chr18	50920554	50920554	A	G	ME2	exonic	nonsynonymous SNV	ME2:NM_001168335:exon8:c.A833G:p.D278G,ME2:NM_002396:exon8:c.A833G:p.D278G	.	1.184	.	.	.	Fam51_f_m_aM_uF	0/1;0/0;0/1;0/0
chr18	56756676	56756676	G	A	WDR7	exonic	nonsynonymous SNV	WDR7:NM_015285:exon15:c.G2083A:p.V695M,WDR7:NM_052834:exon15:c.G2083A:p.V695M	.	1.121	.	2.23E-05	4.89E-05	Fam29_f_m_aF_uM	0/0;0/1;0/1;0/1
chr18	57571470	57571470	C	T	FECH	exonic	nonsynonymous SNV	FECH:NM_000140:exon4:c.G385A:p.G129R,FECH:NM_001012515:exon4:c.G403A:p.G135R,FECH:NM_001371094:exon4:c.G385A:p.G129R,FECH:NM_001371095:exon5:c.G169A:p.G57R	.	1.34	0.0003	0.0003	9.79E-05	Fam92_f_m_aM_raM	0/0;0/1;0/1;0/0
chr18	57607247	57607247	C	A	NARS	exonic	nonsynonymous SNV	NARS:NM_004539:exon9:c.G888T:p.L296F	.	1.012	.	.	.	Fam117_f_m_aM_aF	0/1;0/0;0/1;0/1

chr18	58351045	58351045	A	T	NEDD4L	exonic	nonsynonymous SNV	NEDD4L:NM_001144970:exon16:c.A1285T:p.N429Y,NEDD4L:NM_001243960:exon16:c.A1516T:p.N506Y,NEDD4L:NM_001144966:exon17:c.A1345T:p.N449Y,NEDD4L:NM_001144969:exon17:c.A1624T:p.N542Y,NEDD4L:NM_015277:exon17:c.A1648T:p.N550Y,NEDD4L:NM_001144964:exon18:c.A1345T:p.N449Y,NEDD4L:NM_001144965:exon18:c.A1345T:p.N449Y,NEDD4L:NM_001144967:exon18:c.A1708T:p.N570Y,NEDD4L:NM_001144968:exon18:c.A1684T:p.N562Y,NEDD4L:NM_001144971:exon18:c.A1285T:p.N429Y	.	2.373	.	.	.	Fam91_m_aM_dM_aM_dM	0/1;0/1;0/1;0/0;0/0
chr18	58389182	58389182	G	C	NEDD4L	exonic	nonsynonymous SNV	NEDD4L:NM_001144970:exon26:c.G2222C:p.W741S,NEDD4L:NM_001243960:exon26:c.G2453C:p.W818S,NEDD4L:NM_001144966:exon27:c.G2282C:p.W761S,NEDD4L:NM_001144969:exon27:c.G2561C:p.W854S,NEDD4L:NM_015277:exon27:c.G2585C:p.W862S,NEDD4L:NM_001144964:exon28:c.G2282C:p.W761S,NEDD4L:NM_001144965:exon28:c.G2282C:p.W761S,NEDD4L:NM_001144967:exon28:c.G2645C:p.W882S,NEDD4L:NM_001144968:exon28:c.G2621C:p.W874S,NEDD4L:NM_001144971:exon28:c.G2222C:p.W741S	.	2.726	.	0.0003	4.88E-05	Fam97_f_m_aM_aF	0/1;0/0;0/1;0/0
chr18	58671749	58671749	C	T	MALT1	exonic	nonsynonymous SNV	MALT1:NM_006785:exon1:c.C106T:p.P36S,MALT1:NM_173844:exon1:c.C106T:p.P36S	.	1.058	.	.	.	Fam106_f_m_aM	0/0;0/1;0/1
chr18	59272434	59272434	T	G	RAX	exonic	nonsynonymous SNV	RAX:NM_013435:exon2:c.A470C:p.K157T	.	1.938	.	7.82E-05	6.98E-06	Fam50_f_m_aF	0/0;0/1;0/1
chr18	61554613	61554613	A	C	CDH20	exonic	nonsynonymous SNV	CDH20:NM_031891:exon12:c.A2324C:p.D775A	.	1.177	.	1.21E-05	6.98E-06	Fam89_f_m_aM	0/0;0/1;0/1
chr18	62716171	62716171	C	T	PHLPP1	exonic	nonsynonymous SNV	PHLPP1:NM_194449:exon1:c.C488T:p.A163V	.	1.559	.	4.88E-05	4.89E-05	Fam1_f_m_aM	0/1;0/0;0/1
chr18	62716314	62716314	A	G	PHLPP1	exonic	nonsynonymous SNV	PHLPP1:NM_194449:exon1:c.A631G:p.M211V	.	1.587	7.41E-05	4.82E-05	.	Fam56_f_m_aF_aM	0/0;0/1;0/1;0/1
chr18	76442990	76442990	C	T	ZNF516	exonic	nonsynonymous SNV	ZNF516:NM_014643:exon3:c.G65A:p.R22Q	.	1.646	.	1.16E-05	.	Fam59_f_m_aF_uM	0/0;0/1;0/1;0/0
chr18	76851911	76851911	C	A	ZNF236	exonic	nonsynonymous SNV	ZNF236:NM_001306089:exon3:c.C335A:p.A112E,ZNF236:NM_007345:exon3:c.C329A:p.A110E	.	1.711	.	.	.	Fam15_f_m_aM_aM	0/1;0/0;0/1;0/1
chr18	76925348	76925348	T	C	ZNF236	exonic	nonsynonymous SNV	ZNF236:NM_001306089:exon22:c.T3821C:p.F1274S,ZNF236:NM_007345:exon22:c.T3815C:p.F1272S	.	1.301	.	.	.	Fam102_f_m_aM	0/0;0/1;0/1
chr18	77268610	77268610	T	C	GALR1	exonic	nonsynonymous SNV	GALR1:NM_001480:exon3:c.T758C:p.V253A	.	1.33	.	4.48E-05	6.98E-05	Fam115_f_m_aF_aM_aF	0/1;0/0;0/1;0/0;0/1
chr18	78994128	78994128	G	A	SALL3	exonic	nonsynonymous SNV	SALL3:NM_171999:exon2:c.G2137A:p.G713S	.	1.216	.	3.29E-05	2.79E-05	Fam87_f_m_aM_uM	0/1;0/0;0/1;0/1
chr18	78994519	78994519	T	C	SALL3	exonic	nonsynonymous SNV	SALL3:NM_171999:exon2:c.T2528C:p.L843P	.	1.569	.	9.81E-05	8.80E-06	Fam22_f_m_aF_aF_uF	0/1;0/0;0/1;0/1;0/1
chr18	78994638	78994638	G	A	SALL3	exonic	nonsynonymous SNV	SALL3:NM_171999:exon2:c.G2647A:p.V883M	.	1.052	7.35E-05	6.63E-05	1.40E-05	Fam71_f_m_aF	0/1;0/0;0/1
chr18	79451060	79451060	G	A	NFATC1	exonic	nonsynonymous SNV	NFATC1:NM_001278673:exon4:c.G280A:p.V94I,NFATC1:NM_172388:exon4:c.G280A:p.V94I,NFATC1:NM_001278669:exon5:c.G1696A:p.V566I,NFATC1:NM_001278670:exon5:c.G1696A:p.V566I,NFATC1:NM_001278672:exon5:c.G1657A:p.V553I,NFATC1:NM_001278675:exon5:c.G1657A:p.V553I,NFATC1:NM_006162:exon5:c.G1696A:p.V566I,NFATC1:NM_172387:exon5:c.G1657A:p.V553I,NFATC1:NM_172389:exon5:c.G1657A:p.V553I,NFATC1:NM_172390:exon5:c.G1696A:p.V566I	.	1.443	0.0009	0.0004	5.58E-05	Fam91_m_aM_dM_aM_dM	0/1;0/1;0/1;0/0;0/0
chr18	80159782	80159782	C	A	PARD6G	exonic	nonsynonymous SNV	PARD6G:NM_032510:exon3:c.G1120T:p.V374F	.	1.613	.	0.0005	9.77E-05	Fam71_f_m_aF	0/1;0/0;0/1
chr18	80159857	80159857	G	C	PARD6G	exonic	nonsynonymous SNV	PARD6G:NM_032510:exon3:c.C1045G:p.L349V	.	1.887	.	.	.	Fam42_f_m_aM_uF	0/0;0/1;0/1;0/1
chr18	80160089	80160089	G	C	PARD6G	exonic	nonsynonymous SNV	PARD6G:NM_032510:exon3:c.C813G:p.D271E	.	1.11	0.0006	0.0005	0.0003	Fam70_f_m_aM	0/1;0/0;0/1
chr19	917735	917735	A	G	KISS1R	exonic	nonsynonymous SNV	KISS1R:NM_032551:exon1:c.A233G:p.N78S	.	1.319	.	3.37E-05	6.98E-06	Fam9_f_m_aM_dM_uF	0/1;0/0;0/1;0/0;0/0
chr19	1081707	1081707	G	T	ARHGAP45	exonic	nonsynonymous SNV	ARHGAP45:NM_001282334:exon8:c.G1253T:p.C418F,ARHGAP45:NM_001282335:exon17:c.G1997T:p.C666F,ARHGAP45:NM_001258328:exon18:c.G2396T:p.C799F,ARHGAP45:NM_001321232:exon18:c.G2360T:p.C787F,ARHGAP45:NM_012292:exon18:c.G2348T:p.C783F	.	1.073	.	.	.	Fam55_f_m_aM_aM_dM	0/1;0/0;0/0;0/1;0/0
chr19	1109359	1109359	T	C	SBNO2	exonic	nonsynonymous SNV	SBNO2:NM_001100122:exon26:c.A3110G:p.Y1037C,SBNO2:NM_014963:exon29:c.A3281G:p.Y1094C	.	1.302	.	.	.	Fam82_f_m_aM_uF	0/0;0/1;0/1;0/0
chr19	1453080	1453080	C	G	APC2	exonic	nonsynonymous SNV	APC2:NM_001351273:exon1:c.C79G:p.Q27E,APC2:NM_005883:exon2:c.C79G:p.Q27E	.	1.175	0.0003	.	5.58E-05	Fam7_f_m_aM_aM_uM	0/1;0/0;0/1;0/0;0/0
chr19	1460822	1460822	G	A	APC2	exonic	nonsynonymous SNV	APC2:NM_001351273:exon11:c.G1483A:p.A495T,APC2:NM_005883:exon12:c.G1486A:p.A496T	.	1.429	.	1.14E-05	1.40E-05	Fam26_f_m_aM_uM	0/0;0/1;0/1;0/0
chr19	1465155	1465155	G	T	APC2	exonic	nonsynonymous SNV	APC2:NM_001351273:exon14:c.G1851T:p.R617S,APC2:NM_005883:exon15:c.G1854T:p.R618S	.	1.609	.	.	.	Fam94_f_m_aM	0/0;0/1;0/1
chr19	1466605	1466605	G	A	APC2	exonic	nonsynonymous SNV	APC2:NM_001351273:exon14:c.G3301A:p.E1101K,APC2:NM_005883:exon15:c.G3304A:p.E1102K	.	1.306	.	1.79E-05	.	Fam110_f_m_aM_aM_uMs	0/1;0/0;0/0;0/1;0/1
chr19	1469768	1469768	A	G	APC2	exonic	nonsynonymous SNV	APC2:NM_001351273:exon14:c.A6464G:p.H2155R,APC2:NM_005883:exon15:c.A6467G:p.H2156R	.	1.664	.	.	1.49E-05	Fam110_f_m_aM_aM_uMs	0/1;0/0;0/1;0/0;0/0
chr19	2252568	2252568	C	T	JSRP1	exonic	nonsynonymous SNV	JSRP1:NM_144616:exon7:c.G757A:p.E253K	.	1.17	.	.	.	Fam44_f_m_aM_uF	0/1;0/0;0/1;0/0
chr19	2321772	2321772	C	T	LSM7	exonic	nonsynonymous SNV	LSM7:NM_016199:exon4:c.G220A:p.V74M	.	1.879	.	0.0001	6.98E-06	Fam116_f_m_aM_aF	0/0;0/1;0/1;0/1
chr19	3110232	3110232	G	T	GNA11	exonic	nonsynonymous SNV	GNA11:NM_002067:exon2:c.G220T:p.G74C	.	1.518	.	.	.	Fam12_f_m_aM_uM_aM	0/0;0/1;0/0;0/1;0/1
chr19	3207443	3207443	C	T	NCLN	exonic	nonsynonymous SNV	NCLN:NM_001321463:exon14:c.C1603T:p.L535F,NCLN:NM_020170:exon14:c.C1606T:p.L536F	.	1.477	.	.	.	Fam97_f_m_aM_aF	0/1;0/0;0/0;0/1
chr19	3531961	3531961	A	G	FZR1	exonic	nonsynonymous SNV	FZR1:NM_001136197:exon7:c.A607G:p.M203V,FZR1:NM_001136198:exon9:c.A874G:p.M292V,FZR1:NM_016263:exon10:c.A874G:p.M292V	.	1.32	0.0003	0.0007	0.0003	Fam27_f_m_aF_uM_uM	0/0;0/1;0/1;0/1;0/0
chr19	3532450	3532450	G	A	FZR1	exonic	nonsynonymous SNV	FZR1:NM_001136197:exon8:c.G775A:p.V259M,FZR1:NM_001136198:exon10:c.G1042A:p.V348M,FZR1:NM_016263:exon11:c.G1042A:p.V348M	.	1.377	.	7.63E-05	1.40E-05	Fam24_f_m_aM_aM	0/1;0/0;0/1;0/1

chr19	3612324	3612324	G	T	CACTIN	exonic	nonsynonymous SNV	CACTIN:NM_001080543:exon10:c.C1876A:p.P626T,CACTIN:NM_021231:exon10:c.C1876A:p.P626T	.	2.676	.	.	.		Fam71_f_m_aF	0/1;0/0;0/1
chr19	3908279	3908279	G	A	ATCAY	exonic	nonsynonymous SNV	ATCAY:NM_033064:exon6:c.G556A:p.E186K	.	1.358	7.37E-05	9.24E-05	4.89E-05		Fam36_f_m_aM_uM	0/1;0/0;0/1;0/0
chr19	4254468	4254468	G	C	YJU2	exonic	nonsynonymous SNV	YJU2:NM_018074:exon4:c.G384C:p.E128D	.	1.169	0.0005	0.001	0.0006		Fam13_f_m_aM	0/0;0/1;0/1
chr19	4254468	4254468	G	C	YJU2	exonic	nonsynonymous SNV	YJU2:NM_018074:exon4:c.G384C:p.E128D	.	1.169	0.0005	0.001	0.0006		Fam20_f_m_aM_uF	0/1;0/0;0/1;0/0
chr19	4343779	4343779	G	C	MPND	exonic	nonsynonymous SNV	MPND:NM_001159846:exon2:c.G79C:p.A27P,MPND:NM_001300862:exon2:c.G79C:p.A27P,MPND:NM_032868:exon2:c.G79C:p.A27P	.	2.287	.	.	.		Fam120_f_m_aM_aM	0/0;0/1;0/1;0/1
chr19	4354347	4354347	T	G	MPND	exonic	nonsynonymous SNV	MPND:NM_001159846:exon6:c.T773G:p.V258G,MPND:NM_001300862:exon6:c.T773G:p.V258G,MPND:NM_032868:exon6:c.T773G:p.V258G	.	1.153	.	.	.		Fam72_f_m_aF_uF	0/1;0/0;0/1;0/0
chr19	4363794	4363794	G	A	SH3GL1	exonic	nonsynonymous SNV	SH3GL1:NM_001199943:exon5:c.C406T:p.R136C,SH3GL1:NM_001199944:exon6:c.C358T:p.R120C,SH3GL1:NM_003025:exon6:c.C550T:p.R184C	.	1.323	.	7.46E-05	.		Fam82_f_m_aM_uF	0/0;0/1;0/1;0/0
chr19	4366976	4366976	C	T	SH3GL1	exonic	nonsynonymous SNV	SH3GL1:NM_001199943:exon2:c.G64A:p.G22R,SH3GL1:NM_001199944:exon2:c.G64A:p.G22R,SH3GL1:NM_003025:exon2:c.G64A:p.G22R	.	1.319	.	3.27E-05	6.98E-06		Fam12_f_m_aM_uM_aM	0/1;0/0;0/1;0/1;0/1
chr19	4429451	4429451	G	A	CHAF1A	exonic	nonsynonymous SNV	CHAF1A:NM_005483:exon9:c.G1618A:p.V540M	.	1.738	.	.	1.40E-05		Fam56_f_m_aF_aM	0/1;0/0;0/0;0/1
chr19	4543879	4543879	G	C	SEMA6B	exonic	nonsynonymous SNV	SEMA6B:NM_032108:exon17:c.C2389G:p.R797G	.	3.597	.	.	.		Fam100_f_m_aF	0/1;0/0;0/1
chr19	4548309	4548309	C	T	SEMA6B	exonic	nonsynonymous SNV	SEMA6B:NM_032108:exon13:c.G1408A:p.G470R	.	1.272	.	.	.		Fam118_f_m_aM_aF_uM	0/0;0/1;0/0;0/1;0/1
chr19	5604851	5604851	G	A	SAFB2	exonic	nonsynonymous SNV	SAFB2:NM_014649:exon10:c.C1382T:p.A461V	.	1.303	.	9.83E-05	4.89E-05		Fam24_f_m_aM_aM	0/0;0/1;0/0;0/1
chr19	5827860	5827860	C	A	NRTN	exonic	nonsynonymous SNV	NRTN:NM_004558:exon2:c.C281A:p.A94E	.	1.701	0.0002	0.0006	0.0002		Fam24_f_m_aM_aM	0/0;0/1;0/1;0/0
chr19	6004235	6004235	A	G	RFX2	exonic	nonsynonymous SNV	RFX2:NM_134433:exon12:c.T1391C:p.M464T,RFX2:NM_000635:exon13:c.T1466C:p.M489T	.	1.395	.	.	.		Fam114_f_m_aM	0/1;0/0;0/1
chr19	6418052	6418052	G	T	KHSRP	exonic	nonsynonymous SNV	KHSRP:NM_001366299:exon10:c.C907A:p.L303I,KHSRP:NM_001366300:exon10:c.C907A:p.L303I,KHSRP:NM_003685:exon10:c.C907A:p.L303I	.	2.337	.	.	.		Fam118_f_m_aM_aF_uM	0/0;0/1;0/1;0/0;0/0
chr19	6531050	6531050	C	G	TNFSF9	exonic	nonsynonymous SNV	TNFSF9:NM_003811:exon1:c.C14G:p.S5C	.	1.512	.	.	.		Fam7_f_m_aM_aM_uM	0/1;0/0;0/1;0/0;0/0
chr19	6714442	6714442	G	A	C3	exonic	nonsynonymous SNV	C3:NM_000064:exon5:c.C509T:p.P170L	.	1.583	.	1.12E-05	.		Fam69_f_m_aM	0/1;0/0;0/1
chr19	6826628	6826628	C	T	VAV1	exonic	nonsynonymous SNV	VAV1:NM_001258207:exon8:c.C748T:p.R250C,VAV1:NM_001258206:exon9:c.C844T:p.R282C,VAV1:NM_005428:exon9:c.C844T:p.R282C	.	2.075	7.35E-05	9.35E-05	2.79E-05		Fam52_f_m_aM	0/1;0/0;0/1
chr19	6857093	6857093	T	G	VAV1	exonic	nonsynonymous SNV	VAV1:NM_001258206:exon26:c.T2458G:p.S820A,VAV1:NM_001258207:exon26:c.T2428G:p.S810A,VAV1:NM_005428:exon27:c.T2524G:p.S842A	.	1.805	.	.	.		Fam6_f_m_aM	0/1;0/0;0/1
chr19	7125348	7125348	G	C	INSR	exonic	nonsynonymous SNV	INSR:NM_001079817:exon16:c.C3157G:p.L1053V,INSR:NM_000208:exon17:c.C3193G:p.L1065V	.	1.184	0.0005	0.0005	0.0002		Fam110_f_m_aM_aM_uMs	0/1;0/0;0/1;0/1;0/0
chr19	7126628	7126628	G	A	INSR	exonic	nonsynonymous SNV	INSR:NM_001079817:exon15:c.C2933T:p.P978L,INSR:NM_000208:exon16:c.C2969T:p.P990L	.	1.734	.	9.50E-05	.		Fam119_f_m_aM_aM	0/0;0/1;0/1;0/0
chr19	7170587	7170587	C	T	INSR	exonic	nonsynonymous SNV	INSR:NM_000208:exon6:c.G1433A:p.R478H,INSR:NM_001079817:exon6:c.G1433A:p.R478H	.	2.017	7.35E-05	0.0001	5.59E-05		Fam37_f_m_aF_uM	0/1;0/0;0/1;0/1
chr19	7615554	7615554	G	A	CAMSAP3	exonic	nonsynonymous SNV	CAMSAP3:NM_020902:exon13:c.G2947A:p.E983K,CAMSAP3:NM_001080429:exon15:c.G3028A:p.E1010K	.	1.733	7.35E-05	2.57E-05	6.98E-06		Fam45_f_m_aM_uF	0/1;0/0;0/1;0/0
chr19	7627817	7627817	G	A	XAB2	exonic	nonsynonymous SNV	XAB2:NM_020196:exon3:c.C235T:p.R79W	.	1.339	.	1.12E-05	2.09E-05		Fam2_f_m_aF	0/1;0/0;0/1
chr19	7729433	7729433	A	T	CLEC4G	exonic	nonsynonymous SNV	CLEC4G:NM_001244856:exon9:c.T779A:p.L260Q,CLEC4G:NM_198492:exon9:c.T815A:p.L272Q	.	1.409	.	.	.		Fam117_f_m_aM_aF	0/0;0/1;0/0;0/1
chr19	7767520	7767520	T	C	CLEC4M	exonic	nonsynonymous SNV	CLEC4M:NM_001144906:exon5:c.T533C:p.F178S,CLEC4M:NM_001144907:exon5:c.T740C:p.F247S,CLEC4M:NM_001144904:exon6:c.T788C:p.F263S,CLEC4M:NM_014257:exon6:c.T941C:p.F314S,CLEC4M:NM_001144905:exon7:c.T869C:p.F290S,CLEC4M:NM_001144909:exon7:c.T803C:p.F268S,CLEC4M:NM_001144910:exon7:c.T872C:p.F291S	.	1.077	.	.	.		Fam48_f_m_aM_uM	0/0;0/1;0/1;0/1
chr19	7850027	7850027	G	A	EVI5L	exonic	nonsynonymous SNV	EVI5L:NM_001159944:exon6:c.G658A:p.V220M,EVI5L:NM_145245:exon6:c.G658A:p.V220M	.	2.026	.	3.55E-05	6.99E-06		Fam72_f_m_aF_uF	0/0;0/1;0/1;0/0
chr19	7967610	7967610	G	A	ELAVL1	exonic	nonsynonymous SNV	ELAVL1:NM_001419:exon5:c.C611T:p.A204V	.	1.488	.	4.48E-05	6.98E-06		Fam76_f_m_aM_uM	0/0;0/1;0/1;0/0
chr19	8399920	8399920	G	A	RAB11B	exonic	nonsynonymous SNV	RAB11B:NM_004218:exon2:c.G98A:p.R33H	.	2.605	.	.	.		Fam52_f_m_aM	0/0;0/0;0/1
chr19	8605263	8605263	G	A	ADAMTS10	exonic	nonsynonymous SNV	ADAMTS10:NM_030957:exon4:c.C184T:p.R62W	.	1.553	.	3.30E-05	6.98E-06		Fam116_f_m_aM_aF	0/0;0/1;0/0;0/1
chr19	10223921	10223921	G	A	S1PR2	exonic	nonsynonymous SNV	S1PR2:NM_004230:exon2:c.C985T:p.R329C	.	1.675	.	6.88E-05	2.09E-05		Fam2_f_m_aF	0/1;0/0;0/1
chr19	10256820	10256820	G	A	MRPL4	exonic	nonsynonymous SNV	MRPL4:NM_015956:exon5:c.G440A:p.R147Q,MRPL4:NM_146388:exon5:c.G440A:p.R147Q,MRPL4:NM_146387:exon6:c.G440A:p.R147Q	.	1.194	0.0007	0.0001	7.07E-06		Fam27_f_m_aF_uM_uM	0/0;0/1;0/1;0/1;0/0
chr19	10335275	10335275	A	G	ICAM3	exonic	nonsynonymous SNV	ICAM3:NM_001320606:exon4:c.T497C:p.L166P,ICAM3:NM_002162:exon4:c.T728C:p.L243P	.	1.588	.	0.0001	1.40E-05		Fam30_f_m_aM_uM	0/0;0/1;0/1;0/1
chr19	10489302	10489302	C	T	KEAP1	exonic	nonsynonymous SNV	KEAP1:NM_012289:exon5:c.G1598A:p.S533N,KEAP1:NM_203500:exon5:c.G1598A:p.S533N	.	1.742	.	.	.		Fam53_f_m_aM	0/1;0/0;0/1
chr19	10499441	10499441	T	G	KEAP1	exonic	nonsynonymous SNV	KEAP1:NM_012289:exon2:c.A593C:p.E198A,KEAP1:NM_203500:exon2:c.A593C:p.E198A	.	2.191	.	3.29E-05	.		Fam40_f_m_aM_aM	0/1;0/0;0/1;0/1
chr19	10544813	10544813	G	A	ATG4D	exonic	nonsynonymous SNV	ATG4D:NM_001281504:exon2:c.G77A:p.S26N,ATG4D:NM_032885:exon2:c.G266A:p.S89N	.	1	0.0008	0.001	0.0007		Fam120_f_m_aM_aM	0/0;0/1;0/0;0/1
chr19	10871716	10871716	C	G	CARM1	exonic	nonsynonymous SNV	CARM1:NM_001370088:exon1:c.C14G:p.A5G,CARM1:NM_199141:exon1:c.C14G:p.A5G	.	1.278	.	.	.		Fam49_f_m_aM	0/1;0/0;0/1

chr19	11007921	11007921	C	T	SMARCA4	exonic	nonsynonymous SNV	SMARCA4:NM_001128845:exon13:c.C2021T:p.P674L,SMARCA4:NM_001128846:exon13:c.C2021T:p.P674L,SMARCA4:NM_001128848:exon13:c.C2021T:p.P674L,SMARCA4:NM_001128847:exon14:c.C2021T:p.P674L,SMARCA4:NM_001128849:exon14:c.C2021T:p.P674L,SMARCA4:NM_003072:exon14:c.C2021T:p.P674L,SMARCA4:NM_001128844:exon15:c.C2021T:p.P674L	.	1.275	7.36E-05	8.94E-05	6.99E-06	Fam45_f_m_aM_uF	0/0;0/1;0/1;0/0
chr19	11060110	11060110	G	A	SMARCA4	exonic	nonsynonymous SNV	SMARCA4:NM_001128845:exon32:c.G4744A:p.G1582S,SMARCA4:NM_001128846:exon32:c.G4741A:p.G1581S,SMARCA4:NM_001128848:exon32:c.G4732A:p.G1578S,SMARCA4:NM_001128847:exon33:c.G4735A:p.G1579S,SMARCA4:NM_003072:exon34:c.G4834A:p.G1612S,SMARCA4:NM_001128844:exon35:c.G4834A:p.G1612S,SMARCA4:NM_001128849:exon35:c.G4930A:p.G1644S	.	1.811	7.35E-05	0.0001	1.40E-05	Fam21_f_m_aM_uM	0/0;0/1;0/1;0/1
chr19	11380920	11380920	A	C	EPOR	exonic	nonsynonymous SNV	EPOR:NM_000121:exon6:c.T791G:p.V264G	.	1.105	0.0001	0.001	0.0001	Fam122_f_m_aM	0/1;0/0;0/1
chr19	11383229	11383229	G	A	EPOR	exonic	nonsynonymous SNV	EPOR:NM_000121:exon2:c.C119T:p.A40V	.	1.15	.	2.33E-05	2.09E-05	Fam42_f_m_aM_uF	0/0;0/1;0/1;0/0
chr19	11466710	11466710	C	T	ELAVL3	exonic	nonsynonymous SNV	ELAVL3:NM_001420:exon2:c.G127A:p.V43I,ELAVL3:NM_032281:exon2:c.G127A:p.V43I	.	1.057	.	1.12E-05	.	Fam107_f_m_aM	0/1;0/0;0/1
chr19	11505503	11505503	C	G	ZNF653	exonic	nonsynonymous SNV	ZNF653:NM_138783:exon1:c.G284C:p.R95P	.	1.327	.	.	.	Fam116_f_m_aM_aF	0/0;0/1;0/1;0/0
chr19	11576580	11576580	G	C	ACP5	exonic	nonsynonymous SNV	ACP5:NM_001611:exon4:c.C398G:p.P133R,ACP5:NM_001111034:exon5:c.C398G:p.P133R,ACP5:NM_001111036:exon5:c.C398G:p.P133R,ACP5:NM_001322023:exon5:c.C398G:p.P133R,ACP5:NM_001111035:exon6:c.C398G:p.P133R	.	1.048	.	.	.	Fam32_f_m_aM_uM	0/0;0/1;0/1;0/0
chr19	12691450	12691450	C	A	FBXW9	exonic	nonsynonymous SNV	FBXW9:NM_032301:exon4:c.G683T:p.W228L	.	1.456	.	.	.	Fam95_f_m_aM_aM_uF	0/0;0/1;0/1;0/1;0/1
chr19	12694706	12694706	A	G	FBXW9	exonic	nonsynonymous SNV	FBXW9:NM_032301:exon3:c.T566C:p.L189P	.	1.706	.	3.28E-05	1.40E-05	Fam30_f_m_aM_uM	0/0;0/1;0/1;0/1
chr19	12696529	12696529	G	C	FBXW9	exonic	nonsynonymous SNV	FBXW9:NM_032301:exon1:c.C53G:p.S18W	.	1.605	7.36E-05	3.48E-05	.	Fam114_f_m_aM	0/1;0/0;0/1
chr19	12706612	12706612	C	T	TNPO2	exonic	nonsynonymous SNV	TNPO2:NM_001136196:exon13:c.G1454A:p.R485H,TNPO2:NM_013433:exon13:c.G1454A:p.R485H,TNPO2:NM_001136195:exon14:c.G1454A:p.R485H	.	2.487	.	.	.	Fam94_f_m_aM	0/1;0/0;0/1
chr19	12737528	12737528	G	C	GET3	exonic	nonsynonymous SNV	GET3:NM_004317:exon1:c.G23C:p.W8S	.	1.092	0.0003	0.0004	0.0001	Fam99_f_m_aM_aM	0/0;0/1;0/0;0/1
chr19	12755439	12755439	C	A	BEST2	exonic	nonsynonymous SNV	BEST2:NM_017682:exon6:c.C697A:p.P233T	.	1.515	.	.	.	Fam117_f_m_aM_aF	0/0;0/1;0/0;0/1
chr19	12924185	12924185	C	T	FARSA	exonic	nonsynonymous SNV	FARSA:NM_004461:exon12:c.G1354A:p.V452M	.	1.21	7.34E-05	7.46E-05	2.09E-05	Fam42_f_m_aM_uF	0/1;0/0;0/1;0/1
chr19	12928349	12928349	G	C	FARSA	exonic	nonsynonymous SNV	FARSA:NM_004461:exon7:c.C834G:p.F278L	.	1.308	.	1.12E-05	6.98E-06	Fam122_f_m_aM	0/1;0/0;0/1
chr19	12948742	12948742	C	T	RAD23A	exonic	stopgain	RAD23A:NM_001270362:exon5:c.C529T:p.R177X,RAD23A:NM_001270363:exon5:c.C529T:p.R177X,RAD23A:NM_005053:exon5:c.C529T:p.R177X	0.548	.	.	1.12E-05	.	Fam101_f_m_aM	0/0;0/1;0/1
chr19	12948748	12948748	C	T	RAD23A	exonic	nonsynonymous SNV	RAD23A:NM_001270362:exon5:c.C535T:p.R179W,RAD23A:NM_001270363:exon5:c.C535T:p.R179W,RAD23A:NM_005053:exon5:c.C535T:p.R179W	.	1.154	.	7.46E-05	6.98E-06	Fam17_f_m_aM_uM	0/0;0/1;0/1;0/0
chr19	12954305	12954305	T	G	GADD45GIP1	exonic	nonsynonymous SNV	GADD45GIP1:NM_052850:exon2:c.A572C:p.K191T	.	1.549	.	3.27E-05	.	Fam49_f_m_aM	0/1;0/0;0/1
chr19	13025217	13025217	T	C	NFIX	exonic	nonsynonymous SNV	NFIX:NM_001365984:exon1:c.T221C:p.L74P,NFIX:NM_001365985:exon1:c.T221C:p.L74P,NFIX:NM_001271043:exon2:c.T248C:p.L83P,NFIX:NM_001271044:exon2:c.T200C:p.L67P,NFIX:NM_001365902:exon2:c.T224C:p.L75P,NFIX:NM_001365982:exon2:c.T224C:p.L75P,NFIX:NM_001365983:exon2:c.T83C:p.L28P,NFIX:NM_002501:exon2:c.T224C:p.L75P	.	3.285	.	.	.	Fam81_f_m_aM_uM	0/0;0/0;0/1;0/0
chr19	13983216	13983216	G	A	RFX1	exonic	stopgain	RFX1:NM_002918:exon4:c.C484T:p.Q162X	0.588	.	.	8.70E-05	.	Fam14_f_m_aM_aM	0/0;0/1;0/0;0/1
chr19	14156704	14156704	T	C	ADGRL1	exonic	nonsynonymous SNV	ADGRL1:NM_014921:exon16:c.A2987G:p.N996S,ADGRL1:NM_001008701:exon17:c.A3002G:p.N1001S	.	1.656	.	7.55E-05	.	Fam91_m_aM_dM_aM_dM	0/0;0/0;0/0;0/1;0/1
chr19	14162968	14162968	G	C	ADGRL1	exonic	nonsynonymous SNV	ADGRL1:NM_014921:exon5:c.C833G:p.T278S,ADGRL1:NM_001008701:exon6:c.C848G:p.T283S	.	1.746	0.0004	0.0002	0.0003	Fam63_f_m_aF	0/0;0/1;0/1
chr19	14163220	14163220	G	A	ADGRL1	exonic	nonsynonymous SNV	ADGRL1:NM_014921:exon5:c.C581T:p.A194V,ADGRL1:NM_001008701:exon6:c.C596T:p.A199V	.	1.468	.	.	.	Fam34_f_m_aM_uF	0/0;0/1;0/1;0/1
chr19	14397745	14397745	G	A	ADGRE5	exonic	nonsynonymous SNV	ADGRE5:NM_001784:exon5:c.G434A:p.R145H,ADGRE5:NM_001025160:exon6:c.G566A:p.R189H,ADGRE5:NM_078481:exon7:c.G713A:p.R238H	.	2.232	9.92E-05	0.0005	0.0001	Fam119_f_m_aM_aM	0/0;0/1;0/0;0/1
chr19	14972742	14972742	C	T	SLC1A6	exonic	nonsynonymous SNV	SLC1A6:NM_005071:exon1:c.G169A:p.A57T,SLC1A6:NM_001272088:exon2:c.G169A:p.A57T,SLC1A6:NM_001272087:exon4:c.G169A:p.A57T	.	1.283	.	4.56E-05	1.40E-05	Fam121_f_m_aF_aM	0/0;0/1;0/1;0/0
chr19	14972785	14972785	C	G	SLC1A6	exonic	nonsynonymous SNV	SLC1A6:NM_005071:exon1:c.G126C:p.Q42H,SLC1A6:NM_001272088:exon2:c.G126C:p.Q42H,SLC1A6:NM_001272087:exon4:c.G126C:p.Q42H	.	1.257	.	.	.	Fam53_f_m_aM	0/0;0/1;0/1
chr19	15021466	15021466	G	C	CCDC105	exonic	nonsynonymous SNV	CCDC105:NM_173482:exon4:c.G988C:p.D330H	.	1.245	7.35E-05	8.94E-05	4.88E-05	Fam84_f_m_aF	0/1;0/0;0/1
chr19	15179033	15179033	C	A	NOTCH3	exonic	nonsynonymous SNV	NOTCH3:NM_000435:exon22:c.G3710T:p.G1237V	.	1.273	.	.	.	Fam117_f_m_aM_aF	0/0;0/1;0/0;0/1
chr19	15184333	15184333	C	A	NOTCH3	exonic	nonsynonymous SNV	NOTCH3:NM_000435:exon16:c.G2528T:p.G843V	.	1.429	.	.	.	Fam95_f_m_aM_aM_uF	0/1;0/0;0/1;0/1;0/0
chr19	15191610	15191610	C	T	NOTCH3	exonic	nonsynonymous SNV	NOTCH3:NM_000435:exon6:c.G850A:p.A284T	.	1.251	7.35E-05	0.0002	0.0002	Fam92_f_m_aM_raM	0/1;0/0;0/1;0/1
chr19	15257055	15257055	T	C	BRD4	exonic	nonsynonymous SNV	BRD4:NM_001330384:exon8:c.A1460G:p.D487G,BRD4:NM_014299:exon8:c.A1460G:p.D487G,BRD4:NM_058243:exon8:c.A1460G:p.D487G	.	1.506	.	1.22E-05	1.40E-05	Fam87_f_m_aM_uM	0/1;0/0;0/1;0/1
chr19	17102423	17102423	G	A	MYO9B	exonic	nonsynonymous SNV	MYO9B:NM_001130065:exon2:c.G706A:p.V236M,MYO9B:NM_004145:exon2:c.G706A:p.V236M	.	1.784	.	3.27E-05	6.98E-06	Fam26_f_m_aM_uM	0/1;0/0;0/1;0/0
chr19	17206358	17206358	G	A	MYO9B	exonic	nonsynonymous SNV	MYO9B:NM_001130065:exon33:c.G5368A:p.D1790N,MYO9B:NM_004145:exon33:c.G5368A:p.D1790N	.	1.024	.	.	.	Fam50_f_m_aF	0/0;0/1;0/1

chr19	17207197	17207197	C	A	MYO9B	exonic	nonsynonymous SNV	MYO9B:NM_001130065:exon35:c.C5577A:p.D1859E,MYO9B:NM_004145:exon35:c.C5577A:p.D1859E	.	1.039	0.0002	4.88E-05	3.49E-05	Fam49_f_m_aM	0/0;0/1;0/1
chr19	17210358	17210358	C	T	MYO9B	exonic	nonsynonymous SNV	MYO9B:NM_001130065:exon37:c.C5774T:p.S1925L,MYO9B:NM_004145:exon37:c.C5774T:p.S1925L	.	1.012	0.0006	0.0002	9.77E-05	Fam13_f_m_aM	0/1;0/0;0/1
chr19	17215457	17215457	G	A	USE1	exonic	nonsynonymous SNV	USE1:NM_018467:exon1:c.G52A:p.E18K	.	1.154	.	.	.	Fam14_f_m_aM_aM	0/1;0/0;0/1;0/1
chr19	17232606	17232606	G	A	NR2F6	exonic	nonsynonymous SNV	NR2F6:NM_005234:exon4:c.C961T:p.P321S	.	1.407	.	4.50E-05	6.98E-06	Fam17_f_m_aM_uM	0/0;0/1;0/1;0/0
chr19	17339192	17339192	G	C	GTPBP3	exonic	nonsynonymous SNV	GTPBP3:NM_133644:exon5:c.G830C:p.R277T,GTPBP3:NM_001128855:exon6:c.G734C:p.R245T,GTPBP3:NM_001195422:exon6:c.G800C:p.R267T,GTPBP3:NM_032620:exon6:c.G734C:p.R245T	.	1.201	.	1.13E-05	6.98E-06	Fam73_f_m_aF_dM	0/1;0/0;0/1;0/0
chr19	17540548	17540548	G	T	NIBAN3	exonic	nonsynonymous SNV	NIBAN3:NM_001321828:exon8:c.G407T:p.R136L,NIBAN3:NM_001321826:exon9:c.G1136T:p.R379L,NIBAN3:NM_001321827:exon9:c.G1136T:p.R379L,NIBAN3:NM_001363609:exon9:c.G1136T:p.R379L,NIBAN3:NM_001098524:exon10:c.G1229T:p.R410L,NIBAN3:NM_173544:exon10:c.G1229T:p.R410L	.	1.004	7.35E-05	9.32E-05	5.58E-05	Fam24_f_m_aM_aM	0/1;0/0;0/1;0/1
chr19	17540548	17540548	G	T	NIBAN3	exonic	nonsynonymous SNV	NIBAN3:NM_001321828:exon8:c.G407T:p.R136L,NIBAN3:NM_001321826:exon9:c.G1136T:p.R379L,NIBAN3:NM_001321827:exon9:c.G1136T:p.R379L,NIBAN3:NM_001363609:exon9:c.G1136T:p.R379L,NIBAN3:NM_001098524:exon10:c.G1229T:p.R410L,NIBAN3:NM_173544:exon10:c.G1229T:p.R410L	.	1.004	7.35E-05	9.32E-05	5.58E-05	Fam40_f_m_aM_aM	0/1;0/0;0/1;0/1
chr19	17581337	17581337	C	T	COLGALT1	exonic	nonsynonymous SNV	COLGALT1:NM_024656:exon12:c.C1762T:p.R588C	.	1.143	0.0001	0.0003	0.0004	Fam38_f_m_aM	0/0;0/1;0/1
chr19	17581337	17581337	C	T	COLGALT1	exonic	nonsynonymous SNV	COLGALT1:NM_024656:exon12:c.C1762T:p.R588C	.	1.143	0.0001	0.0003	0.0004	Fam47_f_m_aM	0/1;0/0;0/1
chr19	17617790	17617790	G	T	UNC13A	exonic	nonsynonymous SNV	UNC13A:NM_001080421:exon39:c.C4470A:p.D1490E	.	1.871	.	.	.	Fam91_m_aM_dM_aM_dM	0/1;0/1;0/0;0/1;0/0
chr19	17618943	17618943	T	A	UNC13A	exonic	nonsynonymous SNV	UNC13A:NM_001080421:exon37:c.A4292T:p.N1431I	.	2.095	.	6.47E-05	2.09E-05	Fam40_f_m_aM_aM	0/0;0/1;0/0;0/1
chr19	17641522	17641522	T	C	UNC13A	exonic	nonsynonymous SNV	UNC13A:NM_001080421:exon20:c.A2507G:p.N836S	.	1.141	.	0.0001	0.0001	Fam6_f_m_aM	0/1;0/0;0/1
chr19	17734352	17734352	G	A	MAP1S	exonic	nonsynonymous SNV	MAP1S:NM_001308363:exon7:c.G3026A:p.G1009D,MAP1S:NM_018174:exon7:c.G3104A:p.G1035D	.	1.123	.	.	.	Fam90_f_m_aF_dM	0/0;0/1;0/1;0/1
chr19	17811968	17811968	T	C	B3GNT3	exonic	nonsynonymous SNV	B3GNT3:NM_014256:exon3:c.T965C:p.V322A	.	1.062	0.0004	0.0006	0.0006	Fam70_f_m_aM	0/1;0/0;0/1
chr19	18163344	18163344	C	T	PIK3R2	exonic	nonsynonymous SNV	PIK3R2:NM_005027:exon11:c.C1372T:p.R458C	.	1.385	.	1.12E-05	.	Fam15_f_m_aM_aM	0/0;0/1;0/1;0/1
chr19	18198769	18198769	C	T	RAB3A	exonic	nonsynonymous SNV	RAB3A:NM_002866:exon4:c.G428A:p.R143Q	.	2.131	.	1.12E-05	.	Fam16_f_m_aM_aM	0/0;0/1;0/1;0/0
chr19	18216772	18216772	C	T	PDE4C	exonic	nonsynonymous SNV	PDE4C:NM_001369701:exon11:c.G761A:p.R254Q,PDE4C:NM_001098818:exon12:c.G1358A:p.R453Q,PDE4C:NM_001098819:exon12:c.G1136A:p.R379Q,PDE4C:NM_000923:exon13:c.G1454A:p.R485Q,PDE4C:NM_001330172:exon13:c.G1454A:p.R485Q	.	1.049	.	.	.	Fam38_f_m_aM	0/1;0/0;0/1
chr19	18446757	18446757	T	C	ELL	exonic	nonsynonymous SNV	ELL:NM_006532:exon9:c.A1523G:p.D508G	.	1.795	.	.	.	Fam110_f_m_aM_aM_uMs	0/1;0/0;0/1;0/1;0/0
chr19	18541870	18541870	T	A	FKBP8	exonic	nonsynonymous SNV	FKBP8:NM_001308373:exon2:c.A101T:p.E34V,FKBP8:NM_012181:exon2:c.A101T:p.E34V	.	1.718	.	1.16E-05	.	Fam24_f_m_aM_aM	0/1;0/0;0/1;0/1
chr19	18589521	18589521	C	A	REX1BD	exonic	nonsynonymous SNV	REX1BD:NM_001100418:exon3:c.C291A:p.F97L,REX1BD:NM_001100419:exon3:c.C225A:p.F75L	.	1.259	.	.	.	Fam121_f_m_aF_aM	0/0;0/1;0/0;0/1
chr19	19147090	19147090	C	T	BORCS8-MEF2B;M	exonic	nonsynonymous SNV	MEF2B:NM_001145785:exon5:c.G487A:p.A163T,MEF2B:NM_001367282:exon5:c.G487A:p.A163T,BORCS8-MEF2B:NM_005919:exon7:c.G487A:p.A163T	.	1.132	.	0.0002	4.89E-05	Fam25_f_m_aM	0/0;0/1;0/1
chr19	19219115	19219115	C	A	NCAN	exonic	nonsynonymous SNV	NCAN:NM_004386:exon3:c.C274A:p.P92T	.	1.282	0.0006	7.46E-05	3.49E-05	Fam95_f_m_aM_aM_uF	0/0;0/1;0/1;0/1;0/0
chr19	19219301	19219301	C	G	NCAN	exonic	nonsynonymous SNV	NCAN:NM_004386:exon3:c.C460G:p.P154A	.	1.19	0.0004	0.0002	0.0001	Fam10_f_m_aM_uF	0/0;0/1;0/1;0/1
chr19	19224325	19224325	C	T	NCAN	exonic	nonsynonymous SNV	NCAN:NM_004386:exon5:c.C670T:p.R224C	.	1.42	.	.	.	Fam2_f_m_aF	0/0;0/1;0/1
chr19	19258559	19258559	G	T	HAPLN4	exonic	nonsynonymous SNV	HAPLN4:NM_023002:exon4:c.C781A:p.R261S	.	1.662	.	.	.	Fam106_f_m_aM	0/0;0/1;0/1
chr19	19544950	19544950	C	G	CILP2	exonic	nonsynonymous SNV	CILP2:NM_153221:exon8:c.C2405G:p.P802R	.	1.104	.	.	.	Fam62_f_m_aM_aM	0/0;0/1;0/1;0/1
chr19	19624401	19624401	C	T	LPAR2	exonic	nonsynonymous SNV	LPAR2:NM_004720:exon3:c.G911A:p.R304H	.	1.226	.	0.0006	2.09E-05	Fam116_f_m_aM_aF	0/0;0/1;0/1;0/1
chr19	19635030	19635030	C	T	GMIP	exonic	nonsynonymous SNV	GMIP:NM_001288998:exon15:c.G1657A:p.V553M,GMIP:NM_001288999:exon15:c.G1666A:p.V556M,GMIP:NM_016573:exon16:c.G1744A:p.V582M	.	1.808	.	0.0002	2.09E-05	Fam119_f_m_aM_aM	0/0;0/1;0/1;0/0
chr19	29812751	29812751	G	C	CCNE1	exonic	nonsynonymous SNV	CCNE1:NM_001322262:exon2:c.G41C:p.R14T,CCNE1:NM_001238:exon3:c.G86C:p.R29T,CCNE1:NM_001322259:exon3:c.G86C:p.R29T,CCNE1:NM_001322261:exon3:c.G86C:p.R29T	.	1.646	.	.	.	Fam45_f_m_aM_uF	0/0;0/1;0/1;0/1
chr19	30445414	30445414	T	G	ZNF536	exonic	nonsynonymous SNV	ZNF536:NM_001352260:exon2:c.T1852G:p.Y618D,ZNF536:NM_014717:exon2:c.T1852G:p.Y618D	.	2.321	7.37E-05	0.0002	7.70E-05	Fam74_f_m_aM_uM	0/0;0/1;0/1;0/1
chr19	31278576	31278576	A	G	TSHZ3	exonic	nonsynonymous SNV	TSHZ3:NM_020856:exon2:c.T1217C:p.M406T	.	1.139	.	.	.	Fam44_f_m_aM_uF	0/1;0/0;0/1;0/0
chr19	32864261	32864261	C	T	SLC7A9	exonic	nonsynonymous SNV	SLC7A9:NM_001126335:exon4:c.G313A:p.G105R,SLC7A9:NM_001243036:exon4:c.G313A:p.G105R,SLC7A9:NM_014270:exon4:c.G313A:p.G105R	.	1.076	0.0004	0.0006	0.0002	Fam105_f_m_aM	0/1;0/0;0/1
chr19	33205587	33205587	G	A	LRP3	exonic	nonsynonymous SNV	LRP3:NM_002333:exon5:c.G817A:p.A273T	.	1.016	.	0.0009	6.98E-06	Fam101_f_m_aM	0/0;0/1;0/1
chr19	33207022	33207022	G	A	LRP3	exonic	nonsynonymous SNV	LRP3:NM_002333:exon7:c.G1760A:p.R587Q	.	1.088	0.0003	0.0002	0.0003	Fam25_f_m_aM	0/0;0/1;0/1
chr19	33215911	33215911	C	T	SLC7A10	exonic	nonsynonymous SNV	SLC7A10:NM_019849:exon2:c.G214A:p.V72M	.	1.17	.	0.0002	5.58E-05	Fam4_f_m_aM	0/1;0/0;0/1
chr19	33772506	33772506	G	C	CHST8	exonic	nonsynonymous SNV	CHST8:NM_001127896:exon4:c.G718C:p.D240H,CHST8:NM_022467:exon4:c.G718C:p.D240H,CHST8:NM_001127895:exon5:c.G718C:p.D240H	.	1.63	.	.	.	Fam24_f_m_aM_aM	0/1;0/0;0/1;0/1
chr19	34495739	34495739	A	G	WTIP	exonic	nonsynonymous SNV	WTIP:NM_001080436:exon7:c.A1120G:p.R374G	.	1.108	0.0003	9.84E-05	0.0001	Fam99_f_m_aM_aM	0/1;0/0;0/0;0/1

chr19	35021578	35021578	G	A	GRAMD1A	exonic	nonsynonymous SNV	GRAMD1A:NM_001136199:exon13:c.G1531A:p.G511S,GRAMD1A:NM_001320035:exon13:c.G850A:p.G284S,GRAMD1A:NM_001320034:exon14:c.G1552A:p.G518S,GRAMD1A:NM_020895:exon14:c.G1552A:p.G518S,GRAMD1A:NM_001320036:exon15:c.G1810A:p.G604S	.	1.101	.	2.24E-05	6.98E-06	Fam10_f_m_aM_uF	0/0;0/1;0/1;0/1
chr19	35059888	35059888	C	A	HPN	exonic	nonsynonymous SNV	HPN:NM_182983:exon6:c.C305A:p.S102Y,HPN:NM_002151:exon7:c.C305A:p.S102Y	.	1.4	.	.	.	Fam12_f_m_aM_uM_aM	0/0;0/1;0/0;0/0;0/1
chr19	35351934	35351934	C	A	FFAR1	exonic	nonsynonymous SNV	FFAR1:NM_005303:exon1:c.C383A:p.A128E	.	1.351	.	6.55E-05	.	Fam57_f_m_aM_uF	0/1;0/0;0/1;0/1
chr19	35778298	35778298	C	T	ARHGAP33	exonic	nonsynonymous SNV	ARHGAP33:NM_001366178:exon4:c.C208T:p.R70C,ARHGAP33:NM_052948:exon4:c.C208T:p.R70C	.	1.583	7.35E-05	0.0006	0.0003	Fam33_f_m_aM_uM	0/0;0/1;0/1;0/1
chr19	35890005	35890005	G	A	NFKBID	exonic	nonsynonymous SNV	NFKBID:NM_001321831:exon11:c.C818T:p.P273L,NFKBID:NM_001365705:exon11:c.C773T:p.P258L,NFKBID:NM_032721:exon11:c.C1229T:p.P410L,NFKBID:NM_139239:exon11:c.C773T:p.P258L	.	1.326	.	1.20E-05	.	Fam72_f_m_aF_uF	0/0;0/1;0/1;0/1
chr19	35896459	35896459	G	A	NFKBID	exonic	nonsynonymous SNV	NFKBID:NM_001321831:exon7:c.C383T:p.A128V,NFKBID:NM_001365705:exon7:c.C338T:p.A113V,NFKBID:NM_001365706:exon7:c.C338T:p.A113V,NFKBID:NM_032721:exon7:c.C794T:p.A265V,NFKBID:NM_139239:exon7:c.C338T:p.A113V	.	1.798	0.0001	0.0002	5.58E-05	Fam52_f_m_aM	0/0;0/1;0/1
chr19	35896459	35896459	G	A	NFKBID	exonic	nonsynonymous SNV	NFKBID:NM_001321831:exon7:c.C383T:p.A128V,NFKBID:NM_001365705:exon7:c.C338T:p.A113V,NFKBID:NM_001365706:exon7:c.C338T:p.A113V,NFKBID:NM_032721:exon7:c.C794T:p.A265V,NFKBID:NM_139239:exon7:c.C338T:p.A113V	.	1.798	0.0001	0.0002	5.58E-05	Fam33_f_m_aM_uM	0/1;0/0;0/1;0/0
chr19	35903786	35903786	T	C	HCST	exonic	nonsynonymous SNV	HCST:NM_001007469:exon3:c.T124C:p.C42R,HCST:NM_014266:exon3:c.T124C:p.C42R	.	1.893	0.0004	0.0008	0.0004	Fam10_f_m_aM_uF	0/1;0/0;0/1;0/1
chr19	36393196	36393196	T	A	ZFP82	exonic	nonsynonymous SNV	ZFP82:NM_001321917:exon5:c.A1147T:p.I383F,ZFP82:NM_133466:exon5:c.A1144T:p.I382F	.	1.572	.	6.55E-05	.	Fam48_f_m_aM_uM	0/0;0/1;0/1;0/1
chr19	38081641	38081641	C	T	SIPA1L3	exonic	nonsynonymous SNV	SIPA1L3:NM_015073:exon3:c.C76T:p.L26F	.	1.095	.	4.60E-05	.	Fam34_f_m_aM_uF	0/0;0/1;0/1;0/0
chr19	38081641	38081641	C	T	SIPA1L3	exonic	nonsynonymous SNV	SIPA1L3:NM_015073:exon3:c.C76T:p.L26F	.	1.095	.	4.60E-05	.	Fam62_f_m_aM_aM	0/1;0/0;0/1;0/0
chr19	38082472	38082472	C	G	SIPA1L3	exonic	nonsynonymous SNV	SIPA1L3:NM_015073:exon3:c.C907G:p.R303G	.	1.461	0.0002	9.02E-05	4.19E-05	Fam40_f_m_aM_aM	0/1;0/0;0/1;0/1
chr19	38130651	38130651	G	A	SIPA1L3	exonic	nonsynonymous SNV	SIPA1L3:NM_015073:exon10:c.G3022A:p.V1008M	.	1.294	.	1.12E-05	1.40E-05	Fam119_f_m_aM_aM	0/1;0/0;0/1;0/1
chr19	38411354	38411354	G	A	RASGRP4	exonic	nonsynonymous SNV	RASGRP4:NM_001146206:exon11:c.C1141T:p.R381C,RASGRP4:NM_001146207:exon12:c.C1417T:p.R473C,RASGRP4:NM_001146203:exon13:c.C1432T:p.R478C,RASGRP4:NM_001146202:exon14:c.C1666T:p.R556C,RASGRP4:NM_001146204:exon14:c.C1501T:p.R501C,RASGRP4:NM_001146205:exon14:c.C1606T:p.R536C,RASGRP4:NM_170604:exon14:c.C1708T:p.R570C	.	1.147	0.0001	0.0003	0.0002	Fam115_f_m_aF_aM_aF	0/1;0/0;0/1;0/1;0/0
chr19	38473411	38473411	C	A	RYR1	exonic	nonsynonymous SNV	RYR1:NM_000540:exon28:c.C3800A:p.P1267H,RYR1:NM_001042723:exon28:c.C3800A:p.P1267H	.	1.107	.	6.54E-05	.	Fam10_f_m_aM_uF	0/1;0/0;0/1;0/1
chr19	38596381	38596381	C	T	MAP4K1	exonic	nonsynonymous SNV	MAP4K1:NM_001042600:exon26:c.G2047A:p.G683R,MAP4K1:NM_007181:exon26:c.G2047A:p.G683R	.	1.841	.	1.30E-05	1.40E-05	Fam54_f_m_aM_uF	0/1;0/0;0/1;0/1
chr19	38597550	38597550	C	T	MAP4K1	exonic	nonsynonymous SNV	MAP4K1:NM_001042600:exon23:c.G1714A:p.E572K,MAP4K1:NM_007181:exon23:c.G1714A:p.E572K	.	1.907	.	.	.	Fam35_f_m_aF_uM	0/1;0/0;0/1;0/1
chr19	38907498	38907498	C	T	NFKBIB	exonic	nonsynonymous SNV	NFKBIB:NM_001243116:exon5:c.C550T:p.R184C,NFKBIB:NM_001369699:exon5:c.C808T:p.R270C,NFKBIB:NM_001369700:exon5:c.C532T:p.R178C,NFKBIB:NM_002503:exon5:c.C808T:p.R270C	.	1.21	7.35E-05	0.0004	1.40E-05	Fam114_f_m_aM	0/1;0/0;0/1
chr19	38907580	38907580	G	A	NFKBIB	exonic	nonsynonymous SNV	NFKBIB:NM_001243116:exon5:c.G632A:p.G211E,NFKBIB:NM_001369699:exon5:c.G890A:p.G297E,NFKBIB:NM_001369700:exon5:c.G614A:p.G205E,NFKBIB:NM_002503:exon5:c.G890A:p.G297E	.	1.206	.	.	.	Fam24_f_m_aM_aM	0/0;0/1;0/1;0/1
chr19	39100962	39100962	A	C	ACP7	exonic	nonsynonymous SNV	ACP7:NM_001363687:exon7:c.A697C:p.T233P,ACP7:NM_001004318:exon8:c.A821C:p.N274T	.	1.142	.	.	.	Fam28_f_m_aF_uF	0/1;0/0;0/1;0/0
chr19	39390095	39390095	G	C	PAF1	exonic	nonsynonymous SNV	PAF1:NM_001256826:exon2:c.C114G:p.F38L,PAF1:NM_019088:exon3:c.C144G:p.F48L	.	2.134	.	.	.	Fam83_f_m_aF	0/1;0/0;0/1
chr19	39480897	39480897	T	G	TIMM50	exonic	nonsynonymous SNV	TIMM50:NM_001001563:exon1:c.T44G:p.L15R	.	1.295	.	.	.	Fam113_f_m_aF_raM_uF	0/1;0/0;0/0;0/1;0/1
chr19	39507209	39507209	G	A	DLL3	exonic	nonsynonymous SNV	DLL3:NM_016941:exon7:c.G1264A:p.G422S,DLL3:NM_203486:exon7:c.G1264A:p.G422S	.	1.547	.	.	.	Fam35_f_m_aF_uM	0/0;0/1;0/1;0/1
chr19	39825953	39825953	C	G	DYRK1B	exonic	nonsynonymous SNV	DYRK1B:NM_004714:exon11:c.G1652C:p.R551P,DYRK1B:NM_006483:exon11:c.G1532C:p.R511P,DYRK1B:NM_006484:exon12:c.G1568C:p.R523P	.	1.058	.	1.64E-05	1.41E-05	Fam66_f_m_aM	0/1;0/0;0/1
chr19	39826855	39826855	G	A	DYRK1B	exonic	nonsynonymous SNV	DYRK1B:NM_004714:exon9:c.C1228T:p.R410C,DYRK1B:NM_006483:exon9:c.C1108T:p.R370C,DYRK1B:NM_006484:exon10:c.C1144T:p.R382C	.	1.562	.	.	6.98E-06	Fam109_f_m_aM	0/1;0/0;0/1
chr19	39830431	39830431	G	A	DYRK1B	exonic	nonsynonymous SNV	DYRK1B:NM_004714:exon4:c.C316T:p.R106C,DYRK1B:NM_006483:exon4:c.C316T:p.R106C,DYRK1B:NM_006484:exon4:c.C316T:p.R106C	.	1.032	.	7.45E-05	6.98E-06	Fam80_f_m_aM_uM	0/0;0/1;0/1;0/1
chr19	40192089	40192089	G	T	MAP3K10	exonic	nonsynonymous SNV	MAP3K10:NM_002446:exon1:c.G58T:p.V20F	.	1.113	0.0004	0.0005	0.0003	Fam72_f_m_aF_uF	0/1;0/0;0/1;0/0
chr19	40577597	40577597	T	C	SHKBP1	exonic	nonsynonymous SNV	SHKBP1:NM_138392:exon4:c.T227C:p.L76P	.	1.875	.	.	6.99E-06	Fam9_f_m_aM_dM_uF	0/0;0/1;0/1;0/0;0/1
chr19	40611334	40611334	T	C	LTBP4	exonic	nonsynonymous SNV	LTBP4:NM_001042545:exon13:c.T1993C:p.S665P,LTBP4:NM_001042544:exon16:c.T2194C:p.S732P,LTBP4:NM_003573:exon16:c.T2083C:p.S695P	.	1.244	.	.	.	Fam15_f_m_aM_aM	0/0;0/1;0/0;0/1
chr19	40677369	40677369	C	T	NUMBL	exonic	nonsynonymous SNV	NUMBL:NM_001289979:exon6:c.G470A:p.R157Q,NUMBL:NM_001289980:exon6:c.G470A:p.R157Q,NUMBL:NM_004756:exon7:c.G593A:p.R198Q	.	1.285	.	0.0001	4.88E-05	Fam95_f_m_aM_aM_uF	0/1;0/0;0/1;0/1;0/0
chr19	40702627	40702627	C	T	COQ8B	exonic	nonsynonymous SNV	COQ8B:NM_001142555:exon9:c.G743A:p.R248H,COQ8B:NM_024876:exon10:c.G866A:p.R289H	.	1.038	.	6.56E-05	2.09E-05	Fam77_f_m_aM_aF	0/1;0/0;0/1;0/1

chr19	40718213	40718213	G	C	ITPKC	exonic	nonsynonymous SNV	ITPKC:NM_025194:exon1:c.G1078C:p.A360P	.	1.326	.	.	.		Fam64_f_m_aM	0/0;0/1;0/1
chr19	40800709	40800709	C	T	EGLN2	exonic	nonsynonymous SNV	EGLN2:NM_053046:exon2:c.C137T:p.P46L,EGLN2:NM_080732:exon2:c.C137T:p.P46L	.	1.528	.	6.18E-05	6.98E-06		Fam22_f_m_aF_aF_uF	0/1;0/0;0/1;0/1;0/0
chr19	41197851	41197851	G	A	CYP2S1	exonic	nonsynonymous SNV	CYP2S1:NM_030622:exon3:c.G416A:p.G139D	.	1.136	.	.	.		Fam87_f_m_aM_uM	0/1;0/0;0/1;0/1
chr19	41198537	41198537	G	A	CYP2S1	exonic	nonsynonymous SNV	CYP2S1:NM_030622:exon4:c.G569A:p.R190H	.	1.107	7.36E-05	2.23E-05	.		Fam17_f_m_aM_uM	0/1;0/0;0/1;0/0
chr19	41397216	41397216	A	G	EXOSC5	exonic	nonsynonymous SNV	EXOSC5:NM_020158:exon1:c.T113C:p.L38P	.	1.012	.	.	.		Fam88_f_m_aF	0/0;0/1;0/1
chr19	41879134	41879134	C	T	CD79A	exonic	nonsynonymous SNV	CD79A:NM_001783:exon2:c.C224T:p.T75M,CD79A:NM_021601:exon2:c.C224T:p.T75M	.	1.225	.	0.001	0.0001		Fam32_f_m_aM_uM	0/0;0/1;0/1;0/1
chr19	42042595	42042595	G	A	GRIK5	exonic	nonsynonymous SNV	GRIK5:NM_001301030:exon11:c.C1430T:p.P477L,GRIK5:NM_002088:exon11:c.C1430T:p.P477L	.	1.947	.	.	.		Fam89_f_m_aM	0/1;0/0;0/1
chr19	42216709	42216709	G	A	DEDD2	exonic	nonsynonymous SNV	DEDD2:NM_001270614:exon2:c.C299T:p.P100L,DEDD2:NM_001270615:exon2:c.C299T:p.P100L,DEDD2:NM_133328:exon2:c.C299T:p.P100L	.	1.651	.	7.79E-05	6.98E-06		Fam9_f_m_aM_dM_uF	0/1;0/0;0/1;0/1;0/1
chr19	42242290	42242290	C	T	GSK3A	exonic	nonsynonymous SNV	GSK3A:NM_019884:exon1:c.G176A:p.G59D	.	1.267	.	.	.		Fam20_f_m_aM_uF	0/0;0/1;0/1;0/0
chr19	42249238	42249238	C	T	ERF	exonic	nonsynonymous SNV	ERF:NM_001301035:exon4:c.G649A:p.G217R,ERF:NM_001308402:exon4:c.G649A:p.G217R,ERF:NM_001312656:exon4:c.G649A:p.G217R,ERF:NM_006494:exon4:c.G874A:p.G292R	.	1.756	0.0003	6.49E-05	1.40E-05		Fam107_f_m_aM	0/1;0/0;0/1
chr19	42249926	42249926	G	T	ERF	exonic	nonsynonymous SNV	ERF:NM_001301035:exon3:c.C49A:p.R17S,ERF:NM_001308402:exon3:c.C49A:p.R17S,ERF:NM_001312656:exon3:c.C49A:p.R17S,ERF:NM_006494:exon3:c.C274A:p.R92S	.	1.729	.	.	.		Fam1_f_m_aM	0/1;0/0;0/1
chr19	42375771	42375771	G	A	MEGF8	exonic	nonsynonymous SNV	MEGF8:NM_001410:exon41:c.G7333A:p.V2445M,MEGF8:NM_001271938:exon42:c.G7534A:p.V2512M	.	1.282	0.0006	0.0008	0.0002		Fam28_f_m_aF_uF	0/1;0/0;0/1;0/1
chr19	43719174	43719174	G	A	IRGC	exonic	nonsynonymous SNV	IRGC:NM_019612:exon2:c.G616A:p.G206S	.	1.221	.	3.31E-05	.		Fam16_f_m_aM_aM	0/1;0/0;0/1;0/1
chr19	44647327	44647327	T	C	PVR	exonic	nonsynonymous SNV	PVR:NM_001135768:exon2:c.T184C:p.S62P,PVR:NM_001135769:exon2:c.T184C:p.S62P,PVR:NM_001135770:exon2:c.T184C:p.S62P,PVR:NM_006505:exon2:c.T184C:p.S62P	.	1.234	.	.	.		Fam12_f_m_aM_uM_aM	0/1;0/0;0/1;0/0;0/0
chr19	44781044	44781044	G	A	CBLC	exonic	nonsynonymous SNV	CBLC:NM_001130852:exon2:c.G493A:p.G165R,CBLC:NM_012116:exon2:c.G493A:p.G165R	.	1.081	0.0003	0.0005	0.0004		Fam6_f_m_aM	0/0;0/1;0/1
chr19	44977457	44977457	C	T	CLPTM1	exonic	nonsynonymous SNV	CLPTM1:NM_001282175:exon5:c.C541T:p.R181W,CLPTM1:NM_001282176:exon5:c.C277T:p.R93W,CLPTM1:NM_001294:exon5:c.C583T:p.R195W	.	1.708	.	1.12E-05	.		Fam114_f_m_aM	0/1;0/0;0/1
chr19	44990451	44990451	T	G	CLPTM1	exonic	nonsynonymous SNV	CLPTM1:NM_001282175:exon10:c.T1147G:p.F383V,CLPTM1:NM_001282176:exon10:c.T883G:p.F295V,CLPTM1:NM_001294:exon10:c.T1189G:p.F397V	.	1.157	0.0006	0.0007	0.0003		Fam16_f_m_aM_aM	0/0;0/1;0/1;0/1
chr19	44992641	44992641	G	A	CLPTM1	exonic	nonsynonymous SNV	CLPTM1:NM_001282175:exon14:c.G1712A:p.R571Q,CLPTM1:NM_001282176:exon14:c.G1448A:p.R483Q,CLPTM1:NM_001294:exon14:c.G1754A:p.R585Q	.	1.9	7.35E-05	1.13E-05	.		Fam16_f_m_aM_aM	0/0;0/1;0/1;0/1
chr19	45071876	45071876	C	T	ZNF296	exonic	nonsynonymous SNV	ZNF296:NM_145288:exon3:c.G1153A:p.G385R	.	1.645	7.35E-05	0.001	5.58E-05		Fam84_f_m_aF	0/0;0/1;0/1
chr19	45152615	45152615	G	A	NKPD1	exonic	nonsynonymous SNV	NKPD1:NM_198478:exon4:c.C1822T:p.R608C	.	1.76	.	1.35E-05	.		Fam120_f_m_aM_aM	0/0;0/1;0/1;0/1
chr19	45712213	45712213	G	A	FBXO46	exonic	nonsynonymous SNV	FBXO46:NM_001080469:exon2:c.C1283T:p.P428L,FBXO46:NM_001329632:exon2:c.C1283T:p.P428L,FBXO46:NM_001329633:exon2:c.C1283T:p.P428L,FBXO46:NM_001329634:exon2:c.C1283T:p.P428L	.	1.689	0.0009	0.0007	0.0002		Fam11_f_m_aM_uM	0/1;0/0;0/1;0/1
chr19	45712819	45712819	C	G	FBXO46	exonic	nonsynonymous SNV	FBXO46:NM_001080469:exon2:c.G677C:p.R226P,FBXO46:NM_001329632:exon2:c.G677C:p.R226P,FBXO46:NM_001329633:exon2:c.G677C:p.R226P,FBXO46:NM_001329634:exon2:c.G677C:p.R226P	.	2.004	.	6.75E-05	6.98E-06		Fam52_f_m_aM	0/1;0/0;0/1
chr19	45768654	45768654	G	A	SIX5	exonic	nonsynonymous SNV	SIX5:NM_175875:exon1:c.C191T:p.P64L	.	1.822	.	.	1.44E-05		Fam7_f_m_aM_aM_uM	0/0;0/1;0/0;0/1;0/1
chr19	45771642	45771642	C	T	DMPK	exonic	nonsynonymous SNV	DMPK:NM_001288765:exon10:c.G1259A:p.R420Q,DMPK:NM_001081563:exon11:c.G1556A:p.R519Q,DMPK:NM_001081560:exon12:c.G1511A:p.R504Q,DMPK:NM_001081562:exon12:c.G1511A:p.R504Q,DMPK:NM_001288766:exon12:c.G1511A:p.R504Q,DMPK:NM_004409:exon12:c.G1526A:p.R509Q,DMPK:NM_001288764:exon13:c.G1604A:p.R535Q	.	1.061	.	.	6.98E-06		Fam115_f_m_aF_aM_aF	0/0;0/1;0/0;0/1;0/1
chr19	46723174	46723174	G	A	STRN4	exonic	nonsynonymous SNV	STRN4:NM_001039877:exon13:c.C1726T:p.R576C,STRN4:NM_013403:exon13:c.C1705T:p.R569C	.	1.469	.	0.0004	1.40E-05		Fam113_f_m_aF_raM_uF	0/0;0/0;0/0;0/1;0/0
chr19	47046013	47046013	C	T	TMEM160	exonic	nonsynonymous SNV	TMEM160:NM_017854:exon3:c.G541A:p.E181K	.	1.321	.	.	.		Fam57_f_m_aM_uF	0/0;0/1;0/1;0/1
chr19	47209207	47209207	G	A	SAE1	exonic	stopgain	SAE1:NM_001145713:exon7:c.G782A:p.W261X	0.491	0.753	.	6.56E-05	.		Fam31_f_m_aM_uF	0/0;0/1;0/1;0/1
chr19	48136106	48136106	G	A	LIG1	exonic	nonsynonymous SNV	LIG1:NM_001289064:exon13:c.C1147T:p.R383C,LIG1:NM_001289063:exon14:c.C1258T:p.R420C,LIG1:NM_001320971:exon14:c.C1261T:p.R421C,LIG1:NM_000234:exon15:c.C1351T:p.R451C,LIG1:NM_001320970:exon15:c.C1348T:p.R450C	.	1.129	.	1.73E-05	3.49E-05		Fam15_f_m_aM_aM	0/0;0/1;0/0;0/1
chr19	48462371	48462371	T	G	KCNJ14	exonic	nonsynonymous SNV	KCNJ14:NM_013348:exon2:c.T647G:p.M216R	.	2.676	.	.	.		Fam57_f_m_aM_uF	0/0;0/1;0/1;0/1
chr19	48610846	48610846	A	G	FAM83E	exonic	nonsynonymous SNV	FAM83E:NM_017708:exon2:c.T467C:p.L156P	.	1.124	.	.	.		Fam26_f_m_aM_uM	0/1;0/0;0/1;0/0
chr19	48635666	48635666	C	T	DBP	exonic	nonsynonymous SNV	DBP:NM_001352:exon2:c.G464A:p.G155D	.	1.105	0.0001	.	0.0001		Fam69_f_m_aM	0/1;0/0;0/1
chr19	48645617	48645617	G	A	CA11	exonic	nonsynonymous SNV	CA11:NM_001217:exon1:c.C16T:p.R6C	.	1.957	.	0.0004	7.00E-06		Fam91_m_aM_dM_aM_dM	0/0;0/0;0/1;0/1;0/0
chr19	48729502	48729502	T	C	RASIP1	exonic	nonsynonymous SNV	RASIP1:NM_017805:exon5:c.A1268G:p.Y423C	.	1.722	.	0.0002	4.19E-05		Fam15_f_m_aM_aM	0/0;0/1;0/1;0/0
chr19	48739362	48739362	C	T	RASIP1	exonic	nonsynonymous SNV	RASIP1:NM_017805:exon3:c.G421A:p.A141T	.	1.461	.	.	.		Fam43_f_m_aM	0/0;0/1;0/1
chr19	48745804	48745804	G	T	IZUMO1	exonic	nonsynonymous SNV	IZUMO1:NM_182575:exon2:c.C56A:p.A19D	.	1.324	.	.	.		Fam71_f_m_aF	0/1;0/0;0/1

chr19	48799726	48799726	G	C	BCAT2	exonic	nonsynonymous SNV	BCAT2:NM_001164773:exon4:c.C368G:p.P123R,BCAT2:NM_001190:exon6:c.C644G:p.P215R,BCAT2:NM_001284325:exon7:c.C524G:p.P175R	.	1.263	0.0003	0.0003	0.0003	Fam74_f_m_aM_uM	0/1;0/0;0/1;0/1
chr19	48806597	48806597	C	T	BCAT2	exonic	nonsynonymous SNV	BCAT2:NM_001190:exon3:c.G220A:p.G74S,BCAT2:NM_001284325:exon4:c.G100A:p.G34S	.	1.184	0.0001	0.0007	0.0002	Fam80_f_m_aM_uM	0/1;0/0;0/1;0/0
chr19	48985862	48985862	G	C	GYS1	exonic	nonsynonymous SNV	GYS1:NM_001161587:exon3:c.C474G:p.N158K,GYS1:NM_002103:exon4:c.C666G:p.N222K	.	1.474	0.0007	0.0007	0.0004	Fam90_f_m_aF_dM	0/0;0/1;0/1;0/0
chr19	48985888	48985888	A	G	GYS1	exonic	nonsynonymous SNV	GYS1:NM_001161587:exon3:c.T448C:p.C150R,GYS1:NM_002103:exon4:c.T640C:p.C214R	.	1.857	.	.	.	Fam27_f_m_aF_uM_uM	0/0;0/1;0/1;0/0;0/1
chr19	49086537	49086537	T	G	SNRNP70	exonic	nonsynonymous SNV	SNRNP70:NM_001301069:exon2:c.T123G:p.I41M,SNRNP70:NM_003089:exon2:c.T123G:p.I41M	.	2.248	.	.	.	Fam30_f_m_aM_uM	0/0;0/1;0/1;0/0
chr19	49128018	49128018	G	T	PPFIA3	exonic	nonsynonymous SNV	PPFIA3:NM_003660:exon2:c.G145T:p.A49S	.	1.475	.	.	.	Fam33_f_m_aM_uM	0/0;0/1;0/1;0/1
chr19	49157889	49157889	A	C	TRPM4	exonic	nonsynonymous SNV	TRPM4:NM_001195227:exon1:c.A23C:p.Q8P,TRPM4:NM_001321281:exon1:c.A23C:p.Q8P,TRPM4:NM_017636:exon1:c.A23C:p.Q8P	.	1.084	.	.	.	Fam79_f_m_aM_uM	0/0;0/1;0/1;0/0
chr19	49167957	49167957	A	G	TRPM4	exonic	nonsynonymous SNV	TRPM4:NM_001195227:exon4:c.A308G:p.Y103C,TRPM4:NM_017636:exon4:c.A308G:p.Y103C	.	1.099	0.001	0.0006	0.0004	Fam32_f_m_aM_uM	0/1;0/0;0/1;0/0
chr19	49172108	49172108	G	C	TRPM4	exonic	nonsynonymous SNV	TRPM4:NM_001321281:exon7:c.G805C:p.A269P,TRPM4:NM_001321283:exon7:c.G628C:p.A210P,TRPM4:NM_001195227:exon9:c.G1150C:p.A384P,TRPM4:NM_017636:exon9:c.G1150C:p.A384P	.	1.073	.	0.0004	.	Fam116_f_m_aM_aF	0/0;0/1;0/0;0/1
chr19	49210301	49210301	T	C	TRPM4	exonic	nonsynonymous SNV	TRPM4:NM_001321285:exon16:c.T2162C:p.L721P,TRPM4:NM_001321281:exon19:c.T2879C:p.L960P,TRPM4:NM_001321283:exon19:c.T2702C:p.L901P,TRPM4:NM_001195227:exon20:c.T2789C:p.L930P,TRPM4:NM_001321282:exon20:c.T1616C:p.L539P,TRPM4:NM_017636:exon21:c.T3224C:p.L1075P	.	1.089	0.0008	0.0001	0.0002	Fam59_f_m_aF_uM	0/1;0/0;0/1;0/0
chr19	49447053	49447053	G	A	PIH1D1	exonic	nonsynonymous SNV	PIH1D1:NM_017916:exon7:c.C658T:p.L220F	.	1.329	.	.	.	Fam49_f_m_aM	0/1;0/0;0/1
chr19	49490262	49490262	G	A	RPL13A	exonic	nonsynonymous SNV	RPL13A:NM_012423:exon3:c.G119A:p.G40D	.	1.025	0.0006	0.0005	0.0002	Fam85_f_m_aM_aM	0/0;0/1;0/0;0/1
chr19	49534365	49534365	C	T	RCN3	exonic	nonsynonymous SNV	RCN3:NM_020650:exon3:c.C415T:p.R139C	.	1.939	.	4.60E-05	1.40E-05	Fam118_f_m_aM_aF_uM	0/1;0/0;0/1;0/1;0/1
chr19	49595846	49595846	G	C	PRR12	exonic	nonsynonymous SNV	PRR12:NM_020719:exon4:c.G1511C:p.G504A	.	1.23	.	0.0002	6.98E-06	Fam53_f_m_aM	0/1;0/0;0/1
chr19	49597399	49597399	G	A	PRR12	exonic	nonsynonymous SNV	PRR12:NM_020719:exon4:c.G3064A:p.V1022I	.	1.099	.	.	.	Fam84_f_m_aF	0/0;0/1;0/1
chr19	49653618	49653618	C	T	SCAF1	exonic	nonsynonymous SNV	SCAF1:NM_021228:exon7:c.C3229T:p.R1077C	.	1.736	.	0.0006	.	Fam10_f_m_aM_uF	0/0;0/1;0/1;0/1
chr19	49654384	49654384	C	G	SCAF1	exonic	nonsynonymous SNV	SCAF1:NM_021228:exon8:c.C3352G:p.L1118V	.	1.186	.	.	.	Fam110_f_m_aM_aM_uMs	0/0;0/1;0/0;0/1;0/0
chr19	50625263	50625263	G	A	SYT3	exonic	nonsynonymous SNV	SYT3:NM_032298:exon7:c.C1606T:p.R536C,SYT3:NM_001160328:exon9:c.C1606T:p.R536C,SYT3:NM_001160329:exon9:c.C1606T:p.R536C	.	1.534	.	.	6.98E-06	Fam9_f_m_aM_dM_uF	0/1;0/0;0/1;0/0;0/0
chr19	50629457	50629457	G	A	SYT3	exonic	nonsynonymous SNV	SYT3:NM_032298:exon4:c.C1118T:p.P373L,SYT3:NM_001160328:exon6:c.C1118T:p.P373L,SYT3:NM_001160329:exon6:c.C1118T:p.P373L	.	1.398	.	.	.	Fam62_f_m_aM_aM	0/1;0/0;0/0;0/1
chr19	50825878	50825878	C	A	KLK15	exonic	nonsynonymous SNV	KLK15:NM_001277081:exon5:c.G686T:p.C229F,KLK15:NM_017509:exon5:c.G689T:p.C230F	.	1.521	.	.	.	Fam16_f_m_aM_aM	0/0;0/1;0/1;0/1
chr19	51965747	51965747	A	G	ZNF350	exonic	nonsynonymous SNV	ZNF350:NM_021632:exon5:c.T706C:p.C236R	.	1.042	.	.	.	Fam100_f_m_aF	0/0;0/1;0/1
chr19	53577290	53577290	G	A	ZNF331	exonic	nonsynonymous SNV	ZNF331:NM_001253801:exon5:c.G730A:p.E244K,ZNF331:NM_001317113:exon5:c.G730A:p.E244K,ZNF331:NM_001317116:exon5:c.G730A:p.E244K,ZNF331:NM_001317119:exon5:c.G730A:p.E244K,ZNF331:NM_001317121:exon5:c.G730A:p.E244K,ZNF331:NM_001079906:exon6:c.G730A:p.E244K,ZNF331:NM_001079907:exon6:c.G730A:p.E244K,ZNF331:NM_001253799:exon6:c.G730A:p.E244K,ZNF331:NM_001253800:exon6:c.G730A:p.E244K,ZNF331:NM_001317114:exon6:c.G730A:p.E244K,ZNF331:NM_001317117:exon6:c.G730A:p.E244K,ZNF331:NM_001317118:exon6:c.G730A:p.E244K,ZNF331:NM_001253798:exon7:c.G730A:p.E244K,ZNF331:NM_001317115:exon7:c.G730A:p.E244K,ZNF331:NM_001317120:exon7:c.G730A:p.E244K,ZNF331:NM_018555:exon7:c.G730A:p.E244K	.	1.691	0.0002	0.0001	0.0001	Fam1_f_m_aM	0/1;0/0;0/1
chr19	53914515	53914515	G	A	CACNG7	exonic	nonsynonymous SNV	CACNG7:NM_031896:exon2:c.G212A:p.R71H	.	1.027	.	7.45E-05	6.98E-06	Fam123_f_aF	0/1;0/1
chr19	54096050	54096050	G	C	OSCAR	exonic	nonsynonymous SNV	OSCAR:NM_001282350:exon4:c.C444G:p.F148L,OSCAR:NM_133168:exon4:c.C444G:p.F148L,OSCAR:NM_001282349:exon5:c.C477G:p.F159L,OSCAR:NM_133169:exon5:c.C477G:p.F159L,OSCAR:NM_130771:exon6:c.C489G:p.F163L,OSCAR:NM_206818:exon6:c.C489G:p.F163L	.	1.215	0.0006	0.0002	0.0001	Fam36_f_m_aM_uM	0/1;0/0;0/1;0/0
chr19	54118454	54118454	T	A	PRPF31	exonic	nonsynonymous SNV	PRPF31:NM_015629:exon2:c.T176A:p.M59K	.	1.258	.	.	.	Fam107_f_m_aM	0/1;0/0;0/1
chr19	54121892	54121892	C	T	PRPF31	exonic	nonsynonymous SNV	PRPF31:NM_015629:exon4:c.C271T:p.R91C	.	1.115	0.0004	0.0003	9.77E-05	Fam15_f_m_aM_aM	0/0;0/1;0/1;0/0
chr19	54192323	54192323	T	G	TSEN34	exonic	nonsynonymous SNV	TSEN34:NM_001077446:exon3:c.T695G:p.F232C,TSEN34:NM_001282332:exon4:c.T695G:p.F232C,TSEN34:NM_001282333:exon4:c.T704G:p.F235C,TSEN34:NM_024075:exon4:c.T695G:p.F232C	.	1.195	.	.	.	Fam91_m_aM_dM_aM_dM	0/0;0/1;0/1;0/0;0/1
chr19	55096117	55096117	G	A	PPP1R12C	exonic	nonsynonymous SNV	PPP1R12C:NM_001271618:exon8:c.C1087T:p.R363C,PPP1R12C:NM_017607:exon8:c.C1087T:p.R363C	.	1.287	.	0.0001	4.19E-05	Fam72_f_m_aF_uF	0/1;0/0;0/1;0/0
chr19	55289548	55289548	C	T	BRSK1	exonic	nonsynonymous SNV	BRSK1:NM_032430:exon4:c.C386T:p.T129M	.	2.715	.	0.0001	2.79E-05	Fam16_f_m_aM_aM	0/0;0/1;0/1;0/0
chr19	55354461	55354461	G	A	COX6B2	exonic	nonsynonymous SNV	COX6B2:NM_001369798:exon2:c.C61T:p.P21S,COX6B2:NM_001369799:exon2:c.C61T:p.P21S,COX6B2:NM_001369800:exon2:c.C61T:p.P21S,COX6B2:NM_144613:exon2:c.C61T:p.P21S	.	1.1	.	.	6.98E-06	Fam14_f_m_aM_aM	0/1;0/0;0/1;0/0

chr19	55376993	55376993	G	A	TMEM190	exonic	nonsynonymous SNV	TMEM190:NM_139172:exon2:c.G61A:p.G21R	.	1.412	0.0003	0.0004	0.0002	Fam2_f_m_aF	0/0;0/1;0/1
chr19	55377616	55377616	C	T	TMEM190	exonic	nonsynonymous SNV	TMEM190:NM_139172:exon3:c.C118T:p.R40W	.	1.16	.	3.29E-05	1.40E-05	Fam101_f_m_aM	0/1;0/0;0/1
chr19	55486586	55486586	G	C	NAT14	exonic	nonsynonymous SNV	NAT14:NM_020378:exon3:c.G251C:p.R84P	.	1.143	0.0002	0.0001	7.68E-05	Fam92_f_m_aM_raM	0/1;0/0;0/1;0/1
chr19	55530214	55530214	A	G	SBK2	exonic	nonsynonymous SNV	SBK2:NM_001370096:exon4:c.T566C:p.V189A	.	2.302	.	2.53E-05	6.98E-06	Fam6_f_m_aM	0/1;0/0;0/1
chr19	56146960	56146960	G	A	ZNF444	exonic	nonsynonymous SNV	ZNF444:NM_001253792:exon3:c.G49A:p.D17N,ZNF444:NM_018337:exon3:c.G49A:p.D17N	.	1.858	.	.	.	Fam9_f_m_aM_dM_uF	0/1;0/0;0/1;0/1;0/0
chr19	56159930	56159930	C	G	ZNF444	exonic	nonsynonymous SNV	ZNF444:NM_001253792:exon5:c.C710G:p.P237R,ZNF444:NM_018337:exon5:c.C713G:p.P238R	.	1.748	.	.	.	Fam51_f_m_aM_uF	0/1;0/0;0/1;0/0
chr19	58356914	58356914	G	A	ZNF497	exonic	nonsynonymous SNV	ZNF497:NM_001207009:exon2:c.C722T:p.T241M,ZNF497:NM_198458:exon3:c.C722T:p.T241M	.	1.078	.	2.57E-05	7.00E-06	Fam6_f_m_aM	0/1;0/0;0/1
chr19	58357131	58357131	T	C	ZNF497	exonic	nonsynonymous SNV	ZNF497:NM_001207009:exon2:c.A505G:p.K169E,ZNF497:NM_198458:exon3:c.A505G:p.K169E	.	1.205	0.0004	0.0004	0.0003	Fam10_f_m_aM_uF	0/1;0/0;0/1;0/1
chr19	58470901	58470901	A	C	ZNF324	exonic	nonsynonymous SNV	ZNF324:NM_014347:exon4:c.A409C:p.T137P	.	1.8	.	.	.	Fam19_f_m_aM	0/0;0/1;0/1
chr2	45460	45460	T	C	FAM110C	exonic	nonsynonymous SNV	FAM110C:NM_001077710:exon1:c.A926G:p.Q309R	.	1.088	.	.	6.98E-06	Fam114_f_m_aM	0/1;0/0;0/1
chr2	1648445	1648445	C	T	PXDN	exonic	nonsynonymous SNV	PXDN:NM_012293:exon17:c.G3335A:p.G1112D	.	1.388	.	.	.	Fam70_f_m_aM	0/0;0/1;0/1
chr2	3194030	3194030	G	T	EIPR1	exonic	nonsynonymous SNV	EIPR1:NM_001330531:exon6:c.C358A:p.P120T,EIPR1:NM_003310:exon7:c.C790A:p.P264T,EIPR1:NM_001330530:exon8:c.C871A:p.P291T	.	1.409	0.0002	0.0003	8.38E-05	Fam18_f_m_aM_uF	0/0;0/1;0/1;0/1
chr2	5693118	5693118	A	G	SOX11	exonic	nonsynonymous SNV	SOX11:NM_003108:exon1:c.A397G:p.S133G	.	1.025	.	.	6.98E-06	Fam113_f_m_aF_raM_uF	0/0;0/0;0/0;0/1;0/0
chr2	9961240	9961240	A	G	GRHL1	exonic	nonsynonymous SNV	GRHL1:NM_198182:exon4:c.A473G:p.K158R	.	1.041	.	3.27E-05	.	Fam47_f_m_aM	0/0;0/1;0/1
chr2	10419935	10419935	G	A	HPCAL1	exonic	nonsynonymous SNV	HPCAL1:NM_001258357:exon3:c.G178A:p.D60N,HPCAL1:NM_001258358:exon3:c.G178A:p.D60N,HPCAL1:NM_001258359:exon3:c.G178A:p.D60N,HPCAL1:NM_002149:exon3:c.G178A:p.D60N,HPCAL1:NM_134421:exon4:c.G178A:p.D60N	.	1.945	.	.	.	Fam98_f_m_aM	0/1;0/0;0/1
chr2	10913776	10913776	C	A	KCNF1	exonic	nonsynonymous SNV	KCNF1:NM_002236:exon1:c.C1350A:p.D450E	.	1.053	.	.	.	Fam42_f_m_aM_uF	0/1;0/0;0/1;0/1
chr2	11207781	11207781	T	C	ROCK2	exonic	nonsynonymous SNV	ROCK2:NM_001321643:exon20:c.A2236G:p.N746D,ROCK2:NM_004850:exon20:c.A2494G:p.N832D	.	1.692	7.35E-05	3.37E-05	6.98E-06	Fam7_f_m_aM_aM_uM	0/0;0/1;0/1;0/0;0/1
chr2	11588812	11588812	C	T	GREB1	exonic	nonsynonymous SNV	GREB1:NM_014668:exon10:c.C1226T:p.T409M,GREB1:NM_033090:exon10:c.C1226T:p.T409M	.	1.072	.	0.0002	0.0002	Fam10_f_m_aM_uF	0/1;0/0;0/1;0/1
chr2	11588827	11588827	C	T	GREB1	exonic	nonsynonymous SNV	GREB1:NM_014668:exon10:c.C1241T:p.S414F,GREB1:NM_033090:exon10:c.C1241T:p.S414F	.	1.213	.	6.17E-05	6.98E-06	Fam98_f_m_aM	0/1;0/0;0/1
chr2	14634613	14634613	T	C	LRATD1	exonic	nonsynonymous SNV	LRATD1:NM_145175:exon2:c.T634C:p.S212P,LRATD1:NM_001369364:exon3:c.T634C:p.S212P	.	1.518	.	.	.	Fam87_f_m_aM_uM	0/0;0/1;0/1;0/1
chr2	15618263	15618263	G	C	DDX1	exonic	nonsynonymous SNV	DDX1:NM_004939:exon16:c.G1199C:p.R400T	.	1.139	0.0003	0.0001	7.68E-05	Fam92_f_m_aM_raM	0/0;0/0;0/0;0/1
chr2	20278672	20278672	C	A	PUM2	exonic	nonsynonymous SNV	PUM2:NM_001282752:exon12:c.G1700T:p.G567V,PUM2:NM_001352930:exon12:c.G1190T:p.G397V,PUM2:NM_001352917:exon13:c.G1868T:p.G623V,PUM2:NM_001352918:exon13:c.G1868T:p.G623V,PUM2:NM_001352919:exon13:c.G1868T:p.G623V,PUM2:NM_015317:exon13:c.G1868T:p.G623V	.	1.323	.	0.0002	3.50E-05	Fam80_f_m_aM_uM	0/0;0/1;0/1;0/1
chr2	21006966	21006966	G	C	APOB	exonic	stopgain	APOB:NM_000384:exon26:c.C9902G:p.S3301X	0.537	.	.	.	.	Fam47_f_m_aM	0/1;0/0;0/1
chr2	24826055	24826055	A	T	ADCY3	exonic	nonsynonymous SNV	ADCY3:NM_001320613:exon16:c.T2570A:p.F857Y,ADCY3:NM_004036:exon16:c.T2567A:p.F856Y	.	1.017	0.0006	0.0004	2.10E-05	Fam95_f_m_aM_aM_uF	0/0;0/1;0/1;0/1;0/1
chr2	27222298	27222298	C	G	CAD	exonic	nonsynonymous SNV	CAD:NM_001306079:exon4:c.C457G:p.P153A,CAD:NM_004341:exon4:c.C457G:p.P153A	.	1.054	.	.	.	Fam86_f_m_aF	0/1;0/0;0/1
chr2	27224842	27224842	A	G	CAD	exonic	nonsynonymous SNV	CAD:NM_001306079:exon10:c.A1352G:p.Y451C,CAD:NM_004341:exon10:c.A1352G:p.Y451C	.	1.11	7.35E-05	7.45E-05	1.40E-05	Fam48_f_m_aM_uM	0/1;0/0;0/1;0/1
chr2	27239119	27239119	C	T	CAD	exonic	nonsynonymous SNV	CAD:NM_001306079:exon31:c.C4951T:p.L1651F,CAD:NM_004341:exon32:c.C5140T:p.L1714F	.	1.227	.	8.98E-05	4.88E-05	Fam38_f_m_aM	0/0;0/1;0/1
chr2	27239430	27239430	C	T	CAD	exonic	nonsynonymous SNV	CAD:NM_001306079:exon32:c.C5164T:p.R1722C,CAD:NM_004341:exon33:c.C5353T:p.R1785C	.	1.566	.	2.24E-05	1.40E-05	Fam113_f_m_aF_raM_uF	0/0;0/1;0/1;0/0;0/1
chr2	27364775	27364775	C	T	EIF2B4	exonic	nonsynonymous SNV	EIF2B4:NM_001318965:exon11:c.G1378A:p.E460K,EIF2B4:NM_001318968:exon11:c.G730A:p.E244K,EIF2B4:NM_172195:exon11:c.G1375A:p.E459K,EIF2B4:NM_001034116:exon12:c.G1315A:p.E439K,EIF2B4:NM_001318966:exon12:c.G1270A:p.E424K,EIF2B4:NM_001318967:exon12:c.G1222A:p.E408K,EIF2B4:NM_001318969:exon12:c.G697A:p.E233K,EIF2B4:NM_015636:exon12:c.G1312A:p.E438K	.	1.192	.	.	.	Fam113_f_m_aF_raM_uF	0/1;0/0;0/1;0/1;0/1
chr2	27621162	27621162	G	A	ZNF512	exonic	nonsynonymous SNV	ZNF512:NM_001271286:exon13:c.G1318A:p.V440I,ZNF512:NM_001271287:exon13:c.G1174A:p.V392I,ZNF512:NM_001271288:exon13:c.G1174A:p.V392I,ZNF512:NM_001271289:exon13:c.G1090A:p.V364I,ZNF512:NM_032434:exon14:c.G1405A:p.V469I,ZNF512:NM_001271318:exon15:c.G1174A:p.V392I	.	1.307	0.0002	0.0003	0.0002	Fam122_f_m_aM	0/0;0/1;0/1
chr2	28025316	28025316	C	T	BABAM2	exonic	nonsynonymous SNV	BABAM2:NM_001261840:exon5:c.C391T:p.R131C,BABAM2:NM_001329112:exon5:c.C391T:p.R131C,BABAM2:NM_001329114:exon5:c.C391T:p.R131C,BABAM2:NM_004899:exon5:c.C391T:p.R131C,BABAM2:NM_199191:exon5:c.C391T:p.R131C,BABAM2:NM_199192:exon5:c.C391T:p.R131C,BABAM2:NM_001329113:exon6:c.C391T:p.R131C,BABAM2:NM_001329115:exon6:c.C391T:p.R131C,BABAM2:NM_199193:exon6:c.C391T:p.R131C,BABAM2:NM_199194:exon6:c.C391T:p.R131C	.	1.321	0.0003	1.12E-05	1.40E-05	Fam52_f_m_aM	0/0;0/1;0/1
chr2	31266441	31266441	G	A	EHD3	exonic	nonsynonymous SNV	EHD3:NM_014600:exon6:c.G1345A:p.D449N	.	1.171	0.0001	0.0002	0.0001	Fam51_f_m_aM_uF	0/0;0/1;0/1;0/0

chr2	32039314	32039314	G	A	DPY30	exonic	nonsynonymous SNV	DPY30:NM_001321209:exon3:c.C49T:p.P17S,DPY30:NM_001321210:exon3:c.C49T:p.P17S,DPY30:NM_032574:exon3:c.C49T:p.P17S	.	1.928	.	.	.	Fam117_f_m_aM_aF	0/1;0/0;0/1;0/0
chr2	37060632	37060632	C	T	HEATR5B	exonic	nonsynonymous SNV	HEATR5B:NM_019024:exon12:c.G1798A:p.D600N	.	1.058	.	2.23E-05	.	Fam84_f_m_aF	0/0;0/1;0/1
chr2	37064747	37064747	T	A	HEATR5B	exonic	nonsynonymous SNV	HEATR5B:NM_019024:exon10:c.A1577T:p.K526I	.	1.116	0.0003	8.94E-05	3.49E-05	Fam98_f_m_aM	0/1;0/0;0/1
chr2	39006475	39006475	C	G	SOS1	exonic	nonsynonymous SNV	SOS1:NM_005633:exon17:c.G2728C:p.D910H	.	1.667	.	9.81E-05	1.40E-05	Fam53_f_m_aM	0/1;0/0;0/1
chr2	39051253	39051253	A	G	SOS1	exonic	nonsynonymous SNV	SOS1:NM_005633:exon6:c.T755C:p.I252T	.	1.497	.	0.0003	2.79E-05	Fam94_f_m_aM	0/0;0/1;0/1
chr2	43225113	43225113	C	T	ZFP36L2	exonic	nonsynonymous SNV	ZFP36L2:NM_006887:exon2:c.G691A:p.G231R	.	1.251	.	.	6.98E-06	Fam14_f_m_aM_aM	0/1;0/0;0/1;0/0
chr2	50091394	50091394	C	T	NRXN1	exonic	nonsynonymous SNV	NRXN1:NM_001330091:exon3:c.G542A:p.R181H,NRXN1:NM_001330092:exon3:c.G542A:p.R181H,NRXN1:NM_001330097:exon3:c.G542A:p.R181H,NRXN1:NM_138735:exon3:c.G542A:p.R181H,NRXN1:NM_001330087:exon14:c.G3536A:p.R1179H,NRXN1:NM_001330096:exon14:c.G3536A:p.R1179H,NRXN1:NM_001330083:exon15:c.G3581A:p.R1194H,NRXN1:NM_001330084:exon15:c.G3581A:p.R1194H,NRXN1:NM_001330088:exon15:c.G3566A:p.R1189H,NRXN1:NM_001330095:exon17:c.G3596A:p.R1199H,NRXN1:NM_001330077:exon18:c.G3623A:p.R1208H,NRXN1:NM_001330082:exon18:c.G3623A:p.R1208H,NRXN1:NM_001330085:exon18:c.G3620A:p.R1207H,NRXN1:NM_001330094:exon18:c.G3635A:p.R1212H,NRXN1:NM_001330078:exon19:c.G3647A:p.R1216H,NRXN1:NM_001330086:exon19:c.G3647A:p.R1216H,NRXN1:NM_001330093:exon19:c.G3644A:p.R1215H,NRXN1:NM_004801:exon19:c.G3647A:p.R1216H,NRXN1:NM_001135659:exon20:c.G3767A:p.R1256H	.	2.007	.	2.23E-05	.	Fam57_f_m_aM_uF	0/1;0/0;0/1;0/0
chr2	51027514	51027514	C	G	NRXN1	exonic	nonsynonymous SNV	NRXN1:NM_001135659:exon2:c.G760C:p.D254H,NRXN1:NM_001330077:exon2:c.G760C:p.D254H,NRXN1:NM_001330078:exon2:c.G760C:p.D254H,NRXN1:NM_001330079:exon2:c.G760C:p.D254H,NRXN1:NM_001330081:exon2:c.G760C:p.D254H,NRXN1:NM_001330082:exon2:c.G760C:p.D254H,NRXN1:NM_001330083:exon2:c.G760C:p.D254H,NRXN1:NM_001330084:exon2:c.G760C:p.D254H,NRXN1:NM_001330085:exon2:c.G760C:p.D254H,NRXN1:NM_001330086:exon2:c.G760C:p.D254H,NRXN1:NM_001330087:exon2:c.G760C:p.D254H,NRXN1:NM_001330088:exon2:c.G760C:p.D254H,NRXN1:NM_001330089:exon2:c.G760C:p.D254H,NRXN1:NM_001330090:exon2:c.G760C:p.D254H,NRXN1:NM_001330093:exon2:c.G760C:p.D254H,NRXN1:NM_001330094:exon2:c.G760C:p.D254H,NRXN1:NM_001330095:exon2:c.G760C:p.D254H,NRXN1:NM_001330096:exon2:c.G760C:p.D254H,NRXN1:NM_004801:exon2:c.G760C:p.D254H	.	1.449	.	.	.	Fam20_f_m_aM_uF	0/1;0/0;0/1;0/0
chr2	69172373	69172373	A	-	ANTXR1	exonic	stopgain	ANTXR1:NM_053034:exon15:c.1096delA:p.I367*	0.347	.	0.0001	0.0007	8.39E-05	Fam56_f_m_aF_aM	0/1;0/0;0/1;0/0
chr2	69354287	69354287	G	C	GFPT1	exonic	nonsynonymous SNV	GFPT1:NM_001244710:exon9:c.C711G:p.F237L	.	1.316	.	1.25E-05	1.40E-05	Fam112_f_m_aM	0/0;0/1;0/1
chr2	70704435	70704435	G	A	ADD2	exonic	nonsynonymous SNV	ADD2:NM_001185055:exon3:c.C256T:p.L86F,ADD2:NM_001185054:exon4:c.C208T:p.L70F,ADD2:NM_001617:exon4:c.C208T:p.L70F,ADD2:NM_017482:exon4:c.C208T:p.L70F,ADD2:NM_017488:exon4:c.C208T:p.L70F	.	1.14	.	.	.	Fam88_f_m_aF	0/0;0/1;0/1
chr2	70963244	70963244	G	A	ATP6V1B1	exonic	nonsynonymous SNV	ATP6V1B1:NM_001692:exon10:c.G992A:p.R331Q	.	1.105	0.0003	0.0003	4.89E-05	Fam53_f_m_aM	0/0;0/1;0/1
chr2	72135278	72135278	G	A	CYP26B1	exonic	nonsynonymous SNV	CYP26B1:NM_001277742:exon2:c.C346T:p.R116C,CYP26B1:NM_019885:exon3:c.C571T:p.R191C	.	1.135	7.35E-05	0.0003	0.0002	Fam88_f_m_aF	0/1;0/0;0/1
chr2	73112660	73112660	T	C	RAB11FIP5	exonic	nonsynonymous SNV	RAB11FIP5:NM_001371272:exon1:c.A118G:p.S40G,RAB11FIP5:NM_015470:exon1:c.A118G:p.S40G	.	1.424	.	.	.	Fam118_f_m_aM_aF_uM	0/0;0/1;0/1;0/0;0/0
chr2	73233156	73233156	A	G	PRADC1	exonic	nonsynonymous SNV	PRADC1:NM_032319:exon1:c.T5C:p.V2A	.	1.592	0.0005	.	0.0001	Fam106_f_m_aM	0/0;0/1;0/1
chr2	73250364	73250364	G	A	CCT7	exonic	nonsynonymous SNV	CCT7:NM_001009570:exon5:c.G517A:p.G173S,CCT7:NM_001166284:exon8:c.G868A:p.G290S,CCT7:NM_006429:exon10:c.G1129A:p.G377S,CCT7:NM_001166285:exon11:c.G997A:p.G333S	.	1.041	0.0004	0.0006	0.0003	Fam99_f_m_aM_aM	0/0;0/1;0/0;0/1
chr2	73250364	73250364	G	A	CCT7	exonic	nonsynonymous SNV	CCT7:NM_001009570:exon5:c.G517A:p.G173S,CCT7:NM_001166284:exon8:c.G868A:p.G290S,CCT7:NM_006429:exon10:c.G1129A:p.G377S,CCT7:NM_001166285:exon11:c.G997A:p.G333S	.	1.041	0.0004	0.0006	0.0003	Fam10_f_m_aM_uF	0/0;0/1;0/1;0/0
chr2	73292017	73292017	C	T	EGR4	exonic	nonsynonymous SNV	EGR4:NM_001965:exon2:c.G901A:p.G301R	.	1.496	.	0.0001	1.40E-05	Fam66_f_m_aM	0/0;0/1;0/1
chr2	74530335	74530335	C	G	HTRA2	exonic	nonsynonymous SNV	HTRA2:NM_001321727:exon1:c.C329G:p.A110G,HTRA2:NM_001321728:exon1:c.C329G:p.A110G,HTRA2:NM_013247:exon1:c.C329G:p.A110G,HTRA2:NM_145074:exon1:c.C329G:p.A110G	.	1.309	.	3.62E-05	6.99E-06	Fam43_f_m_aM	0/0;0/1;0/1
chr2	74534424	74534424	G	A	LOXL3	exonic	nonsynonymous SNV	LOXL3:NM_001289165:exon7:c.C748T:p.H250Y,LOXL3:NM_001289164:exon9:c.C1396T:p.H466Y,LOXL3:NM_032603:exon11:c.C1831T:p.H611Y	.	1.364	.	.	.	Fam22_f_m_aF_aF_uF	0/1;0/0;0/1;0/1;0/0
chr2	74881851	74881851	G	A	HK2	exonic	nonsynonymous SNV	HK2:NM_000189:exon11:c.G1711A:p.G571R	.	1.257	.	6.18E-05	2.09E-05	Fam15_f_m_aM_aM	0/1;0/0;0/0;0/1
chr2	80393247	80393247	G	A	CTNNA2	exonic	nonsynonymous SNV	CTNNA2:NM_001282599:exon2:c.G130A:p.A44T,CTNNA2:NM_001164883:exon8:c.G1093A:p.A365T,CTNNA2:NM_001282597:exon8:c.G1093A:p.A365T,CTNNA2:NM_001282598:exon8:c.G1195A:p.A399T,CTNNA2:NM_004389:exon8:c.G1093A:p.A365T	.	1.249	.	.	.	Fam11_f_m_aM_uM	0/0;0/1;0/1;0/1

chr2	85599276	85599276	C	G	TMEM150A	exonic	nonsynonymous SNV	TMEM150A:NM_001369917:exon7:c.G616C:p.G206R,TMEM150A:NM_153342:exon7:c.G457C:p.G153R,TMEM150A:NM_001031738:exon8:c.G616C:p.G206R	.	1.318	.	.	.	Fam78_f_m_aF_uM	0/1;0/0;0/1;0/1
chr2	85600008	85600008	G	C	TMEM150A	exonic	nonsynonymous SNV	TMEM150A:NM_001369917:exon5:c.C279G:p.I93M,TMEM150A:NM_153342:exon5:c.C120G:p.I40M,TMEM150A:NM_001031738:exon6:c.C279G:p.I93M	.	1.062	.	.	6.98E-06	Fam91_m_aM_dM_aM_dM	0/0;0/1;0/1;0/0;0/1
chr2	85609535	85609535	C	A	C2orf68	exonic	nonsynonymous SNV	C2orf68:NM_001013649:exon3:c.G278T:p.S93I	.	1.274	.	.	.	Fam94_f_m_aM	0/1;0/0;0/1
chr2	85840389	85840389	C	A	ST3GAL5	exonic	nonsynonymous SNV	ST3GAL5:NM_001354248:exon6:c.G628T:p.V210F,ST3GAL5:NM_001363847:exon6:c.G853T:p.V285F,ST3GAL5:NM_001042437:exon7:c.G943T:p.V315F,ST3GAL5:NM_001354226:exon7:c.G628T:p.V210F,ST3GAL5:NM_001354238:exon7:c.G928T:p.V310F,ST3GAL5:NM_001354247:exon7:c.G289T:p.V97F,ST3GAL5:NM_003896:exon7:c.G1012T:p.V338F,ST3GAL5:NM_001354224:exon8:c.G628T:p.V210F,ST3GAL5:NM_001354227:exon8:c.G928T:p.V310F,ST3GAL5:NM_001354229:exon8:c.G928T:p.V310F,ST3GAL5:NM_001354233:exon8:c.G628T:p.V210F,ST3GAL5:NM_001354223:exon9:c.G628T:p.V210F,ST3GAL5:NM_001354234:exon9:c.G628T:p.V210F	.	1.172	.	.	.	Fam120_f_m_aM_aM	0/0;0/1;0/1;0/1
chr2	86048953	86048953	C	G	POLR1A	exonic	nonsynonymous SNV	POLR1A:NM_015425:exon18:c.G2565C:p.Q855H	.	1.313	7.34E-05	7.86E-05	2.09E-05	Fam90_f_m_aF_dM	0/0;0/1;0/1;0/0
chr2	86054260	86054260	G	C	POLR1A	exonic	nonsynonymous SNV	POLR1A:NM_015425:exon15:c.C2088G:p.I696M	.	1.361	.	.	.	Fam55_f_m_aM_aM_dM	0/0;0/1;0/1;0/0;0/1
chr2	86078260	86078260	C	G	POLR1A	exonic	nonsynonymous SNV	POLR1A:NM_015425:exon10:c.G1111C:p.D371H	.	1.477	.	.	.	Fam62_f_m_aM_aM	0/0;0/1;0/1;0/0
chr2	86098697	86098697	C	T	POLR1A	exonic	nonsynonymous SNV	POLR1A:NM_015425:exon3:c.G346A:p.V116M	.	1.277	7.35E-05	4.50E-05	4.19E-05	Fam67_f_m_aM_uF	0/0;0/1;0/1;0/0
chr2	95382405	95382405	A	G	KCNIP3	exonic	nonsynonymous SNV	KCNIP3:NM_001034914:exon6:c.A506G:p.Y169C,KCNIP3:NM_013434:exon7:c.A584G:p.Y195C	.	1.428	.	.	.	Fam54_f_m_aM_uF	0/0;0/1;0/1;0/1
chr2	96145218	96145218	G	T	DUSP2	exonic	nonsynonymous SNV	DUSP2:NM_004418:exon1:c.C137A:p.A46E	.	2.291	.	.	.	Fam79_f_m_aM_uM	0/0;0/1;0/1;0/1
chr2	96186734	96186734	G	A	STARD7	exonic	nonsynonymous SNV	STARD7:NM_020151:exon8:c.C1109T:p.A370V	.	1.538	.	.	.	Fam49_f_m_aM	0/1;0/0;0/1
chr2	96284568	96284568	G	C	SNRNP200	exonic	stopgain	SNRNP200:NM_014014:exon31:c.C4182G:p.Y1394X	0.312	.	.	.	.	Fam98_f_m_aM	0/1;0/0;0/1
chr2	96286455	96286455	G	A	SNRNP200	exonic	nonsynonymous SNV	SNRNP200:NM_014014:exon29:c.C3859T:p.R1287W	.	2.153	.	.	.	Fam60_f_m_aF	0/1;0/0;0/1
chr2	96296651	96296651	C	T	SNRNP200	exonic	nonsynonymous SNV	SNRNP200:NM_014014:exon13:c.G1556A:p.R519Q	.	1.092	.	1.12E-05	.	Fam63_f_m_aF	0/1;0/0;0/1
chr2	96296979	96296979	C	T	SNRNP200	exonic	nonsynonymous SNV	SNRNP200:NM_014014:exon12:c.G1469A:p.R490H	.	1.175	.	3.27E-05	1.40E-05	Fam71_f_m_aF	0/1;0/0;0/1
chr2	96865743	96865743	G	T	SEMA4C	exonic	nonsynonymous SNV	SEMA4C:NM_017789:exon5:c.C343A:p.R115S	.	1.962	.	.	.	Fam1_f_m_aM	0/1;0/0;0/1
chr2	97735320	97735320	C	T	ZAP70	exonic	nonsynonymous SNV	ZAP70:NM_207519:exon2:c.C232T:p.R78C,ZAP70:NM_001079:exon10:c.C1153T:p.R385C	.	1.863	0.0001	0.0001	4.88E-05	Fam27_f_m_aF_uM_uM	0/0;0/1;0/1;0/0;0/1
chr2	102619936	102619936	G	C	SLC9A2	exonic	nonsynonymous SNV	SLC9A2:NM_003048:exon1:c.G88C:p.G30R	.	1.388	.	.	.	Fam16_f_m_aM_aM	0/0;0/1;0/0;0/1
chr2	105307898	105307898	C	A	TGFBRAP1	exonic	nonsynonymous SNV	TGFBRAP1:NM_001142621:exon2:c.G404T:p.C135F,TGFBRAP1:NM_001328646:exon2:c.G404T:p.C135F,TGFBRAP1:NM_004257:exon2:c.G404T:p.C135F	.	1.207	7.34E-05	.	.	Fam33_f_m_aM_uM	0/0;0/1;0/1;0/1
chr2	106843814	106843814	T	C	ST6GAL2	exonic	nonsynonymous SNV	ST6GAL2:NM_001142351:exon2:c.A164G:p.Q55R,ST6GAL2:NM_001142352:exon2:c.A164G:p.Q55R,ST6GAL2:NM_001322362:exon2:c.A164G:p.Q55R,ST6GAL2:NM_032528:exon2:c.A164G:p.Q55R	.	1.451	.	1.16E-05	1.40E-05	Fam37_f_m_aF_uM	0/0;0/1;0/1;0/0
chr2	107997852	107997852	G	A	SLC5A7	exonic	nonsynonymous SNV	SLC5A7:NM_001305006:exon4:c.G148A:p.V50M,SLC5A7:NM_001305005:exon5:c.G463A:p.V155M,SLC5A7:NM_021815:exon5:c.G463A:p.V155M	.	1.923	.	1.13E-05	2.79E-05	Fam34_f_m_aM_uF	0/0;0/1;0/1;0/1
chr2	108782651	108782651	C	T	RANBP2	exonic	nonsynonymous SNV	RANBP2:NM_006267:exon28:c.C9158T:p.S3053L	.	1.049	.	0.0001	4.19E-05	Fam14_f_m_aM_aM	0/1;0/0;0/1;0/1
chr2	112187348	112187348	C	A	FBLN7	exonic	nonsynonymous SNV	FBLN7:NM_001128165:exon7:c.C1024A:p.R342S,FBLN7:NM_153214:exon8:c.C1162A:p.R388S	.	1.048	.	.	.	Fam100_f_m_aF	0/1;0/0;0/1
chr2	117819792	117819792	G	-	DDX18	exonic	frameshift deletion	DDX18:NM_006773:exon3:c.514delG:p.G172Efs*20	0.496	.	.	.	.	Fam45_f_m_aM_uF	0/0;0/1;0/1;0/0
chr2	119157614	119157614	A	G	C1QL2	exonic	nonsynonymous SNV	C1QL2:NM_182528:exon1:c.T656C:p.M219T	.	1.543	.	.	.	Fam4_f_m_aM	0/1;0/0;0/1
chr2	120222207	120222207	A	G	TMEM185B	exonic	nonsynonymous SNV	TMEM185B:NM_024121:exon1:c.T770C:p.L257S	.	1.013	0.0006	0.0006	0.0002	Fam119_f_m_aM_aM	0/0;0/1;0/1;0/1
chr2	120349458	120349458	G	A	INHBB	exonic	nonsynonymous SNV	INHBB:NM_002193:exon2:c.G808A:p.E270K	.	1.585	.	.	.	Fam13_f_m_aM	0/0;0/1;0/1
chr2	120986563	120986563	G	A	GLI2	exonic	nonsynonymous SNV	GLI2:NM_001371271:exon13:c.G2242A:p.G748R,GLI2:NM_005270:exon13:c.G2242A:p.G748R	.	1.179	.	0.0004	0.0002	Fam25_f_m_aM	0/0;0/1;0/1
chr2	121242406	121242406	C	T	TFCP2L1	exonic	nonsynonymous SNV	TFCP2L1:NM_014553:exon7:c.G721A:p.E241K	.	1.442	.	.	.	Fam60_f_m_aF	0/0;0/1;0/1
chr2	121367762	121367762	G	A	CLASP1	exonic	nonsynonymous SNV	CLASP1:NM_001142274:exon33:c.C3511T:p.R1171W,CLASP1:NM_001207051:exon33:c.C3529T:p.R1177W,CLASP1:NM_001142273:exon34:c.C3535T:p.R1179W,CLASP1:NM_015282:exon34:c.C3712T:p.R1238W	.	1.23	7.35E-05	3.38E-05	2.09E-05	Fam65_f_m_aM_uF_dF	0/1;0/0;0/1;0/1;0/0
chr2	127292740	127292740	T	G	ERCC3	exonic	nonsynonymous SNV	ERCC3:NM_000122:exon3:c.A341C:p.H114P,ERCC3:NM_001303416:exon3:c.A149C:p.H50P,ERCC3:NM_001303418:exon3:c.A149C:p.H50P	.	1.29	.	1.12E-05	.	Fam44_f_m_aM_uF	0/1;0/0;0/1;0/1
chr2	127496086	127496086	C	T	IWS1	exonic	nonsynonymous SNV	IWS1:NM_017969:exon7:c.G1628A:p.R543H	.	2.4	7.34E-05	7.46E-05	6.98E-06	Fam12_f_m_aM_uM_aM	0/0;0/1;0/1;0/0;0/0
chr2	127651499	127651499	G	A	GPR17	exonic	nonsynonymous SNV	GPR17:NM_001161417:exon2:c.G764A:p.R255H,GPR17:NM_001161416:exon3:c.G764A:p.R255H,GPR17:NM_005291:exon3:c.G848A:p.R283H,GPR17:NM_001161415:exon4:c.G848A:p.R283H	.	1.008	.	0.0001	2.09E-05	Fam28_f_m_aF_uF	0/0;0/1;0/1;0/0
chr2	127708860	127708860	G	A	WDR33	exonic	nonsynonymous SNV	WDR33:NM_018383:exon21:c.C3598T:p.R1200C	.	1.21	0.0003	7.49E-05	4.19E-05	Fam121_f_m_aF_aM	0/0;0/1;0/1;0/1
chr2	132417658	132417658	A	G	GPR39	exonic	nonsynonymous SNV	GPR39:NM_001508:exon1:c.A616G:p.N206D	.	1.131	.	1.12E-05	6.98E-06	Fam4_f_m_aM	0/1;0/0;0/1
chr2	134349873	134349873	C	G	MGAT5	exonic	nonsynonymous SNV	MGAT5:NM_002410:exon9:c.C1181G:p.S394W,MGAT5:NM_001371457:exon10:c.C1181G:p.S394W	.	1.909	.	.	6.98E-06	Fam2_f_m_aF	0/1;0/0;0/1
chr2	135798074	135798074	G	A	LCT	exonic	nonsynonymous SNV	LCT:NM_002299:exon13:c.C4931T:p.T1644M	.	1.611	.	9.80E-05	1.40E-05	Fam25_f_m_aM	0/1;0/0;0/1
chr2	135808933	135808933	T	G	LCT	exonic	nonsynonymous SNV	LCT:NM_002299:exon8:c.A3414C:p.R1138S	.	1.565	.	.	.	Fam95_f_m_aM_aM_uF	0/0;0/1;0/1;0/1;0/0

chr2	135809642	135809642	G	T	LCT	exonic	nonsynonymous SNV	LCT:NM_002299:exon8:c.C2705A:p.T902K	.	1.416	.	3.28E-05	6.98E-06	Fam114_f_m_aM	0/1;0/0;0/1
chr2	147917386	147917386	T	G	ACVR2A	exonic	nonsynonymous SNV	ACVR2A:NM_001278580:exon6:c.T452G:p.V151G,ACVR2A:NM_001616:exon6:c.T776G:p.V259G,ACVR2A:NM_001278579:exon7:c.T776G:p.V259G	.	1.051	.	.	.	Fam103_f_m_aM	0/0;0/1;0/1
chr2	148994424	148994424	G	A	KIF5C	exonic	nonsynonymous SNV	KIF5C:NM_004522:exon17:c.G1909A:p.E637K	.	1.21	.	.	3.49E-05	Fam34_f_m_aM_uF	0/1;0/0;0/1;0/0
chr2	150470051	150470051	G	C	RND3	exonic	nonsynonymous SNV	RND3:NM_001254738:exon5:c.C671G:p.P224R,RND3:NM_005168:exon6:c.C671G:p.P224R	.	1.228	.	1.12E-05	2.09E-05	Fam84_f_m_aF	0/1;0/0;0/1
chr2	151667802	151667802	A	T	NEB	splicing	.	.	0.597	.	.	.	.	Fam69_f_m_aM	0/1;0/0;0/1
chr2	151860740	151860740	A	G	CACNB4	exonic	nonsynonymous SNV	CACNB4:NM_001005747:exon9:c.T737C:p.I246T,CACNB4:NM_001330114:exon9:c.T185C:p.I62T,CACNB4:NM_000726:exon10:c.T839C:p.I280T,CACNB4:NM_001005746:exon10:c.T785C:p.I262T,CACNB4:NM_001145798:exon10:c.T839C:p.I280T,CACNB4:NM_001320722:exon10:c.T698C:p.I233T,CACNB4:NM_001330118:exon10:c.T698C:p.I233T,CACNB4:NM_001330117:exon11:c.T281C:p.I94T	.	1.692	.	0.0003	6.98E-06	Fam98_f_m_aM	0/1;0/0;0/1
chr2	152626621	152626621	G	C	FMNL2	exonic	nonsynonymous SNV	FMNL2:NM_052905:exon17:c.G2059C:p.G687R	.	1.2	.	0.0002	6.29E-05	Fam51_f_m_aM_uF	0/1;0/0;0/1;0/1
chr2	161203491	161203491	A	G	TANK	exonic	nonsynonymous SNV	TANK:NM_001199135:exon3:c.A104G:p.E35G,TANK:NM_004180:exon3:c.A104G:p.E35G,TANK:NM_133484:exon3:c.A104G:p.E35G	.	1.077	.	.	.	Fam67_f_m_aM_uF	0/0;0/1;0/1;0/1
chr2	165090286	165090286	T	C	SCN3A	exonic	nonsynonymous SNV	SCN3A:NM_001081676:exon28:c.A5720G:p.E1907G,SCN3A:NM_001081677:exon28:c.A5720G:p.E1907G,SCN3A:NM_006922:exon28:c.A5867G:p.E1956G	.	1.813	.	.	.	Fam29_f_m_aF_uM	0/1;0/0;0/1;0/1
chr2	165091265	165091265	G	A	SCN3A	exonic	stopgain	SCN3A:NM_001081676:exon28:c.C4741T:p.R1581X,SCN3A:NM_001081677:exon28:c.C4741T:p.R1581X,SCN3A:NM_006922:exon28:c.C4888T:p.R1630X	0.348	.	.	.	.	Fam40_f_m_aM_aM	0/0;0/0;0/1;0/0
chr2	165374892	165374892	C	T	SCN2A	exonic	stopgain	SCN2A:NM_001040142:exon22:c.C4180T:p.Q1394X,SCN2A:NM_001371246:exon22:c.C4180T:p.Q1394X,SCN2A:NM_001371247:exon22:c.C4180T:p.Q1394X,SCN2A:NM_021007:exon22:c.C4180T:p.Q1394X,SCN2A:NM_001040143:exon23:c.C4180T:p.Q1394X	0.161	.	.	.	.	Fam113_f_m_aF_raM_uF	0/0;0/0;0/0;0/1;0/0
chr2	166043728	166043728	A	C	SCN1A	exonic	nonsynonymous SNV	SCN1A:NM_001353948:exon12:c.T1984G:p.S662A,SCN1A:NM_001353949:exon12:c.T1984G:p.S662A,SCN1A:NM_001353951:exon12:c.T1984G:p.S662A,SCN1A:NM_001353955:exon12:c.T1981G:p.S661A,SCN1A:NM_001202435:exon13:c.T1984G:p.S662A,SCN1A:NM_001353950:exon13:c.T1984G:p.S662A,SCN1A:NM_001353952:exon13:c.T1984G:p.S662A,SCN1A:NM_001353954:exon13:c.T1981G:p.S661A,SCN1A:NM_001165963:exon14:c.T1984G:p.S662A,SCN1A:NM_006920:exon14:c.T1984G:p.S662A	.	1.069	.	.	.	Fam43_f_m_aM	0/1;0/0;0/1
chr2	166073542	166073542	C	G	SCN1A	exonic	nonsynonymous SNV	SCN1A:NM_001353948:exon2:c.G80C:p.R27T,SCN1A:NM_001353949:exon2:c.G80C:p.R27T,SCN1A:NM_001353951:exon2:c.G80C:p.R27T,SCN1A:NM_001353955:exon2:c.G80C:p.R27T,SCN1A:NM_001353957:exon2:c.G80C:p.R27T,SCN1A:NM_001165964:exon3:c.G80C:p.R27T,SCN1A:NM_001202435:exon3:c.G80C:p.R27T,SCN1A:NM_001353950:exon3:c.G80C:p.R27T,SCN1A:NM_001353952:exon3:c.G80C:p.R27T,SCN1A:NM_001353954:exon3:c.G80C:p.R27T,SCN1A:NM_001353958:exon3:c.G80C:p.R27T,SCN1A:NM_001353960:exon3:c.G80C:p.R27T,SCN1A:NM_001165963:exon4:c.G80C:p.R27T,SCN1A:NM_006920:exon4:c.G80C:p.R27T	.	1.001	.	0.0001	5.59E-05	Fam69_f_m_aM	0/0;0/1;0/1
chr2	168167368	168167368	C	T	STK39	exonic	nonsynonymous SNV	STK39:NM_013233:exon3:c.G361A:p.V121I	.	1.013	.	0.0003	7.68E-05	Fam47_f_m_aM	0/0;0/1;0/1
chr2	168247235	168247235	C	G	STK39	exonic	nonsynonymous SNV	STK39:NM_013233:exon1:c.G201C:p.E67D	.	1.066	.	.	.	Fam39_f_m_aM	0/0;0/1;0/1
chr2	169170603	169170603	C	A	LRP2	exonic	nonsynonymous SNV	LRP2:NM_004525:exon59:c.G11328T:p.W3776C	.	1.101	7.34E-05	6.70E-05	2.79E-05	Fam38_f_m_aM	0/0;0/1;0/1
chr2	170853871	170853871	A	T	GAD1	splicing	.	.	0.569	.	.	.	.	Fam81_f_m_aM_uM	0/1;0/0;0/1;0/1
chr2	170853966	170853966	A	G	GAD1	exonic	nonsynonymous SNV	GAD1:NM_000817:exon14:c.A1357G:p.I453V	.	1.391	.	.	.	Fam24_f_m_aM_aM	0/1;0/0;0/0;0/1
chr2	171787636	171787636	C	A	SLC25A12	exonic	nonsynonymous SNV	SLC25A12:NM_003705:exon17:c.G1770T:p.Q590H	.	1.509	.	.	.	Fam27_f_m_aF_uM_uM	0/1;0/0;0/1;0/1;0/0
chr2	172487808	172487808	G	A	ITGA6	splicing	.	.	0.52	.	.	1.12E-05	.	Fam99_f_m_aM_aM	0/0;0/1;0/0;0/1
chr2	172491261	172491261	C	T	ITGA6	exonic	nonsynonymous SNV	ITGA6:NM_000210:exon22:c.C2819T:p.P940L,ITGA6:NM_001079818:exon22:c.C2819T:p.P940L,ITGA6:NM_001316306:exon22:c.C2462T:p.P821L,ITGA6:NM_001365529:exon22:c.C2774T:p.P925L,ITGA6:NM_001365530:exon22:c.C2774T:p.P925L	.	1.232	0.0009	0.001	0.0009	Fam12_f_m_aM_uM_aM	0/1;0/0;0/1;0/1;0/1
chr2	172985472	172985472	C	T	RAPGEF4	exonic	nonsynonymous SNV	RAPGEF4:NM_001282901:exon6:c.C469T:p.H157Y,RAPGEF4:NM_001282900:exon8:c.C616T:p.H206Y,RAPGEF4:NM_001100397:exon9:c.C697T:p.H233Y,RAPGEF4:NM_001282899:exon9:c.C670T:p.H224Y,RAPGEF4:NM_007023:exon12:c.C1129T:p.H377Y	.	1.129	.	.	.	Fam42_f_m_aM_uF	0/1;0/0;0/1;0/0
chr2	174754320	174754320	A	G	CHRNA1	exonic	nonsynonymous SNV	CHRNA1:NM_000079:exon5:c.T439C:p.Y147H,CHRNA1:NM_001039523:exon6:c.T514C:p.Y172H	.	1.039	.	1.12E-05	6.98E-06	Fam64_f_m_aM	0/1;0/0;0/1
chr2	176122910	176122910	C	T	HOXD9	exonic	nonsynonymous SNV	HOXD9:NM_014213:exon1:c.C142T:p.R48W	.	1.509	.	.	.	Fam39_f_m_aM	0/0;0/1;0/1
chr2	176123076	176123076	C	G	HOXD9	exonic	nonsynonymous SNV	HOXD9:NM_014213:exon1:c.C308G:p.P103R	.	1.417	.	3.53E-05	.	Fam21_f_m_aM_uM	0/1;0/0;0/1;0/0
chr2	177482157	177482157	G	A	AGPS	exonic	nonsynonymous SNV	AGPS:NM_003659:exon11:c.G1204A:p.V402I	.	1.137	7.40E-05	0.0005	4.20E-05	Fam73_f_m_aF_dM	0/1;0/0;0/1;0/0

chr2	178374007	178374007	G	C	OSBPL6	exonic	nonsynonymous SNV	OSBPL6:NM_001201481:exon14:c.G1420C:p.A474P,OSBPL6:NM_001201482:exon14:c.G1405C:p.A469P,OSBPL6:NM_145739:exon14:c.G1525C:p.A509P,OSBPL6:NM_032523:exon15:c.G1513C:p.A505P,OSBPL6:NM_001201480:exon16:c.G1588C:p.A530P	.	1.143	.	6.56E-05	.	Fam4_f_m_aM	0/0;0/1;0/1
chr2	178384178	178384178	T	C	OSBPL6	splicing	.	.	0.476	.	.	.	.	Fam117_f_m_aM_aF	0/1;0/0;0/0;0/1
chr2	179172200	179172200	C	T	SESTD1	exonic	nonsynonymous SNV	SESTD1:NM_178123:exon5:c.G289A:p.V97M	.	1.532	.	.	.	Fam74_f_m_aM_uM	0/1;0/0;0/1;0/1
chr2	181498664	181498664	G	C	ITGA4	exonic	nonsynonymous SNV	ITGA4:NM_000885:exon15:c.G1582C:p.E528Q	.	1.11	0.0001	6.75E-05	0.0003	Fam53_f_m_aM	0/0;0/1;0/1
chr2	182925721	182925721	C	T	NCKAP1	exonic	nonsynonymous SNV	NCKAP1:NM_013436:exon31:c.G3368A:p.S1123N,NCKAP1:NM_205842:exon32:c.G3386A:p.S1129N	.	1.117	.	.	6.99E-06	Fam15_f_m_aM_aM	0/0;0/1;0/1;0/0
chr2	187360657	187360657	A	G	CALCRL	exonic	nonsynonymous SNV	CALCRL:NM_001271751:exon9:c.T722C:p.I241T,CALCRL:NM_005795:exon10:c.T722C:p.I241T,CALCRL:NM_001369434:exon11:c.T722C:p.I241T,CALCRL:NM_001369435:exon11:c.T722C:p.I241T	.	1.422	.	.	.	Fam119_f_m_aM_aM	0/0;0/1;0/1;0/0
chr2	189179604	189179604	T	C	COL5A2	exonic	startloss	COL5A2:NM_000393:exon1:c.A1G:p.M2del	0.106	.	.	6.87E-05	1.40E-05	Fam40_f_m_aM_aM	0/0;0/1;0/1;0/0
chr2	190659385	190659385	A	T	NAB1	exonic	nonsynonymous SNV	NAB1:NM_001321314:exon1:c.A209T:p.K70M,NAB1:NM_001321315:exon1:c.A209T:p.K70M,NAB1:NM_001321312:exon2:c.A209T:p.K70M,NAB1:NM_001321313:exon2:c.A209T:p.K70M,NAB1:NM_005966:exon4:c.A209T:p.K70M	.	1.096	.	.	.	Fam13_f_m_aM	0/0;0/1;0/1
chr2	190982452	190982452	G	A	STAT1	exonic	stopgain	STAT1:NM_007315:exon18:c.C1513T:p.Q505X,STAT1:NM_139266:exon18:c.C1513T:p.Q505X	0.271	.	.	.	.	Fam37_f_m_aF_uM	0/0;0/1;0/1;0/0
chr2	191341485	191341485	A	G	MYO1B	exonic	nonsynonymous SNV	MYO1B:NM_001130158:exon5:c.A371G:p.Y124C,MYO1B:NM_001161819:exon5:c.A371G:p.Y124C,MYO1B:NM_001330237:exon5:c.A371G:p.Y124C,MYO1B:NM_001330238:exon5:c.A371G:p.Y124C,MYO1B:NM_012223:exon5:c.A371G:p.Y124C	.	1.247	.	2.24E-05	2.09E-05	Fam75_f_m_aM	0/0;0/1;0/1
chr2	191846602	191846602	G	T	CAVIN2	exonic	nonsynonymous SNV	CAVIN2:NM_004657:exon1:c.C324A:p.S108R	.	1.014	.	.	.	Fam65_f_m_aM_uF_dF	0/1;0/0;0/1;0/1;0/1
chr2	195728251	195728251	C	T	SLC39A10	exonic	nonsynonymous SNV	SLC39A10:NM_001127257:exon9:c.C2239T:p.L747F,SLC39A10:NM_020342:exon9:c.C2239T:p.L747F	.	1.361	.	.	.	Fam110_f_m_aM_aM_uMs	0/0;0/1;0/1;0/0;0/0
chr2	196253944	196253944	C	T	HECW2	exonic	nonsynonymous SNV	HECW2:NM_001304840:exon17:c.G2437A:p.V813M,HECW2:NM_001348768:exon19:c.G3505A:p.V1169M,HECW2:NM_020760:exon19:c.G3505A:p.V1169M	.	1.129	7.34E-05	0.0001	6.98E-06	Fam118_f_m_aM_aF_uM	0/0;0/1;0/1;0/1;0/0
chr2	196271199	196271199	G	A	HECW2	exonic	nonsynonymous SNV	HECW2:NM_001304840:exon15:c.C2261T:p.S754L,HECW2:NM_001348768:exon17:c.C3329T:p.S1110L,HECW2:NM_020760:exon17:c.C3329T:p.S1110L	.	1.049	.	.	.	Fam85_f_m_aM_aM	0/0;0/1;0/0;0/1
chr2	196317282	196317282	A	G	HECW2	exonic	nonsynonymous SNV	HECW2:NM_001304840:exon8:c.T1358C:p.L453P,HECW2:NM_001348768:exon10:c.T2426C:p.L809P,HECW2:NM_020760:exon10:c.T2426C:p.L809P	.	1.317	.	.	.	Fam68_f_m_aF_uF_uM	0/0;0/0;0/1;0/0;0/0
chr2	198084406	198084406	C	T	PLCL1	exonic	nonsynonymous SNV	PLCL1:NM_006226:exon2:c.C889T:p.R297C	.	1.042	.	1.12E-05	6.98E-06	Fam37_f_m_aF_uM	0/0;0/1;0/1;0/1
chr2	198084752	198084752	C	G	PLCL1	exonic	nonsynonymous SNV	PLCL1:NM_006226:exon2:c.C1235G:p.S412C	.	1.036	.	.	.	Fam24_f_m_aM_aM	0/0;0/1;0/1;0/1
chr2	198085060	198085060	C	T	PLCL1	exonic	nonsynonymous SNV	PLCL1:NM_006226:exon2:c.C1543T:p.L515F	.	1.094	.	1.12E-05	.	Fam89_f_m_aM	0/1;0/0;0/1
chr2	199959809	199959809	C	T	MAIP1	exonic	nonsynonymous SNV	MAIP1:NM_001369399:exon4:c.C578T:p.A193V,MAIP1:NM_024520:exon4:c.C578T:p.A193V	.	1.206	.	.	6.98E-06	Fam19_f_m_aM	0/0;0/1;0/1
chr2	200981406	200981406	C	T	FAM126B	exonic	nonsynonymous SNV	FAM126B:NM_001321618:exon11:c.G1457A:p.R486Q,FAM126B:NM_001321621:exon11:c.G1457A:p.R486Q,FAM126B:NM_001321619:exon12:c.G1457A:p.R486Q,FAM126B:NM_001321624:exon12:c.G1625A:p.R542Q,FAM126B:NM_173822:exon12:c.G1457A:p.R486Q,FAM126B:NM_001321622:exon13:c.G1457A:p.R486Q,FAM126B:NM_001321623:exon13:c.G1625A:p.R542Q,FAM126B:NM_001321625:exon13:c.G1625A:p.R542Q,FAM126B:NM_001321626:exon13:c.G1625A:p.R542Q,FAM126B:NM_001321629:exon13:c.G1211A:p.R404Q,FAM126B:NM_001321627:exon14:c.G1379A:p.R460Q,FAM126B:NM_001321628:exon14:c.G1379A:p.R460Q	.	1.198	.	.	6.98E-06	Fam33_f_m_aM_uM	0/1;0/0;0/1;0/1
chr2	201477663	201477663	T	C	STRADB	exonic	nonsynonymous SNV	STRADB:NM_001206864:exon8:c.T593C:p.V198A,STRADB:NM_018571:exon8:c.T593C:p.V198A	.	1.721	.	2.23E-05	1.40E-05	Fam106_f_m_aM	0/1;0/0;0/1
chr2	202034999	202034999	C	T	FZD7	exonic	nonsynonymous SNV	FZD7:NM_003507:exon1:c.C352T:p.P118S	.	2.117	.	.	6.98E-06	Fam36_f_m_aM_uM	0/1;0/0;0/1;0/0
chr2	202520142	202520142	G	A	BMPR2	exonic	nonsynonymous SNV	BMPR2:NM_001204:exon7:c.G908A:p.R303H	.	1.147	.	0.0001	4.94E-05	Fam88_f_m_aF	0/1;0/0;0/1
chr2	202552887	202552887	C	T	BMPR2	exonic	nonsynonymous SNV	BMPR2:NM_001204:exon11:c.C1585T:p.R529C	.	1.263	.	9.80E-05	6.98E-06	Fam35_f_m_aF_uM	0/1;0/0;0/1;0/0
chr2	209976983	209976983	G	A	UNC80	exonic	nonsynonymous SNV	UNC80:NM_032504:exon57:c.G8645A:p.R2882Q,UNC80:NM_182587:exon57:c.G8630A:p.R2877Q	.	1.12	.	.	6.98E-06	Fam120_f_m_aM_aM	0/0;0/1;0/1;0/1
chr2	209978684	209978684	C	T	UNC80	exonic	nonsynonymous SNV	UNC80:NM_032504:exon58:c.C8896T:p.R2966W,UNC80:NM_182587:exon58:c.C8881T:p.R2961W	.	1.076	0.0006	0.0001	6.98E-06	Fam43_f_m_aM	0/0;0/1;0/1
chr2	216660707	216660707	A	G	IGFBP2	exonic	nonsynonymous SNV	IGFBP2:NM_000597:exon2:c.A593G:p.Q198R,IGFBP2:NM_001313990:exon2:c.A161G:p.Q54R,IGFBP2:NM_001313992:exon2:c.A95G:p.Q32R,IGFBP2:NM_001313993:exon2:c.A95G:p.Q32R	.	1.69	.	.	.	Fam83_f_m_aF	0/0;0/1;0/1
chr2	218266493	218266493	G	A	AAMP	exonic	nonsynonymous SNV	AAMP:NM_001087:exon5:c.C629T:p.T210I,AAMP:NM_001302545:exon5:c.C632T:p.T211I	.	1.407	.	.	.	Fam56_f_m_aF_aM	0/1;0/0;0/1;0/1
chr2	219418678	219418678	C	A	DES	exonic	nonsynonymous SNV	DES:NM_001927:exon1:c.C216A:p.S72R	.	1.734	.	0.0003	7.68E-05	Fam67_f_m_aM_uF	0/1;0/0;0/1;0/1
chr2	219469011	219469011	T	C	SPEG	exonic	nonsynonymous SNV	SPEG:NM_005876:exon12:c.T3454C:p.Y1152H	.	1.9	.	.	6.98E-06	Fam60_f_m_aF	0/1;0/0;0/1
chr2	219469158	219469158	C	T	SPEG	exonic	nonsynonymous SNV	SPEG:NM_005876:exon13:c.C3494T:p.S1165L	.	1.509	0.0001	6.55E-05	4.19E-05	Fam22_f_m_aF_aF_uF	0/0;0/1;0/1;0/1;0/0
chr2	219483098	219483098	C	T	SPEG	exonic	nonsynonymous SNV	SPEG:NM_005876:exon30:c.C5635T:p.R1879C	.	1.615	0.0003	0.0005	0.0002	Fam118_f_m_aM_aF_uM	0/0;0/1;0/1;0/1;0/0
chr2	219506398	219506398	C	A	GMPPA	exonic	nonsynonymous SNV	GMPPA:NM_013335:exon12:c.C1138A:p.L380M,GMPPA:NM_205847:exon12:c.C1138A:p.L380M	.	1.178	.	.	.	Fam86_f_m_aF	0/1;0/0;0/1

chr2	219531821	219531821	C	T	ASIC4	exonic	nonsynonymous SNV	ASIC4:NM_018674:exon2:c.C1027T:p.R343W,ASIC4:NM_182847:exon2:c.C1027T:p.R343W	.	1.001	.		7.84E-05	0.0001	Fam51_f_m_aM_uF	0/1;0/0;0/1;0/1
chr2	219570911	219570911	C	T	OBSL1	exonic	nonsynonymous SNV	OBSL1:NM_001173408:exon1:c.G322A:p.D108N,OBSL1:NM_001173431:exon1:c.G322A:p.D108N,OBSL1:NM_015311:exon1:c.G322A:p.D108N	.	1.287	.	.	.		Fam12_f_m_aM_uM_aM	0/1;0/0;0/0;0/1;0/1
chr2	219571203	219571203	G	C	OBSL1	exonic	nonsynonymous SNV	OBSL1:NM_001173408:exon1:c.C30G:p.S10R,OBSL1:NM_001173431:exon1:c.C30G:p.S10R,OBSL1:NM_015311:exon1:c.C30G:p.S10R	.	1.562	.	.	.		Fam82_f_m_aM_uF	0/0;0/1;0/1;0/0
chr2	222220315	222220315	G	A	PAX3	exonic	nonsynonymous SNV	PAX3:NM_001127366:exon7:c.C995T:p.P332L,PAX3:NM_181457:exon7:c.C998T:p.P333L,PAX3:NM_181458:exon7:c.C998T:p.P333L,PAX3:NM_181459:exon7:c.C998T:p.P333L,PAX3:NM_181460:exon7:c.C998T:p.P333L,PAX3:NM_181461:exon7:c.C998T:p.P333L	.	1.325	.		6.55E-05	6.98E-06	Fam12_f_m_aM_uM_aM	0/1;0/0;0/1;0/0;0/0
chr2	223991844	223991844	T	C	SERPINE2	exonic	nonsynonymous SNV	SERPINE2:NM_001136528:exon4:c.A644G:p.Y215C,SERPINE2:NM_001136530:exon4:c.A680G:p.Y227C,SERPINE2:NM_006216:exon4:c.A644G:p.Y215C	.	1.12	.		9.89E-05	.	Fam9_f_m_aM_dM_uF	0/1;0/0;0/1;0/0;0/1
chr2	229418193	229418193	-	TCAT	DNER	exonic	stopgain	DNER:NM_139072:exon9:c.1523_1524insATGA:p.C509*	0.562	.	.	.	.		Fam122_f_m_aM	0/0;0/1;0/1
chr2	231397747	231397747	C	T	B3GNT7	exonic	nonsynonymous SNV	B3GNT7:NM_145236:exon2:c.C28T:p.R10W	.	1.285	.		7.73E-05	.	Fam96_f_m_aM_uF	0/0;0/1;0/1;0/1
chr2	232263211	232263211	T	G	DIS3L2	exonic	nonsynonymous SNV	DIS3L2:NM_001257281:exon13:c.T1430G:p.L477R,DIS3L2:NM_152383:exon13:c.T1430G:p.L477R	.	1.009	7.34E-05		0.0001	0.0002	Fam115_f_m_aF_aM_aF	0/1;0/0;0/1;0/0;0/1
chr2	232768789	232768789	C	T	KCNJ13	exonic	nonsynonymous SNV	KCNJ13:NM_001172417:exon3:c.G245A:p.R82Q,KCNJ13:NM_002242:exon3:c.G485A:p.R162Q	.	1.536	.		0.0007	0.0001	Fam98_f_m_aM	0/1;0/0;0/1
chr2	233060513	233060513	G	A	INPP5D	exonic	nonsynonymous SNV	INPP5D:NM_001017915:exon1:c.G35A:p.R12H,INPP5D:NM_005541:exon1:c.G35A:p.R12H	.	3.005	.	.		6.98E-06	Fam68_f_m_aF_uF_uM	0/1;0/0;0/1;0/0;0/1
chr2	233204413	233204413	G	A	INPP5D	exonic	nonsynonymous SNV	INPP5D:NM_001017915:exon26:c.G3263A:p.C1088Y,INPP5D:NM_005541:exon26:c.G3260A:p.C1087Y	.	1.007	.	.	.		Fam66_f_m_aM	0/1;0/0;0/1
chr2	233464251	233464251	C	T	DGKD	exonic	nonsynonymous SNV	DGKD:NM_003648:exon26:c.C3142T:p.L1048F,DGKD:NM_152879:exon27:c.C3274T:p.L1092F	.	1.286	0.0004		0.0005	0.0002	Fam16_f_m_aM_aM	0/0;0/1;0/1;0/0
chr2	235717639	235717639	C	T	AGAP1	exonic	nonsynonymous SNV	AGAP1:NM_001037131:exon3:c.C305T:p.P102L,AGAP1:NM_001244888:exon3:c.C305T:p.P102L,AGAP1:NM_014914:exon3:c.C305T:p.P102L	.	1.262	.		1.16E-05	6.98E-06	Fam107_f_m_aM	0/1;0/0;0/1
chr2	236120347	236120347	G	T	AGAP1	exonic	nonsynonymous SNV	AGAP1:NM_014914:exon16:c.G2111T:p.R704L,AGAP1:NM_001037131:exon17:c.G2270T:p.R757L	.	1.374	.		5.71E-05	2.79E-05	Fam44_f_m_aM_uF	0/0;0/1;0/1;0/0
chr2	236123941	236123941	G	A	AGAP1	exonic	nonsynonymous SNV	AGAP1:NM_014914:exon17:c.G2234A:p.R745Q,AGAP1:NM_001037131:exon18:c.G2393A:p.R798Q	.	1.347	0.0004		0.0008	0.0004	Fam52_f_m_aM	0/0;0/1;0/1
chr2	236123941	236123941	G	A	AGAP1	exonic	nonsynonymous SNV	AGAP1:NM_014914:exon17:c.G2234A:p.R745Q,AGAP1:NM_001037131:exon18:c.G2393A:p.R798Q	.	1.347	0.0004		0.0008	0.0004	Fam72_f_m_aF_uF	0/0;0/1;0/1;0/0
chr2	236214616	236214616	C	T	ASB18	exonic	nonsynonymous SNV	ASB18:NM_212556:exon4:c.G847A:p.G283R	.	1.834	0.0003	.		0.0002	Fam67_f_m_aM_uF	0/0;0/1;0/1;0/1
chr2	236237719	236237719	A	G	ASB18	exonic	nonsynonymous SNV	ASB18:NM_212556:exon3:c.T566C:p.L189P	.	2.798	.	.	.		Fam55_f_m_aM_aM_dM	0/1;0/0;0/1;0/1;0/0
chr2	238239095	238239095	C	T	HES6	exonic	nonsynonymous SNV	HES6:NM_001142853:exon4:c.G401A:p.R134H,HES6:NM_018645:exon4:c.G407A:p.R136H	.	1.518	.		3.47E-05	1.40E-05	Fam39_f_m_aM	0/1;0/0;0/1
chr2	238848286	238848286	G	A	TWIST2	exonic	nonsynonymous SNV	TWIST2:NM_001271893:exon1:c.G71A:p.R24K,TWIST2:NM_057179:exon1:c.G71A:p.R24K	.	1.619	7.37E-05		4.64E-05	2.10E-05	Fam11_f_m_aM_uM	0/1;0/0;0/1;0/1
chr2	239189859	239189859	C	T	HDAC4	exonic	nonsynonymous SNV	HDAC4:NM_006037:exon4:c.G313A:p.E105K	.	1.346	.	.		6.98E-06	Fam96_f_m_aM_uF	0/0;0/1;0/1;0/0
chr2	240723546	240723546	C	T	KIF1A	exonic	nonsynonymous SNV	KIF1A:NM_004321:exon40:c.G4028A:p.R1343Q,KIF1A:NM_001320705:exon41:c.G4055A:p.R1352Q,KIF1A:NM_001330290:exon41:c.G4130A:p.R1377Q,KIF1A:NM_001244008:exon42:c.G4331A:p.R1444Q,KIF1A:NM_001330289:exon42:c.G4082A:p.R1361Q	.	1.377	.		1.92E-05	.	Fam117_f_m_aM_aF	0/1;0/0;0/1;0/1
chr2	240746168	240746168	C	T	KIF1A	exonic	nonsynonymous SNV	KIF1A:NM_004321:exon28:c.G2770A:p.E924K,KIF1A:NM_001320705:exon29:c.G2797A:p.E933K,KIF1A:NM_001330289:exon29:c.G2797A:p.E933K,KIF1A:NM_001330290:exon29:c.G2872A:p.E958K,KIF1A:NM_001244008:exon30:c.G3073A:p.E1025K	.	1.372	.		0.0003	.	Fam116_f_m_aM_aF	0/0;0/1;0/1;0/1
chr2	240998853	240998853	G	C	SNED1	exonic	nonsynonymous SNV	SNED1:NM_001080437:exon1:c.G16C:p.A6P	.	2.287	0.0002	.		5.66E-05	Fam83_f_m_aF	0/1;0/0;0/1
chr2	241182653	241182653	G	A	PPP1R7	exonic	nonsynonymous SNV	PPP1R7:NM_001282409:exon9:c.G784A:p.D262N,PPP1R7:NM_001282413:exon9:c.G736A:p.D246N,PPP1R7:NM_001282412:exon10:c.G865A:p.D289N,PPP1R7:NM_002712:exon10:c.G913A:p.D305N	.	1.802	.		3.28E-05	.	Fam6_f_m_aM	0/0;0/1;0/1
chr2	241495666	241495666	C	T	STK25	exonic	nonsynonymous SNV	STK25:NM_001271978:exon11:c.G1277A:p.R426H,STK25:NM_001271979:exon11:c.G1046A:p.R349H,STK25:NM_001271980:exon11:c.G1046A:p.R349H,STK25:NM_001282305:exon11:c.G995A:p.R332H,STK25:NM_001282307:exon11:c.G995A:p.R332H,STK25:NM_001282308:exon11:c.G995A:p.R332H,STK25:NM_001271977:exon12:c.G1277A:p.R426H,STK25:NM_001282306:exon12:c.G1055A:p.R352H,STK25:NM_006374:exon12:c.G1277A:p.R426H	.	1.858	0.0004		0.0002	0.0001	Fam74_f_m_aM_uM	0/1;0/0;0/1;0/0
chr2	241559533	241559533	C	T	BOK	exonic	nonsynonymous SNV	BOK:NM_032515:exon2:c.C50T:p.A17V	.	1.302	.	.	.		Fam91_m_aM_dM_aM_dM	0/1;0/0;0/1;0/1;0/1
chr2	241678528	241678528	T	C	DTYMK	exonic	nonsynonymous SNV	DTYMK:NM_001320902:exon3:c.A323G:p.Y108C,DTYMK:NM_001320903:exon3:c.A289G:p.M97V,DTYMK:NM_001165031:exon4:c.A380G:p.Y127C,DTYMK:NM_001320904:exon4:c.A395G:p.Y132C,DTYMK:NM_012145:exon4:c.A452G:p.Y151C,DTYMK:NM_001320905:exon5:c.A569G:p.Y190C	.	1.075	.		1.12E-05	1.40E-05	Fam49_f_m_aM	0/1;0/0;0/1
chr2	241742460	241742460	G	A	D2HGDH	exonic	nonsynonymous SNV	D2HGDH:NM_152783:exon4:c.G376A:p.V126M	.	1.172	0.0003		3.30E-05	2.79E-05	Fam87_f_m_aM_uM	0/0;0/0;0/1;0/0
chr2	241804132	241804132	A	C	GAL3ST2	exonic	nonsynonymous SNV	GAL3ST2:NM_022134:exon4:c.A1163C:p.K388T	.	1.365	.		0.0003	3.49E-05	Fam44_f_m_aM_uF	0/0;0/1;0/1;0/0
chr2	241804132	241804132	A	C	GAL3ST2	exonic	nonsynonymous SNV	GAL3ST2:NM_022134:exon4:c.A1163C:p.K388T	.	1.365	.		0.0003	3.49E-05	Fam28_f_m_aF_uF	0/1;0/0;0/1;0/0

chr20	604598	604598	C	T	TCF15	exonic	nonsynonymous SNV	TCF15:NM_004609:exon2:c.G593A:p.R198Q	.	1.331	.	.	2.09E-05	Fam52_f_m_aM	0/1;0/0;0/1
chr20	663779	663779	C	G	SCRT2	exonic	nonsynonymous SNV	SCRT2:NM_033129:exon2:c.G816C:p.Q272H	.	1.231	0.0004	0.0005	0.0002	Fam18_f_m_aM_uF	0/1;0/0;0/1;0/1
chr20	2102068	2102068	T	C	STK35	exonic	nonsynonymous SNV	STK35:NM_080836:exon1:c.T187C:p.S63P	.	1.681	.	.	.	Fam113_f_m_aF_raM_uF	0/0;0/1;0/1;0/0;0/0
chr20	2988403	2988403	G	A	PTPRA	exonic	nonsynonymous SNV	PTPRA:NM_080840:exon8:c.G640A:p.V214M,PTPRA:NM_080841:exon8:c.G640A:p.V214M,PTPRA:NM_002836:exon13:c.G667A:p.V223M	.	1.518	0.0003	0.0002	9.77E-05	Fam55_f_m_aM_aM_dM	0/0;0/1;0/0;0/1;0/1
chr20	3164518	3164518	T	C	LZTS3	exonic	nonsynonymous SNV	LZTS3:NM_001365618:exon5:c.A1958G:p.H653R,LZTS3:NM_001367609:exon5:c.A2048G:p.H683R,LZTS3:NM_001282533:exon6:c.A1820G:p.H607R	.	1.524	.	.	6.98E-06	Fam95_f_m_aM_aM_uF	0/0;0/1;0/1;0/1;0/1
chr20	3230618	3230618	G	C	SLC4A11	exonic	nonsynonymous SNV	SLC4A11:NM_001363745:exon11:c.C1198G:p.L400V,SLC4A11:NM_032034:exon11:c.C1360G:p.L454V,SLC4A11:NM_001174089:exon12:c.C1312G:p.L438V,SLC4A11:NM_001174090:exon12:c.C1441G:p.L481V	.	1.001	.	.	.	Fam65_f_m_aM_uF_dF	0/1;0/0;0/1;0/0;0/1
chr20	3540289	3540289	G	A	ATRN	exonic	nonsynonymous SNV	ATRN:NM_001207047:exon3:c.G214A:p.V72I,ATRN:NM_001323332:exon3:c.G562A:p.V188I,ATRN:NM_139321:exon3:c.G562A:p.V188I,ATRN:NM_139322:exon3:c.G562A:p.V188I	.	1.187	.	.	.	Fam12_f_m_aM_uM_aM	0/1;0/0;0/1;0/1;0/1
chr20	3584059	3584059	T	C	ATRN	exonic	nonsynonymous SNV	ATRN:NM_001207047:exon17:c.T2578C:p.W860R,ATRN:NM_001323332:exon17:c.T2926C:p.W976R,ATRN:NM_139321:exon17:c.T2926C:p.W976R,ATRN:NM_139322:exon17:c.T2926C:p.W976R	.	1.962	.	.	.	Fam91_m_aM_dM_aM_dM	0/1;0/0;0/1;0/1;0/1
chr20	3661142	3661142	C	A	GFRA4	exonic	nonsynonymous SNV	GFRA4:NM_022139:exon2:c.G194T:p.R65L,GFRA4:NM_145762:exon2:c.G194T:p.R65L	.	1.054	0.0002	.	2.11E-05	Fam69_f_m_aM	0/0;0/1;0/1
chr20	3673480	3673480	G	C	ADAM33	exonic	nonsynonymous SNV	ADAM33:NM_001282447:exon11:c.C1007G:p.P336R,ADAM33:NM_025220:exon11:c.C1007G:p.P336R,ADAM33:NM_153202:exon11:c.C1007G:p.P336R	.	1.854	.	.	.	Fam31_f_m_aM_uF	0/0;0/1;0/1;0/1
chr20	3745068	3745068	A	G	HSPA12B	exonic	nonsynonymous SNV	HSPA12B:NM_001318322:exon4:c.A175G:p.M59V,HSPA12B:NM_001197327:exon5:c.A433G:p.M145V,HSPA12B:NM_052970:exon5:c.A433G:p.M145V	.	1.835	.	.	.	Fam48_f_m_aM_uM	0/0;0/1;0/1;0/0
chr20	3785046	3785046	C	A	CENPB	exonic	nonsynonymous SNV	CENPB:NM_001810:exon1:c.G1438T:p.V480L	.	1.437	7.36E-05	3.28E-05	6.98E-06	Fam64_f_m_aM	0/1;0/0;0/1
chr20	4790537	4790537	G	A	RASSF2	exonic	nonsynonymous SNV	RASSF2:NM_170774:exon6:c.C451T:p.R151C,RASSF2:NM_014737:exon7:c.C451T:p.R151C	.	1.353	.	0.0004	1.40E-05	Fam116_f_m_aM_aF	0/1;0/0;0/1;0/0
chr20	5184929	5184929	G	A	CDS2	exonic	nonsynonymous SNV	CDS2:NM_003818:exon8:c.G743A:p.R248Q	.	2.798	.	2.23E-05	.	Fam99_f_m_aM_aM	0/1;0/0;0/0;0/1
chr20	5558719	5558719	T	C	GPCPD1	exonic	nonsynonymous SNV	GPCPD1:NM_019593:exon18:c.A1633G:p.I545V	.	1.103	7.35E-05	0.0003	4.89E-05	Fam44_f_m_aM_uF	0/0;0/1;0/1;0/0
chr20	5580074	5580074	C	T	GPCPD1	exonic	nonsynonymous SNV	GPCPD1:NM_019593:exon7:c.G407A:p.R136H	.	1.463	.	.	.	Fam54_f_m_aM_uF	0/0;0/1;0/1;0/0
chr20	8740398	8740398	C	T	PLCB1	exonic	nonsynonymous SNV	PLCB1:NM_015192:exon22:c.C2363T:p.A788V,PLCB1:NM_182734:exon22:c.C2363T:p.A788V	.	1.132	.	.	.	Fam101_f_m_aM	0/1;0/0;0/1
chr20	10643817	10643817	C	T	JAG1	exonic	nonsynonymous SNV	JAG1:NM_000214:exon20:c.G2419A:p.E807K	.	1.392	.	3.28E-05	.	Fam24_f_m_aM_aM	0/1;0/0;0/1;0/1
chr20	11922786	11922786	C	G	BTBD3	exonic	nonsynonymous SNV	BTBD3:NM_001282552:exon4:c.C236G:p.S79C,BTBD3:NM_001282554:exon4:c.C236G:p.S79C,BTBD3:NM_014962:exon4:c.C689G:p.S230C,BTBD3:NM_001282550:exon5:c.C506G:p.S169C,BTBD3:NM_001282551:exon5:c.C506G:p.S169C,BTBD3:NM_181443:exon5:c.C818G:p.S273C	.	1.283	.	.	.	Fam75_f_m_aM	0/0;0/1;0/1
chr20	13630022	13630022	C	G	TASP1	exonic	nonsynonymous SNV	TASP1:NM_001323602:exon2:c.G57C:p.Q19H,TASP1:NM_017714:exon2:c.G57C:p.Q19H	.	1.387	.	5.59E-05	.	Fam85_f_m_aM_aM	0/0;0/1;0/1;0/0
chr20	16506085	16506085	G	T	KIF16B	exonic	nonsynonymous SNV	KIF16B:NM_001199865:exon8:c.C805A:p.L269I,KIF16B:NM_001199866:exon8:c.C805A:p.L269I,KIF16B:NM_024704:exon8:c.C805A:p.L269I	.	1.408	.	.	2.10E-05	Fam17_f_m_aM_uM	0/1;0/0;0/1;0/0
chr20	17735795	17735795	G	T	BANF2	exonic	nonsynonymous SNV	BANF2:NM_001014977:exon3:c.G257T:p.C86F,BANF2:NM_001159495:exon3:c.G278T:p.C93F,BANF2:NM_178477:exon4:c.G257T:p.C86F	.	1.017	.	.	.	Fam113_f_m_aF_raM_uF	0/0;0/0;0/0;0/1;0/0
chr20	19684188	19684188	G	A	SLC24A3	exonic	nonsynonymous SNV	SLC24A3:NM_020689:exon11:c.G914A:p.R305H	.	1.081	0.0003	0.0007	0.0002	Fam6_f_m_aM	0/0;0/1;0/1
chr20	20052656	20052656	C	-	CRNKL1	exonic	frameshift deletion	CRNKL1:NM_001278625:exon2:c.134delG:p.G45Dfs*83,CRNKL1:NM_016652:exon2:c.170delG:p.G57Dfs*83	0.445	.	.	.	.	Fam57_f_m_aM_uF	0/1;0/0;0/1;0/1
chr20	23036115	23036115	T	C	SSTR4	exonic	nonsynonymous SNV	SSTR4:NM_001052:exon1:c.T632C:p.F211S	.	1.735	7.35E-05	1.14E-05	6.98E-06	Fam65_f_m_aM_uF_dF	0/0;0/1;0/1;0/0;0/0
chr20	23036388	23036388	C	G	SSTR4	exonic	nonsynonymous SNV	SSTR4:NM_001052:exon1:c.C905G:p.A302G	.	1.111	.	.	6.98E-06	Fam110_f_m_aM_aM_uMs	0/0;0/1;0/0;0/1;0/0
chr20	23354275	23354275	G	C	NXT1	exonic	nonsynonymous SNV	NXT1:NM_013248:exon2:c.G234C:p.Q78H	.	1.802	.	1.12E-05	.	Fam50_f_m_aF	0/0;0/1;0/1
chr20	23637679	23637679	T	C	CST3	exonic	nonsynonymous SNV	CST3:NM_000099:exon1:c.A184G:p.K62E,CST3:NM_001288614:exon1:c.A184G:p.K62E	.	1.248	.	2.16E-05	6.98E-06	Fam48_f_m_aM_uM	0/1;0/0;0/1;0/0
chr20	25030885	25030885	C	A	ACSS1	exonic	nonsynonymous SNV	ACSS1:NM_001252676:exon2:c.G142T:p.A48S,ACSS1:NM_001252675:exon3:c.G505T:p.A169S,ACSS1:NM_001252677:exon3:c.G505T:p.A169S,ACSS1:NM_032501:exon3:c.G505T:p.A169S	.	1.298	.	.	6.98E-06	Fam6_f_m_aM	0/0;0/1;0/1
chr20	25030891	25030891	G	T	ACSS1	exonic	nonsynonymous SNV	ACSS1:NM_001252676:exon2:c.C136A:p.R46S,ACSS1:NM_001252675:exon3:c.C499A:p.R167S,ACSS1:NM_001252677:exon3:c.C499A:p.R167S,ACSS1:NM_032501:exon3:c.C499A:p.R167S	.	1.551	.	.	.	Fam115_f_m_aF_aM_aF	0/0;0/1;0/1;0/0;0/0

chr20	25218579	25218579	G	A	ENTPD6	exonic	nonsynonymous SNV	ENTPD6:NM_001322381:exon7:c.G254A:p.R85H,ENTPD6:NM_001322382:exon7:c.G254A:p.R85H,ENTPD6:NM_001322379:exon8:c.G254A:p.R85H,ENTPD6:NM_001322380:exon8:c.G254A:p.R85H,ENTPD6:NM_001322384:exon8:c.G254A:p.R85H,ENTPD6:NM_001322385:exon8:c.G713A:p.R238H,ENTPD6:NM_001114089:exon9:c.G857A:p.R286H,ENTPD6:NM_001322386:exon9:c.G857A:p.R286H,ENTPD6:NM_001322387:exon9:c.G764A:p.R255H,ENTPD6:NM_001322389:exon9:c.G899A:p.R300H,ENTPD6:NM_001322392:exon9:c.G854A:p.R285H,ENTPD6:NM_001322393:exon9:c.G857A:p.R286H,ENTPD6:NM_001322394:exon9:c.G854A:p.R285H,ENTPD6:NM_001322395:exon9:c.G899A:p.R300H,ENTPD6:NM_001322398:exon9:c.G899A:p.R300H,ENTPD6:NM_001247:exon10:c.G908A:p.R303H,ENTPD6:NM_001317941:exon10:c.G905A:p.R302H,ENTPD6:NM_001322378:exon10:c.G908A:p.R303H,ENTPD6:NM_001322388:exon10:c.G908A:p.R303H,ENTPD6:NM_001322391:exon10:c.G908A:p.R303H,ENTPD6:NM_001322396:exon10:c.G905A:p.R302H,ENTPD6:NM_001322397:exon10:c.G908A:p.R303H,ENTPD6:NM_001322383:exon11:c.G254A:p.R85H,ENTPD6:NM_001322390:exon11:c.G908A:p.R303H	.	1.005	0.0009	0.001	0.0007	Fam70_f_m_aM	0/0;0/1;0/1
chr20	32019096	32019096	G	T	CCM2L	exonic	nonsynonymous SNV	CCM2L:NM_001365692:exon5:c.G620T:p.W207L,CCM2L:NM_080625:exon5:c.G620T:p.W207L	.	1.221	.	.	0.0001	Fam32_f_m_aM_uM	0/1;0/0;0/1;0/1
chr20	32431426	32431426	C	A	ASXL1	exonic	nonsynonymous SNV	ASXL1:NM_001363734:exon7:c.C641A:p.A214D,ASXL1:NM_015338:exon8:c.C824A:p.A275D	.	2.057	.	.	.	Fam53_f_m_aM	0/1;0/0;0/1
chr20	33359046	33359046	G	C	CDK5RAP1	exonic	nonsynonymous SNV	CDK5RAP1:NM_001278168:exon13:c.C1530G:p.C510W,CDK5RAP1:NM_001278167:exon14:c.C1758G:p.C586W,CDK5RAP1:NM_001278169:exon14:c.C1680G:p.C560W,CDK5RAP1:NM_016082:exon14:c.C1764G:p.C588W,CDK5RAP1:NM_016408:exon14:c.C1761G:p.C587W,CDK5RAP1:NM_001365728:exon15:c.C1803G:p.C601W	.	1.043	7.34E-05	.	7.68E-05	Fam115_f_m_aF_aM_aF	0/1;0/0;0/0;0/1;0/0
chr20	33667511	33667511	C	T	ACTL10	exonic	nonsynonymous SNV	ACTL10:NM_001024675:exon1:c.C14T:p.A5V	.	1.078	.	.	.	Fam97_f_m_aM_aF	0/1;0/0;0/1;0/0
chr20	33676818	33676818	C	T	E2F1	exonic	nonsynonymous SNV	E2F1:NM_005225:exon7:c.G1228A:p.D410N	.	1.51	7.36E-05	3.55E-05	1.40E-05	Fam15_f_m_aM_aM	0/1;0/0;0/1;0/1
chr20	35619687	35619687	C	T	SPAG4	exonic	nonsynonymous SNV	SPAG4:NM_001317931:exon10:c.C787T:p.P263S,SPAG4:NM_003116:exon10:c.C1018T:p.P340S	.	1.355	.	6.73E-05	.	Fam11_f_m_aM_uM	0/1;0/0;0/1;0/1
chr20	35931414	35931414	A	G	PHF20	exonic	nonsynonymous SNV	PHF20:NM_016436:exon15:c.A2270G:p.H757R	.	1.311	7.34E-05	2.23E-05	6.98E-06	Fam38_f_m_aM	0/0;0/1;0/1
chr20	36432021	36432021	G	A	DLGAP4	exonic	nonsynonymous SNV	DLGAP4:NM_001365621:exon3:c.G304A:p.A102T,DLGAP4:NM_014902:exon3:c.G304A:p.A102T	.	1.389	0.0006	0.0003	0.0001	Fam66_f_m_aM	0/1;0/0;0/1
chr20	36432063	36432063	C	T	DLGAP4	exonic	nonsynonymous SNV	DLGAP4:NM_001365621:exon3:c.C346T:p.R116C,DLGAP4:NM_014902:exon3:c.C346T:p.R116C	.	1.732	.	0.0001	3.49E-05	Fam23_f_m_aM_dF_uFs	0/0;0/1;0/1;0/1;0/1
chr20	36500309	36500309	C	A	DLGAP4	exonic	nonsynonymous SNV	DLGAP4:NM_001042486:exon4:c.C89A:p.T30N,DLGAP4:NM_183006:exon4:c.C593A:p.T198N,DLGAP4:NM_001365621:exon10:c.C2210A:p.T737N,DLGAP4:NM_014902:exon10:c.C2201A:p.T734N	.	1.184	.	1.12E-05	.	Fam91_m_aM_dM_aM_dM	0/1;0/1;0/0;0/0;0/0
chr20	37254297	37254297	C	T	GHRH	exonic	nonsynonymous SNV	GHRH:NM_001184731:exon3:c.G221A:p.R74Q,GHRH:NM_021081:exon3:c.G221A:p.R74Q	.	1.132	.	0.0006	.	Fam103_f_m_aM	0/1;0/0;0/1
chr20	38509160	38509160	A	T	RALGAPB	exonic	nonsynonymous SNV	RALGAPB:NM_001282917:exon6:c.A824T:p.D275V,RALGAPB:NM_001282918:exon6:c.A824T:p.D275V,RALGAPB:NM_020336:exon6:c.A824T:p.D275V	.	1.551	.	1.12E-05	.	Fam21_f_m_aM_uM	0/0;0/1;0/1;0/0
chr20	38539810	38539810	G	A	RALGAPB	exonic	nonsynonymous SNV	RALGAPB:NM_001282917:exon17:c.G2414A:p.R805Q,RALGAPB:NM_001282918:exon17:c.G2402A:p.R801Q,RALGAPB:NM_020336:exon17:c.G2414A:p.R805Q	.	1.381	.	0.0008	1.40E-05	Fam97_f_m_aM_aF	0/1;0/0;0/1;0/0
chr20	38889597	38889597	C	T	PPP1R16B	exonic	nonsynonymous SNV	PPP1R16B:NM_001172735:exon3:c.C253T:p.R85C,PPP1R16B:NM_015568:exon3:c.C253T:p.R85C	.	1.581	.	4.47E-05	.	Fam26_f_m_aM_uM	0/1;0/0;0/1;0/1
chr20	41491815	41491815	C	T	CHD6	exonic	nonsynonymous SNV	CHD6:NM_032221:exon11:c.G1319A:p.R440Q	.	2.436	.	1.12E-05	.	Fam90_f_m_aF_dM	0/1;0/0;0/1;0/0
chr20	42677943	42677943	C	T	PTPRT	exonic	nonsynonymous SNV	PTPRT:NM_007050:exon7:c.G1076A:p.R359Q,PTPRT:NM_133170:exon7:c.G1076A:p.R359Q	.	1.203	.	3.28E-05	1.40E-05	Fam72_f_m_aF_uF	0/0;0/0;0/1;0/0
chr20	44279074	44279074	G	A	GDAP1L1	exonic	nonsynonymous SNV	GDAP1L1:NM_001256740:exon4:c.G665A:p.G222D,GDAP1L1:NM_001256739:exon5:c.G704A:p.G235D,GDAP1L1:NM_001256737:exon6:c.G935A:p.G312D,GDAP1L1:NM_001256738:exon6:c.G611A:p.G204D,GDAP1L1:NM_024034:exon6:c.G878A:p.G293D	.	1.57	.	3.28E-05	6.98E-06	Fam36_f_m_aM_uM	0/0;0/1;0/1;0/0
chr20	44306716	44306716	G	A	FITM2	exonic	nonsynonymous SNV	FITM2:NM_001080472:exon2:c.C698T:p.T233I	.	1.236	.	0.0001	0.0002	Fam75_f_m_aM	0/1;0/0;0/1
chr20	44311115	44311115	G	A	FITM2	exonic	nonsynonymous SNV	FITM2:NM_001080472:exon1:c.C34T:p.R12W	.	1.169	.	.	.	Fam87_f_m_aM_uM	0/1;0/0;0/1;0/0
chr20	44419771	44419771	C	T	HNF4A	exonic	nonsynonymous SNV	HNF4A:NM_000457:exon7:c.C787T:p.R263W,HNF4A:NM_001030003:exon7:c.C721T:p.R241W,HNF4A:NM_001030004:exon7:c.C721T:p.R241W,HNF4A:NM_175914:exon7:c.C721T:p.R241W,HNF4A:NM_178849:exon7:c.C787T:p.R263W,HNF4A:NM_178850:exon7:c.C787T:p.R263W,HNF4A:NM_001258355:exon8:c.C766T:p.R256W,HNF4A:NM_001287182:exon8:c.C712T:p.R238W,HNF4A:NM_001287183:exon8:c.C712T:p.R238W,HNF4A:NM_001287184:exon8:c.C712T:p.R238W	.	1.003	.	1.12E-05	1.40E-05	Fam99_f_m_aM_aM	0/1;0/0;0/0;0/1
chr20	44480246	44480246	C	T	TTPAL	exonic	nonsynonymous SNV	TTPAL:NM_001039199:exon2:c.C247T:p.R83C,TTPAL:NM_001261839:exon2:c.C247T:p.R83C,TTPAL:NM_024331:exon3:c.C247T:p.R83C	.	1.393	.	9.81E-05	1.40E-05	Fam22_f_m_aF_aF_uF	0/0;0/1;0/1;0/1;0/1
chr20	45301137	45301137	C	A	MATN4	exonic	nonsynonymous SNV	MATN4:NM_030590:exon4:c.G731T:p.G244V,MATN4:NM_003833:exon6:c.G854T:p.G285V	.	1.415	.	.	.	Fam80_f_m_aM_uM	0/0;0/1;0/1;0/0

chr20	45304434	45304434	G	T	MATN4	exonic	nonsynonymous SNV	MATN4:NM_030590:exon3:c.C437A:p.T146K,MATN4:NM_030592:exon3:c.C437A:p.T146K,MATN4:NM_003833:exon4:c.C437A:p.T146K	.	1.403	.	.	.	Fam95_f_m_aM_aM_uF	0/1;0/0;0/1;0/1;0/1
chr20	45947372	45947372	G	A	PCIF1	exonic	nonsynonymous SNV	PCIF1:NM_022104:exon16:c.G1817A:p.R606H	.	1.663	.	0.0002	.	Fam26_f_m_aM_uM	0/0;0/1;0/1;0/1
chr20	46041382	46041382	C	A	SLC12A5	exonic	nonsynonymous SNV	SLC12A5:NM_001134771:exon8:c.C977A:p.A326D,SLC12A5:NM_020708:exon8:c.C908A:p.A303D	.	2.044	.	.	.	Fam94_f_m_aM	0/1;0/0;0/1
chr20	46065076	46065076	T	C	NCOA5	exonic	nonsynonymous SNV	NCOA5:NM_001348148:exon5:c.A467G:p.Q156R,NCOA5:NM_001348151:exon5:c.A467G:p.Q156R,NCOA5:NM_001348149:exon6:c.A782G:p.Q261R,NCOA5:NM_020967:exon6:c.A782G:p.Q261R,NCOA5:NM_001348150:exon7:c.A593G:p.Q198R	.	1.17	.	.	.	Fam112_f_m_aM	0/1;0/0;0/1
chr20	47180820	47180820	A	G	EYA2	exonic	nonsynonymous SNV	EYA2:NM_005244:exon14:c.A1319G:p.N440S	.	1.709	.	.	.	Fam80_f_m_aM_uM	0/1;0/0;0/1;0/1
chr20	47294721	47294721	A	G	ZMYND8	exonic	nonsynonymous SNV	ZMYND8:NM_001281771:exon5:c.T437C:p.I146T,ZMYND8:NM_001281775:exon5:c.T512C:p.I171T,ZMYND8:NM_001281776:exon5:c.T512C:p.I171T,ZMYND8:NM_001281777:exon5:c.T437C:p.I146T,ZMYND8:NM_001281778:exon5:c.T437C:p.I146T,ZMYND8:NM_001281781:exon5:c.T437C:p.I146T,ZMYND8:NM_001281782:exon5:c.T437C:p.I146T,ZMYND8:NM_001281783:exon5:c.T512C:p.I171T,ZMYND8:NM_001281784:exon5:c.T437C:p.I146T,ZMYND8:NM_001363714:exon5:c.T533C:p.I178T,ZMYND8:NM_012408:exon5:c.T512C:p.I171T,ZMYND8:NM_183047:exon5:c.T512C:p.I171T,ZMYND8:NM_183048:exon5:c.T437C:p.I146T,ZMYND8:NM_001281773:exon6:c.T452C:p.I151T,ZMYND8:NM_001281774:exon6:c.T452C:p.I151T,ZMYND8:NM_001363741:exon6:c.T452C:p.I151T,ZMYND8:NM_001281772:exon7:c.T452C:p.I151T	.	1.403	0.0003	0.0001	4.19E-05	Fam106_f_m_aM	0/0;0/1;0/1
chr20	48636584	48636584	C	T	PREX1	exonic	nonsynonymous SNV	PREX1:NM_020820:exon32:c.G4046A:p.R1349H	.	1.204	0.0003	0.001	0.0003	Fam118_f_m_aM_aF_uM	0/0;0/1;0/1;0/1;0/1
chr20	48649548	48649548	C	A	PREX1	exonic	nonsynonymous SNV	PREX1:NM_020820:exon25:c.G3057T:p.Q1019H	.	1	.	.	.	Fam69_f_m_aM	0/0;0/1;0/1
chr20	49023106	49023106	A	G	ARFGEF2	exonic	nonsynonymous SNV	ARFGEF2:NM_006420:exon35:c.A4680G:p.I1560M	.	1.265	.	.	.	Fam45_f_m_aM_uF	0/0;0/1;0/1;0/0
chr20	49270740	49270740	T	A	ZNFX1	exonic	nonsynonymous SNV	ZNFX1:NM_021035:exon3:c.A1072T:p.I358F	.	1.09	.	.	.	Fam68_f_m_aF_uF_uM	0/1;0/0;0/1;0/0;0/0
chr20	50191112	50191112	G	A	CEBPB	exonic	nonsynonymous SNV	CEBPB:NM_001285878:exon1:c.G10A:p.A4T,CEBPB:NM_005194:exon1:c.G79A:p.A27T	.	1.937	.	4.02E-05	.	Fam94_f_m_aM	0/0;0/1;0/1
chr20	51671187	51671187	C	T	ATP9A	exonic	nonsynonymous SNV	ATP9A:NM_006045:exon12:c.G1108A:p.G370R	.	1.837	0.0002	0.0001	0.0003	Fam84_f_m_aF	0/1;0/0;0/1
chr20	51791947	51791947	G	C	SALL4	exonic	nonsynonymous SNV	SALL4:NM_001318031:exon2:c.C536G:p.T179S,SALL4:NM_020436:exon2:c.C536G:p.T179S	.	1.018	.	6.17E-05	.	Fam24_f_m_aM_aM	0/1;0/0;0/1;0/1
chr20	56631590	56631590	G	C	TFAP2C	exonic	nonsynonymous SNV	TFAP2C:NM_003222:exon2:c.G434C:p.R145P	.	2.077	.	.	.	Fam53_f_m_aM	0/1;0/0;0/1
chr20	56637775	56637775	C	G	TFAP2C	exonic	nonsynonymous SNV	TFAP2C:NM_003222:exon7:c.C1115G:p.P372R	.	2.1	.	.	.	Fam89_f_m_aM	0/0;0/1;0/1
chr20	57171059	57171059	G	A	BMP7	exonic	nonsynonymous SNV	BMP7:NM_001719:exon7:c.C1196T:p.T399M	.	1.743	.	.	.	Fam15_f_m_aM_aM	0/1;0/0;0/1;0/1
chr20	57173297	57173297	G	A	BMP7	exonic	nonsynonymous SNV	BMP7:NM_001719:exon6:c.C1049T:p.A350V	.	1.706	.	2.23E-05	6.98E-06	Fam74_f_m_aM_uM	0/1;0/0;0/1;0/1
chr20	57228290	57228290	G	C	BMP7	exonic	nonsynonymous SNV	BMP7:NM_001719:exon2:c.C550G:p.R184G	.	1.379	7.35E-05	0.0001	2.79E-05	Fam92_f_m_aM	0/1;0/0;0/1
chr20	57652300	57652300	C	T	PMEPA1	exonic	nonsynonymous SNV	PMEPA1:NM_001255976:exon4:c.G533A:p.C178Y,PMEPA1:NM_020182:exon4:c.G617A:p.C206Y,PMEPA1:NM_199169:exon4:c.G512A:p.C171Y,PMEPA1:NM_199170:exon4:c.G467A:p.C156Y,PMEPA1:NM_199171:exon4:c.G467A:p.C156Y	.	1.919	.	.	.	Fam94_f_m_aM	0/1;0/0;0/1
chr20	58461398	58461398	G	A	APCDD1L	exonic	nonsynonymous SNV	APCDD1L:NM_153360:exon4:c.C898T:p.R300W,APCDD1L:NM_001304787:exon5:c.C931T:p.R311W	.	1.048	.	3.86E-05	1.40E-05	Fam29_f_m_aF_uM	0/1;0/0;0/1;0/1
chr20	62012909	62012909	C	T	TAF4	exonic	nonsynonymous SNV	TAF4:NM_003185:exon3:c.G1547A:p.R516Q	.	1.459	.	1.12E-05	.	Fam94_f_m_aM	0/0;0/1;0/1
chr20	62014567	62014567	C	T	TAF4	exonic	nonsynonymous SNV	TAF4:NM_003185:exon2:c.G1501A:p.V501I	.	1.452	.	3.30E-05	6.99E-06	Fam119_f_m_aM_aM	0/0;0/1;0/1;0/1
chr20	62218601	62218601	C	T	HRH3	exonic	nonsynonymous SNV	HRH3:NM_007232:exon2:c.G307A:p.G103S	.	1.242	.	0.0006	0.0001	Fam27_f_m_aF_uM_uM	0/1;0/0;0/1;0/1;0/0
chr20	62279303	62279303	C	T	OSBPL2	exonic	nonsynonymous SNV	OSBPL2:NM_001278649:exon7:c.C362T:p.A121V,OSBPL2:NM_014835:exon7:c.C602T:p.A201V,OSBPL2:NM_144498:exon7:c.C638T:p.A213V,OSBPL2:NM_001363878:exon8:c.C362T:p.A121V	.	1.578	.	1.12E-05	.	Fam80_f_m_aM_uM	0/1;0/0;0/1;0/1
chr20	62289231	62289231	G	A	OSBPL2	exonic	nonsynonymous SNV	OSBPL2:NM_014835:exon12:c.G1114A:p.V372M,OSBPL2:NM_144498:exon12:c.G1150A:p.V384M,OSBPL2:NM_001363878:exon13:c.G874A:p.V292M	.	1.562	.	0.0002	.	Fam98_f_m_aM	0/0;0/1;0/1
chr20	62475060	62475060	C	G	GATA5	exonic	nonsynonymous SNV	GATA5:NM_080473:exon2:c.G462C:p.W154C	.	2.914	.	.	1.40E-05	Fam31_f_m_aM_uF	0/1;0/0;0/1;0/0
chr20	62760080	62760080	C	G	NTSR1	exonic	nonsynonymous SNV	NTSR1:NM_002531:exon4:c.C1070G:p.S357C	.	1.012	.	.	.	Fam46_f_m_aM_uM	0/1;0/0;0/1;0/1
chr20	62799453	62799454	CA	-	MRGBP	splicing	.	.	0.575	.	.	.	.	Fam53_f_m_aM	0/1;0/0;0/1
chr20	62881698	62881698	C	G	DIDO1	exonic	nonsynonymous SNV	DIDO1:NM_001193369:exon16:c.G4258C:p.E1420Q,DIDO1:NM_033081:exon16:c.G4258C:p.E1420Q	.	1.158	.	9.80E-05	6.98E-06	Fam90_f_m_aF_dM	0/0;0/1;0/1;0/1
chr20	62962697	62962697	G	A	SLC17A9	exonic	nonsynonymous SNV	SLC17A9:NM_022082:exon5:c.G571A:p.G191S,SLC17A9:NM_001302643:exon6:c.G553A:p.G185S	.	1.285	7.35E-05	3.28E-05	1.40E-05	Fam5_f_m_aM	0/1;0/0;0/1
chr20	63433803	63433803	-	G	KCNQ2	exonic	frameshift insertion	KCNQ2:NM_172109:exon8:c.1123dupC:p.R375Pfs*30	0.44	.	.	.	6.98E-06	Fam60_f_m_aF	0/1;0/0;0/1
chr20	63862115	63862115	C	T	ABHD16B	exonic	nonsynonymous SNV	ABHD16B:NM_080622:exon1:c.C575T:p.P192L	.	1.392	7.36E-05	8.03E-05	6.98E-05	Fam123_f_aF	0/1;0/1
chr20	63862133	63862133	C	G	ABHD16B	exonic	nonsynonymous SNV	ABHD16B:NM_080622:exon1:c.C593G:p.S198C	.	1.36	.	4.62E-05	6.98E-06	Fam11_f_m_aM_uM	0/1;0/0;0/1;0/1
chr20	64076559	64076559	A	G	RGS19	exonic	nonsynonymous SNV	RGS19:NM_001039467:exon3:c.T118C:p.C40R,RGS19:NM_005873:exon3:c.T118C:p.C40R	.	1.36	.	1.13E-05	.	Fam54_f_m_aM_uF	0/0;0/1;0/1;0/1
chr20	64083391	64083391	C	G	LKAAEAR1	exonic	nonsynonymous SNV	LKAAEAR1:NM_001007125:exon2:c.G717C:p.K239N	.	1.057	.	1.19E-05	.	Fam60_f_m_aF	0/1;0/0;0/1

chr20	64092938	64092938	T	G	OPRL1	exonic	nonsynonymous SNV	OPRL1:NM_001318854:exon1:c.T218G:p.M73R,OPRL1:NM_001318855:exon1:c.T103G:p.C35G,OPRL1:NM_000913:exon2:c.T218G:p.M73R,OPRL1:NM_182647:exon3:c.T218G:p.M73R,OPRL1:NM_001200019:exon4:c.T218G:p.M73R	.	1.345	.	.	.	Fam42_f_m_aM_uF	0/1;0/0;0/1;0/1
chr21	14964725	14964728	TTCT	-	NRIP1	exonic	frameshift deletion	NRIP1:NM_003489:exon4:c.3465_3468del:p.K1155Nfs*15	0.489	.	0.0006	0.0004	0.0001	Fam64_f_m_aM	0/0;0/1;0/1
chr21	25745266	25745266	A	G	GABPA	exonic	nonsynonymous SNV	GABPA:NM_001197297:exon3:c.A134G:p.N45S,GABPA:NM_002040:exon3:c.A134G:p.N45S	.	1.045	.	7.46E-05	4.19E-05	Fam106_f_m_aM	0/0;0/1;0/1
chr21	26837739	26837739	C	T	ADAMTS1	exonic	nonsynonymous SNV	ADAMTS1:NM_006988:exon9:c.G2744A:p.G915E	.	1.037	7.35E-05	3.28E-05	1.40E-05	Fam32_f_m_aM_uM	0/1;0/0;0/1;0/1
chr21	32741605	32741605	-	A	PAXBP1	exonic	frameshift insertion	PAXBP1:NM_013329:exon16:c.2351dupT:p.Q785Pfs*69	0.442	.	.	.	.	Fam85_f_m_aM_aM	0/0;0/1;0/1;0/1
chr21	33283199	33283199	G	A	IL10RB	exonic	nonsynonymous SNV	IL10RB:NM_000628:exon5:c.G604A:p.G202R	.	1.171	.	1.12E-05	.	Fam100_f_m_aF	0/0;0/1;0/1
chr21	34095505	34095505	C	T	SLC5A3	exonic	nonsynonymous SNV	SLC5A3:NM_006933:exon2:c.C307T:p.R103W	.	1.3	.	0.0002	6.29E-05	Fam106_f_m_aM	0/1;0/0;0/1
chr21	37227657	37227657	C	T	VPS26C	exonic	nonsynonymous SNV	VPS26C:NM_001331018:exon4:c.G433A:p.V145M,VPS26C:NM_001331021:exon6:c.G664A:p.V222M,VPS26C:NM_001331022:exon6:c.G727A:p.V243M,VPS26C:NM_006052:exon7:c.G808A:p.V270M	.	1.358	0.0007	0.0009	0.0005	Fam53_f_m_aM	0/1;0/0;0/1
chr21	38299719	38299719	C	T	KCNJ15	exonic	nonsynonymous SNV	KCNJ15:NM_001276438:exon3:c.C458T:p.T153M,KCNJ15:NM_170736:exon3:c.C458T:p.T153M,KCNJ15:NM_170737:exon3:c.C458T:p.T153M,KCNJ15:NM_001276437:exon4:c.C458T:p.T153M,KCNJ15:NM_001276439:exon4:c.C458T:p.T153M,KCNJ15:NM_002243:exon4:c.C458T:p.T153M,KCNJ15:NM_001276435:exon5:c.C458T:p.T153M,KCNJ15:NM_001276436:exon5:c.C458T:p.T153M	.	1.34	.	.	.	Fam115_f_m_aF_aM_aF	0/1;0/0;0/1;0/1;0/0
chr21	39280235	39280235	G	C	BRWD1	exonic	nonsynonymous SNV	BRWD1:NM_018963:exon9:c.C845G:p.A282G,BRWD1:NM_033656:exon9:c.C845G:p.A282G	.	1.454	.	1.15E-05	6.98E-06	Fam52_f_m_aM	0/1;0/0;0/1
chr21	40353548	40353548	C	T	DSCAM	exonic	nonsynonymous SNV	DSCAM:NM_001271534:exon5:c.G851A:p.R284H,DSCAM:NM_001389:exon5:c.G851A:p.R284H	.	1.602	0.0006	0.0004	5.58E-05	Fam23_f_m_aM_dF_uFs	0/1;0/0;0/1;0/1;0/0
chr21	40369136	40369136	C	A	DSCAM	exonic	nonsynonymous SNV	DSCAM:NM_001271534:exon4:c.G618T:p.E206D,DSCAM:NM_001389:exon4:c.G618T:p.E206D	.	1.399	.	.	.	Fam120_f_m_aM_aM	0/0;0/1;0/0;0/1
chr21	41257212	41257212	G	A	BACE2	exonic	nonsynonymous SNV	BACE2:NM_138991:exon7:c.G1039A:p.G347S,BACE2:NM_012105:exon8:c.G1189A:p.G397S	.	1.428	7.34E-05	0.0002	7.68E-05	Fam56_f_m_aF_aM	0/0;0/1;0/1;0/0
chr21	41756622	41756622	G	A	RIPK4	exonic	nonsynonymous SNV	RIPK4:NM_020639:exon2:c.C377T:p.A126V	.	1.254	.	6.54E-05	6.98E-06	Fam72_f_m_aF_uF	0/1;0/0;0/1;0/0
chr21	41756812	41756812	G	A	RIPK4	exonic	nonsynonymous SNV	RIPK4:NM_020639:exon2:c.C187T:p.R63C	.	1.496	.	0.0001	6.98E-06	Fam123_f_aF	0/1;0/1
chr21	42564785	42564785	G	A	SLC37A1	exonic	nonsynonymous SNV	SLC37A1:NM_001320537:exon14:c.G1213A:p.A405T,SLC37A1:NM_018964:exon15:c.G1213A:p.A405T	.	1.145	.	4.50E-05	6.98E-06	Fam106_f_m_aM	0/1;0/0;0/1
chr21	42564786	42564786	C	A	SLC37A1	exonic	nonsynonymous SNV	SLC37A1:NM_001320537:exon14:c.C1214A:p.A405D,SLC37A1:NM_018964:exon15:c.C1214A:p.A405D	.	1.464	.	4.50E-05	6.98E-06	Fam106_f_m_aM	0/1;0/0;0/1
chr21	43687996	43687996	C	T	RRP1B	exonic	nonsynonymous SNV	RRP1B:NM_015056:exon13:c.C1622T:p.A541V	.	1.099	.	.	.	Fam89_f_m_aM	0/1;0/0;0/1
chr21	43970678	43970678	G	A	AGPAT3	exonic	nonsynonymous SNV	AGPAT3:NM_001037553:exon5:c.G536A:p.R179H,AGPAT3:NM_001369878:exon5:c.G536A:p.R179H,AGPAT3:NM_001369880:exon5:c.G536A:p.R179H,AGPAT3:NM_001369881:exon6:c.G350A:p.R117H,AGPAT3:NM_020132:exon6:c.G536A:p.R179H	.	2.243	.	.	.	Fam17_f_m_aM_uM	0/0;0/0;0/1;0/0
chr21	43970798	43970798	G	A	AGPAT3	exonic	nonsynonymous SNV	AGPAT3:NM_001037553:exon5:c.G656A:p.R219Q,AGPAT3:NM_001369878:exon5:c.G656A:p.R219Q,AGPAT3:NM_001369880:exon5:c.G656A:p.R219Q,AGPAT3:NM_001369881:exon6:c.G470A:p.R157Q,AGPAT3:NM_020132:exon6:c.G656A:p.R219Q	.	1.105	7.36E-05	0.0001	4.89E-05	Fam116_f_m_aM_aF	0/1;0/0;0/1;0/0
chr21	44457129	44457129	T	C	LRRC3	exonic	nonsynonymous SNV	LRRC3:NM_030891:exon2:c.T485C:p.L162P	.	1.193	.	.	.	Fam63_f_m_aF	0/0;0/1;0/1
chr21	44771433	44771433	G	A	UBE2G2	exonic	nonsynonymous SNV	UBE2G2:NM_001202489:exon4:c.C232T:p.R78W,UBE2G2:NM_003343:exon6:c.C442T:p.R148W,UBE2G2:NM_182688:exon7:c.C358T:p.R120W	.	2.099	.	1.12E-05	.	Fam117_f_m_aM_aF	0/0;0/1;0/0;0/1
chr21	44890068	44890068	C	A	ITGB2	exonic	nonsynonymous SNV	ITGB2:NM_000211:exon12:c.G1567T:p.D523Y,ITGB2:NM_001127491:exon12:c.G1567T:p.D523Y,ITGB2:NM_001303238:exon12:c.G1360T:p.D454Y	.	1.172	.	.	.	Fam59_f_m_aF_uM	0/0;0/1;0/1;0/0
chr21	45531506	45531506	A	C	SLC19A1	exonic	nonsynonymous SNV	SLC19A1:NM_001205207:exon2:c.T712G:p.S238A,SLC19A1:NM_001205206:exon3:c.T832G:p.S278A,SLC19A1:NM_001352510:exon3:c.T478G:p.S160A,SLC19A1:NM_001352511:exon3:c.T832G:p.S278A,SLC19A1:NM_001352512:exon3:c.T832G:p.S278A,SLC19A1:NM_194255:exon3:c.T832G:p.S278A	.	1.517	.	.	.	Fam38_f_m_aM	0/0;0/1;0/1
chr21	46554919	46554919	C	T	DIP2A	exonic	nonsynonymous SNV	DIP2A:NM_001146116:exon28:c.C3362T:p.T1121I,DIP2A:NM_001353942:exon28:c.C3377T:p.T1126I,DIP2A:NM_001353943:exon28:c.C3374T:p.T1125I,DIP2A:NM_015151:exon28:c.C3374T:p.T1125I	.	1.738	0.0001	9.95E-05	7.68E-05	Fam68_f_m_aF_uF_uM	0/0;0/1;0/1;0/1;0/1
chr21	46566608	46566608	A	G	DIP2A	exonic	nonsynonymous SNV	DIP2A:NM_001146116:exon37:c.A4376G:p.E1459G,DIP2A:NM_001353942:exon37:c.A4391G:p.E1464G,DIP2A:NM_001353943:exon37:c.A4388G:p.E1463G,DIP2A:NM_015151:exon37:c.A4388G:p.E1463G	.	1.803	.	.	.	Fam7_f_m_aM_aM_uM	0/1;0/0;0/0;0/1;0/1
chr22	17818426	17818426	A	-	MICAL3	exonic	frameshift deletion	MICAL3:NM_015241:exon26:c.4235delT:p.L1412Hfs*26	0.336	.	.	.	.	Fam122_f_m_aM	0/1;0/0;0/1
chr22	19447539	19447539	G	A	C22orf39	exonic	nonsynonymous SNV	C22orf39:NM_001166242:exon2:c.C142T:p.R48C,C22orf39:NM_173793:exon2:c.C142T:p.R48C	.	1.213	.	0.0001	6.98E-06	Fam78_f_m_aF_uM	0/1;0/0;0/1;0/1
chr22	20139810	20139810	C	T	ZDHHC8	exonic	nonsynonymous SNV	ZDHHC8:NM_001185024:exon4:c.C475T:p.H159Y,ZDHHC8:NM_013373:exon4:c.C475T:p.H159Y	.	2.287	.	.	.	Fam37_f_m_aF_uM	0/1;0/0;0/1;0/0
chr22	20464868	20464868	G	C	KLHL22	exonic	nonsynonymous SNV	KLHL22:NM_032775:exon4:c.C1102G:p.R368G	.	1.29	.	5.65E-05	.	Fam56_f_m_aF_aM	0/1;0/0;0/0;0/1

chr22	20973823	20973823	C	G	AIFM3	exonic	nonsynonymous SNV	AIFM3:NM_001018060:exon4:c.C311G:p.A104G,AIFM3:NM_001146288:exon4:c.C329G:p.A110G,AIFM3:NM_144704:exon4:c.C311G:p.A104G	.	1.119	7.35E-05	2.92E-05	5.59E-05	Fam21_f_m_aM_uM	0/0;0/1;0/1;0/0
chr22	20974736	20974736	C	T	AIFM3	exonic	nonsynonymous SNV	AIFM3:NM_001018060:exon8:c.C640T:p.R214W,AIFM3:NM_001146288:exon8:c.C658T:p.R220W,AIFM3:NM_144704:exon8:c.C640T:p.R214W	.	1.136	7.36E-05	0.0001	9.77E-05	Fam54_f_m_aM_uF	0/1;0/0;0/1;0/0
chr22	20974800	20974800	G	A	AIFM3	exonic	nonsynonymous SNV	AIFM3:NM_001018060:exon8:c.G704A:p.R235H,AIFM3:NM_001146288:exon8:c.G722A:p.R241H,AIFM3:NM_144704:exon8:c.G704A:p.R235H	.	1.245	.	0.0003	6.98E-06	Fam119_f_m_aM_aM	0/1;0/0;0/1;0/1
chr22	20979650	20979650	G	T	AIFM3	exonic	nonsynonymous SNV	AIFM3:NM_001018060:exon18:c.G1600T:p.V534F,AIFM3:NM_001146288:exon18:c.G1618T:p.V540F,AIFM3:NM_144704:exon18:c.G1600T:p.V534F	.	1.285	.	.	.	Fam76_f_m_aM_uM	0/1;0/0;0/1;0/1
chr22	21446079	21446079	G	T	HIC2	exonic	nonsynonymous SNV	HIC2:NM_015094:exon3:c.G1184T:p.C395F	.	1.683	.	.	.	Fam84_f_m_aF	0/0;0/1;0/1
chr22	21923264	21923264	C	T	PPM1F	exonic	nonsynonymous SNV	PPM1F:NM_014634:exon8:c.G1193A:p.R398Q	.	1.003	0.0004	0.0006	0.0002	Fam29_f_m_aF_uM	0/0;0/1;0/1;0/0
chr22	21957233	21957233	G	A	TOP3B	exonic	nonsynonymous SNV	TOP3B:NM_001282112:exon18:c.C2470T:p.R824C,TOP3B:NM_001282113:exon18:c.C2470T:p.R824C,TOP3B:NM_003935:exon18:c.C2470T:p.R824C,TOP3B:NM_001349845:exon19:c.C2470T:p.R824C,TOP3B:NM_001349847:exon19:c.C2470T:p.R824C,TOP3B:NM_001349848:exon19:c.C2062T:p.R688C,TOP3B:NM_001349850:exon20:c.C2062T:p.R688C	.	3.328	.	0.0001	2.10E-05	Fam91_m_aM_dM_aM_dM	0/1;0/1;0/1;0/0;0/1
chr22	21959709	21959709	C	A	TOP3B	exonic	nonsynonymous SNV	TOP3B:NM_001282112:exon15:c.G1682T:p.R561L,TOP3B:NM_001282113:exon15:c.G1682T:p.R561L,TOP3B:NM_003935:exon15:c.G1682T:p.R561L,TOP3B:NM_001349845:exon16:c.G1682T:p.R561L,TOP3B:NM_001349847:exon16:c.G1682T:p.R561L,TOP3B:NM_001349848:exon16:c.G1274T:p.R425L,TOP3B:NM_001349851:exon16:c.G1274T:p.R425L,TOP3B:NM_001349850:exon17:c.G1274T:p.R425L,TOP3B:NM_001349852:exon17:c.G1274T:p.R425L	.	1.517	.	.	.	Fam112_f_m_aM	0/0;0/1;0/1
chr22	23158001	23158001	C	T	RAB36	exonic	nonsynonymous SNV	RAB36:NM_001349878:exon7:c.C602T:p.T201M,RAB36:NM_004914:exon7:c.C602T:p.T201M,RAB36:NM_001349877:exon8:c.C674T:p.T225M	.	1.261	7.34E-05	.	6.98E-06	Fam113_f_m_aF_raM_uF	0/1;0/0;0/1;0/0;0/1
chr22	23767502	23767502	T	C	CHCHD10	exonic	nonsynonymous SNV	CHCHD10:NM_001301339:exon2:c.A133G:p.M45V,CHCHD10:NM_213720:exon2:c.A133G:p.M45V	.	1.377	.	.	.	Fam102_f_m_aM	0/1;0/0;0/1
chr22	24050974	24050974	C	T	CABIN1	exonic	nonsynonymous SNV	CABIN1:NM_001199281:exon8:c.C806T:p.T269I,CABIN1:NM_012295:exon8:c.C806T:p.T269I	.	1.004	0.0003	2.24E-05	6.98E-06	Fam92_f_m_aM_raM	0/0;0/1;0/1;0/0
chr22	24072451	24072451	A	G	CABIN1	exonic	nonsynonymous SNV	CABIN1:NM_001201429:exon17:c.A2423G:p.E808G,CABIN1:NM_001199281:exon18:c.A2573G:p.E858G,CABIN1:NM_012295:exon18:c.A2573G:p.E858G	.	1.044	7.35E-05	8.93E-05	7.68E-05	Fam70_f_m_aM	0/0;0/1;0/1
chr22	24113569	24113569	G	A	CABIN1	exonic	nonsynonymous SNV	CABIN1:NM_001201429:exon26:c.G3971A:p.R1324Q,CABIN1:NM_001199281:exon27:c.G4121A:p.R1374Q,CABIN1:NM 012295:exon27:c.G4121A:p.R1374Q	.	1.032	.	0.0001	6.28E-05	Fam116_f_m_aM_aF	0/1;0/0;0/1;0/1
chr22	26443105	26443105	G	C	ASPHD2	exonic	nonsynonymous SNV	ASPHD2:NM_020437:exon4:c.G1009C:p.E337Q	.	1.453	7.34E-05	0.0004	0.0001	Fam109_f_m_aM	0/0;0/1;0/1
chr22	29049289	29049289	G	A	ZNRF3	exonic	nonsynonymous SNV	ZNRF3:NM_001206998:exon8:c.G1108A:p.G370S,ZNRF3:NM_032173:exon8:c.G808A:p.G270S	.	1.189	.	3.29E-05	.	Fam17_f_m_aM_uM	0/0;0/1;0/1;0/1
chr22	29480753	29480753	G	A	NEFH	exonic	nonsynonymous SNV	NEFH:NM_021076:exon1:c.G491A:p.R164H	.	1.325	.	.	.	Fam47_f_m_aM	0/1;0/0;0/1
chr22	29481098	29481098	A	G	NEFH	exonic	nonsynonymous SNV	NEFH:NM_021076:exon1:c.A836G:p.E279G	.	1.261	.	.	.	Fam110_f_m_aM_aM_uMs	0/1;0/0;0/1;0/1;0/0
chr22	29528466	29528466	T	C	THOC5	exonic	nonsynonymous SNV	THOC5:NM_001002879:exon10:c.A926G:p.D309G,THOC5:NM_003678:exon10:c.A926G:p.D309G,THOC5:NM_001002877:exon11:c.A926G:p.D309G,THOC5:NM_001002878:exon11:c.A926G:p.D309G	.	1.331	.	.	.	Fam37_f_m_aF_uM	0/1;0/0;0/1;0/0
chr22	29531899	29531899	G	A	THOC5	exonic	nonsynonymous SNV	THOC5:NM_001002879:exon8:c.C779T:p.T260I,THOC5:NM_003678:exon8:c.C779T:p.T260I,THOC5:NM_001002877:exon9:c.C779T:p.T260I,THOC5:NM_001002878:exon9:c.C779T:p.T260I	.	1.208	.	2.23E-05	.	Fam27_f_m_aF_uM_uM	0/1;0/0;0/1;0/0;0/0
chr22	30009123	30009123	C	T	MTMR3	exonic	nonsynonymous SNV	MTMR3:NM_021090:exon12:c.C1115T:p.P372L,MTMR3:NM_153050:exon12:c.C1115T:p.P372L,MTMR3:NM_153051:exon12:c.C1115T:p.P372L	.	2.04	.	9.84E-05	2.10E-05	Fam7_f_m_aM_aM_uM	0/1;0/0;0/0;0/1;0/1
chr22	30287253	30287253	G	A	CASTOR1	exonic	nonsynonymous SNV	CASTOR1:NM_001037666:exon4:c.C407T:p.T136M	.	1.436	7.35E-05	8.08E-05	7.68E-05	Fam91_m_aM_dM_aM_dM	0/0;0/0;0/0;0/1;0/1
chr22	30294970	30294970	C	T	TBC1D10A	exonic	nonsynonymous SNV	TBC1D10A:NM_001204240:exon5:c.G631A:p.A211T,TBC1D10A:NM_031937:exon5:c.G610A:p.A204T	.	1.789	.	7.46E-05	.	Fam37_f_m_aF_uM	0/1;0/0;0/1;0/0
chr22	30345147	30345147	G	A	SF3A1	exonic	nonsynonymous SNV	SF3A1:NM_005877:exon4:c.C437T:p.P146L	.	1.963	.	.	.	Fam29_f_m_aF_uM	0/0;0/1;0/1;0/0
chr22	30380398	30380398	G	A	RNF215	exonic	nonsynonymous SNV	RNF215:NM_001017981:exon6:c.C748T:p.P250S	.	1.13	.	.	.	Fam91_m_aM_dM_aM_dM	0/0;0/1;0/1;0/0;0/0
chr22	30905926	30905926	T	G	OSBP2	exonic	nonsynonymous SNV	OSBP2:NM_001282742:exon11:c.T1097G:p.L366R,OSBP2:NM_001282740:exon12:c.T1364G:p.L455R,OSBP2:NM_001282741:exon12:c.T1691G:p.L564R,OSBP2:NM_001282739:exon13:c.T2462G:p.L821R,OSBP2:NM_030758:exon13:c.T2465G:p.L822R,OSBP2:NM_001282738:exon14:c.T1967G:p.L656R	.	1.818	.	.	.	Fam113_f_m_aF_raM_uF	0/1;0/0;0/0;0/1;0/1
chr22	30946442	30946442	T	C	MORC2	exonic	nonsynonymous SNV	MORC2:NM_001303256:exon6:c.A325G:p.M109V,MORC2:NM_001303257:exon6:c.A325G:p.M109V,MORC2:NM_014941:exon7:c.A139G:p.M47V	.	1.891	.	0.0002	3.49E-05	Fam75_f_m_aM	0/0;0/1;0/1
chr22	31463937	31463937	A	G	EIF4ENIF1	exonic	nonsynonymous SNV	EIF4ENIF1:NM_001164501:exon5:c.T329C:p.L110S,EIF4ENIF1:NM_019843:exon5:c.T329C:p.L110S	.	2.082	0.0005	0.0006	0.0002	Fam123_f_aF	0/0;0/1

chr22	31906313	31906313	G	A	DEPDC5	exonic	nonsynonymous SNV	DEPDC5:NM_001242897:exon40:c.G4328A:p.R1443Q,DEPDC5:NM_001363854:exon41:c.G4394A:p.R1465Q,DEPDC5:NM_001364319:exon41:c.G4394A:p.R1465Q,DEPDC5:NM_001363852:exon42:c.G4562A:p.R1521Q,DEPDC5:NM_001364320:exon42:c.G4562A:p.R1521Q,DEPDC5:NM_001369901:exon42:c.G4544A:p.R1515Q,DEPDC5:NM_001369902:exon42:c.G4544A:p.R1515Q,DEPDC5:NM_001369903:exon42:c.G4535A:p.R1512Q,DEPDC5:NM_014662:exon42:c.G4535A:p.R1512Q,DEPDC5:NM_001136029:exon43:c.G4601A:p.R1534Q,DEPDC5:NM_001242896:exon43:c.G4628A:p.R1543Q,DEPDC5:NM_001364318:exon43:c.G4628A:p.R1543Q	.	1.3	.	0.0002	1.40E-05	Fam117_f_m_aM_aF	0/1;0/0;0/1;0/0
chr22	35284013	35284013	G	A	HMGXB4	exonic	nonsynonymous SNV	HMGXB4:NM_001362972:exon5:c.G940A:p.V314M,HMGXB4:NM_001003681:exon6:c.G1267A:p.V423M	.	1.801	.	0.0004	1.40E-05	Fam106_f_m_aM	0/0;0/1;0/1
chr22	36284229	36284229	G	A	MYH9	exonic	nonsynonymous SNV	MYH9:NM_002473:exon40:c.C5629T:p.R1877W	.	1.408	.	7.52E-05	1.40E-05	Fam91_m_aM_dM_aM_dM	0/1;0/0;0/0;0/1;0/0
chr22	36300183	36300183	T	G	MYH9	exonic	nonsynonymous SNV	MYH9:NM_002473:exon23:c.A2920C:p.K974Q	.	1.48	.	0.0003	6.98E-06	Fam97_f_m_aM_aF	0/1;0/0;0/1;0/0
chr22	37373685	37373685	T	A	ELFN2	exonic	nonsynonymous SNV	ELFN2:NM_052906:exon3:c.A1850T:p.Q617L	.	1.306	.	.	.	Fam28_f_m_aF_uF	0/0;0/1;0/1;0/1
chr22	37374621	37374621	G	A	ELFN2	exonic	nonsynonymous SNV	ELFN2:NM_052906:exon3:c.C914T:p.T305M	.	1.535	7.35E-05	0.0005	0.0002	Fam60_f_m_aF	0/1;0/0;0/1
chr22	37824913	37824913	C	G	GALR3	exonic	nonsynonymous SNV	GALR3:NM_003614:exon2:c.C550G:p.L184V	.	1.803	.	2.99E-05	.	Fam16_f_m_aM_aM	0/0;0/1;0/1;0/0
chr22	37983675	37983675	C	T	SOX10	exonic	nonsynonymous SNV	SOX10:NM_006941:exon2:c.G110A:p.G37D	.	1.08	.	.	.	Fam110_f_m_aM_aM_uMs	0/0;0/1;0/1;0/1;0/0
chr22	38073058	38073058	T	C	PICK1	exonic	nonsynonymous SNV	PICK1:NM_001039583:exon10:c.T749C:p.I250T,PICK1:NM_001039584:exon10:c.T749C:p.I250T,PICK1:NM_012407:exon10:c.T749C:p.I250T	.	2.067	.	.	.	Fam2_f_m_aF	0/1;0/0;0/1
chr22	38081107	38081107	C	T	SLC16A8	exonic	nonsynonymous SNV	SLC16A8:NM_013356:exon4:c.G931A:p.G311S	.	2.368	.	.	.	Fam57_f_m_aM_uF	0/1;0/0;0/1;0/1
chr22	39233488	39233488	C	T	PDGFB	exonic	nonsynonymous SNV	PDGFB:NM_002608:exon3:c.G197A:p.R66H,PDGFB:NM_033016:exon3:c.G152A:p.R51H	.	1.136	.	0.0002	1.40E-05	Fam55_f_m_aM_aM_dM	0/1;0/0;0/1;0/1;0/0
chr22	39315518	39315518	A	G	RPL3	exonic	nonsynonymous SNV	RPL3:NM_000967:exon5:c.T539C:p.L180P,RPL3:NM_001033853:exon5:c.T392C:p.L131P	.	2.125	.	0.0001	3.49E-05	Fam56_f_m_aF_aM	0/1;0/0;0/1;0/0
chr22	39488750	39488750	C	T	MGAT3	exonic	nonsynonymous SNV	MGAT3:NM_001098270:exon1:c.C1403T:p.T468M,MGAT3:NM_002409:exon2:c.C1403T:p.T468M	.	2.164	.	.	1.40E-05	Fam5_f_m_aM	0/0;0/0;0/1
chr22	39664859	39664859	G	A	CACNA1I	exonic	nonsynonymous SNV	CACNA1I:NM_001003406:exon20:c.G3682A:p.G1228R,CACNA1I:NM_021096:exon21:c.G3787A:p.G1263R	.	2.605	.	6.52E-05	1.40E-05	Fam91_m_aM_dM_aM_dM	0/0;0/1;0/1;0/0;0/0
chr22	39672234	39672234	G	A	CACNA1I	exonic	nonsynonymous SNV	CACNA1I:NM_001003406:exon26:c.G4470A:p.M1490I,CACNA1I:NM_021096:exon27:c.G4575A:p.M1525I	.	1.341	.	2.25E-05	6.98E-06	Fam4_f_m_aM	0/1;0/0;0/1
chr22	39679369	39679369	G	A	CACNA1I	exonic	nonsynonymous SNV	CACNA1I:NM_001003406:exon31:c.G5213A:p.G1738D,CACNA1I:NM_021096:exon32:c.G5318A:p.G1773D	.	2.626	.	.	1.40E-05	Fam14_f_m_aM_aM	0/0;0/1;0/1;0/0
chr22	41578862	41578862	T	A	PMM1	exonic	nonsynonymous SNV	PMM1:NM_002676:exon6:c.A494T:p.K165M	.	1.288	.	1.12E-05	.	Fam28_f_m_aF_uF	0/0;0/1;0/1;0/1
chr22	41996918	41996918	G	T	SEPTIN3	exonic	nonsynonymous SNV	SEPTIN3:NM_145733:exon11:c.G1028T:p.G343V,SEPTIN3:NM_001363845:exon12:c.G2522T:p.G841V	.	2.106	.	.	.	Fam24_f_m_aM_aM	0/1;0/0;0/0;0/1
chr22	42693201	42693201	G	A	A4GALT	exonic	nonsynonymous SNV	A4GALT:NM_001318038:exon3:c.C751T:p.P251S,A4GALT:NM_017436:exon3:c.C751T:p.P251S	.	1.092	.	.	.	Fam99_f_m_aM_aM	0/1;0/0;0/0;0/1
chr22	42693390	42693390	C	T	A4GALT	exonic	nonsynonymous SNV	A4GALT:NM_001318038:exon3:c.G562A:p.G188S,A4GALT:NM_017436:exon3:c.G562A:p.G188S	.	1.083	.	6.19E-05	6.98E-06	Fam90_f_m_aF_dM	0/0;0/0;0/1;0/0
chr22	42882291	42882291	A	T	PACSIN2	exonic	nonsynonymous SNV	PACSIN2:NM_001349974:exon6:c.T676A:p.Y226N,PACSIN2:NM_001184970:exon7:c.T799A:p.Y267N,PACSIN2:NM_001184971:exon7:c.T799A:p.Y267N,PACSIN2:NM_001349968:exon7:c.T799A:p.Y267N,PACSIN2:NM_001349969:exon7:c.T799A:p.Y267N,PACSIN2:NM_001349970:exon7:c.T799A:p.Y267N,PACSIN2:NM_001349971:exon7:c.T799A:p.Y267N,PACSIN2:NM_001349972:exon7:c.T799A:p.Y267N,PACSIN2:NM_001349973:exon7:c.T799A:p.Y267N,PACSIN2:NM_007229:exon7:c.T799A:p.Y267N	.	1.415	0.0003	0.0007	6.98E-05	Fam12_f_m_aM_uM_aM	0/1;0/0;0/0;0/0;0/1
chr22	43212434	43212434	C	G	SCUBE1	exonic	nonsynonymous SNV	SCUBE1:NM_173050:exon17:c.G2212C:p.E738Q	.	1.472	.	4.81E-05	6.98E-06	Fam53_f_m_aM	0/0;0/1;0/1
chr22	49885413	49885413	G	A	ZBED4	exonic	nonsynonymous SNV	ZBED4:NM_014838:exon2:c.G1751A:p.R584Q	.	1.281	7.34E-05	0.0006	0.0004	Fam56_f_m_aF_aM	0/0;0/1;0/0;0/1
chr22	50198442	50198442	C	T	TRABD	exonic	nonsynonymous SNV	TRABD:NM_001320484:exon10:c.C1066T:p.R356C,TRABD:NM_001320485:exon10:c.C1054T:p.R352C,TRABD:NM_001320487:exon10:c.C1054T:p.R352C,TRABD:NM_001320488:exon10:c.C1054T:p.R352C,TRABD:NM_025204:exon10:c.C1054T:p.R352C	.	1.175	.	0.0002	2.79E-05	Fam121_f_m_aF_aM	0/1;0/0;0/0;0/1
chr22	50406822	50406822	G	A	PPP6R2	exonic	nonsynonymous SNV	PPP6R2:NM_001242898:exon4:c.G361A:p.A121T,PPP6R2:NM_001242899:exon4:c.G361A:p.A121T,PPP6R2:NM_001242900:exon4:c.G361A:p.A121T,PPP6R2:NM_001351641:exon4:c.G361A:p.A121T,PPP6R2:NM_001351642:exon4:c.G361A:p.A121T,PPP6R2:NM_001351644:exon4:c.G361A:p.A121T,PPP6R2:NM_001351646:exon4:c.G361A:p.A121T,PPP6R2:NM_014678:exon4:c.G361A:p.A121T,PPP6R2:NM_001351643:exon5:c.G361A:p.A121T,PPP6R2:NM_001351645:exon5:c.G361A:p.A121T,PPP6R2:NM_001365836:exon6:c.G361A:p.A121T	.	1.213	0.0006	0.0007	0.0004	Fam71_f_m_aF	0/0;0/1;0/1
chr22	50461158	50461158	C	T	SBF1	splicing	.	.	0.37	.	.	.	.	Fam11_f_m_aM_uM	0/0;0/1;0/1;0/1
chr22	50720258	50720258	G	T	SHANK3	exonic	stopgain	SHANK3:NM_033517:exon21:c.G2383T:p.E795X	0.446	.	.	.	.	Fam123_f_aF	0/0;0/1
chr22	50730801	50730801	C	A	SHANK3	exonic	stopgain	SHANK3:NM_033517:exon22:c.C4643A:p.S1548X	0.446	.	.	.	.	Fam29_f_m_aF_uM	0/0;0/0;0/1;0/0
chr3	4673278	4673278	T	G	ITPR1	exonic	nonsynonymous SNV	ITPR1:NM_001168272:exon20:c.T2302G:p.C768G,ITPR1:NM_002222:exon20:c.T2302G:p.C768G,ITPR1:NM_001099952:exon21:c.T2347G:p.C783G	.	1.981	.	.	.	Fam36_f_m_aM_uM	0/1;0/0;0/1;0/0
chr3	7146528	7146528	T	G	GRM7	exonic	nonsynonymous SNV	GRM7:NM_000844:exon2:c.T596G:p.V199G,GRM7:NM_181874:exon2:c.T596G:p.V199G	.	1.692	.	.	.	Fam103_f_m_aM	0/0;0/1;0/1

chr3	7452645	7452645	G	A	GRM7	exonic	nonsynonymous SNV	GRM7:NM_000844:exon6:c.G1213A:p.E405K,GRM7:NM_181874:exon6:c.G1213A:p.E405K	.	1.128	.	.	.	Fam85_f_m_aM_aM	0/1;0/0;0/1;0/1
chr3	7578654	7578654	A	T	GRM7	exonic	nonsynonymous SNV	GRM7:NM_000844:exon8:c.A1748T:p.K583I,GRM7:NM_181874:exon8:c.A1748T:p.K583I	.	1.977	.	.	.	Fam7_f_m_aM_aM_uM	0/0;0/1;0/0;0/1;0/1
chr3	8537327	8537327	C	T	LMCD1	exonic	nonsynonymous SNV	LMCD1:NM_001278233:exon2:c.C55T:p.R19W,LMCD1:NM_001278235:exon3:c.C274T:p.R92W,LMCD1:NM_014583:exon3:c.C274T:p.R92W	.	1.043	.	0.0002	4.89E-05	Fam4_f_m_aM	0/1;0/0;0/1
chr3	8994389	8994389	C	T	SRGAP3	exonic	nonsynonymous SNV	SRGAP3:NM_001033117:exon19:c.G2290A:p.G764S,SRGAP3:NM_014850:exon19:c.G2362A:p.G788S	.	1.4	7.34E-05	3.27E-05	2.79E-05	Fam46_f_m_aM_uM	0/1;0/0;0/1;0/0
chr3	9734400	9734400	G	A	BRPF1	exonic	nonsynonymous SNV	BRPF1:NM_001003694:exon2:c.G260A:p.R87H,BRPF1:NM_001319049:exon2:c.G260A:p.R87H,BRPF1:NM_001319050:exon2:c.G260A:p.R87H,BRPF1:NM_004634:exon2:c.G260A:p.R87H	.	1.039	.	7.46E-05	6.98E-06	Fam43_f_m_aM	0/0;0/1;0/1
chr3	9784137	9784137	G	A	TADA3	exonic	nonsynonymous SNV	TADA3:NM_001278270:exon8:c.C997T:p.R333C,TADA3:NM_006354:exon8:c.C997T:p.R333C,TADA3:NM_133480:exon8:c.C997T:p.R333C	.	2.017	.	.	6.98E-06	Fam70_f_m_aM	0/0;0/1;0/1
chr3	9812944	9812944	A	G	ARPC4-TTLL3;TTLL	exonic	nonsynonymous SNV	TTLL3:NM_001025930:exon3:c.A479G:p.Q160R,TTLL3:NM_001366051:exon3:c.A50G:p.Q17R,ARPC4-TTLL3:NM_001198793:exon5:c.A332G:p.Q111R	.	1.278	.	.	.	Fam56_f_m_aF_aM	0/0;0/1;0/0;0/1
chr3	10345392	10345392	T	C	ATP2B2	exonic	nonsynonymous SNV	ATP2B2:NM_001330611:exon15:c.A2560G:p.I854V,ATP2B2:NM_001363862:exon15:c.A2560G:p.I854V,ATP2B2:NM_001683:exon15:c.A2560G:p.I854V,ATP2B2:NM_001353564:exon16:c.A2560G:p.I854V,ATP2B2:NM_001001331:exon18:c.A2695G:p.I899V	.	2.308	.	4.48E-05	.	Fam61_f_m_aM	0/0;0/1;0/1
chr3	10371864	10371864	A	G	ATP2B2	exonic	nonsynonymous SNV	ATP2B2:NM_001330611:exon9:c.T1469C:p.M490T,ATP2B2:NM_001363862:exon9:c.T1469C:p.M490T,ATP2B2:NM_001683:exon9:c.T1469C:p.M490T,ATP2B2:NM_001353564:exon10:c.T1469C:p.M490T,ATP2B2:NM_001001331:exon12:c.T1604C:p.M535T	.	1.004	.	.	.	Fam121_f_m_aF_aM	0/0;0/1;0/1;0/1
chr3	10385329	10385329	T	C	ATP2B2	splicing	.	.	0.231	.	.	2.24E-05	.	Fam38_f_m_aM	0/1;0/0;0/1
chr3	10388394	10388394	C	A	ATP2B2	exonic	nonsynonymous SNV	ATP2B2:NM_001001331:exon6:c.G790T:p.V264L,ATP2B2:NM_001330611:exon6:c.G790T:p.V264L,ATP2B2:NM_001363862:exon6:c.G790T:p.V264L,ATP2B2:NM_001683:exon6:c.G790T:p.V264L,ATP2B2:NM_001353564:exon7:c.G790T:p.V264L	.	1.92	.	.	.	Fam20_f_m_aM_uF	0/0;0/1;0/1;0/0
chr3	10402207	10402207	C	T	ATP2B2	exonic	nonsynonymous SNV	ATP2B2:NM_001001331:exon4:c.G539A:p.R180Q,ATP2B2:NM_001330611:exon4:c.G539A:p.R180Q,ATP2B2:NM_001363862:exon4:c.G539A:p.R180Q,ATP2B2:NM_001683:exon4:c.G539A:p.R180Q,ATP2B2:NM_001353564:exon5:c.G539A:p.R180Q	.	2.077	.	.	.	Fam83_f_m_aF	0/1;0/0;0/1
chr3	10816367	10816367	-	CCCG	SLC6A11	exonic	frameshift insertion	SLC6A11:NM_001317406:exon1:c.102_103insCCCGCGCGCCA:p.V41Afs*25,SLC6A11:NM_014229:exon1:c.102_103insCCCGCGCGCCA:p.V41Afs*25	0.534	.	.	.	.	Fam7_f_m_aM_aM_uM	0/1;0/0;0/1;0/1;0/0
chr3	11033648	11033648	G	A	SLC6A1	exonic	nonsynonymous SNV	SLC6A1:NM_001348252:exon13:c.G902A:p.R301Q,SLC6A1:NM_001348253:exon13:c.G902A:p.R301Q,SLC6A1:NM_001348250:exon14:c.G1436A:p.R479Q,SLC6A1:NM_001348251:exon14:c.G1076A:p.R359Q,SLC6A1:NM_003042:exon14:c.G1436A:p.R479Q	.	2.271	.	0.0003	5.58E-05	Fam59_f_m_aF_uM	0/1;0/0;0/1;0/1
chr3	12803583	12803583	G	T	CAND2	exonic	nonsynonymous SNV	CAND2:NM_001162499:exon2:c.G164T:p.R55L,CAND2:NM_012298:exon2:c.G164T:p.R55L	.	1.49	.	.	.	Fam110_f_m_aM_aM_uMs	0/1;0/0;0/1;0/1;0/1
chr3	12936481	12936481	C	T	IQSEC1	exonic	nonsynonymous SNV	IQSEC1:NM_001134382:exon3:c.G535A:p.E179K,IQSEC1:NM_001330619:exon3:c.G211A:p.E71K,IQSEC1:NM_014869:exon3:c.G577A:p.E193K	.	1.359	7.36E-05	7.46E-05	6.98E-06	Fam5_f_m_aM	0/1;0/0;0/1
chr3	13504244	13504244	G	A	HDAC11	exonic	nonsynonymous SNV	HDAC11:NM_001330636:exon6:c.G563A:p.R188H,HDAC11:NM_001136041:exon9:c.G647A:p.R216H,HDAC11:NM_024827:exon9:c.G800A:p.R267H	.	1.228	.	0.0004	4.88E-05	Fam81_f_m_aM_uM	0/1;0/0;0/1;0/1
chr3	13875012	13875012	C	T	WNT7A	exonic	nonsynonymous SNV	WNT7A:NM_004625:exon2:c.G233A:p.R78H	.	1.844	.	.	.	Fam5_f_m_aM	0/1;0/0;0/1
chr3	20040629	20040629	C	G	KAT2B	exonic	nonsynonymous SNV	KAT2B:NM_003884:exon1:c.C152G:p.A51G	.	1.802	.	0.0001	.	Fam40_f_m_aM_aM	0/1;0/0;0/1;0/1
chr3	20099904	20099904	G	A	KAT2B	exonic	nonsynonymous SNV	KAT2B:NM_003884:exon4:c.G619A:p.E207K	.	1.401	.	.	.	Fam10_f_m_aM_uF	0/1;0/0;0/1;0/1
chr3	26709701	26709701	G	A	LRRC3B	exonic	nonsynonymous SNV	LRRC3B:NM_001317808:exon2:c.G29A:p.R10H,LRRC3B:NM_001317809:exon2:c.G29A:p.R10H,LRRC3B:NM_052953:exon2:c.G29A:p.R10H,LRRC3B:NM_001317810:exon3:c.G29A:p.R10H,LRRC3B:NM_001317811:exon3:c.G29A:p.R10H	.	1.26	.	0.0001	.	Fam22_f_m_aF_aF_uF	0/0;0/1;0/1;0/1;0/0
chr3	27404964	27404964	C	G	SLC4A7	splicing	.	.	0.508	.	.	.	.	Fam50_f_m_aF	0/1;0/0;0/1
chr3	32140277	32140277	G	A	GPD1L	exonic	nonsynonymous SNV	GPD1L:NM_015141:exon4:c.G416A:p.R139H	.	1.361	0.0006	7.45E-05	2.09E-05	Fam91_m_aM_dM_aM_dM	0/1;0/1;0/0;0/0;0/1
chr3	32818529	32818529	G	C	TRIM71	exonic	nonsynonymous SNV	TRIM71:NM_001039111:exon1:c.G449C:p.R150P	.	1.342	7.72E-05	.	1.42E-05	Fam76_f_m_aM_uM	0/0;0/1;0/1;0/0
chr3	33072605	33072605	G	A	GLB1	exonic	nonsynonymous SNV	GLB1:NM_000404:exon2:c.C184T:p.R62C,GLB1:NM_001079811:exon2:c.C94T:p.R32C,GLB1:NM_001135602:exon2:c.C184T:p.R62C,GLB1:NM_001317040:exon3:c.C328T:p.R110C	.	1.008	.	5.61E-05	3.49E-05	Fam11_f_m_aM_uM	0/1;0/0;0/1;0/1
chr3	33364647	33364647	C	T	FBXL2	exonic	nonsynonymous SNV	FBXL2:NM_001349316:exon5:c.C218T:p.S73L,FBXL2:NM_012157:exon5:c.C218T:p.S73L	.	1.807	.	1.12E-05	.	Fam25_f_m_aM	0/1;0/0;0/1
chr3	33408740	33408740	-	G	UBP1	exonic	frameshift insertion	UBP1:NM_014517:exon8:c.876dupC:p.K293Qfs*10,UBP1:NM_001128161:exon9:c.876dupC:p.K293Qfs*10	0.492	.	.	.	.	Fam122_f_m_aM	0/0;0/1;0/1

chr3	33581907	33581907	C	T	CLASP2	exonic	nonsynonymous SNV	CLASP2:NM_001207044:exon17:c.G1562A:p.R521Q,CLASP2:NM_001365630:exon21:c.G2219A:p.R740Q,CLASP2:NM_001365633:exon21:c.G2222A:p.R741Q,CLASP2:NM_001365634:exon21:c.G2219A:p.R740Q,CLASP2:NM_001365627:exon23:c.G2261A:p.R754Q,CLASP2:NM_001365628:exon23:c.G2264A:p.R755Q,CLASP2:NM_001365629:exon23:c.G2261A:p.R754Q,CLASP2:NM_001365631:exon23:c.G2261A:p.R754Q,CLASP2:NM_001365632:exon23:c.G2261A:p.R754Q,CLASP2:NM_015097:exon23:c.G2264A:p.R755Q	.	1.411	.	.	.	Fam35_f_m_aF_uM	0/1;0/0;0/1;0/0
chr3	37997078	37997078	T	A	VILL	exonic	nonsynonymous SNV	VILL:NM_015873:exon5:c.T452A:p.V151E	.	1.007	.	.	.	Fam91_m_aM_dM_aM_dM	0/1;0/0;0/1;0/1;0/1
chr3	38550574	38550574	C	T	SCN5A	exonic	nonsynonymous SNV	SCN5A:NM_001099405:exon27:c.G5744A:p.G1915D,SCN5A:NM_001160161:exon27:c.G5636A:p.G1879D,SCN5A:NM_001354701:exon27:c.G5741A:p.G1914D,SCN5A:NM_000335:exon28:c.G5795A:p.G1932D,SCN5A:NM_001099404:exon28:c.G5798A:p.G1933D,SCN5A:NM_001160160:exon28:c.G5699A:p.G1900D,SCN5A:NM_198056:exon28:c.G5798A:p.G1933D	.	1.311	.	3.29E-05	.	Fam71_f_m_aF	0/0;0/1;0/1
chr3	38550682	38550682	C	T	SCN5A	exonic	nonsynonymous SNV	SCN5A:NM_001099405:exon27:c.G5636A:p.R1879Q,SCN5A:NM_001160161:exon27:c.G5528A:p.R1843Q,SCN5A:NM_001354701:exon27:c.G5633A:p.R1878Q,SCN5A:NM_000335:exon28:c.G5687A:p.R1896Q,SCN5A:NM_001099404:exon28:c.G5690A:p.R1897Q,SCN5A:NM_001160160:exon28:c.G5591A:p.R1864Q,SCN5A:NM_198056:exon28:c.G5690A:p.R1897Q	.	1.544	0.0006	9.81E-05	1.40E-05	Fam54_f_m_aM_uF	0/0;0/1;0/1;0/1
chr3	38551441	38551441	C	T	SCN5A	exonic	nonsynonymous SNV	SCN5A:NM_001099405:exon27:c.G4877A:p.R1626H,SCN5A:NM_001160161:exon27:c.G4769A:p.R1590H,SCN5A:NM_001354701:exon27:c.G4874A:p.R1625H,SCN5A:NM_000335:exon28:c.G4928A:p.R1643H,SCN5A:NM_001099404:exon28:c.G4931A:p.R1644H,SCN5A:NM_001160160:exon28:c.G4832A:p.R1611H,SCN5A:NM_198056:exon28:c.G4931A:p.R1644H	.	1.606	.	.	1.40E-05	Fam117_f_m_aM_aF	0/1;0/0;0/0;0/1
chr3	38630314	38630314	T	C	SCN5A	exonic	nonsynonymous SNV	SCN5A:NM_000335:exon3:c.A389G:p.H130R,SCN5A:NM_001099404:exon3:c.A389G:p.H130R,SCN5A:NM_001099405:exon3:c.A389G:p.H130R,SCN5A:NM_001160160:exon3:c.A389G:p.H130R,SCN5A:NM_001160161:exon3:c.A389G:p.H130R,SCN5A:NM_001354701:exon3:c.A389G:p.H130R,SCN5A:NM_198056:exon3:c.A389G:p.H130R	.	1.225	.	.	.	Fam65_f_m_aM_uF_dF	0/0;0/1;0/1;0/0;0/1
chr3	41233842	41233842	C	T	CTNNB1	exonic	nonsynonymous SNV	CTNNB1:NM_001098209:exon9:c.C1499T:p.P500L,CTNNB1:NM_001098210:exon9:c.C1499T:p.P500L,CTNNB1:NM_001904:exon9:c.C1499T:p.P500L,CTNNB1:NM_001330729:exon10:c.C1478T:p.P493L	.	1.282	.	.	.	Fam38_f_m_aM	0/0;0/1;0/1
chr3	43348132	43348132	C	T	SNRK	exonic	nonsynonymous SNV	SNRK:NM_001330750:exon5:c.C1255T:p.R419C,SNRK:NM_001100594:exon6:c.C1873T:p.R625C,SNRK:NM_017719:exon7:c.C1873T:p.R625C	.	1.069	0.0001	0.0004	0.0001	Fam70_f_m_aM	0/0;0/1;0/1
chr3	45225205	45225205	C	A	TMEM158	exonic	nonsynonymous SNV	TMEM158:NM_015444:exon1:c.G823T:p.A275S	.	1.429	7.51E-05	.	2.82E-05	Fam21_f_m_aM_uM	0/1;0/0;0/1;0/0
chr3	45225870	45225870	G	A	TMEM158	exonic	nonsynonymous SNV	TMEM158:NM_015444:exon1:c.C158T:p.S53L	.	2.098	.	.	.	Fam94_f_m_aM	0/0;0/1;0/1
chr3	45719590	45719590	A	G	SACM1L	exonic	nonsynonymous SNV	SACM1L:NM_001319072:exon7:c.A485G:p.Y162C,SACM1L:NM_014016:exon8:c.A668G:p.Y223C,SACM1L:NM_001319073:exon9:c.A359G:p.Y120C	.	1.391	.	.	.	Fam117_f_m_aM_aF	0/0;0/1;0/0;0/1
chr3	46203847	46203847	A	C	CCR1	exonic	nonsynonymous SNV	CCR1:NM_001295:exon2:c.T467G:p.I156S	.	1.105	7.34E-05	.	6.98E-06	Fam107_f_m_aM	0/1;0/0;0/1
chr3	46204089	46204089	G	C	CCR1	exonic	nonsynonymous SNV	CCR1:NM_001295:exon2:c.C225G:p.N75K	.	1.494	.	.	.	Fam49_f_m_aM	0/0;0/1;0/1
chr3	47007643	47007643	C	T	NBEAL2	exonic	nonsynonymous SNV	NBEAL2:NM_001365116:exon47:c.C7351T:p.R2451W,NBEAL2:NM_015175:exon48:c.C7453T:p.R2485W	.	1.398	7.35E-05	0.0001	5.58E-05	Fam26_f_m_aM_uM	0/1;0/0;0/1;0/1
chr3	47322683	47322683	G	A	KLHL18	exonic	nonsynonymous SNV	KLHL18:NM_025010:exon3:c.G376A:p.A126T	.	1.483	7.34E-05	1.15E-05	6.98E-06	Fam40_f_m_aM_aM	0/0;0/1;0/0;0/1
chr3	47729048	47729048	G	C	SMARCC1	exonic	nonsynonymous SNV	SMARCC1:NM_003074:exon6:c.C623G:p.P208R	.	1.104	.	1.12E-05	.	Fam98_f_m_aM	0/1;0/0;0/1
chr3	47827467	47827467	C	G	DHX30	exonic	nonsynonymous SNV	DHX30:NM_138615:exon5:c.C245G:p.P82R,DHX30:NM_001330990:exon6:c.C161G:p.P54R,DHX30:NM_014966:exon6:c.C128G:p.P43R	.	1.951	.	.	.	Fam82_f_m_aM_uF	0/0;0/1;0/1;0/1
chr3	47840940	47840940	G	C	DHX30	exonic	nonsynonymous SNV	DHX30:NM_138615:exon7:c.G430C:p.D144H,DHX30:NM_001330990:exon8:c.G346C:p.D116H,DHX30:NM_014966:exon8:c.G313C:p.D105H	.	1.376	.	.	.	Fam66_f_m_aM	0/1;0/0;0/1
chr3	48406876	48406876	T	G	PLXNB1	exonic	nonsynonymous SNV	PLXNB1:NM_001130082:exon36:c.A6175C:p.T2059P,PLXNB1:NM_002673:exon36:c.A6175C:p.T2059P	.	1.27	.	.	.	Fam116_f_m_aM_aF	0/1;0/0;0/1;0/1
chr3	48409677	48409677	G	A	PLXNB1	exonic	nonsynonymous SNV	PLXNB1:NM_001130082:exon33:c.C5833T:p.R1945C,PLXNB1:NM_002673:exon33:c.C5833T:p.R1945C	.	1.626	7.35E-05	2.24E-05	2.79E-05	Fam6_f_m_aM	0/1;0/0;0/1
chr3	48414876	48414876	G	C	PLXNB1	exonic	nonsynonymous SNV	PLXNB1:NM_001130082:exon21:c.C4132G:p.P1378A,PLXNB1:NM_002673:exon21:c.C4132G:p.P1378A	.	1.465	.	4.48E-05	6.98E-06	Fam50_f_m_aF	0/0;0/1;0/1
chr3	48465005	48465005	G	T	ATRIP	exonic	nonsynonymous SNV	ATRIP:NM_032166:exon11:c.G2149T:p.V717F,ATRIP:NM_001271023:exon12:c.G1951T:p.V651F,ATRIP:NM_130384:exon12:c.G2230T:p.V744F,ATRIP:NM_001271022:exon13:c.G1849T:p.V617F	.	1.084	.	6.22E-05	.	Fam29_f_m_aF_uM	0/1;0/0;0/1;0/0
chr3	48536386	48536386	C	A	PFKFB4	exonic	nonsynonymous SNV	PFKFB4:NM_001317135:exon8:c.G710T:p.R237L,PFKFB4:NM_001317136:exon8:c.G677T:p.R226L,PFKFB4:NM_001317137:exon8:c.G710T:p.R237L,PFKFB4:NM_004567:exon8:c.G710T:p.R237L,PFKFB4:NM_001317134:exon9:c.G797T:p.R266L,PFKFB4:NM_001317138:exon9:c.G137T:p.R46L	.	1.523	.	3.28E-05	6.98E-06	Fam19_f_m_aM	0/0;0/1;0/1
chr3	48659960	48659960	C	T	CELSR3	exonic	nonsynonymous SNV	CELSR3:NM_001407:exon1:c.G2675A:p.R892H	.	2.078	.	0.0001	3.49E-05	Fam118_f_m_aM_aF_uM	0/0;0/1;0/1;0/1;0/0

chr3	48661503	48661503	T	C	CELSR3	exonic	nonsynonymous SNV	CELSR3:NM_001407:exon1:c.A1132G:p.I378V	.	1.329	7.36E-05	7.08E-05	7.68E-05	Fam91_m_aM_dM_aM_dM	0/1;0/0;0/0;0/1;0/1
chr3	48973752	48973752	A	G	ARIH2	exonic	nonsynonymous SNV	ARIH2:NM_001317334:exon7:c.A398G:p.K133R,ARIH2:NM_001349211:exon8:c.A824G:p.K275R,ARIH2:NM_001349212:exon8:c.A398G:p.K133R,ARIH2:NM_001349209:exon9:c.A824G:p.K275R,ARIH2:NM_001349214:exon9:c.A824G:p.K275R,ARIH2:NM_001349215:exon9:c.A824G:p.K275R,ARIH2:NM_001349218:exon9:c.A608G:p.K203R,ARIH2:NM_001349221:exon9:c.A608G:p.K203R,ARIH2:NM_001349224:exon9:c.A572G:p.K191R,ARIH2:NM_001349226:exon9:c.A572G:p.K191R,ARIH2:NM_006321:exon9:c.A824G:p.K275R,ARIH2:NM_001317333:exon10:c.A824G:p.K275R,ARIH2:NM_001349213:exon10:c.A824G:p.K275R,ARIH2:NM_001349216:exon10:c.A608G:p.K203R,ARIH2:NM_001349217:exon10:c.A608G:p.K203R,ARIH2:NM_001349219:exon10:c.A608G:p.K203R,ARIH2:NM_001349227:exon10:c.A275G:p.K92R,ARIH2:NM_001349210:exon11:c.A824G:p.K275R,ARIH2:NM_001349220:exon11:c.A608G:p.K203R,ARIH2:NM_001349222:exon11:c.A572G:p.K191R,ARIH2:NM_001349223:exon11:c.A572G:p.K191R,ARIH2:NM_001349225:exon12:c.A572G:p.K191R	.	1.205	.	1.12E-05	.	Fam7_f_m_aM_aM_uM	0/0;0/1;0/0;0/1;0/0
chr3	49026836	49026836	G	A	IMPDH2	exonic	nonsynonymous SNV	IMPDH2:NM_000884:exon7:c.C670T:p.R224W	.	1.153	.	6.17E-05	1.40E-05	Fam71_f_m_aF	0/0;0/1;0/1
chr3	49098008	49098008	T	C	QARS	exonic	nonsynonymous SNV	QARS:NM_001272073:exon23:c.A2228G:p.D743G,QARS:NM_005051:exon23:c.A2261G:p.D754G	.	1.2	0.0005	0.0004	0.0003	Fam119_f_m_aM_aM	0/0;0/1;0/1;0/1
chr3	49131675	49131675	G	A	LAMB2	exonic	nonsynonymous SNV	LAMB2:NM_002292:exon5:c.C508T:p.R170C	.	1.552	.	7.48E-05	6.98E-06	Fam13_f_m_aM	0/1;0/0;0/1
chr3	49172830	49172830	C	T	KLHDC8B	exonic	nonsynonymous SNV	KLHDC8B:NM_173546:exon2:c.C61T:p.R21W	.	1.337	.	.	.	Fam119_f_m_aM_aM	0/1;0/0;0/1;0/1
chr3	49532285	49532285	G	A	DAG1	exonic	nonsynonymous SNV	DAG1:NM_001177639:exon3:c.G1774A:p.E592K,DAG1:NM_001177643:exon3:c.G1774A:p.E592K,DAG1:NM_001177644:exon3:c.G1774A:p.E592K,DAG1:NM_004393:exon3:c.G1774A:p.E592K,DAG1:NM_001177635:exon4:c.G1774A:p.E592K,DAG1:NM_001177637:exon4:c.G1774A:p.E592K,DAG1:NM_001177638:exon4:c.G1774A:p.E592K,DAG1:NM_001177640:exon4:c.G1774A:p.E592K,DAG1:NM_001177641:exon4:c.G1774A:p.E592K,DAG1:NM_001177642:exon4:c.G1774A:p.E592K,DAG1:NM_001177636:exon5:c.G1774A:p.E592K,DAG1:NM_001165928:exon6:c.G1774A:p.E592K,DAG1:NM_001177634:exon6:c.G1774A:p.E592K	.	1.013	.	2.24E-05	6.98E-06	Fam14_f_m_aM_aM	0/0;0/1;0/1;0/1
chr3	50093726	50093726	C	T	RBM5	exonic	nonsynonymous SNV	RBM5:NM_005778:exon4:c.C190T:p.R64C	.	1.302	.	3.27E-05	.	Fam75_f_m_aM	0/0;0/1;0/1
chr3	50183239	50183239	G	A	SEMA3F	exonic	nonsynonymous SNV	SEMA3F:NM_001318798:exon10:c.G775A:p.V259I,SEMA3F:NM_001318800:exon10:c.G979A:p.V327I,SEMA3F:NM_004186:exon11:c.G1072A:p.V358I	.	1.517	.	.	1.40E-05	Fam7_f_m_aM_aM_uM	0/0;0/1;0/1;0/0;0/1
chr3	50503216	50503216	A	C	CACNA2D2	splicing	.	.	0.41	.	.	.	.	Fam72_f_m_aF_uF	0/1;0/0;0/1;0/0
chr3	50607989	50607989	T	C	CISH	exonic	nonsynonymous SNV	CISH:NM_145071:exon3:c.A395G:p.E132G,CISH:NM_013324:exon4:c.A446G:p.E149G	.	1.079	.	.	.	Fam51_f_m_aM_uF	0/1;0/0;0/1;0/1
chr3	51717745	51717745	G	A	GRM2	exonic	nonsynonymous SNV	GRM2:NM_001349116:exon4:c.G1339A:p.V447M,GRM2:NM_000839:exon5:c.G2473A:p.V825M,GRM2:NM_001349117:exon6:c.G1282A:p.V428M	.	1.409	.	2.24E-05	.	Fam7_f_m_aM_aM_uM	0/1;0/0;0/1;0/1;0/1
chr3	51935602	51935602	C	T	RRP9	exonic	nonsynonymous SNV	RRP9:NM_004704:exon9:c.G826A:p.V276M	.	1.29	7.34E-05	0.0001	7.68E-05	Fam121_f_m_aF_aM	0/0;0/1;0/0;0/1
chr3	52147085	52147085	C	T	POC1A	exonic	nonsynonymous SNV	POC1A:NM_001161580:exon5:c.G466A:p.D156N,POC1A:NM_001161581:exon5:c.G352A:p.D118N,POC1A:NM_015426:exon5:c.G466A:p.D156N	.	1.009	.	0.0002	6.98E-05	Fam20_f_m_aM_uF	0/1;0/0;0/1;0/0
chr3	52897437	52897437	G	A	STIMATE;STIMATE	exonic	nonsynonymous SNV	STIMATE-MUSTN1:NM_001198974:exon1:c.C14T:p.A5V,STIMATE:NM_198563:exon1:c.C14T:p.A5V	.	1.667	.	.	4.19E-05	Fam7_f_m_aM_aM_uM	0/1;0/0;0/1;0/1;0/1
chr3	52912569	52912569	A	G	SFMBT1	exonic	nonsynonymous SNV	SFMBT1:NM_016329:exon16:c.T1699C:p.W567R	.	1.457	.	3.28E-05	6.98E-06	Fam121_f_m_aF_aM	0/1;0/0;0/0;0/1
chr3	53231388	53231388	G	C	TKT	exonic	nonsynonymous SNV	TKT:NM_001064:exon7:c.C911G:p.P304R,TKT:NM_001135055:exon7:c.C911G:p.P304R,TKT:NM_001258028:exon8:c.C935G:p.P312R	.	1.144	.	.	.	Fam85_f_m_aM_aM	0/1;0/0;0/1;0/1
chr3	53775936	53775936	G	A	CACNA1D	exonic	nonsynonymous SNV	CACNA1D:NM_001128839:exon34:c.G4208A:p.G1403E,CACNA1D:NM_001128840:exon35:c.G4253A:p.G1418E,CACNA1D:NM_000720:exon36:c.G4313A:p.G1438E	.	2.143	.	.	.	Fam54_f_m_aM_uF	0/1;0/0;0/1;0/0
chr3	56646923	56646923	T	C	TASOR	exonic	nonsynonymous SNV	TASOR:NM_015224:exon7:c.A626G:p.Y209C,TASOR:NM_001112736:exon14:c.A1814G:p.Y605C,TASOR:NM_001363940:exon14:c.A1814G:p.Y605C,TASOR:NM_001365635:exon14:c.A1814G:p.Y605C,TASOR:NM_001365636:exon14:c.A1814G:p.Y605C,TASOR:NM_001365637:exon14:c.A1814G:p.Y605C,TASOR:NM_001365638:exon14:c.A1814G:p.Y605C	.	1.044	.	6.66E-05	.	Fam42_f_m_aM_uF	0/1;0/0;0/1;0/1
chr3	57663646	57663646	T	C	DENND6A	exonic	nonsynonymous SNV	DENND6A:NM_152678:exon5:c.A503G:p.Y168C	.	1.399	.	3.63E-05	.	Fam5_f_m_aM	0/0;0/1;0/1
chr3	58136145	58136145	A	T	FLNB	exonic	nonsynonymous SNV	FLNB:NM_001164318:exon28:c.A4838T:p.D1613V,FLNB:NM_001164319:exon28:c.A4838T:p.D1613V,FLNB:NM_001457:exon28:c.A4838T:p.D1613V,FLNB:NM_001164317:exon29:c.A4931T:p.D1644V	.	1.088	.	.	.	Fam99_f_m_aM_aM	0/0;0/1;0/0;0/1

chr3	58424869	58424869	G	T	PXK	exonic	nonsynonymous SNV	PXK:NM_001349532:exon14:c.G1172T:p.R391L,PXK:NM_001289101:exon15:c.G1235T:p.R412L,PXK:NM_001349526:exon15:c.G1232T:p.R411L,PXK:NM_001289096:exon16:c.G1547T:p.R516L,PXK:NM_001349500:exon16:c.G1544T:p.R515L,PXK:NM_001349501:exon16:c.G1535T:p.R512L,PXK:NM_001349502:exon16:c.G1532T:p.R511L,PXK:NM_001349491:exon17:c.G1235T:p.R412L,PXK:NM_001349494:exon17:c.G1598T:p.R533L,PXK:NM_001349495:exon17:c.G1595T:p.R532L,PXK:NM_001349496:exon17:c.G1595T:p.R532L,PXK:NM_001349497:exon17:c.G1592T:p.R531L,PXK:NM_001349499:exon17:c.G1583T:p.R528L,PXK:NM_001289099:exon18:c.G1397T:p.R466L,PXK:NM_001349498:exon18:c.G1586T:p.R529L,PXK:NM_017771:exon18:c.G1646T:p.R549L,PXK:NM_001349489:exon19:c.G1397T:p.R466L,PXK:NM_001349521:exon19:c.G1394T:p.R465L	.	1.052	.	.	.	Fam106_f_m_aM	0/1;0/0;0/1
chr3	58430809	58430809	C	T	PDHB	exonic	nonsynonymous SNV	PDHB:NM_001315536:exon5:c.G383A:p.G128E,PDHB:NM_000925:exon6:c.G437A:p.G146E	.	1.418	.	.	.	Fam121_f_m_aF_aM	0/1;0/0;0/0;0/1
chr3	58430855	58430855	A	T	PDHB	exonic	nonsynonymous SNV	PDHB:NM_001315536:exon5:c.T337A:p.Y113N,PDHB:NM_000925:exon6:c.T391A:p.Y131N,PDHB:NM_001173468:exon6:c.T391A:p.Y131N	.	1.484	.	.	.	Fam44_f_m_aM_uF	0/1;0/0;0/1;0/0
chr3	64019411	64019411	A	C	PSMD6	exonic	nonsynonymous SNV	PSMD6:NM_001271780:exon3:c.T268G:p.Y90D,PSMD6:NM_001271781:exon3:c.T265G:p.Y89D,PSMD6:NM_014814:exon3:c.T382G:p.Y128D,PSMD6:NM_001271779:exon4:c.T541G:p.Y181D	.	1.735	.	1.12E-05	.	Fam7_f_m_aM_aM_uM	0/1;0/0;0/1;0/1;0/1
chr3	64023343	64023343	A	C	PSMD6	exonic	nonsynonymous SNV	PSMD6:NM_001271779:exon1:c.T77G:p.L26R,PSMD6:NM_014814:exon1:c.T77G:p.L26R	.	1.673	.	.	.	Fam70_f_m_aM	0/0;0/1;0/1
chr3	64147517	64147517	C	T	PRICKLE2	exonic	nonsynonymous SNV	PRICKLE2:NM_001370528:exon7:c.G973A:p.A325T,PRICKLE2:NM_198859:exon7:c.G973A:p.A325T	.	1.347	.	9.83E-05	3.49E-05	Fam56_f_m_aF_aM	0/1;0/0;0/0;0/1
chr3	64604091	64604097	TGGG	-	ADAMTS9	splicing	.	.	0.47	.	.	6.71E-05	3.49E-05	Fam101_f_m_aM	0/0;0/1;0/1
chr3	69056810	69056810	A	-	UBA3	exonic	frameshift deletion	UBA3:NM_001363861:exon11:c.847delT:p.C283Vfs*8,UBA3:NM_198195:exon12:c.928delT:p.C310Vfs*8,UBA3:NM_003968:exon13:c.970delT:p.C324Vfs*8	0.437	.	.	.	.	Fam115_f_m_aF_aM_aF	0/0;0/1;0/0;0/1;0/1
chr3	73624208	73624208	C	G	PDZRN3	exonic	nonsynonymous SNV	PDZRN3:NM_015009:exon1:c.G618C:p.Q206H	.	1.328	.	.	.	Fam95_f_m_aM_aM_uF	0/0;0/1;0/1;0/1;0/0
chr3	98581407	98581407	C	T	CPOX	exonic	nonsynonymous SNV	CPOX:NM_000097:exon6:c.G1277A:p.R426Q	.	1.55	.	.	.	Fam30_f_m_aM_uM	0/1;0/0;0/1;0/0
chr3	101328508	101328508	G	C	SENP7	exonic	nonsynonymous SNV	SENP7:NM_001282803:exon19:c.C2342G:p.S781C,SENP7:NM_001077203:exon21:c.C2639G:p.S880C,SENP7:NM_001282801:exon21:c.C2636G:p.S879C,SENP7:NM_001282802:exon21:c.C2735G:p.S912C,SENP7:NM_020654:exon22:c.C2834G:p.S945C	.	1.914	.	0.0002	6.98E-05	Fam28_f_m_aF_uF	0/0;0/1;0/1;0/1
chr3	105751475	105751475	G	A	CBLB	exonic	nonsynonymous SNV	CBLB:NM_001321796:exon4:c.C563T:p.T188I,CBLB:NM_001321799:exon4:c.C563T:p.T188I,CBLB:NM_001321786:exon5:c.C794T:p.T265I,CBLB:NM_001321788:exon5:c.C710T:p.T237I,CBLB:NM_001321789:exon5:c.C794T:p.T265I,CBLB:NM_001321790:exon5:c.C776T:p.T259I,CBLB:NM_001321791:exon5:c.C710T:p.T237I,CBLB:NM_001321793:exon5:c.C710T:p.T237I,CBLB:NM_001321794:exon5:c.C710T:p.T237I,CBLB:NM_001321795:exon5:c.C710T:p.T237I,CBLB:NM_001321797:exon5:c.C710T:p.T237I,CBLB:NM_001321798:exon5:c.C710T:p.T237I,CBLB:NM_170662:exon5:c.C710T:p.T237I	.	1.502	.	1.12E-05	.	Fam50_f_m_aF	0/1;0/0;0/1
chr3	113279353	113279353	C	T	BOC	exonic	nonsynonymous SNV	BOC:NM_001301861:exon12:c.C1921T:p.R641C,BOC:NM_033254:exon12:c.C1918T:p.R640C	.	1.006	.	3.35E-05	.	Fam113_f_m_aF_raM_uF	0/0;0/0;0/0;0/1;0/0
chr3	114171811	114171811	T	G	DRD3	exonic	nonsynonymous SNV	DRD3:NM_000796:exon2:c.A182C:p.Q61P,DRD3:NM_033663:exon2:c.A182C:p.Q61P,DRD3:NM_001282563:exon3:c.A182C:p.Q61P,DRD3:NM_001290809:exon3:c.A182C:p.Q61P	.	1.155	.	.	.	Fam7_f_m_aM_aM_uM	0/0;0/1;0/1;0/1;0/1
chr3	114350526	114350526	C	T	ZBTB20	exonic	nonsynonymous SNV	ZBTB20:NM_001164342:exon4:c.G1552A:p.G518S,ZBTB20:NM_001164346:exon5:c.G1333A:p.G445S,ZBTB20:NM_001164347:exon5:c.G1333A:p.G445S,ZBTB20:NM_001164345:exon6:c.G1333A:p.G445S,ZBTB20:NM_001164344:exon7:c.G1333A:p.G445S,ZBTB20:NM_001348804:exon9:c.G1333A:p.G445S,ZBTB20:NM_015642:exon9:c.G1333A:p.G445S,ZBTB20:NM_001348802:exon10:c.G1333A:p.G445S,ZBTB20:NM_001348805:exon10:c.G1333A:p.G445S,ZBTB20:NM_001164343:exon11:c.G1333A:p.G445S,ZBTB20:NM_001348800:exon11:c.G1552A:p.G518S,ZBTB20:NM_001348801:exon11:c.G1333A:p.G445S,ZBTB20:NM_001348803:exon13:c.G1552A:p.G518S	.	2.337	.	1.12E-05	6.98E-06	Fam69_f_m_aM	0/1;0/0;0/1
chr3	115676160	115676160	C	T	GAP43	exonic	stopgain	GAP43:NM_002045:exon2:c.C178T:p.Q60X,GAP43:NM_001130064:exon3:c.C286T:p.Q96X	0.358	.	.	.	.	Fam48_f_m_aM_uM	0/0;0/1;0/1;0/0
chr3	120335947	120335947	C	A	LRRC58	exonic	nonsynonymous SNV	LRRC58:NM_001099678:exon2:c.G507T:p.E169D	.	1.82	0.0001	6.61E-05	2.79E-05	Fam25_f_m_aM	0/1;0/0;0/1
chr3	121545871	121545871	G	T	POLQ	exonic	nonsynonymous SNV	POLQ:NM_199420:exon1:c.C7A:p.L3I	.	1.084	0.0001	2.25E-05	6.98E-06	Fam31_f_m_aM_uF	0/1;0/0;0/1;0/1
chr3	122283867	122283867	G	A	CASR	exonic	nonsynonymous SNV	CASR:NM_000388:exon7:c.G1913A:p.R638H,CASR:NM_001178065:exon7:c.G1943A:p.R648H	.	1.215	7.35E-05	0.0002	4.19E-05	Fam121_f_m_aF_aM	0/1;0/0;0/1;0/1
chr3	122795254	122795254	C	T	SLC49A4	exonic	nonsynonymous SNV	SLC49A4:NM_032839:exon1:c.C62T:p.P21L	.	1.701	.	.	.	Fam72_f_m_aF_uF	0/1;0/0;0/1;0/0

chr3	123494927	123494927	C	A	HACD2	exonic	nonsynonymous SNV	HACD2:NM_001329786:exon6:c.G393T:p.K131N,HACD2:NM_001329787:exon7:c.G393T:p.K131N,HACD2:NM_198402:exon7:c.G726T:p.K242N,HACD2:NM_001329783:exon8:c.G795T:p.K265N,HACD2:NM_001329784:exon9:c.G447T:p.K149N	.	1.76	.	.	.		Fam64_f_m_aM	0/1;0/0;0/1
chr3	123618765	123618765	C	T	MYLK	exonic	nonsynonymous SNV	MYLK:NM_053031:exon2:c.G91A:p.V31M,MYLK:NM_053032:exon2:c.G94A:p.V32M,MYLK:NM_053028:exon31:c.G5014A:p.V1672M,MYLK:NM_001321309:exon32:c.G4846A:p.V1616M,MYLK:NM_053026:exon32:c.G5167A:p.V1723M,MYLK:NM_053027:exon32:c.G5221A:p.V1741M,MYLK:NM_053025:exon33:c.G5374A:p.V1792M	.	1.799	.	3.35E-05	.	Fam67_f_m_aM_uF	0/1;0/0;0/1;0/0
chr3	124730495	124730495	G	C	UMPS	exonic	nonsynonymous SNV	UMPS:NM_000373:exon1:c.G24C:p.L8F	.	2.173	.	2.23E-05	.	Fam91_m_aM_dM_aM_dM	0/1;0/1;0/0;0/1;0/0
chr3	126475240	126475240	T	C	ZXDC	exonic	nonsynonymous SNV	ZXDC:NM_001040653:exon1:c.A626G:p.K209R,ZXDC:NM_025112:exon1:c.A626G:p.K209R	.	1.818	0.001	0.0008	0.0006	Fam94_f_m_aM	0/0;0/1;0/1
chr3	126542046	126542046	G	A	CHST13	exonic	nonsynonymous SNV	CHST13:NM_152889:exon3:c.G494A:p.R165H	.	1.884	.	2.61E-05	.	Fam42_f_m_aM_uF	0/0;0/1;0/1;0/0
chr3	126542448	126542448	C	T	CHST13	exonic	nonsynonymous SNV	CHST13:NM_152889:exon3:c.C896T:p.A299V	.	1.089	.	.	6.99E-06	Fam36_f_m_aM_uM	0/0;0/1;0/1;0/1
chr3	127030357	127030357	G	T	PLXNA1	exonic	nonsynonymous SNV	PLXNA1:NM_032242:exon28:c.G5176T:p.A1726S	.	2.031	.	.	.	Fam121_f_m_aF_aM	0/1;0/0;0/1;0/1
chr3	127033955	127033955	C	T	PLXNA1	exonic	nonsynonymous SNV	PLXNA1:NM_032242:exon31:c.C5629T:p.R1877W	.	2.066	.	3.54E-05	.	Fam97_f_m_aM_aF	0/0;0/1;0/1;0/0
chr3	127033971	127033971	G	A	PLXNA1	exonic	nonsynonymous SNV	PLXNA1:NM_032242:exon31:c.G5645A:p.R1882Q	.	1.838	.	3.44E-05	6.98E-06	Fam14_f_m_aM_aM	0/1;0/0;0/1;0/1
chr3	127608455	127608455	G	A	MCM2	exonic	nonsynonymous SNV	MCM2:NM_004526:exon7:c.G1175A:p.R392H	.	1.222	.	0.0003	2.09E-05	Fam15_f_m_aM_aM	0/1;0/0;0/0;0/1
chr3	129557129	129557129	G	A	PLXND1	exonic	nonsynonymous SNV	PLXND1:NM_015103:exon34:c.C5540T:p.P1847L	.	1.656	.	1.12E-05	.	Fam30_f_m_aM_uM	0/1;0/0;0/1;0/0
chr3	129605486	129605486	G	C	PLXND1	exonic	nonsynonymous SNV	PLXND1:NM_015103:exon1:c.C1154G:p.P385R	.	1.225	.	3.50E-05	.	Fam59_f_m_aF_uM	0/0;0/1;0/1;0/0
chr3	129605646	129605646	T	G	PLXND1	exonic	nonsynonymous SNV	PLXND1:NM_015103:exon1:c.A994C:p.K332Q	.	2.251	.	.	.	Fam26_f_m_aM_uM	0/1;0/0;0/1;0/1
chr3	129606285	129606285	G	T	PLXND1	exonic	nonsynonymous SNV	PLXND1:NM_015103:exon1:c.C355A:p.R119S	.	1.292	.	.	.	Fam39_f_m_aM	0/0;0/1;0/1
chr3	130733634	130733634	T	C	PIK3R4	exonic	nonsynonymous SNV	PIK3R4:NM_014602:exon4:c.A1364G:p.D455G	.	1.636	.	.	.	Fam13_f_m_aM	0/0;0/1;0/1
chr3	130744696	130744696	G	C	PIK3R4	exonic	nonsynonymous SNV	PIK3R4:NM_014602:exon2:c.C523G:p.L175V	.	1.033	.	.	.	Fam83_f_m_aF	0/0;0/1;0/1
chr3	131381907	131381907	C	A	NUDT16	exonic	nonsynonymous SNV	NUDT16:NM_001171906:exon1:c.C103A:p.L35I,NUDT16:NM_152395:exon1:c.C103A:p.L35I	.	1.273	.	.	.	Fam31_f_m_aM_uF	0/1;0/0;0/1;0/1
chr3	135249409	135249409	G	A	EPHB1	exonic	nonsynonymous SNV	EPHB1:NM_004441:exon15:c.G2764A:p.A922T	.	1.268	.	0.0002	6.98E-06	Fam51_f_m_aM_uF	0/0;0/1;0/1;0/1
chr3	138223477	138223477	G	A	ARMC8	exonic	nonsynonymous SNV	ARMC8:NM_001282342:exon3:c.G157A:p.E53K,ARMC8:NM_001267041:exon4:c.G283A:p.E95K,ARMC8:NM_001267042:exon4:c.G283A:p.E95K,ARMC8:NM_001363941:exon4:c.G283A:p.E95K,ARMC8:NM_001363942:exon4:c.G157A:p.E53K,ARMC8:NM_014154:exon5:c.G241A:p.E81K,ARMC8:NM_015396:exon5:c.G241A:p.E81K,ARMC8:NM_213654:exon5:c.G241A:p.E81K	.	1.31	.	1.12E-05	6.98E-06	Fam15_f_m_aM_aM	0/0;0/1;0/0;0/1
chr3	138714532	138714532	G	A	PIK3CB	exonic	nonsynonymous SNV	PIK3CB:NM_006219:exon9:c.C1238T:p.T413M	.	1.234	.	3.27E-05	.	Fam116_f_m_aM_aF	0/1;0/0;0/1;0/1
chr3	141444040	141444040	C	G	ZBTB38	exonic	nonsynonymous SNV	ZBTB38:NM_001350100:exon4:c.C1652G:p.T551R,ZBTB38:NM_001350099:exon6:c.C1652G:p.T551R,ZBTB38:NM_001080412:exon8:c.C1652G:p.T551R	.	1.302	.	.	.	Fam24_f_m_aM_aM	0/0;0/1;0/1;0/0
chr3	141445797	141445797	G	C	ZBTB38	exonic	nonsynonymous SNV	ZBTB38:NM_001350100:exon4:c.G3409C:p.A1137P,ZBTB38:NM_001350099:exon6:c.G3409C:p.A1137P,ZBTB38:NM_001080412:exon8:c.G3409C:p.A1137P	.	1.436	0.0004	0.0002	0.0001	Fam57_f_m_aM_uF	0/0;0/1;0/1;0/1
chr3	141608540	141608540	T	A	RASA2	exonic	nonsynonymous SNV	RASA2:NM_001303245:exon21:c.T2071A:p.C691S,RASA2:NM_006506:exon21:c.T2068A:p.C690S,RASA2:NM_001303246:exon22:c.T2080A:p.C694S	.	1.234	.	1.12E-05	.	Fam20_f_m_aM_uF	0/0;0/1;0/1;0/1
chr3	142459258	142459258	A	G	ATR	exonic	nonsynonymous SNV	ATR:NM_001354579:exon42:c.T7126C:p.F2376L,ATR:NM_001184:exon43:c.T7318C:p.F2440L	.	1.765	.	.	.	Fam18_f_m_aM_uF	0/0;0/1;0/1;0/0
chr3	142780946	142780946	G	A	TRPC1	exonic	nonsynonymous SNV	TRPC1:NM_003304:exon5:c.G775A:p.E259K,TRPC1:NM_001251845:exon6:c.G877A:p.E293K	.	1.44	7.35E-05	.	1.40E-05	Fam6_f_m_aM	0/1;0/0;0/1
chr3	142962783	142962783	G	C	PAQR9	exonic	nonsynonymous SNV	PAQR9:NM_198504:exon1:c.C554G:p.A185G	.	1.817	.	0.0001	2.79E-05	Fam118_f_m_aM_aF_uM	0/0;0/1;0/1;0/0;0/1
chr3	143989627	143989627	A	T	DIPK2A	exonic	nonsynonymous SNV	DIPK2A:NM_001134470:exon3:c.A452T:p.Y151F,DIPK2A:NM_001363944:exon3:c.A365T:p.Y122F,DIPK2A:NM_173552:exon3:c.A1079T:p.Y360F	.	1.309	.	.	.	Fam68_f_m_aF_uF_uM	0/0;0/1;0/1;0/1;0/0
chr3	147396245	147396245	C	T	ZIC4	exonic	nonsynonymous SNV	ZIC4:NM_001168378:exon3:c.G445A:p.G149R,ZIC4:NM_001168379:exon3:c.G409A:p.G137R,ZIC4:NM_032153:exon3:c.G295A:p.G99R	.	1.566	.	.	1.40E-05	Fam121_f_m_aF_aM	0/1;0/0;0/1;0/1
chr3	149751431	149751431	T	C	COMMD2	exonic	nonsynonymous SNV	COMMD2:NM_016094:exon3:c.A200G:p.Y67C	.	1.246	0.001	0.0007	0.0003	Fam40_f_m_aM_aM	0/0;0/1;0/1;0/1
chr3	149751431	149751431	T	C	COMMD2	exonic	nonsynonymous SNV	COMMD2:NM_016094:exon3:c.A200G:p.Y67C	.	1.246	0.001	0.0007	0.0003	Fam6_f_m_aM	0/1;0/0;0/1
chr3	150762770	150762770	G	C	SIAH2	exonic	nonsynonymous SNV	SIAH2:NM_005067:exon1:c.C80G:p.P27R	.	1.01	0.0005	0.0005	0.0005	Fam117_f_m_aM_aF	0/1;0/0;0/1;0/0
chr3	151188477	151188477	T	G	MED12L	exonic	nonsynonymous SNV	MED12L:NM_053002:exon12:c.T1750G:p.L584V	.	1.217	.	.	.	Fam14_f_m_aM_aM	0/0;0/1;0/1;0/0
chr3	155485571	155485571	C	A	PLCH1	exonic	nonsynonymous SNV	PLCH1:NM_014996:exon21:c.G2759T:p.G920V,PLCH1:NM_001130960:exon22:c.G2783T:p.G928V,PLCH1:NM_001130961:exon22:c.G2783T:p.G928V,PLCH1:NM_001349250:exon22:c.G2783T:p.G928V,PLCH1:NM_001349251:exon22:c.G2759T:p.G920V,PLCH1:NM_001349252:exon23:c.G2819T:p.G940V	.	1.307	.	.	.	Fam92_f_m_aM_raM	0/0;0/0;0/0;0/1
chr3	155494497	155494497	A	T	PLCH1	exonic	nonsynonymous SNV	PLCH1:NM_001130960:exon15:c.T1879A:p.S627T,PLCH1:NM_001130961:exon15:c.T1879A:p.S627T,PLCH1:NM_001349250:exon15:c.T1879A:p.S627T,PLCH1:NM_014996:exon15:c.T1915A:p.S639T,PLCH1:NM_001349251:exon16:c.T1915A:p.S639T,PLCH1:NM_001349252:exon16:c.T1915A:p.S639T	.	1.108	0.0003	0.0009	9.88E-05	Fam108_f_m_aM	0/0;0/1;0/1

chr3	159866202	159866202	G	A	IQCJ-SCHIP1;SCHII	exonic	nonsynonymous SNV	SCHIP1:NM_001197108:exon2:c.G103A:p.A35T,SCHIP1:NM_001197109:exon2:c.G70A:p.A24T,SCHIP1:NM_001197107:exon3:c.G760A:p.A254T,SCHIP1:NM_014575:exon3:c.G799A:p.A267T,IQCJ-SCHIP1:NM_001197114:exon5:c.G946A:p.A316T,IQCJ-SCHIP1:NM_001197113:exon6:c.G1027A:p.A343T	.	1.172	.	.	.		Fam76_f_m_aM_uM	0/0;0/1;0/1;0/0
chr3	169966855	169966855	C	G	SEC62	exonic	nonsynonymous SNV	SEC62:NM_003262:exon1:c.C33G:p.I11M	.	1.038	0.0004	0.0005	0.0003	Fam82_f_m_aM_uF	0/1;0/0;0/1;0/1
chr3	171110795	171110795	T	G	TNIK	exonic	nonsynonymous SNV	TNIK:NM_001161565:exon17:c.A1951C:p.T651P,TNIK:NM_001161566:exon17:c.A1951C:p.T651P,TNIK:NM_001161561:exon18:c.A2116C:p.T706P,TNIK:NM_001161562:exon18:c.A2116C:p.T706P,TNIK:NM_001161563:exon18:c.A2038C:p.T680P,TNIK:NM_001161564:exon18:c.A2038C:p.T680P,TNIK:NM_001161560:exon19:c.A2203C:p.T735P,TNIK:NM_015028:exon19:c.A2203C:p.T735P	.	1.191	7.35E-05	9.71E-05	2.79E-05	Fam47_f_m_aM	0/1;0/0;0/1
chr3	173605072	173605072	A	G	NLGN1	exonic	nonsynonymous SNV	NLGN1:NM_001365923:exon2:c.A474G:p.I158M,NLGN1:NM_001365924:exon2:c.A474G:p.I158M,NLGN1:NM_001365925:exon2:c.A474G:p.I158M,NLGN1:NM_001365926:exon2:c.A474G:p.I158M,NLGN1:NM_001365927:exon2:c.A474G:p.I158M,NLGN1:NM_001365928:exon2:c.A474G:p.I158M,NLGN1:NM_001365929:exon2:c.A474G:p.I158M,NLGN1:NM_001365930:exon2:c.A474G:p.I158M,NLGN1:NM_001365931:exon2:c.A474G:p.I158M,NLGN1:NM_001365932:exon2:c.A474G:p.I158M,NLGN1:NM_001365933:exon2:c.A474G:p.I158M,NLGN1:NM_001365934:exon2:c.A474G:p.I158M,NLGN1:NM_001365935:exon2:c.A474G:p.I158M,NLGN1:NM_001365936:exon2:c.A474G:p.I158M,NLGN1:NM_014932:exon3:c.A474G:p.I158M	.	1.122	.	0.0009	3.49E-05	Fam17_f_m_aM_uM	0/1;0/0;0/1;0/1
chr3	179416548	179416548	A	C	GNB4	exonic	nonsynonymous SNV	GNB4:NM_021629:exon5:c.T212G:p.V71G	.	1.711	.	.	.	Fam59_f_m_aF_uM	0/0;0/1;0/1;0/1
chr3	179708954	179708954	G	C	USP13	exonic	nonsynonymous SNV	USP13:NM_003940:exon6:c.G802C:p.A268P	.	1.126	.	1.12E-05	.	Fam112_f_m_aM	0/0;0/1;0/1
chr3	179721530	179721530	C	G	USP13	exonic	nonsynonymous SNV	USP13:NM_003940:exon8:c.C1029G:p.N343K	.	1.044	.	1.12E-05	.	Fam60_f_m_aF	0/0;0/1;0/1
chr3	179764014	179764014	C	T	USP13	exonic	nonsynonymous SNV	USP13:NM_003940:exon18:c.C2105T:p.P702L	.	1.008	.	1.13E-05	1.40E-05	Fam96_f_m_aM_uF	0/1;0/0;0/1;0/0
chr3	181712424	181712424	G	A	SOX2	exonic	nonsynonymous SNV	SOX2:NM_003106:exon1:c.G64A:p.G22S	.	1.242	7.38E-05	7.81E-05	0.0001	Fam84_f_m_aF	0/1;0/0;0/1
chr3	184165486	184165486	A	G	DVL3	exonic	nonsynonymous SNV	DVL3:NM_004423:exon7:c.A758G:p.N253S	.	1.503	0.0002	0.0002	0.0002	Fam122_f_m_aM	0/0;0/1;0/1
chr3	184165486	184165486	A	G	DVL3	exonic	nonsynonymous SNV	DVL3:NM_004423:exon7:c.A758G:p.N253S	.	1.503	0.0002	0.0002	0.0002	Fam69_f_m_aM	0/0;0/1;0/1
chr3	184237338	184237338	T	C	VWA5B2	exonic	nonsynonymous SNV	VWA5B2:NM_001320373:exon9:c.T989C:p.L330P,VWA5B2:NM_138345:exon11:c.T1646C:p.L549P	.	2.04	.	.	.	Fam12_f_m_aM_uM_aM	0/1;0/0;0/1;0/1;0/0
chr3	184327296	184327296	G	A	EIF4G1	exonic	nonsynonymous SNV	EIF4G1:NM_004953:exon17:c.G2924A:p.R975H,EIF4G1:NM_198242:exon20:c.G3017A:p.R1006H,EIF4G1:NM_198244:exon21:c.G3248A:p.R1083H,EIF4G1:NM_182917:exon23:c.G3512A:p.R1171H,EIF4G1:NM_001194947:exon24:c.G3530A:p.R1177H,EIF4G1:NM_001291157:exon24:c.G3389A:p.R1130H,EIF4G1:NM_198241:exon24:c.G3509A:p.R1170H,EIF4G1:NM_001194946:exon25:c.G3530A:p.R1177H	.	1.061	0.0001	0.0006	1.40E-05	Fam35_f_m_aF_uM	0/1;0/0;0/1;0/1
chr3	184344707	184344707	C	G	FAM131A	exonic	nonsynonymous SNV	FAM131A:NM_001366133:exon5:c.C583G:p.L195V,FAM131A:NM_001171093:exon6:c.C583G:p.L195V,FAM131A:NM_144635:exon6:c.C838G:p.L280V,FAM131A:NM_001366134:exon7:c.C583G:p.L195V	.	1.19	0.0007	0.0003	0.0002	Fam70_f_m_aM	0/0;0/1;0/1
chr3	184386125	184386125	C	T	CHRD	exonic	nonsynonymous SNV	CHRD:NM_001304472:exon15:c.C1898T:p.T633I,CHRD:NM_001304474:exon15:c.C788T:p.T263I,CHRD:NM_003741:exon15:c.C1898T:p.T633I,CHRD:NM_001304473:exon16:c.C788T:p.T263I	.	1.068	0.0003	0.0008	0.0003	Fam66_f_m_aM	0/0;0/1;0/1
chr3	184579539	184579539	C	T	EPHB3	exonic	nonsynonymous SNV	EPHB3:NM_004443:exon10:c.C1864T:p.R622W	.	1.66	.	.	.	Fam100_f_m_aF	0/1;0/0;0/1
chr3	185450055	185450055	C	T	MAP3K13	exonic	nonsynonymous SNV	MAP3K13:NM_001242317:exon5:c.C545T:p.T182M,MAP3K13:NM_004721:exon6:c.C1166T:p.T389M,MAP3K13:NM_001242314:exon7:c.C1166T:p.T389M	.	2.06	.	6.31E-05	1.40E-05	Fam48_f_m_aM_uM	0/1;0/0;0/1;0/1
chr3	185586417	185586417	T	C	SENP2	exonic	nonsynonymous SNV	SENP2:NM_021627:exon1:c.T4C:p.Y2H	.	1.438	.	.	6.98E-06	Fam54_f_m_aM_uF	0/0;0/1;0/1;0/1
chr3	186048727	186048727	G	A	ETV5	exonic	nonsynonymous SNV	ETV5:NM_004454:exon13:c.C1445T:p.P482L	.	1.239	.	.	.	Fam82_f_m_aM_uF	0/0;0/1;0/1;0/1
chr3	186581462	186581462	G	A	DNAJB11	exonic	nonsynonymous SNV	DNAJB11:NM_016306:exon5:c.G548A:p.G183E	.	1.768	.	.	.	Fam12_f_m_aM_uM_aM	0/1;0/0;0/0;0/0;0/1
chr3	186786601	186786601	A	G	EIF4A2	exonic	nonsynonymous SNV	EIF4A2:NM_001967:exon7:c.A727G:p.T243A	.	1.688	.	.	.	Fam110_f_m_aM_aM_uMs	0/0;0/0;0/0;0/1;0/0
chr3	187733578	187733578	C	T	BCL6	exonic	nonsynonymous SNV	BCL6:NM_001134738:exon2:c.G116A:p.S39N,BCL6:NM_001130845:exon3:c.G116A:p.S39N,BCL6:NM_001706:exon3:c.G116A:p.S39N	.	1.114	.	9.83E-05	.	Fam38_f_m_aM	0/1;0/0;0/1
chr3	193617212	193617212	C	-	OPA1	exonic	stopgain	OPA1:NM_015560:exon4:c.483delC:p.V163*,OPA1:NM_130834:exon4:c.483delC:p.V163*,OPA1:NM_130836:exon4:c.483delC:p.V163*,OPA1:NM_130837:exon4:c.483delC:p.V163*,OPA1:NM_001354664:exon5:c.111delC:p.V39*	0.472	.	.	.	.	Fam91_m_aM_dM_aM_dM	0/0;0/1;0/1;0/0;0/0
chr3	193654936	193654936	C	T	OPA1	exonic	nonsynonymous SNV	OPA1:NM_130831:exon19:c.C1814T:p.A605V,OPA1:NM_015560:exon20:c.C1922T:p.A641V,OPA1:NM_130832:exon20:c.C1868T:p.A623V,OPA1:NM_130833:exon20:c.C1925T:p.A642V,OPA1:NM_001354663:exon21:c.C1553T:p.A518V,OPA1:NM_001354664:exon21:c.C1550T:p.A517V,OPA1:NM_130834:exon21:c.C1976T:p.A659V,OPA1:NM_130835:exon21:c.C1979T:p.A660V,OPA1:NM_130836:exon21:c.C2033T:p.A678V,OPA1:NM_130837:exon22:c.C2087T:p.A696V	.	1.082	.	3.28E-05	.	Fam62_f_m_aM_aM	0/0;0/1;0/1;0/1

chr3	195307311	195307311	C	T	ACAP2	exonic	nonsynonymous SNV	ACAP2:NM_012287:exon12:c.G922A:p.V308M	.	1.655	.	.	.	Fam7_f_m_aM_aM_uM	0/0;0/1;0/1;0/0;0/0
chr3	195883222	195883222	G	A	TNK2	exonic	nonsynonymous SNV	TNK2:NM_001010938:exon5:c.C616T:p.H206Y,TNK2:NM_001308046:exon5:c.C640T:p.H214Y,TNK2:NM_005781:exon5:c.C544T:p.H182Y	.	1.033	7.36E-05	2.24E-05	.	Fam79_f_m_aM_uM	0/0;0/1;0/1;0/0
chr3	195888497	195888497	T	C	TNK2	exonic	nonsynonymous SNV	TNK2:NM_001010938:exon2:c.A164G:p.N55S,TNK2:NM_001308046:exon2:c.A188G:p.N63S,TNK2:NM_005781:exon2:c.A92G:p.N31S	.	1.075	.	0.0002	1.40E-05	Fam31_f_m_aM_uF	0/1;0/0;0/1;0/0
chr3	195888509	195888509	C	T	TNK2	exonic	nonsynonymous SNV	TNK2:NM_001010938:exon2:c.G152A:p.R51Q,TNK2:NM_001308046:exon2:c.G176A:p.R59Q,TNK2:NM_005781:exon2:c.G80A:p.R27Q	.	1.116	0.0003	9.84E-05	4.89E-05	Fam98_f_m_aM	0/1;0/0;0/1
chr3	196306971	196306971	T	A	TCTEX1D2	exonic	nonsynonymous SNV	TCTEX1D2:NM_001351628:exon3:c.A289T:p.I97F,TCTEX1D2:NM_152773:exon3:c.A289T:p.I97F	.	1.502	0.0002	0.0005	0.0001	Fam80_f_m_aM_uM	0/1;0/0;0/1;0/1
chr3	196569241	196569241	G	A	FBXO45	exonic	nonsynonymous SNV	FBXO45:NM_001105573:exon1:c.G257A:p.S86N	.	1.105	.	.	6.98E-06	Fam89_f_m_aM	0/1;0/0;0/1
chr4	1406108	1406108	G	A	NKX1-1	exonic	nonsynonymous SNV	NKX1-1:NM_001290079:exon1:c.C335T:p.A112V	.	1.09	.	.	.	Fam20_f_m_aM_uF	0/0;0/1;0/1;0/1
chr4	1959555	1959555	A	C	NSD2	exonic	nonsynonymous SNV	NSD2:NM_001042424:exon17:c.A3070C:p.N1024H,NSD2:NM_133335:exon17:c.A3070C:p.N1024H,NSD2:NM_133331:exon18:c.A3070C:p.N1024H,NSD2:NM_133330:exon19:c.A3070C:p.N1024H	.	1.607	.	.	.	Fam83_f_m_aF	0/1;0/0;0/1
chr4	3206610	3206610	C	T	HTT	exonic	nonsynonymous SNV	HTT:NM_002111:exon43:c.C5833T:p.R1945W	.	1.205	0.0001	4.49E-05	2.09E-05	Fam28_f_m_aF_uF	0/1;0/0;0/1;0/0
chr4	3207344	3207344	G	A	HTT	exonic	nonsynonymous SNV	HTT:NM_002111:exon45:c.G6139A:p.G2047R	.	1.297	0.0002	0.0004	0.0002	Fam48_f_m_aM_uM	0/1;0/0;0/1;0/1
chr4	3223427	3223427	A	T	HTT	exonic	nonsynonymous SNV	HTT:NM_002111:exon55:c.A7492T:p.I2498F	.	1.002	.	3.53E-05	6.98E-06	Fam100_f_m_aF	0/0;0/1;0/1
chr4	5821831	5821831	T	G	CRMP1	exonic	nonsynonymous SNV	CRMP1:NM_001014809:exon14:c.A1990C:p.N664H,CRMP1:NM_001288661:exon14:c.A1642C:p.N548H,CRMP1:NM_001288662:exon14:c.A1630C:p.N544H,CRMP1:NM_001313:exon14:c.A1648C:p.N550H	.	1.277	.	.	.	Fam84_f_m_aF	0/0;0/1;0/1
chr4	6716314	6716314	G	C	BLOC1S4	exonic	nonsynonymous SNV	BLOC1S4:NM_018366:exon1:c.G105C:p.Q35H	.	1.784	.	.	.	Fam97_f_m_aM_aF	0/1;0/0;0/0;0/1
chr4	6716819	6716819	A	T	BLOC1S4	exonic	nonsynonymous SNV	BLOC1S4:NM_018366:exon1:c.A610T:p.T204S	.	1.741	.	.	.	Fam101_f_m_aM	0/0;0/1;0/1
chr4	8868064	8868064	G	A	HMX1	exonic	nonsynonymous SNV	HMX1:NM_018942:exon2:c.C676T:p.R226C	.	2.368	0.0003	0.0001	0.0001	Fam12_f_m_aM_uM_aM	0/1;0/0;0/1;0/1;0/1
chr4	10445801	10445802	CT	-	ZNF518B	exonic	frameshift deletion	ZNF518B:NM_053042:exon3:c.527_528del:p.E176Vfs*17	0.552	.	.	.	.	Fam10_f_m_aM_uF	0/0;0/0;0/1;0/0
chr4	15052491	15052491	C	T	CPEB2	exonic	nonsynonymous SNV	CPEB2:NM_001177384:exon5:c.C2173T:p.P725S,CPEB2:NM_182646:exon5:c.C2164T:p.P722S,CPEB2:NM_001177381:exon6:c.C2197T:p.P733S,CPEB2:NM_001177383:exon6:c.C2188T:p.P730S,CPEB2:NM_182485:exon6:c.C2254T:p.P752S,CPEB2:NM_001177382:exon7:c.C2278T:p.P760S	.	2.302	.	3.33E-05	.	Fam37_f_m_aF_uM	0/1;0/0;0/1;0/1
chr4	20532001	20532001	A	T	SLIT2	exonic	nonsynonymous SNV	SLIT2:NM_001289135:exon16:c.A1619T:p.E540V,SLIT2:NM_001289136:exon16:c.A1607T:p.E536V,SLIT2:NM_004787:exon17:c.A1631T:p.E544V	.	1.125	.	1.21E-05	.	Fam112_f_m_aM	0/0;0/1;0/1
chr4	20618819	20618819	G	T	SLIT2	exonic	nonsynonymous SNV	SLIT2:NM_001289135:exon36:c.G4388T:p.G1463V,SLIT2:NM_001289136:exon36:c.G4376T:p.G1459V,SLIT2:NM_004787:exon37:c.G4400T:p.G1467V	.	1.177	0.0002	0.0006	0.0002	Fam11_f_m_aM_uM	0/0;0/1;0/1;0/1
chr4	24799887	24799887	C	A	SOD3	exonic	nonsynonymous SNV	SOD3:NM_003102:exon2:c.C366A:p.S122R	.	2.155	.	0.0002	6.98E-05	Fam78_f_m_aF_uM	0/0;0/1;0/1;0/1
chr4	24876546	24876546	C	T	CCDC149	exonic	nonsynonymous SNV	CCDC149:NM_001130726:exon2:c.G215A:p.R72Q,CCDC149:NM_001330643:exon2:c.G215A:p.R72Q,CCDC149:NM_001330644:exon3:c.G50A:p.R17Q,CCDC149:NM_173463:exon3:c.G215A:p.R72Q	.	1.435	.	7.45E-05	4.19E-05	Fam55_f_m_aM_aM_dM	0/0;0/1;0/1;0/0;0/1
chr4	25019179	25019179	G	C	LGI2	exonic	nonsynonymous SNV	LGI2:NM_018176:exon5:c.C473G:p.S158C	.	1.36	0.0001	4.51E-05	1.40E-05	Fam73_f_m_aF_dM	0/1;0/0;0/1;0/0
chr4	37631411	37631411	T	-	RELL1	exonic	frameshift deletion	RELL1:NM_001085399:exon6:c.793delA:p.R265Efs*25,RELL1:NM_001085400:exon6:c.793delA:p.R265Efs*14	0.471	.	.	.	.	Fam42_f_m_aM_uF	0/1;0/0;0/1;0/0
chr4	39407716	39407716	C	G	KLB	exonic	nonsynonymous SNV	KLB:NM_175737:exon1:c.C767G:p.A256G	.	1.567	.	.	.	Fam77_f_m_aM_aF_uM_uFs	0/1;0/0;0/1;0/0;0/1;0/0
chr4	40437963	40437963	G	C	RBM47	exonic	nonsynonymous SNV	RBM47:NM_001371113:exon3:c.C931G:p.L311V,RBM47:NM_001371114:exon3:c.C817G:p.L273V,RBM47:NM_019027:exon3:c.C931G:p.L311V,RBM47:NM_001098634:exon4:c.C931G:p.L311V	.	1.203	.	.	.	Fam55_f_m_aM_aM_dM	0/0;0/1;0/1;0/0;0/1
chr4	41013700	41013701	CT	-	APBB2	exonic	frameshift deletion	APBB2:NM_001330658:exon5:c.717_718del:p.A241Qfs*17,APBB2:NM_001166050:exon6:c.717_718del:p.A241Qfs*17,APBB2:NM_001330656:exon6:c.717_718del:p.A241Qfs*17,APBB2:NM_004307:exon6:c.717_718del:p.A241Qfs*17,APBB2:NM_173075:exon6:c.717_718del:p.A241Qfs*17	0.541	.	.	3.27E-05	.	Fam117_f_m_aM_aF	0/1;0/0;0/0;0/1
chr4	41014332	41014332	C	G	APBB2	exonic	nonsynonymous SNV	APBB2:NM_001330658:exon5:c.G86C:p.R29P,APBB2:NM_001166050:exon6:c.G86C:p.R29P,APBB2:NM_001330656:exon6:c.G86C:p.R29P,APBB2:NM_004307:exon6:c.G86C:p.R29P,APBB2:NM_173075:exon6:c.G86C:p.R29P	.	1.234	.	.	.	Fam108_f_m_aM	0/0;0/1;0/1
chr4	41745878	41745878	C	A	PHOX2B	exonic	nonsynonymous SNV	PHOX2B:NM_003924:exon3:c.G874T:p.A292S	.	1.048	.	.	.	Fam5_f_m_aM	0/0;0/1;0/1
chr4	41982099	41982099	A	-	DCAF4L1	exonic	frameshift deletion	DCAF4L1:NM_001029955:exon1:c.307delA:p.S103Afs*5	0.295	.	.	.	6.98E-06	Fam31_f_m_aM_uF	0/1;0/0;0/1;0/0
chr4	42586451	42586451	T	C	ATP8A1	exonic	nonsynonymous SNV	ATP8A1:NM_001105529:exon9:c.A620G:p.K207R,ATP8A1:NM_006095:exon9:c.A620G:p.K207R	.	1.148	.	1.12E-05	.	Fam1_f_m_aM	0/1;0/0;0/1
chr4	48145475	48145475	C	A	TEC	exonic	nonsynonymous SNV	TEC:NM_003215:exon13:c.G1186T:p.A396S	.	1.06	.	4.47E-05	.	Fam113_f_m_aF_raM_uF	0/1;0/0;0/0;0/1;0/1
chr4	54101864	54101864	A	T	GSX2	exonic	nonsynonymous SNV	GSX2:NM_133267:exon2:c.A857T:p.D286V	.	1.887	.	.	.	Fam70_f_m_aM	0/1;0/0;0/1
chr4	55089802	55089802	C	T	KDR	exonic	nonsynonymous SNV	KDR:NM_002253:exon24:c.G3193A:p.A1065T	.	2.204	0.0003	0.0003	0.0004	Fam123_f_aF	0/1;0/1
chr4	56327781	56327781	G	T	KIAA1211	exonic	nonsynonymous SNV	KIAA1211:NM_020722:exon11:c.G3679T:p.D1227Y	.	1.042	.	.	.	Fam68_f_m_aF_uF_uM	0/0;0/1;0/1;0/1;0/0
chr4	57040840	57040840	A	C	IGFBP7	exonic	nonsynonymous SNV	IGFBP7:NM_001253835:exon2:c.T569G:p.V190G,IGFBP7:NM_001553:exon2:c.T569G:p.V190G	.	1.462	.	.	.	Fam62_f_m_aM_aM	0/0;0/1;0/1;0/1

chr4	70993909	70993909	C	T	DCK	exonic	nonsynonymous SNV	DCK:NM_000788:exon1:c.C74T:p.S25F	.	1.151	.	.	6.98E-06	Fam117_f_m_aM_aF	0/1;0/0;0/1;0/0
chr4	73125248	73125248	T	C	ANKRD17	exonic	nonsynonymous SNV	ANKRD17:NM_198889:exon16:c.A2546G:p.Q849R,ANKRD17:NM_001286771:exon17:c.A2960G:p.Q987R,ANKRD17:NM_015574:exon17:c.A3296G:p.Q1099R,ANKRD17:NM_032217:exon17:c.A3299G:p.Q1100R	.	1.038	.	.	.	Fam76_f_m_aM_uM	0/1;0/0;0/1;0/1
chr4	73142764	73142764	G	C	ANKRD17	exonic	nonsynonymous SNV	ANKRD17:NM_001286771:exon12:c.C1622G:p.A541G,ANKRD17:NM_015574:exon12:c.C1961G:p.A654G,ANKRD17:NM_032217:exon12:c.C1961G:p.A654G,ANKRD17:NM_198889:exon12:c.C1961G:p.A654G	.	2.232	.	3.55E-05	.	Fam11_f_m_aM_uM	0/1;0/0;0/1;0/0
chr4	74446701	74446701	T	G	AREG	exonic	nonsynonymous SNV	AREG:NM_001657:exon2:c.T229G:p.S77A	.	2.209	.	.	.	Fam51_f_m_aM_uF	0/0;0/1;0/1;0/0
chr4	75514219	75514219	T	C	RCHY1	exonic	nonsynonymous SNV	RCHY1:NM_001009922:exon1:c.A68G:p.Y23C,RCHY1:NM_001278536:exon1:c.A68G:p.Y23C,RCHY1:NM_001278537:exon1:c.A68G:p.Y23C,RCHY1:NM_015436:exon1:c.A68G:p.Y23C	.	1.514	.	1.12E-05	.	Fam110_f_m_aM_aM_uMs	0/1;0/0;0/1;0/0;0/1
chr4	76136389	76136389	G	C	NUP54	exonic	nonsynonymous SNV	NUP54:NM_001278603:exon3:c.C175G:p.P59A,NUP54:NM_017426:exon4:c.C319G:p.P107A	.	1.075	.	.	.	Fam26_f_m_aM_uM	0/0;0/1;0/1;0/0
chr4	76730823	76730823	C	T	SHROOM3	exonic	stopgain	SHROOM3:NM_020859:exon4:c.C475T:p.R159X	0.449	.	.	3.28E-05	.	Fam67_f_m_aM_uF	0/1;0/0;0/1;0/0
chr4	76730824	76730824	G	A	SHROOM3	exonic	nonsynonymous SNV	SHROOM3:NM_020859:exon4:c.G476A:p.R159Q	.	1.115	.	0.0002	1.40E-05	Fam100_f_m_aF	0/0;0/1;0/1
chr4	76739414	76739414	G	A	SHROOM3	exonic	nonsynonymous SNV	SHROOM3:NM_020859:exon5:c.G1241A:p.R414Q	.	1.119	7.34E-05	0.0007	7.68E-05	Fam81_f_m_aM_uM	0/0;0/1;0/1;0/1
chr4	84696034	84696034	T	C	WDFY3	exonic	nonsynonymous SNV	WDFY3:NM_014991:exon58:c.A8837G:p.N2946S	.	1.226	.	0.0002	0.0001	Fam40_f_m_aM_aM	0/0;0/1;0/1;0/0
chr4	94276457	94276457	C	T	SMARCAD1	exonic	nonsynonymous SNV	SMARCAD1:NM_001254949:exon7:c.C637T:p.H213Y,SMARCAD1:NM_001128429:exon15:c.C1927T:p.H643Y,SMARCAD1:NM_001128430:exon15:c.C1927T:p.H643Y,SMARCAD1:NM_020159:exon15:c.C1927T:p.H643Y	.	1.156	0.0008	0.001	0.0005	Fam43_f_m_aM	0/0;0/1;0/1
chr4	94276457	94276457	C	T	SMARCAD1	exonic	nonsynonymous SNV	SMARCAD1:NM_001254949:exon7:c.C637T:p.H213Y,SMARCAD1:NM_001128429:exon15:c.C1927T:p.H643Y,SMARCAD1:NM_001128430:exon15:c.C1927T:p.H643Y,SMARCAD1:NM_020159:exon15:c.C1927T:p.H643Y	.	1.156	0.0008	0.001	0.0005	Fam64_f_m_aM	0/0;0/1;0/1
chr4	95335598	95335598	A	G	UNC5C	exonic	nonsynonymous SNV	UNC5C:NM_003728:exon2:c.T158C:p.F53S	.	1.021	.	.	.	Fam102_f_m_aM	0/1;0/0;0/1
chr4	106925861	106925861	C	T	DKK2	exonic	nonsynonymous SNV	DKK2:NM_014421:exon2:c.G311A:p.R104Q	.	1.142	.	0.0001	7.11E-05	Fam45_f_m_aM_uF	0/1;0/0;0/1;0/1
chr4	112265009	112265009	C	A	AP1AR	exonic	nonsynonymous SNV	AP1AR:NM_001128426:exon6:c.C283A:p.Q95K,AP1AR:NM_018569:exon7:c.C382A:p.Q128K	.	1.298	0.0006	3.60E-05	7.00E-06	Fam95_f_m_aM_aM_uF	0/1;0/0;0/1;0/1;0/0
chr4	113902954	113902954	G	A	ARSJ	exonic	nonsynonymous SNV	ARSJ:NM_001354210:exon2:c.C1120T:p.L374F,ARSJ:NM_024590:exon2:c.C1120T:p.L374F,ARSJ:NM_001354211:exon6:c.C772T:p.L258F	.	1.13	.	3.37E-05	6.98E-06	Fam84_f_m_aF	0/1;0/0;0/1
chr4	113903650	113903650	G	A	ARSJ	exonic	nonsynonymous SNV	ARSJ:NM_001354210:exon2:c.C424T:p.H142Y,ARSJ:NM_024590:exon2:c.C424T:p.H142Y,ARSJ:NM_001354211:exon6:c.C76T:p.H26Y	.	1.161	.	3.29E-05	6.98E-06	Fam17_f_m_aM_uM	0/1;0/0;0/1;0/0
chr4	121818135	121818135	G	A	CCNA2	exonic	nonsynonymous SNV	CCNA2:NM_001237:exon7:c.C1159T:p.L387F	.	1.163	.	6.55E-05	1.40E-05	Fam66_f_m_aM	0/1;0/0;0/1
chr4	121932635	121932635	A	G	TRPC3	exonic	nonsynonymous SNV	TRPC3:NM_003305:exon1:c.T404C:p.L135P,TRPC3:NM_001130698:exon2:c.T623C:p.L208P,TRPC3:NM_001366479:exon2:c.T623C:p.L208P	.	1.645	.	.	.	Fam34_f_m_aM_uF	0/1;0/0;0/1;0/1
chr4	122174642	122174642	A	G	KIAA1109	exonic	nonsynonymous SNV	KIAA1109:NM_015312:exon3:c.A283G:p.M95V	.	1.398	7.34E-05	0.0003	5.58E-05	Fam105_f_m_aM	0/1;0/0;0/1
chr4	140379628	140379628	T	G	SCOC	exonic	nonsynonymous SNV	SCOC:NM_001153484:exon3:c.T313G:p.L105V,SCOC:NM_001153585:exon3:c.T202G:p.L68V,SCOC:NM_001153663:exon3:c.T310G:p.L104V,SCOC:NM_001153446:exon4:c.T202G:p.L68V,SCOC:NM_001153635:exon4:c.T199G:p.L67V,SCOC:NM_032547:exon4:c.T202G:p.L68V	.	1.223	.	3.30E-05	.	Fam61_f_m_aM	0/1;0/0;0/1
chr4	140634131	140634131	C	T	TBC1D9	exonic	nonsynonymous SNV	TBC1D9:NM_015130:exon16:c.G2563A:p.D855N	.	1.377	.	.	.	Fam64_f_m_aM	0/1;0/0;0/1
chr4	140911345	140911345	C	T	RNF150	exonic	nonsynonymous SNV	RNF150:NM_020724:exon6:c.G997A:p.D333N	.	1.47	0.0003	0.0001	8.38E-05	Fam49_f_m_aM	0/1;0/0;0/1
chr4	145765711	145765711	G	A	ZNF827	exonic	nonsynonymous SNV	ZNF827:NM_001306215:exon12:c.C2888T:p.S963L,ZNF827:NM_178835:exon12:c.C2888T:p.S963L	.	1.216	0.0006	0.0005	0.0002	Fam43_f_m_aM	0/1;0/0;0/1
chr4	146640437	146640437	A	C	POU4F2	exonic	nonsynonymous SNV	POU4F2:NM_004575:exon2:c.A859C:p.K287Q	.	1.46	.	0.0001	2.79E-05	Fam14_f_m_aM_aM	0/1;0/0;0/1;0/0
chr4	147532698	147532698	C	A	EDNRA	exonic	nonsynonymous SNV	EDNRA:NM_001957:exon4:c.C741A:p.F247L	.	2.116	.	.	.	Fam15_f_m_aM_aM	0/0;0/1;0/1;0/1
chr4	157359945	157359945	C	T	GRIA2	exonic	nonsynonymous SNV	GRIA2:NM_000826:exon13:c.C2093T:p.A698V,GRIA2:NM_001083619:exon13:c.C2093T:p.A698V,GRIA2:NM_001083620:exon13:c.C1952T:p.A651V	.	2.565	7.35E-05	0.0001	3.49E-05	Fam7_f_m_aM_aM_uM	0/1;0/0;0/1;0/1;0/0
chr4	163585848	163585848	C	G	MARCHF1	exonic	nonsynonymous SNV	MARCHF1:NM_017923:exon2:c.G273C:p.Q91H,MARCHF1:NM_001166373:exon6:c.G324C:p.Q108H	.	1.149	.	9.84E-05	1.40E-05	Fam18_f_m_aM_uF	0/1;0/0;0/1;0/1
chr4	169106897	169106897	A	C	SH3RF1	exonic	nonsynonymous SNV	SH3RF1:NM_020870:exon11:c.T2448G:p.C816W	.	1.279	.	.	7.00E-06	Fam10_f_m_aM_uF	0/0;0/0;0/1;0/0
chr4	170061947	170061947	G	T	AADAT	exonic	nonsynonymous SNV	AADAT:NM_016228:exon12:c.C1181A:p.P394H,AADAT:NM_001286682:exon13:c.C1193A:p.P398H,AADAT:NM_001286683:exon13:c.C1181A:p.P394H,AADAT:NM_182662:exon13:c.C1181A:p.P394H	.	1.068	.	.	.	Fam28_f_m_aF_uF	0/1;0/0;0/1;0/0
chr4	176327163	176327163	C	T	SPCS3	exonic	nonsynonymous SNV	SPCS3:NM_021928:exon4:c.C296T:p.A99V	.	1.181	.	9.68E-05	6.98E-06	Fam85_f_m_aM_aM	0/0;0/1;0/0;0/1
chr4	182796711	182796711	G	T	TENM3	exonic	nonsynonymous SNV	TENM3:NM_001080477:exon27:c.G7288T:p.D2430Y	.	1.946	.	9.08E-05	4.89E-05	Fam12_f_m_aM_uM_aM	0/1;0/0;0/1;0/0;0/0
chr4	183510632	183510632	G	C	ING2	exonic	nonsynonymous SNV	ING2:NM_001291959:exon2:c.G403C:p.D135H,ING2:NM_001564:exon2:c.G523C:p.D175H	.	1.061	.	.	.	Fam92_f_m_aM_raM	0/0;0/1;0/1;0/0
chr5	225527	225527	T	C	SDHA	exonic	nonsynonymous SNV	SDHA:NM_001330758:exon4:c.T421C:p.Y141H,SDHA:NM_004168:exon4:c.T421C:p.Y141H	.	1.317	.	.	.	Fam116_f_m_aM_aF	0/0;0/1;0/1;0/1
chr5	483327	483327	A	T	SLC9A3	exonic	nonsynonymous SNV	SLC9A3:NM_001284351:exon6:c.T1088A:p.I363N,SLC9A3:NM_004174:exon6:c.T1088A:p.I363N	.	2.464	.	.	.	Fam105_f_m_aM	0/0;0/1;0/1
chr5	485174	485174	C	T	SLC9A3	exonic	nonsynonymous SNV	SLC9A3:NM_001284351:exon4:c.G733A:p.V245M,SLC9A3:NM_004174:exon4:c.G733A:p.V245M	.	1.736	.	0.0002	4.19E-05	Fam91_m_aM_dM_aM_dM	0/1;0/1;0/1;0/0;0/1
chr5	491988	491988	C	T	SLC9A3	exonic	nonsynonymous SNV	SLC9A3:NM_001284351:exon2:c.G295A:p.V99I,SLC9A3:NM_004174:exon2:c.G295A:p.V99I	.	1.617	.	0.0001	9.21E-05	Fam51_f_m_aM_uF	0/0;0/1;0/1;0/1

chr5	1330378	1330378	A	G	CLPTM1L	exonic	nonsynonymous SNV	CLPTM1L:NM_030782:exon9:c.T982C:p.W328R	.	1.376	.	.	.	Fam83_f_m_aF	0/0;0/1;0/1
chr5	1411287	1411287	C	T	SLC6A3	exonic	nonsynonymous SNV	SLC6A3:NM_001044:exon9:c.G1225A:p.V409M	.	2.167	0.0003	.	2.09E-05	Fam107_f_m_aM	0/1;0/0;0/1
chr5	1463784	1463784	A	G	LPCAT1	exonic	nonsynonymous SNV	LPCAT1:NM_024830:exon14:c.T1472C:p.L491P	.	1.736	7.35E-05	.	6.98E-06	Fam25_f_m_aM	0/1;0/0;0/1
chr5	6616810	6616810	C	A	NSUN2	exonic	nonsynonymous SNV	NSUN2:NM_001193455:exon8:c.G833T:p.G278V,NSUN2:NM_017755:exon9:c.G938T:p.G313V	.	1.987	.	.	.	Fam23_f_m_aM_dF_uFs	0/1;0/0;0/1;0/1;0/0
chr5	6620158	6620158	C	T	NSUN2	exonic	nonsynonymous SNV	NSUN2:NM_001193455:exon6:c.G658A:p.G220S,NSUN2:NM_017755:exon7:c.G763A:p.G255S	.	1.487	.	0.0001	2.79E-05	Fam107_f_m_aM	0/1;0/0;0/1
chr5	9224866	9224866	G	A	SEMA5A	exonic	nonsynonymous SNV	SEMA5A:NM_003966:exon8:c.C454T:p.H152Y	.	1.115	7.35E-05	0.0001	3.49E-05	Fam20_f_m_aM_uF	0/0;0/1;0/1;0/0
chr5	11385187	11385187	C	T	CTNND2	exonic	nonsynonymous SNV	CTNND2:NM_001288715:exon6:c.G382A:p.A128T,CTNND2:NM_001332:exon7:c.G655A:p.A219T	.	1.659	0.0002	.	0.0002	Fam69_f_m_aM	0/0;0/1;0/1
chr5	11385187	11385187	C	T	CTNND2	exonic	nonsynonymous SNV	CTNND2:NM_001288715:exon6:c.G382A:p.A128T,CTNND2:NM_001332:exon7:c.G655A:p.A219T	.	1.659	0.0002	.	0.0002	Fam79_f_m_aM_uM	0/0;0/1;0/1;0/1
chr5	14769017	14769017	C	T	ANKH	exonic	nonsynonymous SNV	ANKH:NM_054027:exon2:c.G271A:p.V91M	.	1.521	.	1.12E-05	.	Fam52_f_m_aM	0/1;0/0;0/1
chr5	24511455	24511455	T	C	CDH10	exonic	nonsynonymous SNV	CDH10:NM_001317224:exon6:c.A874G:p.K292E,CDH10:NM_001362460:exon6:c.A874G:p.K292E,CDH10:NM_006727:exon6:c.A874G:p.K292E	.	1.569	.	0.0004	6.29E-05	Fam115_f_m_aF_aM_aF	0/1;0/0;0/1;0/1;0/1
chr5	32229920	32229920	C	T	MTMR12	exonic	nonsynonymous SNV	MTMR12:NM_001294344:exon14:c.G1772A:p.R591H,MTMR12:NM_001294343:exon15:c.G1940A:p.R647H,MTMR12:NM_001040446:exon16:c.G2102A:p.R701H	.	1.531	.	0.0007	4.19E-05	Fam53_f_m_aM	0/1;0/0;0/1
chr5	36181858	36181858	T	C	SKP2	exonic	nonsynonymous SNV	SKP2:NM_001243120:exon8:c.T460C:p.F154L,SKP2:NM_005983:exon10:c.T1102C:p.F368L	.	1.506	.	.	.	Fam38_f_m_aM	0/0;0/1;0/1
chr5	36241774	36241774	G	T	NADK2	exonic	nonsynonymous SNV	NADK2:NM_001085411:exon1:c.C25A:p.L9M	.	2.746	.	.	.	Fam61_f_m_aM	0/0;0/1;0/1
chr5	36671074	36671074	G	A	SLC1A3	exonic	nonsynonymous SNV	SLC1A3:NM_001289939:exon3:c.G227A:p.R76Q,SLC1A3:NM_001166695:exon4:c.G365A:p.R122Q,SLC1A3:NM_001289940:exon4:c.G365A:p.R122Q,SLC1A3:NM_004172:exon4:c.G365A:p.R122Q	.	1.341	.	6.54E-05	6.98E-06	Fam38_f_m_aM	0/0;0/1;0/1
chr5	36679791	36679791	G	A	SLC1A3	exonic	nonsynonymous SNV	SLC1A3:NM_001289940:exon5:c.G689A:p.R230Q,SLC1A3:NM_001289939:exon6:c.G887A:p.R296Q,SLC1A3:NM_001166695:exon7:c.G1025A:p.R342Q,SLC1A3:NM_004172:exon7:c.G1025A:p.R342Q	.	1.203	.	9.83E-05	1.40E-05	Fam109_f_m_aM	0/0;0/1;0/1
chr5	37024700	37024700	A	G	NIPBL	exonic	nonsynonymous SNV	NIPBL:NM_015384:exon30:c.A5690G:p.N1897S,NIPBL:NM_133433:exon30:c.A5690G:p.N1897S	.	1.826	0.0003	0.0001	6.98E-05	Fam66_f_m_aM	0/0;0/1;0/1
chr5	40762833	40762833	T	C	PRKAA1	exonic	nonsynonymous SNV	PRKAA1:NM_001355029:exon8:c.A1193G:p.H398R,PRKAA1:NM_001355035:exon8:c.A851G:p.H284R,PRKAA1:NM_001355036:exon8:c.A851G:p.H284R,PRKAA1:NM_001355037:exon9:c.A851G:p.H284R,PRKAA1:NM_006251:exon9:c.A1625G:p.H542R,PRKAA1:NM_001355028:exon10:c.A1547G:p.H516R,PRKAA1:NM_206907:exon10:c.A1670G:p.H557R	.	1.513	.	.	.	Fam49_f_m_aM	0/1;0/0;0/1
chr5	40777488	40777488	T	C	PRKAA1	exonic	nonsynonymous SNV	PRKAA1:NM_001355034:exon2:c.A226G:p.I76V,PRKAA1:NM_006251:exon2:c.A226G:p.I76V,PRKAA1:NM_206907:exon2:c.A226G:p.I76V,PRKAA1:NM_001355028:exon3:c.A148G:p.I50V	.	1.496	.	3.29E-05	.	Fam59_f_m_aF_uM	0/0;0/1;0/1;0/0
chr5	57247151	57247151	G	T	GPBP1	exonic	nonsynonymous SNV	GPBP1:NM_001127236:exon7:c.G761T:p.G254V,GPBP1:NM_001203246:exon7:c.G227T:p.G76V,GPBP1:NM_001127235:exon8:c.G761T:p.G254V,GPBP1:NM_022913:exon8:c.G740T:p.G247V,GPBP1:NM_001331037:exon9:c.G800T:p.G267V	.	1.117	0.0003	0.0004	0.0003	Fam79_f_m_aM_uM	0/1;0/0;0/1;0/1
chr5	58458527	58458527	G	A	PLK2	exonic	nonsynonymous SNV	PLK2:NM_006622:exon4:c.C497T:p.S166L,PLK2:NM_001252226:exon5:c.C455T:p.S152L	.	2.757	.	.	.	Fam28_f_m_aF_uF	0/0;0/1;0/1;0/1
chr5	62381242	62381242	C	G	KIF2A	exonic	nonsynonymous SNV	KIF2A:NM_001243953:exon19:c.C1967G:p.T656S,KIF2A:NM_004520:exon19:c.C2024G:p.T675S,KIF2A:NM_001098511:exon20:c.C2138G:p.T713S,KIF2A:NM_001243952:exon20:c.C1964G:p.T655S	.	1.097	.	7.46E-05	2.09E-05	Fam12_f_m_aM_uM_aM	0/0;0/1;0/1;0/1;0/1
chr5	65224380	65224380	T	C	ADAMTS6	exonic	nonsynonymous SNV	ADAMTS6:NM_197941:exon18:c.A2212G:p.I738V	.	1.251	.	.	.	Fam38_f_m_aM	0/1;0/0;0/1
chr5	65664576	65664576	T	G	TRAPPC13	exonic	nonsynonymous SNV	TRAPPC13:NM_001365342:exon11:c.T733G:p.C245G,TRAPPC13:NM_001365343:exon11:c.T730G:p.C244G,TRAPPC13:NM_001093756:exon12:c.T1201G:p.C401G,TRAPPC13:NM_001243737:exon12:c.T1204G:p.C402G,TRAPPC13:NM_001093755:exon13:c.T1222G:p.C408G,TRAPPC13:NM_024941:exon13:c.T1219G:p.C407G	.	1.085	.	3.36E-05	2.09E-05	Fam6_f_m_aM	0/1;0/0;0/1
chr5	66159305	66159305	C	T	SREK1	exonic	nonsynonymous SNV	SREK1:NM_001077199:exon3:c.C382T:p.P128S,SREK1:NM_001323529:exon3:c.C382T:p.P128S,SREK1:NM_001323533:exon3:c.C382T:p.P128S,SREK1:NM_001270492:exon4:c.C34T:p.P12S,SREK1:NM_001323527:exon4:c.C34T:p.P12S,SREK1:NM_139168:exon4:c.C34T:p.P12S	.	2.377	.	.	.	Fam118_f_m_aM_aF_uM	0/0;0/1;0/0;0/1;0/1
chr5	69100868	69100868	G	C	SLC30A5	exonic	nonsynonymous SNV	SLC30A5:NM_022902:exon2:c.G145C:p.E49Q,SLC30A5:NM_024055:exon2:c.G145C:p.E49Q	.	1.152	.	2.24E-05	1.41E-05	Fam91_m_aM_dM_aM_dM	0/1;0/1;0/1;0/1;0/0
chr5	76955726	76955726	G	A	CRHBP	exonic	nonsynonymous SNV	CRHBP:NM_001882:exon4:c.G407A:p.R136Q	.	1.587	.	3.35E-05	6.98E-06	Fam45_f_m_aM_uF	0/1;0/0;0/1;0/1
chr5	78510179	78510179	A	G	LHFPL2	exonic	nonsynonymous SNV	LHFPL2:NM_005779:exon4:c.T35C:p.L12P	.	1.665	.	.	.	Fam120_f_m_aM_aM	0/0;0/1;0/1;0/0
chr5	81446921	81446921	C	T	SSBP2	exonic	nonsynonymous SNV	SSBP2:NM_001256733:exon11:c.G665A:p.G222D,SSBP2:NM_001256734:exon11:c.G659A:p.G220D,SSBP2:NM_001256735:exon11:c.G635A:p.G212D,SSBP2:NM_001256736:exon11:c.G635A:p.G212D,SSBP2:NM_001256732:exon12:c.G749A:p.G250D,SSBP2:NM_001345886:exon12:c.G749A:p.G250D,SSBP2:NM_012446:exon12:c.G725A:p.G242D	.	2.212	.	.	.	Fam34_f_m_aM_uF	0/1;0/0;0/1;0/0
chr5	95736954	95736954	C	G	RHOBTB3	exonic	nonsynonymous SNV	RHOBTB3:NM_014899:exon3:c.C294G:p.I98M	.	1.24	.	.	.	Fam101_f_m_aM	0/1;0/0;0/1

chr5	100562140	100562140	A	T	FAM174A	exonic	nonsynonymous SNV	FAM174A:NM_198507:exon2:c.A521T:p.D174V	.	1.779	.	.	.		Fam39_f_m_aM	0/0;0/1;0/1
chr5	114404745	114404745	A	G	KCNN2	exonic	nonsynonymous SNV	KCNN2:NM_021614:exon3:c.A1526G:p.N509S	.	2.068	.	.	.		Fam56_f_m_aF_aM	0/0;0/1;0/1;0/0
chr5	129461444	129461444	C	T	ADAMTS19	exonic	nonsynonymous SNV	ADAMTS19:NM_133638:exon2:c.C434T:p.S145L	.	2.142	7.38E-05	0.0004	6.29E-05		Fam117_f_m_aM_aF	0/1;0/0;0/1;0/1
chr5	131672678	131672678	T	C	FNIP1	exonic	nonsynonymous SNV	FNIP1:NM_001008738:exon13:c.A1682G:p.H561R,FNIP1:NM_001346114:exon13:c.A1631G:p.H544R,FNIP1:NM_133372:exon14:c.A1766G:p.H589R	.	1.404	.	0.0001	4.19E-05		Fam24_f_m_aM_aM	0/1;0/0;0/0;0/1
chr5	131796838	131796838	G	C	FNIP1	exonic	nonsynonymous SNV	FNIP1:NM_001008738:exon1:c.C84G:p.C28W,FNIP1:NM_001346113:exon1:c.C84G:p.C28W,FNIP1:NM_001346114:exon1:c.C84G:p.C28W,FNIP1:NM_133372:exon1:c.C84G:p.C28W	.	1.351	.	1.45E-05	.		Fam67_f_m_aM_uF	0/1;0/0;0/1;0/0
chr5	131989436	131989436	T	A	ACSL6	exonic	nonsynonymous SNV	ACSL6:NM_001009185:exon5:c.A523T:p.I175F,ACSL6:NM_001205247:exon5:c.A418T:p.I140F,ACSL6:NM_001205248:exon5:c.A448T:p.I150F,ACSL6:NM_001205250:exon5:c.A481T:p.I161F,ACSL6:NM_001205251:exon5:c.A343T:p.I115F,ACSL6:NM_015256:exon5:c.A523T:p.I175F	.	1.221	.	0.0005	2.80E-05		Fam113_f_m_aF_raM_uF	0/1;0/0;0/1;0/0;0/0
chr5	132073876	132073876	C	T	CSF2	exonic	nonsynonymous SNV	CSF2:NM_000758:exon1:c.C53T:p.A18V	.	1.051	.	3.39E-05	.		Fam114_f_m_aM	0/1;0/0;0/1
chr5	132747856	132747856	C	G	CCNI2	exonic	nonsynonymous SNV	CCNI2:NM_001039780:exon1:c.C361G:p.R121G,CCNI2:NM_001287252:exon1:c.C361G:p.R121G,CCNI2:NM_001287253:exon1:c.C361G:p.R121G	.	2.479	.	.	.		Fam25_f_m_aM	0/1;0/0;0/1
chr5	138886218	138886218	C	T	CTNNA1	exonic	nonsynonymous SNV	CTNNA1:NM_001290309:exon7:c.C760T:p.R254C,CTNNA1:NM_001290307:exon8:c.C1069T:p.R357C,CTNNA1:NM_001290310:exon8:c.C700T:p.R234C,CTNNA1:NM_001323983:exon8:c.C1069T:p.R357C,CTNNA1:NM_001323985:exon8:c.C1069T:p.R357C,CTNNA1:NM_001323986:exon8:c.C1069T:p.R357C,CTNNA1:NM_001903:exon8:c.C1069T:p.R357C,CTNNA1:NM_001323982:exon9:c.C1069T:p.R357C,CTNNA1:NM_001323984:exon9:c.C1069T:p.R357C	.	1.313	.	7.50E-05	6.99E-06		Fam65_f_m_aM_uF_dF	0/1;0/0;0/1;0/1;0/1
chr5	138932710	138932710	G	A	CTNNA1	exonic	nonsynonymous SNV	CTNNA1:NM_001324010:exon8:c.G982A:p.G328R,CTNNA1:NM_001324012:exon9:c.G1078A:p.G360R,CTNNA1:NM_001290312:exon11:c.G1321A:p.G441R,CTNNA1:NM_001323987:exon11:c.G1321A:p.G441R,CTNNA1:NM_001323994:exon11:c.G1321A:p.G441R,CTNNA1:NM_001324002:exon11:c.G1321A:p.G441R,CTNNA1:NM_001324004:exon11:c.G1321A:p.G441R,CTNNA1:NM_001323989:exon12:c.G1321A:p.G441R,CTNNA1:NM_001323991:exon12:c.G1321A:p.G441R,CTNNA1:NM_001324000:exon12:c.G1321A:p.G441R,CTNNA1:NM_001324008:exon12:c.G982A:p.G328R,CTNNA1:NM_001324009:exon12:c.G982A:p.G328R,CTNNA1:NM_001324013:exon12:c.G1078A:p.G360R,CTNNA1:NM_001323990:exon13:c.G1321A:p.G441R,CTNNA1:NM_001324003:exon13:c.G1321A:p.G441R,CTNNA1:NM_001324011:exon13:c.G1228A:p.G410R,CTNNA1:NM_001323988:exon14:c.G1321A:p.G441R,CTNNA1:NM_001323992:exon14:c.G1321A:p.G441R,CTNNA1:NM_001323993:exon14:c.G1321A:p.G441R,CTNNA1:NM_001323995:exon14:c.G1321A:p.G441R,CTNNA1:NM_001323996:exon14:c.G1321A:p.G441R,CTNNA1:NM_001323997:exon14:c.G1321A:p.G441R,CTNNA1:NM_001323998:exon14:c.G1321A:p.G441R,CTNNA1:NM_001323999:exon14:c.G1321A:p.G441R,CTNNA1:NM_001324005:exon14:c.G1321A:p.G441R,CTNNA1:NM_001324007:exon14:c.G982A:p.G328R,CTNNA1:NM_001324006:exon15:c.G982A:p.G328R,CTNNA1:NM_001290309:exon16:c.G2122A:p.G708R,CTNNA1:NM_001323986:exon16:c.G2338A:p.G780R,CTNNA1:NM_001290307:exon17:c.G2431A:p.G811R,CTNNA1:NM_001290310:exon17:c.G2062A:p.G688R,CTNNA1:NM_001323983:exon17:c.G2431A:p.G811R,CTNNA1:NM_001323985:exon17:c.G2431A:p.G811R,CTNNA1:NM_001903:exon17:c.G2431A:p.G811R,CTNNA1:NM_001323982:exon18:c.G2431A:p.G811R,CTNNA1:NM_001323984:exon18:c.G2431A:p.G811R	.	2.3	.	1.12E-05	.		Fam28_f_m_aF_uF	0/1;0/0;0/1;0/0
chr5	139379259	139379259	C	T	SLC23A1	exonic	nonsynonymous SNV	SLC23A1:NM_005847:exon9:c.G1021A:p.A341T,SLC23A1:NM_152685:exon9:c.G1033A:p.A345T	.	1.325	.	4.47E-05	3.49E-05		Fam54_f_m_aM_uF	0/0;0/1;0/1;0/0
chr5	139680747	139680747	G	A	CXXC5	exonic	nonsynonymous SNV	CXXC5:NM_001317200:exon2:c.G224A:p.R75H,CXXC5:NM_001317201:exon2:c.G224A:p.R75H,CXXC5:NM_001317202:exon2:c.G224A:p.R75H,CXXC5:NM_001317205:exon2:c.G224A:p.R75H,CXXC5:NM_001317207:exon2:c.G224A:p.R75H,CXXC5:NM_001317209:exon2:c.G224A:p.R75H,CXXC5:NM_016463:exon2:c.G224A:p.R75H,CXXC5:NM_001317199:exon3:c.G224A:p.R75H,CXXC5:NM_001317203:exon3:c.G224A:p.R75H,CXXC5:NM_001317204:exon3:c.G224A:p.R75H,CXXC5:NM_001317208:exon3:c.G224A:p.R75H,CXXC5:NM_001317210:exon3:c.G224A:p.R75H,CXXC5:NM_001317211:exon3:c.G224A:p.R75H,CXXC5:NM_001317206:exon4:c.G224A:p.R75H	.	1.13	0.0006	7.70E-05	1.40E-05		Fam63_f_m_aF	0/0;0/1;0/1
chr5	139814307	139814307	G	A	PSD2	exonic	nonsynonymous SNV	PSD2:NM_032289:exon4:c.G959A:p.R320H	.	1.544	7.35E-05	0.0002	4.19E-05		Fam20_f_m_aM_uF	0/1;0/0;0/1;0/0

chr5	139814348	139814348	C	T	PSD2	exonic	nonsynonymous SNV	PSD2:NM_032289:exon4:c.C1000T:p.R334W	.	1.404	.	0.0002	2.09E-05	Fam21_f_m_aM_uM	0/1;0/0;0/1;0/1
chr5	139871826	139871826	G	A	NRG2	exonic	nonsynonymous SNV	NRG2:NM_004883:exon4:c.C1007T:p.S336L,NRG2:NM_013981:exon4:c.C1007T:p.S336L,NRG2:NM_013982:exon4:c.C1007T:p.S336L,NRG2:NM_013983:exon4:c.C1007T:p.S336L	.	1.538	7.34E-05	.	2.09E-05	Fam77_f_m_aM_aF_uM_uFs	0/0;0/1;0/1;0/0;0/1;0/0
chr5	140335956	140335956	T	C	HBEGF	exonic	nonsynonymous SNV	HBEGF:NM_001945:exon4:c.A470G:p.Y157C	.	2.185	.	.	6.98E-06	Fam61_f_m_aM	0/0;0/1;0/1
chr5	140529737	140529737	A	G	ANKHD1;ANKHD1	exonic	nonsynonymous SNV	ANKHD1:NM_017747:exon29:c.A6791G:p.D2264G,ANKHD1-EIF4EBP3:NM_020690:exon29:c.A6791G:p.D2264G	.	1.103	.	4.58E-05	6.98E-06	Fam103_f_m_aM	0/0;0/1;0/1
chr5	140562100	140562100	C	T	APBB3	exonic	nonsynonymous SNV	APBB3:NM_006051:exon6:c.G626A:p.R209H,APBB3:NM_133172:exon6:c.G626A:p.R209H,APBB3:NM_133173:exon6:c.G626A:p.R209H,APBB3:NM_133174:exon6:c.G626A:p.R209Q	.	1.015	.	6.71E-05	6.98E-06	Fam43_f_m_aM	0/1;0/0;0/1
chr5	140659793	140659793	T	G	IK	exonic	nonsynonymous SNV	IK:NM_006083:exon14:c.T1233G:p.N411K	.	1.254	.	.	.	Fam122_f_m_aM	0/0;0/1;0/1
chr5	140674744	140674744	T	G	HARS	exonic	nonsynonymous SNV	HARS:NM_001289093:exon9:c.A1051C:p.I351L,HARS:NM_001289092:exon10:c.A1171C:p.I391L,HARS:NM_001258040:exon11:c.A1273C:p.I425L,HARS:NM_001258042:exon11:c.A1213C:p.I405L,HARS:NM_001258041:exon12:c.A1333C:p.I445L,HARS:NM_001289094:exon12:c.A1306C:p.I436L,HARS:NM_002109:exon12:c.A1393C:p.I465L	.	1.111	.	2.23E-05	.	Fam113_f_m_aF_raM_uF	0/0;0/0;0/0;0/1;0/0
chr5	140786721	140786721	A	C	PCDHA1	exonic	nonsynonymous SNV	PCDHA1:NM_018900:exon1:c.A431C:p.E144A,PCDHA1:NM_031410:exon1:c.A431C:p.E144A,PCDHA1:NM_031411:exon1:c.A431C:p.E144A	.	1.037	7.34E-05	0.0002	6.28E-05	Fam103_f_m_aM	0/0;0/1;0/1
chr5	141051843	141051843	G	T	PCDHB1	exonic	nonsynonymous SNV	PCDHB1:NM_013340:exon1:c.G373T:p.D125Y	.	1.315	0.0004	0.0003	0.0002	Fam120_f_m_aM_aM	0/0;0/1;0/1;0/0
chr5	141340204	141340204	C	A	PCDHGA2	exonic	nonsynonymous SNV	PCDHGA2:NM_018915:exon1:c.C1233A:p.D411E,PCDHGA2:NM_032009:exon1:c.C1233A:p.D411E	.	1.012	.	3.35E-05	6.98E-06	Fam7_f_m_aM_aM_uM	0/1;0/0;0/1;0/1;0/0
chr5	141340331	141340331	C	T	PCDHGA2	exonic	nonsynonymous SNV	PCDHGA2:NM_018915:exon1:c.C1360T:p.R454C,PCDHGA2:NM_032009:exon1:c.C1360T:p.R454C	.	1.125	0.0003	0.0005	0.0002	Fam108_f_m_aM	0/0;0/1;0/1
chr5	141340436	141340436	T	C	PCDHGA2	exonic	nonsynonymous SNV	PCDHGA2:NM_018915:exon1:c.T1465C:p.Y489H,PCDHGA2:NM_032009:exon1:c.T1465C:p.Y489H	.	1.2	.	0.0002	4.19E-05	Fam110_f_m_aM_aM_uMs	0/1;0/0;0/1;0/0;0/1
chr5	141344704	141344704	G	A	PCDHGA3	exonic	nonsynonymous SNV	PCDHGA3:NM_018916:exon1:c.G671A:p.G224D,PCDHGA3:NM_032011:exon1:c.G671A:p.G224D	.	1.409	.	2.25E-05	1.40E-05	Fam90_f_m_aF_dM	0/0;0/1;0/1;0/0
chr5	141351151	141351151	C	G	PCDHGB1	exonic	nonsynonymous SNV	PCDHGB1:NM_018922:exon1:c.C891G:p.I297M,PCDHGB1:NM_032095:exon1:c.C891G:p.I297M	.	1.17	.	.	.	Fam20_f_m_aM_uF	0/0;0/1;0/1;0/1
chr5	141352121	141352121	G	A	PCDHGB1	exonic	nonsynonymous SNV	PCDHGB1:NM_018922:exon1:c.G1861A:p.E621K,PCDHGB1:NM_032095:exon1:c.G1861A:p.E621K	.	1.245	.	.	.	Fam43_f_m_aM	0/1;0/0;0/1
chr5	141375414	141375414	A	G	PCDHGA6	exonic	nonsynonymous SNV	PCDHGA6:NM_018919:exon1:c.A1331G:p.D444G,PCDHGA6:NM_032086:exon1:c.A1331G:p.D444G	.	1.123	.	0.0002	6.98E-06	Fam116_f_m_aM_aF	0/0;0/1;0/0;0/1
chr5	141408488	141408488	T	G	PCDHGB6	exonic	nonsynonymous SNV	PCDHGB6:NM_018926:exon1:c.T286G:p.C96G,PCDHGB6:NM_032100:exon1:c.T286G:p.C96G	.	1.391	0.0003	6.57E-05	6.28E-05	Fam92_f_m_aM_raM	0/0;0/1;0/1;0/0
chr5	141420006	141420006	G	A	PCDHGB7	exonic	nonsynonymous SNV	PCDHGB7:NM_018927:exon1:c.G2147A:p.R716Q,PCDHGB7:NM_032101:exon1:c.G2147A:p.R716Q	.	1.089	.	.	.	Fam24_f_m_aM_aM	0/0;0/1;0/0;0/1
chr5	141423002	141423002	T	C	PCDHGA11	exonic	nonsynonymous SNV	PCDHGA11:NM_018914:exon1:c.T1775C:p.V592A,PCDHGA11:NM_032091:exon1:c.T1775C:p.V592A,PCDHGA11:NM_032092:exon1:c.T1775C:p.V592A	.	1.317	.	.	.	Fam97_f_m_aM_aF	0/0;0/1;0/1;0/1
chr5	141476387	141476387	C	G	PCDHGC3	exonic	nonsynonymous SNV	PCDHGC3:NM_002588:exon1:c.C271G:p.R91G,PCDHGC3:NM_032402:exon1:c.C271G:p.R91G	.	1.71	0.0004	0.0006	0.0003	Fam32_f_m_aM_uM	0/0;0/1;0/1;0/1
chr5	141478175	141478175	A	-	PCDHGC3	exonic	frameshift deletion	PCDHGC3:NM_002588:exon1:c.2059delA:p.N689Ifs*10,PCDHGC3:NM_032402:exon1:c.2059delA:p.N689Ifs*10	0.577	.	0.0006	.	6.99E-06	Fam24_f_m_aM_aM	0/1;0/0;0/0;0/1
chr5	141486792	141486792	G	A	PCDHGC4	exonic	nonsynonymous SNV	PCDHGC4:NM_018928:exon1:c.G1619A:p.R540Q,PCDHGC4:NM_032406:exon1:c.G1619A:p.R540Q	.	1.131	.	4.47E-05	1.40E-05	Fam49_f_m_aM	0/0;0/1;0/1
chr5	141490787	141490787	T	A	PCDHGC5	exonic	nonsynonymous SNV	PCDHGC5:NM_018929:exon1:c.T1547A:p.I516N,PCDHGC5:NM_032407:exon1:c.T1547A:p.I516N	.	1.12	.	.	.	Fam72_f_m_aF_uF	0/0;0/1;0/1;0/1
chr5	141505413	141505413	C	T	PCDHGA1;PCDHG	exonic	nonsynonymous SNV	PCDHGC3:NM_002588:exon3:c.C2510T:p.T837I,PCDHGA12:NM_003735:exon3:c.C2504T:p.T835I,PCDHGB4:NM_003736:exon3:c.C2477T:p.T826I,PCDHGA1:NM_018912:exon3:c.C2501T:p.T834I,PCDHGA10:NM_018913:exon3:c.C2516T:p.T839I,PCDHGA11:NM_018914:exon3:c.C2513T:p.T838I,PCDHGA2:NM_018915:exon3:c.C2504T:p.T835I,PCDHGA3:NM_018916:exon3:c.C2504T:p.T835I,PCDHGA4:NM_018917:exon3:c.C2594T:p.T865I,PCDHGA5:NM_018918:exon3:c.C2501T:p.T834I,PCDHGA6:NM_018919:exon3:c.C2504T:p.T835I,PCDHGA7:NM_018920:exon3:c.C2504T:p.T835I,PCDHGA9:NM_018921:exon3:c.C2504T:p.T835I,PCDHGB1:NM_018922:exon3:c.C2489T:p.T830I,PCDHGB2:NM_018923:exon3:c.C2501T:p.T834I,PCDHGB3:NM_018924:exon3:c.C2495T:p.T832I,PCDHGB5:NM_018925:exon3:c.C2477T:p.T826I,PCDHGB6:NM_018926:exon3:c.C2498T:p.T833I,PCDHGB7:NM_018927:exon3:c.C2495T:p.T832I,PCDHGC4:NM_018928:exon3:c.C2522T:p.T841I,PCDHGC5:NM_018929:exon3:c.C2540T:p.T847I,PCDHGA8:NM_032088:exon3:c.C2504T:p.T835I,PCDHGA11:NM_032092:exon3:c.C1958T:p.T653I,PCDHGC3:NM_032403:exon3:c.C110T:p.T37I	.	1.348	.	1.14E-05	.	Fam119_f_m_aM_aM	0/0;0/1;0/1;0/1
chr5	141659912	141659912	G	A	ARAP3	exonic	nonsynonymous SNV	ARAP3:NM_022481:exon22:c.C3134T:p.A1045V	.	1.054	0.0005	0.0002	9.07E-05	Fam115_f_m_aF_aM_aF	0/0;0/1;0/1;0/0;0/0
chr5	141673783	141673783	G	A	ARAP3	exonic	nonsynonymous SNV	ARAP3:NM_022481:exon5:c.C724T:p.R242W	.	1.008	.	1.12E-05	6.98E-06	Fam101_f_m_aM	0/0;0/1;0/1

chr5	142314589	142314589	G	A	SPRY4	exonic	nonsynonymous SNV	SPRY4:NM_001127496:exon2:c.C520T:p.R174W,SPRY4:NM_001293290:exon2:c.C520T:p.R174W,SPRY4:NM_001293289:exon3:c.C520T:p.R174W,SPRY4:NM_030964:exon3:c.C589T:p.R197W	.	1.099	0.0003	9.83E-05	5.58E-05	Fam119_f_m_aM_aM	0/1;0/0;0/1;0/0
chr5	146340372	146340372	G	C	POU4F3	exonic	nonsynonymous SNV	POU4F3:NM_002700:exon2:c.G945C:p.K315N	.	1.416	0.0006	0.0002	6.98E-06	Fam5_f_m_aM	0/0;0/1;0/1
chr5	147401570	147401570	G	C	DPYSL3	exonic	nonsynonymous SNV	DPYSL3:NM_001197294:exon9:c.C1280G:p.T427S,DPYSL3:NM_001387:exon9:c.C938G:p.T313S	.	1.273	.	.	.	Fam119_f_m_aM_aM	0/1;0/0;0/1;0/1
chr5	150528020	150528020	C	T	NDST1	exonic	nonsynonymous SNV	NDST1:NM_001301063:exon3:c.C730T:p.R244C,NDST1:NM_001543:exon3:c.C730T:p.R244C	.	1.786	.	0.0002	3.49E-05	Fam108_f_m_aM	0/1;0/0;0/1
chr5	150535808	150535808	C	T	NDST1	exonic	nonsynonymous SNV	NDST1:NM_001301063:exon6:c.C1360T:p.R454C,NDST1:NM_001543:exon6:c.C1360T:p.R454C	.	1.713	0.0002	0.0003	0.0001	Fam23_f_m_aM_dF_uFs	0/0;0/1;0/1;0/1;0/0
chr5	150549774	150549774	C	A	NDST1	exonic	nonsynonymous SNV	NDST1:NM_001301063:exon12:c.C2242A:p.H748N,NDST1:NM_001543:exon13:c.C2413A:p.H805N	.	1.178	.	.	.	Fam96_f_m_aM_uF	0/0;0/1;0/1;0/1
chr5	150648441	150648441	-	C	SYNPO	exonic	frameshift insertion	SYNPO:NM_001109974:exon2:c.167dupC:p.P58Tfs*4,SYNPO:NM_007286:exon2:c.167dupC:p.P58Tfs*4,SYNPO:NM_001166208:exon3:c.899dupC:p.P302Tfs*4,SYNPO:NM_001166209:exon3:c.899dupC:p.P302Tfs*4	0.579	.	.	.	.	Fam65_f_m_aM_uF_dF	0/0;0/1;0/1;0/1;0/1
chr5	150696670	150696670	C	A	RBM22	exonic	nonsynonymous SNV	RBM22:NM_018047:exon6:c.G408T:p.M136I	.	1.263	.	.	.	Fam38_f_m_aM	0/0;0/1;0/1
chr5	151465147	151465147	C	T	SLC36A1	exonic	nonsynonymous SNV	SLC36A1:NM_001308150:exon5:c.C397T:p.R133W,SLC36A1:NM_001308151:exon5:c.C397T:p.R133W,SLC36A1:NM_078483:exon5:c.C397T:p.R133W,SLC36A1:NM_001349740:exon6:c.C313T:p.R105W	.	1.009	0.0003	0.0003	0.0001	Fam45_f_m_aM_uF	0/0;0/1;0/1;0/1
chr5	151666438	151666438	G	T	SPARC	exonic	nonsynonymous SNV	SPARC:NM_001309443:exon8:c.C654A:p.D218E,SPARC:NM_001309444:exon8:c.C657A:p.D219E,SPARC:NM_003118:exon8:c.C657A:p.D219E	.	1.088	0.0004	0.001	0.0006	Fam12_f_m_aM_uM_aM	0/1;0/0;0/0;0/0;0/1
chr5	153647083	153647083	C	T	GRIA1	exonic	nonsynonymous SNV	GRIA1:NM_000827:exon3:c.C376T:p.R126C,GRIA1:NM_001114183:exon3:c.C376T:p.R126C,GRIA1:NM_001258021:exon3:c.C406T:p.R136C,GRIA1:NM_001258022:exon3:c.C406T:p.R136C,GRIA1:NM_001258023:exon3:c.C169T:p.R57C,GRIA1:NM_001364165:exon3:c.C376T:p.R126C,GRIA1:NM_001364167:exon3:c.C169T:p.R57C,GRIA1:NM_001258020:exon4:c.C91T:p.R31C,GRIA1:NM_001364166:exon4:c.C403T:p.R135C	.	1.407	7.34E-05	2.23E-05	2.79E-05	Fam26_f_m_aM_uM	0/1;0/0;0/1;0/1
chr5	153698985	153698985	G	A	GRIA1	exonic	nonsynonymous SNV	GRIA1:NM_001258019:exon9:c.G1124A:p.R375H,GRIA1:NM_001364165:exon9:c.G1196A:p.R399H,GRIA1:NM_000827:exon10:c.G1364A:p.R455H,GRIA1:NM_001114183:exon10:c.G1364A:p.R455H,GRIA1:NM_001258021:exon10:c.G1394A:p.R465H,GRIA1:NM_001258022:exon10:c.G1394A:p.R465H,GRIA1:NM_001258023:exon10:c.G1157A:p.R386H,GRIA1:NM_001364167:exon10:c.G1157A:p.R386H,GRIA1:NM_001258020:exon11:c.G1079A:p.R360H,GRIA1:NM_001364166:exon11:c.G1391A:p.R464H	.	2.459	7.34E-05	6.17E-05	2.09E-05	Fam22_f_m_aF_aF_uF	0/1;0/0;0/1;0/1;0/1
chr5	154376427	154376427	G	C	GALNT10	exonic	nonsynonymous SNV	GALNT10:NM_198321:exon5:c.G719C:p.C240S	.	1.343	.	1.20E-05	1.40E-05	Fam34_f_m_aM_uF	0/0;0/1;0/1;0/0
chr5	154416814	154416814	G	A	GALNT10	exonic	nonsynonymous SNV	GALNT10:NM_198321:exon12:c.G1654A:p.D552N	.	1.058	.	.	.	Fam91_m_aM_dM_aM_dM	0/0;0/0;0/0;0/1;0/0
chr5	157240129	157240129	G	A	ITK	exonic	nonsynonymous SNV	ITK:NM_005546:exon10:c.G919A:p.V307M	.	1.018	.	.	.	Fam100_f_m_aF	0/0;0/1;0/1
chr5	157389363	157389363	C	T	CYFIP2	exonic	nonsynonymous SNV	CYFIP2:NM_001291721:exon28:c.C3304T:p.R1102W,CYFIP2:NM_001037333:exon29:c.C3382T:p.R1128W,CYFIP2:NM_014376:exon29:c.C3382T:p.R1128W,CYFIP2:NM_001291722:exon30:c.C3457T:p.R1153W	.	1.699	7.34E-05	5.63E-05	4.19E-05	Fam66_f_m_aM	0/1;0/0;0/1
chr5	159161528	159161528	T	G	RNF145	exonic	nonsynonymous SNV	RNF145:NM_001199380:exon10:c.A1454C:p.N485T,RNF145:NM_001199381:exon10:c.A1415C:p.N472T,RNF145:NM_001199382:exon10:c.A1406C:p.N469T,RNF145:NM_001199383:exon10:c.A1364C:p.N455T,RNF145:NM_144726:exon10:c.A1448C:p.N483T	.	2.528	7.35E-05	.	2.09E-05	Fam12_f_m_aM_uM_aM	0/1;0/0;0/0;0/0;0/1
chr5	160407974	160407974	T	C	SLU7	exonic	nonsynonymous SNV	SLU7:NM_001364517:exon9:c.A959G:p.D320G,SLU7:NM_001364518:exon9:c.A914G:p.D305G,SLU7:NM_001364522:exon9:c.A944G:p.D315G,SLU7:NM_006425:exon9:c.A914G:p.D305G,SLU7:NM_001364520:exon10:c.A914G:p.D305G,SLU7:NM_001364521:exon10:c.A914G:p.D305G,SLU7:NM_001364523:exon10:c.A944G:p.D315G	.	2	.	.	.	Fam92_f_m_aM_raM	0/0;0/0;0/0;0/1
chr5	163516577	163516577	G	C	MAT2B	exonic	nonsynonymous SNV	MAT2B:NM_013283:exon5:c.G586C:p.A196P,MAT2B:NM_182796:exon5:c.G553C:p.A185P	.	1.308	.	0.0004	0.0002	Fam43_f_m_aM	0/0;0/1;0/1
chr5	163518350	163518350	C	T	MAT2B	exonic	nonsynonymous SNV	MAT2B:NM_013283:exon7:c.C992T:p.T331M,MAT2B:NM_182796:exon7:c.C959T:p.T320M	.	1.164	7.49E-05	4.63E-05	5.61E-05	Fam122_f_m_aM	0/1;0/0;0/1
chr5	168246850	168246850	C	T	TENM2	exonic	nonsynonymous SNV	TENM2:NM_001080428:exon23:c.C5194T:p.R1732W,TENM2:NM_001368145:exon25:c.C5434T:p.R1812W,TENM2:NM_001368146:exon26:c.C5428T:p.R1810W,TENM2:NM_001122679:exon28:c.C5884T:p.R1962W	.	1.267	.	2.25E-05	.	Fam105_f_m_aM	0/0;0/1;0/1
chr5	168749502	168749502	C	G	SLIT3	exonic	nonsynonymous SNV	SLIT3:NM_001271946:exon19:c.G2107C:p.D703H,SLIT3:NM_003062:exon19:c.G2107C:p.D703H	.	1.063	.	.	6.98E-06	Fam116_f_m_aM_aF	0/0;0/1;0/0;0/1
chr5	172106725	172106725	A	G	STK10	exonic	nonsynonymous SNV	STK10:NM_005990:exon6:c.T683C:p.I228T	.	1.176	.	.	.	Fam56_f_m_aF_aM	0/0;0/1;0/1;0/1
chr5	172770592	172770592	G	C	DUSP1	exonic	nonsynonymous SNV	DUSP1:NM_004417:exon1:c.C361G:p.L121V	.	1.344	.	.	.	Fam85_f_m_aM_aM	0/1;0/0;0/1;0/0
chr5	173945099	173945099	G	A	CPEB4	exonic	nonsynonymous SNV	CPEB4:NM_001308191:exon3:c.G1340A:p.R447Q,CPEB4:NM_001308193:exon3:c.G194A:p.R65Q,CPEB4:NM_001308189:exon4:c.G1364A:p.R455Q,CPEB4:NM_001308192:exon4:c.G245A:p.R82Q,CPEB4:NM_030627:exon5:c.G1415A:p.R472Q	.	2.832	.	7.45E-05	.	Fam57_f_m_aM_uF	0/0;0/1;0/1;0/1
chr5	176368827	176368827	C	A	ARL10	exonic	nonsynonymous SNV	ARL10:NM_001317948:exon3:c.C406A:p.R136S,ARL10:NM_173664:exon3:c.C406A:p.R136S	.	1.423	.	9.01E-05	1.40E-05	Fam103_f_m_aM	0/1;0/0;0/1

chr5	177295172	177295173	TC	-	NSD1	exonic	frameshift deletion	NSD1:NM_022455:exon23:c.7804_7805del:p.L2603Rfs*10,NSD1:NM_001365684:exon24:c.6997_6998del:p.L2334Rfs*10,NSD1:NM_172349:exon24:c.6997_6998del:p.L2334Rfs*10	0.101	.	.	.	.	Fam5_f_m_aM	0/1;0/0;0/1
chr5	177351257	177351257	G	T	LMAN2	exonic	nonsynonymous SNV	LMAN2:NM_006816:exon2:c.C231A:p.F77L	.	1.176	.	.	.	Fam110_f_m_aM_aM_uMs	0/1;0/0;0/0;0/1;0/0
chr5	177400351	177400351	C	G	PFN3	exonic	nonsynonymous SNV	PFN3:NM_001029886:exon1:c.G226C:p.D76H	.	1.774	.	0.0006	6.98E-06	Fam87_f_m_aM_uM	0/1;0/0;0/1;0/1
chr5	177434915	177434915	G	A	GRK6	exonic	nonsynonymous SNV	GRK6:NM_001364164:exon9:c.G313A:p.E105K,GRK6:NM_001004105:exon10:c.G943A:p.E315K,GRK6:NM_001004106:exon10:c.G943A:p.E315K,GRK6:NM_002082:exon10:c.G943A:p.E315K	.	1.857	.	.	.	Fam103_f_m_aM	0/1;0/0;0/1
chr5	177436406	177436406	A	T	GRK6	exonic	nonsynonymous SNV	GRK6:NM_001364164:exon12:c.A650T:p.D217V,GRK6:NM_001004105:exon13:c.A1280T:p.D427V,GRK6:NM_001004106:exon13:c.A1280T:p.D427V,GRK6:NM_002082:exon13:c.A1280T:p.D427V	.	1.925	.	6.54E-05	.	Fam28_f_m_aF_uF	0/0;0/1;0/1;0/1
chr5	179273037	179273037	C	G	ADAMTS2	exonic	nonsynonymous SNV	ADAMTS2:NM_014244:exon3:c.G562C:p.E188Q,ADAMTS2:NM_021599:exon3:c.G562C:p.E188Q	.	1.23	7.35E-05	0.0002	0.0001	Fam80_f_m_aM_uM	0/1;0/0;0/1;0/0
chr5	179619344	179619344	-	C	HNRNPH1	exonic	frameshift insertion	HNRNPH1:NM_001364250:exon4:c.304dupG:p.E102Gfs*13,HNRNPH1:NM_001257293:exon5:c.460dupG:p.E154Gfs*13,HNRNPH1:NM_001363572:exon5:c.460dupG:p.E154Gfs*13,HNRNPH1:NM_001364227:exon5:c.460dupG:p.E154Gfs*13,HNRNPH1:NM_001364228:exon5:c.460dupG:p.E154Gfs*13,HNRNPH1:NM_001364229:exon5:c.460dupG:p.E154Gfs*13,HNRNPH1:NM_001364230:exon5:c.460dupG:p.E154Gfs*13,HNRNPH1:NM_001364231:exon5:c.460dupG:p.E154Gfs*13,HNRNPH1:NM_001364232:exon5:c.460dupG:p.E154Gfs*13,HNRNPH1:NM_001364235:exon5:c.460dupG:p.E154fs,HNRNPH1:NM_001364236:exon5:c.460dupG:p.E154Gfs*13,HNRNPH1:NM_001364238:exon5:c.460dupG:p.E154Gfs*13,HNRNPH1:NM_001364239:exon5:c.460dupG:p.E154Gfs*13,HNRNPH1:NM_001364240:exon5:c.460dupG:p.E154Gfs*13,HNRNPH1:NM_001364241:exon5:c.460dupG:p.E154Gfs*13,HNRNPH1:NM_001364242:exon5:c.460dupG:p.E154Gfs*13,HNRNPH1:NM_001364243:exon5:c.460dupG:p.E154Gfs*13,HNRNPH1:NM_001364244:exon5:c.460dupG:p.E154Gfs*13,HNRNPH1:NM_001364245:exon5:c.460dupG:p.E154Gfs*13,HNRNPH1:NM_001364246:exon5:c.460dupG:p.E154Gfs*13,HNRNPH1:NM_001364247:exon5:c.460dupG:p.E154Gfs*13,HNRNPH1:NM_001364248:exon5:c.460dupG:p.E154Gfs*13,HNRNPH1:NM_005520:exon5:c.460dupG:p.E154Gfs*13,HNRNPH1:NM_001364226:exon6:c.460dupG:p.E154fs,HNRNPH1:NM_001364233:exon6:c.460dupG:p.E154Gfs*13,HNRNPH1:NM_001364234:exon6:c.460dupG:p.E154Gfs*13,HNRNPH1:NM_001364237:exon6:c.460dupG:p.E154Gfs*13,HNRNPH1:NM_001364225:exon7:c.460dupG:p.E154fs	0.111	.	.	.	.	Fam9_f_m_aM_dM_uF	0/1;0/0;0/1;0/1;0/1
chr5	179709950	179709950	C	A	CANX	exonic	nonsynonymous SNV	CANX:NM_001364001:exon5:c.C282A:p.F94L,CANX:NM_001364000:exon6:c.C282A:p.F94L,CANX:NM_001024649:exon7:c.C606A:p.F202L,CANX:NM_001363994:exon7:c.C711A:p.F237L,CANX:NM_001363995:exon7:c.C606A:p.F202L,CANX:NM_001363996:exon7:c.C606A:p.F202L,CANX:NM_001363997:exon7:c.C606A:p.F202L,CANX:NM_001363998:exon7:c.C606A:p.F202L,CANX:NM_001363999:exon7:c.C606A:p.F202L,CANX:NM_001746:exon7:c.C606A:p.F202L,CANX:NM_001363993:exon8:c.C768A:p.F256L	.	1.109	.	.	.	Fam82_f_m_aM_uF	0/1;0/0;0/1;0/0
chr5	179720409	179720409	A	G	CANX	exonic	nonsynonymous SNV	CANX:NM_001364001:exon8:c.A707G:p.E236G,CANX:NM_001364000:exon9:c.A707G:p.E236G,CANX:NM_001024649:exon10:c.A1031G:p.E344G,CANX:NM_001363994:exon10:c.A1136G:p.E379G,CANX:NM_001363996:exon10:c.A1031G:p.E344G,CANX:NM_001363997:exon10:c.A1031G:p.E344G,CANX:NM_001363998:exon10:c.A1031G:p.E344G,CANX:NM_001363999:exon10:c.A1031G:p.E344G,CANX:NM_001746:exon10:c.A1031G:p.E344G,CANX:NM_001363993:exon11:c.A1193G:p.E398G,CANX:NM_001363995:exon11:c.A1133G:p.E378G	.	1.112	.	.	.	Fam62_f_m_aM_aM	0/1;0/0;0/1;0/0
chr5	179799111	179799111	A	T	MGAT4B	exonic	nonsynonymous SNV	MGAT4B:NM_054013:exon10:c.T1205A:p.F402Y,MGAT4B:NM_014275:exon11:c.T1160A:p.F387Y	.	1.357	.	3.35E-05	4.19E-05	Fam94_f_m_aM	0/0;0/1;0/1
chr5	179966928	179966928	A	G	RNF130	exonic	nonsynonymous SNV	RNF130:NM_001280801:exon7:c.T1028C:p.L343P,RNF130:NM_018434:exon7:c.T1028C:p.L343P	.	1.079	.	.	.	Fam19_f_m_aM	0/0;0/1;0/1
chr5	180550012	180550012	G	A	CNOT6	exonic	nonsynonymous SNV	CNOT6:NM_001370472:exon3:c.G194A:p.R65Q,CNOT6:NM_001370473:exon3:c.G194A:p.R65Q	.	1.585	.	6.17E-05	2.10E-05	Fam109_f_m_aM	0/0;0/1;0/1
chr5	180630235	180630235	G	A	FLT4	exonic	nonsynonymous SNV	FLT4:NM_001354989:exon4:c.C503T:p.T168M,FLT4:NM_002020:exon4:c.C503T:p.T168M,FLT4:NM_182925:exon4:c.C503T:p.T168M	.	1.62	.	6.54E-05	2.09E-05	Fam66_f_m_aM	0/1;0/0;0/1

chr5	180792670	180792670	G	T	MGAT1	exonic	nonsynonymous SNV	MGAT1:NM_001114619:exon2:c.C302A:p.P101H,MGAT1:NM_001114620:exon2:c.C302A:p.P101H,MGAT1:NM_001364382:exon2:c.C302A:p.P101H,MGAT1:NM_001364383:exon2:c.C302A:p.P101H,MGAT1:NM_001364389:exon2:c.C302A:p.P101H,MGAT1:NM_001364390:exon2:c.C302A:p.P101H,MGAT1:NM_001364392:exon2:c.C302A:p.P101H,MGAT1:NM_002406:exon2:c.C302A:p.P101H,MGAT1:NM_001114617:exon3:c.C302A:p.P101H,MGAT1:NM_001114618:exon3:c.C302A:p.P101H,MGAT1:NM_001364377:exon3:c.C302A:p.P101H,MGAT1:NM_001364379:exon3:c.C302A:p.P101H,MGAT1:NM_001364380:exon3:c.C302A:p.P101H,MGAT1:NM_001364381:exon3:c.C302A:p.P101H,MGAT1:NM_001364384:exon3:c.C302A:p.P101H,MGAT1:NM_001364386:exon3:c.C302A:p.P101H,MGAT1:NM_001364387:exon3:c.C302A:p.P101H,MGAT1:NM_001364391:exon3:c.C302A:p.P101H,MGAT1:NM_001364393:exon3:c.C302A:p.P101H,MGAT1:NM_001364394:exon3:c.C302A:p.P101H,MGAT1:NM_001364395:exon3:c.C302A:p.P101H,MGAT1:NM_001364385:exon4:c.C302A:p.P101H,MGAT1:NM_001364388:exon4:c.C302A:p.P101H	.	1.571	.	1.47E-05	.	Fam73_f_m_aF_dM	0/0;0/1;0/1;0/0
chr5	181195539	181195539	G	T	TRIM7	exonic	nonsynonymous SNV	TRIM7:NM_203297:exon5:c.C617A:p.A206E,TRIM7:NM_203293:exon7:c.C1163A:p.A388E,TRIM7:NM_203294:exon7:c.C539A:p.A180E,TRIM7:NM_203295:exon7:c.C539A:p.A180E,TRIM7:NM_203296:exon7:c.C539A:p.A180E	.	1.771	.	6.59E-05	.	Fam71_f_m_aF	0/0;0/1;0/1
chr6	3076950	3076950	A	T	RIPK1	exonic	nonsynonymous SNV	RIPK1:NM_001354930:exon2:c.A127T:p.I43F,RIPK1:NM_001354931:exon2:c.A127T:p.I43F,RIPK1:NM_003804:exon2:c.A127T:p.I43F	.	1.002	7.37E-05	0.0005	0.0001	Fam120_f_m_aM_aM	0/1;0/0;0/1;0/0
chr6	3273372	3273372	C	T	SLC22A23	exonic	nonsynonymous SNV	SLC22A23:NM_001286455:exon10:c.G901A:p.G301S,SLC22A23:NM_015482:exon10:c.G1744A:p.G582S,SLC22A23:NM_021945:exon11:c.G901A:p.G301S	.	1.036	.	6.22E-05	1.40E-05	Fam44_f_m_aM_uF	0/0;0/1;0/1;0/0
chr6	3850410	3850410	C	T	FAM50B	exonic	nonsynonymous SNV	FAM50B:NM_012135:exon2:c.C599T:p.T200M	.	1.029	0.0007	0.001	0.0006	Fam62_f_m_aM_aM	0/1;0/0;0/0;0/1
chr6	4943665	4943665	A	G	CDYL	exonic	nonsynonymous SNV	CDYL:NM_001368127:exon4:c.A536G:p.K179R,CDYL:NM_001143970:exon5:c.A845G:p.K282R,CDYL:NM_001143971:exon5:c.A845G:p.K282R,CDYL:NM_001368126:exon5:c.A1175G:p.K392R,CDYL:NM_004824:exon5:c.A1241G:p.K414R,CDYL:NM_001368125:exon7:c.A1403G:p.K468R	.	1.744	.	3.27E-05	.	Fam68_f_m_aF_uF_uM	0/1;0/0;0/1;0/1;0/1
chr6	7229463	7229463	T	A	RREB1	exonic	nonsynonymous SNV	RREB1:NM_001003698:exon10:c.T1364A:p.V455E,RREB1:NM_001003699:exon10:c.T1364A:p.V455E,RREB1:NM_001003700:exon10:c.T1364A:p.V455E,RREB1:NM_001168344:exon10:c.T1364A:p.V455E	.	1.319	.	.	.	Fam51_f_m_aM_uF	0/1;0/0;0/1;0/1
chr6	11103779	11103779	A	C	ERVFRD-1	exonic	nonsynonymous SNV	ERVFRD-1:NM_207582:exon2:c.T1532G:p.V511G	.	1.388	.	.	.	Fam112_f_m_aM	0/1;0/0;0/1
chr6	13486704	13486704	C	T	GFOD1	exonic	nonsynonymous SNV	GFOD1:NM_001242629:exon1:c.G187A:p.D63N,GFOD1:NM_018988:exon1:c.G187A:p.D63N	.	1.805	.	.	.	Fam122_f_m_aM	0/1;0/0;0/1
chr6	13711061	13711061	G	A	RANBP9	exonic	nonsynonymous SNV	RANBP9:NM_005493:exon1:c.C445T:p.R149W	.	1.23	.	3.86E-05	5.59E-05	Fam51_f_m_aM_uF	0/0;0/1;0/1;0/1
chr6	17284683	17284683	C	A	RBM24	exonic	nonsynonymous SNV	RBM24:NM_153020:exon2:c.C184A:p.H62N,RBM24:NM_001143941:exon3:c.C145A:p.H49N,RBM24:NM_001143942:exon3:c.C319A:p.H107N	.	1.371	.	0.0005	8.38E-05	Fam110_f_m_aM_aM_uMs	0/0;0/1;0/0;0/1;0/0
chr6	17850333	17850333	A	G	KIF13A	exonic	nonsynonymous SNV	KIF13A:NM_001105566:exon8:c.T707C:p.L236P,KIF13A:NM_001105567:exon8:c.T707C:p.L236P,KIF13A:NM_001105568:exon8:c.T707C:p.L236P,KIF13A:NM_022113:exon8:c.T707C:p.L236P	.	1.109	.	.	.	Fam31_f_m_aM_uF	0/1;0/0;0/1;0/0
chr6	20402254	20402254	G	T	E2F3	exonic	nonsynonymous SNV	E2F3:NM_001949:exon1:c.G22T:p.A8S	.	1.082	0.0002	0.0003	0.0001	Fam105_f_m_aM	0/0;0/1;0/1
chr6	21595615	21595615	T	A	SOX4	exonic	nonsynonymous SNV	SOX4:NM_003107:exon1:c.T1081A:p.Y361N	.	1.456	.	.	1.42E-05	Fam55_f_m_aM_aM_dM	0/0;0/1;0/1;0/1;0/1
chr6	24357835	24357835	G	A	KAAG1	exonic	nonsynonymous SNV	KAAG1:NM_181337:exon1:c.G196A:p.A66T	.	1.761	0.0006	0.0005	9.07E-05	Fam16_f_m_aM_aM	0/1;0/0;0/1;0/1
chr6	26444026	26444026	G	A	BTN3A3	exonic	nonsynonymous SNV	BTN3A3:NM_001242803:exon2:c.G29A:p.C10Y,BTN3A3:NM_006994:exon4:c.G155A:p.C52Y,BTN3A3:NM_197974:exon4:c.G29A:p.C10Y	.	5	.	.	.	Fam26_f_m_aM_uM	0/0;0/1;0/1;0/0
chr6	26501671	26501671	C	A	BTN1A1	exonic	nonsynonymous SNV	BTN1A1:NM_001732:exon3:c.C161A:p.P54Q	.	1.237	.	3.27E-05	2.09E-05	Fam60_f_m_aF	0/1;0/0;0/1
chr6	26501671	26501671	C	A	BTN1A1	exonic	nonsynonymous SNV	BTN1A1:NM_001732:exon3:c.C161A:p.P54Q	.	1.237	.	3.27E-05	2.09E-05	Fam84_f_m_aF	0/1;0/0;0/1
chr6	27824414	27824414	C	T	HIST1H4J	exonic	nonsynonymous SNV	HIST1H4J:NM_021968:exon1:c.C290T:p.T97I	.	3.099	.	1.21E-05	.	Fam85_f_m_aM_aM	0/1;0/0;0/1;0/1
chr6	27890415	27890415	C	G	HIST1H3J	exonic	nonsynonymous SNV	HIST1H3J:NM_003535:exon1:c.G378C:p.Q126H	.	1.338	.	6.92E-05	0.0001	Fam109_f_m_aM	0/1;0/0;0/1
chr6	28573244	28573244	G	T	ZBED9	exonic	nonsynonymous SNV	ZBED9:NM_052923:exon4:c.C2645A:p.A882D,ZBED9:NM_001329616:exon6:c.C2192A:p.A731D	.	1.203	.	.	.	Fam82_f_m_aM_uF	0/0;0/1;0/1;0/1
chr6	28923493	28923493	G	A	TRIM27	exonic	nonsynonymous SNV	TRIM27:NM_006510:exon1:c.C140T:p.A47V	.	2.284	.	3.29E-05	.	Fam22_f_m_aF_aF_uF	0/1;0/0;0/1;0/1;0/0
chr6	29440556	29440556	C	A	OR10C1	exonic	nonsynonymous SNV	OR10C1:NM_013941:exon1:c.C541A:p.Q181K	.	1.248	.	.	.	Fam20_f_m_aM_uF	0/1;0/0;0/1;0/1
chr6	29672821	29672821	G	C	ZFP57	exonic	nonsynonymous SNV	ZFP57:NM_001366333:exon4:c.C1074G:p.H358Q,ZFP57:NM_001109809:exon5:c.C1290G:p.H430Q	.	1.2	.	4.61E-05	1.40E-05	Fam31_f_m_aM_uF	0/1;0/0;0/1;0/0
chr6	29725186	29725186	C	A	HLA-F	exonic	nonsynonymous SNV	HLA-F:NM_001098479:exon4:c.C766A:p.P256T,HLA-F:NM_018950:exon4:c.C766A:p.P256T	.	1.467	0.0003	7.82E-05	6.28E-05	Fam12_f_m_aM_uM_aM	0/0;0/1;0/1;0/1;0/0
chr6	30071508	30071508	T	C	RNF39	exonic	nonsynonymous SNV	RNF39:NM_025236:exon4:c.A866G:p.E289G,RNF39:NM_170769:exon4:c.A866G:p.E289G	.	2.537	.	.	.	Fam117_f_m_aM_aF	0/0;0/1;0/1;0/0

chr6	30342159	30342159	C	G	TRIM39	exonic	nonsynonymous SNV	TRIM39:NM_001369521:exon8:c.C1367G:p.T456S,TRIM39:NM_001369522:exon8:c.C1367G:p.T456S,TRIM39:NM_172016:exon8:c.C1367G:p.T456S,TRIM39:NM_001369523:exon9:c.C1367G:p.T456S,TRIM39:NM_021253:exon9:c.C1457G:p.T486S	.	2.212	.	.	.	Fam44_f_m_aM_uF	0/1;0/0;0/1;0/1
chr6	30345376	30345376	G	A	RPP21;TRIM39-RP	exonic	nonsynonymous SNV	RPP21:NM_001199120:exon2:c.G136A:p.A46T,RPP21:NM_001199121:exon2:c.G136A:p.A46T,RPP21:NM_024839:exon2:c.G136A:p.A46T,TRIM39-RPP21:NM_001199119:exon7:c.G1183A:p.A395T	.	1.376	.	1.15E-05	.	Fam4_f_m_aM	0/0;0/1;0/1
chr6	30345495	30345495	C	G	RPP21;TRIM39-RP	exonic	nonsynonymous SNV	RPP21:NM_001199120:exon3:c.C187G:p.P63A,RPP21:NM_001199121:exon3:c.C163G:p.P55A,RPP21:NM_024839:exon3:c.C163G:p.P55A,TRIM39-RPP21:NM_001199119:exon8:c.C1210G:p.P404A	.	1.428	0.0003	0.0003	0.0002	Fam98_f_m_aM	0/0;0/1;0/1
chr6	30554883	30554883	A	G	GNL1	exonic	nonsynonymous SNV	GNL1:NM_005275:exon4:c.T409C:p.Y137H	.	1.881	.	.	.	Fam102_f_m_aM	0/0;0/1;0/1
chr6	30582439	30582439	G	A	ABCF1	exonic	nonsynonymous SNV	ABCF1:NM_001025091:exon9:c.G724A:p.G242R	.	1.037	.	1.18E-05	.	Fam18_f_m_aM_uF	0/0;0/1;0/1;0/0
chr6	30604624	30604624	C	G	PPP1R10	exonic	nonsynonymous SNV	PPP1R10:NM_002714:exon12:c.G1066C:p.V356L	.	1.89	.	.	.	Fam71_f_m_aF	0/0;0/1;0/1
chr6	30740532	30740532	A	C	FLOT1	exonic	nonsynonymous SNV	FLOT1:NM_001318875:exon6:c.T390G:p.I130M,FLOT1:NM_005803:exon7:c.T534G:p.I178M	.	1.499	.	.	.	Fam85_f_m_aM_aM	0/1;0/0;0/1;0/1
chr6	30915992	30915992	G	A	VARS2	exonic	nonsynonymous SNV	VARS2:NM_001167733:exon5:c.G98A:p.R33H,VARS2:NM_001167734:exon6:c.G608A:p.R203H,VARS2:NM_020442:exon6:c.G518A:p.R173H	.	1.198	7.34E-05	1.12E-05	2.79E-05	Fam18_f_m_aM_uF	0/1;0/0;0/1;0/1
chr6	30921957	30921957	G	A	VARS2	exonic	nonsynonymous SNV	VARS2:NM_001167733:exon18:c.G1348A:p.A450T,VARS2:NM_001167734:exon19:c.G1858A:p.A620T,VARS2:NM_020442:exon19:c.G1768A:p.A590T	.	1.284	.	.	.	Fam12_f_m_aM_uM_aM	0/0;0/1;0/0;0/0;0/1
chr6	30941073	30941073	G	C	MUCL3	exonic	nonsynonymous SNV	MUCL3:NM_080870:exon1:c.G74C:p.W25S	.	1.534	0.0003	0.0003	0.0002	Fam109_f_m_aM	0/1;0/0;0/1
chr6	31145180	31145180	C	T	CCHCR1	exonic	nonsynonymous SNV	CCHCR1:NM_001105563:exon13:c.G1754A:p.R585Q,CCHCR1:NM_001105564:exon13:c.G1862A:p.R621Q,CCHCR1:NM_019052:exon13:c.G1595A:p.R532Q	.	1.36	.	3.28E-05	6.98E-06	Fam50_f_m_aF	0/0;0/1;0/1
chr6	31150972	31150972	G	A	CCHCR1	exonic	nonsynonymous SNV	CCHCR1:NM_001105563:exon5:c.C844T:p.R282W,CCHCR1:NM_001105564:exon5:c.C952T:p.R318W,CCHCR1:NM_019052:exon5:c.C685T:p.R229W	.	1.234	7.35E-05	0.0002	2.79E-05	Fam103_f_m_aM	0/1;0/0;0/1
chr6	31644116	31644116	G	A	BAG6	exonic	nonsynonymous SNV	BAG6:NM_001098534:exon12:c.C1526T:p.S509F,BAG6:NM_001199697:exon12:c.C1526T:p.S509F,BAG6:NM_001199698:exon12:c.C1526T:p.S509F,BAG6:NM_004639:exon12:c.C1544T:p.S515F,BAG6:NM_080702:exon12:c.C1526T:p.S509F,BAG6:NM_080703:exon12:c.C1526T:p.S509F	.	1.287	.	1.12E-05	.	Fam52_f_m_aM	0/0;0/1;0/1
chr6	31696971	31696971	G	A	ABHD16A	exonic	nonsynonymous SNV	ABHD16A:NM_001177515:exon3:c.C307T:p.R103W,ABHD16A:NM_021160:exon5:c.C406T:p.R136W	.	1.456	7.35E-05	0.0003	9.77E-05	Fam112_f_m_aM	0/1;0/0;0/1
chr6	31791737	31791737	G	A	VARS	exonic	nonsynonymous SNV	VARS:NM_006295:exon8:c.C973T:p.R325C	.	1.532	.	1.15E-05	.	Fam14_f_m_aM_aM	0/1;0/0;0/1;0/0
chr6	31859799	31859799	A	G	NEU1	exonic	nonsynonymous SNV	NEU1:NM_000434:exon6:c.T1168C:p.Y390H	.	1.468	.	.	.	Fam69_f_m_aM	0/1;0/0;0/1
chr6	31860123	31860123	C	T	NEU1	exonic	nonsynonymous SNV	NEU1:NM_000434:exon5:c.G940A:p.V314M	.	1.357	.	3.29E-05	.	Fam19_f_m_aM	0/1;0/0;0/1
chr6	31865992	31865992	C	A	SLC44A4	exonic	nonsynonymous SNV	SLC44A4:NM_001178044:exon13:c.G1242T:p.W414C,SLC44A4:NM_001178045:exon14:c.G1140T:p.W380C,SLC44A4:NM_025257:exon14:c.G1368T:p.W456C	.	1.024	.	.	.	Fam7_f_m_aM_aM_uM	0/0;0/1;0/1;0/0;0/1
chr6	31884464	31884464	G	A	EHMT2	exonic	nonsynonymous SNV	EHMT2:NM_001289413:exon19:c.C2768T:p.A923V,EHMT2:NM_001363689:exon20:c.C2870T:p.A957V,EHMT2:NM_025256:exon20:c.C2597T:p.A866V,EHMT2:NM_001318833:exon21:c.C2093T:p.A698V,EHMT2:NM_006709:exon21:c.C2699T:p.A900V	.	1.902	.	1.15E-05	.	Fam89_f_m_aM	0/0;0/1;0/1
chr6	31943960	31943960	C	T	C2	exonic	nonsynonymous SNV	C2:NM_001178063:exon10:c.C1135T:p.R379W,C2:NM_001282457:exon10:c.C1039T:p.R347W,C2:NM_001145903:exon12:c.C1381T:p.R461W,C2:NM_000063:exon14:c.C1777T:p.R593W,C2:NM_001282458:exon14:c.C1690T:p.R564W	.	1.045	.	2.30E-05	2.09E-05	Fam122_f_m_aM	0/1;0/0;0/1
chr6	31961059	31961059	C	A	SKIV2L	exonic	nonsynonymous SNV	SKIV2L:NM_006929:exon7:c.C563A:p.P188H	.	1.288	.	.	.	Fam19_f_m_aM	0/0;0/1;0/1
chr6	31971520	31971520	G	T	DXO	exonic	nonsynonymous SNV	DXO:NM_005510:exon2:c.C156A:p.F52L	.	1.267	.	.	.	Fam113_f_m_aF_raM_uF	0/0;0/0;0/0;0/1;0/0
chr6	32119957	32119957	A	T	ATF6B	exonic	nonsynonymous SNV	ATF6B:NM_001136153:exon9:c.T824A:p.V275E,ATF6B:NM_004381:exon9:c.T833A:p.V278E	.	1.294	0.0004	0.0005	0.0002	Fam48_f_m_aM_uM	0/1;0/0;0/1;0/1
chr6	32196401	32196401	C	T	NOTCH4	exonic	nonsynonymous SNV	NOTCH4:NM_004557:exon29:c.G5221A:p.A1741T	.	1.598	7.35E-05	0.0001	3.49E-05	Fam23_f_m_aM_dF_uFs	0/0;0/1;0/1;0/1;0/0
chr6	32443893	32443893	C	T	HLA-DRA	exonic	nonsynonymous SNV	HLA-DRA:NM_019111:exon4:c.C748T:p.R250C	.	1.479	.	0.0002	4.19E-05	Fam12_f_m_aM_uM_aM	0/1;0/0;0/0;0/0;0/1
chr6	32841588	32841588	G	A	PSMB8	exonic	nonsynonymous SNV	PSMB8:NM_004159:exon5:c.C673T:p.R225C,PSMB8:NM_148919:exon5:c.C685T:p.R229C	.	1.52	.	.	6.98E-06	Fam30_f_m_aM_uM	0/1;0/0;0/1;0/1
chr6	32847643	32847643	A	C	TAP1	exonic	nonsynonymous SNV	TAP1:NM_000593:exon9:c.T1953G:p.F651L,TAP1:NM_001292022:exon9:c.T1170G:p.F390L	.	1.621	.	.	0	Fam19_f_m_aM	0/0;0/1;0/1
chr6	32949683	32949683	G	A	HLA-DMA	exonic	nonsynonymous SNV	HLA-DMA:NM_006120:exon3:c.C580T:p.P194S	.	1.221	.	7.52E-05	.	Fam50_f_m_aF	0/1;0/0;0/1
chr6	33080786	33080786	G	T	HLA-DPB1	exonic	nonsynonymous SNV	HLA-DPB1:NM_002121:exon2:c.G215T:p.G72V	.	2.038	.	1.13E-05	.	Fam4_f_m_aM	0/1;0/0;0/1
chr6	33251559	33251559	G	A	VPS52	exonic	nonsynonymous SNV	VPS52:NM_001289176:exon17:c.C1417T:p.R473W,VPS52:NM_001289174:exon18:c.C1783T:p.R595W,VPS52:NM_001289175:exon19:c.C1609T:p.R537W,VPS52:NM_022553:exon19:c.C1984T:p.R662W	.	2.76	.	3.27E-05	.	Fam113_f_m_aF_raM_uF	0/1;0/0;0/1;0/0;0/0
chr6	33264492	33264492	C	G	VPS52	exonic	nonsynonymous SNV	VPS52:NM_001289176:exon12:c.G839C:p.W280S,VPS52:NM_001289174:exon13:c.G1205C:p.W402S,VPS52:NM_001289175:exon14:c.G1031C:p.W344S,VPS52:NM_022553:exon14:c.G1406C:p.W469S	.	1.388	.	.	.	Fam113_f_m_aF_raM_uF	0/1;0/0;0/1;0/0;0/0
chr6	33270239	33270239	C	G	VPS52	exonic	nonsynonymous SNV	VPS52:NM_022553:exon2:c.G135C:p.L45F	.	1.173	.	.	.	Fam117_f_m_aM_aF	0/1;0/0;0/1;0/1

chr6	33315905	33315905	C	G	ZBTB22	exonic	nonsynonymous SNV	ZBTB22:NM_001145338:exon2:c.G1012C:p.E338Q,ZBTB22:NM_005453:exon2:c.G1012C:p.E338Q	.	1.264	.	.	.	Fam121_f_m_aF_aM	0/1;0/0;0/1;0/1
chr6	33405161	33405161	C	T	KIFC1	exonic	nonsynonymous SNV	KIFC1:NM_002263:exon7:c.C1066T:p.R356W	.	1.229	7.35E-05	0.0001	2.79E-05	Fam84_f_m_aF	0/0;0/1;0/1
chr6	33405596	33405596	A	G	KIFC1	exonic	nonsynonymous SNV	KIFC1:NM_002263:exon7:c.A1501G:p.N501D	.	1.289	.	2.97E-05	.	Fam71_f_m_aF	0/0;0/1;0/1
chr6	33675785	33675785	G	T	ITPR3	exonic	nonsynonymous SNV	ITPR3:NM_002224:exon25:c.G3211T:p.V1071F	.	1.048	0.0002	0.0002	0.0001	Fam37_f_m_aF_uM	0/1;0/0;0/1;0/0
chr6	33695740	33695740	G	A	ITPR3	exonic	nonsynonymous SNV	ITPR3:NM_002224:exon58:c.G7976A:p.R2659H	.	1.936	.	1.12E-05	6.98E-06	Fam122_f_m_aM	0/0;0/1;0/1
chr6	34133396	34133396	G	C	GRM4	exonic	nonsynonymous SNV	GRM4:NM_000841:exon2:c.C101G:p.P34R,GRM4:NM_001256811:exon2:c.C101G:p.P34R	.	1.583	0.0001	4.51E-05	4.88E-05	Fam66_f_m_aM	0/0;0/1;0/1
chr6	34421786	34421786	G	C	RPS10;RPS10-NUI	exonic	nonsynonymous SNV	RPS10:NM_001014:exon4:c.C344G:p.A115G,RPS10-NUDT3:NM_001202470:exon4:c.C344G:p.A115G,RPS10:NM_001203245:exon4:c.C344G:p.A115G,RPS10:NM_001204091:exon4:c.C344G:p.A115G	.	1.688	.	1.12E-05	.	Fam28_f_m_aF_uF	0/1;0/0;0/1;0/0
chr6	34425795	34425795	C	G	RPS10	splicing	.	.	0.221	.	.	.	2.09E-05	Fam6_f_m_aM	0/0;0/1;0/1
chr6	35081099	35081099	C	T	ANKS1A	exonic	nonsynonymous SNV	ANKS1A:NM_015245:exon17:c.C2650T:p.R884C	.	1.049	.	0.0002	6.98E-06	Fam60_f_m_aF	0/0;0/1;0/1
chr6	35241240	35241240	T	C	SCUBE3	exonic	nonsynonymous SNV	SCUBE3:NM_001303136:exon10:c.T1166C:p.L389P,SCUBE3:NM_152753:exon10:c.T1169C:p.L390P	.	2.303	0.0003	0.0008	0.0004	Fam2_f_m_aF	0/0;0/1;0/1
chr6	35509890	35509890	G	A	TULP1	exonic	nonsynonymous SNV	TULP1:NM_001289395:exon5:c.C379T:p.R127C,TULP1:NM_003322:exon6:c.C538T:p.R180C	.	1.057	.	9.81E-05	3.50E-05	Fam108_f_m_aM	0/0;0/1;0/1
chr6	35805857	35805857	G	A	LHFPL5	exonic	nonsynonymous SNV	LHFPL5:NM_182548:exon1:c.G187A:p.G63S	.	1.036	.	0.0004	.	Fam75_f_m_aM	0/1;0/0;0/1
chr6	36230577	36230577	C	A	BRPF3	exonic	nonsynonymous SNV	BRPF3:NM_015695:exon13:c.C3586A:p.P1196T	.	1.043	.	2.24E-05	.	Fam110_f_m_aM_aM_uMs	0/0;0/1;0/0;0/1;0/0
chr6	36479191	36479191	G	A	KCTD20	exonic	nonsynonymous SNV	KCTD20:NM_001286580:exon3:c.G70A:p.G24S,KCTD20:NM_173562:exon4:c.G505A:p.G169S	.	1.228	0.0001	0.0001	0.0001	Fam79_f_m_aM_uM	0/0;0/1;0/1;0/1
chr6	36959205	36959205	C	G	PI16	exonic	nonsynonymous SNV	PI16:NM_153370:exon2:c.C232G:p.H78D,PI16:NM_001199159:exon3:c.C232G:p.H78D	.	1.236	.	5.84E-05	1.40E-05	Fam77_f_m_aM_aF_uM_uFs	0/1;0/0;0/0;0/1;0/1;0/0
chr6	37171017	37171017	G	T	PIM1	exonic	nonsynonymous SNV	PIM1:NM_001243186:exon3:c.G499T:p.D167Y,PIM1:NM_002648:exon3:c.G226T:p.D76Y	.	1.221	0.0001	2.24E-05	2.79E-05	Fam4_f_m_aM	0/1;0/0;0/1
chr6	37453280	37453280	C	T	CMTR1	exonic	nonsynonymous SNV	CMTR1:NM_015050:exon8:c.C745T:p.R249C	.	1.076	.	7.45E-05	2.10E-05	Fam57_f_m_aM_uF	0/0;0/1;0/1;0/1
chr6	37471032	37471032	T	C	CMTR1	exonic	nonsynonymous SNV	CMTR1:NM_015050:exon14:c.T1517C:p.L506P	.	1.363	.	.	.	Fam6_f_m_aM	0/0;0/1;0/1
chr6	37650117	37650117	T	C	MDGA1	exonic	nonsynonymous SNV	MDGA1:NM_153487:exon8:c.A1601G:p.N534S	.	1.252	7.36E-05	2.33E-05	2.79E-05	Fam77_f_m_aM_aF_uM_uFs	0/0;0/1;0/0;0/1;0/1;0/1
chr6	37654818	37654818	G	A	MDGA1	exonic	nonsynonymous SNV	MDGA1:NM_153487:exon5:c.C694T:p.R232W	.	1.49	.	6.57E-05	3.49E-05	Fam47_f_m_aM	0/1;0/0;0/1
chr6	37655863	37655863	G	A	MDGA1	exonic	nonsynonymous SNV	MDGA1:NM_153487:exon4:c.C416T:p.T139M	.	1.871	0.0002	0.0001	6.98E-05	Fam99_f_m_aM_aM	0/0;0/1;0/1;0/0
chr6	37658392	37658392	C	T	MDGA1	exonic	nonsynonymous SNV	MDGA1:NM_153487:exon3:c.G235A:p.A79T	.	1.645	.	3.32E-05	.	Fam38_f_m_aM	0/0;0/1;0/1
chr6	39079178	39079178	T	A	GLP1R	exonic	nonsynonymous SNV	GLP1R:NM_002062:exon10:c.T1021A:p.C341S	.	1.047	0.0002	7.82E-05	5.58E-05	Fam10_f_m_aM_uF	0/0;0/1;0/1;0/1
chr6	40432920	40432920	A	T	LRFN2	exonic	nonsynonymous SNV	LRFN2:NM_020737:exon2:c.T194A:p.I65N	.	1.03	.	3.28E-05	.	Fam4_f_m_aM	0/1;0/0;0/1
chr6	42659757	42659757	A	G	UBR2	exonic	nonsynonymous SNV	UBR2:NM_001363705:exon30:c.A3344G:p.Q1115R,UBR2:NM_015255:exon30:c.A3344G:p.Q1115R	.	1.261	.	1.12E-05	.	Fam91_m_aM_dM_aM_dM	0/1;0/1;0/1;0/0;0/1
chr6	42960792	42960792	C	G	GNMT	exonic	nonsynonymous SNV	GNMT:NM_001318865:exon1:c.C25G:p.R9G,GNMT:NM_018960:exon1:c.C25G:p.R9G	.	2.551	.	.	.	Fam12_f_m_aM_uM_aM	0/1;0/0;0/0;0/0;0/1
chr6	43142233	43142233	C	T	PTK7	exonic	nonsynonymous SNV	PTK7:NM_152881:exon10:c.C1591T:p.R531C,PTK7:NM_152880:exon12:c.C1861T:p.R621C,PTK7:NM_001270398:exon13:c.C2005T:p.R669C,PTK7:NM_002821:exon13:c.C1981T:p.R661C	.	2.128	.	3.28E-05	1.40E-05	Fam108_f_m_aM	0/1;0/0;0/1
chr6	43188541	43188541	G	A	CUL9	exonic	nonsynonymous SNV	CUL9:NM_015089:exon8:c.G2006A:p.R669Q	.	1.151	7.34E-05	0.0002	6.98E-06	Fam85_f_m_aM_aM	0/0;0/1;0/1;0/1
chr6	43204488	43204488	G	A	CUL9	exonic	nonsynonymous SNV	CUL9:NM_015089:exon21:c.G4288A:p.V1430M	.	1.08	.	0.0004	3.49E-05	Fam57_f_m_aM_uF	0/1;0/0;0/1;0/0
chr6	43221273	43221273	G	A	CUL9	exonic	nonsynonymous SNV	CUL9:NM_015089:exon34:c.G6704A:p.R2235H	.	2.551	.	9.84E-05	6.99E-06	Fam63_f_m_aF	0/1;0/0;0/1
chr6	43226051	43226051	G	A	DNPH1	exonic	nonsynonymous SNV	DNPH1:NM_006443:exon3:c.C358T:p.R120C,DNPH1:NM_199184:exon3:c.C358T:p.R120C	.	1.385	.	0.0002	3.49E-05	Fam63_f_m_aF	0/1;0/0;0/1
chr6	43226138	43226138	C	T	DNPH1	exonic	nonsynonymous SNV	DNPH1:NM_006443:exon3:c.G271A:p.V91M,DNPH1:NM_199184:exon3:c.G271A:p.V91M	.	1.179	0.0006	0.001	0.0001	Fam67_f_m_aM_uF	0/0;0/1;0/1;0/0
chr6	44265406	44265406	G	A	NFKBIE	exonic	nonsynonymous SNV	NFKBIE:NM_004556:exon1:c.C358T:p.R120W	.	1.184	.	0.0001	6.98E-06	Fam32_f_m_aM_uM	0/1;0/0;0/1;0/1
chr6	46662498	46662498	G	A	SLC25A27	exonic	nonsynonymous SNV	SLC25A27:NM_001204051:exon4:c.G506A:p.R169Q,SLC25A27:NM_001204052:exon4:c.G506A:p.R169Q,SLC25A27:NM_004277:exon4:c.G506A:p.R169Q	.	1.44	0.0003	0.0002	0.0001	Fam13_f_m_aM	0/1;0/0;0/1
chr6	56463131	56463131	T	C	DST	exonic	nonsynonymous SNV	DST:NM_015548:exon82:c.A15044G:p.Y5015C,DST:NM_183380:exon92:c.A16022G:p.Y5341C,DST:NM_001144770:exon93:c.A16142G:p.Y5381C,DST:NM_001144769:exon95:c.A16556G:p.Y5519C	.	1.532	.	0.0006	1.40E-05	Fam116_f_m_aM_aF	0/1;0/0;0/1;0/0
chr6	56470213	56470213	A	T	DST	exonic	nonsynonymous SNV	DST:NM_015548:exon77:c.T14522A:p.I4841N,DST:NM_183380:exon87:c.T15500A:p.I5167N,DST:NM_001144770:exon88:c.T15620A:p.I5207N,DST:NM_001144769:exon90:c.T16034A:p.I5345N	.	1.824	.	.	.	Fam54_f_m_aM_uF	0/1;0/0;0/1;0/1
chr6	56487174	56487174	G	T	DST	exonic	nonsynonymous SNV	DST:NM_015548:exon68:c.C13108A:p.L4370M,DST:NM_183380:exon78:c.C14086A:p.L4696M,DST:NM_001144770:exon79:c.C14206A:p.L4736M,DST:NM_001144769:exon81:c.C14620A:p.L4874M	.	1.293	.	7.71E-05	.	Fam49_f_m_aM	0/1;0/0;0/1

chr6	72213125	72213125	G	A	RIMS1	exonic	startloss	RIMS1:NM_001168407:exon1:c.G3A:p.M1?,RIMS1:NM_001168408:exon1:c.G3A:p.M1?,RIMS1:NM_001350414:exon1:c.G3A:p.M1?,RIMS1:NM_001350415:exon1:c.G3A:p.M1?,RIMS1:NM_001350416:exon1:c.G3A:p.M1?,RIMS1:NM_001350417:exon1:c.G3A:p.M1?,RIMS1:NM_001350418:exon1:c.G3A:p.M1?,RIMS1:NM_001350419:exon1:c.G3A:p.M1?,RIMS1:NM_001350420:exon1:c.G3A:p.M1?,RIMS1:NM_001350422:exon1:c.G3A:p.M1?,RIMS1:NM_001350423:exon1:c.G3A:p.M1?,RIMS1:NM_001350425:exon1:c.G3A:p.M1?,RIMS1:NM_001350426:exon1:c.G3A:p.M1?,RIMS1:NM_001350427:exon1:c.G3A:p.M1?,RIMS1:NM_001350429:exon1:c.G3A:p.M1?,RIMS1:NM_001350431:exon1:c.G3A:p.M1?,RIMS1:NM_001350432:exon1:c.G3A:p.M1?,RIMS1:NM_001350433:exon1:c.G3A:p.M1?,RIMS1:NM_001350434:exon1:c.G3A:p.M1?,RIMS1:NM_001350435:exon1:c.G3A:p.M1?,RIMS1:NM_001350436:exon1:c.G3A:p.M1?,RIMS1:NM_001350438:exon1:c.G3A:p.M1?,RIMS1:NM_001350439:exon1:c.G3A:p.M1?,RIMS1:NM_001350440:exon1:c.G3A:p.M1?,RIMS1:NM_001350441:exon1:c.G3A:p.M1?,RIMS1:NM_001350442:exon1:c.G3A:p.M1?,RIMS1:NM_001350443:exon1:c.G3A:p.M1?,RIMS1:NM_001350444:exon1:c.G3A:p.M1?,RIMS1:NM_001350445:exon1:c.G3A:p.M1?,RIMS1:NM_001350446:exon1:c.G3A:p.M1?,RIMS1:NM_001350447:exon1:c.G3A:p.M1?,RIMS1:NM_001350448:exon1:c.G3A:p.M1?,RIMS1:NM_001350449:exon1:c.G3A:p.M1?,RIMS1:NM_001350452:exon1:c.G3A:p.M1?,RIMS1:NM_001350454:exon1:c.G3A:p.M1?,RIMS1:NM_001350455:exon1:c.G3A:p.M1?,RIMS1:NM_001350456:exon1:c.G3A:p.M1?,RIMS1:NM_001350457:exon1:c.G3A:p.M1?,RIMS1:NM_001350458:exon1:c.G3A:p.M1?,RIMS1:NM_001350460:exon1:c.G3A:p.M1?	0.496	.	.	4.89E-05	2.09E-05	Fam70_f_m_aM	0/0;0/1;0/1
chr6	72248111	72248111	-	A	RIMS1	exonic	frameshift insertion	RIMS1:NM_001168407:exon7:c.648dupA:p.P217Tfs*11,RIMS1:NM_001168408:exon7:c.648dupA:p.P217Tfs*11,RIMS1:NM_001168409:exon7:c.405dupA:p.P136Tfs*11,RIMS1:NM_001168410:exon7:c.603dupA:p.P202Tfs*11,RIMS1:NM_001350414:exon7:c.648dupA:p.P217Tfs*11,RIMS1:NM_001350415:exon7:c.648dupA:p.P217Tfs*11,RIMS1:NM_001350416:exon7:c.648dupA:p.P217Tfs*11,RIMS1:NM_001350417:exon7:c.648dupA:p.P217Tfs*11,RIMS1:NM_001350418:exon7:c.648dupA:p.P217Tfs*11,RIMS1:NM_001350419:exon7:c.648dupA:p.P217Tfs*11,RIMS1:NM_001350420:exon7:c.648dupA:p.P217Tfs*11,RIMS1:NM_001350422:exon7:c.648dupA:p.P217Tfs*11,RIMS1:NM_001350423:exon7:c.648dupA:p.P217Tfs*11,RIMS1:NM_001350425:exon7:c.648dupA:p.P217Tfs*11,RIMS1:NM_001350426:exon7:c.648dupA:p.P217Tfs*11,RIMS1:NM_001350427:exon7:c.648dupA:p.P217Tfs*11,RIMS1:NM_001350429:exon7:c.648dupA:p.P217Tfs*11,RIMS1:NM_001350431:exon7:c.648dupA:p.P217Tfs*11,RIMS1:NM_001350432:exon7:c.648dupA:p.P217Tfs*11,RIMS1:NM_001350433:exon7:c.648dupA:p.P217Tfs*11,RIMS1:NM_001350434:exon7:c.648dupA:p.P217Tfs*11,RIMS1:NM_001350435:exon7:c.648dupA:p.P217Tfs*11,RIMS1:NM_001350436:exon7:c.648dupA:p.P217Tfs*11,RIMS1:NM_001350438:exon7:c.648dupA:p.P217Tfs*11,RIMS1:NM_001350439:exon7:c.648dupA:p.P217Tfs*11,RIMS1:NM_001350440:exon7:c.648dupA:p.P217Tfs*11,RIMS1:NM_001350441:exon7:c.648dupA:p.P217Tfs*11,RIMS1:NM_001350442:exon7:c.648dupA:p.P217Tfs*11,RIMS1:NM_001350443:exon7:c.648dupA:p.P217Tfs*11,RIMS1:NM_001350444:exon7:c.648dupA:p.P217Tfs*11,RIMS1:NM_001350445:exon7:c.648dupA:p.P217Tfs*11,RIMS1:NM_001350446:exon7:c.648dupA:p.P217Tfs*11,RIMS1:NM_001350447:exon7:c.648dupA:p.P217Tfs*11,RIMS1:NM_001350448:exon7:c.648dupA:p.P217Tfs*11,RIMS1:NM_001350449:exon7:c.648dupA:p.P217Tfs*11,RIMS1:NM_001350452:exon7:c.648dupA:p.P217Tfs*11,RIMS1:NM_001350454:exon7:c.648dupA:p.P217Tfs*11,RIMS1:NM_001350455:exon7:c.648dupA:p.P217Tfs*11,RIMS1:NM_001350456:exon7:c.648dupA:p.P217Tfs*11,RIMS1:NM_001350457:exon7:c.648dupA:p.P217Tfs*11,RIMS1:NM_001350458:exon7:c.648dupA:	0.496	.	.	.	.	Fam120_f_m_aM_aM	0/1;0/0;0/1;0/1
chr6	73368860	73368860	T	G	OOEP	exonic	nonsynonymous SNV	OOEP:NM_001080507:exon3:c.A374C:p.E125A	.	1.036	.	.	.	Fam55_f_m_aM_aM_dM	0/1;0/0;0/0;0/1;0/1
chr6	79214974	79214974	C	T	HMGN3	exonic	nonsynonymous SNV	HMGN3:NM_001201362:exon2:c.G64A:p.E22K,HMGN3:NM_001201363:exon2:c.G64A:p.E22K,HMGN3:NM_001318884:exon2:c.G64A:p.E22K,HMGN3:NM_001318886:exon2:c.G64A:p.E22K,HMGN3:NM_001318887:exon2:c.G64A:p.E22K,HMGN3:NM_001318888:exon2:c.G64A:p.E22K,HMGN3:NM_004242:exon2:c.G64A:p.E22K,HMGN3:NM_138730:exon2:c.G64A:p.E22K	.	1.321	.	.	.	Fam27_f_m_aF_uM_uM	0/0;0/1;0/1;0/0;0/0
chr6	81751916	81751916	C	G	TENT5A	exonic	nonsynonymous SNV	TENT5A:NM_017633:exon2:c.G226C:p.G76R	.	1.121	.	1.14E-05	.	Fam77_f_m_aM_aF_uM_uFs	0/1;0/0;0/0;0/1;0/1;0/0

chr6	87015897	87015897	T	G	HTR1E	exonic	nonsynonymous SNV	HTR1E:NM_000865:exon2:c.T563G:p.L188R	.	1.43	.	.	.	Fam72_f_m_aF_uF	0/0;0/1;0/1;0/1
chr6	87016258	87016258	C	G	HTR1E	exonic	nonsynonymous SNV	HTR1E:NM_000865:exon2:c.C924G:p.F308L	.	1.457	.	.	.	Fam71_f_m_aF	0/0;0/1;0/1
chr6	89098933	89098933	C	T	SRSF12	exonic	nonsynonymous SNV	SRSF12:NM_080743:exon5:c.G431A:p.R144Q	.	1.133	0.0006	0.0007	2.79E-05	Fam22_f_m_aF_aF_uF	0/1;0/0;0/1;0/1;0/0
chr6	89146344	89146344	A	G	PM20D2	exonic	nonsynonymous SNV	PM20D2:NM_001010853:exon1:c.A200G:p.E67G	.	1.082	.	0.0004	2.09E-05	Fam105_f_m_aM	0/0;0/1;0/1
chr6	90544605	90544605	C	T	MAP3K7	exonic	nonsynonymous SNV	MAP3K7:NM_145331:exon12:c.G1238A:p.R413H,MAP3K7:NM_145332:exon12:c.G1238A:p.R413H	.	1.3	.	1.12E-05	.	Fam92_f_m_aM_raM	0/1;0/0;0/1;0/1
chr6	107070229	107070229	G	A	BEND3	exonic	nonsynonymous SNV	BEND3:NM_001367314:exon4:c.C962T:p.T321M,BEND3:NM_001080450:exon5:c.C962T:p.T321M	.	1.061	.	3.29E-05	.	Fam102_f_m_aM	0/1;0/0;0/1
chr6	109979671	109979671	C	T	GPR6	exonic	nonsynonymous SNV	GPR6:NM_005284:exon2:c.C559T:p.L187F,GPR6:NM_001286099:exon3:c.C604T:p.L202F	.	1.762	.	3.27E-05	1.40E-05	Fam33_f_m_aM_uM	0/0;0/1;0/1;0/1
chr6	111562995	111562995	G	C	TRAF3IP2	exonic	nonsynonymous SNV	TRAF3IP2:NM_001164283:exon3:c.C153G:p.F51L,TRAF3IP2:NM_001164281:exon8:c.C1518G:p.F506L,TRAF3IP2:NM_147686:exon8:c.C1521G:p.F507L,TRAF3IP2:NM_147200:exon9:c.C1548G:p.F516L	.	2.382	.	.	.	Fam19_f_m_aM	0/0;0/1;0/1
chr6	117676001	117676001	G	C	NUS1	exonic	nonsynonymous SNV	NUS1:NM_138459:exon1:c.G331C:p.E111Q	.	2.946	.	.	.	Fam121_f_m_aF_aM	0/1;0/0;0/0;0/1
chr6	118314152	118314152	C	A	SLC35F1	exonic	nonsynonymous SNV	SLC35F1:NM_001029858:exon8:c.C1127A:p.P376H	.	1.352	0.0008	0.0009	0.0004	Fam44_f_m_aM_uF	0/1;0/0;0/1;0/1
chr6	122997919	122997919	C	G	CLVS2	exonic	nonsynonymous SNV	CLVS2:NM_001010852:exon2:c.C142G:p.R48G	.	1.297	.	.	.	Fam44_f_m_aM_uF	0/0;0/0;0/1;0/0
chr6	122997994	122997994	G	C	CLVS2	exonic	nonsynonymous SNV	CLVS2:NM_001010852:exon2:c.G217C:p.A73P	.	1.837	.	.	.	Fam87_f_m_aM_uM	0/0;0/1;0/1;0/0
chr6	123010985	123010985	G	T	CLVS2	exonic	nonsynonymous SNV	CLVS2:NM_001010852:exon3:c.G390T:p.R130S	.	1.034	.	.	.	Fam80_f_m_aM_uM	0/0;0/1;0/1;0/1
chr6	136924112	136924112	C	G	SLC35D3	exonic	nonsynonymous SNV	SLC35D3:NM_001008783:exon2:c.C667G:p.P223A	.	1.528	.	.	.	Fam7_f_m_aM_aM_uM	0/0;0/1;0/0;0/1;0/0
chr6	137017103	137017103	A	T	IL20RA	exonic	nonsynonymous SNV	IL20RA:NM_014432:exon2:c.T89A:p.V30D	.	1.072	.	1.12E-05	.	Fam54_f_m_aM_uF	0/0;0/1;0/1;0/1
chr6	137493999	137493999	G	A	OLIG3	exonic	nonsynonymous SNV	OLIG3:NM_175747:exon1:c.C172T:p.R58W	.	1.234	.	.	.	Fam81_f_m_aM_uM	0/1;0/0;0/1;0/0
chr6	137874994	137874994	T	C	TNFAIP3	exonic	nonsynonymous SNV	TNFAIP3:NM_001270507:exon3:c.T445C:p.S149P,TNFAIP3:NM_001270508:exon3:c.T445C:p.S149P,TNFAIP3:NM_006290:exon3:c.T445C:p.S149P	.	1.151	.	.	.	Fam23_f_m_aM_dF_uFs	0/1;0/0;0/1;0/1;0/0
chr6	137881303	137881303	A	C	TNFAIP3	exonic	nonsynonymous SNV	TNFAIP3:NM_001270507:exon9:c.A2357C:p.K786T,TNFAIP3:NM_001270508:exon9:c.A2357C:p.K786T,TNFAIP3:NM_006290:exon9:c.A2357C:p.K786T	.	1.119	.	4.33E-05	.	Fam38_f_m_aM	0/0;0/1;0/1
chr6	146304726	146304726	A	G	GRM1	exonic	nonsynonymous SNV	GRM1:NM_001278064:exon3:c.A1066G:p.K356E,GRM1:NM_001278066:exon3:c.A1066G:p.K356E,GRM1:NM_001278067:exon3:c.A1066G:p.K356E,GRM1:NM_001278065:exon4:c.A1066G:p.K356E	.	1.077	.	.	.	Fam12_f_m_aM_uM_aM	0/1;0/0;0/1;0/1;0/1
chr6	147363355	147363355	G	A	STXBP5	exonic	nonsynonymous SNV	STXBP5:NM_139244:exon22:c.G2458A:p.G820S,STXBP5:NM_001127715:exon24:c.G2566A:p.G856S	.	1.404	.	7.19E-05	2.09E-05	Fam121_f_m_aF_aM	0/1;0/0;0/1;0/0
chr6	148747461	148747461	G	C	UST	exonic	nonsynonymous SNV	UST:NM_005715:exon1:c.G31C:p.G11R	.	1.004	0.0003	0.0003	0.0003	Fam6_f_m_aM	0/1;0/0;0/1
chr6	149820601	149820601	C	T	LRP11	exonic	nonsynonymous SNV	LRP11:NM_032832:exon7:c.G1451A:p.R484H	.	1.096	.	0.0008	2.10E-05	Fam5_f_m_aM	0/0;0/1;0/1
chr6	150214760	150214760	A	G	PPP1R14C	exonic	nonsynonymous SNV	PPP1R14C:NM_030949:exon2:c.A323G:p.E108G	.	1.724	.	3.36E-05	.	Fam102_f_m_aM	0/0;0/1;0/1
chr6	154442133	154442133	A	G	CNKSR3	exonic	nonsynonymous SNV	CNKSR3:NM_001368116:exon3:c.T392C:p.V131A,CNKSR3:NM_001368117:exon3:c.T374C:p.V125A,CNKSR3:NM_001368118:exon3:c.T134C:p.V45A,CNKSR3:NM_173515:exon3:c.T374C:p.V125A	.	1.012	.	.	.	Fam16_f_m_aM_aM	0/0;0/1;0/1;0/1
chr6	156829332	156829332	T	C	ARID1B	exonic	nonsynonymous SNV	ARID1B:NM_001346813:exon2:c.T1648C:p.Y550H,ARID1B:NM_017519:exon2:c.T1648C:p.Y550H,ARID1B:NM_020732:exon2:c.T1648C:p.Y550H	.	1.144	.	.	6.98E-06	Fam73_f_m_aF_dM	0/0;0/1;0/1;0/0
chr6	158449062	158449062	G	A	TULP4	exonic	nonsynonymous SNV	TULP4:NM_001007466:exon4:c.G610A:p.V204I,TULP4:NM_020245:exon4:c.G610A:p.V204I	.	1.04	.	0.0001	9.77E-05	Fam21_f_m_aM_uM	0/0;0/1;0/1;0/0
chr6	158771340	158771340	T	C	EZR	exonic	nonsynonymous SNV	EZR:NM_003379:exon8:c.A863G:p.H288R,EZR:NM_001111077:exon9:c.A863G:p.H288R	.	1.163	.	.	.	Fam45_f_m_aM_uF	0/1;0/0;0/1;0/0
chr6	160738583	160738583	G	C	PLG	exonic	nonsynonymous SNV	PLG:NM_000301:exon15:c.G1848C:p.W616C	.	1.026	.	1.12E-05	.	Fam70_f_m_aM	0/0;0/1;0/1
chr6	165339323	165339323	A	C	PDE10A	exonic	nonsynonymous SNV	PDE10A:NM_001130690:exon20:c.T2133G:p.I711M,PDE10A:NM_006661:exon21:c.T2103G:p.I701M	.	1.666	0.0003	0.0006	0.0003	Fam77_f_m_aM_aF	0/0;0/1;0/0;0/1
chr6	166366043	166366043	A	T	MPC1	exonic	nonsynonymous SNV	MPC1:NM_001270879:exon4:c.T107A:p.L36H	.	1.44	.	.	.	Fam106_f_m_aM	0/0;0/1;0/1
chr6	166459457	166459457	G	A	RPS6KA2	exonic	nonsynonymous SNV	RPS6KA2:NM_001318937:exon10:c.C773T:p.T258M,RPS6KA2:NM_001318938:exon12:c.C800T:p.T267M,RPS6KA2:NM_021135:exon12:c.C1067T:p.T356M,RPS6KA2:NM_001006932:exon13:c.C1091T:p.T364M,RPS6KA2:NM_001318936:exon14:c.C1142T:p.T381M	.	1.349	.	0.0004	5.58E-05	Fam114_f_m_aM	0/0;0/1;0/1
chr6	167827175	167827175	G	A	AFDN	exonic	nonsynonymous SNV	AFDN:NM_001040000:exon1:c.G43A:p.D15N,AFDN:NM_001207008:exon1:c.G43A:p.D15N,AFDN:NM_001366319:exon1:c.G43A:p.D15N,AFDN:NM_001366320:exon1:c.G43A:p.D15N,AFDN:NM_001366321:exon1:c.G43A:p.D15N	.	1.519	.	.	.	Fam121_f_m_aF_aM	0/1;0/0;0/1;0/1
chr6	167951822	167951822	C	G	AFDN	exonic	nonsynonymous SNV	AFDN:NM_001207008:exon28:c.C4396G:p.P1466A,AFDN:NM_001040000:exon29:c.C4447G:p.P1483A,AFDN:NM_001291964:exon29:c.C4324G:p.P1442A,AFDN:NM_001366319:exon29:c.C4447G:p.P1483A,AFDN:NM_001366320:exon29:c.C4447G:p.P1483A,AFDN:NM_001366321:exon29:c.C4426G:p.P1476A	.	1.071	0.0001	0.0001	0.0001	Fam57_f_m_aM_uF	0/0;0/1;0/1;0/1
chr6	169225180	169225180	C	T	THBS2	exonic	nonsynonymous SNV	THBS2:NM_003247:exon18:c.G2738A:p.R913Q	.	1.54	7.34E-05	0.0007	1.40E-05	Fam116_f_m_aM_aF	0/1;0/0;0/1;0/1
chr6	169232110	169232110	G	T	THBS2	exonic	nonsynonymous SNV	THBS2:NM_003247:exon14:c.C2021A:p.P674H	.	1.539	.	1.12E-05	.	Fam21_f_m_aM_uM	0/1;0/0;0/1;0/0
chr6	169232901	169232901	C	T	THBS2	exonic	nonsynonymous SNV	THBS2:NM_003247:exon12:c.G1768A:p.D590N	.	1.361	7.35E-05	0.0002	4.19E-05	Fam1_f_m_aM	0/1;0/0;0/1
chr6	169248895	169248895	T	G	THBS2	exonic	nonsynonymous SNV	THBS2:NM_003247:exon4:c.A131C:p.Q44P	.	1.567	.	.	.	Fam109_f_m_aM	0/0;0/1;0/1
chr6	169704062	169704062	G	A	PHF10	exonic	nonsynonymous SNV	PHF10:NM_018288:exon12:c.C1438T:p.R480W,PHF10:NM_133325:exon12:c.C1432T:p.R478W	.	1.28	7.35E-05	0.0002	2.10E-05	Fam36_f_m_aM_uM	0/1;0/0;0/1;0/0

chr7	256743	256743	C	T	FAM20C	exonic	nonsynonymous SNV	FAM20C:NM_020223:exon7:c.C1343T:p.T448M	.	1.1	.	0.0001	1.40E-05	Fam62_f_m_aM_aM	0/1;0/0;0/1;0/1
chr7	2526264	2526264	C	T	LFNG	exonic	nonsynonymous SNV	LFNG:NM_001040167:exon6:c.C842T:p.T281M,LFNG:NM_001040168:exon6:c.C842T:p.T281M,LFNG:NM_001166355:exon7:c.C629T:p.T210M,LFNG:NM_002304:exon7:c.C455T:p.T152M	.	1.143	.	0.0002	1.40E-05	Fam118_f_m_aM_aF_uM	0/1;0/0;0/1;0/1;0/1
chr7	4740915	4740915	C	T	FOXK1	exonic	nonsynonymous SNV	FOXK1:NM_001037165:exon2:c.C638T:p.S213F	.	1.175	.	.	.	Fam83_f_m_aF	0/1;0/0;0/1
chr7	5333004	5333004	C	T	TNRC18	exonic	nonsynonymous SNV	TNRC18:NM_001080495:exon19:c.G5765A:p.R1922H	.	1.385	0.0005	0.0002	0.0002	Fam119_f_m_aM_aM	0/1;0/0;0/0;0/1
chr7	5388560	5388560	G	A	TNRC18	exonic	nonsynonymous SNV	TNRC18:NM_001080495:exon5:c.C1264T:p.R422W	.	3.099	.	.	.	Fam33_f_m_aM_uM	0/1;0/0;0/1;0/1
chr7	8071111	8071111	T	G	GLCCI1	exonic	nonsynonymous SNV	GLCCI1:NM_138426:exon6:c.T1157G:p.L386W	.	1.21	.	2.23E-05	.	Fam122_f_m_aM	0/1;0/0;0/1
chr7	11028803	11028803	G	T	PHF14	exonic	nonsynonymous SNV	PHF14:NM_001007157:exon7:c.G1440T:p.E480D,PHF14:NM_014660:exon7:c.G1440T:p.E480D	.	1.291	.	.	.	Fam53_f_m_aM	0/0;0/1;0/1
chr7	17875515	17875515	C	T	SNX13	exonic	nonsynonymous SNV	SNX13:NM_001350862:exon7:c.G629A:p.R210H,SNX13:NM_001350863:exon7:c.G389A:p.R130H,SNX13:NM_001350868:exon7:c.G629A:p.R210H,SNX13:NM_015132:exon7:c.G629A:p.R210H,SNX13:NM_001350864:exon8:c.G323A:p.R108H,SNX13:NM_001350866:exon8:c.G20A:p.R7H,SNX13:NM_001350867:exon8:c.G20A:p.R7H	.	1.34	7.41E-05	6.75E-05	3.59E-05	Fam31_f_m_aM_uF	0/1;0/0;0/1;0/0
chr7	27094495	27094495	G	A	HOXA1	exonic	nonsynonymous SNV	HOXA1:NM_005522:exon2:c.C953T:p.P318L	.	1.034	.	6.17E-05	2.09E-05	Fam68_f_m_aF_uF_uM	0/1;0/0;0/1;0/1;0/0
chr7	27094626	27094626	C	G	HOXA1	exonic	nonsynonymous SNV	HOXA1:NM_005522:exon2:c.G822C:p.K274N	.	1.237	.	5.58E-05	3.49E-05	Fam59_f_m_aF_uM	0/0;0/1;0/1;0/0
chr7	27102220	27102220	T	C	HOXA2	exonic	nonsynonymous SNV	HOXA2:NM_006735:exon1:c.A281G:p.E94G	.	1.194	.	6.14E-05	2.80E-05	Fam118_f_m_aM_aF_uM	0/1;0/0;0/1;0/0;0/0
chr7	27182812	27182812	G	A	HOXA11	exonic	nonsynonymous SNV	HOXA11:NM_005523:exon2:c.C926T:p.A309V	.	1.142	.	3.28E-05	6.28E-05	Fam56_f_m_aF_aM	0/1;0/0;0/1;0/0
chr7	27245957	27245957	C	A	EVX1	exonic	nonsynonymous SNV	EVX1:NM_001304519:exon3:c.C210A:p.D70E,EVX1:NM_001989:exon3:c.C756A:p.D252E,EVX1:NM_001304520:exon4:c.C210A:p.D70E	.	1.025	0.0002	0.0004	0.0004	Fam34_f_m_aM_uF	0/0;0/1;0/1;0/1
chr7	27245957	27245957	C	A	EVX1	exonic	nonsynonymous SNV	EVX1:NM_001304519:exon3:c.C210A:p.D70E,EVX1:NM_001989:exon3:c.C756A:p.D252E,EVX1:NM_001304520:exon4:c.C210A:p.D70E	.	1.025	0.0002	0.0004	0.0004	Fam114_f_m_aM	0/1;0/0;0/1
chr7	29566541	29566541	C	A	PRR15	exonic	nonsynonymous SNV	PRR15:NM_001329996:exon2:c.C212A:p.P71H,PRR15:NM_001329997:exon2:c.C212A:p.P71H,PRR15:NM_175887:exon2:c.C212A:p.P71H	.	1.111	0.0001	0.0003	0.0002	Fam115_f_m_aF_aM_aF	0/1;0/0;0/0;0/1;0/1
chr7	29566541	29566541	C	A	PRR15	exonic	nonsynonymous SNV	PRR15:NM_001329996:exon2:c.C212A:p.P71H,PRR15:NM_001329997:exon2:c.C212A:p.P71H,PRR15:NM_175887:exon2:c.C212A:p.P71H	.	1.111	0.0001	0.0003	0.0002	Fam92_f_m_aM_raM	0/1;0/0;0/1;0/0
chr7	30055308	30055308	C	G	PLEKHA8	exonic	nonsynonymous SNV	PLEKHA8:NM_001363474:exon8:c.C498G:p.F166L,PLEKHA8:NM_001197026:exon9:c.C1005G:p.F335L,PLEKHA8:NM_001197027:exon9:c.C1005G:p.F335L,PLEKHA8:NM_001350973:exon9:c.C1005G:p.F335L,PLEKHA8:NM_001350974:exon9:c.C1005G:p.F335L,PLEKHA8:NM_001350975:exon9:c.C408G:p.F136L,PLEKHA8:NM_001363473:exon9:c.C1005G:p.F335L,PLEKHA8:NM_032639:exon9:c.C1005G:p.F335L	.	1.098	.	.	.	Fam63_f_m_aF	0/1;0/0;0/1
chr7	30612120	30612120	G	C	GARS	exonic	nonsynonymous SNV	GARS:NM_001316772:exon8:c.G744C:p.Q248H,GARS:NM_002047:exon8:c.G906C:p.Q302H	.	1.087	.	1.12E-05	.	Fam113_f_m_aF_raM_uF	0/0;0/1;0/1;0/0;0/1
chr7	30617153	30617153	C	T	GARS	exonic	nonsynonymous SNV	GARS:NM_001316772:exon10:c.C1072T:p.R358C,GARS:NM_002047:exon10:c.C1234T:p.R412C	.	1.317	.	3.37E-05	1.40E-05	Fam82_f_m_aM_uF	0/0;0/1;0/1;0/0
chr7	30665180	30665180	G	A	CRHR2	exonic	nonsynonymous SNV	CRHR2:NM_001202482:exon5:c.C430T:p.R144C,CRHR2:NM_001202483:exon5:c.C433T:p.R145C,CRHR2:NM_001883:exon5:c.C433T:p.R145C,CRHR2:NM_001202475:exon6:c.C514T:p.R172C,CRHR2:NM_001202481:exon7:c.C391T:p.R131C	.	1.011	0.0006	0.0001	6.98E-06	Fam120_f_m_aM_aM	0/0;0/1;0/1;0/1
chr7	31084827	31084827	C	T	ADCYAP1R1	exonic	nonsynonymous SNV	ADCYAP1R1:NM_001199637:exon6:c.C466T:p.R156C,ADCYAP1R1:NM_001118:exon8:c.C529T:p.R177C,ADCYAP1R1:NM_001199635:exon8:c.C529T:p.R177C,ADCYAP1R1:NM_001199636:exon8:c.C529T:p.R177C	.	1.128	0.0006	0.0002	1.40E-05	Fam46_f_m_aM_uM	0/1;0/0;0/1;0/0
chr7	31338318	31338318	G	C	NEUROD6	exonic	nonsynonymous SNV	NEUROD6:NM_022728:exon2:c.C951G:p.D317E	.	1.311	.	.	.	Fam87_f_m_aM_uM	0/0;0/1;0/1;0/0
chr7	31339267	31339267	A	G	NEUROD6	exonic	startloss	NEUROD6:NM_022728:exon2:c.T2C:p.M1?	0.413	.	.	.	.	Fam5_f_m_aM	0/0;0/1;0/1
chr7	37916424	37916424	G	T	SFRP4	exonic	nonsynonymous SNV	SFRP4:NM_003014:exon1:c.C114A:p.N38K	.	1.268	.	.	.	Fam49_f_m_aM	0/0;0/1;0/1
chr7	38207719	38207719	T	C	STARD3NL	exonic	nonsynonymous SNV	STARD3NL:NM_001363345:exon2:c.T215C:p.I72T,STARD3NL:NM_032016:exon2:c.T215C:p.I72T,STARD3NL:NM_001363339:exon3:c.T215C:p.I72T,STARD3NL:NM_001363340:exon3:c.T215C:p.I72T,STARD3NL:NM_001363343:exon3:c.T215C:p.I72T,STARD3NL:NM_001363344:exon3:c.T215C:p.I72T,STARD3NL:NM_001363347:exon3:c.T215C:p.I72T,STARD3NL:NM_001363346:exon4:c.T215C:p.I72T	.	1.16	.	.	.	Fam33_f_m_aM_uM	0/0;0/1;0/1;0/1
chr7	39950657	39950657	G	A	CDK13	exonic	nonsynonymous SNV	CDK13:NM_003718:exon1:c.G16A:p.D6N,CDK13:NM_031267:exon1:c.G16A:p.D6N	.	1.702	.	.	.	Fam44_f_m_aM_uF	0/1;0/0;0/1;0/0
chr7	41689931	41689931	C	T	INHBA	exonic	nonsynonymous SNV	INHBA:NM_002192:exon3:c.G1000A:p.G334S	.	1.775	7.35E-05	0.0003	0.0001	Fam94_f_m_aM	0/1;0/0;0/1
chr7	41977590	41977590	C	T	GLI3	exonic	nonsynonymous SNV	GLI3:NM_000168:exon12:c.G1780A:p.A594T	.	2.089	.	.	.	Fam12_f_m_aM_uM_aM	0/0;0/1;0/1;0/0;0/0
chr7	43877109	43877109	G	A	URGCP	exonic	nonsynonymous SNV	URGCP:NM_017920:exon5:c.C2327T:p.T776I,URGCP:NM_001077663:exon6:c.C2354T:p.T785I,URGCP:NM_001077664:exon6:c.C2225T:p.T742I,URGCP:NM_001290075:exon6:c.C2225T:p.T742I,URGCP:NM_001290076:exon7:c.C2225T:p.T742I	.	1.078	0.0004	0.0005	0.0004	Fam39_f_m_aM	0/1;0/0;0/1
chr7	44153367	44153367	C	T	GCK	exonic	nonsynonymous SNV	GCK:NM_000162:exon2:c.G142A:p.E48K,GCK:NM_001354800:exon2:c.G142A:p.E48K,GCK:NM_033507:exon2:c.G145A:p.E49K,GCK:NM_033508:exon3:c.G139A:p.E47K	.	1.863	.	5.59E-05	.	Fam92_f_m_aM_raM	0/0;0/1;0/1;0/0

chr7	44206388	44206388	A	G	YKT6	exonic	nonsynonymous SNV	YKT6:NM_001363678:exon3:c.A191G:p.Y64C,YKT6:NM_006555:exon3:c.A191G:p.Y64C	.	1.123	0.0007	0.0008	0.0007	Fam39_f_m_aM	0/1;0/0;0/1
chr7	44697682	44697682	C	T	OGDH	exonic	nonsynonymous SNV	OGDH:NM_001165036:exon17:c.C2246T:p.T749M,OGDH:NM_002541:exon17:c.C2258T:p.T753M,OGDH:NM_001363523:exon18:c.C2291T:p.T764M	.	1.161	.	2.23E-05	.	Fam12_f_m_aM_uM_aM	0/0;0/1;0/1;0/0;0/0
chr7	44969833	44969833	G	A	MYO1G	exonic	nonsynonymous SNV	MYO1G:NM_033054:exon11:c.C1375T:p.R459W	.	1.083	0.0004	0.0003	0.0002	Fam27_f_m_aF_uM_uM	0/0;0/1;0/1;0/1;0/1
chr7	44976893	44976893	G	A	MYO1G	exonic	nonsynonymous SNV	MYO1G:NM_033054:exon2:c.C274T:p.R92W	.	1.083	.	0.0001	3.49E-05	Fam14_f_m_aM_aM	0/0;0/1;0/1;0/1
chr7	45678049	45678049	G	A	ADCY1	exonic	nonsynonymous SNV	ADCY1:NM_021116:exon9:c.G1786A:p.E596K	.	1.412	0.0004	0.0005	0.0002	Fam10_f_m_aM_uF	0/0;0/1;0/1;0/1
chr7	45678049	45678049	G	A	ADCY1	exonic	nonsynonymous SNV	ADCY1:NM_021116:exon9:c.G1786A:p.E596K	.	1.412	0.0004	0.0005	0.0002	Fam11_f_m_aM_uM	0/1;0/0;0/1;0/1
chr7	45917251	45917251	G	C	IGFBP3	exonic	nonsynonymous SNV	IGFBP3:NM_000598:exon2:c.C592G:p.Q198E,IGFBP3:NM_001013398:exon2:c.C610G:p.Q204E	.	1.014	7.35E-05	0.0009	0.0001	Fam119_f_m_aM_aM	0/1;0/0;0/0;0/1
chr7	55173940	55173940	C	G	EGFR	exonic	nonsynonymous SNV	EGFR:NM_001346941:exon12:c.C1280G:p.P427R,EGFR:NM_001346897:exon17:c.C1946G:p.P649R,EGFR:NM_001346899:exon17:c.C1946G:p.P649R,EGFR:NM_001346898:exon18:c.C2081G:p.P694R,EGFR:NM_001346900:exon18:c.C1922G:p.P641R,EGFR:NM_005228:exon18:c.C2081G:p.P694R	.	1.496	.	.	.	Fam14_f_m_aM_aM	0/1;0/0;0/1;0/1
chr7	55192804	55192804	C	G	EGFR	exonic	nonsynonymous SNV	EGFR:NM_001346941:exon16:c.C1863G:p.H621Q,EGFR:NM_001346897:exon21:c.C2529G:p.H843Q,EGFR:NM_001346899:exon21:c.C2529G:p.H843Q,EGFR:NM_001346898:exon22:c.C2664G:p.H888Q,EGFR:NM_001346900:exon22:c.C2505G:p.H835Q,EGFR:NM_005228:exon22:c.C2664G:p.H888Q	.	1.433	.	1.12E-05	.	Fam6_f_m_aM	0/1;0/0;0/1
chr7	57126044	57126044	C	A	ZNF479	exonic	nonsynonymous SNV	ZNF479:NM_001370129:exon3:c.G236T:p.R79I,ZNF479:NM_033273:exon4:c.G236T:p.R79I	.	2.349	0.0006	.	0.0002	Fam118_f_m_aM_aF_uM	0/0;0/0;0/0;0/1;0/1
chr7	65967873	65967873	T	C	GUSB	exonic	nonsynonymous SNV	GUSB:NM_001284290:exon8:c.A1073G:p.Y358C,GUSB:NM_001293105:exon8:c.A854G:p.Y285C,GUSB:NM_001293104:exon9:c.A941G:p.Y314C,GUSB:NM_000181:exon10:c.A1511G:p.Y504C	.	1.365	.	3.27E-05	.	Fam14_f_m_aM_aM	0/1;0/0;0/1;0/1
chr7	73616133	73616133	T	C	MLXIPL	exonic	nonsynonymous SNV	MLXIPL:NM_032951:exon2:c.A338G:p.K113R,MLXIPL:NM_032952:exon2:c.A338G:p.K113R,MLXIPL:NM_032953:exon2:c.A338G:p.K113R,MLXIPL:NM_032954:exon2:c.A338G:p.K113R	.	1.572	0.0003	8.93E-05	7.68E-05	Fam15_f_m_aM_aM	0/0;0/1;0/1;0/1
chr7	73670082	73670082	G	A	VPS37D	exonic	nonsynonymous SNV	VPS37D:NM_001077621:exon3:c.G373A:p.E125K	.	1.025	.	4.06E-05	.	Fam4_f_m_aM	0/1;0/0;0/1
chr7	74252487	74252487	T	C	RFC2	exonic	nonsynonymous SNV	RFC2:NM_002914:exon2:c.A125G:p.Y42C,RFC2:NM_181471:exon2:c.A125G:p.Y42C	.	1.608	.	.	.	Fam88_f_m_aF	0/0;0/1;0/1
chr7	74357317	74357317	C	T	CLIP2	exonic	nonsynonymous SNV	CLIP2:NM_003388:exon6:c.C1055T:p.S352L,CLIP2:NM_032421:exon6:c.C1055T:p.S352L	.	1.55	.	.	.	Fam92_f_m_aM_raM	0/1;0/0;0/1;0/1
chr7	75563244	75563244	G	A	HIP1	exonic	nonsynonymous SNV	HIP1:NM_001243198:exon10:c.C823T:p.R275C,HIP1:NM_005338:exon10:c.C823T:p.R275C	.	1.254	.	1.12E-05	1.40E-05	Fam7_f_m_aM_aM_uM	0/0;0/1;0/1;0/0;0/0
chr7	76329846	76329846	C	T	YWHAG	exonic	nonsynonymous SNV	YWHAG:NM_012479:exon2:c.G475A:p.E159K	.	1.554	.	1.12E-05	6.99E-06	Fam117_f_m_aM_aF	0/0;0/1;0/1;0/1
chr7	77949692	77949692	C	G	PHTF2	exonic	nonsynonymous SNV	PHTF2:NM_001366084:exon14:c.C1728G:p.H576Q,PHTF2:NM_001366087:exon14:c.C1728G:p.H576Q,PHTF2:NM_020432:exon14:c.C1860G:p.H620Q,PHTF2:NM_001127357:exon15:c.C1872G:p.H624Q,PHTF2:NM_001127358:exon15:c.C1860G:p.H620Q,PHTF2:NM_001366086:exon15:c.C1860G:p.H620Q,PHTF2:NM_001366089:exon16:c.C1974G:p.H658Q	.	1.899	.	7.95E-05	6.98E-06	Fam60_f_m_aF	0/0;0/1;0/1
chr7	78521574	78521574	G	C	MAGI2	exonic	nonsynonymous SNV	MAGI2:NM_001301128:exon4:c.C610G:p.L204V,MAGI2:NM_012301:exon4:c.C610G:p.L204V	.	1.134	.	.	.	Fam84_f_m_aF	0/1;0/0;0/1
chr7	87173600	87173600	C	-	DMTF1	exonic	frameshift deletion	DMTF1:NM_001142326:exon5:c.129delC:p.Q44Kfs*13,DMTF1:NM_001142327:exon6:c.393delC:p.Q132Kfs*13,DMTF1:NM_021145:exon8:c.393delC:p.Q132Kfs*13	0.54	.	.	.	.	Fam29_f_m_aF_uM	0/1;0/0;0/1;0/0
chr7	87406437	87406437	C	T	ABCB4	exonic	nonsynonymous SNV	ABCB4:NM_018850:exon25:c.G3196A:p.G1066R,ABCB4:NM_000443:exon26:c.G3337A:p.G1113R,ABCB4:NM_018849:exon26:c.G3358A:p.G1120R	.	1.052	.	.	6.99E-06	Fam56_f_m_aF_aM	0/0;0/1;0/0;0/1
chr7	87453110	87453110	C	G	ABCB4	exonic	nonsynonymous SNV	ABCB4:NM_000443:exon6:c.G370C:p.G124R,ABCB4:NM_018849:exon6:c.G370C:p.G124R,ABCB4:NM_018850:exon6:c.G370C:p.G124R	.	1.051	.	.	.	Fam95_f_m_aM_aM_uF	0/1;0/0;0/1;0/1;0/0
chr7	92615159	92615159	G	A	CDK6	exonic	nonsynonymous SNV	CDK6:NM_001145306:exon8:c.C962T:p.S321L,CDK6:NM_001259:exon8:c.C962T:p.S321L	.	1.025	.	6.54E-05	6.98E-06	Fam44_f_m_aM_uF	0/0;0/1;0/1;0/0
chr7	96080462	96080462	C	T	DYNC1I1	exonic	nonsynonymous SNV	DYNC1I1:NM_001135557:exon15:c.C1690T:p.R564C,DYNC1I1:NM_001278421:exon15:c.C1741T:p.R581C,DYNC1I1:NM_001278422:exon15:c.C1690T:p.R564C,DYNC1I1:NM_001135556:exon16:c.C1750T:p.R584C,DYNC1I1:NM_004411:exon16:c.C1801T:p.R601C	.	1.657	7.35E-05	3.27E-05	1.40E-05	Fam88_f_m_aF	0/0;0/1;0/1
chr7	97022189	97022189	G	A	DLX5	exonic	nonsynonymous SNV	DLX5:NM_005221:exon2:c.C536T:p.T179I	.	1.011	0.0003	6.70E-05	5.58E-05	Fam18_f_m_aM_uF	0/1;0/0;0/1;0/1
chr7	98950182	98950182	A	T	TRRAP	exonic	nonsynonymous SNV	TRRAP:NM_003496:exon36:c.A5179T:p.S1727C,TRRAP:NM_001244580:exon37:c.A5233T:p.S1745C	.	1.397	.	.	.	Fam119_f_m_aM_aM	0/0;0/1;0/0;0/1
chr7	99353989	99353989	T	C	ARPC1A	exonic	nonsynonymous SNV	ARPC1A:NM_001190996:exon6:c.T539C:p.L180P,ARPC1A:NM_006409:exon6:c.T581C:p.L194P	.	1.312	0.0004	0.0006	0.0003	Fam79_f_m_aM_uM	0/0;0/1;0/1;0/0
chr7	100430188	100430188	C	T	MEPCE	exonic	nonsynonymous SNV	MEPCE:NM_019606:exon1:c.C170T:p.S57F	.	1.345	7.40E-05	.	6.98E-06	Fam102_f_m_aM	0/0;0/1;0/1
chr7	100823775	100823775	T	C	EPHB4	exonic	nonsynonymous SNV	EPHB4:NM_004444:exon3:c.A280G:p.M94V	.	1.372	7.35E-05	1.14E-05	6.98E-06	Fam69_f_m_aM	0/0;0/1;0/1
chr7	101088304	101088304	G	A	TRIM56	exonic	nonsynonymous SNV	TRIM56:NM_030961:exon3:c.G992A:p.R331Q	.	1.551	.	1.63E-05	6.98E-06	Fam32_f_m_aM_uM	0/1;0/0;0/1;0/1
chr7	105624241	105624241	C	A	ATXN7L1	exonic	nonsynonymous SNV	ATXN7L1:NM_138495:exon6:c.G857T:p.S286I,ATXN7L1:NM_001318229:exon8:c.G581T:p.S194I,ATXN7L1:NM_020725:exon8:c.G1229T:p.S410I	.	1.314	.	6.14E-05	6.98E-06	Fam18_f_m_aM_uF	0/1;0/0;0/1;0/1
chr7	107475262	107475262	T	C	GPR22	exonic	nonsynonymous SNV	GPR22:NM_005295:exon3:c.T1202C:p.I401T	.	1.02	7.36E-05	4.50E-05	2.10E-05	Fam91_m_aM_dM_aM_dM	0/1;0/0;0/1;0/1;0/0

chr7	111977210	111977210	C	G	DOCK4	exonic	nonsynonymous SNV	DOCK4:NM_001363540:exon8:c.G623C:p.S208T,DOCK4:NM_014705:exon8:c.G623C:p.S208T	.	1.09	.	.	.	Fam36_f_m_aM_uM	0/0;0/1;0/1;0/0
chr7	120275635	120275635	A	G	KCND2	exonic	nonsynonymous SNV	KCND2:NM_012281:exon1:c.A1003G:p.I335V	.	2.311	.	.	.	Fam20_f_m_aM_uF	0/0;0/1;0/1;0/0
chr7	122886121	122886121	G	A	CADPS2	exonic	nonsynonymous SNV	CADPS2:NM_001009571:exon1:c.C217T:p.R73W,CADPS2:NM_001167940:exon1:c.C217T:p.R73W,CADPS2:NM_001363389:exon1:c.C217T:p.R73W,CADPS2:NM_001363390:exon1:c.C217T:p.R73W,CADPS2:NM_001363391:exon1:c.C217T:p.R73W,CADPS2:NM_001363392:exon1:c.C217T:p.R73W,CADPS2:NM_001363393:exon1:c.C217T:p.R73W,CADPS2:NM_001363394:exon1:c.C217T:p.R73W,CADPS2:NM_001363395:exon1:c.C217T:p.R73W,CADPS2:NM_001363396:exon1:c.C217T:p.R73W,CADPS2:NM_001363397:exon1:c.C217T:p.R73W,CADPS2:NM_001363398:exon1:c.C217T:p.R73W,CADPS2:NM_017954:exon1:c.C217T:p.R73W	.	1.103	.	8.58E-05	.	Fam116_f_m_aM_aF	0/0;0/1;0/0;0/1
chr7	128030912	128030912	C	T	LRRC4	splicing	.	.	0.459	.	.	.	6.98E-06	Fam22_f_m_aF_aF_uF	0/1;0/0;0/1;0/1;0/1
chr7	128830777	128830777	A	G	FLNC	exonic	nonsynonymous SNV	FLNC:NM_001127487:exon1:c.A140G:p.N47S,FLNC:NM_001458:exon1:c.A140G:p.N47S	.	1.273	7.35E-05	5.67E-05	4.19E-05	Fam99_f_m_aM_aM	0/0;0/1;0/0;0/1
chr7	128851458	128851458	G	C	FLNC	exonic	nonsynonymous SNV	FLNC:NM_001127487:exon34:c.G5573C:p.G1858A,FLNC:NM_001458:exon35:c.G5672C:p.G1891A	.	1.804	.	.	.	Fam33_f_m_aM_uM	0/0;0/1;0/1;0/0
chr7	128947996	128947999	CCAT	-	IRF5	exonic	frameshift deletion	IRF5:NM_001242452:exon6:c.749_752del:p.I251Afs*84,IRF5:NM_001098627:exon7:c.1007_1010del:p.I337Afs*84,IRF5:NM_001098629:exon7:c.1055_1058del:p.I353Afs*84,IRF5:NM_001098630:exon7:c.1007_1010del:p.I337Afs*84,IRF5:NM_001347928:exon7:c.1055_1058del:p.I353Afs*84,IRF5:NM_001364314:exon7:c.1055_1058del:p.I353Afs*84,IRF5:NM_032643:exon7:c.1007_1010del:p.I337Afs*84	0.537	.	.	.	.	Fam53_f_m_aM	0/1;0/0;0/1
chr7	129205369	129205369	C	T	SMO	exonic	nonsynonymous SNV	SMO:NM_005631:exon3:c.C704T:p.A235V	.	1.009	7.35E-05	0.0003	0.0001	Fam25_f_m_aM	0/1;0/0;0/1
chr7	129205782	129205782	C	T	SMO	exonic	nonsynonymous SNV	SMO:NM_005631:exon4:c.C920T:p.T307I	.	1.13	7.35E-05	8.95E-05	6.29E-05	Fam28_f_m_aF_uF	0/0;0/1;0/1;0/0
chr7	132174797	132174797	G	A	PLXNA4	exonic	nonsynonymous SNV	PLXNA4:NM_020911:exon21:c.C3998T:p.P1333L	.	1.578	.	.	.	Fam101_f_m_aM	0/1;0/0;0/1
chr7	132211112	132211112	A	T	PLXNA4	exonic	nonsynonymous SNV	PLXNA4:NM_020911:exon10:c.T2129A:p.I710N	.	2.031	7.35E-05	1.67E-05	1.40E-05	Fam115_f_m_aF_aM_aF	0/1;0/0;0/1;0/0;0/0
chr7	132507891	132507891	C	A	PLXNA4	exonic	nonsynonymous SNV	PLXNA4:NM_001105543:exon2:c.G803T:p.G268V,PLXNA4:NM_020911:exon2:c.G803T:p.G268V,PLXNA4:NM_181775:exon3:c.G803T:p.G268V	.	1.685	.	6.56E-05	1.40E-05	Fam109_f_m_aM	0/0;0/1;0/1
chr7	134891621	134891621	A	G	CALD1	exonic	startloss	CALD1:NM_033139:exon1:c.A1G:p.M1?,CALD1:NM_033140:exon1:c.A1G:p.M1?	0.498	.	.	3.39E-05	.	Fam14_f_m_aM_aM	0/0;0/1;0/0;0/1
chr7	138460570	138460570	G	C	TRIM24	exonic	nonsynonymous SNV	TRIM24:NM_003852:exon1:c.G22C:p.A8P,TRIM24:NM_015905:exon1:c.G22C:p.A8P	.	1.934	.	.	.	Fam113_f_m_aF_raM_uF	0/0;0/0;0/0;0/1;0/0
chr7	139109084	139109084	T	C	ZC3HAV1	exonic	nonsynonymous SNV	ZC3HAV1:NM_001363491:exon1:c.A248G:p.D83G,ZC3HAV1:NM_020119:exon1:c.A248G:p.D83G,ZC3HAV1:NM_024625:exon1:c.A248G:p.D83G	.	2.21	.	0.0002	4.19E-05	Fam43_f_m_aM	0/0;0/1;0/1
chr7	143268091	143268091	G	A	GSTK1	exonic	nonsynonymous SNV	GSTK1:NM_001143679:exon6:c.G706A:p.A236T,GSTK1:NM_001143680:exon6:c.G502A:p.A168T,GSTK1:NM_001143681:exon6:c.G409A:p.A137T,GSTK1:NM_015917:exon7:c.G538A:p.A180T	.	1.024	7.34E-05	3.37E-05	1.40E-05	Fam84_f_m_aF	0/0;0/1;0/1
chr7	143300046	143300046	C	T	CASP2	exonic	nonsynonymous SNV	CASP2:NM_001224:exon7:c.C778T:p.L260F,CASP2:NM_032982:exon7:c.C871T:p.L291F	.	1.225	.	4.47E-05	6.98E-06	Fam54_f_m_aM_uF	0/1;0/0;0/1;0/1
chr7	143304745	143304745	C	T	CASP2	exonic	nonsynonymous SNV	CASP2:NM_032982:exon10:c.C1189T:p.R397W	.	1.105	.	2.23E-05	6.98E-06	Fam62_f_m_aM_aM	0/1;0/0;0/1;0/0
chr7	149179548	149179548	G	A	ZNF398	exonic	nonsynonymous SNV	ZNF398:NM_170686:exon6:c.G1676A:p.R559H,ZNF398:NM_020781:exon7:c.G1163A:p.R388H	.	1.138	.	.	6.98E-06	Fam61_f_m_aM	0/0;0/1;0/1
chr7	150720500	150720500	G	A	GIMAP1	exonic	nonsynonymous SNV	GIMAP1:NM_130759:exon3:c.G496A:p.V166M	.	1.576	.	0.0001	2.79E-05	Fam15_f_m_aM_aM	0/1;0/0;0/1;0/1
chr7	150952640	150952640	C	T	KCNH2	exonic	nonsynonymous SNV	KCNH2:NM_001204798:exon2:c.G322A:p.A108T,KCNH2:NM_172057:exon2:c.G322A:p.A108T,KCNH2:NM_000238:exon6:c.G1342A:p.A448T,KCNH2:NM_172056:exon6:c.G1342A:p.A448T	.	1.248	.	.	6.98E-06	Fam47_f_m_aM	0/0;0/1;0/1
chr7	151071827	151071827	C	A	SLC4A2	exonic	nonsynonymous SNV	SLC4A2:NM_001199693:exon14:c.C2303A:p.A768D,SLC4A2:NM_001199694:exon14:c.C2288A:p.A763D,SLC4A2:NM_001199692:exon15:c.C2330A:p.A777D,SLC4A2:NM_003040:exon15:c.C2330A:p.A777D	.	1.741	.	.	.	Fam66_f_m_aM	0/1;0/0;0/1
chr7	151072027	151072027	C	A	SLC4A2	exonic	nonsynonymous SNV	SLC4A2:NM_001199693:exon15:c.C2399A:p.A800D,SLC4A2:NM_001199694:exon15:c.C2384A:p.A795D,SLC4A2:NM_001199692:exon16:c.C2426A:p.A809D,SLC4A2:NM_003040:exon16:c.C2426A:p.A809D	.	1.736	.	1.12E-05	6.98E-06	Fam81_f_m_aM_uM	0/1;0/0;0/1;0/1
chr7	151074456	151074456	G	A	SLC4A2	exonic	nonsynonymous SNV	SLC4A2:NM_001199693:exon17:c.G2821A:p.V941M,SLC4A2:NM_001199694:exon17:c.G2806A:p.V936M,SLC4A2:NM_001199692:exon18:c.G2848A:p.V950M,SLC4A2:NM_003040:exon18:c.G2848A:p.V950M	.	1.087	.	.	.	Fam35_f_m_aF_uM	0/0;0/1;0/1;0/1
chr7	151076026	151076026	G	A	SLC4A2	exonic	nonsynonymous SNV	SLC4A2:NM_001199693:exon21:c.G3458A:p.R1153H,SLC4A2:NM_001199694:exon21:c.G3443A:p.R1148H,SLC4A2:NM_001199692:exon22:c.G3485A:p.R1162H,SLC4A2:NM_003040:exon22:c.G3485A:p.R1162H	.	1.584	.	6.70E-05	1.40E-05	Fam56_f_m_aF_aM	0/1;0/0;0/1;0/1
chr7	151117721	151117721	G	A	AGAP3	exonic	nonsynonymous SNV	AGAP3:NM_001042535:exon5:c.G650A:p.R217H,AGAP3:NM_001308304:exon5:c.G650A:p.R217H,AGAP3:NM_001308305:exon5:c.G1190A:p.R397H,AGAP3:NM_001350102:exon5:c.G650A:p.R217H,AGAP3:NM_031946:exon5:c.G650A:p.R217H	.	2.812	.	.	6.98E-06	Fam17_f_m_aM_uM	0/0;0/1;0/1;0/1

chr7	151123798	151123798	G	A	AGAP3	exonic	nonsynonymous SNV	AGAP3:NM_001281300:exon9:c.G449A:p.R150Q,AGAP3:NM_001350102:exon9:c.G1133A:p.R378Q,AGAP3:NM_031946:exon9:c.G1133A:p.R378Q,AGAP3:NM_001350103:exon10:c.G95A:p.R32Q,AGAP3:NM_001350104:exon10:c.G95A:p.R32Q	.	1.015	.	1.13E-05	.	Fam34_f_m_aM_uF	0/1;0/0;0/1;0/0
chr7	151215678	151215678	A	C	ABCF2;LOC11448	exonic	nonsynonymous SNV	LOC114483834:NM_005692:exon13:c.T1456G:p.C486G,ABCF2:NM_007189:exon13:c.T1456G:p.C486G	.	1.175	.	1.12E-05	.	Fam108_f_m_aM	0/1;0/0;0/1
chr7	151239479	151239479	C	T	SMARCD3	exonic	nonsynonymous SNV	SMARCD3:NM_001003801:exon12:c.G1315A:p.G439S,SMARCD3:NM_001003802:exon13:c.G1276A:p.G426S,SMARCD3:NM_003078:exon13:c.G1276A:p.G426S	.	1.628	0.0001	0.0003	0.0001	Fam112_f_m_aM	0/0;0/1;0/1
chr7	151375875	151375875	T	A	NUB1	exonic	nonsynonymous SNV	NUB1:NM_001243351:exon13:c.T1495A:p.W499R,NUB1:NM_001363529:exon13:c.T1423A:p.W475R,NUB1:NM_016118:exon13:c.T1453A:p.W485R	.	1.003	.	1.12E-05	.	Fam68_f_m_aF_uF_uM	0/1;0/0;0/1;0/1;0/0
chr7	152144819	152144819	A	T	KMT2C	exonic	nonsynonymous SNV	KMT2C:NM_170606:exon55:c.T14237A:p.L4746Q	.	2.385	.	.	.	Fam34_f_m_aM_uF	0/0;0/1;0/1;0/0
chr7	154540623	154540623	A	T	DPP6	exonic	nonsynonymous SNV	DPP6:NM_001039350:exon4:c.A357T:p.K119N,DPP6:NM_001290252:exon4:c.A363T:p.K121N,DPP6:NM_001290253:exon4:c.A549T:p.K183N,DPP6:NM_001364501:exon4:c.A357T:p.K119N,DPP6:NM_001364502:exon4:c.A363T:p.K121N,DPP6:NM_001936:exon4:c.A363T:p.K121N,DPP6:NM_130797:exon4:c.A549T:p.K183N,DPP6:NM_001364497:exon5:c.A366T:p.K122N,DPP6:NM_001364498:exon5:c.A366T:p.K122N,DPP6:NM_001364499:exon5:c.A366T:p.K122N,DPP6:NM_001364500:exon5:c.A366T:p.K122N	.	1.303	.	1.28E-05	6.98E-06	Fam14_f_m_aM_aM	0/1;0/0;0/1;0/0
chr7	155071216	155071216	G	A	HTR5A	exonic	nonsynonymous SNV	HTR5A:NM_024012:exon1:c.G317A:p.R106H	.	1.236	.	3.44E-05	.	Fam12_f_m_aM_uM_aM	0/0;0/1;0/1;0/0;0/0
chr7	155298571	155298571	G	T	INSIG1	exonic	nonsynonymous SNV	INSIG1:NM_001346590:exon2:c.G286T:p.V96L,INSIG1:NM_001346591:exon2:c.G286T:p.V96L,INSIG1:NM_001346592:exon2:c.G286T:p.V96L,INSIG1:NM_001346593:exon2:c.G286T:p.V96L,INSIG1:NM_001346594:exon2:c.G286T:p.V96L,INSIG1:NM_005542:exon2:c.G286T:p.V96L,INSIG1:NM_198337:exon2:c.G286T:p.V96L	.	1.389	.	1.17E-05	.	Fam16_f_m_aM_aM	0/1;0/0;0/0;0/1
chr7	158658401	158658401	C	T	NCAPG2	exonic	nonsynonymous SNV	NCAPG2:NM_001281933:exon17:c.G1997A:p.R666H,NCAPG2:NM_017760:exon17:c.G1997A:p.R666H,NCAPG2:NM_001281932:exon18:c.G1997A:p.R666H	.	1.315	7.34E-05	6.82E-05	1.40E-05	Fam17_f_m_aM_uM	0/1;0/0;0/1;0/1
chr8	2001331	2001331	G	A	KBTBD11	exonic	nonsynonymous SNV	KBTBD11:NM_014867:exon2:c.G139A:p.E47K	.	1.181	.	.	.	Fam70_f_m_aM	0/0;0/1;0/1
chr8	2002408	2002408	C	T	KBTBD11	exonic	nonsynonymous SNV	KBTBD11:NM_014867:exon2:c.C1216T:p.R406W	.	2.143	.	.	.	Fam54_f_m_aM_uF	0/1;0/0;0/1;0/1
chr8	9751655	9751655	C	T	TNKS	exonic	nonsynonymous SNV	TNKS:NM_003747:exon19:c.C2879T:p.A960V	.	1.148	.	2.23E-05	2.09E-05	Fam84_f_m_aF	0/1;0/0;0/1
chr8	11201184	11201184	G	C	XKR6	exonic	nonsynonymous SNV	XKR6:NM_173683:exon1:c.C156G:p.S52R	.	1.695	.	.	.	Fam64_f_m_aM	0/1;0/0;0/1
chr8	20250006	20250006	C	G	LZTS1	exonic	nonsynonymous SNV	LZTS1:NM_001362884:exon4:c.G1507C:p.E503Q,LZTS1:NM_021020:exon4:c.G1507C:p.E503Q	.	1.121	.	2.30E-05	6.98E-06	Fam44_f_m_aM_uF	0/0;0/1;0/1;0/0
chr8	22069916	22069916	C	T	DMTN	exonic	nonsynonymous SNV	DMTN:NM_001323400:exon4:c.C310T:p.P104S,DMTN:NM_001302817:exon5:c.C310T:p.P104S,DMTN:NM_001323388:exon5:c.C310T:p.P104S,DMTN:NM_001323398:exon5:c.C310T:p.P104S,DMTN:NM_001323399:exon5:c.C310T:p.P104S,DMTN:NM_001323401:exon5:c.C310T:p.P104S,DMTN:NM_001114139:exon6:c.C355T:p.P119S,DMTN:NM_001323381:exon6:c.C430T:p.P144S,DMTN:NM_001323385:exon6:c.C430T:p.P144S,DMTN:NM_001323387:exon6:c.C355T:p.P119S,DMTN:NM_001323389:exon6:c.C385T:p.P129S,DMTN:NM_001323390:exon6:c.C385T:p.P129S,DMTN:NM_001323391:exon6:c.C355T:p.P119S,DMTN:NM_001323392:exon6:c.C355T:p.P119S,DMTN:NM_001323393:exon6:c.C355T:p.P119S,DMTN:NM_001323394:exon6:c.C355T:p.P119S,DMTN:NM_001323395:exon6:c.C355T:p.P119S,DMTN:NM_001323396:exon6:c.C355T:p.P119S,DMTN:NM_001323397:exon6:c.C355T:p.P119S,DMTN:NM_001114135:exon7:c.C430T:p.P144S,DMTN:NM_001114136:exon7:c.C430T:p.P144S,DMTN:NM_001114137:exon7:c.C430T:p.P144S,DMTN:NM_001114138:exon7:c.C430T:p.P144S,DMTN:NM_001302816:exon7:c.C430T:p.P144S,DMTN:NM_001323378:exon7:c.C430T:p.P144S,DMTN:NM_001323379:exon7:c.C430T:p.P144S,DMTN:NM_001323380:exon7:c.C430T:p.P144S,DMTN:NM_001323382:exon7:c.C430T:p.P144S,DMTN:NM_001323383:exon7:c.C430T:p.P144S,DMTN:NM_001323384:exon7:c.C430T:p.P144S,DMTN:NM_001978:exon7:c.C430T:p.P144S	.	1.031	.	.	.	Fam116_f_m_aM_aF	0/0;0/1;0/1;0/0
chr8	22221541	22221541	C	T	PHYHIP	exonic	nonsynonymous SNV	PHYHIP:NM_001363312:exon5:c.G805A:p.V269M,PHYHIP:NM_014759:exon5:c.G805A:p.V269M,PHYHIP:NM_001099335:exon6:c.G805A:p.V269M,PHYHIP:NM_001363311:exon6:c.G805A:p.V269M	.	1.217	.	2.26E-05	6.98E-06	Fam44_f_m_aM_uF	0/1;0/0;0/1;0/0

chr8	22415856	22415856	G	A	SLC39A14	exonic	nonsynonymous SNV	SLC39A14:NM_001128431:exon6:c.G838A:p.G280R,SLC39A14:NM_001135153:exon6:c.G838A:p.G280R,SLC39A14:NM_001135154:exon6:c.G838A:p.G280R,SLC39A14:NM_001351659:exon6:c.G868A:p.G290R,SLC39A14:NM_001351660:exon6:c.G838A:p.G280R,SLC39A14:NM_015359:exon6:c.G838A:p.G280R,SLC39A14:NM_001351655:exon7:c.G838A:p.G280R,SLC39A14:NM_001351658:exon7:c.G868A:p.G290R,SLC39A14:NM_001351656:exon8:c.G838A:p.G280R,SLC39A14:NM_001351657:exon8:c.G868A:p.G290R	.	1.653	0.0004	0.0004	0.0001	Fam69_f_m_aM	0/1;0/0;0/1
chr8	23010577	23010577	G	A	RHOBTB2	exonic	nonsynonymous SNV	RHOBTB2:NM_001160037:exon7:c.G1681A:p.V561M,RHOBTB2:NM_015178:exon7:c.G1660A:p.V554M,RHOBTB2:NM_001160036:exon9:c.G1726A:p.V576M	.	1.312	7.35E-05	4.47E-05	2.10E-05	Fam102_f_m_aM	0/0;0/1;0/1
chr8	26627903	26627903	C	T	DPYSL2	exonic	nonsynonymous SNV	DPYSL2:NM_001197293:exon7:c.C968T:p.T323M,DPYSL2:NM_001244604:exon7:c.C545T:p.T182M,DPYSL2:NM_001386:exon7:c.C653T:p.T218M	.	1.97	.	7.46E-05	2.79E-05	Fam52_f_m_aM	0/1;0/0;0/1
chr8	27605244	27605244	G	A	CLU	exonic	nonsynonymous SNV	CLU:NM_001831:exon5:c.C509T:p.T170M	.	1.042	.	1.12E-05	1.40E-05	Fam56_f_m_aF_aM	0/1;0/0;0/1;0/0
chr8	27907159	27907159	C	T	SCARA5	exonic	nonsynonymous SNV	SCARA5:NM_173833:exon6:c.G1085A:p.R362H	.	1.023	7.35E-05	0.0008	8.38E-05	Fam19_f_m_aM	0/0;0/1;0/1
chr8	28106778	28106778	A	G	ELP3	exonic	nonsynonymous SNV	ELP3:NM_001284220:exon3:c.A108G:p.I36M,ELP3:NM_001284224:exon3:c.A31G:p.M11V,ELP3:NM_001284225:exon3:c.A31G:p.M11V,ELP3:NM_001284226:exon3:c.A48G:p.I16M,ELP3:NM_001284222:exon4:c.A282G:p.I94M,ELP3:NM_018091:exon4:c.A324G:p.I108M	.	1.122	.	.	6.98E-06	Fam7_f_m_aM_aM_uM	0/0;0/1;0/0;0/1;0/1
chr8	28846766	28846766	G	A	INTS9	exonic	nonsynonymous SNV	INTS9:NM_001172562:exon4:c.C170T:p.P57L,INTS9:NM_001363038:exon4:c.C242T:p.P81L,INTS9:NM_018250:exon4:c.C242T:p.P81L	.	1.11	0.0001	0.0001	7.69E-05	Fam48_f_m_aM_uM	0/0;0/1;0/1;0/0
chr8	29140073	29140073	A	G	KIF13B	exonic	nonsynonymous SNV	KIF13B:NM_015254:exon21:c.T2603C:p.L868P	.	1.007	0.0003	.	6.28E-05	Fam46_f_m_aM_uM	0/0;0/1;0/1;0/0
chr8	30693039	30693039	T	C	GSR	exonic	nonsynonymous SNV	GSR:NM_000637:exon8:c.A812G:p.D271G,GSR:NM_001195103:exon8:c.A812G:p.D271G	.	1.161	.	.	.	Fam16_f_m_aM_aM	0/1;0/0;0/0;0/1
chr8	32754450	32754450	G	A	NRG1	exonic	nonsynonymous SNV	NRG1:NM_001159996:exon4:c.G317A:p.C106Y,NRG1:NM_001322197:exon4:c.G308A:p.C103Y,NRG1:NM_001322202:exon4:c.G5A:p.C2Y,NRG1:NM_001322201:exon5:c.G5A:p.C2Y,NRG1:NM_001322205:exon5:c.G959A:p.C320Y,NRG1:NM_001322206:exon5:c.G959A:p.C320Y,NRG1:NM_001322207:exon5:c.G959A:p.C320Y,NRG1:NM_001160004:exon8:c.G770A:p.C257Y,NRG1:NM_001160008:exon8:c.G770A:p.C257Y,NRG1:NM_013957:exon8:c.G770A:p.C257Y,NRG1:NM_013960:exon8:c.G779A:p.C260Y,NRG1:NM_013964:exon8:c.G779A:p.C260Y,NRG1:NM_013956:exon9:c.G794A:p.C265Y	.	1.089	.	.	.	Fam103_f_m_aM	0/1;0/0;0/1
chr8	33597491	33597491	C	A	DUSP26	exonic	nonsynonymous SNV	DUSP26:NM_001305116:exon1:c.G25T:p.A9S,DUSP26:NM_001305115:exon2:c.G25T:p.A9S,DUSP26:NM_024025:exon2:c.G25T:p.A9S	.	1.272	0.0002	0.0002	0.0002	Fam83_f_m_aF	0/0;0/1;0/1
chr8	33597491	33597491	C	A	DUSP26	exonic	nonsynonymous SNV	DUSP26:NM_001305116:exon1:c.G25T:p.A9S,DUSP26:NM_001305115:exon2:c.G25T:p.A9S,DUSP26:NM_024025:exon2:c.G25T:p.A9S	.	1.272	0.0002	0.0002	0.0002	Fam99_f_m_aM_aM	0/0;0/1;0/1;0/1
chr8	38138971	38138971	A	G	ASH2L	exonic	nonsynonymous SNV	ASH2L:NM_001261832:exon15:c.A1406G:p.D469G,ASH2L:NM_001282272:exon15:c.A1370G:p.D457G,ASH2L:NM_001105214:exon16:c.A1505G:p.D502G,ASH2L:NM_004674:exon16:c.A1787G:p.D596G	.	1.336	.	.	.	Fam45_f_m_aM_uF	0/0;0/1;0/1;0/1
chr8	38337353	38337353	C	T	NSD3	exonic	nonsynonymous SNV	NSD3:NM_017778:exon4:c.G862A:p.V288I,NSD3:NM_023034:exon4:c.G862A:p.V288I	.	1.014	.	1.12E-05	1.40E-05	Fam115_f_m_aF_aM_aF	0/1;0/0;0/1;0/0;0/0
chr8	38348157	38348157	G	C	NSD3	exonic	nonsynonymous SNV	NSD3:NM_017778:exon2:c.C15G:p.F5L,NSD3:NM_023034:exon2:c.C15G:p.F5L	.	1.398	.	.	.	Fam61_f_m_aM	0/1;0/0;0/1
chr8	41693943	41693943	C	G	ANK1	exonic	nonsynonymous SNV	ANK1:NM_000037:exon29:c.G3487C:p.G1163R,ANK1:NM_020475:exon29:c.G3487C:p.G1163R,ANK1:NM_020476:exon29:c.G3487C:p.G1163R,ANK1:NM_020477:exon29:c.G3487C:p.G1163R,ANK1:NM_001142446:exon30:c.G3610C:p.G1204R	.	1.101	.	.	.	Fam43_f_m_aM	0/0;0/1;0/1
chr8	41704042	41704042	G	C	ANK1	exonic	nonsynonymous SNV	ANK1:NM_000037:exon20:c.C2294G:p.S765W,ANK1:NM_001142446:exon20:c.C2393G:p.S798W,ANK1:NM_020475:exon20:c.C2294G:p.S765W,ANK1:NM_020476:exon20:c.C2294G:p.S765W,ANK1:NM_020477:exon20:c.C2294G:p.S765W	.	1.122	.	.	.	Fam1_f_m_aM	0/1;0/0;0/1
chr8	41980860	41980860	G	A	KAT6A	exonic	nonsynonymous SNV	KAT6A:NM_001305878:exon5:c.C893T:p.T298I,KAT6A:NM_006766:exon5:c.C893T:p.T298I	.	1.279	7.34E-05	4.47E-05	2.09E-05	Fam66_f_m_aM	0/0;0/1;0/1
chr8	42154791	42154791	C	G	AP3M2	exonic	nonsynonymous SNV	AP3M2:NM_006803:exon2:c.C104G:p.A35G,AP3M2:NM_001134296:exon3:c.C104G:p.A35G	.	1.19	0.0004	0.0006	0.0003	Fam79_f_m_aM_uM	0/0;0/1;0/1;0/0
chr8	42417936	42417936	C	T	SLC20A2	exonic	nonsynonymous SNV	SLC20A2:NM_001257180:exon11:c.G1826A:p.R609H,SLC20A2:NM_001257181:exon11:c.G1826A:p.R609H,SLC20A2:NM_006749:exon11:c.G1826A:p.R609H	.	1.797	.	6.54E-05	.	Fam116_f_m_aM_aF	0/1;0/0;0/0;0/1
chr8	52940384	52940384	C	A	NPBWR1	exonic	nonsynonymous SNV	NPBWR1:NM_005285:exon1:c.C477A:p.S159R	.	1.226	7.35E-05	3.32E-05	1.40E-05	Fam1_f_m_aM	0/1;0/0;0/1
chr8	52940717	52940717	C	A	NPBWR1	exonic	nonsynonymous SNV	NPBWR1:NM_005285:exon1:c.C810A:p.S270R	.	1.231	.	4.49E-05	1.40E-05	Fam26_f_m_aM_uM	0/0;0/1;0/1;0/1
chr8	54458221	54458221	G	T	SOX17	exonic	nonsynonymous SNV	SOX17:NM_022454:exon1:c.G83T:p.G28V	.	2.12	0.0006	0.0002	0.0001	Fam38_f_m_aM	0/0;0/1;0/1
chr8	56966313	56966313	C	T	IMPAD1	exonic	nonsynonymous SNV	IMPAD1:NM_017813:exon4:c.G686A:p.R229H	.	1.235	.	7.45E-05	4.19E-05	Fam46_f_m_aM_uM	0/0;0/1;0/1;0/1

chr8	63187209	63187209	G	T	YTHDF3	exonic	nonsynonymous SNV	YTHDF3:NM_001277816:exon3:c.G1045T:p.A349S,YTHDF3:NM_001277817:exon4:c.G1045T:p.A349S,YTHDF3:NM_001277818:exon4:c.G1045T:p.A349S,YTHDF3:NM_152758:exon4:c.G1198T:p.A400S,YTHDF3:NM_001277813:exon5:c.G1207T:p.A403S,YTHDF3:NM_001277814:exon5:c.G1207T:p.A403S,YTHDF3:NM_001277815:exon5:c.G1045T:p.A349S	.	1.003	.	1.13E-05	.	Fam9_f_m_aM_dM_uF	0/1;0/0;0/1;0/1;0/0
chr8	66597425	66597425	T	G	MYBL1	exonic	nonsynonymous SNV	MYBL1:NM_001080416:exon5:c.A417C:p.K139N,MYBL1:NM_001144755:exon5:c.A417C:p.K139N,MYBL1:NM_001294282:exon5:c.A417C:p.K139N	.	2.352	.	.	.	Fam110_f_m_aM_aM_uMs	0/0;0/1;0/1;0/0;0/0
chr8	67240203	67240203	T	C	ARFGEF1	exonic	nonsynonymous SNV	ARFGEF1:NM_006421:exon20:c.A2938G:p.I980V	.	1.402	.	.	.	Fam29_f_m_aF_uM	0/0;0/1;0/1;0/1
chr8	68055950	68055950	C	T	PREX2	exonic	nonsynonymous SNV	PREX2:NM_024870:exon10:c.C1214T:p.T405M,PREX2:NM_025170:exon10:c.C1214T:p.T405M	.	1.328	.	0.0002	0.0002	Fam48_f_m_aM_uM	0/0;0/1;0/1;0/0
chr8	68069862	68069862	A	G	PREX2	exonic	nonsynonymous SNV	PREX2:NM_024870:exon13:c.A1471G:p.S491G,PREX2:NM_025170:exon13:c.A1471G:p.S491G	.	1.257	.	.	.	Fam5_f_m_aM	0/1;0/0;0/1
chr8	72568050	72568050	G	A	KCNB2	exonic	nonsynonymous SNV	KCNB2:NM_004770:exon2:c.G316A:p.G106R	.	1.661	7.35E-05	0.0002	2.10E-05	Fam103_f_m_aM	0/1;0/0;0/1
chr8	80486893	80486893	A	G	ZBTB10	exonic	nonsynonymous SNV	ZBTB10:NM_001105539:exon1:c.A83G:p.N28S,ZBTB10:NM_023929:exon1:c.A83G:p.N28S	.	1.417	.	3.10E-05	7.04E-06	Fam95_f_m_aM_aM_uF	0/1;0/0;0/1;0/1;0/0
chr8	84529316	84529316	C	A	RALYL	exonic	nonsynonymous SNV	RALYL:NM_001100391:exon2:c.C34A:p.P12T,RALYL:NM_001354321:exon2:c.C34A:p.P12T,RALYL:NM_001354322:exon2:c.C34A:p.P12T,RALYL:NM_001354323:exon2:c.C34A:p.P12T	.	1.09	0.0009	0.0004	0.0003	Fam69_f_m_aM	0/0;0/1;0/1
chr8	84529428	84529428	T	C	RALYL	exonic	nonsynonymous SNV	RALYL:NM_001100391:exon2:c.T146C:p.V49A,RALYL:NM_001100393:exon2:c.T107C:p.V36A,RALYL:NM_001287243:exon2:c.T107C:p.V36A,RALYL:NM_001354311:exon2:c.T107C:p.V36A,RALYL:NM_001354313:exon2:c.T107C:p.V36A,RALYL:NM_001354314:exon2:c.T107C:p.V36A,RALYL:NM_001354315:exon2:c.T107C:p.V36A,RALYL:NM_001354321:exon2:c.T146C:p.V49A,RALYL:NM_001354322:exon2:c.T146C:p.V49A,RALYL:NM_001354323:exon2:c.T146C:p.V49A,RALYL:NM_001354325:exon2:c.T107C:p.V36A,RALYL:NM_173848:exon2:c.T107C:p.V36A,RALYL:NM_001100392:exon3:c.T107C:p.V36A,RALYL:NM_001354307:exon3:c.T107C:p.V36A,RALYL:NM_001354308:exon3:c.T107C:p.V36A,RALYL:NM_001354312:exon3:c.T107C:p.V36A,RALYL:NM_001354316:exon3:c.T107C:p.V36A,RALYL:NM_001354317:exon3:c.T107C:p.V36A,RALYL:NM_001354318:exon3:c.T107C:p.V36A,RALYL:NM_001354319:exon3:c.T107C:p.V36A,RALYL:NM_001354320:exon3:c.T107C:p.V36A,RALYL:NM_001354305:exon4:c.T107C:p.V36A,RALYL:NM_001354306:exon4:c.T107C:p.V36A,RALYL:NM_001354309:exon4:c.T107C:p.V36A	.	1.125	0.0009	0.0009	0.0006	Fam17_f_m_aM_uM	0/0;0/1;0/1;0/0
chr8	84529428	84529428	T	A	RALYL	exonic	nonsynonymous SNV	RALYL:NM_001100391:exon2:c.T146A:p.V49D,RALYL:NM_001100393:exon2:c.T107A:p.V36D,RALYL:NM_001287243:exon2:c.T107A:p.V36D,RALYL:NM_001354311:exon2:c.T107A:p.V36D,RALYL:NM_001354313:exon2:c.T107A:p.V36D,RALYL:NM_001354314:exon2:c.T107A:p.V36D,RALYL:NM_001354315:exon2:c.T107A:p.V36D,RALYL:NM_001354321:exon2:c.T146A:p.V49D,RALYL:NM_001354322:exon2:c.T146A:p.V49D,RALYL:NM_001354323:exon2:c.T146A:p.V49D,RALYL:NM_001354325:exon2:c.T107A:p.V36D,RALYL:NM_173848:exon2:c.T107A:p.V36D,RALYL:NM_001100392:exon3:c.T107A:p.V36D,RALYL:NM_001354307:exon3:c.T107A:p.V36D,RALYL:NM_001354308:exon3:c.T107A:p.V36D,RALYL:NM_001354312:exon3:c.T107A:p.V36D,RALYL:NM_001354316:exon3:c.T107A:p.V36D,RALYL:NM_001354317:exon3:c.T107A:p.V36D,RALYL:NM_001354318:exon3:c.T107A:p.V36D,RALYL:NM_001354319:exon3:c.T107A:p.V36D,RALYL:NM_001354320:exon3:c.T107A:p.V36D,RALYL:NM_001354305:exon4:c.T107A:p.V36D,RALYL:NM_001354306:exon4:c.T107A:p.V36D,RALYL:NM_001354309:exon4:c.T107A:p.V36D	.	1.464	7.34E-05	0.0003	5.58E-05	Fam34_f_m_aM_uF	0/0;0/1;0/1;0/0
chr8	86448422	86448422	A	T	WWP1	exonic	nonsynonymous SNV	WWP1:NM_007013:exon20:c.A2182T:p.M728L	.	1.058	.	.	.	Fam52_f_m_aM	0/1;0/0;0/1
chr8	89769803	89769803	T	C	RIPK2	exonic	nonsynonymous SNV	RIPK2:NM_003821:exon4:c.T515C:p.M172T	.	1.105	7.37E-05	0.0001	7.69E-05	Fam7_f_m_aM_aM_uM	0/1;0/0;0/1;0/0;0/0
chr8	89769803	89769803	T	C	RIPK2	exonic	nonsynonymous SNV	RIPK2:NM_003821:exon4:c.T515C:p.M172T	.	1.105	7.37E-05	0.0001	7.69E-05	Fam77_f_m_aM_aF_uM_uFs	0/1;0/0;0/1;0/0;0/0;0/0
chr8	90060678	90060678	T	C	CALB1	exonic	nonsynonymous SNV	CALB1:NM_001366795:exon9:c.A548G:p.E183G,CALB1:NM_004929:exon10:c.A623G:p.E208G	.	1.601	.	1.12E-05	.	Fam56_f_m_aF_aM	0/1;0/0;0/0;0/1
chr8	90645482	90645482	G	T	TMEM64	exonic	nonsynonymous SNV	TMEM64:NM_001008495:exon1:c.C424A:p.R142S,TMEM64:NM_001146273:exon1:c.C424A:p.R142S	.	2.009	.	0.0002	.	Fam116_f_m_aM_aF	0/1;0/0;0/1;0/0
chr8	90925596	90925596	C	G	NECAB1	exonic	nonsynonymous SNV	NECAB1:NM_022351:exon7:c.C556G:p.R186G	.	1.362	.	3.28E-05	2.79E-05	Fam7_f_m_aM_aM_uM	0/1;0/0;0/1;0/0;0/0

chr8	92014815	92014815	T	C	RUNX1T1	exonic	nonsynonymous SNV	RUNX1T1:NM_175636:exon2:c.A121G:p.N41D,RUNX1T1:NM_001198633:exon3:c.A172G:p.N58D,RUNX1T1:NM_001198634:exon3:c.A265G:p.N89D,RUNX1T1:NM_004349:exon3:c.A151G:p.N51D,RUNX1T1:NM_175635:exon3:c.A121G:p.N41D,RUNX1T1:NM_001198628:exon4:c.A232G:p.N78D,RUNX1T1:NM_001198629:exon4:c.A232G:p.N78D,RUNX1T1:NM_001198631:exon4:c.A232G:p.N78D,RUNX1T1:NM_001198632:exon4:c.A151G:p.N51D,RUNX1T1:NM_001198679:exon4:c.A409G:p.N137D,RUNX1T1:NM_175634:exon4:c.A232G:p.N78D,RUNX1T1:NM_001198626:exon5:c.A232G:p.N78D,RUNX1T1:NM_001198630:exon5:c.A232G:p.N78D,RUNX1T1:NM_001198627:exon6:c.A232G:p.N78D,RUNX1T1:NM_001198625:exon7:c.A151G:p.N51D	.	1.233	.	.	.		Fam118_f_m_aM_aF_uM	0/0;0/1;0/0;0/1;0/0
chr8	94490022	94490022	C	T	VIRMA	exonic	nonsynonymous SNV	VIRMA:NM_015496:exon23:c.G5201A:p.R1734Q	.	1.377	.		7.46E-05	6.98E-06	Fam26_f_m_aM_uM	0/0;0/1;0/1;0/1
chr8	94492749	94492749	G	A	VIRMA	exonic	nonsynonymous SNV	VIRMA:NM_015496:exon21:c.C4711T:p.R1571C	.	1.128	.		0.0004	.	Fam29_f_m_aF_uM	0/0;0/1;0/1;0/0
chr8	96160371	96160371	C	T	GDF6	exonic	nonsynonymous SNV	GDF6:NM_001001557:exon1:c.G322A:p.A108T	.	1.599	.		0.0002	5.58E-05	Fam12_f_m_aM_uM_aM	0/0;0/1;0/1;0/0;0/1
chr8	96160421	96160421	C	T	GDF6	exonic	nonsynonymous SNV	GDF6:NM_001001557:exon1:c.G272A:p.R91H	.	2.102	.		1.13E-05	6.98E-06	Fam5_f_m_aM	0/1;0/0;0/1
chr8	96299719	96299719	A	G	PTDSS1	exonic	nonsynonymous SNV	PTDSS1:NM_001290225:exon4:c.A188G:p.N63S,PTDSS1:NM_014754:exon6:c.A626G:p.N209S	.	1.512	0.0003		0.0001	1.40E-05	Fam78_f_m_aF_uM	0/0;0/1;0/1;0/1
chr8	97706629	97706629	G	A	MTDH	exonic	nonsynonymous SNV	MTDH:NM_001363139:exon6:c.G986A:p.G329D,MTDH:NM_001363136:exon7:c.G1085A:p.G362D,MTDH:NM_001363138:exon7:c.G1052A:p.G351D,MTDH:NM_178812:exon8:c.G1151A:p.G384D,MTDH:NM_001363137:exon9:c.G1241A:p.G414D	.	1.212	7.34E-05		0.0002	2.09E-05	Fam109_f_m_aM	0/0;0/1;0/1
chr8	98044990	98044990	T	G	RPL30	exonic	nonsynonymous SNV	RPL30:NM_000989:exon3:c.A120C:p.Q40H	.	1.177	.	.	.		Fam82_f_m_aM_uF	0/1;0/0;0/1;0/1
chr8	100718215	100718215	A	C	PABPC1	exonic	nonsynonymous SNV	PABPC1:NM_002568:exon2:c.T259G:p.S87A	.	2.642	.	.	.		Fam92_f_m_aM_raM	0/1;0/0;0/0;0/1
chr8	102265260	102265260	G	-	UBR5	exonic	frameshift deletion	UBR5:NM_001282873:exon53:c.7438delC:p.H2480Mfs*7, UBR5:NM_015902:exon53:c.7441delC:p.H2481Mfs*7	0.062	.	.	.	.		Fam110_f_m_aM_aM_uMs	0/0;0/0;0/1;0/0;0/0
chr8	102360134	102360134	C	T	UBR5	exonic	nonsynonymous SNV	UBR5:NM_001282873:exon5:c.G323A:p.S108N,UBR5:NM_015902:exon5:c.G323A:p.S108N	.	1.398	.	.	.		Fam120_f_m_aM_aM	0/1;0/0;0/1;0/1
chr8	103927883	103927891	GGTT	-	RIMS2	exonic	stopgain	RIMS2:NM_001348504:exon9:c.1662_1668del:p.V555*,RIMS2:NM_001348507:exon9:c.1662_1668del:p.V555*,RIMS2:NM_001100117:exon11:c.2238_2244del:p.V747*	0.408	.	.	.	.		Fam37_f_m_aF_uM	0/1;0/0;0/1;0/1
chr8	107303302	107303302	C	T	ANGPT1	exonic	nonsynonymous SNV	ANGPT1:NM_001314051:exon4:c.G274A:p.A92T,ANGPT1:NM_001146:exon5:c.G874A:p.A292T,ANGPT1:NM_001199859:exon5:c.G871A:p.A291T	.	1.565	.	.	.		Fam71_f_m_aF	0/1;0/0;0/1
chr8	117521031	117521031	T	A	MED30	exonic	nonsynonymous SNV	MED30:NM_001282986:exon1:c.T155A:p.F52Y,MED30:NM_001363182:exon1:c.T155A:p.F52Y,MED30:NM_080651:exon1:c.T155A:p.F52Y	.	1.568	7.35E-05		9.83E-05	6.98E-06	Fam75_f_m_aM	0/1;0/0;0/1
chr8	119746847	119746847	A	C	TAF2	exonic	nonsynonymous SNV	TAF2:NM_003184:exon23:c.T2966G:p.L989W	.	1.578	.		2.23E-05	1.40E-05	Fam86_f_m_aF	0/0;0/1;0/1
chr8	124372078	124372078	C	A	TMEM65	exonic	nonsynonymous SNV	TMEM65:NM_194291:exon1:c.G80T:p.R27L	.	1.079	8.13E-05	.		6.52E-05	Fam44_f_m_aM_uF	0/0;0/1;0/1;0/1
chr8	125044559	125044559	A	C	WASHC5	exonic	nonsynonymous SNV	WASHC5:NM_001330609:exon20:c.T2200G:p.F734V,WASHC5:NM_014846:exon21:c.T2644G:p.F882V	.	1.015	7.34E-05		0.0006	1.40E-05	Fam105_f_m_aM	0/0;0/1;0/1
chr8	125436358	125436358	C	T	TRIB1	exonic	nonsynonymous SNV	TRIB1:NM_001282985:exon3:c.C508T:p.P170S,TRIB1:NM_025195:exon3:c.C1006T:p.P336S	.	1.209	.		4.47E-05	6.99E-06	Fam21_f_m_aM_uM	0/0;0/1;0/1;0/0
chr8	132140080	132140080	C	T	KCNQ3	exonic	nonsynonymous SNV	KCNQ3:NM_001204824:exon11:c.G1204A:p.V402I,KCNQ3:NM_004519:exon11:c.G1564A:p.V522I	.	1.414	.		0.0001	3.49E-05	Fam119_f_m_aM_aM	0/1;0/0;0/1;0/1
chr8	132845831	132845831	C	T	PHF20L1	exonic	nonsynonymous SNV	PHF20L1:NM_001277196:exon20:c.C2884T:p.L962F,PHF20L1:NM_001362971:exon20:c.C2884T:p.L962F,PHF20L1:NM_016018:exon21:c.C2962T:p.L988F	.	1.015	7.35E-05		0.0001	4.89E-05	Fam92_f_m_aM_raM	0/0;0/0;0/0;0/1
chr8	134602650	134602650	G	A	ZFAT	exonic	nonsynonymous SNV	ZFAT:NM_001174157:exon5:c.C883T:p.R295C,ZFAT:NM_001167583:exon6:c.C1033T:p.R345C,ZFAT:NM_001174158:exon6:c.C1033T:p.R345C,ZFAT:NM_020863:exon6:c.C1069T:p.R357C,ZFAT:NM_001029939:exon7:c.C1033T:p.R345C, ZFAT:NM_001289394:exon7:c.C1033T:p.R345C	.	1.064	0.0003		0.0003	0.0002	Fam70_f_m_aM	0/1;0/0;0/1
chr8	139618625	139618625	T	A	KCNK9	exonic	nonsynonymous SNV	KCNK9:NM_001282534:exon2:c.A758T:p.D253V	.	1.649	.	.	.		Fam107_f_m_aM	0/1;0/0;0/1
chr8	140287691	140287691	G	A	TRAPPC9	exonic	nonsynonymous SNV	TRAPPC9:NM_001321646:exon12:c.C1871T:p.A624V,TRAPPC9:NM_001160372:exon13:c.C1898T:p.A633V,TRAPPC9:NM_031466:exon13:c.C2192T:p.A731V	.	1.401	0.0002		0.0005	0.0003	Fam21_f_m_aM_uM	0/1;0/0;0/1;0/0
chr8	140458294	140458294	C	T	TRAPPC9	exonic	nonsynonymous SNV	TRAPPC9:NM_031466:exon1:c.G271A:p.A91T	.	1.085	0.0001		0.0002	8.38E-05	Fam80_f_m_aM_uM	0/0;0/1;0/1;0/0
chr8	140557231	140557231	G	A	AGO2	exonic	nonsynonymous SNV	AGO2:NM_001164623:exon8:c.C884T:p.P295L,AGO2:NM_012154:exon8:c.C884T:p.P295L	.	2.832	.	.	.		Fam84_f_m_aF	0/1;0/0;0/1
chr8	141175412	141175412	G	A	DENND3	exonic	nonsynonymous SNV	DENND3:NM_001352890:exon14:c.G2488A:p.E830K,DENND3:NM_001362798:exon14:c.G2488A:p.E830K,DENND3:NM_014957:exon14:c.G2287A:p.E763K	.	1.285	0.0003		3.35E-05	1.40E-05	Fam63_f_m_aF	0/0;0/1;0/1
chr8	141427767	141427767	C	T	PTP4A3	exonic	nonsynonymous SNV	PTP4A3:NM_032611:exon5:c.C347T:p.A116V	.	1.52	.		0.0001	3.49E-05	Fam100_f_m_aF	0/1;0/0;0/1
chr8	142484668	142484668	G	A	ADGRB1	exonic	nonsynonymous SNV	ADGRB1:NM_001702:exon13:c.G2212A:p.A738T	.	1.644	.		0.0002	2.79E-05	Fam75_f_m_aM	0/0;0/1;0/1
chr8	142537021	142537021	G	C	ADGRB1	exonic	nonsynonymous SNV	ADGRB1:NM_001702:exon26:c.G3605C:p.R1202P	.	1.463	.		0.0001	0.0001	Fam85_f_m_aM_aM	0/1;0/0;0/1;0/1
chr8	143598730	143598730	C	T	TIGD5	exonic	nonsynonymous SNV	TIGD5:NM_032862:exon1:c.C827T:p.T276M	.	1.965	.	.	.		Fam24_f_m_aM_aM	0/0;0/1;0/1;0/1
chr8	143598772	143598772	A	G	TIGD5	exonic	nonsynonymous SNV	TIGD5:NM_032862:exon1:c.A869G:p.K290R	.	1.721	.		3.47E-05	.	Fam92_f_m_aM_raM	0/1;0/0;0/0;0/1
chr8	143727903	143727903	G	A	FAM83H	exonic	nonsynonymous SNV	FAM83H:NM_198488:exon5:c.C1558T:p.R520C	.	1.261	7.43E-05	.	.		Fam92_f_m_aM_raM	0/1;0/0;0/1;0/0
chr8	143729085	143729085	G	A	FAM83H	exonic	nonsynonymous SNV	FAM83H:NM_198488:exon4:c.C619T:p.R207C	.	1.264	.		1.12E-05	.	Fam92_f_m_aM_raM	0/0;0/0;0/0;0/1

chr8	143730237	143730237	C	T	FAM83H	exonic	nonsynonymous SNV	FAM83H:NM_198488:exon2:c.G346A:p.G116S	.	1.068	0.0003	0.0005	9.07E-05	Fam115_f_m_aF_aM_aF	0/1;0/0;0/0;0/1;0/0
chr8	143730461	143730461	T	C	FAM83H	exonic	nonsynonymous SNV	FAM83H:NM_198488:exon2:c.A122G:p.E41G	.	1.142	7.34E-05	0.0006	0.0001	Fam63_f_m_aF	0/0;0/1;0/1
chr8	143817128	143817128	G	A	PUF60	exonic	nonsynonymous SNV	PUF60:NM_001271097:exon9:c.C1024T:p.P342S,PUF60:NM_001271096:exon10:c.C1108T:p.P370S,PUF60:NM_001271099:exon10:c.C1075T:p.P359S,PUF60:NM_001271100:exon10:c.C982T:p.P328S,PUF60:NM_014281:exon10:c.C1111T:p.P371S,PUF60:NM_001136033:exon11:c.C1033T:p.P345S,PUF60:NM_001271098:exon11:c.C1159T:p.P387S,PUF60:NM_001362897:exon11:c.C1222T:p.P408S,PUF60:NM_078480:exon11:c.C1162T:p.P388S,PUF60:NM_001362895:exon12:c.C1273T:p.P425S,PUF60:NM_001362896:exon12:c.C1273T:p.P425S	.	1.623	.	.	.	Fam121_f_m_aF_aM	0/1;0/0;0/1;0/1
chr8	143977969	143977969	G	A	PARP10	exonic	nonsynonymous SNV	PARP10:NM_001317895:exon9:c.C2705T:p.T902M,PARP10:NM_032789:exon10:c.C2669T:p.T890M	.	1.667	.	4.84E-05	4.19E-05	Fam38_f_m_aM	0/0;0/1;0/1
chr8	144080276	144080276	G	C	EXOSC4	exonic	nonsynonymous SNV	EXOSC4:NM_019037:exon3:c.G413C:p.C138S	.	1.17	7.34E-05	6.17E-05	3.49E-05	Fam10_f_m_aM_uF	0/0;0/1;0/1;0/0
chr8	144357737	144357737	G	C	FBXL6	exonic	nonsynonymous SNV	FBXL6:NM_012162:exon2:c.C466G:p.L156V,FBXL6:NM_024555:exon2:c.C466G:p.L156V	.	1.783	.	.	.	Fam43_f_m_aM	0/0;0/1;0/1
chr8	144522608	144522608	A	G	LRRC24	exonic	nonsynonymous SNV	LRRC24:NM_001024678:exon5:c.T1409C:p.M470T	.	1.152	.	0.0001	6.99E-06	Fam24_f_m_aM_aM	0/0;0/1;0/1;0/1
chr8	144522767	144522767	G	A	LRRC24	exonic	nonsynonymous SNV	LRRC24:NM_001024678:exon5:c.C1250T:p.T417M	.	1.092	.	.	.	Fam49_f_m_aM	0/0;0/1;0/1
chr8	144523343	144523343	C	A	LRRC24	exonic	nonsynonymous SNV	LRRC24:NM_001024678:exon5:c.G674T:p.R225L	.	1.197	.	6.68E-05	1.40E-05	Fam95_f_m_aM_aM_uF	0/1;0/0;0/1;0/1;0/0
chr8	144545695	144545695	G	A	ARHGAP39	exonic	nonsynonymous SNV	ARHGAP39:NM_025251:exon5:c.C2075T:p.T692M,ARHGAP39:NM_001308208:exon6:c.C2075T:p.T692M,ARHGAP39:NM_001308207:exon7:c.C2075T:p.T692M	.	1.042	7.35E-05	9.84E-05	9.07E-05	Fam28_f_m_aF_uF	0/0;0/1;0/1;0/1
chr8	144808288	144808288	G	T	ZNF517	exonic	nonsynonymous SNV	ZNF517:NM_001317936:exon5:c.G1351T:p.G451W,ZNF517:NM_213605:exon5:c.G1372T:p.G458W	.	1.027	.	0.0001	2.79E-05	Fam73_f_m_aF_dM	0/0;0/1;0/1;0/0
chr9	2096729	2096729	A	G	SMARCA2	exonic	nonsynonymous SNV	SMARCA2:NM_001289396:exon20:c.A2956G:p.I986V,SMARCA2:NM_001289397:exon20:c.A2782G:p.I928V,SMARCA2:NM_003070:exon20:c.A2956G:p.I986V,SMARCA2:NM_139045:exon20:c.A2956G:p.I986V	.	1.272	.	.	.	Fam11_f_m_aM_uM	0/0;0/1;0/1;0/1
chr9	3248121	3248121	G	A	RFX3	exonic	nonsynonymous SNV	RFX3:NM_002919:exon14:c.C1879T:p.R627C,RFX3:NM_001282116:exon15:c.C1879T:p.R627C,RFX3:NM_134428:exon16:c.C1879T:p.R627C	.	2.832	.	.	.	Fam7_f_m_aM_aM_uM	0/1;0/0;0/1;0/1;0/0
chr9	6007766	6007766	C	A	KIAA2026	exonic	stopgain	KIAA2026:NM_001017969:exon1:c.G22T:p.G8X	0.563	.	0.0005	0.0008	0.0004	Fam62_f_m_aM_aM	0/1;0/0;0/1;0/1
chr9	14125763	14125763	A	G	NFIB	exonic	nonsynonymous SNV	NFIB:NM_001282787:exon6:c.T173C:p.M58T,NFIB:NM_001369477:exon6:c.T875C:p.M292T,NFIB:NM_001369479:exon6:c.T392C:p.M131T,NFIB:NM_001369480:exon6:c.T392C:p.M131T,NFIB:NM_001190737:exon7:c.T929C:p.M310T,NFIB:NM_001190738:exon7:c.T1007C:p.M336T,NFIB:NM_001369458:exon7:c.T995C:p.M332T,NFIB:NM_001369459:exon7:c.T995C:p.M332T,NFIB:NM_001369460:exon7:c.T917C:p.M306T,NFIB:NM_001369461:exon7:c.T929C:p.M310T,NFIB:NM_001369462:exon7:c.T995C:p.M332T,NFIB:NM_001369463:exon7:c.T917C:p.M306T,NFIB:NM_001369464:exon7:c.T929C:p.M310T,NFIB:NM_001369465:exon7:c.T902C:p.M301T,NFIB:NM_001369466:exon7:c.T917C:p.M306T,NFIB:NM_001369467:exon7:c.T902C:p.M301T,NFIB:NM_001369468:exon7:c.T995C:p.M332T,NFIB:NM_001369469:exon7:c.T785C:p.M262T,NFIB:NM_001369470:exon7:c.T692C:p.M231T,NFIB:NM_001369471:exon7:c.T929C:p.M310T,NFIB:NM_001369472:exon7:c.T917C:p.M306T,NFIB:NM_001369473:exon7:c.T917C:p.M306T,NFIB:NM_001369474:exon7:c.T914C:p.M305T,NFIB:NM_001369475:exon7:c.T704C:p.M235T,NFIB:NM_001369476:exon7:c.T902C:p.M301T,NFIB:NM_001369478:exon7:c.T692C:p.M231T,NFIB:NM_005596:exon7:c.T929C:p.M310T	.	2.067	.	.	.	Fam82_f_m_aM_uF	0/1;0/0;0/1;0/0
chr9	16583052	16583052	C	T	BNC2	exonic	nonsynonymous SNV	BNC2:NM_001317939:exon3:c.G238A:p.E80K,BNC2:NM_001317940:exon3:c.G79A:p.E27K,BNC2:NM_017637:exon4:c.G364A:p.E122K	.	1.7	7.35E-05	.	1.40E-05	Fam48_f_m_aM_uM	0/0;0/1;0/1;0/0
chr9	21333187	21333187	C	T	KLHL9	exonic	nonsynonymous SNV	KLHL9:NM_018847:exon1:c.G1673A:p.R558H	.	1.418	7.34E-05	3.27E-05	6.98E-06	Fam119_f_m_aM_aM	0/0;0/1;0/1;0/1
chr9	25678111	25678111	T	A	TUSC1	exonic	nonsynonymous SNV	TUSC1:NM_001004125:exon1:c.A211T:p.S71C	.	1.557	.	.	.	Fam105_f_m_aM	0/1;0/0;0/1
chr9	27455489	27455489	A	G	MOB3B	exonic	nonsynonymous SNV	MOB3B:NM_024761:exon2:c.T62C:p.F21S	.	1.563	.	3.27E-05	.	Fam72_f_m_aF_uF	0/1;0/0;0/1;0/0
chr9	32633849	32633849	-	TG	TAF1L	exonic	frameshift insertion	TAF1L:NM_153809:exon1:c.1730_1731insCA:p.Q577Hfs*5	0.308	.	.	.	.	Fam91_m_aM_dM_aM_dM	0/0;0/1;0/0;0/1;0/0
chr9	34371074	34371074	C	T	MYORG	exonic	nonsynonymous SNV	MYORG:NM_020702:exon2:c.G1870A:p.G624S	.	1.219	0.0004	0.0002	0.0001	Fam23_f_m_aM_dF_uFs	0/1;0/0;0/1;0/1;0/0
chr9	34371406	34371406	G	A	MYORG	exonic	nonsynonymous SNV	MYORG:NM_020702:exon2:c.C1538T:p.S513F	.	1.53	.	.	.	Fam71_f_m_aF	0/1;0/0;0/1
chr9	34611034	34611034	C	G	RPP25L	exonic	nonsynonymous SNV	RPP25L:NM_148178:exon2:c.G263C:p.R88P,RPP25L:NM_148179:exon2:c.G263C:p.R88P	.	1.043	.	.	.	Fam112_f_m_aM	0/1;0/0;0/1
chr9	34972448	34972448	C	T	PHF24	exonic	nonsynonymous SNV	PHF24:NM_001304333:exon3:c.C481T:p.R161C,PHF24:NM_001347982:exon3:c.C481T:p.R161C,PHF24:NM_001347983:exon3:c.C481T:p.R161C,PHF24:NM_015297:exon3:c.C481T:p.R161C	.	1.357	0.0003	1.12E-05	6.98E-06	Fam24_f_m_aM_aM	0/0;0/1;0/1;0/1
chr9	34989730	34989730	G	T	DNAJB5	exonic	nonsynonymous SNV	DNAJB5:NM_001135004:exon1:c.G62T:p.R21L,DNAJB5:NM_001349725:exon1:c.G62T:p.R21L	.	1.221	0.0006	.	0.0002	Fam107_f_m_aM	0/0;0/1;0/1
chr9	34989810	34989810	C	T	DNAJB5	exonic	nonsynonymous SNV	DNAJB5:NM_001135004:exon1:c.C142T:p.R48W,DNAJB5:NM_001349725:exon1:c.C142T:p.R48W	.	1.037	.	.	.	Fam56_f_m_aF_aM	0/0;0/1;0/1;0/0
chr9	35664071	35664071	C	T	ARHGEF39	exonic	nonsynonymous SNV	ARHGEF39:NM_032818:exon4:c.G410A:p.R137H	.	1.05	.	1.12E-05	6.98E-06	Fam63_f_m_aF	0/1;0/0;0/1

chr9	35706003	35706003	C	T	TLN1	exonic	nonsynonymous SNV	TLN1:NM_006289:exon41:c.G5470A:p.V1824M	.	1.415	7.35E-05	1.12E-05	1.40E-05	Fam76_f_m_aM_uM	0/1;0/0;0/1;0/1
chr9	35808527	35808527	G	A	NPR2	exonic	nonsynonymous SNV	NPR2:NM_003995:exon19:c.G2731A:p.A911T	.	1.72	7.35E-05	1.12E-05	2.79E-05	Fam105_f_m_aM	0/1;0/0;0/1
chr9	36222884	36222884	T	C	GNE	exonic	nonsynonymous SNV	GNE:NM_001190384:exon7:c.A1196G:p.H399R,GNE:NM_001190388:exon8:c.A1511G:p.H504R,GNE:NM_001128227:exon9:c.A1619G:p.H540R,GNE:NM_005476:exon9:c.A1526G:p.H509R	.	1.004	.	1.12E-05	.	Fam16_f_m_aM_aM	0/0;0/1;0/0;0/1
chr9	37426623	37426623	C	T	GRHPR	exonic	nonsynonymous SNV	GRHPR:NM_012203:exon4:c.C373T:p.R125W	.	1.217	.	3.27E-05	.	Fam74_f_m_aM_uM	0/0;0/1;0/1;0/0
chr9	37426624	37426624	G	A	GRHPR	exonic	nonsynonymous SNV	GRHPR:NM_012203:exon4:c.G374A:p.R125Q	.	1.236	0.0006	0.0007	0.0002	Fam51_f_m_aM_uF	0/0;0/1;0/1;0/1
chr9	37521571	37521571	C	T	FBXO10	exonic	nonsynonymous SNV	FBXO10:NM_012166:exon8:c.G2198A:p.G733E	.	1.148	.	4.59E-05	2.09E-05	Fam12_f_m_aM_uM_aM	0/1;0/0;0/0;0/0;0/1
chr9	37537201	37537201	C	T	FBXO10	exonic	nonsynonymous SNV	FBXO10:NM_012166:exon3:c.G1328A:p.G443D	.	1.153	.	.	.	Fam79_f_m_aM_uM	0/0;0/1;0/1;0/0
chr9	68822643	68822643	C	A	PIP5K1B	exonic	nonsynonymous SNV	PIP5K1B:NM_001278253:exon3:c.C29A:p.A10E,PIP5K1B:NM_003558:exon4:c.C29A:p.A10E	.	1.118	0.0003	0.0002	9.08E-05	Fam82_f_m_aM_uF	0/1;0/0;0/1;0/0
chr9	69467936	69467936	C	T	APBA1	exonic	nonsynonymous SNV	APBA1:NM_001163:exon5:c.G1369A:p.D457N	.	1.056	0.0006	0.0002	5.59E-05	Fam121_f_m_aF_aM	0/0;0/1;0/1;0/1
chr9	70412987	70412987	A	T	KLF9	exonic	nonsynonymous SNV	KLF9:NM_001206:exon1:c.T377A:p.L126H	.	1.998	.	.	.	Fam115_f_m_aF_aM_aF	0/1;0/0;0/0;0/1;0/0
chr9	70413015	70413015	G	C	KLF9	exonic	nonsynonymous SNV	KLF9:NM_001206:exon1:c.C349G:p.P117A	.	1.132	.	7.46E-05	.	Fam85_f_m_aM_aM	0/0;0/1;0/0;0/1
chr9	70603386	70603386	C	T	TRPM3	exonic	nonsynonymous SNV	TRPM3:NM_001366150:exon18:c.G2686A:p.V896I,TRPM3:NM_206944:exon18:c.G2227A:p.V743I,TRPM3:NM_001007471:exon19:c.G2716A:p.V906I,TRPM3:NM_001366141:exon19:c.G2722A:p.V908I,TRPM3:NM_001366143:exon19:c.G2722A:p.V908I,TRPM3:NM_001366149:exon19:c.G2722A:p.V908I,TRPM3:NM_001366151:exon19:c.G2716A:p.V906I,TRPM3:NM_020952:exon19:c.G2257A:p.V753I,TRPM3:NM_206945:exon19:c.G2263A:p.V755I,TRPM3:NM_206947:exon19:c.G2302A:p.V768I,TRPM3:NM_001366142:exon20:c.G2758A:p.V920I,TRPM3:NM_001366145:exon20:c.G2752A:p.V918I,TRPM3:NM_001366146:exon20:c.G2752A:p.V918I,TRPM3:NM_001366148:exon20:c.G2797A:p.V933I,TRPM3:NM_001366154:exon20:c.G2293A:p.V765I,TRPM3:NM_024971:exon20:c.G2293A:p.V765I,TRPM3:NM_206946:exon20:c.G2332A:p.V778I,TRPM3:NM_001366147:exon21:c.G2827A:p.V943I,TRPM3:NM_001366152:exon21:c.G2827A:p.V943I	.	1.706	0.0003	0.0004	2.79E-05	Fam99_f_m_aM_aM	0/0;0/1;0/1;0/1
chr9	72223150	72223150	C	T	GDA	exonic	nonsynonymous SNV	GDA:NM_001242505:exon7:c.C637T:p.R213C,GDA:NM_001242507:exon7:c.C415T:p.R139C,GDA:NM_001351571:exon7:c.C511T:p.R171C,GDA:NM_001351572:exon7:c.C637T:p.R213C,GDA:NM_001351573:exon7:c.C637T:p.R213C,GDA:NM_004293:exon7:c.C637T:p.R213C,GDA:NM_001242506:exon8:c.C415T:p.R139C	.	1.342	7.34E-05	0.0002	5.58E-05	Fam16_f_m_aM_aM	0/0;0/1;0/1;0/0
chr9	74642501	74642501	G	A	RORB	exonic	nonsynonymous SNV	RORB:NM_001365023:exon4:c.G356A:p.R119Q,RORB:NM_006914:exon4:c.G323A:p.R108Q	.	1.734	7.34E-05	4.58E-05	4.19E-05	Fam54_f_m_aM_uF	0/0;0/1;0/1;0/0
chr9	85596397	85596397	C	G	AGTPBP1	exonic	nonsynonymous SNV	AGTPBP1:NM_001286715:exon17:c.G2544C:p.W848C,AGTPBP1:NM_001286717:exon17:c.G2424C:p.W808C,AGTPBP1:NM_001330701:exon18:c.G2388C:p.W796C,AGTPBP1:NM_015239:exon18:c.G2268C:p.W756C	.	1.078	.	1.12E-05	.	Fam65_f_m_aM_uF_dF	0/0;0/1;0/1;0/1;0/1
chr9	85621235	85621235	A	G	AGTPBP1	exonic	nonsynonymous SNV	AGTPBP1:NM_001286715:exon14:c.T2222C:p.I741T,AGTPBP1:NM_001286717:exon14:c.T2102C:p.I701T,AGTPBP1:NM_001330701:exon15:c.T2066C:p.I689T,AGTPBP1:NM_015239:exon15:c.T1946C:p.I649T	.	1.281	.	5.31E-05	4.19E-05	Fam103_f_m_aM	0/0;0/1;0/1
chr9	86018279	86018279	G	A	NAA35	exonic	nonsynonymous SNV	NAA35:NM_001321881:exon20:c.G1798A:p.G600S,NAA35:NM_001321882:exon20:c.G1798A:p.G600S,NAA35:NM_024635:exon20:c.G1798A:p.G600S	.	1.238	.	9.83E-05	.	Fam118_f_m_aM_aF_uM	0/1;0/0;0/0;0/1;0/0
chr9	87706589	87706589	G	T	DAPK1	exonic	nonsynonymous SNV	DAPK1:NM_001288729:exon26:c.G3518T:p.G1173V,DAPK1:NM_001288730:exon26:c.G3518T:p.G1173V,DAPK1:NM_001288731:exon26:c.G3518T:p.G1173V,DAPK1:NM_004938:exon26:c.G3518T:p.G1173V	.	1.302	.	3.27E-05	.	Fam97_f_m_aM_aF	0/1;0/0;0/1;0/1
chr9	89002119	89002119	C	T	S1PR3	exonic	nonsynonymous SNV	S1PR3:NM_005226:exon2:c.C919T:p.R307W	.	1.133	7.35E-05	8.96E-05	3.49E-05	Fam65_f_m_aM_uF_dF	0/1;0/0;0/1;0/0;0/1
chr9	92390033	92390033	T	G	OGN	exonic	nonsynonymous SNV	OGN:NM_014057:exon5:c.A451C:p.T151P,OGN:NM_024416:exon5:c.A625C:p.T209P,OGN:NM_033014:exon5:c.A451C:p.T151P	.	1.162	.	.	.	Fam48_f_m_aM_uM	0/1;0/0;0/1;0/0
chr9	93010271	93010271	C	T	FGD3	exonic	nonsynonymous SNV	FGD3:NM_001083536:exon7:c.C863T:p.T288M,FGD3:NM_001286993:exon7:c.C863T:p.T288M,FGD3:NM_001369951:exon7:c.C863T:p.T288M,FGD3:NM_001369952:exon7:c.C863T:p.T288M,FGD3:NM_033086:exon7:c.C863T:p.T288M	.	1.021	0.0006	7.73E-05	2.79E-05	Fam94_f_m_aM	0/1;0/0;0/1
chr9	93075808	93075808	C	T	SUSD3	exonic	nonsynonymous SNV	SUSD3:NM_001287006:exon2:c.C113T:p.P38L,SUSD3:NM_145006:exon2:c.C113T:p.P38L,SUSD3:NM_001287005:exon3:c.C74T:p.P25L	.	1.252	.	.	.	Fam89_f_m_aM	0/0;0/1;0/1
chr9	94617859	94617859	C	A	FBP1	exonic	nonsynonymous SNV	FBP1:NM_000507:exon3:c.G335T:p.G112V,FBP1:NM_001127628:exon4:c.G335T:p.G112V	.	1.12	.	.	.	Fam39_f_m_aM	0/0;0/1;0/1
chr9	95461924	95461924	C	T	PTCH1	exonic	nonsynonymous SNV	PTCH1:NM_001354918:exon15:c.G2479A:p.D827N,PTCH1:NM_000264:exon16:c.G2635A:p.D879N,PTCH1:NM_001083602:exon16:c.G2437A:p.D813N,PTCH1:NM_001083603:exon16:c.G2632A:p.D878N,PTCH1:NM_001083604:exon16:c.G2182A:p.D728N,PTCH1:NM_001083605:exon16:c.G2182A:p.D728N,PTCH1:NM_001083606:exon16:c.G2182A:p.D728N,PTCH1:NM_001083607:exon16:c.G2182A:p.D728N	.	1.459	7.34E-05	0.0001	4.19E-05	Fam16_f_m_aM_aM	0/1;0/0;0/0;0/1

chr9	95467191	95467191	C	T	PTCH1	exonic	nonsynonymous SNV	PTCH1:NM_001354918:exon14:c.G2329A:p.V777M,PTCH1:NM_000264:exon15:c.G2485A:p.V829M,PTCH1:NM_001083602:exon15:c.G2287A:p.V763M,PTCH1:NM_001083603:exon15:c.G2482A:p.V828M,PTCH1:NM_001083604:exon15:c.G2032A:p.V678M,PTCH1:NM_001083605:exon15:c.G2032A:p.V678M,PTCH1:NM_001083606:exon15:c.G2032A:p.V678M,PTCH1:NM_001083607:exon15:c.G2032A:p.V678M	.	1.472	0.0003	0.0008	0.0002	Fam78_f_m_aF_uM	0/1;0/0;0/1;0/1
chr9	95469007	95469007	C	T	PTCH1	exonic	nonsynonymous SNV	PTCH1:NM_001354918:exon13:c.G1838A:p.R613H,PTCH1:NM_000264:exon14:c.G1994A:p.R665H,PTCH1:NM_001083602:exon14:c.G1796A:p.R599H,PTCH1:NM_001083603:exon14:c.G1991A:p.R664H,PTCH1:NM_001083604:exon14:c.G1541A:p.R514H,PTCH1:NM_001083605:exon14:c.G1541A:p.R514H,PTCH1:NM_001083606:exon14:c.G1541A:p.R514H,PTCH1:NM_001083607:exon14:c.G1541A:p.R514H	.	1.199	.	0.0001	3.49E-05	Fam90_f_m_aF_dM	0/0;0/1;0/1;0/0
chr9	95477632	95477632	C	G	PTCH1	exonic	nonsynonymous SNV	PTCH1:NM_000264:exon10:c.G1418C:p.G473A,PTCH1:NM_001083602:exon10:c.G1220C:p.G407A,PTCH1:NM_001083603:exon10:c.G1415C:p.G472A,PTCH1:NM_001083604:exon10:c.G965C:p.G322A,PTCH1:NM_001083605:exon10:c.G965C:p.G322A,PTCH1:NM_001083606:exon10:c.G965C:p.G322A,PTCH1:NM_001083607:exon10:c.G965C:p.G322A	.	1.557	.	.	.	Fam113_f_m_aF_raM_uF	0/0;0/0;0/0;0/1;0/0
chr9	97664392	97664392	C	T	NCBP1	exonic	nonsynonymous SNV	NCBP1:NM_001351507:exon18:c.C740T:p.A247V,NCBP1:NM_001351504:exon19:c.C1649T:p.A550V,NCBP1:NM_001351505:exon19:c.C1280T:p.A427V,NCBP1:NM_001351506:exon19:c.C1280T:p.A427V,NCBP1:NM_002486:exon19:c.C1850T:p.A617V	.	1.061	.	.	.	Fam115_f_m_aF_aM_aF	0/0;0/1;0/0;0/0;0/1
chr9	98199523	98199523	C	A	TBC1D2	exonic	nonsynonymous SNV	TBC1D2:NM_001267572:exon7:c.G1265T:p.R422L,TBC1D2:NM_001267571:exon13:c.G2645T:p.R882L,TBC1D2:NM_018421:exon13:c.G2612T:p.R871L	.	1.088	.	.	1.40E-05	Fam48_f_m_aM_uM	0/0;0/1;0/1;0/1
chr9	98311174	98311174	C	A	GABBR2	exonic	nonsynonymous SNV	GABBR2:NM_005458:exon14:c.G1925T:p.R642L	.	1.12	.	4.47E-05	6.98E-06	Fam57_f_m_aM_uF	0/1;0/0;0/1;0/0
chr9	99146594	99146594	C	T	TGFBR1	exonic	stopgain	TGFBR1:NM_001130916:exon6:c.C1009T:p.R337X,TGFBR1:NM_001306210:exon7:c.C1252T:p.R418X,TGFBR1:NM_004612:exon7:c.C1240T:p.R414X	0.494	.	.	.	.	Fam107_f_m_aM	0/1;0/0;0/1
chr9	100330054	100330054	A	C	TEX10	exonic	nonsynonymous SNV	TEX10:NM_001161584:exon6:c.T1375G:p.W459G,TEX10:NM_017746:exon6:c.T1366G:p.W456G	.	1.37	.	.	.	Fam69_f_m_aM	0/0;0/1;0/1
chr9	101552453	101552453	C	G	RNF20	exonic	nonsynonymous SNV	RNF20:NM_019592:exon13:c.C1601G:p.A534G	.	1.042	.	.	.	Fam95_f_m_aM_aM_uF	0/0;0/1;0/1;0/1;0/1
chr9	101561950	101561950	A	G	RNF20	exonic	nonsynonymous SNV	RNF20:NM_019592:exon19:c.A2690G:p.K897R	.	1.177	.	.	.	Fam72_f_m_aF_uF	0/1;0/0;0/1;0/0
chr9	105365539	105365539	G	A	SLC44A1	exonic	nonsynonymous SNV	SLC44A1:NM_001286730:exon11:c.G1310A:p.R437H,SLC44A1:NM_001330731:exon11:c.G1310A:p.R437H,SLC44A1:NM_080546:exon11:c.G1310A:p.R437H	.	1.135	0.0002	0.0001	8.39E-05	Fam69_f_m_aM	0/1;0/0;0/1
chr9	105383237	105383237	G	A	SLC44A1	exonic	nonsynonymous SNV	SLC44A1:NM_001286730:exon14:c.G1747A:p.A583T,SLC44A1:NM_001330731:exon14:c.G1747A:p.A583T,SLC44A1:NM_080546:exon14:c.G1747A:p.A583T	.	1.005	.	.	1.40E-05	Fam27_f_m_aF_uM_uM	0/0;0/1;0/1;0/1;0/0
chr9	108855399	108855399	C	T	ACTL7B	exonic	nonsynonymous SNV	ACTL7B:NM_006686:exon1:c.G532A:p.V178M	.	1.109	0.0002	0.0003	0.0001	Fam31_f_m_aM_uF	0/1;0/0;0/1;0/1
chr9	109320221	109320221	A	C	EPB41L4B	exonic	nonsynonymous SNV	EPB41L4B:NM_018424:exon1:c.T226G:p.S76A,EPB41L4B:NM_019114:exon1:c.T226G:p.S76A	.	1.664	0.0002	0.0001	0.0001	Fam106_f_m_aM	0/1;0/0;0/1
chr9	109404512	109404512	A	G	PTPN3	exonic	nonsynonymous SNV	PTPN3:NM_001145372:exon8:c.T893C:p.L298S,PTPN3:NM_001145371:exon9:c.T1028C:p.L343S,PTPN3:NM_001145370:exon13:c.T1361C:p.L454S,PTPN3:NM_001145369:exon14:c.T1496C:p.L499S,PTPN3:NM_001145368:exon18:c.T1754C:p.L585S,PTPN3:NM_002829:exon19:c.T1889C:p.L630S	.	1.005	7.35E-05	2.25E-05	6.98E-06	Fam16_f_m_aM_aM	0/1;0/0;0/0;0/1
chr9	110941978	110941978	C	T	LPAR1	exonic	nonsynonymous SNV	LPAR1:NM_001401:exon3:c.G236A:p.R79H,LPAR1:NM_001351397:exon4:c.G236A:p.R79H,LPAR1:NM_001351403:exon4:c.G236A:p.R79H,LPAR1:NM_001351406:exon4:c.G236A:p.R79H,LPAR1:NM_057159:exon4:c.G236A:p.R79H,LPAR1:NM_001351399:exon5:c.G236A:p.R79H,LPAR1:NM_001351402:exon5:c.G236A:p.R79H,LPAR1:NM_001351404:exon5:c.G236A:p.R79H,LPAR1:NM_001351405:exon5:c.G236A:p.R79H,LPAR1:NM_001351408:exon5:c.G236A:p.R79H,LPAR1:NM_001351411:exon5:c.G236A:p.R79H,LPAR1:NM_001351414:exon5:c.G236A:p.R79H,LPAR1:NM_001351416:exon5:c.G236A:p.R79H,LPAR1:NM_001351417:exon5:c.G236A:p.R79H,LPAR1:NM_001351419:exon5:c.G236A:p.R79H,LPAR1:NM_001351420:exon5:c.G236A:p.R79H,LPAR1:NM_001351398:exon6:c.G236A:p.R79H,LPAR1:NM_001351400:exon6:c.G236A:p.R79H,LPAR1:NM_001351401:exon6:c.G236A:p.R79H,LPAR1:NM_001351407:exon6:c.G236A:p.R79H,LPAR1:NM_001351409:exon6:c.G236A:p.R79H,LPAR1:NM_001351410:exon6:c.G236A:p.R79H,LPAR1:NM_001351412:exon6:c.G236A:p.R79H,LPAR1:NM_001351413:exon6:c.G236A:p.R79H,LPAR1:NM_001351415:exon6:c.G236A:p.R79H,LPAR1:NM_001351418:exon6:c.G236A:p.R79H	.	1.911	7.34E-05	7.46E-05	1.40E-05	Fam87_f_m_aM_uM	0/1;0/0;0/1;0/0
chr9	111631414	111631414	G	T	DNAJC25;DNAJC2	exonic	nonsynonymous SNV	DNAJC25:NM_001015882:exon1:c.G7T:p.A3S,DNAJC25-GNG10:NM_004125:exon1:c.G7T:p.A3S	.	1.977	.	.	.	Fam4_f_m_aM	0/0;0/1;0/1
chr9	113267439	113267439	T	C	CDC26	exonic	nonsynonymous SNV	CDC26:NM_139286:exon4:c.A82G:p.T28A	.	1.086	.	4.65E-05	.	Fam92_f_m_aM_raM	0/0;0/0;0/0;0/1

chr9	114008528	114008528	C	T	ZNF618	exonic	nonsynonymous SNV	ZNF618:NM_001318040:exon8:c.C632T:p.P211L,ZNF618:NM_001318041:exon8:c.C632T:p.P211L,ZNF618:NM_133374:exon8:c.C632T:p.P211L,ZNF618:NM_001318042:exon9:c.C728T:p.P243L	.	1.154	0.0003	6.58E-05	2.09E-05	Fam121_f_m_aF_aM	0/0;0/1;0/1;0/0
chr9	114048867	114048867	C	T	ZNF618	exonic	nonsynonymous SNV	ZNF618:NM_001318040:exon14:c.C1466T:p.A489V,ZNF618:NM_001318041:exon14:c.C1469T:p.A490V,ZNF618:NM_133374:exon14:c.C1286T:p.A429V,ZNF618:NM_001318042:exon15:c.C1565T:p.A522V	.	1.327	7.35E-05	0.0002	3.49E-05	Fam6_f_m_aM	0/0;0/1;0/1
chr9	114504317	114504317	T	C	WHRN	exonic	nonsynonymous SNV	WHRN:NM_001173425:exon1:c.A485G:p.H162R,WHRN:NM_015404:exon1:c.A485G:p.H162R	.	1.786	.	.	.	Fam85_f_m_aM_aM	0/0;0/1;0/0;0/1
chr9	116187799	116187799	G	A	PAPPA	exonic	nonsynonymous SNV	PAPPA:NM_002581:exon2:c.G1061A:p.R354H	.	1.131	.	6.54E-05	.	Fam89_f_m_aM	0/1;0/0;0/1
chr9	116698209	116698209	T	C	TRIM32	exonic	nonsynonymous SNV	TRIM32:NM_001099679:exon2:c.T467C:p.L156P,TRIM32:NM_012210:exon2:c.T467C:p.L156P	.	1.116	0.0001	0.0001	4.89E-05	Fam2_f_m_aF	0/1;0/0;0/1
chr9	120788188	120788188	T	C	FBXW2	exonic	nonsynonymous SNV	FBXW2:NM_012164:exon3:c.A71G:p.Q24R	.	1.698	0.0007	0.001	0.0006	Fam39_f_m_aM	0/1;0/0;0/1
chr9	122858086	122858086	C	T	RC3H2	exonic	nonsynonymous SNV	RC3H2:NM_001100588:exon13:c.G2291A:p.C764Y,RC3H2:NM_001354478:exon13:c.G2291A:p.C764Y,RC3H2:NM_001354482:exon13:c.G2291A:p.C764Y,RC3H2:NM_018835:exon13:c.G2291A:p.C764Y	.	1.005	7.34E-05	0.0001	6.98E-05	Fam19_f_m_aM	0/0;0/1;0/1
chr9	123035533	123035533	T	C	GPR21	exonic	nonsynonymous SNV	GPR21:NM_005294:exon2:c.T967C:p.C323R	.	1.005	.	6.70E-05	4.89E-05	Fam110_f_m_aM_aM_uMs	0/1;0/0;0/1;0/1;0/0
chr9	123101617	123101617	C	T	RABGAP1	exonic	nonsynonymous SNV	RABGAP1:NM_012197:exon25:c.C2941T:p.R981C	.	1.057	0.0003	0.0006	0.0004	Fam70_f_m_aM	0/0;0/1;0/1
chr9	124771058	124771058	A	T	NR6A1	exonic	nonsynonymous SNV	NR6A1:NM_001278546:exon1:c.T62A:p.L21Q,NR6A1:NM_001489:exon1:c.T62A:p.L21Q,NR6A1:NM_033334:exon1:c.T62A:p.L21Q	.	1.135	.	.	4.90E-05	Fam15_f_m_aM_aM	0/0;0/1;0/0;0/1
chr9	125238588	125238588	A	G	HSPA5	splicing	.	.	0.505	.	.	1.12E-05	.	Fam102_f_m_aM	0/0;0/1;0/1
chr9	125354665	125354665	C	T	GAPVD1	exonic	nonsynonymous SNV	GAPVD1:NM_001330778:exon21:c.C3500T:p.P1167L,GAPVD1:NM_001282681:exon22:c.C3518T:p.P1173L,GAPVD1:NM_001330777:exon22:c.C3437T:p.P1146L,GAPVD1:NM_001354294:exon22:c.C3581T:p.P1194L,GAPVD1:NM_001354298:exon22:c.C3581T:p.P1194L,GAPVD1:NM_001354295:exon23:c.C3581T:p.P1194L,GAPVD1:NM_001354300:exon23:c.C3518T:p.P1173L,GAPVD1:NM_015635:exon23:c.C3662T:p.P1221L,GAPVD1:NM_001282679:exon24:c.C3635T:p.P1212L,GAPVD1:NM_001282680:exon24:c.C3581T:p.P1194L,GAPVD1:NM_001354296:exon24:c.C3581T:p.P1194L,GAPVD1:NM_001354297:exon24:c.C3518T:p.P1173L,GAPVD1:NM_001354301:exon24:c.C3581T:p.P1194L,GAPVD1:NM_001354299:exon25:c.C3581T:p.P1194L	.	1.131	.	3.29E-05	.	Fam12_f_m_aM_uM_aM	0/0;0/1;0/1;0/0;0/1
chr9	125657708	125657708	T	A	MAPKAP1	exonic	nonsynonymous SNV	MAPKAP1:NM_001006617:exon4:c.A441T:p.E147D,MAPKAP1:NM_001006618:exon4:c.A441T:p.E147D,MAPKAP1:NM_001006619:exon4:c.A441T:p.E147D,MAPKAP1:NM_024117:exon4:c.A441T:p.E147D	.	1.452	.	2.23E-05	.	Fam21_f_m_aM_uM	0/0;0/1;0/1;0/1
chr9	126327003	126327003	G	A	MVB12B	exonic	nonsynonymous SNV	MVB12B:NM_001011703:exon1:c.G74A:p.R25Q,MVB12B:NM_033446:exon1:c.G74A:p.R25Q	.	1.103	8.21E-05	0.0005	8.01E-05	Fam15_f_m_aM_aM	0/0;0/1;0/0;0/1
chr9	126327003	126327003	G	A	MVB12B	exonic	nonsynonymous SNV	MVB12B:NM_001011703:exon1:c.G74A:p.R25Q,MVB12B:NM_033446:exon1:c.G74A:p.R25Q	.	1.103	8.21E-05	0.0005	8.01E-05	Fam1_f_m_aM	0/0;0/1;0/1
chr9	126880220	126880220	T	C	ZBTB34	exonic	nonsynonymous SNV	ZBTB34:NM_001099270:exon2:c.T809C:p.V270A	.	1.866	.	.	2.10E-05	Fam62_f_m_aM_aM	0/0;0/1;0/1;0/1
chr9	127212684	127212684	G	A	RALGPS1	exonic	nonsynonymous SNV	RALGPS1:NM_001190728:exon14:c.G1285A:p.G429R,RALGPS1:NM_001322323:exon14:c.G1195A:p.G399R,RALGPS1:NM_001366401:exon14:c.G1285A:p.G429R,RALGPS1:NM_001190729:exon15:c.G1285A:p.G429R,RALGPS1:NM_001322320:exon15:c.G1285A:p.G429R,RALGPS1:NM_001322324:exon15:c.G1282A:p.G428R,RALGPS1:NM_001366400:exon16:c.G1411A:p.G471R,RALGPS1:NM_014636:exon16:c.G1411A:p.G471R,RALGPS1:NM_001322321:exon17:c.G1492A:p.G498R,RALGPS1:NM_001322322:exon17:c.G1492A:p.G498R,RALGPS1:NM_001322325:exon17:c.G1495A:p.G499R	.	1.048	.	2.24E-05	6.98E-06	Fam59_f_m_aF_uM	0/0;0/1;0/1;0/0
chr9	127339670	127339670	G	A	GARNL3	exonic	nonsynonymous SNV	GARNL3:NM_032293:exon13:c.G1054A:p.V352I,GARNL3:NM_001286779:exon14:c.G988A:p.V330I	.	1.01	.	0.0001	2.09E-05	Fam88_f_m_aF	0/1;0/0;0/1
chr9	127747179	127747179	G	A	SH2D3C	exonic	nonsynonymous SNV	SH2D3C:NM_001142533:exon4:c.C758T:p.S253F,SH2D3C:NM_001142534:exon4:c.C752T:p.S251F,SH2D3C:NM_001142531:exon5:c.C170T:p.S57F,SH2D3C:NM_001142532:exon5:c.C170T:p.S57F,SH2D3C:NM_005489:exon5:c.C761T:p.S254F,SH2D3C:NM_001252334:exon6:c.C1028T:p.S343F,SH2D3C:NM_170600:exon6:c.C1232T:p.S411F	.	1.287	.	3.28E-05	6.98E-06	Fam10_f_m_aM_uF	0/1;0/0;0/1;0/0
chr9	127890820	127890820	C	T	ST6GALNAC6	exonic	nonsynonymous SNV	ST6GALNAC6:NM_001287000:exon4:c.G419A:p.R140Q,ST6GALNAC6:NM_001287001:exon4:c.G419A:p.R140Q,ST6GALNAC6:NM_001287002:exon4:c.G419A:p.R140Q,ST6GALNAC6:NM_001286999:exon5:c.G521A:p.R174Q,ST6GALNAC6:NM_013443:exon5:c.G521A:p.R174Q	.	1.179	0.0001	0.0002	0.0002	Fam109_f_m_aM	0/0;0/1;0/1
chr9	127921932	127921932	C	G	PIP5KL1	exonic	nonsynonymous SNV	PIP5KL1:NM_173492:exon5:c.G491C:p.R164P,PIP5KL1:NM_001135219:exon10:c.G1100C:p.R367P	.	1.391	.	.	.	Fam22_f_m_aF_aF_uF	0/0;0/1;0/1;0/1;0/0

chr9	128499080	128499080	G	A	ODF2	exonic	nonsynonymous SNV	ODF2:NM_001351581:exon19:c.G2387A:p.S796N,ODF2:NM_001351587:exon19:c.G2069A:p.S690N,ODF2:NM_001351588:exon19:c.G2012A:p.S671N,ODF2:NM_001242352:exon20:c.G2240A:p.S747N,ODF2:NM_001242353:exon20:c.G2255A:p.S752N,ODF2:NM_001351577:exon20:c.G2660A:p.S887N,ODF2:NM_001351578:exon20:c.G2504A:p.S835N,ODF2:NM_001351579:exon20:c.G2396A:p.S799N,ODF2:NM_001351583:exon20:c.G2339A:p.S780N,ODF2:NM_001351584:exon20:c.G2240A:p.S747N,ODF2:NM_001351586:exon20:c.G2183A:p.S728N,ODF2:NM_153433:exon20:c.G2255A:p.S752N,ODF2:NM_153435:exon20:c.G2447A:p.S816N,ODF2:NM_001351580:exon21:c.G2396A:p.S799N,ODF2:NM_001351582:exon21:c.G2372A:p.S791N,ODF2:NM_001351585:exon21:c.G2240A:p.S747N,ODF2:NM_002540:exon22:c.G2183A:p.S728N	.	1.114	0.0005	0.0008	0.0005	Fam28_f_m_aF_uF	0/0;0/1;0/1;0/0
chr9	128499080	128499080	G	A	ODF2	exonic	nonsynonymous SNV	ODF2:NM_001351581:exon19:c.G2387A:p.S796N,ODF2:NM_001351587:exon19:c.G2069A:p.S690N,ODF2:NM_001351588:exon19:c.G2012A:p.S671N,ODF2:NM_001242352:exon20:c.G2240A:p.S747N,ODF2:NM_001242353:exon20:c.G2255A:p.S752N,ODF2:NM_001351577:exon20:c.G2660A:p.S887N,ODF2:NM_001351578:exon20:c.G2504A:p.S835N,ODF2:NM_001351579:exon20:c.G2396A:p.S799N,ODF2:NM_001351583:exon20:c.G2339A:p.S780N,ODF2:NM_001351584:exon20:c.G2240A:p.S747N,ODF2:NM_001351586:exon20:c.G2183A:p.S728N,ODF2:NM_153433:exon20:c.G2255A:p.S752N,ODF2:NM_153435:exon20:c.G2447A:p.S816N,ODF2:NM_001351580:exon21:c.G2396A:p.S799N,ODF2:NM_001351582:exon21:c.G2372A:p.S791N,ODF2:NM_001351585:exon21:c.G2240A:p.S747N,ODF2:NM_002540:exon22:c.G2183A:p.S728N	.	1.114	0.0005	0.0008	0.0005	Fam1_f_m_aM	0/1;0/0;0/1
chr9	128582755	128582755	A	G	SPTAN1	exonic	nonsynonymous SNV	SPTAN1:NM_001130438:exon14:c.A1712G:p.D571G,SPTAN1:NM_001195532:exon14:c.A1712G:p.D571G,SPTAN1:NM_001363759:exon14:c.A1712G:p.D571G,SPTAN1:NM_001363765:exon14:c.A1712G:p.D571G,SPTAN1:NM_003127:exon14:c.A1712G:p.D571G	.	1.526	.	.	.	Fam79_f_m_aM_uM	0/0;0/1;0/1;0/1
chr9	128603568	128603568	T	C	SPTAN1	exonic	nonsynonymous SNV	SPTAN1:NM_001195532:exon27:c.T3545C:p.V1182A,SPTAN1:NM_001363765:exon27:c.T3545C:p.V1182A,SPTAN1:NM_001130438:exon28:c.T3605C:p.V1202A,SPTAN1:NM_001363759:exon28:c.T3605C:p.V1202A,SPTAN1:NM_003127:exon28:c.T3605C:p.V1202A	.	1.032	.	.	.	Fam92_f_m_aM_raM	0/0;0/1;0/1;0/0
chr9	128735415	128735415	G	T	ZER1	exonic	nonsynonymous SNV	ZER1:NM_006336:exon14:c.C2059A:p.L687I	.	1.648	.	.	.	Fam120_f_m_aM_aM	0/0;0/1;0/1;0/0
chr9	128792561	128792561	G	A	TBC1D13	exonic	nonsynonymous SNV	TBC1D13:NM_001286772:exon6:c.G370A:p.D124N,TBC1D13:NM_018201:exon6:c.G370A:p.D124N	.	1.493	0.0003	0.0001	5.58E-05	Fam16_f_m_aM_aM	0/0;0/1;0/0;0/1
chr9	128803439	128803439	G	A	TBC1D13	exonic	nonsynonymous SNV	TBC1D13:NM_018201:exon8:c.G733A:p.D245N	.	1.075	0.0001	0.0002	8.38E-05	Fam112_f_m_aM	0/0;0/1;0/1
chr9	128819107	128819107	C	G	ENDOG	exonic	nonsynonymous SNV	ENDOG:NM_004435:exon1:c.C423G:p.H141Q	.	1.011	.	.	.	Fam54_f_m_aM_uF	0/1;0/0;0/1;0/1
chr9	128945745	128945745	G	T	DOLK	exonic	nonsynonymous SNV	DOLK:NM_014908:exon1:c.C1559A:p.T520N	.	1.229	.	.	.	Fam64_f_m_aM	0/1;0/0;0/1
chr9	128945974	128945974	C	A	DOLK	exonic	nonsynonymous SNV	DOLK:NM_014908:exon1:c.G1330T:p.V444F	.	1.327	.	.	.	Fam27_f_m_aF_uM_uM	0/1;0/0;0/1;0/1;0/0
chr9	129010152	129010152	G	A	SH3GLB2	exonic	nonsynonymous SNV	SH3GLB2:NM_001369915:exon7:c.C643T:p.R215C,SH3GLB2:NM_001287046:exon8:c.C706T:p.R236C,SH3GLB2:NM_020145:exon8:c.C706T:p.R236C,SH3GLB2:NM_001287045:exon9:c.C718T:p.R240C,SH3GLB2:NM_001369913:exon9:c.C751T:p.R251C,SH3GLB2:NM_001369914:exon9:c.C718T:p.R240C	.	1.104	.	7.46E-05	6.98E-06	Fam12_f_m_aM_uM_aM	0/1;0/0;0/1;0/0;0/0
chr9	129100563	129100563	C	G	CRAT	exonic	nonsynonymous SNV	CRAT:NM_000755:exon7:c.G932C:p.G311A,CRAT:NM_001346547:exon7:c.G932C:p.G311A,CRAT:NM_001346549:exon7:c.G812C:p.G271A,CRAT:NM_001257363:exon8:c.G869C:p.G290A,CRAT:NM_001346546:exon8:c.G935C:p.G312A,CRAT:NM_001346548:exon8:c.G869C:p.G290A,CRAT:NM_004003:exon8:c.G869C:p.G290A	.	1.011	.	.	.	Fam12_f_m_aM_uM_aM	0/1;0/0;0/0;0/1;0/1
chr9	129638221	129638221	A	G	ASB6	exonic	nonsynonymous SNV	ASB6:NM_001202403:exon5:c.T748C:p.F250L,ASB6:NM_017873:exon6:c.T835C:p.F279L	.	1.185	0.0001	0.0001	5.58E-05	Fam100_f_m_aF	0/0;0/1;0/1
chr9	129719341	129719341	G	C	PRRX2	exonic	nonsynonymous SNV	PRRX2:NM_016307:exon2:c.G370C:p.E124Q	.	1.232	.	.	.	Fam57_f_m_aM_uF	0/0;0/1;0/1;0/1
chr9	129720701	129720701	G	A	PRRX2	exonic	nonsynonymous SNV	PRRX2:NM_016307:exon3:c.G553A:p.E185K	.	1.237	.	.	6.98E-06	Fam73_f_m_aF_dM	0/0;0/1;0/1;0/1
chr9	129720701	129720701	G	A	PRRX2	exonic	nonsynonymous SNV	PRRX2:NM_016307:exon3:c.G553A:p.E185K	.	1.237	.	.	6.98E-06	Fam92_f_m_aM_raM	0/1;0/0;0/1;0/1
chr9	129804272	129804272	T	G	TOR1B	exonic	nonsynonymous SNV	TOR1B:NM_001317893:exon2:c.T399G:p.S133R,TOR1B:NM_001317894:exon2:c.T399G:p.S133R,TOR1B:NM_014506:exon2:c.T399G:p.S133R	.	1.186	0.0005	0.0004	0.0002	Fam59_f_m_aF_uM	0/0;0/1;0/1;0/0
chr9	129868897	129868897	C	T	USP20	exonic	nonsynonymous SNV	USP20:NM_001008563:exon12:c.C1171T:p.R391C,USP20:NM_001110303:exon12:c.C1171T:p.R391C,USP20:NM_006676:exon12:c.C1171T:p.R391C	.	1.072	0.0003	0.0004	6.28E-05	Fam106_f_m_aM	0/1;0/0;0/1
chr9	130076430	130076430	C	T	GPR107	exonic	nonsynonymous SNV	GPR107:NM_001136557:exon3:c.C274T:p.R92C,GPR107:NM_001136558:exon3:c.C274T:p.R92C,GPR107:NM_020960:exon3:c.C274T:p.R92C	.	1.146	0.0006	0.0005	0.0002	Fam51_f_m_aM_uF	0/0;0/1;0/1;0/1
chr9	130862802	130862802	G	A	ABL1	exonic	nonsynonymous SNV	ABL1:NM_005157:exon4:c.G589A:p.E197K,ABL1:NM_007313:exon4:c.G646A:p.E216K	.	2.472	0.0003	0.0009	0.0003	Fam11_f_m_aM_uM	0/0;0/1;0/1;0/1

chr9	131197620	131197620	-	C	NUP214	exonic	frameshift insertion	NUP214:NM_001318325:exon4:c.605dupC:p.V205Gfs*60,NUP214:NM_001318324:exon29:c.4097dupC:p.V1369Gfs*60,NUP214:NM_005085:exon29:c.4127dupC:p.V1379Gfs*60	0.458	.	.	9.83E-05	2.10E-05	Fam82_f_m_aM_uF	0/1;0/0;0/1;0/1
chr9	131308058	131308058	G	A	PLPP7	exonic	nonsynonymous SNV	PLPP7:NM_032728:exon2:c.G587A:p.R196H	.	1.325	0.0003	0.0001	2.79E-05	Fam79_f_m_aM_uM	0/1;0/0;0/1;0/1
chr9	131507425	131507425	C	A	POMT1	exonic	nonsynonymous SNV	POMT1:NM_001077366:exon4:c.C176A:p.A59D,POMT1:NM_001353194:exon4:c.C176A:p.A59D,POMT1:NM_001353196:exon4:c.C248A:p.A83D,POMT1:NM_001353197:exon4:c.C176A:p.A59D,POMT1:NM_001077365:exon5:c.C338A:p.A113D,POMT1:NM_001136113:exon5:c.C338A:p.A113D,POMT1:NM_001353193:exon5:c.C338A:p.A113D,POMT1:NM_001353198:exon5:c.C176A:p.A59D,POMT1:NM_007171:exon5:c.C338A:p.A113D	.	1.064	.	.	.	Fam89_f_m_aM	0/0;0/1;0/1
chr9	132582843	132582843	C	T	BARHL1	exonic	nonsynonymous SNV	BARHL1:NM_020064:exon1:c.C46T:p.R16C	.	2.237	.	3.28E-05	6.98E-06	Fam84_f_m_aF	0/1;0/0;0/1
chr9	132679603	132679603	C	T	GTF3C4	exonic	nonsynonymous SNV	GTF3C4:NM_012204:exon2:c.C1984T:p.R662C	.	1.578	0.0002	0.0001	0.0001	Fam15_f_m_aM_aM	0/0;0/1;0/0;0/1
chr9	132896566	132896566	C	A	TSC1	exonic	nonsynonymous SNV	TSC1:NM_001162427:exon22:c.G3011T:p.R1004M,TSC1:NM_001362177:exon22:c.G2801T:p.R934M,TSC1:NM_000368:exon23:c.G3164T:p.R1055M,TSC1:NM_001162426:exon23:c.G3161T:p.R1054M	.	1.502	.	.	.	Fam115_f_m_aF_aM_aF	0/1;0/0;0/0;0/1;0/1
chr9	132904429	132904429	C	A	TSC1	exonic	nonsynonymous SNV	TSC1:NM_001162427:exon15:c.G1870T:p.D624Y,TSC1:NM_001362177:exon15:c.G1660T:p.D554Y,TSC1:NM_000368:exon16:c.G2023T:p.D675Y,TSC1:NM_001162426:exon16:c.G2020T:p.D674Y	.	1.578	.	.	.	Fam100_f_m_aF	0/0;0/0;0/1
chr9	133108735	133108735	C	T	RALGDS	exonic	nonsynonymous SNV	RALGDS:NM_001042368:exon5:c.G551A:p.R184H,RALGDS:NM_001271774:exon5:c.G629A:p.R210H,RALGDS:NM_001271775:exon5:c.G713A:p.R238H,RALGDS:NM_001271776:exon5:c.G680A:p.R227H,RALGDS:NM_006266:exon5:c.G716A:p.R239H	.	1.126	.	9.84E-05	4.19E-05	Fam11_f_m_aM_uM	0/1;0/0;0/1;0/0
chr9	133121003	133121003	G	A	RALGDS	exonic	nonsynonymous SNV	RALGDS:NM_001271776:exon1:c.C152T:p.T51M,RALGDS:NM_006266:exon1:c.C152T:p.T51M	.	1.081	.	.	6.98E-06	Fam15_f_m_aM_aM	0/1;0/0;0/1;0/1
chr9	133515501	133515501	A	C	MYMK	exonic	nonsynonymous SNV	MYMK:NM_001080483:exon4:c.T506G:p.F169C	.	1.268	.	3.36E-05	2.79E-05	Fam71_f_m_aF	0/1;0/0;0/1
chr9	133788445	133788445	C	T	VAV2	exonic	nonsynonymous SNV	VAV2:NM_003371:exon14:c.G1301A:p.R434Q,VAV2:NM_001134398:exon15:c.G1316A:p.R439Q	.	1.11	0.0003	0.0004	0.0001	Fam16_f_m_aM_aM	0/0;0/1;0/0;0/1
chr9	134780135	134780135	A	G	COL5A1	exonic	nonsynonymous SNV	COL5A1:NM_000093:exon28:c.A2419G:p.K807E,COL5A1:NM_001278074:exon28:c.A2419G:p.K807E	.	1.205	.	.	.	Fam6_f_m_aM	0/0;0/1;0/1
chr9	136050500	136050500	C	G	NACC2	exonic	nonsynonymous SNV	NACC2:NM_144653:exon2:c.G22C:p.E8Q	.	1.701	.	.	.	Fam9_f_m_aM_dM_uF	0/0;0/1;0/1;0/1;0/0
chr9	136496801	136496801	C	T	NOTCH1	exonic	nonsynonymous SNV	NOTCH1:NM_017617:exon34:c.G6938A:p.R2313Q	.	1.21	0.0001	0.0001	0.0001	Fam13_f_m_aM	0/1;0/0;0/1
chr9	136502033	136502033	C	T	NOTCH1	exonic	nonsynonymous SNV	NOTCH1:NM_017617:exon29:c.G5440A:p.G1814R	.	1.803	.	6.51E-05	.	Fam118_f_m_aM_aF_uM	0/0;0/1;0/1;0/0;0/0
chr9	136502408	136502408	C	T	NOTCH1	exonic	nonsynonymous SNV	NOTCH1:NM_017617:exon28:c.G5248A:p.V1750M	.	1.086	.	7.26E-05	4.89E-05	Fam45_f_m_aM_uF	0/1;0/0;0/1;0/0
chr9	136505865	136505865	G	A	NOTCH1	exonic	nonsynonymous SNV	NOTCH1:NM_017617:exon25:c.C4031T:p.T1344M	.	1.06	0.0006	0.0008	0.0004	Fam85_f_m_aM_aM	0/1;0/0;0/1;0/0
chr9	136506950	136506950	C	T	NOTCH1	exonic	nonsynonymous SNV	NOTCH1:NM_017617:exon23:c.G3667A:p.D1223N	.	1.172	.	2.41E-05	1.40E-05	Fam16_f_m_aM_aM	0/1;0/0;0/1;0/0
chr9	136513116	136513116	T	C	NOTCH1	exonic	nonsynonymous SNV	NOTCH1:NM_017617:exon15:c.A2372G:p.N791S	.	1.025	.	.	.	Fam50_f_m_aF	0/1;0/0;0/1
chr9	136515525	136515525	G	A	NOTCH1	exonic	nonsynonymous SNV	NOTCH1:NM_017617:exon11:c.C1861T:p.R621C	.	1.391	7.35E-05	6.90E-05	2.79E-05	Fam102_f_m_aM	0/0;0/1;0/1
chr9	136517898	136517898	G	A	NOTCH1	exonic	nonsynonymous SNV	NOTCH1:NM_017617:exon8:c.C1295T:p.T432M	.	1.21	0.0001	0.0005	0.0001	Fam62_f_m_aM_aM	0/0;0/1;0/1;0/1
chr9	137010275	137010275	C	T	ABCA2	exonic	nonsynonymous SNV	ABCA2:NM_001606:exon41:c.G6271A:p.G2091S,ABCA2:NM_212533:exon41:c.G6361A:p.G2121S	.	1.718	.	8.77E-05	6.98E-06	Fam78_f_m_aF_uM	0/1;0/0;0/1;0/1
chr9	137011971	137011971	A	G	ABCA2	exonic	nonsynonymous SNV	ABCA2:NM_001606:exon35:c.T5408C:p.M1803T,ABCA2:NM_212533:exon35:c.T5498C:p.M1833T	.	1.842	.	4.56E-05	4.19E-05	Fam68_f_m_aF_uF_uM	0/0;0/1;0/1;0/1;0/0
chr9	137012158	137012158	G	C	ABCA2	exonic	nonsynonymous SNV	ABCA2:NM_001606:exon34:c.C5304G:p.I1768M,ABCA2:NM_212533:exon34:c.C5394G:p.I1798M	.	1.622	.	1.15E-05	.	Fam7_f_m_aM_aM_uM	0/1;0/0;0/1;0/1;0/0
chr9	137012498	137012498	C	T	ABCA2	exonic	nonsynonymous SNV	ABCA2:NM_001606:exon32:c.G5174A:p.R1725H,ABCA2:NM_212533:exon32:c.G5264A:p.R1755H	.	1.856	.	6.58E-05	.	Fam71_f_m_aF	0/1;0/0;0/1
chr9	137012543	137012543	G	A	ABCA2	exonic	nonsynonymous SNV	ABCA2:NM_001606:exon32:c.C5129T:p.S1710L,ABCA2:NM_212533:exon32:c.C5219T:p.S1740L	.	1.209	.	0.0002	.	Fam55_f_m_aM_aM_dM	0/0;0/1;0/0;0/1;0/1
chr9	137013144	137013144	C	A	ABCA2	exonic	nonsynonymous SNV	ABCA2:NM_001606:exon30:c.G4725T:p.M1575I,ABCA2:NM_212533:exon30:c.G4815T:p.M1605I	.	1.563	.	1.27E-05	.	Fam10_f_m_aM_uF	0/0;0/1;0/1;0/0
chr9	137017309	137017309	C	A	ABCA2	exonic	nonsynonymous SNV	ABCA2:NM_001606:exon18:c.G2440T:p.A814S,ABCA2:NM_212533:exon18:c.G2530T:p.A844S	.	2.041	.	.	.	Fam36_f_m_aM_uM	0/0;0/1;0/1;0/0
chr9	137017650	137017650	C	T	ABCA2	exonic	nonsynonymous SNV	ABCA2:NM_001606:exon17:c.G2254A:p.A752T,ABCA2:NM_212533:exon17:c.G2344A:p.A782T	.	2.197	.	.	.	Fam94_f_m_aM	0/1;0/0;0/1
chr9	137031072	137031072	G	A	FUT7	exonic	nonsynonymous SNV	FUT7:NM_004479:exon2:c.C667T:p.R223C	.	1.123	7.34E-05	6.80E-05	2.09E-05	Fam75_f_m_aM	0/1;0/0;0/1
chr9	137070393	137070393	C	G	SAPCD2	exonic	nonsynonymous SNV	SAPCD2:NM_178448:exon1:c.G68C:p.G23A	.	1.131	.	.	.	Fam4_f_m_aM	0/1;0/0;0/1
chr9	137189119	137189119	G	A	SSNA1	exonic	nonsynonymous SNV	SSNA1:NM_003731:exon2:c.G106A:p.E36K	.	1.682	.	.	.	Fam27_f_m_aF_uM_uM	0/0;0/1;0/1;0/1;0/0
chr9	137834904	137834904	A	C	EHMT1	exonic	nonsynonymous SNV	EHMT1:NM_001354263:exon27:c.A3827C:p.E1276A,EHMT1:NM_024757:exon27:c.A3848C:p.E1283A	.	1.451	.	0.0002	8.38E-05	Fam68_f_m_aF_uF_uM	0/0;0/1;0/1;0/1;0/0
chr9	138023642	138023642	C	T	CACNA1B	exonic	nonsynonymous SNV	CACNA1B:NM_000718:exon19:c.C2899T:p.R967W,CACNA1B:NM_001243812:exon19:c.C2899T:p.R967W	.	1.092	7.74E-05	4.04E-05	4.25E-05	Fam66_f_m_aM	0/0;0/1;0/1
chrX	10067293	10067293	C	G	WWC3	exonic	nonsynonymous SNV	WWC3:NM_015691:exon3:c.C23G:p.T8S	.	1.009	.	1.57E-05	.	Fam69_f_m_aM	0/0;0/1;1/1
chrX	12888602	12888602	G	A	TLR7	exonic	nonsynonymous SNV	TLR7:NM_016562:exon3:c.G3094A:p.A1032T	.	1.499	0.0004	0.0007	0.0006	Fam115_f_m_aF_aM_aF	0/0;0/1;0/1;0/0;0/0
chrX	13776249	13776249	C	T	GPM6B	exonic	nonsynonymous SNV	GPM6B:NM_001001994:exon6:c.G649A:p.V217I,GPM6B:NM_001318729:exon6:c.G649A:p.V217I,GPM6B:NM_005278:exon6:c.G706A:p.V236I,GPM6B:NM_001001995:exon7:c.G826A:p.V276I,GPM6B:NM_001001996:exon7:c.G826A:p.V276I	.	1.433	0.0004	0.0004	0.0002	Fam90_f_m_aF_dM	0/0;0/1;0/1;0/0
chrX	18941608	18941608	A	C	PHKA2	exonic	nonsynonymous SNV	PHKA2:NM_000292:exon8:c.T785G:p.I262S	.	1.858	0.0004	0.0003	0.0005	Fam66_f_m_aM	0/0;0/1;1/1

chrX	18990970	18990970	T	C	ADGRG2	exonic	nonsynonymous SNV	ADGRG2:NM_001184835:exon26:c.A2834G:p.K945R,ADGRG2:NM_001079860:exon27:c.A2882G:p.K961R,ADGRG2:NM_001184836:exon27:c.A2876G:p.K959R,ADGRG2:NM_001184837:exon27:c.A2858G:p.K953R,ADGRG2:NM_001079859:exon28:c.A2906G:p.K969R,ADGRG2:NM_001184833:exon28:c.A2900G:p.K967R,ADGRG2:NM_001184834:exon28:c.A2795G:p.K932R,ADGRG2:NM_001079858:exon29:c.A2948G:p.K983R,ADGRG2:NM_005756:exon29:c.A2939G:p.K980R	.	1.128	.	.	.	Fam35_f_m_aF_uM	0/0;0/1;0/1;1/1
chrX	18996068	18996068	C	T	ADGRG2	exonic	nonsynonymous SNV	ADGRG2:NM_001184835:exon24:c.G2585A:p.R862Q,ADGRG2:NM_001079860:exon25:c.G2633A:p.R878Q,ADGRG2:NM_001184836:exon25:c.G2627A:p.R876Q,ADGRG2:NM_001184837:exon25:c.G2609A:p.R870Q,ADGRG2:NM_001079859:exon26:c.G2657A:p.R886Q,ADGRG2:NM_001184833:exon26:c.G2651A:p.R884Q,ADGRG2:NM_001079858:exon27:c.G2699A:p.R900Q,ADGRG2:NM_001184834:exon27:c.G2699A:p.R900Q,ADGRG2:NM_005756:exon27:c.G2690A:p.R897Q	.	1.349	0.0005	0.0004	0.0001	Fam123_f_aF	0/0;0/1
chrX	21655991	21655991	C	T	KLHL34	exonic	nonsynonymous SNV	KLHL34:NM_153270:exon1:c.G1798A:p.D600N	.	1.278	.	1.60E-05	.	Fam114_f_m_aM	0/0;0/1;1/1
chrX	22077535	22077535	C	T	PHEX	exonic	nonsynonymous SNV	PHEX:NM_000444:exon5:c.C496T:p.R166C,PHEX:NM_001282754:exon5:c.C496T:p.R166C	.	1.377	.	5.24E-05	.	Fam29_f_m_aF_uM	0/0;0/1;0/1;0/0
chrX	22077544	22077544	G	A	PHEX	exonic	nonsynonymous SNV	PHEX:NM_000444:exon5:c.G505A:p.V169M,PHEX:NM_001282754:exon5:c.G505A:p.V169M	.	1.162	.	4.64E-05	8.51E-05	Fam88_f_m_aF	0/0;0/1;0/1
chrX	23785707	23785707	A	G	SAT1	exonic	nonsynonymous SNV	SAT1:NM_002970:exon6:c.A367G:p.S123G	.	1.012	0.0004	7.64E-05	2.84E-05	Fam77_f_m_aM_aF_uM_uFs	0/0;0/1;1/1;0/1;1/1;0/0
chrX	41224766	41224766	T	C	USP9X	exonic	nonsynonymous SNV	USP9X:NM_001039590:exon40:c.T6776C:p.F2259S,USP9X:NM_001039591:exon40:c.T6776C:p.F2259S	.	1.563	.	.	.	Fam91_m_aM_dM_aM_dM	0/1;1/1;1/1;1/1;1/1
chrX	41474066	41474066	C	G	NYX	exonic	nonsynonymous SNV	NYX:NM_022567:exon2:c.C613G:p.L205V	.	1.237	.	.	.	Fam51_f_m_aM_uF	0/0;0/1;1/1;0/0
chrX	45051714	45051714	A	T	KDM6A	exonic	nonsynonymous SNV	KDM6A:NM_001291415:exon9:c.A660T:p.K220N,KDM6A:NM_001291416:exon9:c.A660T:p.K220N,KDM6A:NM_001291417:exon9:c.A660T:p.K220N,KDM6A:NM_001291418:exon9:c.A660T:p.K220N,KDM6A:NM_021140:exon9:c.A660T:p.K220N	.	1.884	.	4.37E-05	1.89E-05	Fam118_f_m_aM_aF_uM	0/0;0/1;1/1;0/0;0/0
chrX	47202995	47202995	T	C	UBA1	exonic	nonsynonymous SNV	UBA1:NM_003334:exon12:c.T1286C:p.L429P,UBA1:NM_153280:exon12:c.T1286C:p.L429P	.	1.484	.	1.55E-05	.	Fam12_f_m_aM_uM_aM	0/0;0/1;1/1;1/1;1/1
chrX	47626101	47626101	G	A	CFP	exonic	nonsynonymous SNV	CFP:NM_001145252:exon8:c.C1201T:p.R401C,CFP:NM_002621:exon9:c.C1201T:p.R401C	.	1.914	.	2.33E-05	.	Fam53_f_m_aM	0/0;0/1;1/1
chrX	48601844	48601844	G	T	WDR13	exonic	nonsynonymous SNV	WDR13:NM_001166426:exon5:c.G616T:p.G206W,WDR13:NM_017883:exon6:c.G892T:p.G298W,WDR13:NM_001347217:exon7:c.G892T:p.G298W,WDR13:NM_001347219:exon7:c.G616T:p.G206W	.	2.355	0.0001	0.0002	8.46E-05	Fam15_f_m_aM_aM	0/0;0/1;0/0;1/1
chrX	48808106	48808106	C	T	HDAC6	exonic	nonsynonymous SNV	HDAC6:NM_001321226:exon9:c.C706T:p.R236C,HDAC6:NM_001321227:exon9:c.C706T:p.R236C,HDAC6:NM_001321228:exon9:c.C706T:p.R236C,HDAC6:NM_001321229:exon9:c.C706T:p.R236C,HDAC6:NM_006044:exon9:c.C706T:p.R236C,HDAC6:NM_001321225:exon10:c.C748T:p.R250C	.	2.352	.	.	.	Fam56_f_m_aF_aM	0/0;0/1;0/1;0/0
chrX	50086401	50086401	G	A	CLCN5	exonic	nonsynonymous SNV	CLCN5:NM_000084:exon8:c.G878A:p.R293H,CLCN5:NM_001282163:exon8:c.G938A:p.R313H,CLCN5:NM_001127898:exon11:c.G1088A:p.R363H,CLCN5:NM_001127899:exon11:c.G1088A:p.R363H	.	1.583	0.0001	.	2.86E-05	Fam119_f_m_aM_aM	0/0;0/1;1/1;0/0
chrX	55452768	55452768	C	T	MAGEH1	exonic	nonsynonymous SNV	MAGEH1:NM_014061:exon1:c.C394T:p.P132S	.	1.534	.	.	.	Fam92_f_m_aM_raM	0/0;0/1;1/1;0/0
chrX	65518271	65518271	G	C	LAS1L	exonic	nonsynonymous SNV	LAS1L:NM_001170650:exon10:c.C1466G:p.S489C,LAS1L:NM_001170649:exon11:c.C1592G:p.S531C,LAS1L:NM_031206:exon12:c.C1643G:p.S548C	.	1.057	.	.	.	Fam121_f_m_aF_aM	0/0;0/1;0/1;1/1
chrX	65729651	65729651	G	A	MSN	exonic	nonsynonymous SNV	MSN:NM_002444:exon4:c.G406A:p.D136N	.	1.9	.	0.0003	.	Fam92_f_m_aM_raM	0/0;0/1;1/1;0/0
chrX	69529693	69529693	T	C	FAM155B	exonic	nonsynonymous SNV	FAM155B:NM_015686:exon3:c.T1156C:p.F386L	.	1.201	0.0001	9.97E-05	6.69E-05	Fam34_f_m_aM_uF	0/0;0/1;1/1;0/0
chrX	70429972	70429972	C	T	GDPD2	exonic	nonsynonymous SNV	GDPD2:NM_001171191:exon10:c.C979T:p.R327C,GDPD2:NM_001171193:exon11:c.C979T:p.R327C,GDPD2:NM_001171192:exon12:c.C1216T:p.R406C,GDPD2:NM_017711:exon12:c.C1216T:p.R406C	.	1.317	.	.	.	Fam33_f_m_aM_uM	0/0;0/1;1/1;1/1
chrX	71129381	71129381	A	C	MED12	exonic	nonsynonymous SNV	MED12:NM_005120:exon26:c.A3643C:p.I1215L	.	2.151	.	.	.	Fam10_f_m_aM_uF	0/0;0/0;0/1;0/0
chrX	71224395	71224395	C	T	GJB1	exonic	nonsynonymous SNV	GJB1:NM_000166:exon2:c.C688T:p.R230C,GJB1:NM_001097642:exon2:c.C688T:p.R230C	.	1.252	0.0002	0.0002	0.0002	Fam12_f_m_aM_uM_aM	0/0;0/1;1/1;0/0;1/1
chrX	75424702	75424702	A	T	ZDHHC15	exonic	nonsynonymous SNV	ZDHHC15:NM_001146256:exon7:c.T659A:p.I220N,ZDHHC15:NM_144969:exon8:c.T686A:p.I229N	.	1.068	.	.	.	Fam76_f_m_aM_uM	0/0;0/1;1/1;1/1
chrX	78755174	78755174	G	A	LPAR4	exonic	nonsynonymous SNV	LPAR4:NM_005296:exon2:c.G305A:p.R102H,LPAR4:NM_001278000:exon5:c.G305A:p.R102H	.	1.256	.	1.55E-05	.	Fam56_f_m_aF_aM	0/0;0/1;0/1;0/0
chrX	80691862	80691862	C	T	BRWD3	exonic	nonsynonymous SNV	BRWD3:NM_153252:exon30:c.G3442A:p.E1148K	.	2.003	.	.	.	Fam113_f_m_aF_raM_uF	1/1;0/0;0/1;1/1;0/1
chrX	80717678	80717678	C	G	BRWD3	exonic	nonsynonymous SNV	BRWD3:NM_153252:exon19:c.G2126C:p.R709T	.	2.13	.	.	.	Fam72_f_m_aF_uF	0/0;0/1;0/1;0/1
chrX	100407796	100407796	C	T	PCDH19	exonic	nonsynonymous SNV	PCDH19:NM_001105243:exon1:c.G802A:p.G268S,PCDH19:NM_001184880:exon1:c.G802A:p.G268S,PCDH19:NM_020766:exon1:c.G802A:p.G268S	.	2.367	.	.	.	Fam118_f_m_aM_aF_uM	0/0;0/1;1/1;0/1;1/1
chrX	101241790	101241790	C	T	DRP2	exonic	nonsynonymous SNV	DRP2:NM_001171184:exon5:c.C448T:p.R150W,DRP2:NM_001939:exon7:c.C682T:p.R228W	.	1.237	0.0001	1.55E-05	9.50E-06	Fam56_f_m_aF_aM	0/0;0/1;0/1;0/0
chrX	105767516	105767516	A	G	IL1RAPL2	exonic	nonsynonymous SNV	IL1RAPL2:NM_017416:exon11:c.A1916G:p.H639R	.	1.063	0.0003	4.64E-05	5.71E-05	Fam91_m_aM_dM_aM_dM	0/1;1/1;1/1;1/1;1/1
chrX	106928284	106928284	T	C	CLDN2	exonic	nonsynonymous SNV	CLDN2:NM_001171092:exon2:c.T56C:p.L19S,CLDN2:NM_001171095:exon2:c.T56C:p.L19S,CLDN2:NM_020384:exon2:c.T56C:p.L19S	.	1.193	0.0002	0.0006	0.0001	Fam39_f_m_aM	0/0;0/1;1/1

chrX	106986166	106986166	G	T	MORC4	exonic	nonsynonymous SNV	MORC4:NM_001085354:exon4:c.C343A:p.Q115K,MORC4:NM_024657:exon4:c.C343A:p.Q115K	.	1.083	0.0002	0.0001	4.75E-05	Fam105_f_m_aM	0/0;0/1;1/1
chrX	106986167	106986167	G	C	MORC4	exonic	nonsynonymous SNV	MORC4:NM_001085354:exon4:c.C342G:p.S114R,MORC4:NM_024657:exon4:c.C342G:p.S114R	.	1.151	0.0002	0.0001	4.75E-05	Fam105_f_m_aM	0/0;0/1;1/1
chrX	109665485	109665485	T	C	ACSL4	exonic	nonsynonymous SNV	ACSL4:NM_001318509:exon12:c.A1448G:p.Y483C,ACSL4:NM_001318510:exon12:c.A1325G:p.Y442C,ACSL4:NM_004458:exon12:c.A1325G:p.Y442C,ACSL4:NM_022977:exon13:c.A1448G:p.Y483C	.	1.635	0.0007	0.0007	0.0005	Fam9_f_m_aM_dM_uF	0/0;0/1;1/1;1/1;0/1
chrX	110317627	110317627	G	A	AMMECR1	exonic	nonsynonymous SNV	AMMECR1:NM_001025580:exon1:c.C445T:p.R149W,AMMECR1:NM_015365:exon1:c.C445T:p.R149W,AMMECR1:NM_001171689:exon3:c.C76T:p.R26W	.	1.764	.	.	.	Fam81_f_m_aM_uM	0/0;0/1;1/1;0/0
chrX	111194411	111194411	G	C	PAK3	exonic	nonsynonymous SNV	PAK3:NM_001128167:exon11:c.G1103C:p.C368S,PAK3:NM_001128172:exon11:c.G1166C:p.C389S,PAK3:NM_001128166:exon12:c.G1103C:p.C368S,PAK3:NM_001324325:exon12:c.G1103C:p.C368S,PAK3:NM_001324326:exon12:c.G1103C:p.C368S,PAK3:NM_001324330:exon12:c.G1103C:p.C368S,PAK3:NM_001324328:exon13:c.G1148C:p.C383S,PAK3:NM_001324331:exon13:c.G1103C:p.C368S,PAK3:NM_001324334:exon13:c.G1103C:p.C368S,PAK3:NM_001324327:exon14:c.G1148C:p.C383S,PAK3:NM_001324332:exon14:c.G1103C:p.C368S,PAK3:NM_001324333:exon14:c.G1148C:p.C383S,PAK3:NM_002578:exon14:c.G1103C:p.C368S,PAK3:NM_001128173:exon15:c.G1148C:p.C383S,PAK3:NM_001324329:exon15:c.G1148C:p.C383S,PAK3:NM_001128168:exon16:c.G1211C:p.C404S	.	3.521	.	.	.	Fam79_f_m_aM_uM	0/0;0/1;1/1;1/1
chrX	129565827	129565827	G	A	OCRL	exonic	nonsynonymous SNV	OCRL:NM_000276:exon13:c.G1300A:p.E434K,OCRL:NM_001318784:exon13:c.G1303A:p.E435K,OCRL:NM_001587:exon13:c.G1300A:p.E434K	.	2.017	.	.	.	Fam56_f_m_aF_aM	0/0;0/1;0/0;1/1
chrX	129788035	129788035	G	A	SASH3	exonic	nonsynonymous SNV	SASH3:NM_018990:exon2:c.G118A:p.V40M	.	1.296	.	3.11E-05	5.83E-05	Fam23_f_m_aM_dF_uFs	0/0;0/1;1/1;0/1;0/1
chrX	129793748	129793748	G	C	SASH3	exonic	nonsynonymous SNV	SASH3:NM_018990:exon8:c.G1059C:p.E353D	.	1.248	.	.	.	Fam5_f_m_aM	0/0;0/1;1/1
chrX	134413584	134413584	A	T	PHF6	exonic	nonsynonymous SNV	PHF6:NM_001015877:exon6:c.A512T:p.N171I,PHF6:NM_032335:exon6:c.A515T:p.N172I,PHF6:NM_032458:exon6:c.A512T:p.N171I	.	1.325	.	.	.	Fam39_f_m_aM	0/0;0/1;1/1
chrX	135552001	135552001	G	A	INTS6L	exonic	nonsynonymous SNV	INTS6L:NM_001351601:exon8:c.G914A:p.R305Q,INTS6L:NM_001351603:exon8:c.G914A:p.R305Q,INTS6L:NM_001351604:exon8:c.G914A:p.R305Q,INTS6L:NM_001351605:exon8:c.G914A:p.R305Q,INTS6L:NM_001351606:exon8:c.G320A:p.R107Q,INTS6L:NM_182540:exon8:c.G914A:p.R305Q	.	1.378	.	0.0001	.	Fam21_f_m_aM_uM	0/0;0/1;1/1;0/0
chrX	139802242	139802242	A	C	ATP11C	exonic	nonsynonymous SNV	ATP11C:NM_001010986:exon7:c.T662G:p.L221R,ATP11C:NM_001353811:exon7:c.T653G:p.L218R,ATP11C:NM_001353812:exon7:c.T653G:p.L218R,ATP11C:NM_173694:exon7:c.T662G:p.L221R,ATP11C:NM_001353810:exon8:c.T653G:p.L218R	.	1.623	.	7.62E-05	1.89E-05	Fam47_f_m_aM	0/0;0/1;1/1
chrX	153725316	153725316	G	A	ABCD1	exonic	nonsynonymous SNV	ABCD1:NM_000033:exon1:c.G50A:p.R17H	.	1.404	0.0001	0.0002	0.0001	Fam44_f_m_aM_uF	0/0;0/1;1/1;0/0
chrX	153785963	153785963	G	A	IDH3G	exonic	nonsynonymous SNV	IDH3G:NM_004135:exon13:c.C1091T:p.P364L	.	1.241	.	.	9.41E-06	Fam92_f_m_aM_raM	0/0;0/0;0/0;1/1
chrX	153905849	153905849	G	A	AVPR2	exonic	nonsynonymous SNV	AVPR2:NM_000054:exon3:c.G343A:p.V115M,AVPR2:NM_001146151:exon3:c.G343A:p.V115M	.	1.399	0.001	4.11E-05	2.80E-05	Fam51_f_m_aM_uF	0/0;0/1;1/1;0/1
chrX	153951650	153951650	G	A	HCFC1	exonic	nonsynonymous SNV	HCFC1:NM_005334:exon21:c.C5318T:p.A1773V	.	1.45	.	.	.	Fam53_f_m_aM	0/0;0/1;1/1
chrX	154442150	154442150	C	T	GDI1	exonic	nonsynonymous SNV	GDI1:NM_001493:exon9:c.C1031T:p.A344V	.	2.277	.	.	.	Fam39_f_m_aM	0/0;0/1;1/1
chrX	154450310	154450310	C	G	FAM50A	exonic	nonsynonymous SNV	FAM50A:NM_004699:exon12:c.C1002G:p.D334E	.	1.301	.	.	.	Fam7_f_m_aM_aM_uM	0/0;0/1;1/1;1/1;1/1
chrX	155046562	155046562	G	A	FUNDC2	exonic	nonsynonymous SNV	FUNDC2:NM_023934:exon3:c.G338A:p.G113E	.	1.027	.	.	.	Fam20_f_m_aM_uF	0/0;0/1;1/1;0/0
chrX	155077198	155077198	A	G	BRCC3	exonic	nonsynonymous SNV	BRCC3:NM_001018055:exon4:c.A224G:p.H75R,BRCC3:NM_001242640:exon4:c.A227G:p.H76R,BRCC3:NM_024332:exon4:c.A224G:p.H75R	.	2.221	.	1.56E-05	9.45E-06	Fam16_f_m_aM_aM	0/0;0/1;1/1;1/1
