# Supplementary material for: Genomic analysis of 116 autism families strengthens known risk genes and highlights promising candidates
Source: NPJ Genom Med. 2024 Mar 22;9:21. doi: 10.1038/s41525-024-00411-1 (PMC10959942; doi:10.1038/s41525-024-00411-1)
Supplement: Supplementary file 2 — REPORTING SUMMARY [file 41525_2024_411_MOESM2_ESM.pdf]

nature portfolio

Corresponding author(s): DAPR NPJGENMED-02178R1

Last updated by author(s): Feb 12, 2024

Reporting Summary

Nature Portfolio wishes to improve the reproducibility of the work that we publish. This form provides structure for consistency and transparency in reporting. For further information on Nature Portfolio policies, see our Editorial Policies and the Editorial Policy Checklist.

Statistics

For all statistical analyses, confirm that the following items are present in the figure legend, table legend, main text, or Methods section.

n/a	Confirmed	
☐	☒	The exact sample size (*n*) for each experimental group/condition, given as a discrete number and unit of measurement
☒	☐	A statement on whether measurements were taken from distinct samples or whether the same sample was measured repeatedly
☐	☒	The statistical test(s) used AND whether they are one- or two-sided<br>*Only common tests should be described solely by name; describe more complex techniques in the Methods section.*
☐	☒	A description of all covariates tested
☐	☒	A description of any assumptions or corrections, such as tests of normality and adjustment for multiple comparisons
☐	☒	A full description of the statistical parameters including central tendency (e.g. means) or other basic estimates (e.g. regression coefficient) AND variation (e.g. standard deviation) or associated estimates of uncertainty (e.g. confidence intervals)
☐	☒	For null hypothesis testing, the test statistic (e.g. *F*, *t*, *r*) with confidence intervals, effect sizes, degrees of freedom and *P* value noted<br>*Give P values as exact values whenever suitable.*
☒	☐	For Bayesian analysis, information on the choice of priors and Markov chain Monte Carlo settings
☒	☐	For hierarchical and complex designs, identification of the appropriate level for tests and full reporting of outcomes
☒	☐	Estimates of effect sizes (e.g. Cohen's *d*, Pearson's *r*), indicating how they were calculated

Our web collection on statistics for biologists contains articles on many of the points above.

Software and code

Policy information about availability of computer code

Data collection	No software was used for data collection
Data analysis	PLINK1.9, Burrows–Wheeler Aligner-MEM, Picard v.2.4.1, GATK v.3.5.0, FreeBayes v1.1.0, ANNOVAR, pTDT v1.0.0, AlphaMissense (https://www.science.org/doi/10.1126/science.adg7492), LOFTEE (https://github.com/konradjk/loftee), pext (https://www.nature.com/articles/s41586-020-2329-2)

For manuscripts utilizing custom algorithms or software that are central to the research but not yet described in published literature, software must be made available to editors and reviewers. We strongly encourage code deposition in a community repository (e.g. GitHub). See the Nature Portfolio guidelines for submitting code & software for further information.

Data

Policy information about availability of data

All manuscripts must include a data availability statement. This statement should provide the following information, where applicable:

- Accession codes, unique identifiers, or web links for publicly available datasets
- A description of any restrictions on data availability
- For clinical datasets or third party data, please ensure that the statement adheres to our policy

The genomic and phenotypic data for the families analysed in this study are available by request from dbGAP (dbGaP accession phs002509.v1.p1).

Research involving human participants, their data, or biological material

Policy information about studies with human participants or human data. See also policy information about sex, gender (identity/presentation), and sexual orientation and race, ethnicity and racism.

Reporting on sex and gender	All data analysis was stratified by sex. For all individuals, the self-reported sex matched with genotype data.
Reporting on race, ethnicity, or other socially relevant groupings	No race or ethnicity criteria were used in the study design or analysis.<br>Ancestry was determined based on genotype data using PCA analysis, in order to control for confounding variables.<br>To define rare variants, we used a MAF threshold of 0.1% in gnomADv3_All and gnomAD-v2NonNeuro, including individuals of different ancestries to account for the admixed origin of the non-European families of our cohort.
Population characteristics	The population characteristics are described in supplementary Table S1
Recruitment	The cohort analysed in this study consisted of 116 families with at least one individual with a clinical diagnosis of Autism Spectrum Disorder. All families were recruited by “UOSI Disturbi dello Spettro Autistico” (IRCCS Istituto delle Scienze Neurologiche, Bologna, Italy).
Ethics oversight	Comitato Etico di Area Vasta Emilia Centro (CE-AVEC); code CE14060

Note that full information on the approval of the study protocol must also be provided in the manuscript.

Field-specific reporting

Please select the one below that is the best fit for your research. If you are not sure, read the appropriate sections before making your selection.

☒ Life sciences ☐ Behavioural & social sciences ☐ Ecological, evolutionary & environmental sciences

For a reference copy of the document with all sections, see nature.com/documents/nr-reporting-summary-flat.pdf

Life sciences study design

All studies must disclose on these points even when the disclosure is negative.

Sample size	No sample size calculation was performed as the aim of this study was the clinical and genomic characterization of a defined cohort of 116 ASD families. We did not attempt association analysis requiring large sample sizes, while we focused on the characterization of relevant rare variants.
Data exclusions	We exclude genotyping/sequencing data not fulfilling quality check criteria, described in the Methods section of the manuscript.
Replication	All relevant de novo SNVs identified by NGS were validated by Sanger sequencing. All relevant CNVs identified by SNP-array or whole genome sequencing were validated by Sanger sequencing or real time qPCR.
Randomization	Experimental groups were defined according to clinical diagnosis.
Blinding	NGS and genotyping data collection was blinded to affection status.

Reporting for specific materials, systems and methods

We require information from authors about some types of materials, experimental systems and methods used in many studies. Here, indicate whether each material, system or method listed is relevant to your study. If you are not sure if a list item applies to your research, read the appropriate section before selecting a response.

Materials & experimental systems

n/a	Involved in the study	
☒	☐	Antibodies
☒	☐	Eukaryotic cell lines
☒	☐	Palaeontology and archaeology
☒	☐	Animals and other organisms
☒	☐	Clinical data
☒	☐	Dual use research of concern
☒	☐	Plants

Methods

n/a	Involved in the study	
☒	☐	ChIP-seq
☒	☐	Flow cytometry
☒	☐	MRI-based neuroimaging

Plants

Seed stocks	*Report on the source of all seed stocks or other plant material used. If applicable, state the seed stock centre and catalogue number. If plant specimens were collected from the field, describe the collection location, date and sampling procedures.*
Novel plant genotypes	*Describe the methods by which all novel plant genotypes were produced. This includes those generated by transgenic approaches, gene editing, chemical/radiation-based mutagenesis and hybridization. For transgenic lines, describe the transformation method, the number of independent lines analyzed and the generation upon which experiments were performed. For gene-edited lines, describe the editor used, the endogenous sequence targeted for editing, the targeting guide RNA sequence (if applicable) and how the editor was applied.*
Authentication	*Describe any authentication procedures for each seed stock used or novel genotype generated. Describe any experiments used to assess the effect of a mutation and, where applicable, how potential secondary effects (e.g. second site T-DNA insertions, mosiacism, off-target gene editing) were examined.*
